# NET-GE: a novel NETwork-based Gene Enrichment for detecting biological processes associated to Mendelian diseases

**DOI:** 10.1186/1471-2164-16-S8-S6

**Published:** 2015-06-18

**Authors:** Pietro Di Lena, Pier Luigi Martelli, Piero Fariselli, Rita Casadio

**Affiliations:** 1 Bologna Biocomputing Group, University of Bologna, Italy; 2 Department of Computer Science and Engineering, University of Bologna, Italy; 3 Department of Biology, University of Bologna, Italy

**Keywords:** Network-based enrichment, OMIM, Gene prioritization

## Abstract

**Background:**

Enrichment analysis is a widely applied procedure for shedding light on the molecular mechanisms and functions at the basis of phenotypes, for enlarging the dataset of possibly related genes/proteins and for helping interpretation and prioritization of newly determined variations. Several standard and Network-based enrichment methods are available. Both approaches rely on the annotations that characterize the genes/proteins included in the input set; network based ones also include in different ways physical and functional relationships among different genes or proteins that can be extracted from the available biological networks of interactions.

**Results:**

Here we describe a novel procedure based on the extraction from the STRING interactome of sub-networks connecting proteins that share the same Gene Ontology(GO) terms for Biological Process (BP). Enrichment analysis is performed by mapping the protein set to be analyzed on the sub-networks, and then by collecting the corresponding annotations. We test the ability of our enrichment method in finding annotation terms disregarded by other enrichment methods available. We benchmarked 244 sets of proteins associated to different Mendelian diseases, according to the OMIM web resource. In 143 cases (58%), the network-based procedure extracts GO terms neglected by the standard method, and in 86 cases (35%), some of the newly enriched GO terms are not included in the set of annotations characterizing the input proteins. We present in detail six cases where our network-based enrichment provides an insight into the biological basis of the diseases, outperforming other freely available network-based methods.

**Conclusions:**

Considering a set of proteins in the context of their interaction network can help in better defining their functions. Our novel method exploits the information contained in the STRING database for building the minimal connecting network containing all the proteins annotated with the same GO term. The enrichment procedure is performed considering the GO-specific network modules and, when tested on the OMIM-derived benchmark sets, it is able to extract enrichment terms neglected by other methods. Our procedure is effective even when the size of the input protein set is small, requiring at least two input proteins.

## Background

Next Generation Sequencing (NGS) technologies enable the discovery of large sets of genetic variations characterizing the individual variability. One common problem is to dig out variations potentially related to different phenotypes, including susceptibility to diseases. A widely adopted procedure relies on the extraction of functional information from sets of genes or proteins already associated to the phenotype under investigation: this procedure allows extending the set of genes or proteins potentially associated to the phenotype and can therefore be useful for prioritizing large sets of experimental variations detected with NGS experiments. Functional association is routinely performed by means of statistical enrichment analysis over a gene/protein set of interest (see [1] for a comprehensive review of different approaches). Standard enrichment methods treat each gene/protein as an isolated object and completely neglect the different types of relations among molecules. However, the analysis of genes and proteins in the context of their physical interaction networks, gene regulatory networks, metabolic and signaling pathways can help in extracting new biological information (see [2] for a comprehensive review on the applications of interaction networks to the study of human diseases).

Several approaches exploiting the interaction networks for functional association analysis (network-based enrichment analysis) have emerged in the last few years [3]. These network-based methods can be broadly classified into two main classes: A) methods that use the topology of the interaction network to infer how much similar distinct sets of gene/proteins are (among them, EnrichNET [4], PWEA [5], THINKBack [6], NetPEA [7], PathNet [8], NetGSA [9], SANTA [10], SPIA [11], JEPETTO [12], PathwayExpress[13], DEGraph [14]); B) methods that identify functionally-related modules in interaction networks and then infer protein/gene biological roles from such modules (among them, FunMod [15], PINA [16], MetaCORE [17]). In both classes, graph-theoretic measures and graph properties(such as shortest paths, degree, etc) are commonly used to extract information from the interaction network. Most methods deal with pathway enrichment analysis, some of them with both pathway and Gene Ontology (GO) terms. Among the publicly available tools that perform GO enrichment analysis, EnrichNet [4] and PINA [16] are two of the most cited methods, representative of the A and B classes above, respectively.

PINA (Protein Interaction Network Analysis) is a web resource based on the integration of six protein-protein interaction databases (IntAct [18], MINT [19], BioGRID [20], DIP [21], HPRD [22] and MIPS MPact [23]). The core of PINA consists of a computational pre-analysis of the molecular interaction network aiming at identifying clusters of densely interconnected nodes, which are likely to represent sets of functionally related proteins. Each cluster is annotated, through a standard enrichment analysis, with terms derived from different biological databases (KEGG [24], PFAM [25], GO [26]). Given an input dataset of genes/proteins, they are mapped on the pre-computed clusters and the overrepresented clusters are identified by means of a hypergeometric enrichment test. The input dataset is then characterized by the significantly enriched annotations of the overrepresented clusters. EnrichNet is a web platform for enrichment analysis based on a network integrating different information: molecular interactions (STRING [27]), cellular pathways (KEGG [24], BioCarta [28], WikiPathways [29], REACTOME [30], PID [31]), biological annotations (GO [26], InterPro [32]) and tissue-specific gene expression data. EnrichNet introduces i) a network-based distance between sets of proteins, computed by means of a random walk with a restart procedure; ii) a statistical framework for assessing the significance of distance between two protein sets. These measures allow comparing an input protein set with all the sets of proteins that share the same annotation term on the network. Given an input set, its network-based distances are computed and the annotations corresponding with significantly close sets are retained.

Here we introduce a method for enrichment analysis that implements a novel computational strategy designed to mine and extract information from publicly available interactomics datasets. Our method falls within class B and, similarly to PINA, it is based on a preprocessing phase aimed at identifying interconnected and compact modules in a molecular interaction network. However, differently from all the other approaches in class B, the modules found by our method are function-specific by construction, since they are built starting from seed sets collecting all the proteins related to a specific biological annotation. We make use of graph-theoretic and information-theoretic measures to extend the seed sets into connected subgraphs of a molecular interaction network. Each subgraph represents a compact and function specific module in the interaction network. Our enrichment pipeline consists of two independent analyses: a standard enrichment and a network-based enrichment. The network-based analysis is performed by mapping an input set of proteins into the pre-computed network modules and by collecting the corresponding annotations for an enrichment test. The network-based enrichment allows the detection of statistical associations not directly inferable from the annotations of the starting protein set, and thus not detectable through the standard enrichment. Here, we test the ability of our network-based approach to detect novel biological associations for sets of proteins related to 244 different Mendelian diseases that are associated to two or more proteins, according to the Online Catalog of Human Genes and Genetic Disorders of Mendelian Inheritance in Man (OMIM) [33].

## Methods

### Interaction network and protein annotations

The human protein interaction network was downloaded from STRING [27] (release 9.1). We retained all the links with documented action (file protein.actions.v9.1.txt.gz on the STRING website), irrespectively of the STRING score and of the supporting evidence. The actions associated to the links are activation, binding, catalysis, expression, post-translational modification, and reaction. The resulting network consisting of 16,958 nodes and 457,546 links, summarizes a large variety of interactions types and integrates different large datasets.

All the nodes in STRING were unambiguously mapped onto UniProtKB, using the UniProt id mapping data file [34]. Human proteins in UniProtKB were annotated with Gene ontology (GO) terms for Biological Process (BP) [26,35], as retrieved from the UniProt-GOA web resource [36]. Out of 138,517 human proteins included in UniProtKB, 37,743 are annotated with 12,785 different GO BP terms. A total of 14,056 annotated proteins are mapped on the STRING interactome and a total of 12,621 out of 12,785 GO BP terms are represented in the STRING network. For 8,098 terms, it is possible to extract specific modules from the STRING network, containing a total of 33,315 proteins (see "Module extraction" section for details).

### General workflow of the enrichment pipeline

Given a set of input proteins, our pipeline implements the novel network-based enrichment and a standard one.

The standard enrichment is performed with a Bonferroni-corrected Fisher's exact test to highlight the overrepresented BP terms associated to the input proteins, as annotated in UniProtKB. All the human proteins in UniProtKB with at least one BP annotation are used as background for the Fisher's test (37,743 protein identifiers and 12,785 related BP terms).

The network-based enrichment relies on a preprocessing phase aimed at extracting modules starting from seed sets of proteins sharing the same GO BP annotation. By construction, a module is a compact and connected subgraph of the molecular-interaction network. Given a GO BP term (our reference GO term), the corresponding module contains all the proteins directly annotated with the same term in UniProtKB (seed nodes) and some of their interacting partners (connecting nodes). The module is determined by computing all the shortest paths among the seeds and by reducing the resulting network into the minimal connecting network preserving the distances among seeds. The minimal connecting network adds to the seeds a set of connecting nodes that are more reliably related to the reference GO term. The details of module extraction are given below and the algorithmic description is available in the Additional file 1. The enrichment procedure determines whether there are significant overlaps between the input proteins and the network modules built for each GO BP term. In addition, in the network-based enrichment, the Bonferroni-corrected Fisher's exact test is adopted. The whole set of human proteins in the network-modules is used as background for the Fisher's test (33,315 protein identifiers and 8,098 related GO BP terms).

The output of the pipeline consists of a non-redundant ranking of GO BP terms overrepresented in the input set, ranked according to their Bonferroni-corrected p-values. It is important to notice that with a standard enrichment only GO terms already associated to input proteins can result as overrepresented. On the contrary, the network-based enrichment allows to detect statistical associations with GO terms not included in the annotations of the input protein set. Such terms represent the added-value information of the network-based enrichment analysis.

### Module extraction

**Figure 1:**
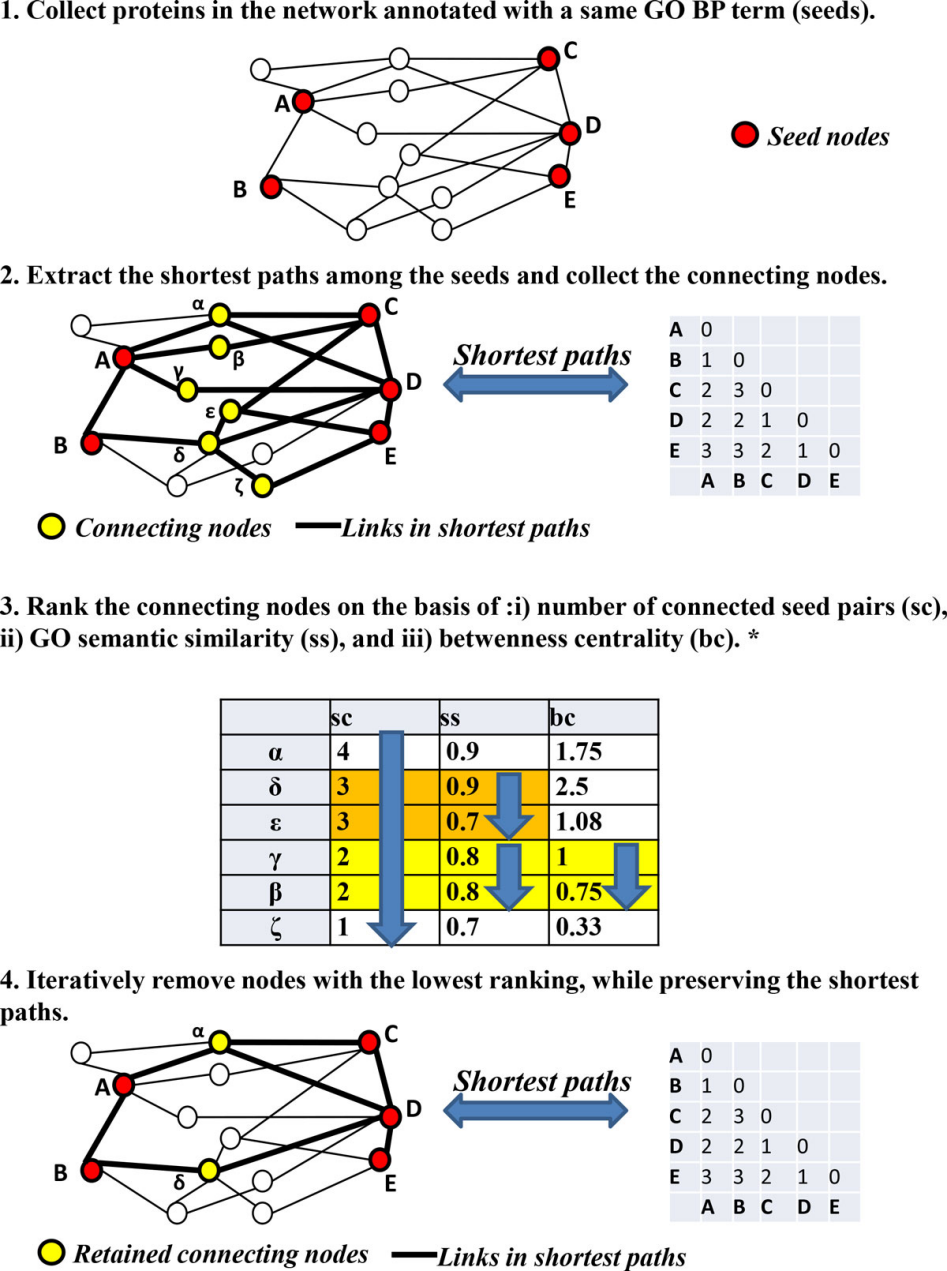
**Outline of the network module generation of NET-GE**. Details on the different steps are explained in Methods. *Ranking scores are hierarchically applied.

The module extraction is schematized in Figure 1 and includes four steps. We extract modules for 8,098 out of 12,621 GO BP terms represented in the STRING network. For each reference GO BP term, all the proteins in the network that are directly annotated with the same term are collected in a seed set (Figure 1, step 1). Each seed set is then extended into a function-specific module, i.e. a compact and connected subgraph of the STRING network. The function-specific module is built in three steps: extraction of the shortest path network (Figure 1, step 2), reduction to the minimal network (Figure 1, steps 3 and 4) and quality filtering, as detailed below.

#### Extraction of the shortest path network

We extract the sub-network of STRING consisting of all the shortest paths between the proteins in the seed set. Seed proteins not appearing in STRING are kept as isolated nodes in the shortest path network. For the shortest paths computation, we do not make use of the edge-scores provided in STRING, i.e. we treat STRING as an undirected and unweighted graph, without self-loops. The size of the shortest path networks extracted from STRING is usually large, even for relatively small input protein sets. On average, the shortest path networks extracted for the different GO BP terms contain 15 times more proteins than their seed sets.

#### Minimal connecting network

Due to the large number of retrieved connecting nodes, a minimization is applied to the shortest path network in order to simplify its topology and thus highlight its main structure. In particular, the computational goal of the procedure is to extract from the shortest path network, the smallest distance-preserving network, i.e. the smallest subgraph that preserves the shortest distances between the seed proteins. The minimization procedure is the most computationally expensive step of the module construction, as it closely resembles the Steiner tree problem [37,38]. Furthermore, the optimal solution is usually not unique. Our implementation makes use of the following heuristic approach:

i) The nodes in the network are split into two disjoint groups: *seed nodes *(i.e. the nodes related to the seed proteins) and *connecting nodes *(i.e. the remaining nodes in the shortest path network).

ii) The connecting nodes are ranked according to three predefined relevance criteria. Their description is detailed in the "Ranking scores" section.

iii) The ranked list is iteratively processed starting from the least important node.

iv) The currently evaluated node is removed from the shortest path network only if its deletion does not increase the shortest distance between any pair of seed nodes.

#### Ranking scores

In the current version, the ranking of a connecting node is obtained by applying three scores (sc, ss, cc), which are used as primary, secondary and tertiary sort key, respectively.

i) *Seed centrality (sc)*. We say that a node connects two seed nodes if it appears in some shortest path connecting them. Thus, the seed centrality measure simply counts the number of distinct seed pairs connected by a node. This property implicitly assumes that the higher the number of seed pairs a node connects, the higher the probability that such node appears in a minimal connecting network.

ii) *Maximum semantic similarity with the reference GO term (ss)*. The semantic similarity measures to which extent the annotation terms of each connecting node is related to the reference GO term: a connecting node with a high semantic similarity score is more likely to be functionally related to the seed nodes. The semantic similarity is defined as the Lin's information-theoretic metric [39]. In detail, we define the maximum semantic similarity of a connecting node with respect to the reference GO term as the highest Lin's score between the GO terms associated to the connecting node/protein and the reference GO term. The background for the information content measure used in Lin's metric is given by the entire set of UniProt-GOA annotations for human proteins [36]. The maximum semantic similarity property explicitly gives more importance to connecting proteins whose annotations are more closely related to the reference GO term (see Additional file 1 for further details).

iii) *Betweenness centrality (bc)*. The betweenness centrality (with respect to the nodes in the seed set) is a measure of centrality of a node in a network [40]. This property is mainly used to assess a local ranking for those connecting nodes that have exactly the same ranking with respect to the previous two properties. In large shortest path networks, this happens quite often, due to the limited range of values of the previous two properties above.

**Figure 2:**
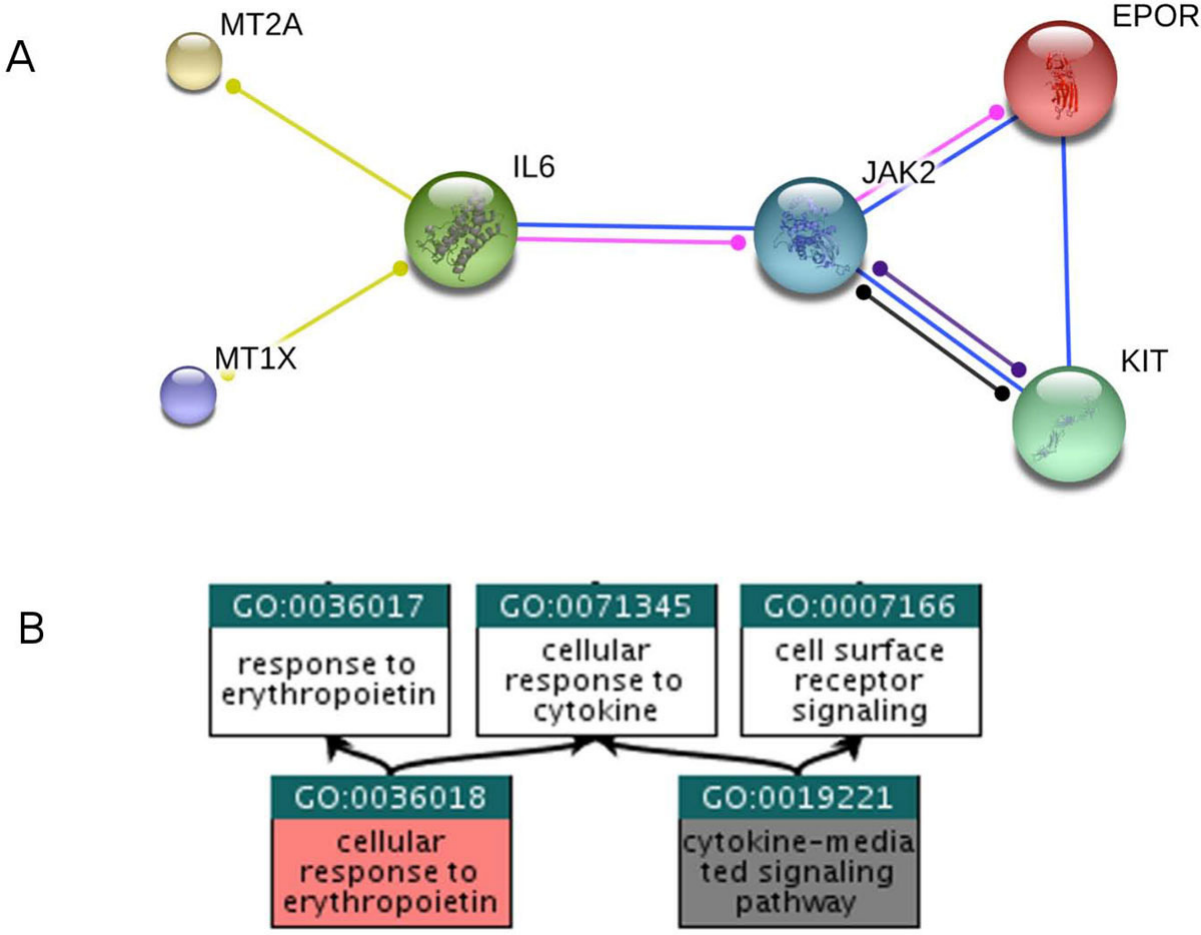
**Minimal connecting network for GO:0036018**. A) Minimal connecting network extracted from STRING 9.2 (http://www.string-db.org) build for the Biological Process term GO:003601 (cellular response to erythropoietin). The seed genes, directly annotated with GO:0036018, are HGNC:MT2A, HGNC:KIT, HGNC:EPOR and HGNC:MT1X. The connecting genes HGNC:JAK2 and HGNC:IL6, recovered by the minimization procedure, are associated to GO:0019221 (cytokine-mediated signaling pathway). B) Relation between the reference GO term (GO:0036018) and the GO associated to the connecting genes (GO:0019221).

As for the shortest path network, seed proteins not appearing in STRING are kept as isolated nodes in the minimal networks. Differently from the shortest path networks, the minimal connecting networks are quite compact. On the average, they contain only 1.5 times more proteins that their seed sets. One example of a shortest path network is provided in Figure 2.

#### Quality filtering

A quality filtering procedure is applied to the minimal connecting networks built in the previous step. The idea is to filter out those networks for which the GO annotations of the connecting nodes are weakly related to the reference GO term. In particular, rare BP terms (i.e. BP terms with few related proteins) tend to produce minimal networks consisting uniquely of long paths. In most of such cases, the annotations of the connecting proteins are unrelated to the reference GO, and then the resulting minimal network is unlikely to include many proteins related to the reference GO. Such network-modules are discarded and not considered for the enrichment. The quality filtering procedure makes use of the maximum semantic similarity measure, as defined above. In particular, a minimal network is retained if, with respect to the reference GO term, the average maximum similarity of the connecting nodes is significantly higher than the average maximum similarity of all the nodes in STRING, as assessed by a Student's t-test with significance set to 5%. The quality test discharges 1,205 networks out of 12,621 (with sizes ranging from 3 to 137 nodes, with an average of 13).

We also filter out minimal networks that do not contain any connecting node. The number of GO BP terms for which we extract a non-trivial network is then 8,098.

### Benchmark set

In order to benchmark the method, we extracted from the OMIM web resource [33] a list of genetic diseases that have been associated to two or more genes. We filtered out all the diseases associated to genes ambiguously mapped on UniProtKB. For performance assessment, we retained only the diseases associated to at least two proteins present in the function-specific network modules. We ended up with a set of 244 genetic diseases. The number of proteins associated to each selected disease ranges from 2 to 29, with an average of 4.

## Results

The annotation pipeline retrieves enriched GO BP terms computed with a standard and a network-based procedure. Both are performed with Bonferroni-corrected Fisher tests, considering a significance level of 5%. We benchmarked on the OMIM-derived benchmark set the level of annotation added by the network-based method from both a quantitative and qualitative point of view. The quantitative analysis highlights the ability of the network-based method in recovering new enriched functions. The qualitative analysis focuses on six cases for which the newly enriched terms add new biological insights, as confirmed by previously published experimental data.

### Quantitative analysis on OMIM diseases

**Figure 3:**
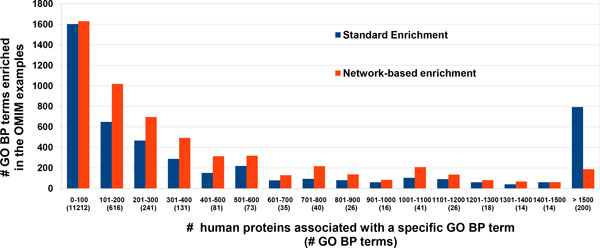
**Number of enriched GO BP terms as a function of the frequency of occurrence in the human proteome**. The x-axis groups GO BP terms based on their frequency of occurrence in the human proteome. The numbers between parentheses indicate the number of GO BP terms falling in each class.

For assessing the power of the network-based enrichment, we focus uniquely on GO BP terms that are not enriched by the standard method (filtering out also all the terms that are ancestors of terms enriched by the standard method). Results are listed in Table 1. In eleven cases out of 244 (5%), neither the standard enrichment nor the network-based enrichment retrieve significantly overrepresented BP term (first row in Table 1). In 143 cases (58%) the network-based enrichment detects more terms than the standard one (last two rows in Table 1). The average number of these terms is 38 per disease. Moreover, in 86 cases (35%) the network-based procedure is able to enrich terms that were not included in the sets of annotations characterizing the input protein set (last row in Table 1). The average number of these new terms is 17. It is also worth noticing that the network-based enrichment returns significant terms in 7 cases out of the 18 where the standard method fails to provide any result (data not shown). 30% of the annotations refer to GO terms that are associated to less than 100 proteins in the human proteome, describing quite specific functions. Terms that are more common are less frequently enriched, mainly owing to the Bonferroni-corrected Fisher test that we applied (see Figure 3). Network-based methods introduce a bias towards terms associated to the most connected nodes (see in our case Figure 1S, Additional file 2). We find that the bias is also present in the case of the standard enrichment procedure that does not make use of the network information (Figure 1S).

**Table 1:** Functional annotation of 244 OMIM diseases with our pipeline.

Annotation*	OMIM diseases°
No significant GO BP terms extracted by SE and NET-GE	11 (5%)

Same significant terms extracted by SE and NET-GE	90 (37%)

NET-GE enriches more terms already included in the annotation of the input proteins	57 (23%)

NET-GE adds new terms not included in the annotation of the input proteins	86 (35%)

*Functional annotation is performed with our network-based procedure (NET-GE) and with a standard enrichment (SE) method. ° number out of the 244 OMIM diseases.

### Qualitative analysis on OMIM diseases

The newly enriched terms that are absent in the original annotations of the input genes are likely to gain new knowledge on the disease at hand. We focus the qualitative analysis on them and we detail here six case studies for which experimental validations are available for the annotations derived with our method. For all the reported cases, PINA does not return any significant association. EnrichNet enriches only terms that are already included in the annotations of the input proteins. However EnrichNet is best suited to analyze sets including at least 10 proteins, while in our case studies, four out of six cases consist of input sets comprising two to four proteins.

#### OMIM #133100 ERYTHROCYTOSIS, FAMILIAL, 1

The disease is characterized by the increase of the red blood cell mass and hemoglobin concentration, and by hypersensitivity of erythrocyte progenitors (myeloid cells) to erythropoietin. The disease is associated to three genes (the tyrosine-protein kinase HGNC: JAK2, the SH2B adapter protein HGNC: SH2B3, and the erythropoietin receptor HGNC: EPOR) and the standard method enriches two terms related to histone phosphorylation. NET-GE adds three terms, reported in Table 2 (see file OMIM133100.pdf in Additional file 3 for the complete annotation). Two are already present in the set of annotations of the input proteins and are related to the response to erythropoietin (one of these terms is shown in Figure 2). The novel term is related to the regulation of myeloid cell apoptosis. Interestingly enough, the involvement of this last process is reported in [41].

**Table 2:** GO BP terms enriched with NET-GE for OMIM disease #133100 (FAMILIAL ERYTHROCYTOSIS 1).

Biological Process GO Term	Description	Bonferroni corrected p-value
GO:0036017	response to erythropoietin	8.1·10^-5^

GO:0036018	cellular response to erythropoietin	8.1·10^-5^

GO:0033033*	negative regulation of myeloid cell apoptotic process	2.5·10^-3^

*GO BP term not directly associated to the input proteins.

#### OMIM #143465 ATTENTION DEFICIT-HYPERACTIVITY DISORDER; ADHD

ADHD is a psychiatric disease related to the development of the nervous system in children and adolescents. It has been linked to variations in dopamine receptors HGNC: DRD4 and HGNC: DRD5. Standard enrichment is able to highlight the connection between the disorder and the dopaminergic pathway, including the second messenger intracellular pathway based on cAMP. It also enriches GO-terms related to psychiatric functions (cognition, learning) and to the response to several compounds (amphetamine, cocaine, alkaloids, ammonium ion). As reported in Table 3 the network-based procedure enriches several terms already present in the annotation of the two input proteins (see file OMIM143465.pdf in Additional file 3 for the complete annotation). In addition, in this case, they refer to behavioral characters (response to fear, stress and defense) or to the response to chemical compounds (histamine). More interestingly, new terms are enriched, highlighting processes unexpectedly involved in ADHD such as the regulation of the GABAergic pathway and the transport of aminoacids. Both these processes, although non-characterizing the input proteins, have been experimentally related to ADHD in [42] and [43].

**Table 3:** GO BP terms enriched with NET-GE for OMIM disease #143465 (ATTENTION DEFICIT-HYPERACTIVITY DISORDER; ADHD).

Biological Process GO Term	Description	Bonferroni corrected p-value
GO:0014052*	regulation of gamma-aminobutyric acid secretion	1.1·10^-4^

GO:0034776	response to histamine	1.6·10^-4^

GO:0051954	positive regulation of amine transport	1.9·10^-3^

GO:0001662	behavioral fear response	2.8·10^-3^

GO:0032228*	regulation of synaptic transmission, GABAergic	4.7·10^-3^

GO:0050805	negative regulation of synaptic transmission	6.0·10^-3^

GO:0060078	regulation of postsynaptic membrane potential	2.0·10^-2^

GO:0098661*	inorganic anion transmembrane transport	4.2·10^-2^

*GO BP term not directly associated to the input proteins.

#### OMIM #188890 TOBACCO ADDICTION, SUSCEPTIBILITY TO

Susceptibility to tobacco addiction has been linked to four proteins (the dopamine transporter HGNC: SLC6A3, the GABAergic G-protein coupled receptor HGNC: GABBR2, the cholinergic receptor HGNC: CHRNA4 and the cytochrome P450 HGNC: CYP2A6). Standard procedure enriches the processes related to intercellular signaling and to the response to nicotine and to alkaloids. Network-based enrichment (Table 4) is able to highlight processes such as "social behavior" and "intraspecies interactions between organisms" that are relevant for tobacco addiction and that were not present among the annotations characterizing the four starting proteins (see file OMIM188890.pdf in Additional file 3 for the complete annotation). Moreover, a GO term referring to the response to cocaine is enriched. Connections between nicotine consumption and response to cocaine have been recently described at the molecular level [44].

**Table 4:** GO BP terms enriched with NET-GE for OMIM disease #188890 (SUSCEPTIBILITY TO TOBACCO ADDICTION).

Biological Process GO Term	Description	Bonferroni corrected p-value
GO:0035176*	social behavior	3.8·10^-2^

GO:0051703*	intraspecies interaction between organisms	3.8·10^-2^

GO:0042220	response to cocaine	4.3·10^-2^

*GO BP term not directly associated to the input proteins.

#### OMIM #188050 THROMBOPHILIA DUE TO THROMBIN DEFECT; THPH1

THPH1 is a disorder of impaired clot formation linked to four different proteins: the coagulation factor HGNC: F13A1, the prothrombin HGNC: F2, the methylenetetrahydrofolate reductase HGNC: MTHFR, and the hyaluronan-binding protein HGNC: HABP2. Standard enrichment only extracts a GPCR signaling pathway, while the network based procedure is able to correctly identify the main impaired process, namely the platelet aggregation (Table 5 see file OMIM188050.pdf in Additional file 3 for the complete annotation). This term is more specific than those reported in the annotation of the input proteins.

**Table 5:** GO BP terms enriched with NET-GE for OMIM disease #188050 (THROMBOPHILIA DUE TO THROMBIN DEFECT; THPH1).

Biological Process GO Term	Description	Bonferroni corrected p-value
GO:0070527*	platelet aggregation	3.0·10^-2^

*GO BP term not directly associated to the input proteins.

#### OMIM #608446 SUSCEPTIBILITY TO MYOCARDIAL INFARCTION

The susceptibility to myocardial infarction is linked to 12 different proteins (see file OMIM608446.pdf in Additional file 3). Both standard and network based enrichment extract different terms, already associated to the input proteins. However, NET-GE is able to add two new important processes related to the disease: regulation of angiogenesis and regulation of vasculature development (Table 6).

**Table 6:** GO BP terms enriched with NET-GE for OMIM disease #608446 (SUSCEPTIBILITY TO MYOCARDIAL INFARCTION).

Biological Process GO Term	Description	Bonferroni corrected p-value
GO:0045765*	regulation of angiogenesis	6.0·10^-3^

GO:1901342*	regulation of vasculature development	9.0·10^-3^

*only GO BP term not directly associated to the input proteins are reported

#### OMIM #601665 OBESITY

Obesity is linked to 16 different proteins (see file OMIM 601665.pdf in Additional file 3). Both the standard and the network based enrichment extract many terms, already associated to the input proteins. NET-GE is able to newly enrich several processes listed in table 7. Most of these processes are known to be related to obesity. In particular, the most specific ones are: i) the sodium ion homeostasis [45]; ii) the CD4-positive, alpha-beta T cell differentiation/activation [46]; iii)the negative regulation of bile acid biosynthetic process [47]; iv) the regulation of adrenergic receptor signaling pathway [48]; v) the regulation of serotonin secretion [49]; vi) the inflammatory response [50]; vii) the negative regulation of cAMP-mediated signaling [51].

**Table 7:** GO BP terms enriched with NET-GE for OMIM disease #601665 (OBESITY).

Biological Process GO Term	Description	Bonferroni corrected p-value
GO:0055078*	sodium ion homeostasis	3.0·10^-3^

GO:0048468*	cell development	7.0·10^-3^

GO:0055067*	monovalent inorganic cation homeostasis	1.0·10^-2^

GO:0007492*	endoderm development	1.0·10^-2^

GO:0043367*	CD4-positive, alpha-beta T cell differentiation	1.8·10^-2^

GO:0035710*	CD4-positive, alpha-beta T cell activation	2.2·10^-2^

GO:0070858	negative regulation of bile acid biosynthetic process	2.3·10^-2^

GO:0019935*	cyclic-nucleotide-mediated signaling	2.4·10^-2^

GO:0071877*	regulation of adrenergic receptor signaling pathway	3.8·10^-2^

GO:0014062*	regulation of serotonin secretion	4.1·10^-2^

GO:0048806*	genitalia development	4.1·10^-2^

GO:0006954*	inflammatory response	4.1·10^-2^

GO:0043951*	negative regulation of cAMP-mediated signalling	4.4·10^-2^

GO:0050994*	regulation of lipid catabolic process	4.9·10^-2^

*only GO BP term not directly associated to the input proteins are reported

## Conclusions

We describe a novel computational method, NET-GE, for enrichment analysis, which exploits the information contained into molecular interaction networks. Given a set of input proteins, our method can detect functional associations not directly inferable from the annotations of the starting protein set, and thus not detectable through a standard enrichment. The method has been benchmarked on a set of 244 different Mendelian diseases associated to more than two proteins, as reported in the OMIM database. The lists of enriched terms for the benchmark examples are available in Additional file 3. NET-GE is able to enrich terms neglected by the standard method and, in a considerable amount of cases, the terms are not even included in the annotation of the input set. For some diseases, it is possible to prove that new enrichment terms are coherent with the experimental information available for the diseases. Therefore, we propose our novel network-based enrichment as a procedure helping in formulating new hypotheses on the biological processes underlying a particular phenotype for which a pool of associated proteins is known. Enriched GO-terms can suggest pools of new proteins potentially associated to the phenotype at hand and can therefore help the prioritization of new variants to be discovered with sequencing techniques. One of the advantages of our method, with respect to other similar ones, is its ability to extract new information even from very small sets of input proteins. In the current version, the network-based method makes use of the STRING network of physical interactions and analyzes only the GO BP annotations. However, the method is quite general and it does not rely on such specific interaction network and biological annotations. For future development, we plan to extend it to different networks and different biological annotations.

## Competing interests

The authors declare they have no conflict of interests in relation to this VarISIG issue article.

## Authors' contributions

PDL, PLM, PF and RC planned the research and discussed the result; PDL implemented the computational method; PLM and RC analyzed the biological test sets. All the authors contributed to writing and correcting the paper.

## Supplementary Material

Additional file 1 **Details on the method implementation**. Click here for file

Additional file 2 **Figure S1: Number of enriched GO BP terms as a function of the maximum degree of the human proteins annotated with a the same term**. Click here for file

Additional file 3 **Detailed results for the OMIM-derived benchmark set**. The archive contains pdf documents listing the enriched terms for each one of the 244 diseases in the OMIM-derived benchmark set. Click here for file
