# Supplementary material for: NET-GE: a novel NETwork-based Gene Enrichment for detecting biological processes associated to Mendelian diseases
Source: BMC Genomics. 2015 Jun 18;16(Suppl 8):S6. doi: 10.1186/1471-2164-16-S8-S6 (PMC4480278; doi:10.1186/1471-2164-16-S8-S6)
Supplement: Additional file 1 — Details on the method implementation. [file 1471-2164-16-S8-S6-S1.pdf]

SUPPLEMENTARY MATERIALS

NET-GE: a novel NETwork-based Gene Enrichment for detecting biological processes associated to Mendelian diseases

Pietro Di Lena, Pier Luigi Martelli, Piero Fariselli, Rita Casadio

1 General workflow of the enrichment analysis

Given a set of input proteins, our pipeline implements the novel network-based enrichment and a standard one. The standard enrichment is performed with a Bonferroni-corrected Fishers exact test to highlight the overrepresented BP terms associated to the input proteins, as annotated in UniProtKB. All the human proteins in UniProtKB with at least one BP annotation are used as background for the Fishers test (37,743 protein identifiers and 12,785 related BP terms). The network-based enrichment relies on a preprocessing phase aimed at extracting modules starting from seed sets of proteins sharing the same GO BP annotation. A module by construction is a compact and connected subgraph of the molecular-interaction network. Given a GO BP term (our reference GO term), the corresponding module contains all the proteins directly annotated with the same term in UniProtKB (seed nodes) and some of their interacting partners (connecting nodes). The module is determined by computing all the shortest paths among the seeds and by reducing the resulting network into the minimal connecting network preserving the distances among seeds. The minimal connecting network adds to the seeds a set of connecting nodes that are more reliably related to the reference GO term. The details of module extraction are given below. The enrichment procedure determines whether there are significant overlaps between the input proteins and the network modules built for each GO BP term. Also in the network based enrichment, the Bonferroni-corrected Fishers exact test is adopted. The whole set of human proteins in the network-modules is used as background for the Fishers test (33,315 protein identifiers and 8,098 related GO BP terms). The output of the pipeline consists of a non-redundant ranking of GO BP terms overrepresented in the input set, ranked according to their Bonferroni-corrected p-values. It is important to notice that with a standard enrichment only GO terms already associated to input proteins can result as overrepresented. On the contrary, the network-based enrichment allows to detect statistical associations with GO terms not included in the annotations of the input protein set. Such terms represent the added-value information of the network-based enrichment analysis.

The entire pipeline is summarized with pseudo-code in Alg. 1 and Alg. 2.

Algorithm 1 Enrichment pipeline

procedure ENRICHMENT-PIPELINE($P, List_{GOA}, List_{STRING}, th$)
    ▷ P is a set of UniProtAC identifiers.
    ▷ $List_{GOA}$ is a collection of sets of proteins. Each set is related to the same GO term.
    ▷ $List_{STRING}$ is a collection of sets of proteins. Each set is related to the same GO term.
    ▷ th is the P-value threshold.
    $E_{GOA} \leftarrow$ ENRICHMENT($P, List_{GOA}, th$)
    $E_{STRING} \leftarrow$ ENRICHMENT($P, List_{STRING}, th$)
    for each $(L, GO, Pvalue) \in E_{GOA}$ **do**
        PRINT-REPORT(P,L,GO,Pvalue)
    end for
    for each $(L, GO, Pvalue) \in E_{STRING}$ **do**
        if GO is not in E_{GOA} **then**
            PRINT-REPORT(P,L,GO,Pvalue)
        end if
    end for
end procedure

Algorithm 2 Enrichment analysis

function ENRICHMENT($P, List, th$)
    ▷ P is a set of UniProtAC identifiers.
    ▷ $List = \{L1, ..., L_N\}$ is a collection of sets of proteins. Each set is related to the same GO term.
    ▷ th is the p-value threshold.
    $E \leftarrow \emptyset$ ▷ List of enriched terms
    $m \leftarrow 0$ ▷ Total number of distinct proteins in *List*.
    $n \leftarrow 0$ ▷ Number of comparisons for Bonferroni's correction.
    $tmp \leftarrow \emptyset$
    for *each* $L \in List$ ***do***
        $tmp \leftarrow tmp \cup L$
    end for
    $m \leftarrow |tmp|$
    for *each* $L \in List$ ***do***
        if $P \cap L \neq \emptyset$ ***then***
            $n \leftarrow n + 1$
        end if
    end for
    for *each* $L \in List$ ***do***
        $Pvalue \leftarrow$ FISHER-TEST($|P \cap L|, |P|, |L|, m$)
        if $Pvalue \cdot n \leq th$ ***then***
            $GO \leftarrow$ GOTERM-ID(L) ▷ *Gets the GO id associated to set L*
            ADD($E, (L, GO, Pvalue \cdot n)$)
        end if
    end for
    SORT*(E)* ▷ *Sort the list of enriched terms wrt Pvalue.*
    return E
end function

2 Modules extraction

We extract modules for 8,098 out of 12,621 GO BP terms represented in the STRING network. For each reference GO BP term, all the proteins in the network that are directly annotated with the same term are collected in a seed set . Each seed set is then extended into a function-specific module, i.e. a compact and connected subgraph of the STRING network. The function-specific module is built in three steps: extraction of the shortest path network, reduction to the minimal network and quality filtering, as detailed below

2.1 Extraction of the shortest path network

We extract the sub-network of STRING consisting of all the shortest paths between the proteins in the seed set (see Alg. 3). Recall that, given a GO term t, we define a seed set as the set of proteins that are annotated in GOA with term t. Seed proteins not appearing in STRING are kept as isolated nodes in the shortest path network.

Algorithm 3 Shortest Path Network

function SPN($G, \mathcal{S}$)
    ▷ $G = (V, E)$ is a graph (STRING)-
    ▷ $\mathcal{S}$ is the set of seed nodes.
    $V' \leftarrow \emptyset$
    $E' \leftarrow \emptyset$
    for *each* u, v *in* $\mathcal{S} \cap V$ ***do***
        ▷ *Extract the subgraph* $G'' = (V'', E'')$ *of all the shortest paths between* u, v *in* G.
        $G'' \leftarrow \mathrm{SP}(G, u, v)$
        $V' \leftarrow V' \cup V''$
        $E' \leftarrow E' \cup E''$
    end for
    $V' \leftarrow V' \cup \mathcal{S}$ ▷ *Add to* V' *seed nodes not appearing in* G.
    return $G' = (V', E')$
end function

For the shortest paths computation (SP procedure in Alg. 3), we do not make use of the edge-scores provided in STRING, i.e. we treat STRING as an undirected and unweighted graph, without self-loops. The size of the shortest path networks extracted from STRING is usually large, even for relatively small input protein sets. On average, the shortest path networks extracted for the different BP GO terms contain 15 times more proteins than their seed sets.

2.2 Minimal connecting network

Due to the large number of retrieved connecting nodes, a minimization is applied to the shortest path network in order to simplify its topology, and thus highlight its main structure. In particular, the computational goal of the minimization procedure is to extract from the shortest path network the smallest distance-preserving network, i.e. the smallest subgraph that preserves the shortest distances between the seed proteins. We call such subgraph *minimal connecting network*.

We can formalize the definition of *minimal connecting network* as follows:

Definition 2.1 Let $G = (V, E)$ be a shortest path network and let $\mathcal{S} \subseteq V$ denote the set

of seed nodes in G. A *distance-preserving subnetwork* in G is a subgraph $G' = (V', E')$ such that

(1) $\mathcal{S} \subseteq V' \subseteq V$ and $E' \subseteq E$ (i.e., G' is a subgraph of G, containing all seed nodes in $\mathcal{S}$)

(2) $\forall u, v \in \mathcal{S}$, $d_G(u, v) = d_{G'}(u, v)$ (i.e. the shortest path length between all pair of seed nodes is the same in both G and G'), where $d_G(u, v)$ denotes the shortest path length between u and v in G.

We say that a distance-preserving subnetwork G' is a *minimal connecting network* if V' is the smallest possible set that is consistent with properties (1) and (2). □

The minimization procedure of a shortest path network is the most computationally expensive step of the module construction, as it closely resembles the Steiner tree problem [1]. Furthermore, the optimal solution is usually not unique. Our implementation makes use of the following heuristic approach (see Alg. 4):

i) The nodes in the network are split into two disjoint groups: seed nodes (i.e. the nodes related to the seed proteins) and connecting nodes (i.e. the remaining nodes in the shortest path network). Line 4 in Alg. 4.

ii) The connecting nodes are ranked according to three predefined relevance criteria. Line 6 in Alg. 4. Their description is detailed in the Ranking scores section.

iii) The ranked list is iteratively processed starting from the least important node. Lines 7-13 in Alg. 4.

iv) The currently evaluated node is removed from the shortest path network only if its deletion does not increase the shortest distance between any pair of seed nodes. Lines 10-12 in Alg. 4.

Algorithm 4 Minimal Connecting Network

1: **function** MCN($G, \mathcal{S}$)
2:     ▷ $G = (V, E)$ is a shortest path network.
3:     ▷ $\mathcal{S} \subseteq V$ is the set of seed nodes in G.
4:     $\mathcal{C} \leftarrow V \setminus \mathcal{S}$ ▷ $\mathcal{C}$ is the set of connecting nodes in G.
5:     $G' \leftarrow G$ ▷ Make a copy of G.
6:     SORT($\mathcal{C}$)   ▷ Sort $\mathcal{C}$ wrt some node-ranking criteria.
7:     **for** *each* $w \in \mathcal{C}$ ***do***
8:         G'' ← *remove* w *and all its edges from* G'
9:         ▷ *Check whether the SP distances between seed nodes are preserved in* G''.
10:         ***if*** $\forall u, v \in \mathcal{S}$, $d_{G'}(u, v) = d_{G''}(u, v)$ ***then***
11:             $G' \leftarrow G''$
12:         ***end if***
13:     ***end for***
14:     ***return*** G'
15: ***end function***

As for the shortest path network, seed proteins not appearing in STRING are kept as isolated nodes in the minimal networks. Differently from the shortest path networks, the minimal connecting networks are quite compact. On the average, they contain only 1.5 times more proteins that their seed sets.

2.2.1 Ranking scores

In the current version, the ranking of a connecting node is obtained by applying three scores (sc,ss,cc), which are used as primary, secondary and tertiary sort key, respectively.

i) *Seed centrality (sc).* We say that a node connects two seed nodes if it appears in some shortest path connecting them. Thus, the seed centrality measure simply counts the number of distinct seed pairs connected by a node.

   Definition 2.2 (Seed centrality) Let $G = (V, E)$ be a shortest path network. Let $\mathcal{S} \subseteq V$ and $\mathcal{C} = V \setminus \mathcal{S}$ be the set of connecting and seed nodes in G, respectively. The seed centrality of a connecting node $w \in \mathcal{C}$ is defined by

$$sc(w) = |\{\{u, v\} \mid u, v \in \mathcal{S}, |sp_w(u, v)| > 0\}|$$

   where $sp_w(u, v)$ is the set of shortest paths between u and v in G passing through node w. □

   Note that, if $|\mathcal{S}| = n$ we have a total number of $n \cdot (n-1)/2$ distinct (unordered) pairs in $\mathcal{S}$. Then $\forall w \in \mathcal{C}$, $0 \leq cc(w) \leq n \cdot (n-1)/2$. The seed centrality property implicitly assumes that the higher $sc(w)$, the higher the probability that node w appears in a minimal connecting network.

ii) *Maximum semantic similarity with the reference GO term (ss).* The semantic similarity measures to which extent the annotation terms of each connecting node are related to the reference GO term: a connecting node with a high semantic similarity score is more likely to be functionally-related to the seed nodes. The semantic similarity is defined as the Lin's information-theoretic metric [4].

   The information-theoretic semantic similarity measures rely on the *information content of individual* terms t in the GO hierarchy:

$$ic(t) = -\log Pr(t),$$

   where $Pr(t)$ is the relative frequency of GO term t with respect to some background distribution. The background for the information content measure used here is given by the entire set of UniProt-GOA annotations for human proteins [2]. The Resnik's similarity [3] between two terms t_1 and t_2 is defined as the maximum information content among the common ancestors of t_1 and t_2:

$$sim_{Resnick}(t_1, t_2) = \max_{a \in \mathcal{A}(t_1) \cap \mathcal{A}(t_2)} \{ic(a)\},$$

   where $\mathcal{A}(t)$ denotes the set of all the ancestors of term t, recursively propagated up to the root of the GO hierarchy. The Lin's similarity [4] between two terms t_1 and t_2 is the normalized version of the Resnik's similarity:

$$sim_{Lin}(t_1, t_2) = \frac{2 \cdot sim_{Resnik}(t_1, t_2)}{ic(t_1) + ic(t_2)}$$

   We use Lin's similarity to evaluate how well a protein is related to a reference GO term. In detail, we define the maximum semantic similarity of a connecting node with respect to the reference GO term as the highest Lins score between the GO terms associated to the connecting node/protein and the reference GO term:

Definition 2.3 (Semantic similarity with the reference GO term) Let $G = (V, E)$ be a shortest path network built with respect to the reference GO term t. For each connecting node $w \in \mathcal{C}$, we define the *semantic similarity* with respect to reference GO term t by

$$ss(w, t) = \max\{sim_{Lin}(t', t) \mid t' \text{ is a GO term associated to } w\}.$$

□

The maximum semantic similarity property explicitly gives more importance to connecting proteins whose annotations are more closely related to the reference GO term.

iii) *Betweenness centrality (bc)*. The betweenness centrality is a measure of centrality of a node in a network [5]. Differently from the standard definition of betweenness centrality, here we compute this measure by considering uniquely the shortest paths connecting seed nodes.

Definition 2.4 (Betweenness centrality) Let $G = (V, E)$ be a shortest path network, let $\mathcal{S} \subseteq V$ be the set of seed nodes and $\mathcal{C} = V \setminus \mathcal{S}$ the set of connecting nodes. The *betweenness centrality* of a connecting node $w \in \mathcal{C}$ is defined by

$$bc(w) = \sum_{u,v \in \mathcal{S}} \frac{|sp_w(u, v)|}{|sp(u, v)|}$$

where

- $sp_w(u, v)$ is the set of shortest paths between u and v in G passing through node w.
- $sp(u, v)$ is the set of shortest paths between u and v in G. We have that, $sp(u, v) = \cup_{w \in V} \; sp_w(u, v)$. □

This property is mainly used to assess a local ranking for those connecting nodes that have exactly the same ranking with respect to the previous two properties. In large shortest path networks, this happens quite often, due to the limited range of values of the previous two above properties.

2.3 Quality filtering

A quality filtering procedure is applied to the minimal connecting networks built in the previous step (see Alg. 5). The idea is to filter out those networks for which the GO annotations of the connecting nodes are weakly related to the reference GO term. In particular, rare BP terms (i.e. BP terms with few related proteins) tend to produce minimal networks consisting uniquely of long paths. In most of such cases, the annotations of the connecting proteins are unrelated to the reference GO, and then the resulting minimal network is unlikely to include many proteins related to the reference GO. Such network-modules are discarded and not considered for the enrichment. The quality filtering procedure makes use of the maximum semantic similarity measure, as defined above (Definition 2.3). In particular, a minimal network is retained if, with respect to the reference GO term, the average maximum similarity of the connecting nodes is significantly higher than the average maximum similarity

of all the nodes in STRING, as assessed by a Students t-test with significance set to 5% (Line 12-23 in Alg. 5). The quality test filters out 1,205 networks out of 12,621, with sizes ranging from 3 to 137 nodes, with an average of 13. In this step, we filter out also minimal networks that do not contain any connecting node (Lines 9-11 in Alg. 5). Such networks are uninformative for a network-based enrichment analysis, since they do not contain more knowledge than their seed sets. The number of BP GO terms for which we extract a non trivial network is then 8,098.

Algorithm 5 Quality filtering

1: **function** QF($G, \mathcal{S}, t, \mu_t, \sigma_t, n$)
2:     ▷ $G = (V, E)$ is a minimal connecting network.
3:     ▷ $\mathcal{S} \subseteq V$ is the set of seed nodes in G.
4:     ▷ t is the reference GO term for the minimal connecting network G.
5:     ▷ μ_t is the mean semantic similarity in STRING wrt GO term t.
6:     ▷ σ_t is the semantic similarity variance in STRING wrt GO term t.
7:     ▷ n is the number of nodes in STRING.
8:     $\mathcal{C} \leftarrow V \setminus \mathcal{S}$ ▷ $\mathcal{C}$ is the set of connecting nodes in G.
9:     **if** $\mathcal{C} = \emptyset$ **then**
10:         **return** $TRUE$ ▷ The minimal connecting network has to be filtered-out
11:     **else**
12:         $tmp \leftarrow \emptyset$
13:         **for** *each* w *in* $\mathcal{C}$ **do**
14:   ADD($tmp, ss(w, t)$)   ▷ *Semantic similarity wrt t (Definition 2.3)*
15:   ***end for***
16: $\mu'_t \leftarrow$ MEAN(tmp)
17: $\sigma'_t \leftarrow$ VAR(tmp)
18: $n' \leftarrow |\mathcal{C}|$
19:   ***if*** TTEST($\mu'_t, \sigma'_t, n', \mu_t, \sigma_t, n$) < 0.05 ***then***
20:   ***return*** $FALSE$ ▷ *The minimal connecting network has to be retained*
21:   ***else***
22:   ***return*** $TRUE$ ▷ *The minimal connecting network has to be filtered-out*
23:   ***end if***
24:   ***end if***
25: ***end function***

References

[1] Hwang, F., Richards, D., Winter, P.: The Stainer Tree Problem. Elsevier, Amsterdam (1992)

[2] Gene Ontology Annotation database (UniProt-GOA). Generated on September 1, 2014. http://www.ebi.ac.uk/GOA

[3] Resnik,P. (1995) Using information content to evaluate semantic similarity in a taxonomy. In: Proceedings of the 14th International Joint Conference on Artificial Intelligence, Morgan Kaufmann, San Francisco, CA, pp. 448453.

[4] Lin, D.: An information-theoretic definition of similarity. In: Kaufmann, M. (ed.) Proceedings of the 15th International Conference on Machine Learning, pp. 296304 (1998)

[5] Freeman, L.: A set of measures of centrality based on betweenness. Sociometry 40, 3541 (1977)
