# Supplementary material for: NET-GE: a novel NETwork-based Gene Enrichment for detecting biological processes associated to Mendelian diseases
Source: BMC Genomics. 2015 Jun 18;16(Suppl 8):S6. doi: 10.1186/1471-2164-16-S8-S6 (PMC4480278; doi:10.1186/1471-2164-16-S8-S6)
Supplement: Additional file 2 — Figure S1: Number of enriched GO BP terms as a function of the maximum degree of the human proteins annotated with a the same term. [file 1471-2164-16-S8-S6-S2.pdf]

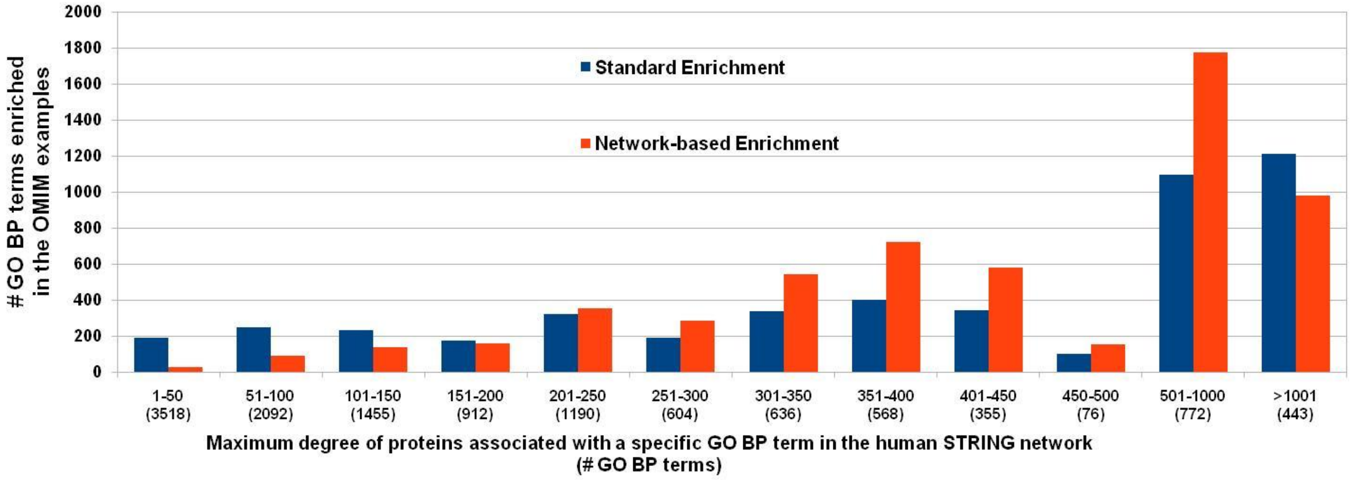

Figure 1S- Number of enriched GO BP terms as a function of the maximum degree of the human proteins annotated with a the same term.
