# Supplementary material for: NET-GE: a novel NETwork-based Gene Enrichment for detecting biological processes associated to Mendelian diseases
Source: BMC Genomics. 2015 Jun 18;16(Suppl 8):S6. doi: 10.1186/1471-2164-16-S8-S6 (PMC4480278; doi:10.1186/1471-2164-16-S8-S6)
Supplement: Additional file 3 — Detailed results for the OMIM-derived benchmark set. The archive contains pdf documents listing the enriched terms for each one of the 244 diseases in the OMIM-derived benchmark set. [file 1471-2164-16-S8-S6-S3.tgz › SUPPMAT/OMIM101400.pdf]

#101400 SAETHRE-CHOTZEN SYNDROME; SCS

OMIM Gene ID	HGNC	UniProtAC
176943	FGFR2	P21802
601622	TWIST1	Q15672

Table 1: OMIM - UniProtAC mapping

Legend

- N1: #input proteins associated to the significant GO term
- N2: #proteins associated to the significant GO term
- P-value: Bonferroni-corrected p-value of Fisher's exact test
- *red*: go terms not related to the input proteins
- *blue*: go terms related to the input proteins (enriched uniquely by network-based method)
- *green*: go terms ancestors of terms enriched with the standard method (enriched uniquely by network-based method)

1 Standard enrichment

GO Term	N1	N2	P-value	Description
GO:0060363	2	15	8.81578e-05	cranial suture morphogenesis
GO:0097094	2	20	0.000159523	craniofacial suture morphogenesis
GO:0048701	2	56	0.00129298	embryonic cranial skeleton morphogenesis
GO:0048704	2	134	0.00748164	embryonic skeletal system morphogenesis
GO:0042476	2	169	0.0119189	odontogenesis
GO:0045667	2	169	0.0119189	regulation of osteoblast differentiation
GO:0048705	2	180	0.0135259	skeletal system morphogenesis
GO:0050679	2	188	0.0147584	positive regulation of epithelial cell proliferation
GO:0048562	2	199	0.0165409	embryonic organ morphogenesis
GO:0016202	2	203	0.0172142	regulation of striated muscle tissue development
GO:1901861	2	204	0.0173846	regulation of muscle tissue development
GO:0048634	2	207	0.017901	regulation of muscle organ development
GO:2000027	2	225	0.0211578	regulation of organ morphogenesis
GO:0030278	2	261	0.0284875	regulation of ossification
GO:0035602	1	1	0.031688	fibroblast growth factor receptor signaling pathway involved in negative regulation of apoptotic process in bon
GO:0035603	1	1	0.031688	fibroblast growth factor receptor signaling pathway involved in hemopoiesis
GO:0035604	1	1	0.031688	fibroblast growth factor receptor signaling pathway involved in positive regulation of cell proliferation in bone
GO:0060365	1	1	0.031688	coronal suture morphogenesis
GO:2000793	1	1	0.031688	cell proliferation involved in heart valve development
GO:2000800	1	1	0.031688	regulation of endocardial cushion to mesenchymal transition involved in heart valve formation
GO:2000802	1	1	0.031688	positive regulation of endocardial cushion to mesenchymal transition involved in heart valve formation
GO:0010639	2	327	0.0447513	negative regulation of organelle organization

Table 2: Overrepresented GO terms with the standard enrichment

2 Network-based enrichment

GO Term	N1	N2	P-value	Description
GO:0060411	2	146	0.0114256	cardiac septum morphogenesis
GO:0060485	2	161	0.0139029	mesenchyme development
GO:0072089	2	169	0.0153234	stem cell proliferation
GO:0060415	2	181	0.0175838	muscle tissue morphogenesis

Table 3: Overrepresented terms with the network-based enrichment. Only terms not detected with the standard method.
