# Supplementary material for: NET-GE: a novel NETwork-based Gene Enrichment for detecting biological processes associated to Mendelian diseases
Source: BMC Genomics. 2015 Jun 18;16(Suppl 8):S6. doi: 10.1186/1471-2164-16-S8-S6 (PMC4480278; doi:10.1186/1471-2164-16-S8-S6)
Supplement: Additional file 3 — Detailed results for the OMIM-derived benchmark set. The archive contains pdf documents listing the enriched terms for each one of the 244 diseases in the OMIM-derived benchmark set. [file 1471-2164-16-S8-S6-S3.tgz › SUPPMAT/OMIM101600-OMIM123150.pdf]

#101600 PFEIFFER SYNDROME
#123150 JACKSON-WEISS SYNDROME; JWS

OMIM Gene ID	HGNC	UniProtAC
136350	FGFR1	P11362
176943	FGFR2	P21802

Table 1: OMIM - UniProtAC mapping

Legend

- N1: #input proteins associated to the significant GO term
- N2: #proteins associated to the significant GO term
- P-value: Bonferroni-corrected p-value of Fisher's exact test
- *red*: go terms not related to the input proteins
- *blue*: go terms related to the input proteins (enriched uniquely by network-based method)
- *green*: go terms ancestors of terms enriched with the standard method (enriched uniquely by network-based method)

1 Standard enrichment

GO Term	N1	N2	P-value	Description
GO:0035607	2	4	4.08565e-06	fibroblast growth factor receptor signaling pathway involved in orbitofrontal cortex development
GO:0021847	2	5	6.80945e-06	ventricular zone neuroblast division
GO:0021869	2	8	1.90664e-05	forebrain ventricular zone progenitor cell division
GO:0021873	2	15	7.14992e-05	forebrain neuroblast division
GO:0036445	2	15	7.14992e-05	neuronal stem cell division
GO:0055057	2	15	7.14992e-05	neuroblast division
GO:0060484	2	20	0.000129379	lung-associated mesenchyme development
GO:0048103	2	24	0.00018794	somatic stem cell division
GO:0060445	2	28	0.000257396	branching involved in salivary gland morphogenesis
GO:0048762	2	36	0.000428994	mesenchymal cell differentiation
GO:0060045	2	36	0.000428994	positive regulation of cardiac muscle cell proliferation
GO:0010453	2	39	0.000504579	regulation of cell fate commitment
GO:0017145	2	44	0.000644172	stem cell division
GO:0002053	2	53	0.000938339	positive regulation of mesenchymal cell proliferation
GO:0030901	2	54	0.000974428	midbrain development
GO:0060043	2	56	0.00104865	regulation of cardiac muscle cell proliferation
GO:0010464	2	60	0.00120527	regulation of mesenchymal cell proliferation
GO:0055021	2	62	0.00128766	regulation of cardiac muscle tissue growth
GO:0001657	2	66	0.00146062	ureteric bud development
GO:0060420	2	70	0.00164447	regulation of heart growth
GO:0072164	2	70	0.00164447	mesonephric tubule development
GO:0072163	2	71	0.00169214	mesonephric epithelium development
GO:0042472	2	72	0.00174049	inner ear morphogenesis
GO:0055024	2	77	0.00199244	regulation of cardiac muscle tissue development
GO:0060485	2	77	0.00199244	mesenchyme development
GO:0060688	2	83	0.00231725	regulation of morphogenesis of a branching structure
GO:2000648	2	87	0.0025474	positive regulation of stem cell proliferation
GO:0048863	2	91	0.00278846	stem cell differentiation
GO:0010518	2	93	0.00291307	positive regulation of phospholipase activity
GO:0072073	2	97	0.00317047	kidney epithelium development
GO:0010517	2	104	0.00364713	regulation of phospholipase activity
GO:0060193	2	108	0.00393448	positive regulation of lipase activity
GO:0046620	2	123	0.00510909	regulation of organ growth
GO:0072091	2	137	0.00634365	regulation of stem cell proliferation
GO:0002064	2	153	0.00791801	epithelial cell development
GO:0060191	2	155	0.00812705	regulation of lipase activity
GO:0045787	2	165	0.00921316	positive regulation of cell cycle
GO:0051301	2	177	0.0106064	cell division
GO:0048705	2	180	0.01097	skeletal system morphogenesis
GO:0048015	2	182	0.0112158	phosphatidylinositol-mediated signaling
GO:0048017	2	182	0.0112158	inositol lipid-mediated signaling
GO:0048666	2	186	0.0117156	neuron development
GO:0008286	2	195	0.01288	insulin receptor signaling pathway
GO:0007173	2	202	0.0138238	epidermal growth factor receptor signaling pathway
GO:0016202	2	203	0.0139614	regulation of striated muscle tissue development
GO:1901861	2	204	0.0140996	regulation of muscle tissue development
GO:0038127	2	205	0.0142385	ERBB signaling pathway
GO:0048634	2	207	0.0145184	regulation of muscle organ development
GO:0048638	2	207	0.0145184	regulation of developmental growth
GO:0008543	2	211	0.0150863	fibroblast growth factor receptor signaling pathway
GO:2000027	2	225	0.0171597	regulation of organ morphogenesis
GO:0044344	2	237	0.0190432	cellular response to fibroblast growth factor stimulus
GO:0071774	2	243	0.0200218	response to fibroblast growth factor
GO:0035295	2	247	0.0206877	tube development
GO:0046777	2	268	0.0243628	protein autophosphorylation
GO:0032869	2	270	0.0247284	cellular response to insulin stimulus
GO:0035602	1	1	0.0257001	fibroblast growth factor receptor signaling pathway involved in negative regulation of apoptotic process in bon
GO:0035603	1	1	0.0257001	fibroblast growth factor receptor signaling pathway involved in hemopoiesis
GO:0035604	1	1	0.0257001	fibroblast growth factor receptor signaling pathway involved in positive regulation of cell proliferation in bone
GO:0060365	1	1	0.0257001	coronal suture morphogenesis

Table 2: Overrepresented GO terms with the standard enrichment

GO Term	N1	N2	P-value	Description
GO:0048011	2	276	0.0258418	neurotrophin TRK receptor signaling pathway
GO:0038179	2	285	0.0275577	neurotrophin signaling pathway
GO:0061138	2	286	0.0277518	morphogenesis of a branching epithelium
GO:0038095	2	294	0.0293289	Fc-epsilon receptor signaling pathway
GO:0001763	2	303	0.0311551	morphogenesis of a branching structure
GO:0018108	2	322	0.0351918	peptidyl-tyrosine phosphorylation
GO:0018212	2	332	0.0374151	peptidyl-tyrosine modification
GO:0038093	2	350	0.0415886	Fc receptor signaling pathway
GO:0001525	2	358	0.0435142	angiogenesis
GO:0060429	2	368	0.0459827	epithelium development
GO:0071375	2	372	0.0469891	cellular response to peptide hormone stimulus
GO:0032868	2	376	0.0480065	response to insulin

Table 3: Overrepresented GO terms with the standard enrichment

2 Network-based enrichment

GO Term	N1	N2	P-value	Description
GO:0090080	2	10	5.18175e-05	positive regulation of MAPKKK cascade by fibroblast growth factor receptor signaling pathway
GO:0030916	2	21	0.000241815	otic vesicle formation
GO:0007413	2	57	0.0018378	axonal fasciculation
GO:0030947	2	71	0.00286148	regulation of vascular endothelial growth factor receptor signaling pathway
GO:0040036	2	82	0.00382413	regulation of fibroblast growth factor receptor signaling pathway
GO:0008038	2	104	0.00616744	neuron recognition
GO:2001222	2	109	0.00677775	regulation of neuron migration
GO:0072089	2	169	0.0163467	stem cell proliferation
GO:0009880	2	185	0.0195985	embryonic pattern specification
GO:0032941	2	214	0.0262439	secretion by tissue
GO:0051781	2	266	0.0405846	positive regulation of cell division
GO:0030324	2	285	0.0466012	lung development
GO:0007589	2	291	0.0485876	body fluid secretion

Table 4: Overrepresented terms with the network-based enrichment. Only terms not detected with the standard method.
