# Supplementary material for: NET-GE: a novel NETwork-based Gene Enrichment for detecting biological processes associated to Mendelian diseases
Source: BMC Genomics. 2015 Jun 18;16(Suppl 8):S6. doi: 10.1186/1471-2164-16-S8-S6 (PMC4480278; doi:10.1186/1471-2164-16-S8-S6)
Supplement: Additional file 3 — Detailed results for the OMIM-derived benchmark set. The archive contains pdf documents listing the enriched terms for each one of the 244 diseases in the OMIM-derived benchmark set. [file 1471-2164-16-S8-S6-S3.tgz › SUPPMAT/OMIM102530.pdf]

#102530 SPERMATOGENIC FAILURE 6; SPGF6

**OMIM Gene ID**	**HGNC**	**UniProtAC**
606845	GOPC	Q9HD26
609856	SPATA16	Q9BXB7

Table 1: OMIM - UniProtAC mapping

Legend

- N1: #input proteins associated to the significant GO term
- N2: #proteins associated to the significant GO term
- P-value: Bonferroni-corrected p-value of Fisher's exact test
- *red*: go terms not related to the input proteins
- *blue*: go terms related to the input proteins (enriched uniquely by network-based method)
- *green*: go terms ancestors of terms enriched with the standard method (enriched uniquely by network-based method)

1 Standard enrichment

GO Term	N1	N2	P-value	Description
GO:0042999	1	1	0.00466312	regulation of Golgi to plasma membrane CFTR protein transport
GO:0043002	1	1	0.00466312	negative regulation of Golgi to plasma membrane CFTR protein transport
GO:0043004	1	1	0.00466312	cytoplasmic sequestering of CFTR protein
GO:0042997	1	7	0.0326392	negative regulation of Golgi to plasma membrane protein transport
GO:0042996	1	10	0.0466256	regulation of Golgi to plasma membrane protein transport

Table 2: Overrepresented GO terms with the standard enrichment

2 Network-based enrichment

No novel enriched terms
