# Supplementary material for: NET-GE: a novel NETwork-based Gene Enrichment for detecting biological processes associated to Mendelian diseases
Source: BMC Genomics. 2015 Jun 18;16(Suppl 8):S6. doi: 10.1186/1471-2164-16-S8-S6 (PMC4480278; doi:10.1186/1471-2164-16-S8-S6)
Supplement: Additional file 3 — Detailed results for the OMIM-derived benchmark set. The archive contains pdf documents listing the enriched terms for each one of the 244 diseases in the OMIM-derived benchmark set. [file 1471-2164-16-S8-S6-S3.tgz › SUPPMAT/OMIM103470.pdf]

#103470 ALBINISM, OCULAR, WITH SENSORINEURAL DEAFNESS

OMIM Gene ID	HGNC	UniProtAC
156845	MITF	O75030
606933	TYR	P14679

Table 1: OMIM - UniProtAC mapping

Legend

- N1: #input proteins associated to the significant GO term
- N2: #proteins associated to the significant GO term
- P-value: Bonferroni-corrected p-value of Fisher's exact test
- *red*: go terms not related to the input proteins
- *blue*: go terms related to the input proteins (enriched uniquely by network-based method)
- *green*: go terms ancestors of terms enriched with the standard method (enriched uniquely by network-based method)

1 Standard enrichment

GO Term	N1	N2	P-value	Description
GO:0006583	1	2	0.0172746	melanin biosynthetic process from tyrosine
GO:0006726	1	4	0.034548	eye pigment biosynthetic process
GO:0042441	1	4	0.034548	eye pigment metabolic process
GO:0043324	1	4	0.034548	pigment metabolic process involved in developmental pigmentation
GO:0043474	1	4	0.034548	pigment metabolic process involved in pigmentation

Table 2: Overrepresented GO terms with the standard enrichment

2 Network-based enrichment

GO Term	N1	N2	P-value	Description
GO:0042438	2	28	0.000236366	melanin biosynthetic process
GO:0044550	2	37	0.000416456	secondary metabolite biosynthetic process
GO:0046189	2	108	0.00361303	phenol-containing compound biosynthetic process
GO:0046148	2	116	0.0041708	pigment biosynthetic process
GO:0019748	2	123	0.00469168	secondary metabolic process
GO:0042440	2	152	0.00717603	pigment metabolic process
GO:0018958	2	319	0.0317162	phenol-containing compound metabolic process

Table 3: Overrepresented terms with the network-based enrichment. Only terms not detected with the standard method.
