# Supplementary material for: NET-GE: a novel NETwork-based Gene Enrichment for detecting biological processes associated to Mendelian diseases
Source: BMC Genomics. 2015 Jun 18;16(Suppl 8):S6. doi: 10.1186/1471-2164-16-S8-S6 (PMC4480278; doi:10.1186/1471-2164-16-S8-S6)
Supplement: Additional file 3 — Detailed results for the OMIM-derived benchmark set. The archive contains pdf documents listing the enriched terms for each one of the 244 diseases in the OMIM-derived benchmark set. [file 1471-2164-16-S8-S6-S3.tgz › SUPPMAT/OMIM104300.pdf]

#104300 ALZHEIMER DISEASE; AD

**OMIM Gene ID**	**HGNC**	**UniProtAC**
103950	A2M	P01023
104760	APP	P05067
106180	ACE	P12821
163729	NOS3	P29474
191840	PLAU	P00749
602005	SORL1	Q92673
602403	BLMH	Q13867
602710	APBB2	Q92870
606989	MPO	P05164
608254	PAXIP1	Q6ZW49
613609	HFE	Q30201

Table 1: OMIM - UniProtAC mapping

Legend

- N1: #input proteins associated to the significant GO term
- N2: #proteins associated to the significant GO term
- P-value: Bonferroni-corrected p-value of Fisher's exact test
- *red*: go terms not related to the input proteins
- *blue*: go terms related to the input proteins (enriched uniquely by network-based method)
- *green*: go terms ancestors of terms enriched with the standard method (enriched uniquely by network-based method)

1 Standard enrichment

GO Term	N1	N2	P-value	Description
GO:0014910	3	46	0.000235428	regulation of smooth muscle cell migration
GO:0016477	5	1046	0.00552042	cell migration
GO:0006928	6	1973	0.00631821	cellular component movement
GO:0007596	4	501	0.00797285	blood coagulation
GO:0050817	4	501	0.00797285	coagulation
GO:0048870	5	1136	0.0082461	cell motility
GO:0007599	4	510	0.00855166	hemostasis
GO:0051346	4	521	0.00930069	negative regulation of hydrolase activity
GO:0040011	5	1284	0.0149249	locomotion
GO:0065008	7	3888	0.0235299	regulation of biological quality
GO:0001990	2	29	0.0264721	regulation of systemic arterial blood pressure by hormone
GO:0003044	2	29	0.0264721	regulation of systemic arterial blood pressure mediated by a chemical signal
GO:0050886	2	29	0.0264721	endocrine process
GO:0009628	5	1467	0.0283693	response to abiotic stimulus
GO:0050878	4	717	0.0324981	regulation of body fluid levels

Table 2: Overrepresented GO terms with the standard enrichment

2 Network-based enrichment

GO Term	N1	N2	P-value	Description
GO:0050819	5	119	5.62363e-07	negative regulation of coagulation
GO:0006869	6	627	4.28883e-05	lipid transport
GO:0050818	5	299	5.77055e-05	regulation of coagulation
GO:0030195	4	106	7.27875e-05	negative regulation of blood coagulation
GO:1900047	4	106	7.27875e-05	negative regulation of hemostasis
GO:0051604	6	741	0.000115551	protein maturation
GO:0002684	8	2287	0.000154512	positive regulation of immune system process
GO:0043065	7	1406	0.000154822	positive regulation of apoptotic process
GO:0043068	7	1416	0.000162537	positive regulation of programmed cell death
GO:0010035	7	1420	0.000165713	response to inorganic substance
GO:0051247	9	3557	0.00018623	positive regulation of protein metabolic process
GO:0050776	8	2377	0.000208899	regulation of immune response
GO:0051241	7	1513	0.000255977	negative regulation of multicellular organismal process
GO:0010942	7	1542	0.000291514	positive regulation of cell death
GO:0002003	3	32	0.000306254	angiotensin maturation
GO:0033993	8	2604	0.000425441	response to lipid
GO:0060548	8	2642	0.00047624	negative regulation of cell death
GO:0000303	3	44	0.00081598	response to superoxide
GO:0000302	5	514	0.000850477	response to reactive oxygen species
GO:0000305	3	45	0.000874106	response to oxygen radical
GO:0030198	6	1111	0.0012588	extracellular matrix organization
GO:0090287	5	557	0.00126457	regulation of cellular response to growth factor stimulus
GO:0043062	6	1116	0.0012924	extracellular structure organization
GO:0006979	6	1117	0.00129921	response to oxidative stress
GO:2000027	5	574	0.00146669	regulation of organ morphogenesis
GO:0006955	8	3063	0.00150095	immune response
GO:0043207	7	2065	0.00213315	response to external biotic stimulus
GO:0006897	6	1228	0.00226309	endocytosis
GO:0010631	4	251	0.00230853	epithelial cell migration
GO:0007507	5	638	0.00246842	heart development
GO:0032270	8	3279	0.00254215	positive regulation of cellular protein metabolic process
GO:1903034	6	1274	0.00280589	regulation of response to wounding
GO:0032101	7	2173	0.00301312	regulation of response to external stimulus
GO:0030193	4	273	0.00322494	regulation of blood coagulation
GO:1900046	4	273	0.00322494	regulation of hemostasis
GO:0009607	7	2197	0.00324562	response to biotic stimulus
GO:0007565	4	290	0.00409996	female pregnancy
GO:0040008	7	2308	0.00452868	regulation of growth
GO:0002694	6	1443	0.00580096	regulation of leukocyte activation
GO:0008202	5	760	0.00582648	steroid metabolic process
GO:0048585	8	3759	0.00727711	negative regulation of response to stimulus
GO:0043066	7	2487	0.00749289	negative regulation of apoptotic process
GO:0010951	5	804	0.00767388	negative regulation of endopeptidase activity
GO:1901214	5	808	0.00786238	regulation of neuron death
GO:0010743	3	93	0.00792473	regulation of macrophage derived foam cell differentiation
GO:0043069	7	2511	0.00799304	negative regulation of programmed cell death
GO:0006801	3	94	0.00818451	superoxide metabolic process
GO:0070374	4	349	0.00854452	positive regulation of ERK1 and ERK2 cascade
GO:0003073	3	97	0.00899757	regulation of systemic arterial blood pressure
GO:0010466	5	836	0.00928646	negative regulation of peptidase activity
GO:0050865	6	1568	0.00939937	regulation of cell activation
GO:2001237	4	358	0.0094503	negative regulation of extrinsic apoptotic signaling pathway
GO:0006935	5	841	0.00956089	chemotaxis
GO:0042330	5	841	0.00956089	taxis
GO:0032496	5	847	0.0098986	response to lipopolysaccharide
GO:0007568	5	848	0.00995579	aging
GO:0016486	3	101	0.0101626	peptide hormone processing
GO:0050727	5	853	0.0102457	regulation of inflammatory response
GO:0033628	3	102	0.0104686	regulation of cell adhesion mediated by integrin
GO:0031347	6	1612	0.011035	regulation of defense response

Table 3: Overrepresented terms with the network-based enrichment. Only terms not detected with the standard method.

GO Term	N1	N2	P-value	Description
GO:0051223	6	1645	0.0124092	regulation of protein transport
GO:0045926	5	891	0.0126729	negative regulation of growth
GO:0001974	3	109	0.0127836	blood vessel remodeling
GO:0035023	3	114	0.0146295	regulation of Rho protein signal transduction
GO:0002237	5	918	0.0146572	response to molecule of bacterial origin
GO:0046686	3	115	0.0150186	response to cadmium ion
GO:0061041	4	405	0.0153861	regulation of wound healing
GO:0051054	4	411	0.0163053	positive regulation of DNA metabolic process
GO:0008284	7	2808	0.0169214	positive regulation of cell proliferation
GO:0001525	5	949	0.0172291	angiogenesis
GO:0010038	5	951	0.0174065	response to metal ion
GO:0000187	4	421	0.0179269	activation of MAPK activity
GO:0042594	4	427	0.0189555	response to starvation
GO:0001817	6	1774	0.0191977	regulation of cytokine production
GO:0098542	5	972	0.0193571	defense response to other organism
GO:0006066	5	986	0.0207503	alcohol metabolic process
GO:0001666	5	987	0.0208528	response to hypoxia
GO:0008217	4	438	0.0209539	regulation of blood pressure
GO:0044087	6	1812	0.0216939	regulation of cellular component biogenesis
GO:0036293	5	999	0.0221136	response to decreased oxygen levels
GO:0051349	3	133	0.02324	positive regulation of lyase activity
GO:0031401	7	2947	0.0233689	positive regulation of protein modification process
GO:0043408	6	1837	0.0234765	regulation of MAPK cascade
GO:0070201	6	1849	0.0243738	regulation of establishment of protein localization
GO:0051222	5	1034	0.0261366	positive regulation of protein transport
GO:0002697	5	1046	0.027641	regulation of immune effector process
GO:0097190	5	1052	0.0284184	apoptotic signaling pathway
GO:0019229	3	143	0.0288802	regulation of vasoconstriction
GO:0046903	6	1910	0.0293797	secretion
GO:0016192	7	3055	0.0297059	vesicle-mediated transport
GO:0030334	6	1926	0.0308231	regulation of cell migration
GO:0080134	7	3072	0.0308238	regulation of response to stress
GO:0016049	4	484	0.0310408	cell growth
GO:0051347	6	1933	0.0314726	positive regulation of transferase activity
GO:0044706	4	486	0.031548	multi-multicellular organism process
GO:0070482	5	1076	0.031701	response to oxygen levels
GO:0030252	2	18	0.0350493	growth hormone secretion
GO:0046889	3	157	0.0381958	positive regulation of lipid biosynthetic process
GO:0032411	3	158	0.038928	positive regulation of transporter activity
GO:0034097	6	2017	0.0401788	response to cytokine
GO:0009636	4	517	0.0402185	response to toxic substance
GO:0032642	3	160	0.0404201	regulation of chemokine production
GO:0009968	7	3204	0.0407734	negative regulation of signal transduction
GO:2000145	6	2039	0.0427571	regulation of cell motility
GO:0048146	3	164	0.0435167	positive regulation of fibroblast proliferation
GO:0001775	6	2070	0.0466182	cell activation
GO:0010459	2	21	0.0480809	negative regulation of heart rate
GO:0051046	6	2094	0.049799	regulation of secretion

Table 4: Overrepresented terms with the network-based enrichment. Only terms not detected with the standard method.
