# Supplementary material for: NET-GE: a novel NETwork-based Gene Enrichment for detecting biological processes associated to Mendelian diseases
Source: BMC Genomics. 2015 Jun 18;16(Suppl 8):S6. doi: 10.1186/1471-2164-16-S8-S6 (PMC4480278; doi:10.1186/1471-2164-16-S8-S6)
Supplement: Additional file 3 — Detailed results for the OMIM-derived benchmark set. The archive contains pdf documents listing the enriched terms for each one of the 244 diseases in the OMIM-derived benchmark set. [file 1471-2164-16-S8-S6-S3.tgz › SUPPMAT/OMIM105200.pdf]

#105200 AMYLOIDOSIS, FAMILIAL VISCERAL

OMIM Gene ID	HGNC	UniProtAC
107680	APOA1	P02647
134820	FGA	P02671
153450	LYZ	P61626

Table 1: OMIM - UniProtAC mapping

Legend

- N1: #input proteins associated to the significant GO term
- N2: #proteins associated to the significant GO term
- P-value: Bonferroni-corrected p-value of Fisher's exact test
- *red*: go terms not related to the input proteins
- *blue*: go terms related to the input proteins (enriched uniquely by network-based method)
- *green*: go terms ancestors of terms enriched with the standard method (enriched uniquely by network-based method)

1 Standard enrichment

GO Term	N1	N2	P-value	Description
GO:0034114	2	17	0.000254271	regulation of heterotypic cell-cell adhesion
GO:1900026	2	31	0.000869166	positive regulation of substrate adhesion-dependent cell spreading
GO:1900024	2	37	0.00124474	regulation of substrate adhesion-dependent cell spreading
GO:0010770	2	73	0.00490855	positive regulation of cell morphogenesis involved in differentiation
GO:0002576	2	82	0.00620193	platelet degranulation
GO:0022407	2	120	0.0133249	regulation of cell-cell adhesion
GO:0010811	2	150	0.0208441	positive regulation of cell-substrate adhesion
GO:0060354	1	1	0.0352913	negative regulation of cell adhesion molecule production
GO:0072377	1	1	0.0352913	blood coagulation, common pathway
GO:2000260	1	1	0.0352913	regulation of blood coagulation, common pathway
GO:2000261	1	1	0.0352913	negative regulation of blood coagulation, common pathway
GO:0030168	2	216	0.0432604	platelet activation

Table 2: Overrepresented GO terms with the standard enrichment

2 Network-based enrichment

GO Term	N1	N2	P-value	Description
GO:0007159	3	109	3.09002e-05	leukocyte cell-cell adhesion
GO:0032368	3	263	0.000441188	regulation of lipid transport
GO:0007229	3	274	0.000499125	integrin-mediated signaling pathway
GO:0070374	3	349	0.00103386	positive regulation of ERK1 and ERK2 cascade
GO:0032372	2	22	0.00113222	negative regulation of sterol transport
GO:0032375	2	22	0.00113222	negative regulation of cholesterol transport
GO:0046890	3	401	0.00157002	regulation of lipid biosynthetic process
GO:0002523	2	29	0.00198968	leukocyte migration involved in inflammatory response
GO:0050920	3	450	0.00222056	regulation of chemotaxis
GO:0010803	2	32	0.0024306	regulation of tumor necrosis factor-mediated signaling pathway
GO:0070372	3	536	0.00375653	regulation of ERK1 and ERK2 cascade
GO:0001818	3	578	0.00471252	negative regulation of cytokine production
GO:0050706	2	50	0.00600082	regulation of interleukin-1 beta secretion
GO:0050795	3	630	0.00610489	regulation of behavior
GO:0045785	3	632	0.00616331	positive regulation of cell adhesion
GO:0032369	2	51	0.00624563	negative regulation of lipid transport
GO:0050900	3	635	0.00625163	leukocyte migration
GO:0050704	2	56	0.00754298	regulation of interleukin-1 secretion
GO:0050708	3	710	0.00874309	regulation of protein secretion
GO:0019835	2	62	0.0092611	cytolysis
GO:0042304	2	62	0.0092611	regulation of fatty acid biosynthetic process
GO:0090022	2	62	0.0092611	regulation of neutrophil chemotaxis
GO:1902622	2	63	0.0095645	regulation of neutrophil migration
GO:0032102	3	737	0.00978045	negative regulation of response to external stimulus
GO:0006935	3	841	0.01454	chemotaxis
GO:0042330	3	841	0.01454	taxis
GO:0019216	3	845	0.0147487	regulation of lipid metabolic process
GO:0032371	2	79	0.0150839	regulation of sterol transport
GO:0032374	2	79	0.0150839	regulation of cholesterol transport
GO:0043030	2	79	0.0150839	regulation of macrophage activation
GO:0071622	2	82	0.0162579	regulation of granulocyte chemotaxis
GO:0016337	3	900	0.017824	single organismal cell-cell adhesion
GO:0032956	3	928	0.0195419	regulation of actin cytoskeleton organization
GO:0001525	3	949	0.0209003	angiogenesis
GO:0098542	3	972	0.0224587	defense response to other organism
GO:0098602	3	981	0.023089	single organism cell adhesion
GO:0032970	3	996	0.0241655	regulation of actin filament-based process
GO:0051222	3	1034	0.0270413	positive regulation of protein transport
GO:0051047	3	1063	0.0293833	positive regulation of secretion
GO:0051055	2	111	0.0298696	negative regulation of lipid biosynthetic process
GO:0030198	3	1111	0.0335502	extracellular matrix organization
GO:0043062	3	1116	0.0340057	extracellular structure organization
GO:0032651	2	119	0.0343457	regulation of interleukin-1 beta production
GO:0045806	2	120	0.0349272	negative regulation of endocytosis
GO:0032844	3	1155	0.0377003	regulation of homeostatic process
GO:0032652	2	131	0.0416442	regulation of interleukin-1 production
GO:0030155	3	1217	0.044109	regulation of cell adhesion
GO:0060627	3	1229	0.0454278	regulation of vesicle-mediated transport
GO:0052548	3	1238	0.046434	regulation of endopeptidase activity
GO:0010638	3	1260	0.0489558	positive regulation of organelle organization

Table 3: Overrepresented terms with the network-based enrichment. Only terms not detected with the standard method.
