# Supplementary material for: NET-GE: a novel NETwork-based Gene Enrichment for detecting biological processes associated to Mendelian diseases
Source: BMC Genomics. 2015 Jun 18;16(Suppl 8):S6. doi: 10.1186/1471-2164-16-S8-S6 (PMC4480278; doi:10.1186/1471-2164-16-S8-S6)
Supplement: Additional file 3 — Detailed results for the OMIM-derived benchmark set. The archive contains pdf documents listing the enriched terms for each one of the 244 diseases in the OMIM-derived benchmark set. [file 1471-2164-16-S8-S6-S3.tgz › SUPPMAT/OMIM105400.pdf]

#105400 AMYOTROPHIC LATERAL SCLEROSIS 1; ALS1

**OMIM Gene ID**	**HGNC**	**UniProtAC**
147450	SOD1	P00441
162230	NEFH	P12036
170710	PRPH	P41219
601143	DCTN1	Q14203

Table 1: OMIM - UniProtAC mapping

Legend

- N1: #input proteins associated to the significant GO term
- N2: #proteins associated to the significant GO term
- P-value: Bonferroni-corrected p-value of Fisher's exact test
- *red*: go terms not related to the input proteins
- *blue*: go terms related to the input proteins (enriched uniquely by network-based method)
- *green*: go terms ancestors of terms enriched with the standard method (enriched uniquely by network-based method)

1 Standard enrichment

GO Term	N1	N2	P-value	Description
GO:0045104	3	47	2.85674e-06	intermediate filament cytoskeleton organization
GO:0045103	3	50	3.4529e-06	intermediate filament-based process
GO:0060052	2	16	0.0003991	neurofilament cytoskeleton organization
GO:1902589	4	2159	0.00421817	single-organism organelle organization
GO:0006996	4	2908	0.0138932	organelle organization
GO:0007017	3	992	0.0280403	microtubule-based process
GO:0007010	3	1074	0.0355335	cytoskeleton organization
GO:0008219	3	1106	0.0387832	cell death
GO:0016265	3	1117	0.039944	death
GO:0010970	2	164	0.044221	microtubule-based transport
GO:0030705	2	173	0.0492079	cytoskeleton-dependent intracellular transport

Table 2: Overrepresented GO terms with the standard enrichment

2 Network-based enrichment

GO Term	N1	N2	P-value	Description
GO:0008089	2	57	0.0120356	anterograde axon cargo transport
GO:0031175	3	614	0.01718	neuron projection development
GO:0030030	4	2507	0.0223653	cell projection organization
GO:0000226	3	828	0.0419788	microtubule cytoskeleton organization
GO:0030031	3	845	0.0446037	cell projection assembly
GO:0008088	2	111	0.0459389	axon cargo transport

Table 3: Overrepresented terms with the network-based enrichment. Only terms not detected with the standard method.
