# Supplementary material for: NET-GE: a novel NETwork-based Gene Enrichment for detecting biological processes associated to Mendelian diseases
Source: BMC Genomics. 2015 Jun 18;16(Suppl 8):S6. doi: 10.1186/1471-2164-16-S8-S6 (PMC4480278; doi:10.1186/1471-2164-16-S8-S6)
Supplement: Additional file 3 — Detailed results for the OMIM-derived benchmark set. The archive contains pdf documents listing the enriched terms for each one of the 244 diseases in the OMIM-derived benchmark set. [file 1471-2164-16-S8-S6-S3.tgz › SUPPMAT/OMIM105830.pdf]

#105830 ANGELMAN SYNDROME; AS

OMIM Gene ID	HGNC	UniProtAC
300005	MECP2	P51608
300203	CDKL5	O76039
601623	UBE3A	Q05086

Table 1: OMIM - UniProtAC mapping

Legend

- N1: #input proteins associated to the significant GO term
- N2: #proteins associated to the significant GO term
- P-value: Bonferroni-corrected p-value of Fisher's exact test
- *red*: go terms not related to the input proteins
- *blue*: go terms related to the input proteins (enriched uniquely by network-based method)
- *green*: go terms ancestors of terms enriched with the standard method (enriched uniquely by network-based method)

1 Standard enrichment

GO Term	N1	N2	P-value	Description
GO:0035037	1	1	0.0317145	sperm entry

Table 2: Overrepresented GO terms with the standard enrichment

2 Network-based enrichment

GO Term	N1	N2	P-value	Description
GO:0032320	3	1071	0.0181905	positive regulation of Ras GTPase activity
GO:0032318	3	1285	0.031433	regulation of Ras GTPase activity
GO:0001558	3	1390	0.0397922	regulation of cell growth

Table 3: Overrepresented terms with the network-based enrichment. Only terms not detected with the standard method.
