# Supplementary material for: NET-GE: a novel NETwork-based Gene Enrichment for detecting biological processes associated to Mendelian diseases
Source: BMC Genomics. 2015 Jun 18;16(Suppl 8):S6. doi: 10.1186/1471-2164-16-S8-S6 (PMC4480278; doi:10.1186/1471-2164-16-S8-S6)
Supplement: Additional file 3 — Detailed results for the OMIM-derived benchmark set. The archive contains pdf documents listing the enriched terms for each one of the 244 diseases in the OMIM-derived benchmark set. [file 1471-2164-16-S8-S6-S3.tgz › SUPPMAT/OMIM109400.pdf]

#109400 BASAL CELL NEVUS SYNDROME; BCNS

OMIM Gene ID	HGNC	UniProtAC
601309	PTCH1	Q13635
603673	PTCH2	Q9Y6C5
607035	SUFU	Q9UMX1

Table 1: OMIM - UniProtAC mapping

Legend

- N1: #input proteins associated to the significant GO term
- N2: #proteins associated to the significant GO term
- P-value: Bonferroni-corrected p-value of Fisher's exact test
- *red*: go terms not related to the input proteins
- *blue*: go terms related to the input proteins (enriched uniquely by network-based method)
- *green*: go terms ancestors of terms enriched with the standard method (enriched uniquely by network-based method)

1 Standard enrichment

GO Term	N1	N2	P-value	Description
GO:0045879	3	40	2.95508e-07	negative regulation of smoothened signaling pathway
GO:0008589	3	118	7.98337e-06	regulation of smoothened signaling pathway
GO:0045668	2	61	0.00206359	negative regulation of osteoblast differentiation
GO:0001843	2	120	0.00804297	neural tube closure
GO:0060562	2	120	0.00804297	epithelial tube morphogenesis
GO:0060606	2	122	0.00831416	tube closure
GO:0007224	2	138	0.0106451	smoothened signaling pathway
GO:0035239	2	141	0.0111141	tube morphogenesis
GO:0043433	2	147	0.0120824	negative regulation of sequence-specific DNA binding transcription factor activity
GO:0009968	3	1361	0.0125394	negative regulation of signal transduction
GO:0008544	2	151	0.0127502	epidermis development
GO:0023057	3	1420	0.0142432	negative regulation of signaling
GO:0010648	3	1424	0.014364	negative regulation of cell communication
GO:0045667	2	169	0.0159775	regulation of osteoblast differentiation
GO:0035148	2	178	0.017727	tube formation
GO:0048585	3	1639	0.021908	negative regulation of response to stimulus
GO:0030278	2	261	0.0381257	regulation of ossification

Table 2: Overrepresented GO terms with the standard enrichment

2 Network-based enrichment

No novel enriched terms
