# Supplementary material for: NET-GE: a novel NETwork-based Gene Enrichment for detecting biological processes associated to Mendelian diseases
Source: BMC Genomics. 2015 Jun 18;16(Suppl 8):S6. doi: 10.1186/1471-2164-16-S8-S6 (PMC4480278; doi:10.1186/1471-2164-16-S8-S6)
Supplement: Additional file 3 — Detailed results for the OMIM-derived benchmark set. The archive contains pdf documents listing the enriched terms for each one of the 244 diseases in the OMIM-derived benchmark set. [file 1471-2164-16-S8-S6-S3.tgz › SUPPMAT/OMIM109800.pdf]

#109800 BLADDER CANCER

OMIM Gene ID	HGNC	UniProtAC
134934	FGFR3	P22607
190020	HRAS	P01112
190070	KRAS	P01116
605303	TACC3	Q9Y6A5
614041	RB1	P06400

Table 1: OMIM - UniProtAC mapping

Legend

- N1: #input proteins associated to the significant GO term
- N2: #proteins associated to the significant GO term
- P-value: Bonferroni-corrected p-value of Fisher's exact test
- *red*: go terms not related to the input proteins
- *blue*: go terms related to the input proteins (enriched uniquely by network-based method)
- *green*: go terms ancestors of terms enriched with the standard method (enriched uniquely by network-based method)

1 Standard enrichment

GO Term	N1	N2	P-value	Description
GO:0035022	2	4	5.98885e-05	positive regulation of Rac protein signal transduction
GO:0051146	3	93	0.000102601	striated muscle cell differentiation
GO:0030154	5	2446	0.000809673	cell differentiation
GO:0008286	3	195	0.000958229	insulin receptor signaling pathway
GO:0042692	3	200	0.00103404	muscle cell differentiation
GO:0007265	3	201	0.00104966	Ras protein signal transduction
GO:0007173	3	202	0.00106545	epidermal growth factor receptor signaling pathway
GO:0038127	3	205	0.00111373	ERBB signaling pathway
GO:0008543	3	211	0.00121464	fibroblast growth factor receptor signaling pathway
GO:0035020	2	18	0.00152603	regulation of Rac protein signal transduction
GO:0044344	3	237	0.00172216	cellular response to fibroblast growth factor stimulus
GO:0071774	3	243	0.00185644	response to fibroblast growth factor
GO:0000165	3	249	0.0019975	MAPK cascade
GO:0007010	4	1074	0.00226562	cytoskeleton organization
GO:0043549	4	1079	0.00230791	regulation of kinase activity
GO:0032869	3	270	0.00254698	cellular response to insulin stimulus
GO:0048011	3	276	0.00272059	neurotrophin TRK receptor signaling pathway
GO:0038179	3	285	0.00299546	neurotrophin signaling pathway
GO:0038095	3	294	0.00328818	Fc-epsilon receptor signaling pathway
GO:0043523	3	300	0.00349351	regulation of neuron apoptotic process
GO:0051338	4	1198	0.00350009	regulation of transferase activity
GO:0023014	3	309	0.0038172	signal transduction by phosphorylation
GO:0051726	4	1232	0.00391231	regulation of cell cycle
GO:0045786	3	343	0.00521894	negative regulation of cell cycle
GO:1901214	3	348	0.00545011	regulation of neuron death
GO:0038093	3	350	0.00554445	Fc receptor signaling pathway
GO:0010948	3	358	0.00593264	negative regulation of cell cycle process
GO:0050678	3	361	0.00608273	regulation of epithelial cell proliferation
GO:0048869	5	3694	0.00636958	cellular developmental process
GO:0048169	2	37	0.006636	regulation of long-term neuronal synaptic plasticity
GO:0071375	3	372	0.0066546	cellular response to peptide hormone stimulus
GO:0032868	3	376	0.00687108	response to insulin
GO:0001932	4	1440	0.00727403	regulation of protein phosphorylation
GO:0060441	2	39	0.00738253	epithelial tube branching involved in lung morphogenesis
GO:1901653	3	391	0.00772438	cellular response to peptide
GO:0045879	2	40	0.00777066	negative regulation of smoothened signaling pathway
GO:0032228	2	48	0.0112328	regulation of synaptic transmission, GABAergic
GO:0035176	2	50	0.0121975	social behavior
GO:0051703	2	50	0.0121975	intraspecies interaction between organisms
GO:0002768	3	486	0.0147994	immune response-regulating cell surface receptor signaling pathway
GO:0002009	3	492	0.0153518	morphogenesis of an epithelium
GO:0002682	4	1758	0.0160583	regulation of immune system process
GO:0008542	2	58	0.0164521	visual learning
GO:0042325	4	1770	0.0164973	regulation of phosphorylation
GO:0007346	3	518	0.0179033	regulation of mitotic cell cycle
GO:0031399	4	1823	0.0185439	regulation of protein modification process
GO:0007632	2	63	0.0194328	visual behavior
GO:0043410	3	539	0.0201577	positive regulation of MAPK cascade
GO:0048168	2	66	0.0213399	regulation of neuronal synaptic plasticity
GO:0046579	2	67	0.0219954	positive regulation of Ras protein signal transduction
GO:0043434	3	567	0.0234453	response to peptide hormone
GO:0043550	2	70	0.0240209	regulation of lipid kinase activity
GO:0048729	3	578	0.024828	tissue morphogenesis
GO:0071417	3	588	0.026131	cellular response to organonitrogen compound
GO:0042127	4	2008	0.0271936	regulation of cell proliferation
GO:1901652	3	600	0.0277531	response to peptide
GO:0051057	2	76	0.0283387	positive regulation of small GTPase mediated signal transduction
GO:0002764	3	615	0.0298727	immune response-regulating signaling pathway
GO:0045595	4	2111	0.0331464	regulation of cell differentiation
GO:0000186	2	83	0.0338248	activation of MAPKK activity

Table 2: Overrepresented GO terms with the standard enrichment

GO Term	N1	N2	P-value	Description
GO:0009653	4	2131	0.0344061	anatomical structure morphogenesis
GO:1901699	3	645	0.0344273	cellular response to nitrogen compound
GO:0033674	3	656	0.0362058	positive regulation of kinase activity
GO:1902589	4	2159	0.0362292	single-organism organelle organization
GO:0010564	3	662	0.0372009	regulation of cell cycle process
GO:0008306	2	89	0.0389115	associative learning
GO:0032268	4	2272	0.0443252	regulation of cellular protein metabolic process
GO:0032870	3	716	0.046981	cellular response to hormone stimulus

Table 3: Overrepresented GO terms with the standard enrichment

2 Network-based enrichment

GO Term	N1	N2	P-value	Description
GO:0050680	4	392	0.000144955	negative regulation of epithelial cell proliferation
GO:0043524	4	459	0.000272651	negative regulation of neuron apoptotic process
GO:0008361	3	104	0.000455979	regulation of cell size
GO:1901215	4	524	0.000463131	negative regulation of neuron death
GO:0048640	3	122	0.000738665	negative regulation of developmental growth
GO:0048534	4	682	0.0013274	hematopoietic or lymphoid organ development
GO:0048609	5	2109	0.00156886	multicellular organismal reproductive process
GO:0035019	3	163	0.00176948	somatic stem cell maintenance
GO:0000122	5	2314	0.00249575	negative regulation of transcription from RNA polymerase II promoter
GO:0043066	5	2487	0.0035801	negative regulation of apoptotic process
GO:0010243	5	2509	0.00374141	response to organonitrogen compound
GO:0043069	5	2511	0.00375635	negative regulation of programmed cell death
GO:0045597	5	2514	0.00377885	positive regulation of cell differentiation
GO:0008283	5	2526	0.00386999	cell proliferation
GO:1901701	5	2540	0.00397851	cellular response to oxygen-containing compound
GO:0045639	3	226	0.00472736	positive regulation of myeloid cell differentiation
GO:0060548	5	2642	0.00484487	negative regulation of cell death
GO:0002683	4	945	0.00487371	negative regulation of immune system process
GO:1901698	5	2693	0.00533127	response to nitrogen compound
GO:0009725	5	2824	0.00676158	response to hormone
GO:0000079	3	255	0.00679212	regulation of cyclin-dependent protein serine/threonine kinase activity
GO:0097190	4	1052	0.00747023	apoptotic signaling pathway
GO:0032507	3	273	0.00833407	maintenance of protein location in cell
GO:0031398	3	276	0.00861171	positive regulation of protein ubiquitination
GO:0071495	5	3038	0.00974477	cellular response to endogenous stimulus
GO:0030855	4	1135	0.0101052	epithelial cell differentiation
GO:0080134	5	3072	0.0103028	regulation of response to stress
GO:0051651	3	307	0.011848	maintenance of location in cell
GO:1903322	3	310	0.0121983	positive regulation of protein modification by small protein conjugation or removal
GO:0051094	5	3200	0.0126373	positive regulation of developmental process
GO:0060560	3	320	0.0134153	developmental growth involved in morphogenesis
GO:0045892	5	3247	0.0135937	negative regulation of transcription, DNA-templated
GO:0007088	3	327	0.0143134	regulation of mitosis
GO:1902679	5	3295	0.0146292	negative regulation of RNA biosynthetic process
GO:0051253	5	3369	0.0163485	negative regulation of RNA metabolic process
GO:0045185	3	343	0.016514	maintenance of protein location
GO:0009628	5	3444	0.0182523	response to abiotic stimulus
GO:0010563	4	1353	0.0203134	negative regulation of phosphorus metabolic process
GO:0045936	4	1353	0.0203134	negative regulation of phosphate metabolic process
GO:0010629	5	3561	0.0215726	negative regulation of gene expression
GO:0051051	4	1375	0.0216568	negative regulation of transport
GO:0010608	4	1385	0.0222888	posttranscriptional regulation of gene expression
GO:2000113	5	3633	0.0238449	negative regulation of cellular macromolecule biosynthetic process
GO:0045934	5	3691	0.0258111	negative regulation of nucleobase-containing compound metabolic process
GO:0031669	3	400	0.0261559	cellular response to nutrient levels
GO:0045787	3	401	0.026352	positive regulation of cell cycle
GO:0002548	2	44	0.0263567	monocyte chemotaxis
GO:0007050	3	403	0.0267468	cell cycle arrest
GO:0010558	5	3771	0.0287339	negative regulation of macromolecule biosynthetic process
GO:0051641	4	1477	0.0287697	cellular localization
GO:0051172	5	3798	0.0297778	negative regulation of nitrogen compound metabolic process
GO:0071900	4	1492	0.0299463	regulation of protein serine/threonine kinase activity
GO:0040007	4	1496	0.0302661	growth
GO:0043433	3	432	0.0329195	negative regulation of sequence-specific DNA binding transcription factor activity
GO:0032940	4	1530	0.0330877	secretion by cell
GO:0051783	3	444	0.0357269	regulation of nuclear division
GO:0031396	3	451	0.0374356	regulation of protein ubiquitination
GO:0050768	3	457	0.0389424	negative regulation of neurogenesis
GO:0031347	4	1612	0.0406966	regulation of defense response
GO:0009266	3	469	0.0420755	response to temperature stimulus

Table 4: Overrepresented terms with the network-based enrichment. Only terms not detected with the standard method.

GO Term	N1	N2	P-value	Description
GO:0051224	3	470	0.0423438	negative regulation of protein transport
GO:0051223	4	1645	0.0440998	regulation of protein transport
GO:0002040	2	57	0.0444317	sprouting angiogenesis
GO:0031668	3	479	0.0448103	cellular response to extracellular stimulus

Table 5: Overrepresented terms with the network-based enrichment. Only terms not detected with the standard method.
