# Supplementary material for: NET-GE: a novel NETwork-based Gene Enrichment for detecting biological processes associated to Mendelian diseases
Source: BMC Genomics. 2015 Jun 18;16(Suppl 8):S6. doi: 10.1186/1471-2164-16-S8-S6 (PMC4480278; doi:10.1186/1471-2164-16-S8-S6)
Supplement: Additional file 3 — Detailed results for the OMIM-derived benchmark set. The archive contains pdf documents listing the enriched terms for each one of the 244 diseases in the OMIM-derived benchmark set. [file 1471-2164-16-S8-S6-S3.tgz › SUPPMAT/OMIM112600.pdf]

#112600 BRACHYDACTYLY, TYPE A2; BDA2

OMIM Gene ID	HGNC	UniProtAC
112261	BMP2	P12643
601146	GDF5	P43026
603248	BMPR1B	O00238

Table 1: OMIM - UniProtAC mapping

Legend

- N1: #input proteins associated to the significant GO term
- N2: #proteins associated to the significant GO term
- P-value: Bonferroni-corrected p-value of Fisher's exact test
- *red*: go terms not related to the input proteins
- *blue*: go terms related to the input proteins (enriched uniquely by network-based method)
- *green*: go terms ancestors of terms enriched with the standard method (enriched uniquely by network-based method)

1 Standard enrichment

GO Term	N1	N2	P-value	Description
GO:0002062	3	68	2.43859e-06	chondrocyte differentiation
GO:0007178	3	293	0.000201909	transmembrane receptor protein serine/threonine kinase signaling pathway
GO:0030501	2	49	0.00215786	positive regulation of bone mineralization
GO:0070169	2	51	0.00233943	positive regulation of biomineral tissue development
GO:0045778	2	62	0.00346903	positive regulation of ossification
GO:0030500	2	86	0.00670224	regulation of bone mineralization
GO:0030509	2	88	0.00701921	BMP signaling pathway
GO:0045597	3	985	0.00772675	positive regulation of cell differentiation
GO:0070167	2	93	0.00784364	regulation of biomineral tissue development
GO:0045669	2	95	0.00818625	positive regulation of osteoblast differentiation
GO:0007167	3	1091	0.0105025	enzyme linked receptor protein signaling pathway
GO:0061035	2	108	0.0105911	regulation of cartilage development
GO:0051094	3	1326	0.0188651	positive regulation of developmental process
GO:0035107	2	159	0.0230035	appendage morphogenesis
GO:0035108	2	159	0.0230035	limb morphogenesis
GO:0045666	2	168	0.025686	positive regulation of neuron differentiation
GO:0045667	2	169	0.0259932	regulation of osteoblast differentiation
GO:0051042	1	1	0.0346554	negative regulation of calcium-independent cell-cell adhesion
GO:0060804	1	1	0.0346554	positive regulation of Wnt signaling pathway by BMP signaling pathway
GO:0001501	2	213	0.0413086	skeletal system development

Table 2: Overrepresented GO terms with the standard enrichment

2 Network-based enrichment

GO Term	N1	N2	P-value	Description
GO:0051216	3	282	0.000697331	cartilage development
GO:0055009	2	18	0.000960823	atrial cardiac muscle tissue morphogenesis
GO:0060389	2	20	0.00119313	pathway-restricted SMAD protein phosphorylation
GO:2001054	2	23	0.00158865	negative regulation of mesenchymal cell apoptotic process
GO:0001503	3	452	0.00288306	ossification
GO:2001053	2	34	0.00352188	regulation of mesenchymal cell apoptotic process
GO:0060828	3	550	0.00520045	regulation of canonical Wnt signaling pathway
GO:0030278	3	644	0.00835521	regulation of ossification
GO:0001502	2	57	0.0100149	cartilage condensation
GO:0048762	2	62	0.0118648	mesenchymal cell differentiation
GO:0010718	2	65	0.0130498	positive regulation of epithelial to mesenchymal transition
GO:0030111	3	814	0.0168887	regulation of Wnt signaling pathway
GO:0061005	2	79	0.0193246	cell differentiation involved in kidney development
GO:0010720	3	977	0.0292197	positive regulation of cell development
GO:0001666	3	987	0.0301271	response to hypoxia
GO:0036293	3	999	0.0312404	response to decreased oxygen levels
GO:0007292	2	105	0.0342285	female gamete generation
GO:0070482	3	1076	0.0390437	response to oxygen levels
GO:0035136	2	114	0.040371	forelimb morphogenesis
GO:2000242	2	118	0.0432632	negative regulation of reproductive process
GO:0010769	3	1115	0.0434492	regulation of cell morphogenesis involved in differentiation

Table 3: Overrepresented terms with the network-based enrichment. Only terms not detected with the standard method.
