# Supplementary material for: NET-GE: a novel NETwork-based Gene Enrichment for detecting biological processes associated to Mendelian diseases
Source: BMC Genomics. 2015 Jun 18;16(Suppl 8):S6. doi: 10.1186/1471-2164-16-S8-S6 (PMC4480278; doi:10.1186/1471-2164-16-S8-S6)
Supplement: Additional file 3 — Detailed results for the OMIM-derived benchmark set. The archive contains pdf documents listing the enriched terms for each one of the 244 diseases in the OMIM-derived benchmark set. [file 1471-2164-16-S8-S6-S3.tgz › SUPPMAT/OMIM114480.pdf]

#114480 BREAST CANCER

OMIM Gene ID	HGNC	UniProtAC
133430	ESR1	P03372
160998	NQO2	P16083
164730	AKT1	P31749
171834	PIK3CA	P42336
176705	PHB	P35232
179617	RAD51	Q06609
190070	KRAS	P01116
191170	TP53	P04637
192090	CDH1	P12830
600185	BRCA2	P51587
600675	XRCC3	O43542
600936	HMMR	O75330
601387	TSG101	Q99816
601593	BARD1	Q99728
601763	CASP8	Q14790
602631	SLC22A18	Q96BI1
603615	RAD54L	Q92698
604373	CHEK2	O96017
605100	PPM1D	O15297
605882	BRIP1	Q9BX63
606837	RB1CC1	Q8TDY2
607585	ATM	Q13315
610355	PALB2	Q86YC2

Table 1: OMIM - UniProtAC mapping

Legend

- N1: #input proteins associated to the significant GO term
- N2: #proteins associated to the significant GO term
- P-value: Bonferroni-corrected p-value of Fisher's exact test
- *red*: go terms not related to the input proteins
- *blue*: go terms related to the input proteins (enriched uniquely by network-based method)
- *green*: go terms ancestors of terms enriched with the standard method (enriched uniquely by network-based method)

1 Standard enrichment

GO Term	N1	N2	P-value	Description
GO:0006302	9	175	9.00906e-13	double-strand break repair
GO:0006281	10	781	1.7672e-08	DNA repair
GO:0000724	6	89	2.05105e-08	double-strand break repair via homologous recombination
GO:0000725	6	90	2.1967e-08	recombinational repair
GO:0009314	9	591	5.1887e-08	response to radiation
GO:0033554	12	1930	3.57542e-07	cellular response to stress
GO:0042981	12	1970	4.52527e-07	regulation of apoptotic process
GO:0006259	11	1502	4.81853e-07	DNA metabolic process
GO:0043067	12	1982	4.85192e-07	regulation of programmed cell death
GO:0006974	10	1132	6.58118e-07	cellular response to DNA damage stimulus
GO:0010941	12	2079	8.38907e-07	regulation of cell death
GO:0006950	15	4134	1.14859e-06	response to stress
GO:0010212	6	188	1.92177e-06	response to ionizing radiation
GO:0048523	16	5279	2.79425e-06	negative regulation of cellular process
GO:0009628	10	1467	7.96575e-06	response to abiotic stimulus
GO:0097193	6	246	9.61432e-06	intrinsic apoptotic signaling pathway
GO:0048519	16	5756	1.0149e-05	negative regulation of biological process
GO:0010604	13	3285	1.15429e-05	positive regulation of macromolecule metabolic process
GO:0006310	6	294	2.77775e-05	DNA recombination
GO:0009893	13	3630	3.86389e-05	positive regulation of metabolic process
GO:0044710	18	8611	4.00915e-05	single-organism metabolic process
GO:0050896	20	11721	6.18464e-05	response to stimulus
GO:0010332	4	65	0.000100261	response to gamma radiation
GO:0022402	9	1409	0.000101233	cell cycle process
GO:0048522	15	5768	0.00011831	positive regulation of cellular process
GO:0051000	3	15	0.000129689	positive regulation of nitric-oxide synthase activity
GO:0042127	10	2008	0.000155499	regulation of cell proliferation
GO:0051716	18	9450	0.000186922	cellular response to stimulus
GO:0090399	3	17	0.000193668	replicative senescence
GO:0043069	8	1068	0.000194966	negative regulation of programmed cell death
GO:0031325	12	3418	0.000225519	positive regulation of cellular metabolic process
GO:0097190	6	439	0.000296032	apoptotic signaling pathway
GO:0060548	8	1147	0.000335967	negative regulation of cell death
GO:0043170	20	12986	0.000418755	macromolecule metabolic process
GO:0007569	4	93	0.000427525	cell aging
GO:0032770	3	22	0.000437728	positive regulation of monooxygenase activity
GO:0006996	11	2908	0.000456684	organelle organization
GO:0051726	8	1232	0.000578177	regulation of cell cycle
GO:0048518	15	6624	0.000773905	positive regulation of biological process
GO:1900739	3	27	0.000829748	regulation of protein insertion into mitochondrial membrane involved in apoptotic signaling pathway
GO:1900740	3	27	0.000829748	positive regulation of protein insertion into mitochondrial membrane involved in apoptotic signaling pathwa
GO:0090304	14	5696	0.000954085	nucleic acid metabolic process
GO:0042770	4	118	0.00111238	signal transduction in response to DNA damage
GO:0008285	7	920	0.00126229	negative regulation of cell proliferation
GO:0071214	5	302	0.00137465	cellular response to abiotic stimulus
GO:0008630	4	126	0.00144623	intrinsic apoptotic signaling pathway in response to DNA damage
GO:0044699	23	20881	0.001761	single-organism process
GO:0097285	4	135	0.0019051	cell-type specific apoptotic process
GO:0006139	15	7311	0.00289071	nucleobase-containing compound metabolic process
GO:0050999	3	41	0.00300718	regulation of nitric-oxide synthase activity
GO:1901028	3	41	0.00300718	regulation of mitochondrial outer membrane permeabilization involved in apoptotic signaling pathway
GO:0043066	7	1050	0.00304013	negative regulation of apoptotic process
GO:0010564	6	662	0.00322869	regulation of cell cycle process
GO:0051353	3	42	0.00323723	positive regulation of oxidoreductase activity
GO:0044763	21	16559	0.00377415	single-organism cellular process
GO:0044238	21	16587	0.00390104	primary metabolic process
GO:0051052	5	374	0.00391589	regulation of DNA metabolic process
GO:0006807	16	8714	0.00415779	nitrogen compound metabolic process
GO:0007568	5	394	0.00504755	aging
GO:0046483	15	7642	0.00518719	heterocycle metabolic process

Table 2: Overrepresented GO terms with the standard enrichment

GO Term	N1	N2	P-value	Description
GO:0006725	15	7662	0.0053686	cellular aromatic compound metabolic process
GO:0016043	14	6578	0.00570348	cellular component organization
GO:0060255	16	8942	0.00597726	regulation of macromolecule metabolic process
GO:0071840	14	6631	0.00629377	cellular component organization or biogenesis
GO:0008156	3	53	0.00657704	negative regulation of DNA replication
GO:0071704	21	17115	0.00719417	organic substance metabolic process
GO:0007050	4	189	0.00725391	cell cycle arrest
GO:0044767	14	6740	0.00768477	single-organism developmental process
GO:0034641	15	7912	0.00817888	cellular nitrogen compound metabolic process
GO:0032768	3	57	0.00820193	regulation of monooxygenase activity
GO:1901360	15	8010	0.00960528	organic cyclic compound metabolic process
GO:0071310	9	2482	0.0115111	cellular response to organic substance
GO:0071495	7	1291	0.0118316	cellular response to endogenous stimulus
GO:0010822	3	66	0.0127813	positive regulation of mitochondrion organization
GO:0035556	9	2537	0.0137568	intracellular signal transduction
GO:0000733	2	8	0.0143797	DNA strand renaturation
GO:0048513	8	1910	0.0151949	organ development
GO:0010628	8	1919	0.0157264	positive regulation of gene expression
GO:0071479	3	71	0.0159323	cellular response to ionizing radiation
GO:0010468	14	7158	0.0159771	regulation of gene expression
GO:0072401	3	72	0.0166184	signal transduction involved in DNA integrity checkpoint
GO:0072413	3	72	0.0166184	signal transduction involved in mitotic cell cycle checkpoint
GO:0072422	3	72	0.0166184	signal transduction involved in DNA damage checkpoint
GO:1902402	3	72	0.0166184	signal transduction involved in mitotic DNA damage checkpoint
GO:1902403	3	72	0.0166184	signal transduction involved in mitotic DNA integrity checkpoint
GO:0009968	7	1361	0.0166874	negative regulation of signal transduction
GO:0072395	3	73	0.0173238	signal transduction involved in cell cycle checkpoint
GO:0044260	17	11069	0.0183665	cellular macromolecule metabolic process
GO:0000075	4	241	0.0189102	cell cycle checkpoint
GO:0001756	3	76	0.0195577	somitogenesis
GO:0032502	14	7299	0.0202199	developmental process
GO:0035282	3	79	0.0219738	segmentation
GO:0023057	7	1420	0.0219755	negative regulation of signaling
GO:0009719	8	2012	0.0222087	response to endogenous stimulus
GO:0010648	7	1424	0.0223794	negative regulation of cell communication
GO:0032386	5	537	0.0225741	regulation of intracellular transport
GO:0010033	10	3487	0.0239352	response to organic substance
GO:0045935	8	2060	0.0263583	positive regulation of nucleobase-containing compound metabolic process
GO:0051173	8	2108	0.0311465	positive regulation of nitrogen compound metabolic process
GO:0051341	3	90	0.0325021	regulation of oxidoreductase activity
GO:0071417	5	588	0.0348715	cellular response to organonitrogen compound
GO:0048583	11	4515	0.0348835	regulation of response to stimulus
GO:0051247	7	1526	0.0349726	positive regulation of protein metabolic process
GO:0008152	22	20927	0.0361253	metabolic process
GO:0048869	10	3694	0.0397487	cellular developmental process
GO:0070887	9	2904	0.0408375	cellular response to chemical stimulus
GO:0019219	14	7797	0.0445536	regulation of nucleobase-containing compound metabolic process
GO:0043523	4	300	0.0445575	regulation of neuron apoptotic process
GO:0006975	2	14	0.04663	DNA damage induced protein phosphorylation
GO:0051246	9	2954	0.0467915	regulation of protein metabolic process
GO:0030330	3	102	0.0472767	DNA damage response, signal transduction by p53 class mediator

Table 3: Overrepresented GO terms with the standard enrichment

2 Network-based enrichment

GO Term	N1	N2	P-value	Description
GO:0044270	18	3027	1.33939e-11	cellular nitrogen compound catabolic process
GO:0007346	14	1240	1.9923e-11	regulation of mitotic cell cycle
GO:1901361	18	3169	2.99687e-11	organic cyclic compound catabolic process
GO:0009057	16	2318	1.61118e-10	macromolecule catabolic process
GO:0044265	15	1850	1.63841e-10	cellular macromolecule catabolic process
GO:0045786	12	824	1.86705e-10	negative regulation of cell cycle
GO:0034655	17	2890	1.89632e-10	nucleobase-containing compound catabolic process
GO:0046700	17	3029	4.11607e-10	heterocycle catabolic process
GO:0019439	17	3055	4.73864e-10	aromatic compound catabolic process
GO:1901565	16	2901	5.20789e-09	organonitrogen compound catabolic process
GO:0071900	13	1492	7.62297e-09	regulation of protein serine/threonine kinase activity
GO:0008406	9	424	2.05663e-08	gonad development
GO:0009887	13	1719	4.52925e-08	organ morphogenesis
GO:0003002	11	988	5.27589e-08	regionalization
GO:0016568	12	1339	5.55551e-08	chromatin modification
GO:0031400	12	1368	7.12635e-08	negative regulation of protein modification process
GO:0007126	8	313	8.81409e-08	meiotic nuclear division
GO:0048608	11	1062	1.14324e-07	reproductive structure development
GO:0042592	16	3571	1.26103e-07	homeostatic process
GO:0031401	15	2947	1.38666e-07	positive regulation of protein modification process
GO:0048646	15	2968	1.53498e-07	anatomical structure formation involved in morphogenesis
GO:0071363	13	1903	1.61681e-07	cellular response to growth factor stimulus
GO:0007389	12	1481	1.78882e-07	pattern specification process
GO:0051302	10	796	1.81247e-07	regulation of cell division
GO:1901136	14	2479	2.42367e-07	carbohydrate derivative catabolic process
GO:0071407	11	1148	2.62505e-07	cellular response to organic cyclic compound
GO:0070848	13	1987	2.77051e-07	response to growth factor
GO:0008283	14	2526	3.11493e-07	cell proliferation
GO:0006275	8	373	3.5508e-07	regulation of DNA replication
GO:0006325	12	1572	3.56385e-07	chromatin organization
GO:0009952	9	585	3.58728e-07	anterior/posterior pattern specification
GO:0007276	12	1582	3.83444e-07	gamete generation
GO:0051053	7	228	5.2413e-07	negative regulation of DNA metabolic process
GO:0070304	7	232	5.91943e-07	positive regulation of stress-activated protein kinase signaling cascade
GO:0032270	15	3279	6.36042e-07	positive regulation of cellular protein metabolic process
GO:0051248	13	2189	9.22309e-07	negative regulation of protein metabolic process
GO:0023014	9	653	9.46355e-07	signal transduction by phosphorylation
GO:0009607	13	2197	9.64941e-07	response to biotic stimulus
GO:0043409	8	424	9.78607e-07	negative regulation of MAPK cascade
GO:0033043	14	2759	1.0087e-06	regulation of organelle organization
GO:0050707	8	430	1.09341e-06	regulation of cytokine secretion
GO:2001233	11	1313	1.09369e-06	regulation of apoptotic signaling pathway
GO:0018193	13	2224	1.12248e-06	peptidyl-amino acid modification
GO:0071216	8	432	1.13419e-06	cellular response to biotic stimulus
GO:0000280	10	969	1.22899e-06	nuclear division
GO:0046434	13	2260	1.36913e-06	organophosphate catabolic process
GO:0032269	12	1804	1.73605e-06	negative regulation of cellular protein metabolic process
GO:0007265	8	457	1.76731e-06	Ras protein signal transduction
GO:0000122	13	2314	1.83295e-06	negative regulation of transcription from RNA polymerase II promoter
GO:0080135	11	1388	1.96841e-06	regulation of cellular response to stress
GO:0048469	8	468	2.13133e-06	cell maturation
GO:0050878	12	1837	2.13666e-06	regulation of body fluid levels
GO:1901987	9	721	2.26035e-06	regulation of cell cycle phase transition
GO:0007283	11	1413	2.37676e-06	spermatogenesis
GO:0031349	9	726	2.40162e-06	positive regulation of defense response
GO:0001934	13	2371	2.47477e-06	positive regulation of protein phosphorylation
GO:0048232	11	1420	2.50401e-06	male gamete generation
GO:0009967	15	3621	2.59535e-06	positive regulation of signal transduction
GO:1901988	8	482	2.68759e-06	negative regulation of cell cycle phase transition
GO:2000113	15	3633	2.7195e-06	negative regulation of cellular macromolecule biosynthetic process

Table 4: Overrepresented terms with the network-based enrichment. Only terms not detected with the standard method.

GO Term	N1	N2	P-value	Description
GO:0048285	10	1056	2.82406e-06	organelle fission
GO:0022602	7	294	3.08567e-06	ovulation cycle process
GO:1902533	13	2418	3.15181e-06	positive regulation of intracellular signal transduction
GO:0051129	12	1913	3.39666e-06	negative regulation of cellular component organization
GO:0045934	15	3691	3.40068e-06	negative regulation of nucleobase-containing compound metabolic process
GO:0009203	12	1920	3.54133e-06	ribonucleoside triphosphate catabolic process
GO:0009207	12	1920	3.54133e-06	purine ribonucleoside triphosphate catabolic process
GO:0008584	7	300	3.55054e-06	male gonad development
GO:0009146	12	1925	3.64807e-06	purine nucleoside triphosphate catabolic process
GO:0042493	11	1473	3.68478e-06	response to drug
GO:0009143	12	1934	3.84758e-06	nucleoside triphosphate catabolic process
GO:0080134	14	3072	4.18217e-06	regulation of response to stress
GO:2001020	7	308	4.26218e-06	regulation of response to DNA damage stimulus
GO:0010558	15	3771	4.60055e-06	negative regulation of macromolecule biosynthetic process
GO:0006152	12	1974	4.85957e-06	purine nucleoside catabolic process
GO:0046130	12	1974	4.85957e-06	purine ribonucleoside catabolic process
GO:0042110	9	787	4.86773e-06	T cell activation
GO:0051172	15	3798	5.08659e-06	negative regulation of nitrogen compound metabolic process
GO:0045597	13	2514	5.08722e-06	positive regulation of cell differentiation
GO:0010647	15	3801	5.14343e-06	positive regulation of cell communication
GO:0009653	16	4591	5.63805e-06	anatomical structure morphogenesis
GO:0042454	12	2000	5.64097e-06	ribonucleoside catabolic process
GO:0045017	8	530	5.66323e-06	glycerolipid biosynthetic process
GO:0010942	11	1542	5.96419e-06	positive regulation of cell death
GO:0009154	12	2010	5.97063e-06	purine ribonucleotide catabolic process
GO:0009261	12	2011	6.00456e-06	ribonucleotide catabolic process
GO:0060706	5	75	6.0241e-06	cell differentiation involved in embryonic placenta development
GO:0000165	8	537	6.27653e-06	MAPK cascade
GO:0009888	13	2570	6.66636e-06	tissue development
GO:0042176	9	816	6.6776e-06	regulation of protein catabolic process
GO:0006461	14	3196	7.04158e-06	protein complex assembly
GO:0006195	12	2041	7.10673e-06	purine nucleotide catabolic process
GO:0042326	10	1164	7.21748e-06	negative regulation of phosphorylation
GO:0009164	12	2045	7.26678e-06	nucleoside catabolic process
GO:1901658	12	2055	7.68132e-06	glycosyl compound catabolic process
GO:0072523	12	2067	8.20696e-06	purine-containing compound catabolic process
GO:0031589	8	556	8.24067e-06	cell-substrate adhesion
GO:0001775	12	2070	8.34337e-06	cell activation
GO:0048609	12	2109	1.03137e-05	multicellular organismal reproductive process
GO:0009166	12	2116	1.0709e-05	nucleotide catabolic process
GO:0031344	11	1640	1.13813e-05	regulation of cell projection organization
GO:1901292	12	2130	1.1541e-05	nucleoside phosphate catabolic process
GO:0051223	11	1645	1.17498e-05	regulation of protein transport
GO:0006897	10	1228	1.2066e-05	endocytosis
GO:0051251	9	874	1.21499e-05	positive regulation of lymphocyte activation
GO:0042327	13	2706	1.25309e-05	positive regulation of phosphorylation
GO:0009205	12	2149	1.27638e-05	purine ribonucleoside triphosphate metabolic process
GO:0007411	10	1242	1.34509e-05	axon guidance
GO:0009144	12	2159	1.34535e-05	purine nucleoside triphosphate metabolic process
GO:0051403	6	197	1.34584e-05	stress-activated MAPK cascade
GO:0097485	10	1243	1.3555e-05	neuron projection guidance
GO:0001932	14	3361	1.3631e-05	regulation of protein phosphorylation
GO:0051348	9	887	1.38155e-05	negative regulation of transferase activity
GO:0042476	7	366	1.40682e-05	odontogenesis
GO:0009199	12	2170	1.42511e-05	ribonucleoside triphosphate metabolic process
GO:0031098	6	201	1.51803e-05	stress-activated protein kinase signaling cascade
GO:0009617	8	605	1.59429e-05	response to bacterium
GO:0019318	8	609	1.67838e-05	hexose metabolic process
GO:0009141	12	2225	1.89189e-05	nucleoside triphosphate metabolic process
GO:1902532	10	1289	1.91941e-05	negative regulation of intracellular signal transduction

Table 5: Overrepresented terms with the network-based enrichment. Only terms not detected with the standard method.

GO Term	N1	N2	P-value	Description
GO:0021533	5	95	2.00416e-05	cell differentiation in hindbrain
GO:0046425	6	211	2.02981e-05	regulation of JAK-STAT cascade
GO:0061024	11	1750	2.24268e-05	membrane organization
GO:0050671	7	392	2.25821e-05	positive regulation of lymphocyte proliferation
GO:0050680	7	392	2.25821e-05	negative regulation of epithelial cell proliferation
GO:0002696	9	941	2.30841e-05	positive regulation of leukocyte activation
GO:0007507	8	638	2.41168e-05	heart development
GO:0032946	7	396	2.42175e-05	positive regulation of mononuclear cell proliferation
GO:0001525	9	949	2.48427e-05	angiogenesis
GO:1903047	11	1769	2.50986e-05	mitotic cell cycle process
GO:0010038	9	951	2.53003e-05	response to metal ion
GO:0000077	6	219	2.53541e-05	DNA damage checkpoint
GO:0045931	5	100	2.59833e-05	positive regulation of mitotic cell cycle
GO:0045787	7	401	2.64024e-05	positive regulation of cell cycle
GO:0050870	8	647	2.68949e-05	positive regulation of T cell activation
GO:0070665	7	403	2.73221e-05	positive regulation of leukocyte proliferation
GO:0001933	9	963	2.82051e-05	negative regulation of protein phosphorylation
GO:0046486	9	970	3.00316e-05	glycerolipid metabolic process
GO:0010563	10	1353	3.04892e-05	negative regulation of phosphorus metabolic process
GO:0045936	10	1353	3.04892e-05	negative regulation of phosphate metabolic process
GO:0045639	6	226	3.05939e-05	positive regulation of myeloid cell differentiation
GO:0016485	8	661	3.17672e-05	protein processing
GO:0007292	5	105	3.325e-05	female gamete generation
GO:0021700	8	666	3.36835e-05	developmental maturation
GO:0032874	6	230	3.39702e-05	positive regulation of stress-activated MAPK cascade
GO:0006469	8	668	3.44776e-05	negative regulation of protein kinase activity
GO:0045862	6	233	3.66999e-05	positive regulation of proteolysis
GO:0021559	3	7	3.67501e-05	trigeminal nerve development
GO:0050867	9	996	3.77588e-05	positive regulation of cell activation
GO:0005996	8	680	3.95917e-05	monosaccharide metabolic process
GO:0070201	11	1849	3.97663e-05	regulation of establishment of protein localization
GO:1901654	7	426	4.00173e-05	response to ketone
GO:0048534	8	682	4.05049e-05	hematopoietic or lymphoid organ development
GO:0043065	10	1406	4.39661e-05	positive regulation of apoptotic process
GO:0070232	5	111	4.40133e-05	regulation of T cell apoptotic process
GO:0043433	7	432	4.40518e-05	negative regulation of sequence-specific DNA binding transcription factor activity
GO:0010562	13	3001	4.41356e-05	positive regulation of phosphorus metabolic process
GO:0045937	13	3001	4.41356e-05	positive regulation of phosphate metabolic process
GO:0065003	14	3681	4.46487e-05	macromolecular complex assembly
GO:0046128	12	2404	4.52797e-05	purine ribonucleoside metabolic process
GO:1901990	8	692	4.535e-05	regulation of mitotic cell cycle phase transition
GO:0042278	12	2412	4.70036e-05	purine nucleoside metabolic process
GO:0043068	10	1416	4.70325e-05	positive regulation of programmed cell death
GO:0031570	6	243	4.71441e-05	DNA integrity checkpoint
GO:0010035	10	1420	4.83116e-05	response to inorganic substance
GO:0042129	7	439	4.91904e-05	regulation of T cell proliferation
GO:0051707	10	1426	5.02877e-05	response to other organism
GO:0045664	11	1894	5.10413e-05	regulation of neuron differentiation
GO:0007093	6	247	5.19577e-05	mitotic cell cycle checkpoint
GO:0045859	12	2438	5.30224e-05	regulation of protein kinase activity
GO:0051783	7	444	5.31648e-05	regulation of nuclear division
GO:0033673	8	709	5.47419e-05	negative regulation of kinase activity
GO:0050708	8	710	5.53432e-05	regulation of protein secretion
GO:2000106	6	251	5.71707e-05	regulation of leukocyte apoptotic process
GO:0010948	8	717	5.97147e-05	negative regulation of cell cycle process
GO:1901991	7	457	6.47966e-05	negative regulation of mitotic cell cycle phase transition
GO:0051047	9	1063	6.62522e-05	positive regulation of secretion
GO:0018393	6	258	6.7333e-05	internal peptidyl-lysine acetylation
GO:0009119	12	2491	6.74992e-05	ribonucleoside metabolic process
GO:0018394	6	260	7.0496e-05	peptidyl-lysine acetylation

Table 6: Overrepresented terms with the network-based enrichment. Only terms not detected with the standard method.

GO Term	N1	N2	P-value	Description
GO:0051050	12	2507	7.25225e-05	positive regulation of transport
GO:0046474	7	465	7.29764e-05	glycerophospholipid biosynthetic process
GO:0071496	8	736	7.31198e-05	cellular response to external stimulus
GO:0010243	12	2509	7.3174e-05	response to organonitrogen compound
GO:0045321	10	1486	7.43749e-05	leukocyte activation
GO:0051604	8	741	7.70529e-05	protein maturation
GO:0009150	12	2531	8.06935e-05	purine ribonucleotide metabolic process
GO:0051090	9	1091	8.28869e-05	regulation of sequence-specific DNA binding transcription factor activity
GO:0006475	6	269	8.62922e-05	internal protein amino acid acetylation
GO:0010954	6	271	9.01722e-05	positive regulation of protein processing
GO:1903319	6	271	9.01722e-05	positive regulation of protein maturation
GO:0007264	10	1517	9.04492e-05	small GTPase mediated signal transduction
GO:0009116	12	2561	9.20721e-05	nucleoside metabolic process
GO:0042102	6	273	9.41952e-05	positive regulation of T cell proliferation
GO:0042475	6	273	9.41952e-05	odontogenesis of dentin-containing tooth
GO:0051094	13	3200	9.58832e-05	positive regulation of developmental process
GO:0043549	12	2576	9.82882e-05	regulation of kinase activity
GO:0009259	12	2583	0.000101317	ribonucleotide metabolic process
GO:0006163	12	2586	0.00010264	purine nucleotide metabolic process
GO:0010464	5	132	0.000105223	regulation of mesenchymal cell proliferation
GO:0019693	12	2596	0.000107166	ribose phosphate metabolic process
GO:0045892	13	3247	0.000114287	negative regulation of transcription, DNA-templated
GO:1901657	12	2611	0.000114292	glycosyl compound metabolic process
GO:0048731	12	2612	0.000114782	system development
GO:0008610	10	1559	0.000117121	lipid biosynthetic process
GO:0044092	13	3258	0.000119033	negative regulation of molecular function
GO:0009896	8	786	0.00012151	positive regulation of catabolic process
GO:0046427	5	136	0.000122219	positive regulation of JAK-STAT cascade
GO:0043207	11	2065	0.000124766	response to external biotic stimulus
GO:0007420	8	791	0.000127602	brain development
GO:0044344	7	505	0.000128305	cellular response to fibroblast growth factor stimulus
GO:1902679	13	3295	0.000136345	negative regulation of RNA biosynthetic process
GO:0050954	7	510	0.000137231	sensory perception of mechanical stimulus
GO:0050770	7	511	0.000139078	regulation of axonogenesis
GO:0006473	6	292	0.000140377	protein acetylation
GO:0035412	4	50	0.000142421	regulation of catenin import into nucleus
GO:0051046	11	2094	0.000144043	regulation of secretion
GO:0033135	6	294	0.000146169	regulation of peptidyl-serine phosphorylation
GO:1901214	8	808	0.000150333	regulation of neuron death
GO:0009636	7	517	0.000150608	response to toxic substance
GO:0010565	7	517	0.000150608	regulation of cellular ketone metabolic process
GO:0071774	7	519	0.000154627	response to fibroblast growth factor
GO:0031347	10	1612	0.000160599	regulation of defense response
GO:0071158	5	144	0.000162744	positive regulation of cell cycle arrest
GO:0008104	10	1615	0.000163441	protein localization
GO:0022604	10	1615	0.000163441	regulation of cell morphogenesis
GO:1901699	10	1626	0.000174244	cellular response to nitrogen compound
GO:0002700	6	303	0.000174733	regulation of production of molecular mediator of immune response
GO:0051253	13	3369	0.000178006	negative regulation of RNA metabolic process
GO:0072521	12	2722	0.000181827	purine-containing compound metabolic process
GO:0032870	10	1636	0.00018461	cellular response to hormone stimulus
GO:0033036	10	1642	0.000191088	macromolecule localization
GO:0050877	12	2735	0.000191729	neurological system process
GO:0006915	11	2154	0.00019261	apoptotic process
GO:0070372	7	536	0.000192617	regulation of ERK1 and ERK2 cascade
GO:0046677	5	149	0.000193053	response to antibiotic
GO:0046034	9	1204	0.000193188	ATP metabolic process
GO:0030168	7	539	0.00020008	platelet activation
GO:0051092	6	312	0.000207757	positive regulation of NF-kappaB transcription factor activity
GO:0007052	4	55	0.000210433	mitotic spindle organization

Table 7: Overrepresented terms with the network-based enrichment. Only terms not detected with the standard method.

GO Term	N1	N2	P-value	Description
GO:0019216	8	845	0.000212193	regulation of lipid metabolic process
GO:0030522	7	544	0.000213063	intracellular receptor signaling pathway
GO:0032496	8	847	0.000216084	response to lipopolysaccharide
GO:0016032	10	1665	0.00021783	viral process
GO:0044403	10	1665	0.00021783	symbiosis, encompassing mutualism through parasitism
GO:0032535	7	546	0.000218453	regulation of cellular component size
GO:0036296	4	56	0.000226517	response to increased oxygen levels
GO:0055093	4	56	0.000226517	response to hyperoxia
GO:0050727	8	853	0.000228133	regulation of inflammatory response
GO:0006470	7	550	0.000229579	protein dephosphorylation
GO:0008654	7	550	0.000229579	phospholipid biosynthetic process
GO:0060627	9	1229	0.000230328	regulation of vesicle-mediated transport
GO:0060744	3	12	0.00023048	mammary gland branching involved in thelarche
GO:0014070	12	2783	0.000232646	response to organic cyclic compound
GO:0032880	11	2200	0.000239265	regulation of protein localization
GO:0044764	10	1682	0.000239675	multi-organism cellular process
GO:0046631	5	159	0.00026708	alpha-beta T cell activation
GO:0051338	12	2819	0.000268333	regulation of transferase activity
GO:0007088	6	327	0.000274168	regulation of mitosis
GO:0070741	4	59	0.000280303	response to interleukin-6
GO:0022603	12	2832	0.000282385	regulation of anatomical structure morphogenesis
GO:0033157	8	878	0.000284793	regulation of intracellular protein transport
GO:0070302	7	569	0.000289221	regulation of stress-activated protein kinase signaling cascade
GO:0010717	5	162	0.000293196	regulation of epithelial to mesenchymal transition
GO:0012501	11	2247	0.000297133	programmed cell death
GO:0030308	7	575	0.000310585	negative regulation of cell growth
GO:0009167	9	1274	0.000313142	purine ribonucleoside monophosphate metabolic process
GO:0009126	9	1275	0.000315245	purine nucleoside monophosphate metabolic process
GO:0045926	8	891	0.000318782	negative regulation of growth
GO:0001764	7	578	0.000321758	neuron migration
GO:0001818	7	578	0.000321758	negative regulation of cytokine production
GO:0006260	7	578	0.000321758	DNA replication
GO:0010975	9	1281	0.000328126	regulation of neuron projection development
GO:0044703	7	580	0.000329394	multi-organism reproductive process
GO:2001236	7	581	0.000333269	regulation of extrinsic apoptotic signaling pathway
GO:0031346	8	897	0.000335614	positive regulation of cell projection organization
GO:0071902	8	897	0.000335614	positive regulation of protein serine/threonine kinase activity
GO:1902582	12	2881	0.000341516	single-organism intracellular transport
GO:0019637	14	4312	0.000343521	organophosphate metabolic process
GO:0048468	11	2280	0.000344934	cell development
GO:0010629	13	3561	0.000345397	negative regulation of gene expression
GO:0050863	8	912	0.000381087	regulation of T cell activation
GO:0001817	10	1774	0.000395101	regulation of cytokine production
GO:0070228	5	172	0.000395232	regulation of lymphocyte apoptotic process
GO:0002237	8	918	0.000400708	response to molecule of bacterial origin
GO:1900180	7	602	0.000424044	regulation of protein localization to nucleus
GO:0007067	7	605	0.000438574	mitotic nuclear division
GO:0050670	7	605	0.000438574	regulation of lymphocyte proliferation
GO:0002521	8	929	0.000438955	leukocyte differentiation
GO:0016570	8	929	0.000438955	histone modification
GO:0003006	11	2335	0.000440027	developmental process involved in reproduction
GO:0043405	8	930	0.000442582	regulation of MAP kinase activity
GO:0048511	8	930	0.000442582	rhythmic process
GO:0050804	8	931	0.000446235	regulation of synaptic transmission
GO:0090183	5	177	0.000455849	regulation of kidney development
GO:0010721	7	609	0.000458605	negative regulation of cell development
GO:0032944	7	609	0.000458605	regulation of mononuclear cell proliferation
GO:0016569	8	935	0.000461112	covalent chromatin modification
GO:0001568	6	358	0.000467572	blood vessel development
GO:0071222	6	359	0.00047531	cellular response to lipopolysaccharide

Table 8: Overrepresented terms with the network-based enrichment. Only terms not detected with the standard method.

GO Term	N1	N2	P-value	Description
GO:0009161	9	1341	0.000484551	ribonucleoside monophosphate metabolic process
GO:0044702	13	3664	0.000485052	single organism reproductive process
GO:0002683	8	945	0.000500187	negative regulation of immune system process
GO:0044711	13	3675	0.00050265	single-organism biosynthetic process
GO:0010467	9	1348	0.000506482	gene expression
GO:0070663	7	621	0.000523398	regulation of leukocyte proliferation
GO:0021953	6	365	0.000523988	central nervous system neuron differentiation
GO:0050767	11	2377	0.000527746	regulation of neurogenesis
GO:0043408	10	1837	0.000547631	regulation of MAPK cascade
GO:0051259	9	1363	0.000556415	protein oligomerization
GO:0038093	7	627	0.000558593	Fc receptor signaling pathway
GO:0009123	9	1367	0.00057044	nucleoside monophosphate metabolic process
GO:0043122	7	630	0.000576929	regulation of I-kappaB kinase/NF-kappaB signaling
GO:0046330	5	187	0.000599153	positive regulation of JNK cascade
GO:0045732	6	374	0.000604654	positive regulation of protein catabolic process
GO:0008286	6	375	0.000614214	insulin receptor signaling pathway
GO:0002757	8	972	0.000620257	immune response-activating signal transduction
GO:0098542	8	972	0.000620257	defense response to other organism
GO:0001649	6	378	0.000643648	osteoblast differentiation
GO:0016192	12	3055	0.0006526	vesicle-mediated transport
GO:0001558	9	1390	0.000657223	regulation of cell growth
GO:0048585	13	3759	0.000657347	negative regulation of response to stimulus
GO:0071364	4	73	0.000666072	cellular response to epidermal growth factor stimulus
GO:0034612	6	381	0.000674234	response to tumor necrosis factor
GO:0002252	9	1396	0.000681675	immune effector process
GO:0071219	6	382	0.000684694	cellular response to molecule of bacterial origin
GO:0090407	9	1397	0.000685829	organophosphate biosynthetic process
GO:0006401	6	386	0.000727868	RNA catabolic process
GO:0051301	6	386	0.000727868	cell division
GO:0030212	4	75	0.000743123	hyaluronan metabolic process
GO:0071241	6	388	0.000750282	cellular response to inorganic substance
GO:0044419	10	1908	0.000780034	interspecies interaction between organisms
GO:0000003	4	76	0.000784049	reproduction
GO:0043406	7	661	0.000798176	positive regulation of MAP kinase activity
GO:0050730	7	663	0.000814621	regulation of peptidyl-tyrosine phosphorylation
GO:0008543	6	394	0.00082096	fibroblast growth factor receptor signaling pathway
GO:0035148	6	396	0.000845702	tube formation
GO:0006978	3	18	0.000852564	DNA damage response, signal transduction by p53 class mediator resulting in transcription of p21 class med
GO:0006200	8	1017	0.000875728	ATP catabolic process
GO:0045833	5	202	0.000878814	negative regulation of lipid metabolic process
GO:0045087	10	1935	0.000889036	innate immune response
GO:0031669	6	400	0.000897029	cellular response to nutrient levels
GO:0046822	7	675	0.000919421	regulation of nucleocytoplasmic transport
GO:0043543	6	403	0.000937187	protein acylation
GO:0051091	7	677	0.000937948	positive regulation of sequence-specific DNA binding transcription factor activity
GO:0009158	8	1027	0.000943416	ribonucleoside monophosphate catabolic process
GO:0009169	8	1027	0.000943416	purine ribonucleoside monophosphate catabolic process
GO:0009128	8	1028	0.000950429	purine nucleoside monophosphate catabolic process
GO:0016311	8	1031	0.000971726	dephosphorylation
GO:0005975	11	2526	0.000978794	carbohydrate metabolic process
GO:0009125	8	1033	0.000986154	nucleoside monophosphate catabolic process
GO:0051222	8	1034	0.000993437	positive regulation of protein transport
GO:0042772	3	19	0.00101196	DNA damage response, signal transduction resulting in transcription
GO:1901701	11	2540	0.00103515	cellular response to oxygen-containing compound
GO:0051054	6	411	0.00105156	positive regulation of DNA metabolic process
GO:0009117	12	3194	0.00106396	nucleotide metabolic process
GO:0048011	7	692	0.00108722	neurotrophin TRK receptor signaling pathway
GO:0045665	5	213	0.0011429	negative regulation of neuron differentiation
GO:0008544	6	418	0.00116083	epidermis development
GO:0007610	10	1993	0.00116975	behavior

Table 9: Overrepresented terms with the network-based enrichment. Only terms not detected with the standard method.

GO Term	N1	N2	P-value	Description
GO:0006753	12	3223	0.0011747	nucleoside phosphate metabolic process
GO:0051960	11	2584	0.00123159	regulation of nervous system development
GO:0043279	6	424	0.00126177	response to alkaloid
GO:0051271	7	710	0.00129238	negative regulation of cellular component movement
GO:0034097	10	2017	0.0013071	response to cytokine
GO:0010944	3	21	0.00138772	negative regulation of transcription by competitive promoter binding
GO:0007173	6	432	0.00140749	epidermal growth factor receptor signaling pathway
GO:0043551	4	88	0.00141727	regulation of phosphatidylinositol 3-kinase activity
GO:1901796	4	88	0.00141727	regulation of signal transduction by p53 class mediator
GO:0038179	7	720	0.00141983	neurotrophin signaling pathway
GO:0006650	7	721	0.00143314	glycerophospholipid metabolic process
GO:0006006	6	435	0.00146557	glucose metabolic process
GO:0090068	7	724	0.0014737	positive regulation of cell cycle process
GO:0043086	11	2637	0.00151186	negative regulation of catalytic activity
GO:0018205	6	438	0.00152562	peptidyl-lysine modification
GO:0071156	5	226	0.00153211	regulation of cell cycle arrest
GO:0006886	10	2070	0.00166191	intracellular protein transport
GO:0009895	6	445	0.00167362	negative regulation of catabolic process
GO:0035710	4	92	0.00169513	CD4-positive, alpha-beta T cell activation
GO:0002253	8	1110	0.00170154	activation of immune response
GO:0070167	5	231	0.00170706	regulation of biomineral tissue development
GO:0030198	8	1111	0.00171317	extracellular matrix organization
GO:0060341	12	3341	0.00174007	regulation of cellular localization
GO:0010769	8	1115	0.00176041	regulation of cell morphogenesis involved in differentiation
GO:0043124	4	93	0.00177051	negative regulation of I-kappaB kinase/NF-kappaB signaling
GO:2001021	4	93	0.00177051	negative regulation of response to DNA damage stimulus
GO:0043062	8	1116	0.0017724	extracellular structure organization
GO:0006979	8	1117	0.00178445	response to oxidative stress
GO:0038127	6	452	0.00183322	ERBB signaling pathway
GO:0051093	11	2690	0.0018476	negative regulation of developmental process
GO:1901698	11	2693	0.00186845	response to nitrogen compound
GO:0045088	7	751	0.00188439	regulation of innate immune response
GO:2000648	5	237	0.00193747	positive regulation of stem cell proliferation
GO:0050768	6	457	0.00195472	negative regulation of neurogenesis
GO:0043524	6	459	0.00200513	negative regulation of neuron apoptotic process
GO:0008202	7	760	0.0020411	steroid metabolic process
GO:0055086	12	3391	0.00204579	nucleobase-containing small molecule metabolic process
GO:0009615	7	761	0.00205917	response to virus
GO:0009894	12	3408	0.00216022	regulation of catabolic process
GO:0051056	7	769	0.00220865	regulation of small GTPase mediated signal transduction
GO:0043966	4	99	0.00227644	histone H3 acetylation
GO:0051224	6	470	0.00230188	negative regulation of protein transport
GO:0007154	11	2752	0.00232339	cell communication
GO:0048771	5	246	0.00232853	tissue remodeling
GO:0097284	3	25	0.0023955	hepatocyte apoptotic process
GO:0048568	6	476	0.00247835	embryonic organ development
GO:0060249	7	783	0.00249225	anatomical structure homeostasis
GO:0001101	8	1170	0.00253287	response to acid chemical
GO:0033628	4	102	0.00256634	regulation of cell adhesion mediated by integrin
GO:0031668	6	479	0.00257067	cellular response to extracellular stimulus
GO:0042752	5	251	0.00257129	regulation of circadian rhythm
GO:0051130	11	2782	0.00259053	positive regulation of cellular component organization
GO:0000086	5	254	0.00272627	G2/M transition of mitotic cell cycle
GO:0044839	5	254	0.00272627	cell cycle G2/M phase transition
GO:0016049	6	484	0.0027308	cell growth
GO:0007006	4	104	0.0027743	mitochondrial membrane organization
GO:0008361	4	104	0.0027743	regulation of cell size
GO:0034146	4	104	0.0027743	toll-like receptor 5 signaling pathway
GO:0007605	6	487	0.00283074	sensory perception of sound
GO:0038095	6	487	0.00283074	Fc-epsilon receptor signaling pathway

Table 10: Overrepresented terms with the network-based enrichment. Only terms not detected with the standard method.

GO Term	N1	N2	P-value	Description
GO:0072331	5	256	0.00283362	signal transduction by p53 class mediator
GO:0046683	6	488	0.00286471	response to organophosphorus
GO:0010771	4	105	0.00288285	negative regulation of cell morphogenesis involved in differentiation
GO:0034166	4	105	0.00288285	toll-like receptor 10 signaling pathway
GO:0060326	6	489	0.00289902	cell chemotaxis
GO:0007155	11	2817	0.00293645	cell adhesion
GO:0044255	11	2817	0.00293645	cellular lipid metabolic process
GO:0015031	12	3506	0.00293927	protein transport
GO:0046649	8	1196	0.00298925	lymphocyte activation
GO:0042993	4	106	0.00299454	positive regulation of transcription factor import into nucleus
GO:0009725	11	2824	0.00301037	response to hormone
GO:0022610	11	2824	0.00301037	biological adhesion
GO:0071396	8	1198	0.00302711	cellular response to lipid
GO:0008219	11	2827	0.00304255	cell death
GO:0071480	3	27	0.0030437	cellular response to gamma radiation
GO:0034121	4	107	0.00310942	regulation of toll-like receptor signaling pathway
GO:0035116	4	107	0.00310942	embryonic hindlimb morphogenesis
GO:0040012	10	2224	0.00322015	regulation of locomotion
GO:0030111	7	814	0.00323092	regulation of Wnt signaling pathway
GO:0016265	11	2848	0.00327653	death
GO:0002433	5	264	0.00329695	immune response-regulating cell surface receptor signaling pathway involved in phagocytosis
GO:0038094	5	264	0.00329695	Fc-gamma receptor signaling pathway
GO:0038096	5	264	0.00329695	Fc-gamma receptor signaling pathway involved in phagocytosis
GO:0016071	8	1213	0.00332436	mRNA metabolic process
GO:0090066	7	820	0.0033933	regulation of anatomical structure size
GO:0002431	5	266	0.00342163	Fc receptor mediated stimulatory signaling pathway
GO:0046328	6	505	0.00349547	regulation of JNK cascade
GO:0031638	5	269	0.00361562	zymogen activation
GO:0032147	7	832	0.00373873	activation of protein kinase activity
GO:0014067	3	29	0.00379886	negative regulation of phosphatidylinositol 3-kinase signaling
GO:0032868	7	834	0.00379908	response to insulin
GO:0010959	7	835	0.00382953	regulation of metal ion transport
GO:0002053	4	113	0.00386891	positive regulation of mesenchymal cell proliferation
GO:0043550	4	113	0.00386891	regulation of lipid kinase activity
GO:0045216	6	516	0.00396118	cell-cell junction organization
GO:0040013	7	840	0.00398504	negative regulation of locomotion
GO:0022409	4	114	0.00400777	positive regulation of cell-cell adhesion
GO:0042742	6	519	0.00409664	defense response to bacterium
GO:0046777	6	520	0.00414264	protein autophosphorylation
GO:1903320	6	520	0.00414264	regulation of protein modification by small protein conjugation or removal
GO:0023052	10	2287	0.00416069	signaling
GO:0044700	10	2287	0.00416069	single organism signaling
GO:0022408	4	116	0.00429655	negative regulation of cell-cell adhesion
GO:0090150	6	524	0.00433088	establishment of protein localization to membrane
GO:1901215	6	524	0.00433088	negative regulation of neuron death
GO:0097191	5	280	0.00440243	extrinsic apoptotic signaling pathway
GO:0038123	4	117	0.00444661	toll-like receptor TLR1:TLR2 signaling pathway
GO:0038124	4	117	0.00444661	toll-like receptor TLR6:TLR2 signaling pathway
GO:0042306	6	527	0.00447658	regulation of protein import into nucleus
GO:0040008	10	2308	0.00452387	regulation of growth
GO:0051249	8	1264	0.00452995	regulation of lymphocyte activation
GO:0008203	5	282	0.00455897	cholesterol metabolic process
GO:0043410	8	1269	0.0046661	positive regulation of MAPK cascade
GO:0051345	11	2953	0.00470361	positive regulation of hydrolase activity
GO:0051705	5	285	0.00480199	multi-organism behavior
GO:1903034	8	1274	0.00480573	regulation of response to wounding
GO:0035666	4	120	0.00492021	TRIF-dependent toll-like receptor signaling pathway
GO:2000177	5	288	0.00505512	regulation of neural precursor cell proliferation
GO:0007254	4	121	0.00508612	JNK cascade
GO:0034162	4	121	0.00508612	toll-like receptor 9 signaling pathway

Table 11: Overrepresented terms with the network-based enrichment. Only terms not detected with the standard method.

GO Term	N1	N2	P-value	Description
GO:0048666	6	539	0.00510025	neuron development
GO:0060284	11	2979	0.00513275	regulation of cell development
GO:0010595	4	122	0.00525613	positive regulation of endothelial cell migration
GO:0031331	6	542	0.00526681	positive regulation of cellular catabolic process
GO:0045944	11	2989	0.00530685	positive regulation of transcription from RNA polymerase II promoter
GO:0044802	8	1291	0.00530762	single-organism membrane organization
GO:0002756	4	124	0.00560877	MyD88-independent toll-like receptor signaling pathway
GO:0034134	4	124	0.00560877	toll-like receptor 2 signaling pathway
GO:0070373	4	124	0.00560877	negative regulation of ERK1 and ERK2 cascade
GO:0033599	3	33	0.00566209	regulation of mammary gland epithelial cell proliferation
GO:0045184	12	3728	0.00570539	establishment of protein localization
GO:0022407	5	297	0.00587822	regulation of cell-cell adhesion
GO:0014074	6	555	0.00604087	response to purine-containing compound
GO:0006954	8	1314	0.00605767	inflammatory response
GO:0044087	9	1812	0.0060902	regulation of cellular component biogenesis
GO:0090287	6	557	0.00616783	regulation of cellular response to growth factor stimulus
GO:0006282	4	127	0.00617028	regulation of DNA repair
GO:0051225	4	127	0.00617028	spindle assembly
GO:0051784	4	127	0.00617028	negative regulation of nuclear division
GO:0045662	3	34	0.00620725	negative regulation of myoblast differentiation
GO:0006468	11	3039	0.00625842	protein phosphorylation
GO:0071356	5	301	0.00627635	cellular response to tumor necrosis factor
GO:0034138	4	128	0.00636642	toll-like receptor 3 signaling pathway
GO:0032675	5	302	0.00637912	regulation of interleukin-6 production
GO:2000736	5	302	0.00637912	regulation of stem cell differentiation
GO:0046907	12	3768	0.00639922	intracellular transport
GO:0030326	5	303	0.00648321	embryonic limb morphogenesis
GO:0035113	5	303	0.00648321	embryonic appendage morphogenesis
GO:0006308	5	304	0.00658866	DNA catabolic process
GO:0051262	5	304	0.00658866	protein tetramerization
GO:0042509	4	130	0.00677254	regulation of tyrosine phosphorylation of STAT protein
GO:0032872	6	568	0.00690572	regulation of stress-activated MAPK cascade
GO:0001654	5	307	0.00691308	eye development
GO:0046887	5	308	0.00702398	positive regulation of hormone secretion
GO:1903322	5	310	0.00724998	positive regulation of protein modification by small protein conjugation or removal
GO:0019725	9	1855	0.00739825	cellular homeostasis
GO:0033158	3	36	0.00739971	regulation of protein import into nucleus, translocation
GO:0021675	4	133	0.00741736	nerve development
GO:0032104	4	133	0.00741736	regulation of response to extracellular stimulus
GO:0032107	4	133	0.00741736	regulation of response to nutrient levels
GO:0031329	11	3094	0.00747731	regulation of cellular catabolic process
GO:0044723	9	1859	0.00753147	single-organism carbohydrate metabolic process
GO:0034330	6	577	0.00756182	cell junction organization
GO:2000179	4	134	0.00764208	positive regulation of neural precursor cell proliferation
GO:2000241	5	316	0.00796262	regulation of reproductive process
GO:0010893	3	37	0.008049	positive regulation of steroid biosynthetic process
GO:2000779	3	37	0.008049	regulation of double-strand break repair
GO:2000107	4	136	0.00810668	negative regulation of leukocyte apoptotic process
GO:0007596	8	1367	0.00813882	blood coagulation
GO:0050817	8	1367	0.00813882	coagulation
GO:0006644	7	938	0.00829528	phospholipid metabolic process
GO:0016125	5	319	0.00833902	sterol metabolic process
GO:0035137	4	137	0.00834667	hindlimb morphogenesis
GO:0046632	4	137	0.00834667	alpha-beta T cell differentiation
GO:0048661	4	137	0.00834667	positive regulation of smooth muscle cell proliferation
GO:0060560	5	320	0.00846756	developmental growth involved in morphogenesis
GO:0051051	8	1375	0.00850068	negative regulation of transport
GO:0071229	6	589	0.00851514	cellular response to acid chemical
GO:0044772	6	590	0.00859884	mitotic cell cycle phase transition
GO:0007599	8	1382	0.00882847	hemostasis

Table 12: Overrepresented terms with the network-based enrichment. Only terms not detected with the standard method.

GO Term	N1	N2	P-value	Description
GO:0010608	8	1385	0.00897227	posttranscriptional regulation of gene expression
GO:0044770	6	595	0.00902743	cell cycle phase transition
GO:0000186	4	140	0.00909836	activation of MAPKK activity
GO:0002763	4	140	0.00909836	positive regulation of myeloid leukocyte differentiation
GO:0045637	6	596	0.00911519	regulation of myeloid cell differentiation
GO:0072091	5	327	0.00941162	regulation of stem cell proliferation
GO:0032026	3	39	0.00945865	response to magnesium ion
GO:0070243	3	39	0.00945865	regulation of thymocyte apoptotic process
GO:0007167	10	2504	0.00950806	enzyme linked receptor protein signaling pathway
GO:0007049	8	1396	0.00951666	cell cycle
GO:0001819	7	963	0.00987183	positive regulation of cytokine production
GO:0051099	5	331	0.00998719	positive regulation of binding
GO:0030334	9	1926	0.0100938	regulation of cell migration
GO:0002768	7	967	0.010146	immune response-regulating cell surface receptor signaling pathway
GO:0033598	3	40	0.010221	mammary gland epithelial cell proliferation
GO:0060765	3	40	0.010221	regulation of androgen receptor signaling pathway
GO:0032869	6	609	0.0103212	cellular response to insulin stimulus
GO:0070613	6	609	0.0103212	regulation of protein processing
GO:1903317	6	609	0.0103212	regulation of protein maturation
GO:0051347	9	1933	0.0104007	positive regulation of transferase activity
GO:2000045	5	334	0.0104367	regulation of G1/S transition of mitotic cell cycle
GO:0022411	7	972	0.0104976	cellular component disassembly
GO:0051603	7	974	0.0106411	proteolysis involved in cellular protein catabolic process
GO:1902806	5	336	0.0107452	regulation of cell cycle G1/S phase transition
GO:0002755	4	146	0.010751	MyD88-dependent toll-like receptor signaling pathway
GO:0044030	3	41	0.0110229	regulation of DNA methylation
GO:0001501	6	620	0.0114408	skeletal system development
GO:0006066	7	986	0.011537	alcohol metabolic process
GO:0001666	7	987	0.0116145	response to hypoxia
GO:0030518	4	149	0.0116561	intracellular steroid hormone receptor signaling pathway
GO:0048015	5	342	0.0117138	phosphatidylinositol-mediated signaling
GO:0048017	5	342	0.0117138	inositol lipid-mediated signaling
GO:0071901	5	342	0.0117138	negative regulation of protein serine/threonine kinase activity
GO:0090200	3	42	0.0118655	positive regulation of release of cytochrome c from mitochondria
GO:0014068	4	150	0.01197	positive regulation of phosphatidylinositol 3-kinase signaling
GO:0071230	4	150	0.01197	cellular response to amino acid stimulus
GO:0002694	8	1443	0.0121727	regulation of leukocyte activation
GO:0022412	7	998	0.0124953	cellular process involved in reproduction in multicellular organism
GO:0036293	7	999	0.0125781	response to decreased oxygen levels
GO:1990138	4	152	0.0126165	neuron projection extension
GO:0051606	8	1452	0.0127475	detection of stimulus
GO:0097194	3	43	0.0127496	execution phase of apoptosis
GO:0035107	5	348	0.0127499	appendage morphogenesis
GO:0035108	5	348	0.0127499	limb morphogenesis
GO:0016458	4	153	0.0129493	gene silencing
GO:0060429	7	1006	0.0131705	epithelium development
GO:0000018	4	154	0.0132885	regulation of DNA recombination
GO:0033993	10	2604	0.013553	response to lipid
GO:0007399	7	1018	0.0142404	nervous system development
GO:0046889	4	157	0.0143458	positive regulation of lipid biosynthetic process
GO:0050678	7	1020	0.0144256	regulation of epithelial cell proliferation
GO:0060707	3	45	0.0146467	trophoblast giant cell differentiation
GO:0007033	4	158	0.0147117	vacuole organization
GO:0050778	8	1484	0.0149831	positive regulation of immune response
GO:0007566	4	160	0.0154642	embryo implantation
GO:0030098	6	654	0.015545	lymphocyte differentiation
GO:0034394	3	46	0.0156615	protein localization to cell surface
GO:0007498	4	161	0.0158509	mesoderm development
GO:0009611	6	657	0.0159583	response to wounding
GO:0009612	6	658	0.0160981	response to mechanical stimulus

Table 13: Overrepresented terms with the network-based enrichment. Only terms not detected with the standard method.

GO Term	N1	N2	P-value	Description
GO:2000145	9	2039	0.0161399	regulation of cell motility
GO:1903035	5	366	0.0162966	negative regulation of response to wounding
GO:0030336	6	660	0.0163805	negative regulation of cell migration
GO:0006605	6	661	0.0165233	protein targeting
GO:0035019	4	163	0.0166459	somatic stem cell maintenance
GO:0051106	2	5	0.0166654	positive regulation of DNA ligation
GO:1901563	2	5	0.0166654	response to camptothecin
GO:0045580	5	369	0.0169563	regulation of T cell differentiation
GO:0035295	6	664	0.0169576	tube development
GO:0002064	5	370	0.0171808	epithelial cell development
GO:2001234	6	666	0.0172523	negative regulation of apoptotic signaling pathway
GO:0051149	4	165	0.0174699	positive regulation of muscle cell differentiation
GO:0048545	8	1516	0.0175447	response to steroid hormone
GO:0006022	5	372	0.0176368	aminoglycan metabolic process
GO:0045444	5	372	0.0176368	fat cell differentiation
GO:0035066	3	48	0.0178285	positive regulation of histone acetylation
GO:0032355	5	374	0.0181023	response to estradiol
GO:0046635	4	167	0.0183238	positive regulation of alpha-beta T cell activation
GO:0032940	8	1530	0.0187777	secretion by cell
GO:0008347	3	49	0.0189827	glial cell migration
GO:0051591	5	378	0.0190622	response to cAMP
GO:0048844	4	170	0.0196621	artery morphogenesis
GO:0043687	5	383	0.0203178	post-translational protein modification
GO:0070482	7	1076	0.0204951	response to oxygen levels
GO:0034142	4	172	0.0205936	toll-like receptor 4 signaling pathway
GO:2000146	6	688	0.0207787	negative regulation of cell motility
GO:0061351	4	173	0.0210714	neural precursor cell proliferation
GO:0030217	5	386	0.0211017	T cell differentiation
GO:0031647	5	386	0.0211017	regulation of protein stability
GO:0000278	6	691	0.021302	mitotic cell cycle
GO:0051607	5	387	0.0213683	defense response to virus
GO:0010837	3	51	0.0214372	regulation of keratinocyte proliferation
GO:0097305	7	1084	0.0215149	response to alcohol
GO:0050865	8	1568	0.0225028	regulation of cell activation
GO:0032677	4	176	0.0225541	regulation of interleukin-8 production
GO:0010952	5	393	0.0230234	positive regulation of peptidase activity
GO:0060688	4	177	0.023065	regulation of morphogenesis of a branching structure
GO:0071383	5	394	0.0233088	cellular response to steroid hormone stimulus
GO:0007548	3	53	0.0240929	sex differentiation
GO:0008585	3	53	0.0240929	female gonad development
GO:0060441	3	53	0.0240929	epithelial tube branching involved in lung morphogenesis
GO:0090342	3	53	0.0240929	regulation of cell aging
GO:0016310	12	4277	0.0246725	phosphorylation
GO:0002028	4	181	0.0251948	regulation of sodium ion transport
GO:0032024	4	182	0.0257492	positive regulation of insulin secretion
GO:0007160	5	403	0.0260042	cell-matrix adhesion
GO:0046823	4	183	0.0263125	negative regulation of nucleocytoplasmic transport
GO:0009308	5	405	0.0266351	amine metabolic process
GO:0008284	10	2808	0.0267	positive regulation of cell proliferation
GO:0034122	3	55	0.0269577	negative regulation of toll-like receptor signaling pathway
GO:0009880	4	185	0.0274666	embryonic pattern specification
GO:0048565	4	185	0.0274666	digestive tract development
GO:0051270	9	2182	0.0280962	regulation of cellular component movement
GO:0019827	5	410	0.0282648	stem cell maintenance
GO:0034976	5	410	0.0282648	response to endoplasmic reticulum stress
GO:0046578	5	410	0.0282648	regulation of Ras protein signal transduction
GO:0007267	9	2185	0.0284125	cell-cell signaling
GO:0046849	3	56	0.0284707	bone remodeling
GO:0017157	5	411	0.0286	regulation of exocytosis
GO:0031334	5	411	0.0286	positive regulation of protein complex assembly

Table 14: Overrepresented terms with the network-based enrichment. Only terms not detected with the standard method.

GO Term	N1	N2	P-value	Description
GO:0071260	4	187	0.0286576	cellular response to mechanical stimulus
GO:2001242	5	412	0.0289382	regulation of intrinsic apoptotic signaling pathway
GO:0030858	4	188	0.0292672	positive regulation of epithelial cell differentiation
GO:0055114	11	3568	0.0303081	oxidation-reduction process
GO:2001239	4	190	0.0305151	regulation of extrinsic apoptotic signaling pathway in absence of ligand
GO:0045860	8	1636	0.0307478	positive regulation of protein kinase activity
GO:0090305	6	737	0.0307682	nucleic acid phosphodiester bond hydrolysis
GO:0009416	7	1146	0.0309538	response to light stimulus
GO:0071345	8	1640	0.0313037	cellular response to cytokine stimulus
GO:0010632	5	419	0.0313947	regulation of epithelial cell migration
GO:0010719	3	58	0.0316631	negative regulation of epithelial to mesenchymal transition
GO:0045940	3	58	0.0316631	positive regulation of steroid metabolic process
GO:0032388	6	743	0.0322217	positive regulation of intracellular transport
GO:0007423	5	423	0.0328701	sensory organ development
GO:1902107	5	423	0.0328701	positive regulation of leukocyte differentiation
GO:0006629	11	3598	0.0328768	lipid metabolic process
GO:0045596	9	2226	0.0330529	negative regulation of cell differentiation
GO:0048010	3	59	0.0333444	vascular endothelial growth factor receptor signaling pathway
GO:0042594	5	427	0.0343992	response to starvation
GO:0090002	4	196	0.0344953	establishment of protein localization to plasma membrane
GO:0090263	4	197	0.0351941	positive regulation of canonical Wnt signaling pathway
GO:0055085	11	3627	0.0355402	transmembrane transport
GO:0050810	4	198	0.0359034	regulation of steroid biosynthetic process
GO:0002706	5	431	0.0359835	regulation of lymphocyte mediated immunity
GO:0007600	8	1675	0.0365433	sensory perception
GO:0071887	3	61	0.0368815	leukocyte apoptotic process
GO:1901985	3	61	0.0368815	positive regulation of protein acetylation
GO:2000758	3	61	0.0368815	positive regulation of peptidyl-lysine acetylation
GO:0042692	5	437	0.0384666	muscle cell differentiation
GO:0001838	3	62	0.0387396	embryonic epithelial tube formation
GO:0007051	4	202	0.0388461	spindle organization
GO:0008217	5	438	0.0388934	regulation of blood pressure
GO:0034103	4	203	0.0396089	regulation of tissue remodeling
GO:1902807	4	204	0.0403826	negative regulation of cell cycle G1/S phase transition
GO:2000134	4	204	0.0403826	negative regulation of G1/S transition of mitotic cell cycle
GO:0072175	3	63	0.0406582	epithelial tube formation
GO:0033674	8	1706	0.0417888	positive regulation of kinase activity
GO:0007059	4	206	0.0419637	chromosome segregation
GO:0000045	3	64	0.0426386	autophagic vacuole assembly
GO:0030879	3	64	0.0426386	mammary gland development
GO:0001938	4	207	0.0427708	positive regulation of endothelial cell proliferation
GO:0042472	4	207	0.0427708	inner ear morphogenesis
GO:0010639	6	783	0.0434047	negative regulation of organelle organization
GO:0035567	3	65	0.0446816	non-canonical Wnt signaling pathway
GO:0060749	3	65	0.0446816	mammary gland alveolus development
GO:0014066	4	210	0.0452618	regulation of phosphatidylinositol 3-kinase signaling
GO:0030500	4	210	0.0452618	regulation of bone mineralization
GO:0034613	7	1216	0.0455341	cellular protein localization
GO:0030155	7	1217	0.0457778	regulation of cell adhesion
GO:0031667	7	1219	0.0462683	response to nutrient levels
GO:0007131	3	66	0.0467883	reciprocal meiotic recombination
GO:0035825	3	66	0.0467883	reciprocal DNA recombination
GO:0046825	3	66	0.0467883	regulation of protein export from nucleus
GO:0006184	6	794	0.0469772	GTP catabolic process
GO:0010634	4	212	0.0469805	positive regulation of epithelial cell migration
GO:0060041	4	212	0.0469805	retina development in camera-type eye
GO:0032088	4	213	0.0478578	negative regulation of NF-kappaB transcription factor activity
GO:0000902	6	800	0.0490242	cell morphogenesis
GO:0050714	5	460	0.0492541	positive regulation of protein secretion

Table 15: Overrepresented terms with the network-based enrichment. Only terms not detected with the standard method.
