# Supplementary material for: NET-GE: a novel NETwork-based Gene Enrichment for detecting biological processes associated to Mendelian diseases
Source: BMC Genomics. 2015 Jun 18;16(Suppl 8):S6. doi: 10.1186/1471-2164-16-S8-S6 (PMC4480278; doi:10.1186/1471-2164-16-S8-S6)
Supplement: Additional file 3 — Detailed results for the OMIM-derived benchmark set. The archive contains pdf documents listing the enriched terms for each one of the 244 diseases in the OMIM-derived benchmark set. [file 1471-2164-16-S8-S6-S3.tgz › SUPPMAT/OMIM114500.pdf]

#114500 COLORECTAL CANCER; CRC

OMIM Gene ID	HGNC	UniProtAC
116806	CTNNB1	P35222
120470	DCC	P43146
134934	FGFR3	P22607
159350	MCC	P23508
164730	AKT1	P31749
164790	NRAS	P01111
165640	ODC1	P11926
168461	CCND1	P24385
171834	PIK3CA	P42336
172411	PLA2G2A	P14555
191170	TP53	P04637
600040	BAX	Q07812
600079	PTPN12	Q05209
600925	PTPRJ	Q12913
602700	EP300	Q09472
602860	BUB1B	O60566
603028	TLR2	O60603
603030	TLR4	O00206
603072	AURKA	O14965
604025	AXIN2	Q9Y2T1
604289	RAD54B	Q9Y620
604395	MLH3	Q9UHC1
604584	PDGFRL	Q15198
607273	FLCN	Q8NFG4
611731	APC	P25054

Table 1: OMIM - UniProtAC mapping

Legend

- N1: #input proteins associated to the significant GO term
- N2: #proteins associated to the significant GO term
- P-value: Bonferroni-corrected p-value of Fisher's exact test
- *red*: go terms not related to the input proteins
- *blue*: go terms related to the input proteins (enriched uniquely by network-based method)
- *green*: go terms ancestors of terms enriched with the standard method (enriched uniquely by network-based method)

1 Standard enrichment

GO Term	N1	N2	P-value	Description
GO:0051246	18	2954	6.92309e-12	regulation of protein metabolic process
GO:0042127	16	2008	1.06886e-11	regulation of cell proliferation
GO:0032268	16	2272	7.29671e-11	regulation of cellular protein metabolic process
GO:0031399	15	1823	7.54819e-11	regulation of protein modification process
GO:0048523	20	5279	4.41192e-10	negative regulation of cellular process
GO:0045595	15	2111	6.37075e-10	regulation of cell differentiation
GO:0042325	14	1770	1.37802e-09	regulation of phosphorylation
GO:0051239	17	3432	2.15693e-09	regulation of multicellular organismal process
GO:0048519	20	5756	2.32806e-09	negative regulation of biological process
GO:0051247	13	1526	5.15225e-09	positive regulation of protein metabolic process
GO:0051726	12	1232	1.03608e-08	regulation of cell cycle
GO:0048583	18	4515	1.07979e-08	regulation of response to stimulus
GO:0008285	11	920	1.18039e-08	negative regulation of cell proliferation
GO:0010564	10	662	1.41238e-08	regulation of cell cycle process
GO:0009966	16	3261	1.89612e-08	regulation of signal transduction
GO:0009968	12	1361	3.29783e-08	negative regulation of signal transduction
GO:0048522	19	5768	4.54335e-08	positive regulation of cellular process
GO:0023057	12	1420	5.39336e-08	negative regulation of signaling
GO:0010648	12	1424	5.57193e-08	negative regulation of cell communication
GO:0050793	15	2884	5.67436e-08	regulation of developmental process
GO:0001932	12	1440	6.34126e-08	regulation of protein phosphorylation
GO:0009893	16	3630	9.66388e-08	positive regulation of metabolic process
GO:0042327	11	1129	1.055e-07	positive regulation of phosphorylation
GO:0007166	17	4368	1.06681e-07	cell surface receptor signaling pathway
GO:0050678	8	361	1.29354e-07	regulation of epithelial cell proliferation
GO:0023051	16	3708	1.33303e-07	regulation of signaling
GO:0045596	10	834	1.3544e-07	negative regulation of cell differentiation
GO:0010646	16	3714	1.36602e-07	regulation of cell communication
GO:0010942	9	579	1.54401e-07	positive regulation of cell death
GO:0048585	12	1639	2.82312e-07	negative regulation of response to stimulus
GO:0010562	11	1255	3.24885e-07	positive regulation of phosphorus metabolic process
GO:0045937	11	1255	3.24885e-07	positive regulation of phosphate metabolic process
GO:0010604	15	3285	3.60789e-07	positive regulation of macromolecule metabolic process
GO:0048518	19	6624	5.42638e-07	positive regulation of biological process
GO:2000026	13	2229	5.80779e-07	regulation of multicellular organismal development
GO:0031325	15	3418	6.32047e-07	positive regulation of cellular metabolic process
GO:0044767	19	6740	7.39327e-07	single-organism developmental process
GO:1902531	12	1847	1.11035e-06	regulation of intracellular signal transduction
GO:0051093	10	1051	1.27713e-06	negative regulation of developmental process
GO:0019220	14	2977	1.44068e-06	regulation of phosphate metabolic process
GO:0051174	14	2996	1.56646e-06	regulation of phosphorus metabolic process
GO:0033043	10	1117	2.29744e-06	regulation of organelle organization
GO:0043408	9	794	2.47841e-06	regulation of MAPK cascade
GO:0006915	9	805	2.7949e-06	apoptotic process
GO:0043065	8	536	2.92061e-06	positive regulation of apoptotic process
GO:0032502	19	7299	3.0392e-06	developmental process
GO:0043068	8	543	3.23309e-06	positive regulation of programmed cell death
GO:0010941	12	2079	4.26899e-06	regulation of cell death
GO:0065009	15	3941	4.6579e-06	regulation of molecular function
GO:0012501	9	868	5.39115e-06	programmed cell death
GO:0006950	15	4134	9.0605e-06	response to stress
GO:0008283	9	940	1.07743e-05	cell proliferation
GO:0002682	11	1758	1.1233e-05	regulation of immune system process
GO:0050896	22	11721	1.12754e-05	response to stimulus
GO:0051094	10	1326	1.18918e-05	positive regulation of developmental process
GO:0001934	9	956	1.2472e-05	positive regulation of protein phosphorylation
GO:0048584	12	2308	1.38996e-05	positive regulation of response to stimulus
GO:0032270	10	1363	1.54596e-05	positive regulation of cellular protein metabolic process
GO:0044707	15	4361	1.89983e-05	single-multicellular organism process
GO:1902533	9	1008	1.9725e-05	positive regulation of intracellular signal transduction

Table 2: Overrepresented GO terms with the standard enrichment

GO Term	N1	N2	P-value	Description
GO:0032501	15	4447	2.48753e-05	multicellular organismal process
GO:0051716	20	9450	2.68393e-05	cellular response to stimulus
GO:0051130	9	1059	3.02034e-05	positive regulation of cellular component organization
GO:0031324	12	2479	3.1002e-05	negative regulation of cellular metabolic process
GO:0042981	11	1970	3.65797e-05	regulation of apoptotic process
GO:0043067	11	1982	3.8947e-05	regulation of programmed cell death
GO:0051128	12	2549	4.23233e-05	regulation of cellular component organization
GO:0008219	9	1106	4.38993e-05	cell death
GO:0065008	14	3888	4.65458e-05	regulation of biological quality
GO:0016265	9	1117	4.77986e-05	death
GO:0009967	10	1548	5.17271e-05	positive regulation of signal transduction
GO:0007165	18	7592	6.69618e-05	signal transduction
GO:0060284	9	1166	6.91041e-05	regulation of cell development
GO:0009892	12	2679	7.36691e-05	negative regulation of metabolic process
GO:0032880	8	819	7.8791e-05	regulation of protein localization
GO:0040008	8	822	8.1041e-05	regulation of growth
GO:0001775	8	825	8.33462e-05	cell activation
GO:0045087	8	825	8.33462e-05	innate immune response
GO:0023056	10	1630	8.41796e-05	positive regulation of signaling
GO:0045926	6	303	8.43661e-05	negative regulation of growth
GO:0051338	9	1198	8.71479e-05	regulation of transferase activity
GO:0010647	10	1637	8.76458e-05	positive regulation of cell communication
GO:0031401	9	1213	9.69409e-05	positive regulation of protein modification process
GO:0060255	19	8942	0.000105918	regulation of macromolecule metabolic process
GO:0030111	6	325	0.000127686	regulation of Wnt signaling pathway
GO:0010639	6	327	0.000132396	negative regulation of organelle organization
GO:0010033	13	3487	0.000132814	response to organic substance
GO:0048856	14	4289	0.000163399	anatomical structure development
GO:0070887	12	2904	0.00017999	cellular response to chemical stimulus
GO:1901214	6	348	0.000191128	regulation of neuron death
GO:0010948	6	358	0.000225836	negative regulation of cell cycle process
GO:0002764	7	615	0.000233492	immune response-regulating signaling pathway
GO:0060341	9	1363	0.000261898	regulation of cellular localization
GO:0002376	11	2446	0.000335453	immune system process
GO:0010605	11	2452	0.000343893	negative regulation of macromolecule metabolic process
GO:0048513	10	1910	0.000371353	organ development
GO:0043409	5	196	0.000372486	negative regulation of MAPK cascade
GO:0071310	11	2482	0.000389005	cellular response to organic substance
GO:0031323	19	9728	0.000450601	regulation of cellular metabolic process
GO:0009628	9	1467	0.000488223	response to abiotic stimulus
GO:0080090	19	9808	0.000518223	regulation of primary metabolic process
GO:0051129	7	694	0.000528302	negative regulation of cellular component organization
GO:0042221	14	4712	0.000537162	response to chemical
GO:0009719	10	2012	0.000601945	response to endogenous stimulus
GO:0043549	8	1079	0.000648549	regulation of kinase activity
GO:0010243	8	1094	0.000720117	response to organonitrogen compound
GO:0032481	4	92	0.000854712	positive regulation of type I interferon production
GO:0006952	9	1580	0.000912245	defense response
GO:0050790	12	3371	0.000921262	regulation of catalytic activity
GO:1902589	10	2159	0.00115436	single-organism organelle organization
GO:0042326	6	475	0.00118337	negative regulation of phosphorylation
GO:1901698	8	1186	0.00132575	response to nitrogen compound
GO:0002768	6	486	0.00135211	immune response-regulating cell surface receptor signaling pathway
GO:0006796	14	5088	0.0014039	phosphate-containing compound metabolic process
GO:0032879	11	2827	0.00144149	regulation of localization
GO:0010821	4	107	0.00156808	regulation of mitochondrion organization
GO:0006793	14	5157	0.00165989	phosphorus metabolic process
GO:0006996	11	2908	0.00191092	organelle organization
GO:0007346	6	518	0.0019587	regulation of mitotic cell cycle
GO:0010563	6	529	0.00221263	negative regulation of phosphorus metabolic process

Table 3: Overrepresented GO terms with the standard enrichment

GO Term	N1	N2	P-value	Description
GO:0045936	6	529	0.00221263	negative regulation of phosphate metabolic process
GO:0009725	8	1273	0.00225751	response to hormone
GO:0032386	6	537	0.00241375	regulation of intracellular transport
GO:0070602	2	3	0.00268533	regulation of centromeric sister chromatid cohesion
GO:0019222	19	10848	0.00285265	regulation of metabolic process
GO:0043523	5	300	0.00304239	regulation of neuron apoptotic process
GO:0031400	6	561	0.00310896	negative regulation of protein modification process
GO:0071214	5	302	0.00314308	cellular response to abiotic stimulus
GO:0030154	10	2446	0.00361511	cell differentiation
GO:0009798	4	132	0.00363076	axis specification
GO:0007389	6	579	0.00373141	pattern specification process
GO:0045321	6	579	0.00373141	leukocyte activation
GO:0051302	5	316	0.00392381	regulation of cell division
GO:0032479	4	135	0.00397103	regulation of type I interferon production
GO:0044772	5	318	0.00404678	mitotic cell cycle phase transition
GO:0071417	6	588	0.00407884	cellular response to organonitrogen compound
GO:0044093	10	2479	0.0040829	positive regulation of molecular function
GO:0044770	5	321	0.00423693	cell cycle phase transition
GO:0007088	4	139	0.00446086	regulation of mitosis
GO:0045429	3	38	0.00453376	positive regulation of nitric oxide biosynthetic process
GO:0008104	6	604	0.00476175	protein localization
GO:0022402	8	1409	0.00481907	cell cycle process
GO:0016043	15	6578	0.00484037	cellular component organization
GO:0006955	8	1414	0.00494775	immune response
GO:0071840	15	6631	0.00537565	cellular component organization or biogenesis
GO:0009888	7	984	0.00543142	tissue development
GO:0033036	6	618	0.00543344	macromolecule localization
GO:0045597	7	985	0.00546797	positive regulation of cell differentiation
GO:1901028	3	41	0.00572149	regulation of mitochondrial outer membrane permeabilization involved in apoptotic signaling pathway
GO:0007093	4	148	0.00572564	mitotic cell cycle checkpoint
GO:0045859	7	1005	0.00624359	regulation of protein kinase activity
GO:0009605	9	1995	0.00634507	response to external stimulus
GO:1901699	6	645	0.00694768	cellular response to nitrogen compound
GO:0016055	5	361	0.00751407	Wnt signaling pathway
GO:0050680	4	159	0.00761157	negative regulation of epithelial cell proliferation
GO:0033674	6	656	0.00765587	positive regulation of kinase activity
GO:0032868	5	376	0.00915955	response to insulin
GO:0003002	5	380	0.00964283	regionalization
GO:0051983	3	49	0.0098541	regulation of chromosome segregation
GO:1901701	7	1086	0.0103949	cellular response to oxygen-containing compound
GO:0090199	3	50	0.0104785	regulation of release of cytochrome c from mitochondria
GO:0007167	7	1091	0.0107127	enzyme linked receptor protein signaling pathway
GO:0009653	9	2131	0.0108944	anatomical structure morphogenesis
GO:0008284	7	1101	0.0113728	positive regulation of cell proliferation
GO:0009411	4	177	0.0116409	response to UV
GO:0045839	3	53	0.0125075	negative regulation of mitosis
GO:0044763	22	16559	0.0128022	single-organism cellular process
GO:0070141	2	6	0.0134103	response to UV-A
GO:0006974	7	1132	0.013639	cellular response to DNA damage stimulus
GO:0050768	4	185	0.0138637	negative regulation of neurogenesis
GO:0051783	4	186	0.0141621	regulation of nuclear division
GO:0030334	6	733	0.0144434	regulation of cell migration
GO:0050679	4	188	0.014773	positive regulation of epithelial cell proliferation
GO:0045428	3	56	0.0147808	regulation of nitric oxide biosynthetic process
GO:0060548	7	1147	0.014864	negative regulation of cell death
GO:0032269	6	737	0.0148985	negative regulation of cellular protein metabolic process
GO:0030178	4	193	0.0163856	negative regulation of Wnt signaling pathway
GO:0009416	5	424	0.0163998	response to light stimulus
GO:0072332	3	58	0.0164386	intrinsic apoptotic signaling pathway by p53 class mediator
GO:0051347	6	751	0.0165841	positive regulation of transferase activity

Table 4: Overrepresented GO terms with the standard enrichment

GO Term	N1	N2	P-value	Description
GO:0050776	7	1167	0.0166391	regulation of immune response
GO:0008286	4	195	0.0170658	insulin receptor signaling pathway
GO:0033044	4	197	0.0177665	regulation of chromosome organization
GO:0071363	6	762	0.0180146	cellular response to growth factor stimulus
GO:0033077	3	60	0.0182148	T cell differentiation in thymus
GO:0035019	3	60	0.0182148	somatic stem cell maintenance
GO:0030308	4	199	0.0184883	negative regulation of cell growth
GO:0097190	5	439	0.0193996	apoptotic signaling pathway
GO:0007173	4	202	0.0196113	epidermal growth factor receptor signaling pathway
GO:0048869	11	3694	0.0199927	cellular developmental process
GO:0038127	4	205	0.0207839	ERBB signaling pathway
GO:2000145	6	786	0.0214856	regulation of cell motility
GO:0070848	6	793	0.0225941	response to growth factor
GO:0010638	5	454	0.0228133	positive regulation of organelle organization
GO:0001558	5	455	0.0230567	regulation of cell growth
GO:0008543	4	211	0.0232833	fibroblast growth factor receptor signaling pathway
GO:0007169	6	798	0.0234141	transmembrane receptor protein tyrosine kinase signaling pathway
GO:0010822	3	66	0.0242936	positive regulation of mitochondrion organization
GO:0034613	5	465	0.0256026	cellular protein localization
GO:0043525	3	68	0.0265829	positive regulation of neuron apoptotic process
GO:0006357	9	2379	0.0266224	regulation of transcription from RNA polymerase II promoter
GO:0070727	5	473	0.0277934	cellular macromolecule localization
GO:0060828	4	225	0.0299719	regulation of canonical Wnt signaling pathway
GO:0040011	7	1284	0.0309499	locomotion
GO:0044092	7	1284	0.0309499	negative regulation of molecular function
GO:0006468	8	1815	0.0310353	protein phosphorylation
GO:0046649	5	484	0.0310422	lymphocyte activation
GO:0051270	6	843	0.03194	regulation of cellular component movement
GO:0071495	7	1291	0.0320575	cellular response to endogenous stimulus
GO:0007091	2	9	0.0321455	metaphase/anaphase transition of mitotic cell cycle
GO:0044784	2	9	0.0321455	metaphase/anaphase transition of cell cycle
GO:0070372	4	231	0.0332334	regulation of ERK1 and ERK2 cascade
GO:0010721	4	232	0.0338015	negative regulation of cell development
GO:0080134	7	1307	0.0347135	regulation of response to stress
GO:0045944	7	1312	0.0355801	positive regulation of transcription from RNA polymerase II promoter
GO:1901700	8	1851	0.0357889	response to oxygen-containing compound
GO:0050794	22	17465	0.0366633	regulation of cellular process
GO:0040012	6	864	0.0366992	regulation of locomotion
GO:0044344	4	237	0.0367492	cellular response to fibroblast growth factor stimulus
GO:1902532	5	502	0.0369911	negative regulation of intracellular signal transduction
GO:0051240	6	866	0.0371808	positive regulation of multicellular organismal process
GO:0051784	3	77	0.0386486	negative regulation of nuclear division
GO:0000075	4	241	0.0392404	cell cycle checkpoint
GO:0051248	6	875	0.0394122	negative regulation of protein metabolic process
GO:1900180	4	242	0.0398821	regulation of protein localization to nucleus
GO:0044336	2	10	0.0401657	canonical Wnt signaling pathway involved in negative regulation of apoptotic process
GO:0071774	4	243	0.0405316	response to fibroblast growth factor
GO:1901215	4	244	0.0411887	negative regulation of neuron death
GO:1903047	6	886	0.0422853	mitotic cell cycle process
GO:0097193	4	246	0.0425264	intrinsic apoptotic signaling pathway
GO:0035556	9	2537	0.0446143	intracellular signal transduction
GO:0016310	9	2550	0.0464744	phosphorylation
GO:1901991	4	252	0.046732	negative regulation of mitotic cell cycle phase transition
GO:0033554	8	1930	0.0484162	cellular response to stress

Table 5: Overrepresented GO terms with the standard enrichment

2 Network-based enrichment

GO Term	N1	N2	P-value	Description
GO:0048011	14	692	3.56505e-14	neurotrophin TRK receptor signaling pathway
GO:0038179	14	720	6.18964e-14	neurotrophin signaling pathway
GO:0045785	13	632	6.2197e-13	positive regulation of cell adhesion
GO:0071900	16	1492	1.36378e-12	regulation of protein serine/threonine kinase activity
GO:0030162	16	1768	1.93973e-11	regulation of proteolysis
GO:0040017	14	1124	2.91223e-11	positive regulation of locomotion
GO:0030177	10	316	5.95286e-11	positive regulation of Wnt signaling pathway
GO:0030155	14	1217	8.65808e-11	regulation of cell adhesion
GO:0043434	14	1303	2.20203e-10	response to peptide hormone
GO:0030335	13	1024	3.02601e-10	positive regulation of cell migration
GO:2000147	13	1042	3.77693e-10	positive regulation of cell motility
GO:1901652	14	1373	4.49599e-10	response to peptide
GO:0051272	13	1060	4.69573e-10	positive regulation of cellular component movement
GO:0032101	16	2173	4.77774e-10	regulation of response to external stimulus
GO:0080135	14	1388	5.21338e-10	regulation of cellular response to stress
GO:0042176	12	816	6.75189e-10	regulation of protein catabolic process
GO:0019827	10	410	8.00328e-10	stem cell maintenance
GO:0014070	17	2783	1.02136e-09	response to organic cyclic compound
GO:0050767	16	2377	1.90886e-09	regulation of neurogenesis
GO:0051345	17	2953	2.69136e-09	positive regulation of hydrolase activity
GO:0046822	11	675	3.03277e-09	regulation of nucleocytoplasmic transport
GO:1903322	9	310	3.37275e-09	positive regulation of protein modification by small protein conjugation or removal
GO:0002683	12	945	3.7889e-09	negative regulation of immune system process
GO:0051050	16	2507	4.33044e-09	positive regulation of transport
GO:0043410	13	1269	4.58887e-09	positive regulation of MAPK cascade
GO:0045860	14	1636	4.85717e-09	positive regulation of protein kinase activity
GO:0060561	6	49	5.16154e-09	apoptotic process involved in morphogenesis
GO:0051046	15	2094	6.51163e-09	regulation of secretion
GO:0051960	16	2584	6.89006e-09	regulation of nervous system development
GO:0048731	16	2612	8.12832e-09	system development
GO:1903320	10	520	8.43156e-09	regulation of protein modification by small protein conjugation or removal
GO:0043086	16	2637	9.40605e-09	negative regulation of catalytic activity
GO:0042306	10	527	9.6211e-09	regulation of protein import into nucleus
GO:0009896	11	786	1.56774e-08	positive regulation of catabolic process
GO:0070482	12	1076	1.72885e-08	response to oxygen levels
GO:1902742	6	62	2.25468e-08	apoptotic process involved in development
GO:0002694	13	1443	2.31311e-08	regulation of leukocyte activation
GO:0070201	14	1849	2.5318e-08	regulation of establishment of protein localization
GO:0045786	11	824	2.60465e-08	negative regulation of cell cycle
GO:0051098	11	826	2.67337e-08	regulation of binding
GO:0009894	17	3408	2.76389e-08	regulation of catabolic process
GO:0022603	16	2832	2.80043e-08	regulation of anatomical structure morphogenesis
GO:0032147	11	832	2.88949e-08	activation of protein kinase activity
GO:0032989	14	1879	3.14344e-08	cellular component morphogenesis
GO:0006935	11	841	3.24334e-08	chemotaxis
GO:0042330	11	841	3.24334e-08	taxis
GO:0050727	11	853	3.77596e-08	regulation of inflammatory response
GO:0051241	13	1513	4.1894e-08	negative regulation of multicellular organismal process
GO:0031214	8	257	4.38696e-08	biomineral tissue development
GO:0033157	11	878	5.147e-08	regulation of intracellular protein transport
GO:0000187	9	421	5.2091e-08	activation of MAPK activity
GO:0031346	11	897	6.47386e-08	positive regulation of cell projection organization
GO:0050865	13	1568	6.54949e-08	regulation of cell activation
GO:0043433	9	432	6.55312e-08	negative regulation of sequence-specific DNA binding transcription factor activity
GO:0051052	11	905	7.11949e-08	regulation of DNA metabolic process
GO:0048538	7	155	7.45158e-08	thymus development
GO:0043406	10	661	8.92404e-08	positive regulation of MAP kinase activity
GO:0043405	11	930	9.52927e-08	regulation of MAP kinase activity
GO:0031396	9	451	9.60781e-08	regulation of protein ubiquitination
GO:1903034	12	1274	1.23103e-07	regulation of response to wounding

Table 6: Overrepresented terms with the network-based enrichment. Only terms not detected with the standard method.

GO Term	N1	N2	P-value	Description
GO:0016477	15	2574	1.25782e-07	cell migration
GO:0010623	6	83	1.37019e-07	developmental programmed cell death
GO:0001933	11	963	1.38315e-07	negative regulation of protein phosphorylation
GO:0022407	8	297	1.39095e-07	regulation of cell-cell adhesion
GO:0006954	12	1314	1.76034e-07	inflammatory response
GO:0001666	11	987	1.7987e-07	response to hypoxia
GO:2001020	8	308	1.85768e-07	regulation of response to DNA damage stimulus
GO:0051100	8	309	1.90619e-07	negative regulation of binding
GO:0036293	11	999	2.04609e-07	response to decreased oxygen levels
GO:0032103	10	736	2.55155e-07	positive regulation of response to external stimulus
GO:0046328	9	505	2.61914e-07	regulation of JNK cascade
GO:0007010	14	2220	2.92639e-07	cytoskeleton organization
GO:0009636	9	517	3.22402e-07	response to toxic substance
GO:0051047	11	1063	3.96304e-07	positive regulation of secretion
GO:0048870	15	2808	4.33593e-07	cell motility
GO:0002684	14	2287	4.34607e-07	positive regulation of immune system process
GO:0000165	9	537	4.50908e-07	MAPK cascade
GO:0035065	6	103	5.13993e-07	regulation of histone acetylation
GO:0051090	11	1091	5.22396e-07	regulation of sequence-specific DNA binding transcription factor activity
GO:0006470	9	550	5.56938e-07	protein dephosphorylation
GO:1902275	8	355	5.73288e-07	regulation of chromatin organization
GO:0090287	9	557	6.22689e-07	regulation of cellular response to growth factor stimulus
GO:0071222	8	359	6.26463e-07	cellular response to lipopolysaccharide
GO:0042493	12	1473	6.56831e-07	response to drug
GO:0045664	13	1894	6.86711e-07	regulation of neuron differentiation
GO:0032872	9	568	7.3989e-07	regulation of stress-activated MAPK cascade
GO:0070302	9	569	7.51448e-07	regulation of stress-activated protein kinase signaling cascade
GO:0001764	9	578	8.629e-07	neuron migration
GO:0032355	8	374	8.65986e-07	response to estradiol
GO:0045732	8	374	8.65986e-07	positive regulation of protein catabolic process
GO:0071407	11	1148	8.96545e-07	cellular response to organic cyclic compound
GO:0048646	15	2968	9.50059e-07	anatomical structure formation involved in morphogenesis
GO:0032496	10	847	1.00057e-06	response to lipopolysaccharide
GO:0071219	8	382	1.02364e-06	cellular response to molecule of bacterial origin
GO:0001101	11	1170	1.09612e-06	response to acid chemical
GO:0035412	5	50	1.30056e-06	regulation of catenin import into nucleus
GO:0043066	14	2487	1.3202e-06	negative regulation of apoptotic process
GO:0043254	10	874	1.35627e-06	regulation of protein complex assembly
GO:0070613	9	609	1.36664e-06	regulation of protein processing
GO:1903317	9	609	1.36664e-06	regulation of protein maturation
GO:2000756	6	121	1.36946e-06	regulation of peptidyl-lysine acetylation
GO:0071396	11	1198	1.40764e-06	cellular response to lipid
GO:0045668	6	122	1.43959e-06	negative regulation of osteoblast differentiation
GO:0061136	7	237	1.46928e-06	regulation of proteasomal protein catabolic process
GO:0043123	8	400	1.47229e-06	positive regulation of I-kappaB kinase/NF-kappaB signaling
GO:0043069	14	2511	1.4988e-06	negative regulation of programmed cell death
GO:0051896	8	402	1.53136e-06	regulation of protein kinase B signaling
GO:0001501	9	620	1.59952e-06	skeletal system development
GO:1901653	10	892	1.65221e-06	cellular response to peptide
GO:0031329	15	3094	1.70785e-06	regulation of cellular catabolic process
GO:0071902	10	897	1.74403e-06	positive regulation of protein serine/threonine kinase activity
GO:0016337	10	900	1.80129e-06	single organismal cell-cell adhesion
GO:0031334	8	411	1.82342e-06	positive regulation of protein complex assembly
GO:0043122	9	630	1.84089e-06	regulation of I-kappaB kinase/NF-kappaB signaling
GO:0060627	11	1229	1.84372e-06	regulation of vesicle-mediated transport
GO:0031347	12	1612	1.84691e-06	regulation of defense response
GO:0022604	12	1615	1.8866e-06	regulation of cell morphogenesis
GO:1901983	6	128	1.92593e-06	regulation of protein acetylation
GO:0050900	9	635	1.9732e-06	leukocyte migration
GO:0052548	11	1238	1.99131e-06	regulation of endopeptidase activity

Table 7: Overrepresented terms with the network-based enrichment. Only terms not detected with the standard method.

GO Term	N1	N2	P-value	Description
GO:0043207	13	2065	1.99563e-06	response to external biotic stimulus
GO:0030182	10	911	2.02585e-06	neuron differentiation
GO:0007411	11	1242	2.06026e-06	axon guidance
GO:0097485	11	1243	2.07783e-06	neuron projection guidance
GO:0010632	8	419	2.12249e-06	regulation of epithelial cell migration
GO:0007052	5	55	2.13004e-06	mitotic spindle organization
GO:0002237	10	918	2.18143e-06	response to molecule of bacterial origin
GO:0010631	7	251	2.19218e-06	epithelial cell migration
GO:0031344	12	1640	2.24867e-06	regulation of cell projection organization
GO:0051223	12	1645	2.32824e-06	regulation of protein transport
GO:0033993	14	2604	2.42141e-06	response to lipid
GO:0000079	7	255	2.44736e-06	regulation of cyclin-dependent protein serine/threonine kinase activity
GO:0002521	10	929	2.44752e-06	leukocyte differentiation
GO:0023014	9	653	2.52163e-06	signal transduction by phosphorylation
GO:0048609	13	2109	2.58641e-06	multicellular organismal reproductive process
GO:0071216	8	432	2.69961e-06	cellular response to biotic stimulus
GO:0016485	9	661	2.80571e-06	protein processing
GO:0042692	8	437	2.95539e-06	muscle cell differentiation
GO:0052547	11	1292	3.12307e-06	regulation of peptidase activity
GO:0051781	7	266	3.28338e-06	positive regulation of cell division
GO:0048145	7	268	3.45891e-06	regulation of fibroblast proliferation
GO:0001819	10	963	3.46253e-06	positive regulation of cytokine production
GO:0048534	9	682	3.68994e-06	hematopoietic or lymphoid organ development
GO:0035914	6	143	3.76438e-06	skeletal muscle cell differentiation
GO:0098542	10	972	3.78743e-06	defense response to other organism
GO:0010720	10	977	3.97943e-06	positive regulation of cell development
GO:0050673	7	275	4.13773e-06	epithelial cell proliferation
GO:0098602	10	981	4.13922e-06	single organism cell adhesion
GO:0014013	7	276	4.24337e-06	regulation of gliogenesis
GO:0031398	7	276	4.24337e-06	positive regulation of protein ubiquitination
GO:0030282	6	146	4.26693e-06	bone mineralization
GO:0009607	13	2197	4.27161e-06	response to biotic stimulus
GO:0043524	8	459	4.34748e-06	negative regulation of neuron apoptotic process
GO:0071347	6	147	4.4463e-06	cellular response to interleukin-1
GO:0097191	7	280	4.68931e-06	extrinsic apoptotic signaling pathway
GO:0043393	8	465	4.81406e-06	regulation of protein binding
GO:0018193	13	2224	4.96108e-06	peptidyl-amino acid modification
GO:0007154	14	2752	5.00794e-06	cell communication
GO:0048589	9	714	5.51028e-06	developmental growth
GO:0007596	11	1367	5.65184e-06	blood coagulation
GO:0050817	11	1367	5.65184e-06	coagulation
GO:0030888	6	153	5.65915e-06	regulation of B cell proliferation
GO:0045619	8	475	5.68856e-06	regulation of lymphocyte differentiation
GO:0007399	10	1018	5.91149e-06	nervous system development
GO:0051051	11	1375	6.00869e-06	negative regulation of transport
GO:0007599	11	1382	6.33747e-06	hemostasis
GO:0031349	9	726	6.37375e-06	positive regulation of defense response
GO:1901988	8	482	6.37985e-06	negative regulation of cell cycle phase transition
GO:0033135	7	294	6.57833e-06	regulation of peptidyl-serine phosphorylation
GO:0016311	10	1031	6.67848e-06	dephosphorylation
GO:0007155	14	2817	6.80037e-06	cell adhesion
GO:0044087	12	1812	7.0018e-06	regulation of cellular component biogenesis
GO:0022610	14	2824	7.02499e-06	biological adhesion
GO:0002252	11	1396	7.0439e-06	immune effector process
GO:0001822	8	489	7.14296e-06	kidney development
GO:0001894	8	489	7.14296e-06	tissue homeostasis
GO:0032102	9	737	7.26768e-06	negative regulation of response to external stimulus
GO:0055088	7	299	7.39397e-06	lipid homeostasis
GO:0032091	6	160	7.40863e-06	negative regulation of protein binding
GO:0051604	9	741	7.61908e-06	protein maturation

Table 8: Overrepresented terms with the network-based enrichment. Only terms not detected with the standard method.

GO Term	N1	N2	P-value	Description
GO:1901216	6	161	7.69163e-06	positive regulation of neuron death
GO:0010717	6	162	7.98357e-06	regulation of epithelial to mesenchymal transition
GO:0000122	13	2314	8.05831e-06	negative regulation of transcription from RNA polymerase II promoter
GO:0050878	12	1837	8.17967e-06	regulation of body fluid levels
GO:0048285	10	1056	8.40667e-06	organelle fission
GO:0010035	11	1420	8.42122e-06	response to inorganic substance
GO:0034599	8	502	8.77197e-06	cellular response to oxidative stress
GO:0051707	11	1426	8.80134e-06	response to other organism
GO:0090090	7	307	8.8777e-06	negative regulation of canonical Wnt signaling pathway
GO:0003006	13	2335	8.99773e-06	developmental process involved in reproduction
GO:0019725	12	1855	9.13585e-06	cellular homeostasis
GO:0034655	14	2890	9.50317e-06	nucleobase-containing compound catabolic process
GO:0043903	8	510	9.92705e-06	regulation of symbiosis, encompassing mutualism through parasitism
GO:1901565	14	2901	9.98672e-06	organonitrogen compound catabolic process
GO:0051770	4	24	1.04759e-05	positive regulation of nitric-oxide synthase biosynthetic process
GO:0097305	10	1084	1.08056e-05	response to alcohol
GO:1903050	7	316	1.08428e-05	regulation of proteolysis involved in cellular protein catabolic process
GO:2000241	7	316	1.08428e-05	regulation of reproductive process
GO:0031056	7	318	1.13264e-05	regulation of histone modification
GO:0042742	8	519	1.13821e-05	defense response to bacterium
GO:0021987	6	172	1.1443e-05	cerebral cortex development
GO:0060249	9	783	1.23172e-05	anatomical structure homeostasis
GO:0051641	11	1477	1.27101e-05	cellular localization
GO:0042592	15	3571	1.27173e-05	homeostatic process
GO:0042110	9	787	1.28755e-05	T cell activation
GO:0032677	6	176	1.31363e-05	regulation of interleukin-8 production
GO:0050778	11	1484	1.33535e-05	positive regulation of immune response
GO:0002253	10	1110	1.35601e-05	activation of immune response
GO:0007611	9	793	1.37548e-05	learning or memory
GO:0010769	10	1115	1.41562e-05	regulation of cell morphogenesis involved in differentiation
GO:0006979	10	1117	1.44011e-05	response to oxidative stress
GO:0040007	11	1496	1.4525e-05	growth
GO:0007063	4	26	1.47239e-05	regulation of sister chromatid cohesion
GO:0051099	7	331	1.49401e-05	positive regulation of binding
GO:0019221	10	1123	1.51586e-05	cytokine-mediated signaling pathway
GO:0001667	8	542	1.59698e-05	ameboidal cell migration
GO:0048754	8	542	1.59698e-05	branching morphogenesis of an epithelial tube
GO:2000113	15	3633	1.61567e-05	negative regulation of cellular macromolecule biosynthetic process
GO:0048545	11	1516	1.66837e-05	response to steroid hormone
GO:0044270	14	3027	1.73855e-05	cellular nitrogen compound catabolic process
GO:0003012	9	815	1.74479e-05	muscle system process
GO:0046700	14	3029	1.75356e-05	heterocycle catabolic process
GO:0044702	15	3664	1.81806e-05	single organism reproductive process
GO:1903308	7	342	1.87225e-05	regulation of chromatin modification
GO:0071260	6	187	1.8894e-05	cellular response to mechanical stimulus
GO:1900542	13	2485	1.91986e-05	regulation of purine nucleotide metabolic process
GO:0019439	14	3055	1.95981e-05	aromatic compound catabolic process
GO:0032844	10	1155	1.98298e-05	regulation of homeostatic process
GO:0071248	7	346	2.02861e-05	cellular response to metal ion
GO:0002009	10	1159	2.04956e-05	morphogenesis of an epithelium
GO:0006140	13	2500	2.06537e-05	regulation of nucleotide metabolic process
GO:0009743	8	561	2.08923e-05	response to carbohydrate
GO:1902105	9	834	2.13119e-05	regulation of leukocyte differentiation
GO:0030030	13	2507	2.13665e-05	cell projection organization
GO:0033500	8	567	2.26977e-05	carbohydrate homeostasis
GO:0042593	8	567	2.26977e-05	glucose homeostasis
GO:0034097	12	2017	2.35327e-05	response to cytokine
GO:0019216	9	845	2.3877e-05	regulation of lipid metabolic process
GO:0009150	13	2531	2.39839e-05	purine ribonucleotide metabolic process
GO:0071375	9	846	2.4123e-05	cellular response to peptide hormone stimulus

Table 9: Overrepresented terms with the network-based enrichment. Only terms not detected with the standard method.

GO Term	N1	N2	P-value	Description
GO:0032434	6	196	2.50337e-05	regulation of proteasomal ubiquitin-dependent protein catabolic process
GO:0050773	7	357	2.51705e-05	regulation of dendrite development
GO:0090263	6	197	2.58075e-05	positive regulation of canonical Wnt signaling pathway
GO:0043618	5	90	2.64424e-05	regulation of transcription from RNA polymerase II promoter in response to stress
GO:0045667	7	360	2.66641e-05	regulation of osteoblast differentiation
GO:0006195	12	2041	2.68859e-05	purine nucleotide catabolic process
GO:0010558	15	3771	2.70983e-05	negative regulation of macromolecule biosynthetic process
GO:0043269	11	1590	2.74049e-05	regulation of ion transport
GO:0021953	7	365	2.93208e-05	central nervous system neuron differentiation
GO:0042476	7	366	2.98783e-05	odontogenesis
GO:0051172	15	3798	2.99108e-05	negative regulation of nitrogen compound metabolic process
GO:0009259	13	2583	3.06842e-05	ribonucleotide metabolic process
GO:0034103	6	203	3.08775e-05	regulation of tissue remodeling
GO:0072523	12	2067	3.10012e-05	purine-containing compound catabolic process
GO:0006163	13	2586	3.11183e-05	purine nucleotide metabolic process
GO:0043281	8	591	3.13406e-05	regulation of cysteine-type endopeptidase activity involved in apoptotic process
GO:1901361	14	3169	3.15354e-05	organic cyclic compound catabolic process
GO:0002064	7	370	3.21993e-05	epithelial cell development
GO:0071478	7	370	3.21993e-05	cellular response to radiation
GO:0019693	13	2596	3.26061e-05	ribose phosphate metabolic process
GO:0045444	7	372	3.34161e-05	fat cell differentiation
GO:0001938	6	207	3.4695e-05	positive regulation of endothelial cell proliferation
GO:0051250	7	375	3.53143e-05	negative regulation of lymphocyte activation
GO:0051348	9	887	3.63428e-05	negative regulation of transferase activity
GO:0035335	7	377	3.66302e-05	peptidyl-tyrosine dephosphorylation
GO:0031018	5	96	3.66654e-05	endocrine pancreas development
GO:0009617	8	605	3.75956e-05	response to bacterium
GO:0048638	8	605	3.75956e-05	regulation of developmental growth
GO:0050670	8	605	3.75956e-05	regulation of lymphocyte proliferation
GO:0071345	11	1640	3.77996e-05	cellular response to cytokine stimulus
GO:0050796	8	606	3.80811e-05	regulation of insulin secretion
GO:0046425	6	211	3.88951e-05	regulation of JAK-STAT cascade
GO:0032869	8	609	3.95701e-05	cellular response to insulin stimulus
GO:0032944	8	609	3.95701e-05	regulation of mononuclear cell proliferation
GO:0050890	9	896	3.96581e-05	cognition
GO:0009166	12	2116	4.03371e-05	nucleotide catabolic process
GO:0051301	7	386	4.30793e-05	cell division
GO:1901292	12	2130	4.34357e-05	nucleoside phosphate catabolic process
GO:0051607	7	387	4.38522e-05	defense response to virus
GO:0071241	7	388	4.46363e-05	cellular response to inorganic substance
GO:0070663	8	621	4.60423e-05	regulation of leukocyte proliferation
GO:0050863	9	912	4.62119e-05	regulation of T cell activation
GO:0051249	10	1264	4.68252e-05	regulation of lymphocyte activation
GO:0061138	8	624	4.77964e-05	morphogenesis of a branching epithelium
GO:2000116	8	624	4.77964e-05	regulation of cysteine-type endopeptidase activity
GO:0009205	12	2149	4.79847e-05	purine ribonucleoside triphosphate metabolic process
GO:0038093	8	627	4.96084e-05	Fc receptor signaling pathway
GO:0009144	12	2159	5.05481e-05	purine nucleoside triphosphate metabolic process
GO:0000910	6	222	5.26658e-05	cytokinesis
GO:0009199	12	2170	5.35098e-05	ribonucleoside triphosphate metabolic process
GO:0033365	7	399	5.40823e-05	protein localization to organelle
GO:0010771	5	105	5.76617e-05	negative regulation of cell morphogenesis involved in differentiation
GO:0043620	5	105	5.76617e-05	regulation of DNA-templated transcription in response to stress
GO:0072521	13	2722	5.77853e-05	purine-containing compound metabolic process
GO:0007267	12	2185	5.78015e-05	cell-cell signaling
GO:0007050	7	403	5.79138e-05	cell cycle arrest
GO:0002696	9	941	6.05447e-05	positive regulation of leukocyte activation
GO:0050877	13	2735	6.11991e-05	neurological system process
GO:0001525	9	949	6.51284e-05	angiogenesis
GO:0044057	10	1316	6.86501e-05	regulation of system process

Table 10: Overrepresented terms with the network-based enrichment. Only terms not detected with the standard method.

GO Term	N1	N2	P-value	Description
GO:0002088	5	109	6.96246e-05	lens development in camera-type eye
GO:0009141	12	2225	7.08091e-05	nucleoside triphosphate metabolic process
GO:0009611	8	657	7.12583e-05	response to wounding
GO:0009749	7	416	7.19989e-05	response to glucose
GO:0090317	6	234	7.20574e-05	negative regulation of intracellular protein transport
GO:0045471	7	417	7.31931e-05	response to ethanol
GO:0030336	8	660	7.38161e-05	negative regulation of cell migration
GO:0050769	8	660	7.38161e-05	positive regulation of neurogenesis
GO:0071482	6	237	7.77304e-05	cellular response to light stimulus
GO:0002757	9	972	8.00478e-05	immune response-activating signal transduction
GO:0022411	9	972	8.00478e-05	cellular component disassembly
GO:0043550	5	113	8.34838e-05	regulation of lipid kinase activity
GO:0046434	12	2260	8.42986e-05	organophosphate catabolic process
GO:0090276	8	675	8.783e-05	regulation of peptide hormone secretion
GO:0002573	6	242	8.80045e-05	myeloid leukocyte differentiation
GO:0035821	6	242	8.80045e-05	modification of morphology or physiology of other organism
GO:0051091	8	677	8.98621e-05	positive regulation of sequence-specific DNA binding transcription factor activity
GO:0050707	7	430	9.03178e-05	regulation of cytokine secretion
GO:0046580	5	115	9.11909e-05	negative regulation of Ras protein signal transduction
GO:0042113	7	431	9.1765e-05	B cell activation
GO:0048468	12	2280	9.30097e-05	cell development
GO:0048729	10	1359	9.30788e-05	tissue morphogenesis
GO:0023052	12	2287	9.62453e-05	signaling
GO:0044700	12	2287	9.62453e-05	single organism signaling
GO:0070555	6	246	9.70097e-05	response to interleukin-1
GO:0002791	8	684	9.72973e-05	regulation of peptide secretion
GO:0050867	9	996	9.8734e-05	positive regulation of cell activation
GO:0043506	6	247	9.93759e-05	regulation of JUN kinase activity
GO:0038123	5	117	9.94531e-05	toll-like receptor TLR1:TLR2 signaling pathway
GO:0038124	5	117	9.94531e-05	toll-like receptor TLR6:TLR2 signaling pathway
GO:0090087	8	688	0.000101781	regulation of peptide transport
GO:2000146	8	688	0.000101781	negative regulation of cell motility
GO:0009746	7	439	0.000104069	response to hexose
GO:0001763	8	690	0.000104088	morphogenesis of a branching structure
GO:0000278	8	691	0.000105259	mitotic cell cycle
GO:1901990	8	692	0.000106441	regulation of mitotic cell cycle phase transition
GO:2000106	6	251	0.000109324	regulation of leukocyte apoptotic process
GO:0009057	12	2318	0.000111831	macromolecule catabolic process
GO:0072593	6	252	0.000111935	reactive oxygen species metabolic process
GO:0003013	7	444	0.000112448	circulatory system process
GO:0035666	5	120	0.000112952	TRIF-dependent toll-like receptor signaling pathway
GO:0051058	5	120	0.000112952	negative regulation of small GTPase mediated signal transduction
GO:0009895	7	445	0.000114191	negative regulation of catabolic process
GO:0043900	9	1017	0.000118117	regulation of multi-organism process
GO:0007049	10	1396	0.000119975	cell cycle
GO:0002089	4	43	0.000120506	lens morphogenesis in camera-type eye
GO:0010595	5	122	0.000122733	positive regulation of endothelial cell migration
GO:0072331	6	256	0.000122903	signal transduction by p53 class mediator
GO:0001503	7	452	0.000127043	ossification
GO:0003382	5	123	0.000127869	epithelial cell morphogenesis
GO:0033673	8	709	0.000128367	negative regulation of kinase activity
GO:0046651	6	258	0.00012871	lymphocyte proliferation
GO:0050708	8	710	0.00012977	regulation of protein secretion
GO:0051271	8	710	0.00012977	negative regulation of cellular component movement
GO:0002756	5	124	0.000133175	MyD88-independent toll-like receptor signaling pathway
GO:0034134	5	124	0.000133175	toll-like receptor 2 signaling pathway
GO:0032943	6	260	0.000134743	mononuclear cell proliferation
GO:0051222	9	1034	0.000136174	positive regulation of protein transport
GO:0007265	7	457	0.000136956	Ras protein signal transduction
GO:0050729	6	261	0.000137846	positive regulation of inflammatory response

Table 11: Overrepresented terms with the network-based enrichment. Only terms not detected with the standard method.

GO Term	N1	N2	P-value	Description
GO:0034284	7	458	0.000139015	response to monosaccharide
GO:0002695	7	459	0.000141101	negative regulation of leukocyte activation
GO:0010629	14	3561	0.00014179	negative regulation of gene expression
GO:1901987	8	721	0.000146101	regulation of cell cycle phase transition
GO:0006282	5	127	0.000150151	regulation of DNA repair
GO:0055082	10	1433	0.000153551	cellular chemical homeostasis
GO:0034138	5	128	0.000156176	toll-like receptor 3 signaling pathway
GO:0032990	9	1056	0.000163106	cell part morphogenesis
GO:0031638	6	269	0.000164858	zymogen activation
GO:0030856	7	470	0.00016585	regulation of epithelial cell differentiation
GO:0046128	12	2404	0.000167703	purine ribonucleoside metabolic process
GO:0071496	8	736	0.000171214	cellular response to external stimulus
GO:0048608	9	1062	0.000171216	reproductive structure development
GO:0048878	12	2410	0.000172409	chemical homeostasis
GO:0010927	8	737	0.000173013	cellular component assembly involved in morphogenesis
GO:0042278	12	2412	0.000174005	purine nucleoside metabolic process
GO:0048660	6	272	0.000176052	regulation of smooth muscle cell proliferation
GO:0042475	6	273	0.00017992	odontogenesis of dentin-containing tooth
GO:0044419	11	1908	0.000180183	interspecies interaction between organisms
GO:0032388	8	743	0.000184154	positive regulation of intracellular transport
GO:0044708	10	1465	0.000189049	single-organism behavior
GO:0009203	11	1920	0.000192148	ribonucleoside triphosphate catabolic process
GO:0009207	11	1920	0.000192148	purine ribonucleoside triphosphate catabolic process
GO:0043087	11	1921	0.000193176	regulation of GTPase activity
GO:0009146	11	1925	0.000197339	purine nucleoside triphosphate catabolic process
GO:0045088	8	751	0.00019997	regulation of innate immune response
GO:0006259	12	2443	0.000200491	DNA metabolic process
GO:0033124	11	1931	0.000203735	regulation of GTP catabolic process
GO:1902106	6	279	0.00020463	negative regulation of leukocyte differentiation
GO:0033137	4	49	0.000206264	negative regulation of peptidyl-serine phosphorylation
GO:0009143	11	1934	0.000207002	nucleoside triphosphate catabolic process
GO:0038095	7	487	0.000211297	Fc-epsilon receptor signaling pathway
GO:0046427	5	136	0.000211622	positive regulation of JAK-STAT cascade
GO:0045927	8	757	0.000212588	positive regulation of growth
GO:0070661	6	281	0.000213464	leukocyte proliferation
GO:0007281	7	489	0.000217278	germ cell development
GO:0007601	8	760	0.000219151	visual perception
GO:0048661	5	137	0.000219528	positive regulation of smooth muscle cell proliferation
GO:0009615	8	761	0.000221378	response to virus
GO:0071456	6	283	0.000222609	cellular response to hypoxia
GO:0032757	4	50	0.000224087	positive regulation of interleukin-8 production
GO:0045934	14	3691	0.000224281	negative regulation of nucleobase-containing compound metabolic process
GO:1901136	12	2479	0.000235773	carbohydrate derivative catabolic process
GO:0050953	8	769	0.000239901	sensory perception of light stimulus
GO:0051056	8	769	0.000239901	regulation of small GTPase mediated signal transduction
GO:0048546	4	51	0.000243036	digestive tract morphogenesis
GO:0048286	5	140	0.000244663	lung alveolus development
GO:0036294	6	288	0.000246898	cellular response to decreased oxygen levels
GO:0009119	12	2491	0.000248721	ribonucleoside metabolic process
GO:0030198	9	1111	0.000251809	extracellular matrix organization
GO:0018209	6	289	0.000252009	peptidyl-serine modification
GO:0006152	11	1974	0.000255263	purine nucleoside catabolic process
GO:0046130	11	1974	0.000255263	purine ribonucleoside catabolic process
GO:0045165	7	501	0.00025626	cell fate commitment
GO:0002274	6	290	0.000257207	myeloid leukocyte activation
GO:0050866	7	502	0.00025976	negative regulation of cell activation
GO:0043062	9	1116	0.000261651	extracellular structure organization
GO:0007264	10	1517	0.000262377	small GTPase mediated signal transduction
GO:0007186	12	2507	0.000266981	G-protein coupled receptor signaling pathway
GO:0048812	8	780	0.000267547	neuron projection morphogenesis

Table 12: Overrepresented terms with the network-based enrichment. Only terms not detected with the standard method.

GO Term	N1	N2	P-value	Description
GO:0007610	11	1993	0.000281529	behavior
GO:0006310	7	508	0.00028162	DNA recombination
GO:0001952	6	295	0.000284541	regulation of cell-matrix adhesion
GO:0042454	11	2000	0.000291798	ribonucleoside catabolic process
GO:0002755	5	146	0.000301779	MyD88-dependent toll-like receptor signaling pathway
GO:0060411	5	146	0.000301779	cardiac septum morphogenesis
GO:0030855	9	1135	0.000302192	epithelial cell differentiation
GO:0000302	7	514	0.000305019	response to reactive oxygen species
GO:0009154	11	2010	0.000307049	purine ribonucleotide catabolic process
GO:0032387	6	299	0.000308096	negative regulation of intracellular transport
GO:0009261	11	2011	0.000308613	ribonucleotide catabolic process
GO:0008584	6	300	0.000314229	male gonad development
GO:0000902	8	800	0.000324898	cell morphogenesis
GO:0032675	6	302	0.000326798	regulation of interleukin-6 production
GO:2000736	6	302	0.000326798	regulation of stem cell differentiation
GO:0046777	7	520	0.000330043	protein autophosphorylation
GO:0006927	3	12	0.000330268	transformed cell apoptotic process
GO:0060744	3	12	0.000330268	mammary gland branching involved in thelarche
GO:0090153	3	12	0.000330268	regulation of sphingolipid biosynthetic process
GO:2000303	3	12	0.000330268	regulation of ceramide biosynthetic process
GO:0043567	4	55	0.000331018	regulation of insulin-like growth factor receptor signaling pathway
GO:0002700	6	303	0.000333236	regulation of production of molecular mediator of immune response
GO:0009116	12	2561	0.000337872	nucleoside metabolic process
GO:0046883	8	805	0.00034079	regulation of hormone secretion
GO:0009790	9	1155	0.000350706	embryo development
GO:0001936	6	306	0.000353182	regulation of endothelial cell proliferation
GO:0042534	4	56	0.000356301	regulation of tumor necrosis factor biosynthetic process
GO:0045598	6	307	0.000360045	regulation of fat cell differentiation
GO:0009164	11	2045	0.000366198	nucleoside catabolic process
GO:0002040	4	57	0.000382999	sprouting angiogenesis
GO:1901658	11	2055	0.00038487	glycosyl compound catabolic process
GO:0043270	7	532	0.000385335	positive regulation of ion transport
GO:0009117	13	3194	0.00039099	nucleotide metabolic process
GO:0006461	13	3196	0.000393904	protein complex assembly
GO:0090316	7	535	0.00040032	positive regulation of intracellular protein transport
GO:1901657	12	2611	0.000418176	glycosyl compound metabolic process
GO:0045834	6	315	0.000419013	positive regulation of lipid metabolic process
GO:0048666	7	539	0.000421065	neuron development
GO:0000226	8	828	0.000422839	microtubule cytoskeleton organization
GO:0032330	5	157	0.000433666	regulation of chondrocyte differentiation
GO:0006753	13	3223	0.000435205	nucleoside phosphate metabolic process
GO:0000904	7	542	0.000437217	cell morphogenesis involved in differentiation
GO:0031331	7	542	0.000437217	positive regulation of cellular catabolic process
GO:0050864	6	319	0.000451353	regulation of B cell activation
GO:0071453	6	319	0.000451353	cellular response to oxygen levels
GO:0060560	6	320	0.000459752	developmental growth involved in morphogenesis
GO:0040013	8	840	0.00047203	negative regulation of locomotion
GO:0045843	4	60	0.00047209	negative regulation of striated muscle tissue development
GO:1901862	4	60	0.00047209	negative regulation of muscle tissue development
GO:0045892	13	3247	0.000475173	negative regulation of transcription, DNA-templated
GO:0032642	5	160	0.000476575	regulation of chemokine production
GO:0010810	7	552	0.000494888	regulation of cell-substrate adhesion
GO:0033121	11	2107	0.000496375	regulation of purine nucleotide catabolic process
GO:0046034	9	1204	0.000499392	ATP metabolic process
GO:0030811	11	2111	0.000506038	regulation of nucleotide catabolic process
GO:0007568	8	848	0.000507497	aging
GO:0070647	10	1628	0.000508147	protein modification by small protein conjugation or removal
GO:0051495	7	556	0.000519693	positive regulation of cytoskeleton organization
GO:0042542	6	327	0.000522223	response to hydrogen peroxide
GO:0032870	10	1636	0.000531935	cellular response to hormone stimulus

Table 13: Overrepresented terms with the network-based enrichment. Only terms not detected with the standard method.

GO Term	N1	N2	P-value	Description
GO:0001838	4	62	0.000539518	embryonic epithelial tube formation
GO:0010823	4	62	0.000539518	negative regulation of mitochondrion organization
GO:0009118	11	2125	0.000541206	regulation of nucleoside metabolic process
GO:0031667	9	1219	0.000554757	response to nutrient levels
GO:0051149	5	165	0.00055552	positive regulation of muscle cell differentiation
GO:1902679	13	3295	0.000565236	negative regulation of RNA biosynthetic process
GO:0051384	7	564	0.000572468	response to glucocorticoid
GO:0072175	4	63	0.000575805	epithelial tube formation
GO:1902042	4	63	0.000575805	negative regulation of extrinsic apoptotic signaling pathway via death domain receptors
GO:0006897	9	1228	0.00059049	endocytosis
GO:0010830	5	169	0.000625861	regulation of myotube differentiation
GO:0016032	10	1665	0.000626628	viral process
GO:0044403	10	1665	0.000626628	symbiosis, encompassing mutualism through parasitism
GO:0051251	8	874	0.000639104	positive regulation of lymphocyte activation
GO:1903362	6	339	0.000645535	regulation of cellular protein catabolic process
GO:1901976	4	65	0.000653809	regulation of cell cycle checkpoint
GO:1903036	6	341	0.000668244	positive regulation of response to wounding
GO:0001818	7	578	0.000675738	negative regulation of cytokine production
GO:0071901	6	342	0.000679843	negative regulation of protein serine/threonine kinase activity
GO:0034142	5	172	0.000683107	toll-like receptor 4 signaling pathway
GO:0044764	10	1682	0.000688808	multi-organism cellular process
GO:0044703	7	580	0.000691705	multi-organism reproductive process
GO:0050830	5	173	0.000703085	defense response to Gram-positive bacterium
GO:0006508	13	3357	0.000704378	proteolysis
GO:0009952	7	585	0.000733014	anterior/posterior pattern specification
GO:0051253	13	3369	0.000734646	negative regulation of RNA metabolic process
GO:0090092	7	586	0.000741522	regulation of transmembrane receptor protein serine/threonine kinase signaling pathway
GO:0002065	5	175	0.000744431	columnar/cuboidal epithelial cell differentiation
GO:0001701	8	893	0.000752963	in utero embryonic development
GO:0008630	5	176	0.000765814	intrinsic apoptotic signaling pathway in response to DNA damage
GO:0045727	5	176	0.000765814	positive regulation of translation
GO:0043901	6	350	0.000778738	negative regulation of multi-organism process
GO:0002718	5	177	0.000787683	regulation of cytokine production involved in immune response
GO:0090183	5	177	0.000787683	regulation of kidney development
GO:0055086	13	3391	0.000793206	nucleobase-containing small molecule metabolic process
GO:0009167	9	1274	0.000806352	purine ribonucleoside monophosphate metabolic process
GO:0009126	9	1275	0.000811721	purine nucleoside monophosphate metabolic process
GO:0045637	7	596	0.000831287	regulation of myeloid cell differentiation
GO:0001947	5	179	0.000832899	heart looping
GO:0007585	5	179	0.000832899	respiratory gaseous exchange
GO:0048635	4	69	0.000833153	negative regulation of muscle organ development
GO:0009887	10	1719	0.000843322	organ morphogenesis
GO:0010975	9	1281	0.000844606	regulation of neuron projection development
GO:0043009	8	909	0.000861987	chordate embryonic development
GO:0043542	5	182	0.00090456	endothelial cell migration
GO:0048525	5	182	0.00090456	negative regulation of viral process
GO:0070301	5	182	0.00090456	cellular response to hydrogen peroxide
GO:0007067	7	605	0.000919759	mitotic nuclear division
GO:0046823	5	183	0.000929503	negative regulation of nucleocytoplasmic transport
GO:2000108	4	71	0.000935458	positive regulation of leukocyte apoptotic process
GO:0032886	6	362	0.000949065	regulation of microtubule-based process
GO:0044255	12	2817	0.000962284	cellular lipid metabolic process
GO:0031960	7	610	0.000972254	response to corticosteroid
GO:0002685	6	365	0.000996115	regulation of leukocyte migration
GO:0032956	8	928	0.00100886	regulation of actin cytoskeleton organization
GO:0016567	9	1310	0.00102051	protein ubiquitination
GO:0048511	8	930	0.00102551	rhythmic process
GO:0050804	8	931	0.00103391	regulation of synaptic transmission
GO:0048857	5	187	0.00103478	neural nucleus development
GO:0009991	9	1313	0.00104041	response to extracellular stimulus

Table 14: Overrepresented terms with the network-based enrichment. Only terms not detected with the standard method.

GO Term	N1	N2	P-value	Description
GO:2001233	9	1313	0.00104041	regulation of apoptotic signaling pathway
GO:0030858	5	188	0.00106252	positive regulation of epithelial cell differentiation
GO:0009792	8	937	0.00108563	embryo development ending in birth or egg hatching
GO:0001817	10	1774	0.00112959	regulation of cytokine production
GO:0006275	6	373	0.00113105	regulation of DNA replication
GO:0042108	5	191	0.00114929	positive regulation of cytokine biosynthetic process
GO:0048814	5	191	0.00114929	regulation of dendrite morphogenesis
GO:0048147	4	75	0.00116783	negative regulation of fibroblast proliferation
GO:0071322	5	192	0.00117943	cellular response to carbohydrate stimulus
GO:1903321	5	192	0.00117943	negative regulation of protein modification by small protein conjugation or removal
GO:0006631	8	949	0.00119577	fatty acid metabolic process
GO:0051493	9	1336	0.00120457	regulation of cytoskeleton organization
GO:0051817	5	193	0.00121019	modification of morphology or physiology of other organism involved in symbiotic interaction
GO:0010038	8	951	0.00121503	response to metal ion
GO:0016568	9	1339	0.00122757	chromatin modification
GO:1902582	12	2881	0.00122973	single-organism intracellular transport
GO:0009161	9	1341	0.00124311	ribonucleoside monophosphate metabolic process
GO:0002761	6	381	0.00128068	regulation of myeloid leukocyte differentiation
GO:0051346	9	1348	0.00129887	negative regulation of hydrolase activity
GO:0033081	4	77	0.00129895	regulation of T cell differentiation in thymus
GO:0030163	7	637	0.00130159	protein catabolic process
GO:0002429	7	638	0.0013154	immune response-activating cell surface receptor signaling pathway
GO:0007507	7	638	0.0013154	heart development
GO:0048858	8	963	0.00133629	cell projection morphogenesis
GO:0002263	6	385	0.00136138	cell activation involved in immune response
GO:0002366	6	385	0.00136138	leukocyte activation involved in immune response
GO:0030217	6	386	0.0013822	T cell differentiation
GO:0031647	6	386	0.0013822	regulation of protein stability
GO:0000280	8	969	0.00140072	nuclear division
GO:0030278	7	644	0.00140088	regulation of ossification
GO:0007219	6	388	0.00142461	Notch signaling pathway
GO:0043627	7	647	0.00144535	response to estrogen
GO:0009123	9	1367	0.00146135	nucleoside monophosphate metabolic process
GO:0050671	6	392	0.00151266	positive regulation of lymphocyte proliferation
GO:0007051	5	202	0.00151664	spindle organization
GO:0006919	5	203	0.00155416	activation of cysteine-type endopeptidase activity involved in apoptotic process
GO:0019637	14	4312	0.00160261	organophosphate metabolic process
GO:0032946	6	396	0.00160513	positive regulation of mononuclear cell proliferation
GO:0035148	6	396	0.00160513	tube formation
GO:0009612	7	658	0.00161873	response to mechanical stimulus
GO:0010608	9	1385	0.00163135	posttranscriptional regulation of gene expression
GO:0010001	5	205	0.0016314	glial cell differentiation
GO:0007059	5	206	0.00167114	chromosome segregation
GO:0031641	4	82	0.00167462	regulation of myelination
GO:0061035	5	207	0.00171165	regulation of cartilage development
GO:0032970	8	996	0.00172483	regulation of actin filament-based process
GO:0045787	6	401	0.00172721	positive regulation of cell cycle
GO:0022412	8	998	0.00175121	cellular process involved in reproduction in multicellular organism
GO:0034762	8	998	0.00175121	regulation of transmembrane transport
GO:0021700	7	666	0.00175556	developmental maturation
GO:2001234	7	666	0.00175556	negative regulation of apoptotic signaling pathway
GO:0010543	4	83	0.00175852	regulation of platelet activation
GO:0042100	4	83	0.00175852	B cell proliferation
GO:2000178	4	83	0.00175852	negative regulation of neural precursor cell proliferation
GO:0050792	6	403	0.00177813	regulation of viral process
GO:0070665	6	403	0.00177813	positive regulation of leukocyte proliferation
GO:0006469	7	668	0.00179125	negative regulation of protein kinase activity
GO:0043407	5	209	0.00179499	negative regulation of MAP kinase activity
GO:0045685	5	210	0.00183785	regulation of glial cell differentiation
GO:0032735	4	84	0.00184552	positive regulation of interleukin-12 production

Table 15: Overrepresented terms with the network-based enrichment. Only terms not detected with the standard method.

GO Term	N1	N2	P-value	Description
GO:0060429	8	1006	0.00186022	epithelium development
GO:0010634	5	212	0.00192599	positive regulation of epithelial cell migration
GO:0048641	5	212	0.00192599	regulation of skeletal muscle tissue development
GO:0060041	5	212	0.00192599	retina development in camera-type eye
GO:0097202	5	212	0.00192599	activation of cysteine-type endopeptidase activity
GO:0034976	6	410	0.00196615	response to endoplasmic reticulum stress
GO:0046578	6	410	0.00196615	regulation of Ras protein signal transduction
GO:0048732	8	1014	0.00197501	gland development
GO:0017157	6	411	0.0019943	regulation of exocytosis
GO:0051054	6	411	0.0019943	positive regulation of DNA metabolic process
GO:0043154	5	214	0.00201745	negative regulation of cysteine-type endopeptidase activity involved in apoptotic process
GO:0021952	4	86	0.00202907	central nervous system projection neuron axonogenesis
GO:0043388	4	86	0.00202907	positive regulation of DNA binding
GO:0051147	6	413	0.00205159	regulation of muscle cell differentiation
GO:0065003	13	3681	0.00207524	macromolecular complex assembly
GO:0032446	9	1426	0.00208425	protein modification by small protein conjugation
GO:0003143	5	216	0.00211231	embryonic heart tube morphogenesis
GO:0008544	6	418	0.00220074	epidermis development
GO:0043551	4	88	0.00222589	regulation of phosphatidylinositol 3-kinase activity
GO:0007584	7	690	0.002226	response to nutrient
GO:0046903	10	1910	0.00223469	secretion
GO:0030029	8	1031	0.00223929	actin filament-based process
GO:0000077	5	219	0.00226118	DNA damage checkpoint
GO:0097306	5	219	0.00226118	cellular response to alcohol
GO:0006520	9	1443	0.00230199	cellular amino acid metabolic process
GO:0033138	5	220	0.00231261	positive regulation of peptidyl-serine phosphorylation
GO:0050918	4	89	0.00232946	positive chemotaxis
GO:0008406	6	424	0.00239136	gonad development
GO:0043279	6	424	0.00239136	response to alkaloid
GO:1901654	6	426	0.00245784	response to ketone
GO:0042594	6	427	0.00249164	response to starvation
GO:0002697	8	1046	0.00249714	regulation of immune effector process
GO:0045639	5	226	0.00264092	positive regulation of myeloid cell differentiation
GO:0035414	3	23	0.00264421	negative regulation of catenin import into nucleus
GO:0051101	5	227	0.00269904	regulation of DNA binding
GO:0046907	13	3768	0.00272378	intracellular transport
GO:0009913	5	228	0.00275817	epidermal cell differentiation
GO:0003156	4	93	0.00277999	regulation of organ formation
GO:0032722	4	93	0.00277999	positive regulation of chemokine production
GO:0043124	4	93	0.00277999	negative regulation of I-kappaB kinase/NF-kappaB signaling
GO:0050829	4	93	0.00277999	defense response to Gram-negative bacterium
GO:2001021	4	93	0.00277999	negative regulation of response to DNA damage stimulus
GO:0045765	7	715	0.00282479	regulation of angiogenesis
GO:0008217	6	438	0.00288933	regulation of blood pressure
GO:0051928	5	231	0.00294174	positive regulation of calcium ion transport
GO:0001816	5	232	0.00300503	cytokine production
GO:0060742	3	24	0.00302046	epithelial cell differentiation involved in prostate gland development
GO:0090307	3	24	0.00302046	spindle assembly involved in mitosis
GO:0021533	4	95	0.00302812	cell differentiation in hindbrain
GO:0045862	5	233	0.00306939	positive regulation of proteolysis
GO:0090068	7	724	0.00307114	positive regulation of cell cycle process
GO:0001960	4	96	0.00315818	negative regulation of cytokine-mediated signaling pathway
GO:0002224	5	235	0.00320137	toll-like receptor signaling pathway
GO:0034764	4	97	0.00329235	positive regulation of transmembrane transport
GO:0042990	5	237	0.0033378	regulation of transcription factor import into nucleus
GO:0060070	5	237	0.0033378	canonical Wnt signaling pathway
GO:2000648	5	237	0.0033378	positive regulation of stem cell proliferation
GO:0010463	3	25	0.00343063	mesenchymal cell proliferation
GO:0002687	5	239	0.00347879	positive regulation of leukocyte migration
GO:0090277	5	239	0.00347879	positive regulation of peptide hormone secretion

Table 16: Overrepresented terms with the network-based enrichment. Only terms not detected with the standard method.

GO Term	N1	N2	P-value	Description
GO:0032526	6	457	0.00369837	response to retinoic acid
GO:0031330	5	243	0.00377486	negative regulation of cellular catabolic process
GO:0050714	6	460	0.0038416	positive regulation of protein secretion
GO:0007252	3	26	0.00387619	I-kappaB phosphorylation
GO:0060711	3	26	0.00387619	labyrinthine layer development
GO:0006921	4	102	0.00402785	cellular component disassembly involved in execution phase of apoptosis
GO:0033628	4	102	0.00402785	regulation of cell adhesion mediated by integrin
GO:0045840	4	102	0.00402785	positive regulation of mitosis
GO:0002793	5	247	0.0040905	positive regulation of peptide secretion
GO:0010955	5	247	0.0040905	negative regulation of protein processing
GO:0018105	5	247	0.0040905	peptidyl-serine phosphorylation
GO:1903318	5	247	0.0040905	negative regulation of protein maturation
GO:2001235	6	466	0.00414173	positive regulation of apoptotic signaling pathway
GO:0010506	5	248	0.00417255	regulation of autophagy
GO:0030307	6	468	0.00424592	positive regulation of cell growth
GO:0009266	6	469	0.00429884	response to temperature stimulus
GO:0016202	6	469	0.00429884	regulation of striated muscle tissue development
GO:0003018	5	250	0.00434062	vascular process in circulatory system
GO:0007631	5	250	0.00434062	feeding behavior
GO:0051224	6	470	0.00435229	negative regulation of protein transport
GO:0008361	4	104	0.00435383	regulation of cell size
GO:0034146	4	104	0.00435383	toll-like receptor 5 signaling pathway
GO:0045620	4	104	0.00435383	negative regulation of lymphocyte differentiation
GO:0060563	4	104	0.00435383	neuroepithelial cell differentiation
GO:1901861	6	471	0.00440627	regulation of muscle tissue development
GO:0034166	4	105	0.00452395	toll-like receptor 10 signaling pathway
GO:0046879	5	253	0.00460265	hormone secretion
GO:0007409	6	476	0.0046845	axonogenesis
GO:0045600	4	106	0.004699	positive regulation of fat cell differentiation
GO:0006325	9	1572	0.00470539	chromatin organization
GO:1901342	7	774	0.00479423	regulation of vasculature development
GO:0001890	4	107	0.00487903	placenta development
GO:0034121	4	107	0.00487903	regulation of toll-like receptor signaling pathway
GO:1902850	3	28	0.00487915	microtubule cytoskeleton organization involved in mitosis
GO:0048634	6	482	0.00503723	regulation of muscle organ development
GO:0032787	9	1592	0.00522687	monocarboxylic acid metabolic process
GO:0044706	6	486	0.00528424	multi-multicellular organism process
GO:0045089	6	486	0.00528424	positive regulation of innate immune response
GO:0045638	5	261	0.00536286	negative regulation of myeloid cell differentiation
GO:0007017	9	1599	0.00542091	microtubule-based process
GO:0014067	3	29	0.00543946	negative regulation of phosphatidylinositol 3-kinase signaling
GO:0060428	3	29	0.00543946	lung epithelium development
GO:0060326	6	489	0.00547594	cell chemotaxis
GO:0022404	5	263	0.00556756	molting cycle process
GO:0022405	5	263	0.00556756	hair cycle process
GO:0032755	4	111	0.00565079	positive regulation of interleukin-6 production
GO:0060761	4	111	0.00565079	negative regulation of response to cytokine stimulus
GO:0070232	4	111	0.00565079	regulation of T cell apoptotic process
GO:0060606	5	265	0.00577837	tube closure
GO:0017015	5	266	0.00588612	regulation of transforming growth factor beta receptor signaling pathway
GO:0008016	6	497	0.00601516	regulation of heart contraction
GO:0034644	4	113	0.00606901	cellular response to UV
GO:0061515	4	113	0.00606901	myeloid cell development
GO:0006690	5	269	0.00621889	icosanoid metabolic process
GO:0060021	5	269	0.00621889	palate development
GO:1901568	5	269	0.00621889	fatty acid derivative metabolic process
GO:0022409	4	114	0.00628652	positive regulation of cell-cell adhesion
GO:0032729	4	114	0.00628652	positive regulation of interferon-gamma production
GO:0043392	4	114	0.00628652	negative regulation of DNA binding
GO:0050731	6	503	0.00644748	positive regulation of peptidyl-tyrosine phosphorylation

Table 17: Overrepresented terms with the network-based enrichment. Only terms not detected with the standard method.

GO Term	N1	N2	P-value	Description
GO:0010954	5	271	0.00644885	positive regulation of protein processing
GO:1903319	5	271	0.00644885	positive regulation of protein maturation
GO:2000243	4	115	0.00650977	positive regulation of reproductive process
GO:0051769	3	31	0.00668475	regulation of nitric-oxide synthase biosynthetic process
GO:0030193	5	273	0.00668547	regulation of blood coagulation
GO:1900046	5	273	0.00668547	regulation of hemostasis
GO:0055010	4	116	0.00673888	ventricular cardiac muscle tissue morphogenesis
GO:0007268	8	1195	0.00678461	synaptic transmission
GO:0007229	5	274	0.00680631	integrin-mediated signaling pathway
GO:0071560	6	511	0.00706317	cellular response to transforming growth factor beta stimulus
GO:0050715	5	277	0.0071792	positive regulation of cytokine secretion
GO:0042535	3	32	0.00737264	positive regulation of tumor necrosis factor biosynthetic process
GO:0060297	3	32	0.00737264	regulation of sarcomere organization
GO:0021515	4	119	0.00746212	cell differentiation in spinal cord
GO:0030279	4	119	0.00746212	negative regulation of ossification
GO:0045216	6	516	0.00747182	cell-cell junction organization
GO:0010565	6	517	0.00755585	regulation of cellular ketone metabolic process
GO:0007626	7	829	0.00756365	locomotory behavior
GO:0030595	5	281	0.00770129	leukocyte chemotaxis
GO:0032653	4	120	0.00771555	regulation of interleukin-10 production
GO:0007275	9	1673	0.00788693	multicellular organismal development
GO:0007417	6	521	0.00789961	central nervous system development
GO:0071559	6	521	0.00789961	response to transforming growth factor beta
GO:0010959	7	835	0.00793383	regulation of metal ion transport
GO:0007600	9	1675	0.00796522	sensory perception
GO:0034162	4	121	0.00797532	toll-like receptor 9 signaling pathway
GO:0033599	3	33	0.0081059	regulation of mammary gland epithelial cell proliferation
GO:0035112	3	33	0.0081059	genitalia morphogenesis
GO:0021549	4	122	0.00824152	cerebellum development
GO:0048640	4	122	0.00824152	negative regulation of developmental growth
GO:1902692	4	122	0.00824152	regulation of neuroblast proliferation
GO:0001959	5	285	0.00825283	regulation of cytokine-mediated signaling pathway
GO:0055001	5	285	0.00825283	muscle cell development
GO:0031333	5	286	0.00839548	negative regulation of protein complex assembly
GO:0010594	5	287	0.00854003	regulation of endothelial cell migration
GO:0009914	5	288	0.00868657	hormone transport
GO:0043280	5	288	0.00868657	positive regulation of cysteine-type endopeptidase activity involved in apoptotic process
GO:2000177	5	288	0.00868657	regulation of neural precursor cell proliferation
GO:0070373	4	124	0.00879359	negative regulation of ERK1 and ERK2 cascade
GO:0045662	3	34	0.00888594	negative regulation of myoblast differentiation
GO:0072148	3	34	0.00888594	epithelial cell fate commitment
GO:1900739	3	34	0.00888594	regulation of protein insertion into mitochondrial membrane involved in apoptotic signaling pathway
GO:1900740	3	34	0.00888594	positive regulation of protein insertion into mitochondrial membrane involved in apoptotic signaling pathway
GO:0001954	4	125	0.0090797	positive regulation of cell-matrix adhesion
GO:0003007	4	125	0.0090797	heart morphogenesis
GO:0002699	6	536	0.00930485	positive regulation of immune effector process
GO:1901888	4	126	0.00937268	regulation of cell junction assembly
GO:0046620	5	293	0.00944916	regulation of organ growth
GO:0051225	4	127	0.00967261	spindle assembly
GO:1902041	4	127	0.00967261	regulation of extrinsic apoptotic signaling pathway via death domain receptors
GO:0051153	5	295	0.00976856	regulation of striated muscle cell differentiation
GO:0030099	6	543	0.0100272	myeloid cell differentiation
GO:0033559	5	297	0.0100964	unsaturated fatty acid metabolic process
GO:0030522	6	544	0.010134	intracellular receptor signaling pathway
GO:2001244	4	129	0.0102937	positive regulation of intrinsic apoptotic signaling pathway
GO:0050818	5	299	0.0104329	regulation of coagulation
GO:0014912	3	36	0.010592	negative regulation of smooth muscle cell migration
GO:0030540	3	36	0.010592	female genitalia development
GO:0048485	3	36	0.010592	sympathetic nervous system development
GO:0070507	5	300	0.0106044	regulation of microtubule cytoskeleton organization

Table 18: Overrepresented terms with the network-based enrichment. Only terms not detected with the standard method.

GO Term	N1	N2	P-value	Description
GO:0071356	5	301	0.0107781	cellular response to tumor necrosis factor
GO:0090288	5	301	0.0107781	negative regulation of cellular response to growth factor stimulus
GO:0051924	6	553	0.011138	regulation of calcium ion transport
GO:0007005	6	554	0.0112543	mitochondrion organization
GO:0010464	4	132	0.0112802	regulation of mesenchymal cell proliferation
GO:0031589	6	556	0.0114901	cell-substrate adhesion
GO:0035264	5	305	0.0114955	multicellular organism growth
GO:0002062	4	133	0.0116242	chondrocyte differentiation
GO:0002221	5	306	0.0116805	pattern recognition receptor signaling pathway
GO:2000179	4	134	0.0119758	positive regulation of neural precursor cell proliferation
GO:0046887	5	308	0.0120577	positive regulation of hormone secretion
GO:2001251	4	135	0.0123353	negative regulation of chromosome organization
GO:0006352	6	564	0.0124739	DNA-templated transcription, initiation
GO:0032321	6	565	0.0126016	positive regulation of Rho GTPase activity
GO:2000107	4	136	0.0127026	negative regulation of leukocyte apoptotic process
GO:0051092	5	312	0.0128409	positive regulation of NF-kappaB transcription factor activity
GO:0046632	4	137	0.013078	alpha-beta T cell differentiation
GO:0003136	2	4	0.0130923	negative regulation of heart induction by canonical Wnt signaling pathway
GO:0009997	2	4	0.0130923	negative regulation of cardioblast cell fate specification
GO:0014908	2	4	0.0130923	myotube differentiation involved in skeletal muscle regeneration
GO:0042686	2	4	0.0130923	regulation of cardioblast cell fate specification
GO:1902445	2	4	0.0130923	regulation of mitochondrial membrane permeability involved in programmed necrotic cell death
GO:1990009	2	4	0.0130923	retinal cell apoptotic process
GO:0051276	6	569	0.0131231	chromosome organization
GO:0043547	9	1783	0.013332	positive regulation of GTPase activity
GO:0006629	12	3598	0.0134207	lipid metabolic process
GO:0033233	3	39	0.0135374	regulation of protein sumoylation
GO:0002758	5	316	0.0136636	innate immune response-activating signal transduction
GO:0090101	5	316	0.0136636	negative regulation of transmembrane receptor protein serine/threonine kinase signaling pathway
GO:2000027	6	574	0.0137994	regulation of organ morphogenesis
GO:2001056	5	317	0.0138756	positive regulation of cysteine-type endopeptidase activity
GO:0034330	6	577	0.0142187	cell junction organization
GO:0042098	4	140	0.0142538	T cell proliferation
GO:0006260	6	578	0.0143608	DNA replication
GO:0030036	7	914	0.014413	actin cytoskeleton organization
GO:0009950	3	40	0.0146277	dorsal/ventral axis specification
GO:0033598	3	40	0.0146277	mammary gland epithelial cell proliferation
GO:0060765	3	40	0.0146277	regulation of androgen receptor signaling pathway
GO:2001236	6	581	0.014794	regulation of extrinsic apoptotic signaling pathway
GO:0030216	4	142	0.0150799	keratinocyte differentiation
GO:0050921	5	324	0.015433	positive regulation of chemotaxis
GO:0019752	11	2978	0.0157216	carboxylic acid metabolic process
GO:0032105	3	41	0.0157747	negative regulation of response to extracellular stimulus
GO:0032108	3	41	0.0157747	negative regulation of response to nutrient levels
GO:0044030	3	41	0.0157747	regulation of DNA methylation
GO:0060644	3	41	0.0157747	mammary gland epithelial cell differentiation
GO:0071158	4	144	0.015941	positive regulation of cell cycle arrest
GO:0016570	7	929	0.0160424	histone modification
GO:0072091	5	327	0.0161412	regulation of stem cell proliferation
GO:0060562	5	329	0.0166273	epithelial tube morphogenesis
GO:0016569	7	935	0.0167359	covalent chromatin modification
GO:0044711	12	3675	0.0167891	single-organism biosynthetic process
GO:0090200	3	42	0.0169798	positive regulation of release of cytochrome c from mitochondria
GO:0006644	7	938	0.017092	phospholipid metabolic process
GO:0001709	4	147	0.0173003	cell fate determination
GO:0048592	4	147	0.0173003	eye morphogenesis
GO:0014706	5	333	0.017634	striated muscle tissue development
GO:0071333	4	148	0.0177719	cellular response to glucose stimulus
GO:0044265	9	1850	0.0180419	cellular macromolecule catabolic process
GO:0001889	5	335	0.018155	liver development

Table 19: Overrepresented terms with the network-based enrichment. Only terms not detected with the standard method.

GO Term	N1	N2	P-value	Description
GO:0043200	5	335	0.018155	response to amino acid
GO:0070230	3	43	0.0182442	positive regulation of lymphocyte apoptotic process
GO:0030534	6	605	0.0186579	adult behavior
GO:0051785	4	152	0.019754	positive regulation of nuclear division
GO:1990138	4	152	0.019754	neuron projection extension
GO:0002218	5	341	0.0197908	activation of innate immune response
GO:0010976	5	341	0.0197908	positive regulation of neuron projection development
GO:0048015	5	342	0.0200744	phosphatidylinositol-mediated signaling
GO:0048017	5	342	0.0200744	inositol lipid-mediated signaling
GO:0016192	11	3055	0.0201714	vesicle-mediated transport
GO:0016458	4	153	0.0202741	gene silencing
GO:0006986	5	345	0.0209442	response to unfolded protein
GO:0045737	3	45	0.0209569	positive regulation of cyclin-dependent protein serine/threonine kinase activity
GO:0007178	6	618	0.0210718	transmembrane receptor protein serine/threonine kinase signaling pathway
GO:0010212	5	346	0.0212406	response to ionizing radiation
GO:0001658	4	155	0.0213445	branching involved in ureteric bud morphogenesis
GO:0022600	4	155	0.0213445	digestive system process
GO:0031058	4	155	0.0213445	positive regulation of histone modification
GO:0090407	8	1397	0.0216132	organophosphate biosynthetic process
GO:0034334	2	5	0.0218105	adherens junction maintenance
GO:0048524	4	156	0.0218952	positive regulation of viral process
GO:0070374	5	349	0.0221497	positive regulation of ERK1 and ERK2 cascade
GO:0034394	3	46	0.0224079	protein localization to cell surface
GO:0042308	4	157	0.0224563	negative regulation of protein import into nucleus
GO:0010950	5	351	0.0227726	positive regulation of endopeptidase activity
GO:0045666	5	351	0.0227726	positive regulation of neuron differentiation
GO:0071331	4	158	0.0230279	cellular response to hexose stimulus
GO:0007283	8	1413	0.0235037	spermatogenesis
GO:0050795	6	630	0.0235203	regulation of behavior
GO:0046631	4	159	0.0236103	alpha-beta T cell activation
GO:0048678	4	159	0.0236103	response to axon injury
GO:0006576	5	355	0.0240595	cellular biogenic amine metabolic process
GO:0044106	5	355	0.0240595	cellular amine metabolic process
GO:0045069	4	160	0.0242034	regulation of viral genome replication
GO:0071326	4	160	0.0242034	cellular response to monosaccharide stimulus
GO:1903310	4	160	0.0242034	positive regulation of chromatin modification
GO:0048232	8	1420	0.024374	male gamete generation
GO:0034341	5	356	0.02439	response to interferon-gamma
GO:0060485	4	161	0.0248076	mesenchyme development
GO:0070542	4	161	0.0248076	response to fatty acid
GO:0001568	5	358	0.0250616	blood vessel development
GO:2001237	5	358	0.0250616	negative regulation of extrinsic apoptotic signaling pathway
GO:0048839	4	162	0.0254228	inner ear development
GO:0061097	4	162	0.0254228	regulation of protein tyrosine kinase activity
GO:0010092	3	48	0.0255062	specification of organ identity
GO:0035066	3	48	0.0255062	positive regulation of histone acetylation
GO:0048146	4	164	0.0266869	positive regulation of fibroblast proliferation
GO:0009584	5	363	0.0268043	detection of visible light
GO:0071392	3	49	0.0271562	cellular response to estradiol stimulus
GO:0007179	5	364	0.0271639	transforming growth factor beta receptor signaling pathway
GO:0055008	4	165	0.0273362	cardiac muscle tissue morphogenesis
GO:0050870	6	647	0.0273811	positive regulation of T cell activation
GO:0042035	5	365	0.0275273	regulation of cytokine biosynthetic process
GO:0032655	4	166	0.027997	regulation of interleukin-12 production
GO:0045861	4	167	0.0286696	negative regulation of proteolysis
GO:0051606	8	1452	0.0287106	detection of stimulus
GO:0010935	3	50	0.0288752	regulation of macrophage cytokine production
GO:0006200	7	1017	0.0290303	ATP catabolic process
GO:0030098	6	654	0.0291138	lymphocyte differentiation
GO:0032092	4	168	0.029354	positive regulation of protein binding

Table 20: Overrepresented terms with the network-based enrichment. Only terms not detected with the standard method.

GO Term	N1	N2	P-value	Description
GO:0001678	4	169	0.0300504	cellular glucose homeostasis
GO:0072089	4	169	0.0300504	stem cell proliferation
GO:0010837	3	51	0.0306647	regulation of keratinocyte proliferation
GO:0030511	3	51	0.0306647	positive regulation of transforming growth factor beta receptor signaling pathway
GO:0031397	4	170	0.030759	negative regulation of protein ubiquitination
GO:0009158	7	1027	0.0309451	ribonucleoside monophosphate catabolic process
GO:0009169	7	1027	0.0309451	purine ribonucleoside monophosphate catabolic process
GO:0009128	7	1028	0.0311422	purine nucleoside monophosphate catabolic process
GO:0035966	5	375	0.0313758	response to topologically incorrect protein
GO:0050730	6	663	0.0314716	regulation of peptidyl-tyrosine phosphorylation
GO:0035295	6	664	0.0317428	tube development
GO:0009125	7	1033	0.0321437	nucleoside monophosphate catabolic process
GO:0070228	4	172	0.0322131	regulation of lymphocyte apoptotic process
GO:0002063	3	52	0.0325261	chondrocyte development
GO:0032007	3	52	0.0325261	negative regulation of TOR signaling
GO:0050775	3	52	0.0325261	positive regulation of dendrite morphogenesis
GO:0070233	3	52	0.0325261	negative regulation of T cell apoptotic process
GO:0030100	6	667	0.0325681	regulation of endocytosis
GO:0001649	5	378	0.0326088	osteoblast differentiation
GO:0051591	5	378	0.0326088	response to cAMP
GO:0050771	4	174	0.0337174	negative regulation of axonogenesis
GO:0034612	5	381	0.0338795	response to tumor necrosis factor
GO:0060441	3	53	0.0344606	epithelial tube branching involved in lung morphogenesis
GO:0071174	3	53	0.0344606	mitotic spindle checkpoint
GO:0072182	3	53	0.0344606	regulation of nephron tubule epithelial cell differentiation
GO:0090342	3	53	0.0344606	regulation of cell aging
GO:0007569	4	175	0.0344887	cell aging
GO:0048520	5	383	0.034748	positive regulation of behavior
GO:0045670	4	176	0.035273	regulation of osteoclast differentiation
GO:0045740	4	176	0.035273	positive regulation of DNA replication
GO:0010770	4	177	0.0360703	positive regulation of cell morphogenesis involved in differentiation
GO:0060688	4	177	0.0360703	regulation of morphogenesis of a branching structure
GO:0043436	11	3244	0.0361145	oxoacid metabolic process
GO:0070925	7	1052	0.036198	organelle assembly
GO:0009581	6	680	0.0363487	detection of external stimulus
GO:0045445	3	54	0.0364696	myoblast differentiation
GO:1901797	3	54	0.0364696	negative regulation of signal transduction by p53 class mediator
GO:0060537	5	387	0.0365372	muscle tissue development
GO:0006968	4	179	0.0377048	cellular defense response
GO:0050871	4	179	0.0377048	positive regulation of B cell activation
GO:1900117	3	55	0.0385545	regulation of execution phase of apoptosis
GO:0009582	6	688	0.0388473	detection of abiotic stimulus
GO:0019882	6	688	0.0388473	antigen processing and presentation
GO:0010952	5	393	0.0393556	positive regulation of peptidase activity
GO:0035239	5	393	0.0393556	tube morphogenesis
GO:0060415	4	181	0.0393934	muscle tissue morphogenesis
GO:0032024	4	182	0.0402584	positive regulation of insulin secretion
GO:0006082	11	3284	0.0406481	organic acid metabolic process
GO:0046902	3	56	0.0407167	regulation of mitochondrial membrane permeability
GO:0070059	3	56	0.0407167	intrinsic apoptotic signaling pathway in response to endoplasmic reticulum stress
GO:2000647	3	56	0.0407167	negative regulation of stem cell proliferation
GO:0045766	5	397	0.0413272	positive regulation of angiogenesis
GO:0001704	4	184	0.0420301	formation of primary germ layer
GO:0045669	4	184	0.0420301	positive regulation of osteoblast differentiation
GO:0032940	8	1530	0.0421122	secretion by cell
GO:1901605	6	700	0.0428555	alpha-amino acid metabolic process
GO:0009880	4	185	0.0429371	embryonic pattern specification
GO:0048565	4	185	0.0429371	digestive tract development
GO:0007517	5	401	0.0433751	muscle organ development
GO:0046330	4	187	0.0447947	positive regulation of JNK cascade

Table 21: Overrepresented terms with the network-based enrichment. Only terms not detected with the standard method.

GO Term	N1	N2	P-value	Description
GO:0007162	5	404	0.0449628	negative regulation of cell adhesion
GO:0002067	3	58	0.0452783	glandular epithelial cell differentiation
GO:0010719	3	58	0.0452783	negative regulation of epithelial to mesenchymal transition
GO:0033688	3	58	0.0452783	regulation of osteoblast proliferation
GO:0090559	3	58	0.0452783	regulation of membrane permeability
GO:0009308	5	405	0.0455017	amine metabolic process
GO:0061041	5	405	0.0455017	regulation of wound healing
GO:0002244	5	406	0.0460459	hematopoietic progenitor cell differentiation
GO:0050801	8	1555	0.0473981	ion homeostasis
GO:0070664	4	190	0.0476914	negative regulation of leukocyte proliferation
GO:0009314	8	1557	0.0478441	response to radiation
GO:2001252	4	191	0.0486873	positive regulation of chromosome organization
GO:2001242	5	412	0.0494193	regulation of intrinsic apoptotic signaling pathway
GO:0051146	4	192	0.0496981	striated muscle cell differentiation

Table 22: Overrepresented terms with the network-based enrichment. Only terms not detected with the standard method.
