# Supplementary material for: NET-GE: a novel NETwork-based Gene Enrichment for detecting biological processes associated to Mendelian diseases
Source: BMC Genomics. 2015 Jun 18;16(Suppl 8):S6. doi: 10.1186/1471-2164-16-S8-S6 (PMC4480278; doi:10.1186/1471-2164-16-S8-S6)
Supplement: Additional file 3 — Detailed results for the OMIM-derived benchmark set. The archive contains pdf documents listing the enriched terms for each one of the 244 diseases in the OMIM-derived benchmark set. [file 1471-2164-16-S8-S6-S3.tgz › SUPPMAT/OMIM114550.pdf]

#114550 HEPATOCELLULAR CARCINOMA

**OMIM Gene ID**	**HGNC**	**UniProtAC**
116806	CTNNB1	P35222
147280	IGF2R	P11717
164860	MET	P08581
171834	PIK3CA	P42336
191170	TP53	P04637
601763	CASP8	Q14790
603816	AXIN1	O15169
604584	PDGFRL	Q15198
611731	APC	P25054

Table 1: OMIM - UniProtAC mapping

Legend

- N1: #input proteins associated to the significant GO term
- N2: #proteins associated to the significant GO term
- P-value: Bonferroni-corrected p-value of Fisher's exact test
- *red*: go terms not related to the input proteins
- *blue*: go terms related to the input proteins (enriched uniquely by network-based method)
- *green*: go terms ancestors of terms enriched with the standard method (enriched uniquely by network-based method)

1 Standard enrichment

GO Term	N1	N2	P-value	Description
GO:0007166	9	4368	4.65081e-06	cell surface receptor signaling pathway
GO:0051247	7	1526	7.34735e-06	positive regulation of protein metabolic process
GO:0043067	7	1982	4.4956e-05	regulation of programmed cell death
GO:0010941	7	2079	6.25475e-05	regulation of cell death
GO:0032268	7	2272	0.000115444	regulation of cellular protein metabolic process
GO:0048856	8	4289	0.00028145	anatomical structure development
GO:0006921	3	54	0.000290748	cellular component disassembly involved in execution phase of apoptosis
GO:0033077	3	60	0.000400833	T cell differentiation in thymus
GO:0048585	6	1639	0.000627196	negative regulation of response to stimulus
GO:0006915	5	805	0.000643604	apoptotic process
GO:0007165	9	7592	0.000675637	signal transduction
GO:0032880	5	819	0.000700819	regulation of protein localization
GO:0051246	7	2954	0.000702604	regulation of protein metabolic process
GO:0001775	5	825	0.000726542	cell activation
GO:0042110	4	319	0.000767711	T cell activation
GO:0012501	5	868	0.000933626	programmed cell death
GO:0042325	6	1770	0.000986368	regulation of phosphorylation
GO:0031399	6	1823	0.00117326	regulation of protein modification process
GO:1902531	6	1847	0.00126702	regulation of intracellular signal transduction
GO:0010604	7	3285	0.00145452	positive regulation of macromolecule metabolic process
GO:0002521	4	375	0.00146148	leukocyte differentiation
GO:0003002	4	380	0.00154051	regionalization
GO:0048513	6	1910	0.00154296	organ development
GO:0048732	4	391	0.00172552	gland development
GO:0042981	6	1970	0.00185013	regulation of apoptotic process
GO:1902533	5	1008	0.0019503	positive regulation of intracellular signal transduction
GO:0044336	2	10	0.00285848	canonical Wnt signaling pathway involved in negative regulation of apoptotic process
GO:0007167	5	1091	0.00287739	enzyme linked receptor protein signaling pathway
GO:0009893	7	3630	0.00287769	positive regulation of metabolic process
GO:0048522	8	5768	0.00289948	positive regulation of cellular process
GO:0060070	3	116	0.0029491	canonical Wnt signaling pathway
GO:0008219	5	1106	0.00307692	cell death
GO:0016265	5	1117	0.00323008	death
GO:0042327	5	1129	0.00340398	positive regulation of phosphorylation
GO:0060548	5	1147	0.00367866	negative regulation of cell death
GO:0046649	4	484	0.00402317	lymphocyte activation
GO:0009798	3	132	0.00435098	axis specification
GO:0048646	5	1201	0.00460931	anatomical structure formation involved in morphogenesis
GO:0048584	6	2308	0.00467537	positive regulation of response to stimulus
GO:0051716	9	9450	0.00485063	cellular response to stimulus
GO:0010562	5	1255	0.00571704	positive regulation of phosphorus metabolic process
GO:0045937	5	1255	0.00571704	positive regulation of phosphate metabolic process
GO:0043065	4	536	0.00602499	positive regulation of apoptotic process
GO:0002761	3	148	0.00613622	regulation of myeloid leukocyte differentiation
GO:0043068	4	543	0.00634212	positive regulation of programmed cell death
GO:0030217	3	154	0.00691374	T cell differentiation
GO:0007389	4	579	0.00817287	pattern specification process
GO:0010942	4	579	0.00817287	positive regulation of cell death
GO:0045321	4	579	0.00817287	leukocyte activation
GO:0051259	4	582	0.00834136	protein oligomerization
GO:0009968	5	1361	0.00849842	negative regulation of signal transduction
GO:0032270	5	1363	0.00855958	positive regulation of cellular protein metabolic process
GO:0060341	5	1363	0.00855958	regulation of cellular localization
GO:0048518	8	6624	0.00857238	positive regulation of biological process
GO:0006461	5	1365	0.0086211	protein complex assembly
GO:0001889	3	173	0.00980036	liver development
GO:0044767	8	6740	0.00981841	single-organism developmental process
GO:0044707	7	4361	0.0100237	single-multicellular organism process
GO:0023057	5	1420	0.0104543	negative regulation of signaling
GO:0010648	5	1424	0.0105987	negative regulation of cell communication

Table 2: Overrepresented GO terms with the standard enrichment

GO Term	N1	N2	P-value	Description
GO:0001932	5	1440	0.0111922	regulation of protein phosphorylation
GO:0032501	7	4447	0.0114422	multicellular organismal process
GO:0048583	7	4515	0.0126809	regulation of response to stimulus
GO:0070243	2	22	0.0146518	regulation of thymocyte apoptotic process
GO:0032879	6	2827	0.015227	regulation of localization
GO:0009967	5	1548	0.0159184	positive regulation of signal transduction
GO:0060272	2	23	0.0160452	embryonic skeletal joint morphogenesis
GO:0060768	2	23	0.0160452	regulation of epithelial cell proliferation involved in prostate gland development
GO:0042221	7	4712	0.016929	response to chemical
GO:0050793	6	2884	0.0170952	regulation of developmental process
GO:0032502	8	7299	0.0182877	developmental process
GO:0065003	5	1623	0.0200353	macromolecular complex assembly
GO:0023056	5	1630	0.0204586	positive regulation of signaling
GO:0019220	6	2977	0.0205452	regulation of phosphate metabolic process
GO:0009950	2	26	0.0206038	dorsal/ventral axis specification
GO:0010647	5	1637	0.0208888	positive regulation of cell communication
GO:0051174	6	2996	0.0213157	regulation of phosphorus metabolic process
GO:0045637	3	225	0.0215133	regulation of myeloid cell differentiation
GO:2000027	3	225	0.0215133	regulation of organ morphogenesis
GO:1900739	2	27	0.0222492	regulation of protein insertion into mitochondrial membrane involved in apoptotic signaling pathway
GO:1900740	2	27	0.0222492	positive regulation of protein insertion into mitochondrial membrane involved in apoptotic signaling pathway
GO:0009952	3	235	0.0244958	anterior/posterior pattern specification
GO:0090287	3	237	0.0251233	regulation of cellular response to growth factor stimulus
GO:0043408	4	794	0.0283236	regulation of MAPK cascade
GO:0007169	4	798	0.0288873	transmembrane receptor protein tyrosine kinase signaling pathway
GO:0050896	9	11721	0.0337221	response to stimulus
GO:0030098	3	263	0.0342685	lymphocyte differentiation
GO:0045596	4	834	0.0343416	negative regulation of cell differentiation
GO:0009966	6	3261	0.0347792	regulation of signal transduction
GO:0048523	7	5279	0.0364236	negative regulation of cellular process
GO:0071496	3	275	0.0391399	cellular response to external stimulus
GO:0001838	2	36	0.0398902	embryonic epithelial tube formation
GO:0050790	6	3371	0.0421041	regulation of catalytic activity
GO:0072175	2	37	0.0421644	epithelial tube formation
GO:0031325	6	3418	0.0455963	positive regulation of cellular metabolic process
GO:0051239	6	3432	0.0466812	regulation of multicellular organismal process
GO:0071822	5	1933	0.0467155	protein complex subunit organization
GO:0031122	2	40	0.0493634	cytoplasmic microtubule organization

Table 3: Overrepresented GO terms with the standard enrichment

2 Network-based enrichment

GO Term	N1	N2	P-value	Description
GO:0030111	7	814	4.70478e-07	regulation of Wnt signaling pathway
GO:0045786	7	824	5.12345e-07	negative regulation of cell cycle
GO:0008544	6	418	8.24269e-07	epidermis development
GO:0035412	4	50	1.51324e-06	regulation of catenin import into nucleus
GO:0001933	7	963	1.5199e-06	negative regulation of protein phosphorylation
GO:0060828	6	550	4.27051e-06	regulation of canonical Wnt signaling pathway
GO:0070302	6	569	5.23273e-06	regulation of stress-activated protein kinase signaling cascade
GO:0042326	7	1164	5.68891e-06	negative regulation of phosphorylation
GO:0070613	6	609	7.85516e-06	regulation of protein processing
GO:1903317	6	609	7.85516e-06	regulation of protein maturation
GO:0060627	7	1229	8.30055e-06	regulation of vesicle-mediated transport
GO:0007346	7	1240	8.83084e-06	regulation of mitotic cell cycle
GO:0051249	7	1264	1.00892e-05	regulation of lymphocyte activation
GO:0002684	8	2287	1.10907e-05	positive regulation of immune system process
GO:1902532	7	1289	1.15597e-05	negative regulation of intracellular signal transduction
GO:2001233	7	1313	1.31399e-05	regulation of apoptotic signaling pathway
GO:0010563	7	1353	1.61839e-05	negative regulation of phosphorus metabolic process
GO:0045936	7	1353	1.61839e-05	negative regulation of phosphate metabolic process
GO:1901990	6	692	1.68481e-05	regulation of mitotic cell cycle phase transition
GO:0031400	7	1368	1.74712e-05	negative regulation of protein modification process
GO:0080135	7	1388	1.93227e-05	regulation of cellular response to stress
GO:0007257	4	95	2.08054e-05	activation of JUN kinase activity
GO:0090090	5	307	2.11602e-05	negative regulation of canonical Wnt signaling pathway
GO:0038179	6	720	2.13462e-05	neurotrophin signaling pathway
GO:1901987	6	721	2.15237e-05	regulation of cell cycle phase transition
GO:0002694	7	1443	2.5302e-05	regulation of leukocyte activation
GO:0032386	7	1443	2.5302e-05	regulation of intracellular transport
GO:0071900	7	1492	3.18948e-05	regulation of protein serine/threonine kinase activity
GO:0009896	6	786	3.60056e-05	positive regulation of catabolic process
GO:0050865	7	1568	4.5004e-05	regulation of cell activation
GO:0042176	6	816	4.50046e-05	regulation of protein catabolic process
GO:0033043	8	2759	4.91912e-05	regulation of organelle organization
GO:0032147	6	832	5.052e-05	activation of protein kinase activity
GO:0051130	8	2782	5.25388e-05	positive regulation of cellular component organization
GO:0045860	7	1636	6.0386e-05	positive regulation of protein kinase activity
GO:0072310	3	23	6.47901e-05	glomerular epithelial cell development
GO:0031647	5	386	6.64088e-05	regulation of protein stability
GO:0043254	6	874	6.77222e-05	regulation of protein complex assembly
GO:0033157	6	878	6.95868e-05	regulation of intracellular protein transport
GO:0071902	6	897	7.90352e-05	positive regulation of protein serine/threonine kinase activity
GO:0033674	7	1706	8.06988e-05	positive regulation of kinase activity
GO:0051052	6	905	8.33195e-05	regulation of DNA metabolic process
GO:0043405	6	930	9.79694e-05	regulation of MAP kinase activity
GO:0000187	5	421	0.000102354	activation of MAPK activity
GO:0097193	5	421	0.000102354	intrinsic apoptotic signaling pathway
GO:0030162	7	1768	0.000103299	regulation of proteolysis
GO:0043409	5	424	0.000106039	negative regulation of MAPK cascade
GO:0043547	7	1783	0.000109513	positive regulation of GTPase activity
GO:0080134	8	3072	0.000115282	regulation of response to stress
GO:0030178	5	432	0.000116385	negative regulation of Wnt signaling pathway
GO:0032269	7	1804	0.000118746	negative regulation of cellular protein metabolic process
GO:0044087	7	1812	0.000122434	regulation of cellular component biogenesis
GO:0070201	7	1849	0.000140789	regulation of establishment of protein localization
GO:1901991	5	457	0.000153998	negative regulation of mitotic cell cycle phase transition
GO:0060429	6	1006	0.000156207	epithelium development
GO:0071363	7	1903	0.000171762	cellular response to growth factor stimulus
GO:0030335	6	1024	0.000173544	positive regulation of cell migration
GO:0051129	7	1913	0.000178092	negative regulation of cellular component organization
GO:0043087	7	1921	0.000183299	regulation of GTPase activity
GO:0030334	7	1926	0.000186618	regulation of cell migration

Table 4: Overrepresented terms with the network-based enrichment. Only terms not detected with the standard method.

GO Term	N1	N2	P-value	Description
GO:0033124	7	1931	0.000189989	regulation of GTP catabolic process
GO:2000147	6	1042	0.000192445	positive regulation of cell motility
GO:1901988	5	482	0.000200709	negative regulation of cell cycle phase transition
GO:0051272	6	1060	0.000213021	positive regulation of cellular component movement
GO:0048608	6	1062	0.000215416	reproductive structure development
GO:0070848	7	1987	0.000231433	response to growth factor
GO:0051090	6	1091	0.000252723	regulation of sequence-specific DNA binding transcription factor activity
GO:0046328	5	505	0.000253041	regulation of JNK cascade
GO:2000145	7	2039	0.000276584	regulation of cell motility
GO:1903320	5	520	0.000292644	regulation of protein modification by small protein conjugation or removal
GO:0040017	6	1124	0.000301532	positive regulation of locomotion
GO:0042306	5	527	0.000312737	regulation of protein import into nucleus
GO:0071260	4	187	0.000318849	cellular response to mechanical stimulus
GO:0033233	3	39	0.000333619	regulation of protein sumoylation
GO:0071407	6	1148	0.000341727	cellular response to organic cyclic compound
GO:0033121	7	2107	0.000346786	regulation of purine nucleotide catabolic process
GO:0048609	7	2109	0.000349063	multicellular organismal reproductive process
GO:0030811	7	2111	0.00035135	regulation of nucleotide catabolic process
GO:0048754	5	542	0.000359496	branching morphogenesis of an epithelial tube
GO:0009118	7	2125	0.000367728	regulation of nucleoside metabolic process
GO:0001101	6	1170	0.000382378	response to acid chemical
GO:0032101	7	2173	0.00042891	regulation of response to external stimulus
GO:0051270	7	2182	0.000441297	regulation of cellular component movement
GO:0051248	7	2189	0.000451143	negative regulation of protein metabolic process
GO:0032872	5	568	0.00045359	regulation of stress-activated MAPK cascade
GO:0030155	6	1217	0.000482738	regulation of cell adhesion
GO:0040012	7	2224	0.000503217	regulation of locomotion
GO:0048320	2	3	0.00052365	axial mesoderm formation
GO:0010638	6	1260	0.000592773	positive regulation of organelle organization
GO:1900180	5	602	0.00060512	regulation of protein localization to nucleus
GO:0043410	6	1269	0.000618244	positive regulation of MAPK cascade
GO:0050670	5	605	0.000620211	regulation of lymphocyte proliferation
GO:0032944	5	609	0.000640794	regulation of mononuclear cell proliferation
GO:0000122	7	2314	0.000661203	negative regulation of transcription from RNA polymerase II promoter
GO:0051101	4	227	0.000692965	regulation of DNA binding
GO:0043434	6	1303	0.000722792	response to peptide hormone
GO:0061138	5	624	0.000722881	morphogenesis of a branching epithelium
GO:0008285	7	2354	0.00074393	negative regulation of cell proliferation
GO:0070304	4	232	0.000756047	positive regulation of stress-activated protein kinase signaling cascade
GO:0045862	4	233	0.00076916	positive regulation of proteolysis
GO:0001934	7	2371	0.000781668	positive regulation of protein phosphorylation
GO:0051493	6	1336	0.000837851	regulation of cytoskeleton organization
GO:0030278	5	644	0.0008451	regulation of ossification
GO:0045859	7	2438	0.000946702	regulation of protein kinase activity
GO:0006259	7	2443	0.000960125	DNA metabolic process
GO:0043406	5	661	0.000961416	positive regulation of MAP kinase activity
GO:0007093	4	247	0.000971152	mitotic cell cycle checkpoint
GO:0043506	4	247	0.000971152	regulation of JUN kinase activity
GO:1901652	6	1373	0.000984465	response to peptide
GO:0010608	6	1385	0.00103633	posttranscriptional regulation of gene expression
GO:1901320	2	4	0.00104715	negative regulation of heart induction
GO:2000044	2	4	0.00104715	negative regulation of cardiac cell fate specification
GO:0046822	5	675	0.00106645	regulation of nucleocytoplasmic transport
GO:1900542	7	2485	0.00107941	regulation of purine nucleotide metabolic process
GO:0006140	7	2500	0.00112497	regulation of nucleotide metabolic process
GO:0045597	7	2514	0.00116895	positive regulation of cell differentiation
GO:0001763	5	690	0.0011889	morphogenesis of a branching structure
GO:0048011	5	692	0.00120604	neurotrophin TRK receptor signaling pathway
GO:0008283	7	2526	0.0012078	cell proliferation
GO:0043549	7	2576	0.00138176	regulation of kinase activity

Table 5: Overrepresented terms with the network-based enrichment. Only terms not detected with the standard method.

GO Term	N1	N2	P-value	Description
GO:0010954	4	271	0.00140625	positive regulation of protein processing
GO:1903319	4	271	0.00140625	positive regulation of protein maturation
GO:0010948	5	717	0.00143728	negative regulation of cell cycle process
GO:0033993	7	2604	0.00148817	response to lipid
GO:0048731	7	2612	0.00151982	system development
GO:0043086	7	2637	0.00162246	negative regulation of catalytic activity
GO:0032102	5	737	0.00164651	negative regulation of response to external stimulus
GO:1901028	3	66	0.00166438	regulation of mitochondrial outer membrane permeabilization involved in apoptotic signaling pathway
GO:0051291	4	286	0.00174329	protein heterooligomerization
GO:0051093	7	2690	0.00185968	negative regulation of developmental process
GO:0033135	4	294	0.00194592	regulation of peptidyl-serine phosphorylation
GO:0008584	4	300	0.00210904	male gonad development
GO:0070507	4	300	0.00210904	regulation of microtubule cytoskeleton organization
GO:0051726	7	2760	0.00221771	regulation of cell cycle
GO:0022402	7	2771	0.00227897	cell cycle process
GO:0043269	6	1590	0.002337	regulation of ion transport
GO:2001020	4	308	0.00234213	regulation of response to DNA damage stimulus
GO:0060706	3	75	0.00245302	cell differentiation involved in embryonic placenta development
GO:0000902	5	800	0.00246807	cell morphogenesis
GO:0010564	6	1606	0.00247874	regulation of cell cycle process
GO:0022604	6	1615	0.00256154	regulation of cell morphogenesis
GO:0051338	7	2819	0.00256345	regulation of transferase activity
GO:0030177	4	316	0.00259389	positive regulation of Wnt signaling pathway
GO:0009725	7	2824	0.00259475	response to hormone
GO:0030910	2	6	0.00261714	olfactory placode formation
GO:0022603	7	2832	0.0026455	regulation of anatomical structure morphogenesis
GO:0016055	5	814	0.00268846	Wnt signaling pathway
GO:0051223	6	1645	0.00285424	regulation of protein transport
GO:0051098	5	826	0.00288954	regulation of binding
GO:0048008	3	80	0.00298265	platelet-derived growth factor receptor signaling pathway
GO:1902105	5	834	0.00303007	regulation of leukocyte differentiation
GO:0010959	5	835	0.00304801	regulation of metal ion transport
GO:0016032	6	1665	0.00306427	viral process
GO:0044403	6	1665	0.00306427	symbiosis, encompassing mutualism through parasitism
GO:2000045	4	334	0.00323364	regulation of G1/S transition of mitotic cell cycle
GO:0044764	6	1682	0.00325269	multi-organism cellular process
GO:1902806	4	336	0.00331136	regulation of cell cycle G1/S phase transition
GO:0031401	7	2947	0.00347381	positive regulation of protein modification process
GO:0051345	7	2953	0.00352247	positive regulation of hydrolase activity
GO:0071901	4	342	0.00355285	negative regulation of protein serine/threonine kinase activity
GO:0051251	5	874	0.00381635	positive regulation of lymphocyte activation
GO:0045944	7	2989	0.00382676	positive regulation of transcription from RNA polymerase II promoter
GO:0045667	4	360	0.0043563	regulation of osteoblast differentiation
GO:1903047	6	1769	0.0043732	mitotic cell cycle process
GO:0016337	5	900	0.00440853	single organismal cell-cell adhesion
GO:0001817	6	1774	0.0044462	regulation of cytokine production
GO:0032886	4	362	0.00445326	regulation of microtubule-based process
GO:0006955	7	3063	0.00452295	immune response
GO:0043124	3	93	0.00470263	negative regulation of I-kappaB kinase/NF-kappaB signaling
GO:0050863	5	912	0.00470529	regulation of T cell activation
GO:0031329	7	3094	0.00484504	regulation of cellular catabolic process
GO:0060596	2	8	0.00488398	mammary placode formation
GO:0045732	4	374	0.00506912	positive regulation of protein catabolic process
GO:0050878	6	1837	0.00545555	regulation of body fluid levels
GO:0002696	5	941	0.00548808	positive regulation of leukocyte activation
GO:0010038	5	951	0.00578067	response to metal ion
GO:0051094	7	3200	0.00609775	positive regulation of developmental process
GO:0048858	5	963	0.00614797	cell projection morphogenesis
GO:0032989	6	1879	0.006228	cellular component morphogenesis
GO:0045664	6	1894	0.00652479	regulation of neuron differentiation

Table 6: Overrepresented terms with the network-based enrichment. Only terms not detected with the standard method.

GO Term	N1	N2	P-value	Description
GO:0048662	3	104	0.00658951	negative regulation of smooth muscle cell proliferation
GO:0045892	7	3247	0.00673549	negative regulation of transcription, DNA-templated
GO:0044419	6	1908	0.00681224	interspecies interaction between organisms
GO:0007050	4	403	0.00681784	cell cycle arrest
GO:0044092	7	3258	0.00689265	negative regulation of molecular function
GO:0050867	5	996	0.00725405	positive regulation of cell activation
GO:0006974	6	1933	0.00735149	cellular response to DNA damage stimulus
GO:0051347	6	1933	0.00735149	positive regulation of transferase activity
GO:0031334	4	411	0.00737054	positive regulation of protein complex assembly
GO:0045087	6	1935	0.00739611	innate immune response
GO:1902679	7	3295	0.00744433	negative regulation of RNA biosynthetic process
GO:0045471	4	417	0.00780643	response to ethanol
GO:0010632	4	419	0.00795589	regulation of epithelial cell migration
GO:0009409	3	111	0.00801888	response to cold
GO:0070232	3	111	0.00801888	regulation of T cell apoptotic process
GO:0008406	4	424	0.00833887	gonad development
GO:0006508	7	3357	0.00845281	proteolysis
GO:0031345	4	428	0.00865498	negative regulation of cell projection organization
GO:0051253	7	3369	0.0086608	negative regulation of RNA metabolic process
GO:0043392	3	114	0.00868954	negative regulation of DNA binding
GO:0050707	4	430	0.00881633	regulation of cytokine secretion
GO:0043433	4	432	0.00897992	negative regulation of sequence-specific DNA binding transcription factor activity
GO:0000075	4	434	0.00914574	cell cycle checkpoint
GO:0009894	7	3408	0.00936707	regulation of catabolic process
GO:0097190	5	1052	0.00948645	apoptotic signaling pathway
GO:0042129	4	439	0.00957027	regulation of T cell proliferation
GO:0032990	5	1056	0.00966457	cell part morphogenesis
GO:0001569	3	119	0.00988816	patterning of blood vessels
GO:0009895	4	445	0.0100988	negative regulation of catabolic process
GO:0032320	5	1071	0.0103562	positive regulation of Ras GTPase activity
GO:0010595	3	122	0.0106574	positive regulation of endothelial cell migration
GO:0045668	3	122	0.0106574	negative regulation of osteoblast differentiation
GO:0003382	3	123	0.0109223	epithelial cell morphogenesis
GO:0097305	5	1084	0.0109867	response to alcohol
GO:0032526	4	457	0.0112207	response to retinoic acid
GO:0015031	7	3506	0.0113608	protein transport
GO:0035303	4	461	0.0116144	regulation of dephosphorylation
GO:0043393	4	465	0.0120183	regulation of protein binding
GO:0009266	4	469	0.0124326	response to temperature stimulus
GO:0030856	4	470	0.0125377	regulation of epithelial cell differentiation
GO:0010629	7	3561	0.0126296	negative regulation of gene expression
GO:0042592	7	3571	0.0128728	homeostatic process
GO:2000113	7	3633	0.0144706	negative regulation of cellular macromolecule biosynthetic process
GO:0002009	5	1159	0.0152424	morphogenesis of an epithelium
GO:0044702	7	3664	0.0153305	single organism reproductive process
GO:0090244	2	14	0.0158595	Wnt signaling pathway involved in somitogenesis
GO:0045934	7	3691	0.0161144	negative regulation of nucleobase-containing compound metabolic process
GO:0042098	3	140	0.0161172	T cell proliferation
GO:0018193	6	2224	0.0166707	peptidyl-amino acid modification
GO:0035914	3	143	0.0171765	skeletal muscle cell differentiation
GO:0045184	7	3728	0.0172436	establishment of protein localization
GO:0071396	5	1198	0.0179185	cellular response to lipid
GO:0045216	4	516	0.0181343	cell-cell junction organization
GO:0030282	3	146	0.0182809	bone mineralization
GO:0046434	6	2260	0.0183049	organophosphate catabolic process
GO:0042742	4	519	0.0185542	defense response to bacterium
GO:0010558	7	3771	0.0186395	negative regulation of macromolecule biosynthetic process
GO:0018108	4	523	0.0191253	peptidyl-tyrosine phosphorylation
GO:1901215	4	524	0.0192701	negative regulation of neuron death
GO:0051172	7	3798	0.019564	negative regulation of nitrogen compound metabolic process

Table 7: Overrepresented terms with the network-based enrichment. Only terms not detected with the standard method.

GO Term	N1	N2	P-value	Description
GO:0014068	3	150	0.0198252	positive regulation of phosphatidylinositol 3-kinase signaling
GO:0006897	5	1228	0.0202189	endocytosis
GO:0018212	4	533	0.0206099	peptidyl-tyrosine modification
GO:0040008	6	2308	0.0206864	regulation of growth
GO:0052548	5	1238	0.0210354	regulation of endopeptidase activity
GO:0000165	4	537	0.0212271	MAPK cascade
GO:0048538	3	155	0.021874	thymus development
GO:0031331	4	542	0.0220177	positive regulation of cellular catabolic process
GO:0003006	6	2335	0.0221343	developmental process involved in reproduction
GO:0042308	3	157	0.0227312	negative regulation of protein import into nucleus
GO:0032091	3	160	0.0240583	negative regulation of protein binding
GO:0050767	6	2377	0.0245512	regulation of neurogenesis
GO:0050776	6	2377	0.0245512	regulation of immune response
GO:0032318	5	1285	0.0252283	regulation of Ras GTPase activity
GO:0035019	3	163	0.0254356	somatic stem cell maintenance
GO:0052547	5	1292	0.0259054	regulation of peptidase activity
GO:0007368	3	164	0.0259061	determination of left/right symmetry
GO:0009855	3	167	0.0273519	determination of bilateral symmetry
GO:0009799	3	168	0.0278454	specification of symmetry
GO:0006954	5	1314	0.0281271	inflammatory response
GO:0034330	4	577	0.0281791	cell junction organization
GO:0001764	4	578	0.028372	neuron migration
GO:2001236	4	581	0.0289565	regulation of extrinsic apoptotic signaling pathway
GO:0070228	3	172	0.0298784	regulation of lymphocyte apoptotic process
GO:0070646	3	174	0.0309309	protein modification by small protein removal
GO:0051346	5	1348	0.0318536	negative regulation of hydrolase activity
GO:0048729	5	1359	0.0331389	tissue morphogenesis
GO:0030030	6	2507	0.0334448	cell projection organization
GO:0051050	6	2507	0.0334448	positive regulation of transport
GO:0010243	6	2509	0.0336001	response to organonitrogen compound
GO:0009617	4	605	0.0339607	response to bacterium
GO:0007596	5	1367	0.0340993	blood coagulation
GO:0050817	5	1367	0.0340993	coagulation
GO:0051051	5	1375	0.0350812	negative regulation of transport
GO:0007599	5	1382	0.0359588	hemostasis
GO:0046823	3	183	0.0359698	negative regulation of nucleocytoplasmic transport
GO:1901701	6	2540	0.0360796	cellular response to oxygen-containing compound
GO:0001704	3	184	0.036561	formation of primary germ layer
GO:0001841	2	21	0.0365632	neural tube formation
GO:0001501	4	620	0.0373968	skeletal system development
GO:0070663	4	621	0.0376347	regulation of leukocyte proliferation
GO:0006497	3	186	0.0377628	protein lipidation
GO:0002252	5	1396	0.0377652	immune effector process
GO:0046330	3	187	0.0383731	positive regulation of JNK cascade
GO:0009888	6	2570	0.0386202	tissue development
GO:0016477	6	2574	0.0389697	cell migration
GO:0030858	3	188	0.0389902	positive regulation of epithelial cell differentiation
GO:0045682	3	189	0.0396137	regulation of epidermis development
GO:0043122	4	630	0.0398263	regulation of I-kappaB kinase/NF-kappaB signaling
GO:0050795	4	630	0.0398263	regulation of behavior
GO:0051960	6	2584	0.0398551	regulation of nervous system development
GO:2001239	3	190	0.0402439	regulation of extrinsic apoptotic signaling pathway in absence of ligand
GO:0045785	4	632	0.0403257	positive regulation of cell adhesion
GO:0042108	3	191	0.0408803	positive regulation of cytokine biosynthetic process
GO:0010035	5	1420	0.0410283	response to inorganic substance
GO:1903321	3	192	0.0415237	negative regulation of protein modification by small protein conjugation or removal
GO:0007507	4	638	0.0418523	heart development
GO:0051707	5	1426	0.0418776	response to other organism
GO:0043507	3	193	0.0421736	positive regulation of JUN kinase activity
GO:0034244	2	23	0.0440374	negative regulation of transcription elongation from RNA polymerase II promoter

Table 8: Overrepresented terms with the network-based enrichment. Only terms not detected with the standard method.

GO Term	N1	N2	P-value	Description
GO:0048332	2	23	0.0440374	mesoderm morphogenesis
GO:0032434	3	196	0.0441636	regulation of proteasomal ubiquitin-dependent protein catabolic process
GO:0050870	4	647	0.0442215	positive regulation of T cell activation
GO:0090263	3	197	0.0448407	positive regulation of canonical Wnt signaling pathway
GO:0023014	4	653	0.0458554	signal transduction by phosphorylation
GO:0009612	4	658	0.0472505	response to mechanical stimulus
GO:0030336	4	660	0.0478175	negative regulation of cell migration
GO:0032785	2	24	0.0480341	negative regulation of DNA-templated transcription, elongation
GO:0060638	2	24	0.0480341	mesenchymal-epithelial cell signaling
GO:0016485	4	661	0.0481027	protein processing
GO:0042493	5	1473	0.0490211	response to drug
GO:0021700	4	666	0.049548	developmental maturation
GO:2001234	4	666	0.049548	negative regulation of apoptotic signaling pathway
GO:1902807	3	204	0.0497714	negative regulation of cell cycle G1/S phase transition
GO:2000134	3	204	0.0497714	negative regulation of G1/S transition of mitotic cell cycle
GO:0030100	4	667	0.0498408	regulation of endocytosis

Table 9: Overrepresented terms with the network-based enrichment. Only terms not detected with the standard method.
