# Supplementary material for: NET-GE: a novel NETwork-based Gene Enrichment for detecting biological processes associated to Mendelian diseases
Source: BMC Genomics. 2015 Jun 18;16(Suppl 8):S6. doi: 10.1186/1471-2164-16-S8-S6 (PMC4480278; doi:10.1186/1471-2164-16-S8-S6)
Supplement: Additional file 3 — Detailed results for the OMIM-derived benchmark set. The archive contains pdf documents listing the enriched terms for each one of the 244 diseases in the OMIM-derived benchmark set. [file 1471-2164-16-S8-S6-S3.tgz › SUPPMAT/OMIM122700.pdf]

#122700 COUMARIN RESISTANCE

OMIM Gene ID	HGNC	UniProtAC
122720	CYP2A6	P11509
300746	F9	P00740
601130	CYP2C9	P11712
608547	VKORC1	Q9BQB6

Table 1: OMIM - UniProtAC mapping

Legend

- N1: #input proteins associated to the significant GO term
- N2: #proteins associated to the significant GO term
- P-value: Bonferroni-corrected p-value of Fisher's exact test
- *red*: go terms not related to the input proteins
- *blue*: go terms related to the input proteins (enriched uniquely by network-based method)
- *green*: go terms ancestors of terms enriched with the standard method (enriched uniquely by network-based method)

1 Standard enrichment

GO Term	N1	N2	P-value	Description
GO:0017144	3	37	3.18898e-07	drug metabolic process
GO:0042738	2	12	5.11327e-05	exogenous drug catabolic process
GO:0017187	2	15	8.13388e-05	peptidyl-glutamic acid carboxylation
GO:0018214	2	15	8.13388e-05	protein carboxylation
GO:0042737	2	15	8.13388e-05	drug catabolic process
GO:0018200	2	45	0.000766096	peptidyl-glutamic acid modification
GO:0006805	2	163	0.0101744	xenobiotic metabolic process
GO:0043687	2	219	0.0183588	post-translational protein modification
GO:0046226	1	2	0.0194995	coumarin catabolic process
GO:0046271	1	3	0.0292481	phenylpropanoid catabolic process
GO:0008202	2	325	0.0403402	steroid metabolic process
GO:0007598	1	5	0.048743	blood coagulation, extrinsic pathway

Table 2: Overrepresented GO terms with the standard enrichment

2 Network-based enrichment

No novel enriched terms
