# Supplementary material for: NET-GE: a novel NETwork-based Gene Enrichment for detecting biological processes associated to Mendelian diseases
Source: BMC Genomics. 2015 Jun 18;16(Suppl 8):S6. doi: 10.1186/1471-2164-16-S8-S6 (PMC4480278; doi:10.1186/1471-2164-16-S8-S6)
Supplement: Additional file 3 — Detailed results for the OMIM-derived benchmark set. The archive contains pdf documents listing the enriched terms for each one of the 244 diseases in the OMIM-derived benchmark set. [file 1471-2164-16-S8-S6-S3.tgz › SUPPMAT/OMIM125853.pdf]

#125853 DIABETES MELLITUS, NONINSULIN-DEPENDENT; NIDDM

OMIM Gene ID	HGNC	UniProtAC
138033	GCGR	P47871
138079	GCK	P35557
138190	SLC2A4	P14672
138430	GPD2	P43304
142410	HNF1A	P20823
147545	IRS1	P35568
147620	IL6	P05231
151670	LIPC	P11150
164731	AKT2	P31751
167413	PAX4	O43316
173335	ENPP1	P22413
176885	PTPN1	P18031
189907	HNF1B	P35680
600281	HNF4A	P41235
600509	ABCC8	Q09428
600701	HMGA1	P17096
600733	PDX1	P52945
600797	IRS2	Q9Y4H2
600804	MTNR1B	P49286
600937	KCNJ11	Q14654
601487	PPARG	P37231
601724	NEUROD1	Q13562
602228	TCF7L2	Q9NQB0
604641	MAPK8IP1	Q9UQF2
605565	RETN	Q9HD89
606201	WFS1	O76024
608289	IGF2BP2	Q9Y6M1
611145	SLC30A8	Q8IWU4
611259	CDKAL1	Q5VV42

Table 1: OMIM - UniProtAC mapping

Legend

- N1: #input proteins associated to the significant GO term
- N2: #proteins associated to the significant GO term
- P-value: Bonferroni-corrected p-value of Fisher's exact test
- *red*: go terms not related to the input proteins
- *blue*: go terms related to the input proteins (enriched uniquely by network-based method)
- *green*: go terms ancestors of terms enriched with the standard method (enriched uniquely by network-based method)

1 Standard enrichment

GO Term	N1	N2	P-value	Description
GO:0033500	14	264	5.22176e-20	carbohydrate homeostasis
GO:0042593	14	264	5.22176e-20	glucose homeostasis
GO:0046883	13	336	1.628e-16	regulation of hormone secretion
GO:0048878	17	1094	3.71743e-16	chemical homeostasis
GO:1901700	19	1851	2.35672e-15	response to oxygen-containing compound
GO:0042592	18	1658	1.15441e-14	homeostatic process
GO:0050796	11	258	5.94989e-14	regulation of insulin secretion
GO:0090276	11	279	1.41737e-13	regulation of peptide hormone secretion
GO:0002791	11	284	1.72556e-13	regulation of peptide secretion
GO:0090087	11	286	1.86498e-13	regulation of peptide transport
GO:0051049	18	2081	6.21988e-13	regulation of transport
GO:0031018	7	42	2.98222e-12	endocrine pancreas development
GO:0009749	9	171	9.39771e-12	response to glucose
GO:0010033	20	3487	1.33914e-11	response to organic substance
GO:0009746	9	183	1.74508e-11	response to hexose
GO:0051046	13	829	1.93217e-11	regulation of secretion
GO:0010243	14	1094	2.19671e-11	response to organonitrogen compound
GO:0034284	9	197	3.41429e-11	response to monosaccharide
GO:1901698	14	1186	6.61175e-11	response to nitrogen compound
GO:0065008	20	3888	1.07458e-10	regulation of biological quality
GO:0010906	8	133	1.20824e-10	regulation of glucose metabolic process
GO:0032879	18	2827	1.26491e-10	regulation of localization
GO:0009725	14	1273	1.73219e-10	response to hormone
GO:0032868	10	376	2.25613e-10	response to insulin
GO:0009743	9	247	2.65134e-10	response to carbohydrate
GO:0043434	11	567	3.37579e-10	response to peptide hormone
GO:0060341	14	1363	4.37569e-10	regulation of cellular localization
GO:1901652	11	600	6.23198e-10	response to peptide
GO:0023051	19	3708	8.08172e-10	regulation of signaling
GO:0010646	19	3714	8.32062e-10	regulation of cell communication
GO:0010675	8	183	1.60694e-09	regulation of cellular carbohydrate metabolic process
GO:2000674	5	17	1.78517e-09	regulation of type B pancreatic cell apoptotic process
GO:0070873	6	47	1.94055e-09	regulation of glycogen metabolic process
GO:0006109	8	191	2.26879e-09	regulation of carbohydrate metabolic process
GO:0043255	7	111	3.62438e-09	regulation of carbohydrate biosynthetic process
GO:0042221	20	4712	4.1077e-09	response to chemical
GO:0032881	6	53	4.13598e-09	regulation of polysaccharide metabolic process
GO:0009719	15	2012	4.50859e-09	response to endogenous stimulus
GO:2000675	4	6	6.56707e-09	negative regulation of type B pancreatic cell apoptotic process
GO:0071375	9	372	1.04644e-08	cellular response to peptide hormone stimulus
GO:1901701	12	1086	1.56346e-08	cellular response to oxygen-containing compound
GO:1901653	9	391	1.63128e-08	cellular response to peptide
GO:0071417	10	588	1.86938e-08	cellular response to organonitrogen compound
GO:0032869	8	270	3.63453e-08	cellular response to insulin stimulus
GO:0030072	6	77	4.21816e-08	peptide hormone secretion
GO:1901699	10	645	4.62396e-08	cellular response to nitrogen compound
GO:0002790	6	79	4.9401e-08	peptide secretion
GO:0051050	11	919	6.07868e-08	positive regulation of transport
GO:0032870	10	716	1.28017e-07	cellular response to hormone stimulus
GO:0043467	6	93	1.34482e-07	regulation of generation of precursor metabolites and energy
GO:0005979	5	39	1.64173e-07	regulation of glycogen biosynthetic process
GO:0010962	5	39	1.64173e-07	regulation of glucan biosynthetic process
GO:0048519	20	5756	1.72514e-07	negative regulation of biological process
GO:0010907	5	40	1.87526e-07	positive regulation of glucose metabolic process
GO:0015833	6	100	2.09544e-07	peptide transport
GO:0046879	6	103	2.50948e-07	hormone secretion
GO:0032885	5	45	3.47268e-07	regulation of polysaccharide biosynthetic process
GO:0009914	6	109	3.54293e-07	hormone transport
GO:0048523	19	5279	4.31789e-07	negative regulation of cellular process
GO:0030073	5	48	4.85926e-07	insulin secretion

Table 2: Overrepresented GO terms with the standard enrichment

GO Term	N1	N2	P-value	Description
GO:0006006	7	225	5.30653e-07	glucose metabolic process
GO:0045725	4	15	5.94759e-07	positive regulation of glycogen biosynthetic process
GO:0010565	7	233	6.77118e-07	regulation of cellular ketone metabolic process
GO:0070875	4	17	1.03592e-06	positive regulation of glycogen metabolic process
GO:0046887	6	133	1.18464e-06	positive regulation of hormone secretion
GO:0071310	14	2482	1.28765e-06	cellular response to organic substance
GO:0010557	13	2042	1.53331e-06	positive regulation of macromolecule biosynthetic process
GO:0042886	6	139	1.54659e-06	amide transport
GO:0010676	5	61	1.67667e-06	positive regulation of cellular carbohydrate metabolic process
GO:0046321	4	20	2.10549e-06	positive regulation of fatty acid oxidation
GO:0071495	11	1291	2.20152e-06	cellular response to endogenous stimulus
GO:0045913	5	68	2.927e-06	positive regulation of carbohydrate metabolic process
GO:0031328	13	2177	3.3409e-06	positive regulation of cellular biosynthetic process
GO:0009891	13	2208	3.96583e-06	positive regulation of biosynthetic process
GO:0010604	15	3285	4.48169e-06	positive regulation of macromolecule metabolic process
GO:0023061	6	168	4.83993e-06	signal release
GO:0019318	7	321	6.2522e-06	hexose metabolic process
GO:0006091	8	531	7.52095e-06	generation of precursor metabolites and energy
GO:0006112	6	185	8.62555e-06	energy reserve metabolic process
GO:0032024	5	86	9.68599e-06	positive regulation of insulin secretion
GO:0070887	14	2904	9.87106e-06	cellular response to chemical stimulus
GO:0071702	14	2983	1.39401e-05	organic substance transport
GO:0005996	7	362	1.43082e-05	monosaccharide metabolic process
GO:0009893	15	3630	1.76456e-05	positive regulation of metabolic process
GO:0048522	18	5768	1.97092e-05	positive regulation of cellular process
GO:0048518	19	6624	2.1818e-05	positive regulation of biological process
GO:0090277	5	101	2.18551e-05	positive regulation of peptide hormone secretion
GO:0046320	4	35	2.25738e-05	regulation of fatty acid oxidation
GO:0051179	20	7547	2.40959e-05	localization
GO:0051047	7	392	2.47112e-05	positive regulation of secretion
GO:0002793	5	104	2.53338e-05	positive regulation of peptide secretion
GO:0044765	17	5114	2.70052e-05	single-organism transport
GO:0045923	4	37	2.84435e-05	positive regulation of fatty acid metabolic process
GO:0015980	6	231	3.24418e-05	energy derivation by oxidation of organic compounds
GO:0006810	19	6814	3.52883e-05	transport
GO:0048583	16	4515	3.88559e-05	regulation of response to stimulus
GO:0051234	19	6974	5.22831e-05	establishment of localization
GO:0010827	5	128	7.19606e-05	regulation of glucose transport
GO:0031325	14	3418	7.90635e-05	positive regulation of cellular metabolic process
GO:0043066	9	1050	9.13173e-05	negative regulation of apoptotic process
GO:0043069	9	1068	0.000105545	negative regulation of programmed cell death
GO:0045834	5	139	0.000108725	positive regulation of lipid metabolic process
GO:0032502	19	7299	0.000112629	developmental process
GO:0051051	7	493	0.00011806	negative regulation of transport
GO:0032000	3	12	0.000139307	positive regulation of fatty acid beta-oxidation
GO:2000112	18	6579	0.000162238	regulation of cellular macromolecule biosynthetic process
GO:0031324	12	2479	0.000177225	negative regulation of cellular metabolic process
GO:0050896	23	11721	0.000184217	response to stimulus
GO:0060548	9	1147	0.000193491	negative regulation of cell death
GO:0010556	18	6784	0.000263635	regulation of macromolecule biosynthetic process
GO:0045444	5	167	0.000271476	fat cell differentiation
GO:0010817	6	337	0.000302041	regulation of hormone levels
GO:0019725	8	867	0.000327742	cellular homeostasis
GO:0048585	10	1639	0.000339952	negative regulation of response to stimulus
GO:0032940	7	592	0.000406842	secretion by cell
GO:0009892	12	2679	0.000412545	negative regulation of metabolic process
GO:0031326	18	6987	0.00041934	regulation of cellular biosynthetic process
GO:0060255	20	8942	0.000483288	regulation of macromolecule metabolic process
GO:0009889	18	7051	0.000483825	regulation of biosynthetic process
GO:0019216	6	375	0.000564667	regulation of lipid metabolic process

Table 3: Overrepresented GO terms with the standard enrichment

GO Term	N1	N2	P-value	Description
GO:0046888	4	79	0.000632834	negative regulation of hormone secretion
GO:0031327	10	1764	0.000669471	negative regulation of cellular biosynthetic process
GO:0031998	3	20	0.000718884	regulation of fatty acid beta-oxidation
GO:0055082	7	645	0.000723623	cellular chemical homeostasis
GO:0046324	4	82	0.00073551	regulation of glucose import
GO:0009890	10	1786	0.000750174	negative regulation of biosynthetic process
GO:0009968	9	1361	0.000816685	negative regulation of signal transduction
GO:0001678	4	86	0.0008911	cellular glucose homeostasis
GO:0071705	7	691	0.00114708	nitrogen compound transport
GO:0023057	9	1420	0.00116373	negative regulation of signaling
GO:0010648	9	1424	0.00119132	negative regulation of cell communication
GO:0048856	14	4289	0.00133841	anatomical structure development
GO:1900076	4	98	0.0015062	regulation of cellular response to insulin stimulus
GO:0044767	17	6740	0.00168015	single-organism developmental process
GO:0005975	9	1491	0.00174566	carbohydrate metabolic process
GO:0019217	4	102	0.00176818	regulation of fatty acid metabolic process
GO:0042981	10	1970	0.00183663	regulation of apoptotic process
GO:0050996	3	27	0.00183785	positive regulation of lipid catabolic process
GO:0043067	10	1982	0.00194076	regulation of programmed cell death
GO:0060397	3	28	0.00205733	JAK-STAT cascade involved in growth hormone signaling pathway
GO:0031323	20	9728	0.00207067	regulation of cellular metabolic process
GO:0019222	21	10848	0.0021872	regulation of metabolic process
GO:0065007	27	19296	0.00219759	biological regulation
GO:0046903	7	778	0.00252403	secretion
GO:0032368	4	112	0.00257055	regulation of lipid transport
GO:0009730	2	3	0.00266645	detection of carbohydrate stimulus
GO:0009732	2	3	0.00266645	detection of hexose stimulus
GO:0034287	2	3	0.00266645	detection of monosaccharide stimulus
GO:0051594	2	3	0.00266645	detection of glucose
GO:0031667	6	493	0.00276672	response to nutrient levels
GO:0010941	10	2079	0.00299118	regulation of cell death
GO:0009966	12	3261	0.00338493	regulation of signal transduction
GO:0048660	4	127	0.00424345	regulation of smooth muscle cell proliferation
GO:0009991	6	532	0.00428967	response to extracellular stimulus
GO:0044763	25	16559	0.00468398	single-organism cellular process
GO:0010558	9	1696	0.0050464	negative regulation of macromolecule biosynthetic process
GO:0010748	2	4	0.00533035	negative regulation of plasma membrane long-chain fatty acid transport
GO:0006950	13	4134	0.00619119	response to stress
GO:0008643	4	140	0.0062512	carbohydrate transport
GO:0055088	4	140	0.0062512	lipid homeostasis
GO:0045893	9	1762	0.00689311	positive regulation of transcription, DNA-templated
GO:0045598	4	144	0.00699045	regulation of fat cell differentiation
GO:0042325	9	1770	0.00715233	regulation of phosphorylation
GO:0045944	8	1312	0.00728099	positive regulation of transcription from RNA polymerase II promoter
GO:0051716	19	9450	0.00775487	cellular response to stimulus
GO:0010677	3	44	0.00824877	negative regulation of cellular carbohydrate metabolic process
GO:0046627	3	44	0.00824877	negative regulation of insulin receptor signaling pathway
GO:1902680	9	1811	0.00861719	positive regulation of RNA biosynthetic process
GO:1900077	3	46	0.0094448	negative regulation of cellular response to insulin stimulus
GO:0051254	9	1838	0.00971729	positive regulation of RNA metabolic process
GO:0009059	13	4344	0.0107714	macromolecule biosynthetic process
GO:0042493	6	633	0.0115949	response to drug
GO:0045912	3	51	0.0129236	negative regulation of carbohydrate metabolic process
GO:0010605	10	2452	0.0130698	negative regulation of macromolecule metabolic process
GO:0046323	2	6	0.0133132	glucose import
GO:0010628	9	1919	0.0137746	positive regulation of gene expression
GO:0080090	19	9808	0.0139821	regulation of primary metabolic process
GO:1901576	15	6037	0.014309	organic substance biosynthetic process
GO:0044093	10	2479	0.014397	positive regulation of molecular function
GO:0050794	25	17465	0.0151025	regulation of cellular process

Table 4: Overrepresented GO terms with the standard enrichment

GO Term	N1	N2	P-value	Description
GO:0007259	3	56	0.0171581	JAK-STAT cascade
GO:0044723	7	1049	0.0178991	single-organism carbohydrate metabolic process
GO:2000147	5	393	0.017994	positive regulation of cell motility
GO:0006111	3	57	0.0181021	regulation of gluconeogenesis
GO:0046326	3	57	0.0181021	positive regulation of glucose import
GO:1902236	2	7	0.0186295	negative regulation of endoplasmic reticulum stress-induced intrinsic apoptotic signaling pathway
GO:0042127	9	2008	0.0198381	regulation of cell proliferation
GO:0043279	4	188	0.020038	response to alkaloid
GO:0009058	15	6217	0.0206795	biosynthetic process
GO:0051272	5	405	0.0207968	positive regulation of cellular component movement
GO:0040017	5	414	0.0231144	positive regulation of locomotion
GO:0008286	4	195	0.0231342	insulin receptor signaling pathway
GO:0051090	5	415	0.0233838	regulation of sequence-specific DNA binding transcription factor activity
GO:0051649	10	2624	0.0237254	establishment of localization in cell
GO:0065009	12	3941	0.0242628	regulation of molecular function
GO:0045935	9	2060	0.024346	positive regulation of nucleobase-containing compound metabolic process
GO:0035883	2	8	0.0248276	enteroendocrine cell differentiation
GO:2000192	2	8	0.0248276	negative regulation of fatty acid transport
GO:0010828	3	64	0.0256829	positive regulation of glucose transport
GO:0031331	4	203	0.0270889	positive regulation of cellular catabolic process
GO:0051173	9	2108	0.0292608	positive regulation of nitrogen compound metabolic process
GO:0050994	3	67	0.0294843	regulation of lipid catabolic process
GO:2000113	8	1622	0.0340869	negative regulation of cellular macromolecule biosynthetic process
GO:0030855	5	451	0.0348406	epithelial cell differentiation
GO:0045599	3	72	0.0366123	negative regulation of fat cell differentiation
GO:0046626	3	73	0.0381617	regulation of insulin receptor signaling pathway
GO:0071333	3	73	0.0381617	cellular response to glucose stimulus
GO:0045165	4	222	0.0384552	cell fate commitment
GO:0010746	2	10	0.0398635	regulation of plasma membrane long-chain fatty acid transport
GO:0051048	4	225	0.0405268	negative regulation of secretion
GO:0014074	4	233	0.046451	response to purine-containing compound

Table 5: Overrepresented GO terms with the standard enrichment

2 Network-based enrichment

GO Term	N1	N2	P-value	Description
GO:0014070	19	2783	7.75063e-11	response to organic cyclic compound
GO:0035295	12	664	3.84921e-10	tube development
GO:0032844	14	1155	4.54071e-10	regulation of homeostatic process
GO:0033993	18	2604	4.7618e-10	response to lipid
GO:0045596	17	2226	6.7533e-10	negative regulation of cell differentiation
GO:0051093	18	2690	8.31744e-10	negative regulation of developmental process
GO:0051094	19	3200	9.74075e-10	positive regulation of developmental process
GO:0045892	19	3247	1.26721e-09	negative regulation of transcription, DNA-templated
GO:0051172	20	3798	1.33706e-09	negative regulation of nitrogen compound metabolic process
GO:1902679	19	3295	1.65095e-09	negative regulation of RNA biosynthetic process
GO:1902532	14	1289	2.00831e-09	negative regulation of intracellular signal transduction
GO:0001932	19	3361	2.35949e-09	regulation of protein phosphorylation
GO:0051253	19	3369	2.46261e-09	negative regulation of RNA metabolic process
GO:0031401	18	2947	3.9591e-09	positive regulation of protein modification process
GO:0033674	15	1706	4.58206e-09	positive regulation of kinase activity
GO:0097305	13	1084	5.06875e-09	response to alcohol
GO:0006629	19	3598	8.01758e-09	lipid metabolic process
GO:0080134	18	3072	8.03038e-09	regulation of response to stress
GO:0045934	19	3691	1.26568e-08	negative regulation of nucleobase-containing compound metabolic process
GO:0042327	17	2706	1.58432e-08	positive regulation of phosphorylation
GO:0007154	17	2752	2.07642e-08	cell communication
GO:0000122	16	2314	2.25206e-08	negative regulation of transcription from RNA polymerase II promoter
GO:0032270	18	3279	2.42688e-08	positive regulation of cellular protein metabolic process
GO:0051347	15	1933	2.73645e-08	positive regulation of transferase activity
GO:0008284	17	2808	2.8676e-08	positive regulation of cell proliferation
GO:0001934	16	2371	3.25468e-08	positive regulation of protein phosphorylation
GO:0043269	14	1590	3.36973e-08	regulation of ion transport
GO:0045860	14	1636	4.93103e-08	positive regulation of protein kinase activity
GO:0060284	17	2979	7.37474e-08	regulation of cell development
GO:0045597	16	2514	7.87559e-08	positive regulation of cell differentiation
GO:0010562	17	3001	8.29232e-08	positive regulation of phosphorus metabolic process
GO:0045937	17	3001	8.29232e-08	positive regulation of phosphate metabolic process
GO:0051247	18	3557	9.57428e-08	positive regulation of protein metabolic process
GO:0010629	18	3561	9.75687e-08	negative regulation of gene expression
GO:0046890	9	401	1.11425e-07	regulation of lipid biosynthetic process
GO:0043549	16	2576	1.1362e-07	regulation of kinase activity
GO:0048731	16	2612	1.39958e-07	system development
GO:0007267	15	2185	1.55809e-07	cell-cell signaling
GO:0040011	17	3142	1.72111e-07	locomotion
GO:0023052	15	2287	2.96614e-07	signaling
GO:0044700	15	2287	2.96614e-07	single organism signaling
GO:0048545	13	1516	3.31898e-07	response to steroid hormone
GO:0006873	12	1202	3.84586e-07	cellular ion homeostasis
GO:0048870	16	2808	4.13474e-07	cell motility
GO:0051338	16	2819	4.38321e-07	regulation of transferase activity
GO:0050801	13	1555	4.54057e-07	ion homeostasis
GO:0022603	16	2832	4.69465e-07	regulation of anatomical structure morphogenesis
GO:0050767	15	2377	5.10477e-07	regulation of neurogenesis
GO:1902582	16	2881	6.06245e-07	single-organism intracellular transport
GO:0045859	15	2438	7.28407e-07	regulation of protein kinase activity
GO:0009628	17	3444	7.34525e-07	response to abiotic stimulus
GO:0008283	15	2526	1.19647e-06	cell proliferation
GO:0008202	10	760	1.38276e-06	steroid metabolic process
GO:0009888	15	2570	1.52276e-06	tissue development
GO:0016477	15	2574	1.55617e-06	cell migration
GO:0009887	13	1719	1.55644e-06	organ morphogenesis
GO:0048729	12	1359	1.56446e-06	tissue morphogenesis
GO:0051960	15	2584	1.64266e-06	regulation of nervous system development
GO:0080135	12	1388	1.98931e-06	regulation of cellular response to stress
GO:0051248	14	2189	2.3083e-06	negative regulation of protein metabolic process

Table 6: Overrepresented terms with the network-based enrichment. Only terms not detected with the standard method.

GO Term	N1	N2	P-value	Description
GO:0046907	17	3768	3.00751e-06	intracellular transport
GO:0043408	13	1837	3.50178e-06	regulation of MAPK cascade
GO:0050877	15	2735	3.6199e-06	neurological system process
GO:0044092	16	3258	3.7473e-06	negative regulation of molecular function
GO:1901615	12	1475	3.96551e-06	organic hydroxy compound metabolic process
GO:0030003	11	1151	4.42555e-06	cellular cation homeostasis
GO:0040008	14	2308	4.6027e-06	regulation of growth
GO:0008219	15	2827	5.72493e-06	cell death
GO:0016265	15	2848	6.34185e-06	death
GO:0010942	12	1542	6.55257e-06	positive regulation of cell death
GO:0009653	18	4591	6.63618e-06	anatomical structure morphogenesis
GO:0043433	8	432	7.19158e-06	negative regulation of sequence-specific DNA binding transcription factor activity
GO:0007610	13	1993	9.42199e-06	behavior
GO:0019218	7	278	9.89003e-06	regulation of steroid metabolic process
GO:0048646	15	2968	1.12066e-05	anatomical structure formation involved in morphogenesis
GO:0010638	11	1260	1.13728e-05	positive regulation of organelle organization
GO:0003002	10	988	1.71299e-05	regionalization
GO:0001822	8	489	1.89121e-05	kidney development
GO:0050678	10	1020	2.31971e-05	regulation of epithelial cell proliferation
GO:0006875	10	1046	2.94599e-05	cellular metal ion homeostasis
GO:0009607	13	2197	3.04912e-05	response to biotic stimulus
GO:0055080	11	1386	3.05437e-05	cation homeostasis
GO:0010647	16	3801	3.56004e-05	positive regulation of cell communication
GO:0007169	12	1794	3.58181e-05	transmembrane receptor protein tyrosine kinase signaling pathway
GO:0050679	8	531	3.58661e-05	positive regulation of epithelial cell proliferation
GO:0044087	12	1812	4.00345e-05	regulation of cellular component biogenesis
GO:0001667	8	542	4.20421e-05	ameboidal cell migration
GO:0006811	16	3852	4.31702e-05	ion transport
GO:0048468	13	2280	4.75388e-05	cell development
GO:0051130	14	2782	5.11649e-05	positive regulation of cellular component organization
GO:0044708	11	1465	5.40849e-05	single-organism behavior
GO:0007389	11	1481	6.04752e-05	pattern specification process
GO:0072507	9	827	6.24008e-05	divalent inorganic cation homeostasis
GO:0003006	13	2335	6.31912e-05	developmental process involved in reproduction
GO:0045664	12	1894	6.54934e-05	regulation of neuron differentiation
GO:2000027	8	574	6.55195e-05	regulation of organ morphogenesis
GO:0040007	11	1496	6.7071e-05	growth
GO:0008285	13	2354	6.96008e-05	negative regulation of cell proliferation
GO:0071407	10	1148	7.10289e-05	cellular response to organic cyclic compound
GO:0051241	11	1513	7.53185e-05	negative regulation of multicellular organismal process
GO:0007568	9	848	7.73846e-05	aging
GO:0002009	10	1159	7.77084e-05	morphogenesis of an epithelium
GO:0030334	12	1926	7.88663e-05	regulation of cell migration
GO:0001101	10	1170	8.49388e-05	response to acid chemical
GO:0010721	8	609	0.000103424	negative regulation of cell development
GO:0051345	14	2953	0.000109334	positive regulation of hydrolase activity
GO:0045787	7	401	0.000122471	positive regulation of cell cycle
GO:0050890	9	896	0.000123987	cognition
GO:0071902	9	897	0.000125175	positive regulation of protein serine/threonine kinase activity
GO:0055065	10	1233	0.000139042	metal ion homeostasis
GO:0007167	13	2504	0.000144967	enzyme linked receptor protein signaling pathway
GO:0030030	13	2507	0.000147036	cell projection organization
GO:2000145	12	2039	0.000148093	regulation of cell motility
GO:0002521	9	929	0.000168837	leukocyte differentiation
GO:0006886	12	2070	0.000174865	intracellular protein transport
GO:0043410	10	1269	0.000182069	positive regulation of MAPK cascade
GO:0009611	8	657	0.000185287	response to wounding
GO:2001233	10	1313	0.000250365	regulation of apoptotic signaling pathway
GO:0001708	6	264	0.000270646	cell fate specification
GO:0006812	13	2652	0.000285466	cation transport

Table 7: Overrepresented terms with the network-based enrichment. Only terms not detected with the standard method.

GO Term	N1	N2	P-value	Description
GO:0051270	12	2182	0.000311774	regulation of cellular component movement
GO:0051240	12	2185	0.000316497	positive regulation of multicellular organismal process
GO:0060429	9	1006	0.000332313	epithelium development
GO:0014013	6	276	0.000351742	regulation of gliogenesis
GO:0043436	14	3244	0.000357795	oxoacid metabolic process
GO:0031400	10	1368	0.000366953	negative regulation of protein modification process
GO:0001817	11	1774	0.000381104	regulation of cytokine production
GO:0040012	12	2224	0.000384024	regulation of locomotion
GO:0030335	9	1024	0.000386206	positive regulation of cell migration
GO:0048568	7	476	0.000392572	embryonic organ development
GO:0006082	14	3284	0.000417175	organic acid metabolic process
GO:0012501	12	2247	0.000429665	programmed cell death
GO:0032269	11	1804	0.000451478	negative regulation of cellular protein metabolic process
GO:0033043	13	2759	0.000454057	regulation of organelle organization
GO:0051726	13	2760	0.000455987	regulation of cell cycle
GO:0002684	12	2287	0.000520762	positive regulation of immune system process
GO:0048608	9	1062	0.000525615	reproductive structure development
GO:0050731	7	503	0.000569967	positive regulation of peptidyl-tyrosine phosphorylation
GO:0044255	13	2817	0.000579068	cellular lipid metabolic process
GO:0072503	8	772	0.000634812	cellular divalent inorganic cation homeostasis
GO:0017038	6	307	0.000657675	protein import
GO:0001838	4	62	0.000707635	embryonic epithelial tube formation
GO:0007417	7	521	0.000722455	central nervous system development
GO:0072175	4	63	0.000755158	epithelial tube formation
GO:0055074	8	790	0.000756196	calcium ion homeostasis
GO:0051129	11	1913	0.000814923	negative regulation of cellular component organization
GO:0071900	10	1492	0.000819895	regulation of protein serine/threonine kinase activity
GO:0015031	14	3506	0.00094269	protein transport
GO:1902533	12	2418	0.000953114	positive regulation of intracellular signal transduction
GO:0090066	8	820	0.00100294	regulation of anatomical structure size
GO:0051098	8	826	0.00105981	regulation of binding
GO:0070328	4	69	0.00109203	triglyceride homeostasis
GO:0032147	8	832	0.00111943	activation of protein kinase activity
GO:0010959	8	835	0.00115031	regulation of metal ion transport
GO:0055090	4	70	0.00115752	acylglycerol homeostasis
GO:0055114	14	3568	0.00117135	oxidation-reduction process
GO:0051384	7	564	0.00123126	response to glucocorticoid
GO:0048598	10	1566	0.00128038	embryonic morphogenesis
GO:0032496	8	847	0.00128123	response to lipopolysaccharide
GO:0034097	11	2017	0.00138417	response to cytokine
GO:0009967	14	3621	0.00140551	positive regulation of signal transduction
GO:0007268	9	1195	0.00141747	synaptic transmission
GO:0055085	14	3627	0.00143453	transmembrane transport
GO:0071396	9	1198	0.00144752	cellular response to lipid
GO:0032787	10	1592	0.00148931	monocarboxylic acid metabolic process
GO:0048565	5	185	0.00151286	digestive tract development
GO:0006955	13	3063	0.00153068	immune response
GO:0010564	10	1606	0.00161378	regulation of cell cycle process
GO:0044702	14	3664	0.00162582	single organism reproductive process
GO:0044711	14	3675	0.00168698	single-organism biosynthetic process
GO:0043207	11	2065	0.00174971	response to external biotic stimulus
GO:0001775	11	2070	0.00179232	cell activation
GO:0071322	5	192	0.00181756	cellular response to carbohydrate stimulus
GO:0031344	10	1640	0.00195492	regulation of cell projection organization
GO:0048638	7	605	0.00196966	regulation of developmental growth
GO:0031346	8	897	0.00197388	positive regulation of cell projection organization
GO:0045184	14	3728	0.00201198	establishment of protein localization
GO:0016337	8	900	0.00202406	single organismal cell-cell adhesion
GO:0031960	7	610	0.00208099	response to corticosteroid
GO:0035150	5	198	0.00211562	regulation of tube size

Table 8: Overrepresented terms with the network-based enrichment. Only terms not detected with the standard method.

GO Term	N1	N2	P-value	Description
GO:0050810	5	198	0.00211562	regulation of steroid biosynthetic process
GO:0016051	6	378	0.00221729	carbohydrate biosynthetic process
GO:0002237	8	918	0.00234871	response to molecule of bacterial origin
GO:0061138	7	624	0.00242124	morphogenesis of a branching epithelium
GO:0038093	7	627	0.00249993	Fc receptor signaling pathway
GO:0010975	9	1281	0.00253099	regulation of neuron projection development
GO:0050795	7	630	0.00258076	regulation of behavior
GO:0043405	8	930	0.0025891	regulation of MAP kinase activity
GO:0001938	5	207	0.00263378	positive regulation of endothelial cell proliferation
GO:0006915	11	2154	0.0026599	apoptotic process
GO:0034765	8	934	0.00267378	regulation of ion transmembrane transport
GO:0035148	6	396	0.00290523	tube formation
GO:0043627	7	647	0.0030815	response to estrogen
GO:0016042	7	650	0.00317782	lipid catabolic process
GO:0023014	7	653	0.00327667	signal transduction by phosphorylation
GO:0032880	11	2200	0.003278	regulation of protein localization
GO:0001933	8	963	0.00336172	negative regulation of protein phosphorylation
GO:0002768	8	967	0.00346755	immune response-regulating cell surface receptor signaling pathway
GO:2001243	5	219	0.00347519	negative regulation of intrinsic apoptotic signaling pathway
GO:0050730	7	663	0.00362501	regulation of peptidyl-tyrosine phosphorylation
GO:2001234	7	666	0.00373538	negative regulation of apoptotic signaling pathway
GO:0010720	8	977	0.00374471	positive regulation of cell development
GO:0098602	8	981	0.00386073	single organism cell adhesion
GO:0051091	7	677	0.00416451	positive regulation of sequence-specific DNA binding transcription factor activity
GO:0048534	7	682	0.00437285	hematopoietic or lymphoid organ development
GO:0034762	8	998	0.00438891	regulation of transmembrane transport
GO:0042594	6	427	0.00449539	response to starvation
GO:0001763	7	690	0.00472433	morphogenesis of a branching structure
GO:0007155	12	2817	0.00489243	cell adhesion
GO:0006970	5	235	0.00491229	response to osmotic stress
GO:0048732	8	1014	0.00494124	gland development
GO:0001558	9	1390	0.00497862	regulation of cell growth
GO:0016482	10	1819	0.00501452	cytoplasmic transport
GO:0022610	12	2824	0.00502278	biological adhesion
GO:2000648	5	237	0.00512063	positive regulation of stem cell proliferation
GO:0009894	13	3408	0.00519748	regulation of catabolic process
GO:0010867	3	28	0.00543298	positive regulation of triglyceride biosynthetic process
GO:0045821	3	28	0.00543298	positive regulation of glycolytic process
GO:0003014	5	240	0.00544609	renal system process
GO:0043065	9	1406	0.00547144	positive regulation of apoptotic process
GO:0050878	10	1837	0.00548111	regulation of body fluid levels
GO:0003013	6	444	0.00563238	circulatory system process
GO:0043068	9	1416	0.00580045	positive regulation of programmed cell death
GO:0050920	6	450	0.00608575	regulation of chemotaxis
GO:0018105	5	247	0.00626909	peptidyl-serine phosphorylation
GO:0090068	7	724	0.00649462	positive regulation of cell cycle process
GO:0032526	6	457	0.00665196	response to retinoic acid
GO:0002694	9	1443	0.00677578	regulation of leukocyte activation
GO:0043524	6	459	0.00682139	negative regulation of neuron apoptotic process
GO:0006366	9	1448	0.00697118	transcription from RNA polymerase II promoter
GO:0050776	11	2377	0.00701162	regulation of immune response
GO:0031532	4	110	0.00711529	actin cytoskeleton reorganization
GO:0071496	7	736	0.00723909	cellular response to external stimulus
GO:2001273	3	31	0.00744155	regulation of glucose import in response to insulin stimulus
GO:2000021	6	471	0.00791376	regulation of ion homeostasis
GO:0002053	4	113	0.00792128	positive regulation of mesenchymal cell proliferation
GO:0006874	7	749	0.008125	cellular calcium ion homeostasis
GO:0048806	4	114	0.0082044	genitalia development
GO:0055081	4	115	0.00849496	anion homeostasis
GO:0045927	7	757	0.00871399	positive regulation of growth

Table 9: Overrepresented terms with the network-based enrichment. Only terms not detected with the standard method.

GO Term	N1	N2	P-value	Description
GO:0031668	6	479	0.00871831	cellular response to extracellular stimulus
GO:0019752	12	2978	0.00879547	carboxylic acid metabolic process
GO:0044282	7	760	0.00894387	small molecule catabolic process
GO:0048562	6	484	0.00925421	embryonic organ morphogenesis
GO:0032846	5	270	0.00968371	positive regulation of homeostatic process
GO:0006979	8	1117	0.0101266	response to oxidative stress
GO:0016310	14	4277	0.010678	phosphorylation
GO:0002792	4	122	0.0107475	negative regulation of peptide secretion
GO:0090278	4	122	0.0107475	negative regulation of peptide hormone secretion
GO:0070848	10	1987	0.0110981	response to growth factor
GO:0032409	6	500	0.0111527	regulation of transporter activity
GO:0007420	7	791	0.011632	brain development
GO:0010866	3	36	0.0117858	regulation of triglyceride biosynthetic process
GO:0007611	7	793	0.0118263	learning or memory
GO:0008203	5	282	0.011968	cholesterol metabolic process
GO:0051302	7	796	0.012123	regulation of cell division
GO:0010893	3	37	0.0128183	positive regulation of steroid biosynthetic process
GO:0044262	6	514	0.0130639	cellular carbohydrate metabolic process
GO:0031329	12	3094	0.0131256	regulation of cellular catabolic process
GO:0050865	9	1568	0.0133731	regulation of cell activation
GO:0018209	5	289	0.0134838	peptidyl-serine modification
GO:0042326	8	1164	0.0137216	negative regulation of phosphorylation
GO:1901215	6	524	0.014586	negative regulation of neuron death
GO:0010464	4	132	0.0146962	regulation of mesenchymal cell proliferation
GO:0046649	8	1196	0.0167471	lymphocyte activation
GO:0000165	6	537	0.0167777	MAPK cascade
GO:0001936	5	306	0.0177963	regulation of endothelial cell proliferation
GO:0033273	5	308	0.0183674	response to vitamin
GO:0044246	4	140	0.018556	regulation of multicellular organismal metabolic process
GO:0009755	5	309	0.0186582	hormone-mediated signaling pathway
GO:0051924	6	553	0.0198356	regulation of calcium ion transport
GO:0090287	6	557	0.0206669	regulation of cellular response to growth factor stimulus
GO:0016125	5	319	0.0217702	sterol metabolic process
GO:0007411	8	1242	0.0220786	axon guidance
GO:0097485	8	1243	0.0222089	neuron projection guidance
GO:0002764	8	1248	0.02287	immune response-regulating signaling pathway
GO:0050921	5	324	0.0234713	positive regulation of chemotaxis
GO:0015850	5	327	0.0245409	organic hydroxy compound transport
GO:0072091	5	327	0.0245409	regulation of stem cell proliferation
GO:0051348	7	887	0.0245862	negative regulation of transferase activity
GO:0051249	8	1264	0.0250991	regulation of lymphocyte activation
GO:0045926	7	891	0.0253161	negative regulation of growth
GO:0046470	4	152	0.0256868	phosphatidylcholine metabolic process
GO:0006694	5	331	0.026026	steroid biosynthetic process
GO:0051099	5	331	0.026026	positive regulation of binding
GO:0045995	5	334	0.0271853	regulation of embryonic development
GO:0009952	6	585	0.0273142	anterior/posterior pattern specification
GO:0051052	7	905	0.0280146	regulation of DNA metabolic process
GO:0032330	4	157	0.0291866	regulation of chondrocyte differentiation
GO:0046889	4	157	0.0291866	positive regulation of lipid biosynthetic process
GO:0030182	7	911	0.0292422	neuron differentiation
GO:0015758	4	158	0.0299267	glucose transport
GO:0018193	10	2224	0.030173	peptidyl-amino acid modification
GO:0032966	2	6	0.0310781	negative regulation of collagen biosynthetic process
GO:0071326	4	160	0.0314484	cellular response to monosaccharide stimulus
GO:0050995	3	51	0.034075	negative regulation of lipid catabolic process
GO:0045666	5	351	0.0345404	positive regulation of neuron differentiation
GO:0008645	4	167	0.0372263	hexose transport
GO:0051493	8	1336	0.0375558	regulation of cytoskeleton organization
GO:0006631	7	949	0.0381027	fatty acid metabolic process

Table 10: Overrepresented terms with the network-based enrichment. Only terms not detected with the standard method.

GO Term	N1	N2	P-value	Description
GO:0070663	6	621	0.038311	regulation of leukocyte proliferation
GO:0010038	7	951	0.0386249	response to metal ion
GO:0015749	4	169	0.039012	monosaccharide transport
GO:0006869	6	627	0.0404506	lipid transport
GO:0010563	8	1353	0.0411598	negative regulation of phosphorus metabolic process
GO:0045936	8	1353	0.0411598	negative regulation of phosphate metabolic process
GO:0015711	7	964	0.0421653	organic anion transport
GO:0072171	2	7	0.0434858	mesonephric tubule morphogenesis
GO:0072180	2	7	0.0434858	mesonephric duct morphogenesis
GO:1901074	2	7	0.0434858	regulation of engulfment of apoptotic cell
GO:0007596	8	1367	0.0443438	blood coagulation
GO:0050817	8	1367	0.0443438	coagulation
GO:0002064	5	370	0.0445115	epithelial cell development
GO:0007507	6	638	0.0446241	heart development
GO:0007599	8	1382	0.0479838	hemostasis
GO:0010608	8	1385	0.0487412	posttranscriptional regulation of gene expression
GO:0006066	7	986	0.0487699	alcohol metabolic process
GO:0001947	4	179	0.0489021	heart looping

Table 11: Overrepresented terms with the network-based enrichment. Only terms not detected with the standard method.
