# Supplementary material for: NET-GE: a novel NETwork-based Gene Enrichment for detecting biological processes associated to Mendelian diseases
Source: BMC Genomics. 2015 Jun 18;16(Suppl 8):S6. doi: 10.1186/1471-2164-16-S8-S6 (PMC4480278; doi:10.1186/1471-2164-16-S8-S6)
Supplement: Additional file 3 — Detailed results for the OMIM-derived benchmark set. The archive contains pdf documents listing the enriched terms for each one of the 244 diseases in the OMIM-derived benchmark set. [file 1471-2164-16-S8-S6-S3.tgz › SUPPMAT/OMIM127750.pdf]

#127750 DEMENTIA, LEWY BODY; DLB

OMIM Gene ID	HGNC	UniProtAC
163890	SNCA	P37840
602569	SNCB	Q16143
606463	GBA	P04062

Table 1: OMIM - UniProtAC mapping

Legend

- N1: #input proteins associated to the significant GO term
- N2: #proteins associated to the significant GO term
- P-value: Bonferroni-corrected p-value of Fisher's exact test
- *red*: go terms not related to the input proteins
- *blue*: go terms related to the input proteins (enriched uniquely by network-based method)
- *green*: go terms ancestors of terms enriched with the standard method (enriched uniquely by network-based method)

1 Standard enrichment

GO Term	N1	N2	P-value	Description
GO:0042417	2	35	0.00132999	dopamine metabolic process
GO:1901615	3	621	0.00235393	organic hydroxy compound metabolic process
GO:0006584	2	53	0.00307923	catecholamine metabolic process
GO:0009712	2	53	0.00307923	catechol-containing compound metabolic process
GO:0043086	3	1047	0.0113036	negative regulation of catalytic activity
GO:0018958	2	128	0.0181385	phenol-containing compound metabolic process
GO:0044092	3	1284	0.0208593	negative regulation of molecular function
GO:0050808	2	184	0.037534	synapse organization
GO:0051585	1	1	0.0422065	negative regulation of dopamine uptake involved in synaptic transmission
GO:0051621	1	1	0.0422065	regulation of norepinephrine uptake
GO:0051622	1	1	0.0422065	negative regulation of norepinephrine uptake
GO:0051945	1	1	0.0422065	negative regulation of catecholamine uptake involved in synaptic transmission
GO:0070494	1	1	0.0422065	regulation of thrombin receptor signaling pathway
GO:0070495	1	1	0.0422065	negative regulation of thrombin receptor signaling pathway
GO:1903282	1	1	0.0422065	regulation of glutathione peroxidase activity
GO:1903284	1	1	0.0422065	positive regulation of glutathione peroxidase activity
GO:1903285	1	1	0.0422065	positive regulation of hydrogen peroxide catabolic process
GO:2000470	1	1	0.0422065	positive regulation of peroxidase activity
GO:1901617	2	203	0.0456938	organic hydroxy compound biosynthetic process

Table 2: Overrepresented GO terms with the standard enrichment

2 Network-based enrichment

No novel enriched terms
