# Supplementary material for: NET-GE: a novel NETwork-based Gene Enrichment for detecting biological processes associated to Mendelian diseases
Source: BMC Genomics. 2015 Jun 18;16(Suppl 8):S6. doi: 10.1186/1471-2164-16-S8-S6 (PMC4480278; doi:10.1186/1471-2164-16-S8-S6)
Supplement: Additional file 3 — Detailed results for the OMIM-derived benchmark set. The archive contains pdf documents listing the enriched terms for each one of the 244 diseases in the OMIM-derived benchmark set. [file 1471-2164-16-S8-S6-S3.tgz › SUPPMAT/OMIM130000.pdf]

#130000 EHLERS-DANLOS SYNDROME, TYPE I

OMIM Gene ID	HGNC	UniProtAC
120150	COL1A1	P02452
120190	COL5A2	P05997
120215	COL5A1	P20908

Table 1: OMIM - UniProtAC mapping

Legend

- N1: #input proteins associated to the significant GO term
- N2: #proteins associated to the significant GO term
- P-value: Bonferroni-corrected p-value of Fisher's exact test
- *red*: go terms not related to the input proteins
- *blue*: go terms related to the input proteins (enriched uniquely by network-based method)
- *green*: go terms ancestors of terms enriched with the standard method (enriched uniquely by network-based method)

1 Standard enrichment

GO Term	N1	N2	P-value	Description
GO:1903225	2	2	1.03616e-06	negative regulation of endodermal cell differentiation
GO:0030199	3	76	1.93004e-06	collagen fibril organization
GO:0030574	3	78	2.08862e-06	collagen catabolic process
GO:0044243	3	84	2.61596e-06	multicellular organismal catabolic process
GO:0032963	3	96	3.92269e-06	collagen metabolic process
GO:0044259	3	105	5.14659e-06	multicellular organismal macromolecule metabolic process
GO:0044236	3	112	6.2574e-06	multicellular organismal metabolic process
GO:0022617	3	117	7.1417e-06	extracellular matrix disassembly
GO:1903224	2	6	1.55412e-05	regulation of endodermal cell differentiation
GO:0032964	2	14	9.42702e-05	collagen biosynthetic process
GO:0022411	3	404	0.000299483	cellular component disassembly
GO:0030198	3	486	0.000522017	extracellular matrix organization
GO:0043062	3	487	0.000525252	extracellular structure organization
GO:0010470	2	43	0.000934972	regulation of gastrulation
GO:0043588	2	45	0.00102502	skin development
GO:0009887	3	653	0.00126824	organ morphogenesis
GO:0048592	2	72	0.00264514	eye morphogenesis
GO:0071230	2	77	0.00302777	cellular response to amino acid stimulus
GO:0022603	3	1116	0.00634286	regulation of anatomical structure morphogenesis
GO:0001568	2	137	0.00962982	blood vessel development
GO:0045995	2	139	0.00991373	regulation of embryonic development
GO:0043200	2	157	0.012654	response to amino acid
GO:0045112	1	1	0.0195533	integrin biosynthetic process
GO:0001501	2	213	0.0233071	skeletal system development
GO:0071229	2	233	0.027891	cellular response to acid chemical
GO:0048513	3	1910	0.0318331	organ development
GO:0071822	3	1933	0.0329975	protein complex subunit organization
GO:0006928	3	1973	0.0350897	cellular component movement
GO:0044712	3	2063	0.0401167	single-organism catabolic process
GO:0045595	3	2111	0.0429841	regulation of cell differentiation
GO:0009653	3	2131	0.044218	anatomical structure morphogenesis
GO:0043933	3	2217	0.0497931	macromolecular complex subunit organization

Table 2: Overrepresented GO terms with the standard enrichment

2 Network-based enrichment

GO Term	N1	N2	P-value	Description
GO:0048593	2	47	0.00284347	camera-type eye morphogenesis
GO:0007411	3	1242	0.0251747	axon guidance
GO:0097485	3	1243	0.0252356	neuron projection guidance

Table 3: Overrepresented terms with the network-based enrichment. Only terms not detected with the standard method.
