# Supplementary material for: NET-GE: a novel NETwork-based Gene Enrichment for detecting biological processes associated to Mendelian diseases
Source: BMC Genomics. 2015 Jun 18;16(Suppl 8):S6. doi: 10.1186/1471-2164-16-S8-S6 (PMC4480278; doi:10.1186/1471-2164-16-S8-S6)
Supplement: Additional file 3 — Detailed results for the OMIM-derived benchmark set. The archive contains pdf documents listing the enriched terms for each one of the 244 diseases in the OMIM-derived benchmark set. [file 1471-2164-16-S8-S6-S3.tgz › SUPPMAT/OMIM130020.pdf]

#130020 EHLERS-DANLOS SYNDROME, TYPE III

OMIM Gene ID	HGNC	UniProtAC
120180	COL3A1	P02461
600985	TNXB	P22105

Table 1: OMIM - UniProtAC mapping

Legend

- N1: #input proteins associated to the significant GO term
- N2: #proteins associated to the significant GO term
- P-value: Bonferroni-corrected p-value of Fisher's exact test
- *red*: go terms not related to the input proteins
- *blue*: go terms related to the input proteins (enriched uniquely by network-based method)
- *green*: go terms ancestors of terms enriched with the standard method (enriched uniquely by network-based method)

1 Standard enrichment

GO Term	N1	N2	P-value	Description
GO:0043206	2	15	2.77151e-05	extracellular fibril organization
GO:0097435	2	17	3.58977e-05	fibril organization
GO:0030199	2	76	0.000752265	collagen fibril organization
GO:0032963	2	96	0.00120362	collagen metabolic process
GO:0044259	2	105	0.00144118	multicellular organismal macromolecule metabolic process
GO:0044236	2	112	0.00164073	multicellular organismal metabolic process
GO:0007160	2	228	0.00683057	cell-matrix adhesion
GO:0031589	2	292	0.0112143	cell-substrate adhesion
GO:0030198	2	486	0.0311082	extracellular matrix organization
GO:0043062	2	487	0.0312364	extracellular structure organization

Table 2: Overrepresented GO terms with the standard enrichment

2 Network-based enrichment

No novel enriched terms
