# Supplementary material for: NET-GE: a novel NETwork-based Gene Enrichment for detecting biological processes associated to Mendelian diseases
Source: BMC Genomics. 2015 Jun 18;16(Suppl 8):S6. doi: 10.1186/1471-2164-16-S8-S6 (PMC4480278; doi:10.1186/1471-2164-16-S8-S6)
Supplement: Additional file 3 — Detailed results for the OMIM-derived benchmark set. The archive contains pdf documents listing the enriched terms for each one of the 244 diseases in the OMIM-derived benchmark set. [file 1471-2164-16-S8-S6-S3.tgz › SUPPMAT/OMIM130060-OMIM166210-OMIM166220-OMIM259420.pdf]

#130060 EHLERS-DANLOS SYNDROME, TYPE VII, AUTOSOMAL DOMINANT
#166210 OSTEOGENESIS IMPERFECTA, TYPE II
#259420 OSTEOGENESIS IMPERFECTA, TYPE III
#166220 OSTEOGENESIS IMPERFECTA, TYPE IV

**OMIM Gene ID**	**HGNC**	**UniProtAC**
120150	COL1A1	P02452
120160	COL1A2	P08123

Table 1: OMIM - UniProtAC mapping

Legend

- N1: #input proteins associated to the significant GO term
- N2: #proteins associated to the significant GO term
- P-value: Bonferroni-corrected p-value of Fisher's exact test
- *red*: go terms not related to the input proteins
- *blue*: go terms related to the input proteins (enriched uniquely by network-based method)
- *green*: go terms ancestors of terms enriched with the standard method (enriched uniquely by network-based method)

1 Standard enrichment

GO Term	N1	N2	P-value	Description
GO:0070208	2	13	2.59543e-05	protein heterotrimerization
GO:0043589	2	15	3.49388e-05	skin morphogenesis
GO:0070206	2	40	0.000259543	protein trimerization
GO:0030199	2	76	0.000948334	collagen fibril organization
GO:0071230	2	77	0.000973624	cellular response to amino acid stimulus
GO:0030574	2	78	0.000999247	collagen catabolic process
GO:0044243	2	84	0.00115996	multicellular organismal catabolic process
GO:0032963	2	96	0.00151734	collagen metabolic process
GO:0044259	2	105	0.00181681	multicellular organismal macromolecule metabolic process
GO:0044236	2	112	0.00206837	multicellular organismal metabolic process
GO:0022617	2	117	0.00225804	extracellular matrix disassembly
GO:0001568	2	137	0.00309989	blood vessel development
GO:0043200	2	157	0.00407484	response to amino acid
GO:0051291	2	177	0.00518291	protein heterooligomerization
GO:0001501	2	213	0.00751281	skeletal system development
GO:0030168	2	216	0.00772644	platelet activation
GO:0071229	2	233	0.00899353	cellular response to acid chemical
GO:0071560	2	238	0.00938454	cellular response to transforming growth factor beta stimulus
GO:0071559	2	248	0.0101915	response to transforming growth factor beta
GO:0050900	2	265	0.0116396	leukocyte migration
GO:0022411	2	404	0.0270877	cellular component disassembly
GO:0001101	2	456	0.0345193	response to acid chemical
GO:0030198	2	486	0.0392162	extracellular matrix organization
GO:0043062	2	487	0.0393778	extracellular structure organization
GO:0007596	2	501	0.0416769	blood coagulation
GO:0050817	2	501	0.0416769	coagulation
GO:0007599	2	510	0.0431892	hemostasis

Table 2: Overrepresented GO terms with the standard enrichment

2 Network-based enrichment

GO Term	N1	N2	P-value	Description
GO:0048593	2	47	0.00108698	camera-type eye morphogenesis
GO:0008585	2	53	0.00138563	female gonad development
GO:0043588	2	99	0.00487786	skin development
GO:0002062	2	133	0.00882661	chondrocyte differentiation
GO:0010812	2	141	0.00992464	negative regulation of cell-substrate adhesion
GO:0048592	2	147	0.0107904	eye morphogenesis
GO:0060348	2	155	0.0120011	bone development
GO:0010770	2	177	0.0156622	positive regulation of cell morphogenesis involved in differentiation
GO:0090263	2	197	0.0194129	positive regulation of canonical Wnt signaling pathway
GO:0007519	2	214	0.0229172	skeletal muscle tissue development
GO:0016525	2	262	0.0343803	negative regulation of angiogenesis
GO:0050673	2	275	0.0378836	epithelial cell proliferation

Table 3: Overrepresented terms with the network-based enrichment. Only terms not detected with the standard method.
