# Supplementary material for: NET-GE: a novel NETwork-based Gene Enrichment for detecting biological processes associated to Mendelian diseases
Source: BMC Genomics. 2015 Jun 18;16(Suppl 8):S6. doi: 10.1186/1471-2164-16-S8-S6 (PMC4480278; doi:10.1186/1471-2164-16-S8-S6)
Supplement: Additional file 3 — Detailed results for the OMIM-derived benchmark set. The archive contains pdf documents listing the enriched terms for each one of the 244 diseases in the OMIM-derived benchmark set. [file 1471-2164-16-S8-S6-S3.tgz › SUPPMAT/OMIM131760-OMIM131900-OMIM601001.pdf]

#131760 EPIDERMOLYSIS BULLOSA SIMPLEX, DOWLING-MEARA TYPE
#131900 EPIDERMOLYSIS BULLOSA SIMPLEX, GENERALIZED
#601001 EPIDERMOLYSIS BULLOSA SIMPLEX, AUTOSOMAL RECESSIVE 1; EBSB1

**OMIM Gene ID**	**HGNC**	**UniProtAC**
148040	KRT5	P13647
148066	KRT14	P02533

Table 1: OMIM - UniProtAC mapping

Legend

- N1: #input proteins associated to the significant GO term
- N2: #proteins associated to the significant GO term
- P-value: Bonferroni-corrected p-value of Fisher's exact test
- *red*: go terms not related to the input proteins
- *blue*: go terms related to the input proteins (enriched uniquely by network-based method)
- *green*: go terms ancestors of terms enriched with the standard method (enriched uniquely by network-based method)

1 Standard enrichment

GO Term	N1	N2	P-value	Description
GO:0031581	2	14	5.49385e-06	hemidesmosome assembly
GO:0007044	2	63	0.000117907	cell-substrate junction assembly
GO:0008544	2	151	0.000683717	epidermis development
GO:0034329	2	254	0.00193982	cell junction assembly
GO:0034330	2	300	0.00270769	cell junction organization
GO:0060429	2	368	0.00407682	epithelium development
GO:0045110	1	6	0.0136705	intermediate filament bundle assembly
GO:0009888	2	984	0.0291982	tissue development
GO:0042303	1	16	0.0364499	molting cycle
GO:0042633	1	16	0.0364499	hair cycle
GO:0045109	1	19	0.0432825	intermediate filament organization

Table 2: Overrepresented GO terms with the standard enrichment

2 Network-based enrichment

No novel enriched terms
