# Supplementary material for: NET-GE: a novel NETwork-based Gene Enrichment for detecting biological processes associated to Mendelian diseases
Source: BMC Genomics. 2015 Jun 18;16(Suppl 8):S6. doi: 10.1186/1471-2164-16-S8-S6 (PMC4480278; doi:10.1186/1471-2164-16-S8-S6)
Supplement: Additional file 3 — Detailed results for the OMIM-derived benchmark set. The archive contains pdf documents listing the enriched terms for each one of the 244 diseases in the OMIM-derived benchmark set. [file 1471-2164-16-S8-S6-S3.tgz › SUPPMAT/OMIM131800.pdf]

#131800 EPIDERMOLYSIS BULLOSA SIMPLEX, LOCALIZED

**OMIM Gene ID**	**HGNC**	**UniProtAC**
147557	ITGB4	P16144
148040	KRT5	P13647
148066	KRT14	P02533

Table 1: OMIM - UniProtAC mapping

Legend

- N1: #input proteins associated to the significant GO term
- N2: #proteins associated to the significant GO term
- P-value: Bonferroni-corrected p-value of Fisher's exact test
- *red*: go terms not related to the input proteins
- *blue*: go terms related to the input proteins (enriched uniquely by network-based method)
- *green*: go terms ancestors of terms enriched with the standard method (enriched uniquely by network-based method)

1 Standard enrichment

GO Term	N1	N2	P-value	Description
GO:0031581	3	14	3.33114e-09	hemidesmosome assembly
GO:0007044	3	63	3.63413e-07	cell-substrate junction assembly
GO:0034329	3	254	2.46998e-05	cell junction assembly
GO:0034330	3	300	4.07706e-05	cell junction organization
GO:0008544	2	151	0.00390119	epidermis development
GO:0060429	2	368	0.0231724	epithelium development
GO:0022607	3	2496	0.0236892	cellular component assembly
GO:0035878	1	6	0.0391014	nail development
GO:0045110	1	6	0.0391014	intermediate filament bundle assembly

Table 2: Overrepresented GO terms with the standard enrichment

2 Network-based enrichment

GO Term	N1	N2	P-value	Description
GO:0050891	2	87	0.00452252	multicellular organismal water homeostasis
GO:0030104	2	114	0.00778236	water homeostasis
GO:0030148	2	144	0.0124327	sphingolipid biosynthetic process
GO:0006672	2	149	0.0133129	ceramide metabolic process
GO:0019233	2	169	0.0171334	sensory perception of pain
GO:0046467	2	222	0.0295754	membrane lipid biosynthetic process

Table 3: Overrepresented terms with the network-based enrichment. Only terms not detected with the standard method.
