# Supplementary material for: NET-GE: a novel NETwork-based Gene Enrichment for detecting biological processes associated to Mendelian diseases
Source: BMC Genomics. 2015 Jun 18;16(Suppl 8):S6. doi: 10.1186/1471-2164-16-S8-S6 (PMC4480278; doi:10.1186/1471-2164-16-S8-S6)
Supplement: Additional file 3 — Detailed results for the OMIM-derived benchmark set. The archive contains pdf documents listing the enriched terms for each one of the 244 diseases in the OMIM-derived benchmark set. [file 1471-2164-16-S8-S6-S3.tgz › SUPPMAT/OMIM133239.pdf]

# #133239 ESOPHAGEAL CANCER

| **OMIM Gene ID** | **HGNC** | **UniProtAC** |
|---|---|---|
| 120470 | DCC | P43146 |
| 190182 | TGFBR2 | P37173 |
| 604050 | DLEC1 | Q9Y238 |
| 604242 | RNF6 | Q9Y252 |
| 604767 | DEC1 | Q9P2X7 |
| 605131 | WWOX | Q9NZC7 |
| 606551 | LZTS1 | Q9Y250 |

Table 1: OMIM - UniProtAC mapping

Legend

- N1: #input proteins associated to the significant GO term
- N2: #proteins associated to the significant GO term
- P-value: Bonferroni-corrected p-value of Fisher's exact test
- *red*: go terms not related to the input proteins
- *blue*: go terms related to the input proteins (enriched uniquely by network-based method)
- *green*: go terms ancestors of terms enriched with the standard method (enriched uniquely by network-based method)

# 1 Standard enrichment

| GO Term | N1 | N2 | P-value | Description |
|---|---|---|---|---|
| GO:0048523 | 7 | 5279 | 0.000483173 | negative regulation of cellular process |
| GO:0048519 | 7 | 5756 | 0.00088558 | negative regulation of biological process |
| GO:0048638 | 3 | 207 | 0.00259246 | regulation of developmental growth |
| GO:0022603 | 4 | 1116 | 0.0114721 | regulation of anatomical structure morphogenesis |
| GO:0048640 | 2 | 44 | 0.0128662 | negative regulation of developmental growth |
| GO:0010769 | 3 | 381 | 0.0160485 | regulation of cell morphogenesis involved in differentiation |
| GO:0010975 | 3 | 488 | 0.0334927 | regulation of neuron projection development |
| GO:0050771 | 2 | 79 | 0.041774 | negative regulation of axonogenesis |

Table 2: Overrepresented GO terms with the standard enrichment

# 2 Network-based enrichment

| GO Term | N1 | N2 | P-value | Description |
|---|---|---|---|---|
| GO:0030308 | 4 | 575 | 0.00224156 | negative regulation of cell growth |
| GO:0045926 | 4 | 891 | 0.0126752 | negative regulation of growth |

Table 3: Overrepresented terms with the network-based enrichment. Only terms not detected with the standard method.
