# Supplementary material for: NET-GE: a novel NETwork-based Gene Enrichment for detecting biological processes associated to Mendelian diseases
Source: BMC Genomics. 2015 Jun 18;16(Suppl 8):S6. doi: 10.1186/1471-2164-16-S8-S6 (PMC4480278; doi:10.1186/1471-2164-16-S8-S6)
Supplement: Additional file 3 — Detailed results for the OMIM-derived benchmark set. The archive contains pdf documents listing the enriched terms for each one of the 244 diseases in the OMIM-derived benchmark set. [file 1471-2164-16-S8-S6-S3.tgz › SUPPMAT/OMIM136880.pdf]

#136880 FUNDUS ALBIPUNCTATUS

OMIM Gene ID	HGNC	UniProtAC
179605	PRPH2	P23942
180090	RLBP1	P12271
180380	RHO	P08100
601617	RDH5	Q92781

Table 1: OMIM - UniProtAC mapping

Legend

- N1: #input proteins associated to the significant GO term
- N2: #proteins associated to the significant GO term
- P-value: Bonferroni-corrected p-value of Fisher's exact test
- *red*: go terms not related to the input proteins
- *blue*: go terms related to the input proteins (enriched uniquely by network-based method)
- *green*: go terms ancestors of terms enriched with the standard method (enriched uniquely by network-based method)

1 Standard enrichment

GO Term	N1	N2	P-value	Description
GO:0007601	4	214	8.74289e-08	visual perception
GO:0050953	4	218	9.42001e-08	sensory perception of light stimulus
GO:0007603	3	89	4.40305e-06	phototransduction, visible light
GO:0007600	4	586	5.00469e-06	sensory perception
GO:0001523	3	96	5.53891e-06	retinoid metabolic process
GO:0016101	3	105	7.2658e-06	diterpenoid metabolic process
GO:0009584	3	106	7.47727e-06	detection of visible light
GO:0007602	3	112	8.83276e-06	phototransduction
GO:0006721	3	119	1.061e-05	terpenoid metabolic process
GO:0009583	3	129	1.354e-05	detection of light stimulus
GO:0006720	3	155	2.35686e-05	isoprenoid metabolic process
GO:0050877	4	1063	5.44404e-05	neurological system process
GO:0009581	3	221	6.86243e-05	detection of external stimulus
GO:0009582	3	224	7.14659e-05	detection of abiotic stimulus
GO:0003008	4	1588	0.000271645	system process
GO:0009416	3	424	0.000485819	response to light stimulus
GO:0009314	3	591	0.00131387	response to radiation
GO:0051606	3	761	0.00279868	detection of stimulus
GO:0060041	2	90	0.00292611	retina development in camera-type eye
GO:0009585	1	1	0.00922026	red, far-red light phototransduction
GO:0044707	4	4361	0.0154878	single-multicellular organism process
GO:0044255	3	1372	0.016227	cellular lipid metabolic process
GO:0032501	4	4447	0.0167465	multicellular organismal process
GO:0009628	3	1467	0.0198009	response to abiotic stimulus
GO:0006629	3	1831	0.0382285	lipid metabolic process
GO:0009605	3	1995	0.0492877	response to external stimulus

Table 2: Overrepresented GO terms with the standard enrichment

2 Network-based enrichment

No novel enriched terms
