# Supplementary material for: NET-GE: a novel NETwork-based Gene Enrichment for detecting biological processes associated to Mendelian diseases
Source: BMC Genomics. 2015 Jun 18;16(Suppl 8):S6. doi: 10.1186/1471-2164-16-S8-S6 (PMC4480278; doi:10.1186/1471-2164-16-S8-S6)
Supplement: Additional file 3 — Detailed results for the OMIM-derived benchmark set. The archive contains pdf documents listing the enriched terms for each one of the 244 diseases in the OMIM-derived benchmark set. [file 1471-2164-16-S8-S6-S3.tgz › SUPPMAT/OMIM137215.pdf]

#137215 GASTRIC CANCER, HEREDITARY DIFFUSE; HDGC

**OMIM Gene ID**	**HGNC**	**UniProtAC**
147679	IL1RN	P18510
147720	IL1B	P01584
190070	KRAS	P01116
192090	CDH1	P12830

Table 1: OMIM - UniProtAC mapping

Legend

- N1: #input proteins associated to the significant GO term
- N2: #proteins associated to the significant GO term
- P-value: Bonferroni-corrected p-value of Fisher's exact test
- *red*: go terms not related to the input proteins
- *blue*: go terms related to the input proteins (enriched uniquely by network-based method)
- *green*: go terms ancestors of terms enriched with the standard method (enriched uniquely by network-based method)

1 Standard enrichment

GO Term	N1	N2	P-value	Description
GO:0001660	2	4	4.02303e-05	fever generation
GO:0022407	3	120	9.95629e-05	regulation of cell-cell adhesion
GO:0031649	2	7	0.000140791	heat generation
GO:0010243	4	1094	0.000558884	response to organonitrogen compound
GO:1901698	4	1186	0.000772282	response to nitrogen compound
GO:0014070	4	1205	0.00082304	response to organic cyclic compound
GO:0034114	2	17	0.000911468	regulation of heterotypic cell-cell adhesion
GO:0043406	3	275	0.00121169	positive regulation of MAP kinase activity
GO:0032770	2	22	0.00154789	positive regulation of monooxygenase activity
GO:0003008	4	1588	0.0024854	system process
GO:0071902	3	357	0.00265325	positive regulation of protein serine/threonine kinase activity
GO:0001659	2	31	0.00311488	temperature homeostasis
GO:0043405	3	381	0.00322531	regulation of MAP kinase activity
GO:1901700	4	1851	0.00459043	response to oxygen-containing compound
GO:0042993	2	38	0.00470799	positive regulation of transcription factor import into nucleus
GO:0051353	2	42	0.0057653	positive regulation of oxidoreductase activity
GO:0042981	4	1970	0.00589085	regulation of apoptotic process
GO:0043067	4	1982	0.00603581	regulation of programmed cell death
GO:0009719	4	2012	0.00640992	response to endogenous stimulus
GO:0030155	3	485	0.00665048	regulation of cell adhesion
GO:0006953	2	46	0.00692943	acute-phase response
GO:0010941	4	2079	0.00730803	regulation of cell death
GO:0022408	2	49	0.00787262	negative regulation of cell-cell adhesion
GO:0043410	3	539	0.00912415	positive regulation of MAPK cascade
GO:0032768	2	57	0.0106812	regulation of monooxygenase activity
GO:0071900	3	600	0.0125775	regulation of protein serine/threonine kinase activity
GO:0045860	3	626	0.0142799	positive regulation of protein kinase activity
GO:0042493	3	633	0.0147631	response to drug
GO:0033674	3	656	0.0164267	positive regulation of kinase activity
GO:0032890	2	72	0.017097	regulation of organic acid transport
GO:0048871	2	77	0.0195685	multicellular organismal homeostasis
GO:0002526	2	84	0.0233078	acute inflammatory response
GO:0051347	3	751	0.0246138	positive regulation of transferase activity
GO:0051341	2	90	0.0267722	regulation of oxidoreductase activity
GO:0050793	4	2884	0.0270841	regulation of developmental process
GO:0043408	3	794	0.0290694	regulation of MAPK cascade
GO:0051246	4	2954	0.0298123	regulation of protein metabolic process
GO:0042990	2	101	0.0337446	regulation of transcription factor import into nucleus
GO:0042307	2	102	0.0344182	positive regulation of protein import into nucleus
GO:0034097	3	880	0.0395211	response to cytokine
GO:0009966	4	3261	0.0442833	regulation of signal transduction
GO:0010604	4	3285	0.045602	positive regulation of macromolecule metabolic process
GO:0046824	2	119	0.0468851	positive regulation of nucleocytoplasmic transport

Table 2: Overrepresented GO terms with the standard enrichment

2 Network-based enrichment

GO Term	N1	N2	P-value	Description
GO:0002790	4	199	2.25188e-06	peptide secretion
GO:0015833	4	254	6.01636e-06	peptide transport
GO:0009914	4	288	9.97219e-06	hormone transport
GO:0042886	4	301	1.19091e-05	amide transport
GO:0051384	4	564	0.000148181	response to glucocorticoid
GO:0031960	4	610	0.000202929	response to corticosteroid
GO:0050870	4	647	0.000256974	positive regulation of T cell activation
GO:0030336	4	660	0.000278308	negative regulation of cell migration
GO:2000146	4	688	0.000328749	negative regulation of cell motility
GO:0051271	4	710	0.00037296	negative regulation of cellular component movement
GO:0040013	4	840	0.000731672	negative regulation of locomotion
GO:0032496	4	847	0.000756412	response to lipopolysaccharide
GO:0010817	4	868	0.000834407	regulation of hormone levels
GO:0051251	4	874	0.00085776	positive regulation of lymphocyte activation
GO:0050863	4	912	0.00101724	regulation of T cell activation
GO:0002237	4	918	0.00104432	response to molecule of bacterial origin
GO:0002696	4	941	0.00115317	positive regulation of leukocyte activation
GO:0030072	3	189	0.00130498	peptide hormone secretion
GO:0050867	4	996	0.00144785	positive regulation of cell activation
GO:0043615	2	13	0.00153675	astrocyte cell migration
GO:0043271	3	206	0.00169133	negative regulation of ion transport
GO:0045639	3	226	0.00223522	positive regulation of myeloid cell differentiation
GO:0019221	4	1123	0.00234153	cytokine-mediated signaling pathway
GO:0048771	3	246	0.00288453	tissue remodeling
GO:0070555	3	246	0.00288453	response to interleukin-1
GO:0046879	3	253	0.0031384	hormone secretion
GO:0051249	4	1264	0.00376038	regulation of lymphocyte activation
GO:0045621	3	270	0.00381592	positive regulation of lymphocyte differentiation
GO:0042102	3	273	0.00394475	positive regulation of T cell proliferation
GO:0044070	3	280	0.00425654	regulation of anion transport
GO:0006954	4	1314	0.00439241	inflammatory response
GO:0001959	3	285	0.00448901	regulation of cytokine-mediated signaling pathway
GO:0060556	2	22	0.00454952	regulation of vitamin D biosynthetic process
GO:0007565	3	290	0.00472976	female pregnancy
GO:0033135	3	294	0.00492844	regulation of peptidyl-serine phosphorylation
GO:0051051	4	1375	0.00526769	negative regulation of transport
GO:0051092	3	312	0.0058913	positive regulation of NF-kappaB transcription factor activity
GO:0060759	3	318	0.00623805	regulation of response to cytokine stimulus
GO:0002694	4	1443	0.00639093	regulation of leukocyte activation
GO:0030656	2	28	0.00744289	regulation of vitamin metabolic process
GO:0071705	4	1511	0.00768489	nitrogen compound transport
GO:0048545	4	1516	0.00778722	response to steroid hormone
GO:0019371	2	29	0.00799389	cyclooxygenase pathway
GO:0032940	4	1530	0.00807917	secretion by cell
GO:0010942	4	1542	0.00833589	positive regulation of cell death
GO:0050865	4	1568	0.00891307	regulation of cell activation
GO:0043269	4	1590	0.0094244	regulation of ion transport
GO:0045580	3	369	0.00974795	regulation of T cell differentiation
GO:0002544	2	32	0.00976475	chronic inflammatory response
GO:0031347	4	1612	0.00995744	regulation of defense response
GO:1901699	4	1626	0.0103082	cellular response to nitrogen compound
GO:0001649	3	378	0.0104786	osteoblast differentiation
GO:0071345	4	1640	0.0106682	cellular response to cytokine stimulus
GO:0030217	3	386	0.0111579	T cell differentiation
GO:0050671	3	392	0.0116862	positive regulation of lymphocyte proliferation
GO:0050680	3	392	0.0116862	negative regulation of epithelial cell proliferation
GO:0032946	3	396	0.0120474	positive regulation of mononuclear cell proliferation
GO:0031669	3	400	0.012416	cellular response to nutrient levels
GO:0070665	3	403	0.0126973	positive regulation of leukocyte proliferation
GO:0007162	3	404	0.012792	negative regulation of cell adhesion

Table 3: Overrepresented terms with the network-based enrichment. Only terms not detected with the standard method.

GO Term	N1	N2	P-value	Description
GO:0032026	2	39	0.014584	response to magnesium ion
GO:0060395	2	39	0.014584	SMAD protein signal transduction
GO:0001817	4	1774	0.01461	regulation of cytokine production
GO:1902107	3	423	0.0146817	positive regulation of leukocyte differentiation
GO:0043409	3	424	0.0147859	negative regulation of MAPK cascade
GO:1901654	3	426	0.014996	response to ketone
GO:0043433	3	432	0.015638	negative regulation of sequence-specific DNA binding transcription factor activity
GO:0023061	3	434	0.015856	signal release
GO:0051044	2	41	0.0161375	positive regulation of membrane protein ectodomain proteolysis
GO:0042129	3	439	0.0164098	regulation of T cell proliferation
GO:0002548	2	44	0.018615	monocyte chemotaxis
GO:0046903	4	1910	0.019637	secretion
GO:0048469	3	468	0.0198767	cell maturation
GO:0030334	4	1926	0.0203038	regulation of cell migration
GO:0045619	3	475	0.0207807	regulation of lymphocyte differentiation
GO:0031668	3	479	0.0213092	cellular response to extracellular stimulus
GO:0044706	3	486	0.0222557	multi-multicellular organism process
GO:0007605	3	487	0.0223932	sensory perception of sound
GO:0046683	3	488	0.0225312	response to organophosphorus
GO:0060326	3	489	0.0226697	cell chemotaxis
GO:0008347	2	49	0.0231362	glial cell migration
GO:0007610	4	1993	0.0232823	behavior
GO:0046328	3	505	0.0249643	regulation of JNK cascade
GO:2000145	4	2039	0.0255091	regulation of cell motility
GO:0043903	3	510	0.0257118	regulation of symbiosis, encompassing mutualism through parasitism
GO:0050954	3	510	0.0257118	sensory perception of mechanical stimulus
GO:0050770	3	511	0.0258631	regulation of axonogenesis
GO:0009636	3	517	0.026783	response to toxic substance
GO:0043207	4	2065	0.0268364	response to external biotic stimulus
GO:0051043	2	53	0.0271058	regulation of membrane protein ectodomain proteolysis
GO:0060441	2	53	0.0271058	epithelial tube branching involved in lung morphogenesis
GO:0051046	4	2094	0.028377	regulation of secretion
GO:0043270	3	532	0.0291773	positive regulation of ion transport
GO:0006692	2	56	0.0302888	prostanoid metabolic process
GO:0006693	2	56	0.0302888	prostaglandin metabolic process
GO:0048754	3	542	0.0308499	branching morphogenesis of an epithelial tube
GO:0032535	3	546	0.0315364	regulation of cellular component size
GO:0014074	3	555	0.0331179	response to purine-containing compound
GO:0051270	4	2182	0.0334603	regulation of cellular component movement
GO:0051240	4	2185	0.0336447	positive regulation of multicellular organismal process
GO:0010762	2	59	0.033648	regulation of fibroblast migration
GO:0009607	4	2197	0.0343905	response to biotic stimulus
GO:0042445	3	566	0.0351212	hormone metabolic process
GO:0032872	3	568	0.035494	regulation of stress-activated MAPK cascade
GO:0070302	3	569	0.0356812	regulation of stress-activated protein kinase signaling cascade
GO:0018193	4	2224	0.0361138	peptidyl-amino acid modification
GO:0040012	4	2224	0.0361138	regulation of locomotion
GO:0001818	3	578	0.0373967	negative regulation of cytokine production
GO:0044703	3	580	0.0377851	multi-organism reproductive process
GO:2001236	3	581	0.0379804	regulation of extrinsic apoptotic signaling pathway
GO:0051048	3	583	0.0383731	negative regulation of secretion
GO:0002684	4	2287	0.040386	positive regulation of immune system process
GO:0045637	3	596	0.0409902	regulation of myeloid cell differentiation
GO:1900180	3	602	0.0422369	regulation of protein localization to nucleus
GO:0048638	3	605	0.0428697	regulation of developmental growth
GO:0050670	3	605	0.0428697	regulation of lymphocyte proliferation
GO:0010721	3	609	0.043723	negative regulation of cell development
GO:0032869	3	609	0.043723	cellular response to insulin stimulus
GO:0032944	3	609	0.043723	regulation of mononuclear cell proliferation
GO:0001501	3	620	0.0461277	skeletal system development

Table 4: Overrepresented terms with the network-based enrichment. Only terms not detected with the standard method.

GO Term	N1	N2	P-value	Description
GO:0070663	3	621	0.0463505	regulation of leukocyte proliferation
GO:0001934	4	2371	0.0466586	positive regulation of protein phosphorylation
GO:0061138	3	624	0.0470234	morphogenesis of a branching epithelium
GO:0050900	3	635	0.049546	leukocyte migration

Table 5: Overrepresented terms with the network-based enrichment. Only terms not detected with the standard method.
