# Supplementary material for: NET-GE: a novel NETwork-based Gene Enrichment for detecting biological processes associated to Mendelian diseases
Source: BMC Genomics. 2015 Jun 18;16(Suppl 8):S6. doi: 10.1186/1471-2164-16-S8-S6 (PMC4480278; doi:10.1186/1471-2164-16-S8-S6)
Supplement: Additional file 3 — Detailed results for the OMIM-derived benchmark set. The archive contains pdf documents listing the enriched terms for each one of the 244 diseases in the OMIM-derived benchmark set. [file 1471-2164-16-S8-S6-S3.tgz › SUPPMAT/OMIM137800.pdf]

#137800 GLIOMA SUSCEPTIBILITY 1; GLM1

OMIM Gene ID	HGNC	UniProtAC
147700	IDH1	O75874
164870	ERBB2	P04626
191170	TP53	P04637

Table 1: OMIM - UniProtAC mapping

Legend

- N1: #input proteins associated to the significant GO term
- N2: #proteins associated to the significant GO term
- P-value: Bonferroni-corrected p-value of Fisher's exact test
- *red*: go terms not related to the input proteins
- *blue*: go terms related to the input proteins (enriched uniquely by network-based method)
- *green*: go terms ancestors of terms enriched with the standard method (enriched uniquely by network-based method)

1 Standard enrichment

No enriched terms

2 Network-based enrichment

GO Term	N1	N2	P-value	Description
GO:0045943	2	13	0.000988614	positive regulation of transcription from RNA polymerase I promoter
GO:0010944	2	21	0.00266122	negative regulation of transcription by competitive promoter binding
GO:0045945	2	21	0.00266122	positive regulation of transcription from RNA polymerase III promoter
GO:0035794	2	25	0.00380146	positive regulation of mitochondrial membrane permeability
GO:0006356	2	32	0.00628418	regulation of transcription from RNA polymerase I promoter
GO:1902108	2	34	0.00710741	regulation of mitochondrial membrane permeability involved in apoptotic process
GO:0010665	2	37	0.00843719	regulation of cardiac muscle cell apoptotic process
GO:0010662	2	41	0.0103873	regulation of striated muscle cell apoptotic process
GO:0006359	2	50	0.0155148	regulation of transcription from RNA polymerase III promoter
GO:0008585	2	53	0.0174515	female gonad development
GO:0046902	2	56	0.019502	regulation of mitochondrial membrane permeability
GO:0035909	2	57	0.0202107	aorta morphogenesis
GO:0090559	2	58	0.0209321	regulation of membrane permeability
GO:0070741	2	59	0.0216662	response to interleukin-6
GO:0048864	2	62	0.0239441	stem cell development
GO:0002437	2	64	0.0255258	inflammatory response to antigenic stimulus
GO:0005979	2	65	0.0263355	regulation of glycogen biosynthetic process
GO:0010962	2	65	0.0263355	regulation of glucan biosynthetic process
GO:0003208	2	68	0.0288407	cardiac ventricle morphogenesis
GO:0035088	2	68	0.0288407	establishment or maintenance of apical/basal cell polarity
GO:0061245	2	68	0.0288407	establishment or maintenance of bipolar cell polarity
GO:0090103	2	70	0.0305741	cochlea morphogenesis
GO:0002886	2	73	0.0332687	regulation of myeloid leukocyte mediated immunity
GO:0006739	2	74	0.0341922	NADP metabolic process
GO:0048147	2	75	0.0351281	negative regulation of fibroblast proliferation
GO:0032885	2	77	0.0370381	regulation of polysaccharide biosynthetic process
GO:0010907	2	80	0.0399977	positive regulation of glucose metabolic process
GO:0048008	2	80	0.0399977	platelet-derived growth factor receptor signaling pathway
GO:0003206	2	83	0.0430711	cardiac chamber morphogenesis
GO:0070873	2	86	0.0462577	regulation of glycogen metabolic process
GO:0048738	2	87	0.0473451	cardiac muscle tissue development

Table 2: Overrepresented terms with the network-based enrichment. Only terms not detected with the standard method.
