# Supplementary material for: NET-GE: a novel NETwork-based Gene Enrichment for detecting biological processes associated to Mendelian diseases
Source: BMC Genomics. 2015 Jun 18;16(Suppl 8):S6. doi: 10.1186/1471-2164-16-S8-S6 (PMC4480278; doi:10.1186/1471-2164-16-S8-S6)
Supplement: Additional file 3 — Detailed results for the OMIM-derived benchmark set. The archive contains pdf documents listing the enriched terms for each one of the 244 diseases in the OMIM-derived benchmark set. [file 1471-2164-16-S8-S6-S3.tgz › SUPPMAT/OMIM138500-OMIM242600.pdf]

#138500 HYPERGLYCINURIA
#242600 IMINOGLYCINURIA

OMIM Gene ID	HGNC	UniProtAC
605616	SLC6A20	Q9NP91
608331	SLC36A2	Q495M3
608893	SLC6A19	Q695T7

Table 1: OMIM - UniProtAC mapping

Legend

- N1: #input proteins associated to the significant GO term
- N2: #proteins associated to the significant GO term
- P-value: Bonferroni-corrected p-value of Fisher's exact test
- *red*: go terms not related to the input proteins
- *blue*: go terms related to the input proteins (enriched uniquely by network-based method)
- *green*: go terms ancestors of terms enriched with the standard method (enriched uniquely by network-based method)

1 Standard enrichment

GO Term	N1	N2	P-value	Description
GO:0015804	3	56	1.20652e-07	neutral amino acid transport
GO:0006865	3	223	7.93669e-06	amino acid transport
GO:0015824	2	15	1.72442e-05	proline transport
GO:0015816	2	19	2.80815e-05	glycine transport
GO:0046942	3	378	3.88697e-05	carboxylic acid transport
GO:0015849	3	382	4.01201e-05	organic acid transport
GO:0015711	3	544	0.000116142	organic anion transport
GO:0071705	3	691	0.000238307	nitrogen compound transport
GO:0006820	3	806	0.000378422	anion transport
GO:0015807	2	88	0.000627865	L-amino acid transport
GO:0003333	2	109	0.000965059	amino acid transmembrane transport
GO:0006836	2	154	0.00193005	neurotransmitter transport
GO:0098656	2	320	0.00833719	anion transmembrane transport
GO:0055085	3	2352	0.00942646	transmembrane transport
GO:0006811	3	2423	0.0103065	ion transport
GO:0071702	3	2983	0.0192359	organic substance transport
GO:0035524	1	11	0.03409	proline transmembrane transport
GO:0015808	1	15	0.0464814	L-alanine transport

Table 2: Overrepresented GO terms with the standard enrichment

2 Network-based enrichment

No novel enriched terms
