# Supplementary material for: NET-GE: a novel NETwork-based Gene Enrichment for detecting biological processes associated to Mendelian diseases
Source: BMC Genomics. 2015 Jun 18;16(Suppl 8):S6. doi: 10.1186/1471-2164-16-S8-S6 (PMC4480278; doi:10.1186/1471-2164-16-S8-S6)
Supplement: Additional file 3 — Detailed results for the OMIM-derived benchmark set. The archive contains pdf documents listing the enriched terms for each one of the 244 diseases in the OMIM-derived benchmark set. [file 1471-2164-16-S8-S6-S3.tgz › SUPPMAT/OMIM140700.pdf]

#140700 HEINZ BODY ANEMIAS

OMIM Gene ID	HGNC	UniProtAC
141800	HBA1	P69905
141850	HBA2	P69905
141900	HBB	P68871

Table 1: OMIM - UniProtAC mapping

Legend

- N1: #input proteins associated to the significant GO term
- N2: #proteins associated to the significant GO term
- P-value: Bonferroni-corrected p-value of Fisher's exact test
- *red*: go terms not related to the input proteins
- *blue*: go terms related to the input proteins (enriched uniquely by network-based method)
- *green*: go terms ancestors of terms enriched with the standard method (enriched uniquely by network-based method)

1 Standard enrichment

GO Term	N1	N2	P-value	Description
GO:0042744	2	20	2.48088e-05	hydrogen peroxide catabolic process
GO:0015671	2	31	6.07162e-05	oxygen transport
GO:0015669	2	35	7.76906e-05	gas transport
GO:0015701	2	35	7.76906e-05	bicarbonate transport
GO:0042743	2	39	9.67544e-05	hydrogen peroxide metabolic process
GO:0070301	2	90	0.000522943	cellular response to hydrogen peroxide
GO:0072593	2	110	0.000782782	reactive oxygen species metabolic process
GO:0034614	2	133	0.00114617	cellular response to reactive oxygen species
GO:0042542	2	167	0.00180986	response to hydrogen peroxide
GO:0051291	2	177	0.0020338	protein heterooligomerization
GO:0000302	2	236	0.00362078	response to reactive oxygen species
GO:0034599	2	250	0.00406407	cellular response to oxidative stress
GO:0030185	1	3	0.0147838	nitric oxide transport
GO:0006979	2	523	0.0178235	response to oxidative stress
GO:0015711	2	544	0.019285	organic anion transport
GO:0010942	2	579	0.0218489	positive regulation of cell death
GO:0051259	2	582	0.0220761	protein oligomerization
GO:0010035	2	612	0.0244126	response to inorganic substance
GO:0006820	2	806	0.0423596	anion transport

Table 2: Overrepresented GO terms with the standard enrichment

2 Network-based enrichment

GO Term	N1	N2	P-value	Description
GO:0045429	2	70	0.000922607	positive regulation of nitric oxide biosynthetic process
GO:0045907	2	78	0.00114724	positive regulation of vasoconstriction
GO:0045428	2	117	0.00259246	regulation of nitric oxide biosynthetic process
GO:0019229	2	143	0.00387877	regulation of vasoconstriction
GO:0050880	2	187	0.00664391	regulation of blood vessel size
GO:0035150	2	198	0.00745076	regulation of tube size
GO:0050821	2	232	0.0102369	protein stabilization
GO:0003018	2	250	0.0118907	vascular process in circulatory system
GO:0030097	2	338	0.0217578	hemopoiesis
GO:0031647	2	386	0.0283868	regulation of protein stability
GO:0008217	2	438	0.0365615	regulation of blood pressure
GO:0003013	2	444	0.0375713	circulatory system process

Table 3: Overrepresented terms with the network-based enrichment. Only terms not detected with the standard method.
