# Supplementary material for: NET-GE: a novel NETwork-based Gene Enrichment for detecting biological processes associated to Mendelian diseases
Source: BMC Genomics. 2015 Jun 18;16(Suppl 8):S6. doi: 10.1186/1471-2164-16-S8-S6 (PMC4480278; doi:10.1186/1471-2164-16-S8-S6)
Supplement: Additional file 3 — Detailed results for the OMIM-derived benchmark set. The archive contains pdf documents listing the enriched terms for each one of the 244 diseases in the OMIM-derived benchmark set. [file 1471-2164-16-S8-S6-S3.tgz › SUPPMAT/OMIM141749.pdf]

#141749 FETAL HEMOGLOBIN QUANTITATIVE TRAIT LOCUS 1; HBFQTL1

OMIM Gene ID	HGNC	UniProtAC
141900	HBB	P68871
142200	HBG1	P69891
142250	HBG2	P69892

Table 1: OMIM - UniProtAC mapping

Legend

- N1: #input proteins associated to the significant GO term
- N2: #proteins associated to the significant GO term
- P-value: Bonferroni-corrected p-value of Fisher's exact test
- *red*: go terms not related to the input proteins
- *blue*: go terms related to the input proteins (enriched uniquely by network-based method)
- *green*: go terms ancestors of terms enriched with the standard method (enriched uniquely by network-based method)

1 Standard enrichment

GO Term	N1	N2	P-value	Description
GO:0015671	3	31	4.6654e-08	oxygen transport
GO:0015669	3	35	6.79312e-08	gas transport
GO:0007596	3	501	0.000216231	blood coagulation
GO:0050817	3	501	0.000216231	coagulation
GO:0007599	3	510	0.000228118	hemostasis
GO:0050878	3	717	0.00063496	regulation of body fluid levels
GO:0030185	1	3	0.0221751	nitric oxide transport

Table 2: Overrepresented GO terms with the standard enrichment

2 Network-based enrichment

GO Term	N1	N2	P-value	Description
GO:0030097	2	338	0.0336406	hemopoiesis

Table 3: Overrepresented terms with the network-based enrichment. Only terms not detected with the standard method.
