# Supplementary material for: NET-GE: a novel NETwork-based Gene Enrichment for detecting biological processes associated to Mendelian diseases
Source: BMC Genomics. 2015 Jun 18;16(Suppl 8):S6. doi: 10.1186/1471-2164-16-S8-S6 (PMC4480278; doi:10.1186/1471-2164-16-S8-S6)
Supplement: Additional file 3 — Detailed results for the OMIM-derived benchmark set. The archive contains pdf documents listing the enriched terms for each one of the 244 diseases in the OMIM-derived benchmark set. [file 1471-2164-16-S8-S6-S3.tgz › SUPPMAT/OMIM143465.pdf]

#143465 ATTENTION DEFICIT-HYPERACTIVITY DISORDER; ADHD

**OMIM Gene ID**	**HGNC**	**UniProtAC**
126452	DRD4	P21917
126453	DRD5	P21918

Table 1: OMIM - UniProtAC mapping

Legend

- N1: #input proteins associated to the significant GO term
- N2: #proteins associated to the significant GO term
- P-value: Bonferroni-corrected p-value of Fisher's exact test
- *red*: go terms not related to the input proteins
- *blue*: go terms related to the input proteins (enriched uniquely by network-based method)
- *green*: go terms ancestors of terms enriched with the standard method (enriched uniquely by network-based method)

1 Standard enrichment

GO Term	N1	N2	P-value	Description
GO:0001963	2	19	8.71509e-05	synaptic transmission, dopaminergic
GO:0007212	2	42	0.000438813	dopamine receptor signaling pathway
GO:0001975	2	50	0.000624327	response to amphetamine
GO:0042220	2	52	0.000675801	response to cocaine
GO:0045761	2	69	0.00119565	regulation of adenylate cyclase activity
GO:0014075	2	71	0.00126649	response to amine
GO:0031279	2	81	0.00165128	regulation of cyclase activity
GO:0007270	2	85	0.00181946	neuron-neuron synaptic transmission
GO:0051339	2	85	0.00181946	regulation of lyase activity
GO:0008306	2	89	0.0019958	associative learning
GO:0060359	2	105	0.00278271	response to ammonium ion
GO:0051705	2	116	0.00339939	multi-organism behavior
GO:0030817	2	117	0.00345851	regulation of cAMP biosynthetic process
GO:0030814	2	133	0.00447376	regulation of cAMP metabolic process
GO:0030802	2	137	0.00474793	regulation of cyclic nucleotide biosynthetic process
GO:0030808	2	140	0.00495894	regulation of nucleotide biosynthetic process
GO:1900371	2	140	0.00495894	regulation of purine nucleotide biosynthetic process
GO:0030799	2	155	0.00608272	regulation of cyclic nucleotide metabolic process
GO:0007188	2	162	0.00664638	adenylate cyclase-modulating G-protein coupled receptor signaling pathway
GO:0007612	2	163	0.00672897	learning
GO:0043279	2	188	0.00895869	response to alkaloid
GO:0007187	2	193	0.00944286	G-protein coupled receptor signaling pathway, coupled to cyclic nucleotide second messenger
GO:0007611	2	329	0.0274989	learning or memory
GO:0006874	2	359	0.0327509	cellular calcium ion homeostasis
GO:0050890	2	365	0.0338563	cognition
GO:0055074	2	373	0.0353588	calcium ion homeostasis
GO:0072503	2	373	0.0353588	cellular divalent inorganic cation homeostasis
GO:0008355	1	2	0.0384704	olfactory learning
GO:0033861	1	2	0.0384704	negative regulation of NAD(P)H oxidase activity
GO:0046960	1	2	0.0384704	sensitization
GO:0050804	2	390	0.0386599	regulation of synaptic transmission
GO:0072507	2	398	0.0402643	divalent inorganic cation homeostasis

Table 2: Overrepresented GO terms with the standard enrichment

2 Network-based enrichment

GO Term	N1	N2	P-value	Description
GO:0014052	2	16	0.000110933	regulation of gamma-aminobutyric acid secretion
GO:0034776	2	19	0.00015808	response to histamine
GO:0051954	2	65	0.00192284	positive regulation of amine transport
GO:0051955	2	69	0.00216875	regulation of amino acid transport
GO:0001662	2	78	0.0027761	behavioral fear response
GO:0002209	2	78	0.0027761	behavioral defense response
GO:0042596	2	88	0.00353877	fear response
GO:0032228	2	101	0.00466844	regulation of synaptic transmission, GABAergic
GO:0050805	2	114	0.00595434	negative regulation of synaptic transmission
GO:0032890	2	156	0.0111765	regulation of organic acid transport
GO:0051952	2	167	0.0128137	regulation of amine transport
GO:0060078	2	208	0.0199014	regulation of postsynaptic membrane potential
GO:0033555	2	237	0.025853	multicellular organismal response to stress
GO:0044070	2	280	0.0361088	regulation of anion transport
GO:0098661	2	303	0.0422961	inorganic anion transmembrane transport

Table 3: Overrepresented terms with the network-based enrichment. Only terms not detected with the standard method.
