# Supplementary material for: NET-GE: a novel NETwork-based Gene Enrichment for detecting biological processes associated to Mendelian diseases
Source: BMC Genomics. 2015 Jun 18;16(Suppl 8):S6. doi: 10.1186/1471-2164-16-S8-S6 (PMC4480278; doi:10.1186/1471-2164-16-S8-S6)
Supplement: Additional file 3 — Detailed results for the OMIM-derived benchmark set. The archive contains pdf documents listing the enriched terms for each one of the 244 diseases in the OMIM-derived benchmark set. [file 1471-2164-16-S8-S6-S3.tgz › SUPPMAT/OMIM143890.pdf]

#143890 HYPERCHOLESTEROLEMIA, FAMILIAL

**OMIM Gene ID**	**HGNC**	**UniProtAC**
107670	APOA2	P02652
132811	EPHX2	P34913
600046	ABCA1	O95477
600564	ITIH4	Q14624
600946	GHR	P10912
604088	PPP1R17	O96001
606945	LDLR	P01130

Table 1: OMIM - UniProtAC mapping

Legend

- N1: #input proteins associated to the significant GO term
- N2: #proteins associated to the significant GO term
- P-value: Bonferroni-corrected p-value of Fisher's exact test
- *red*: go terms not related to the input proteins
- *blue*: go terms related to the input proteins (enriched uniquely by network-based method)
- *green*: go terms ancestors of terms enriched with the standard method (enriched uniquely by network-based method)

1 Standard enrichment

GO Term	N1	N2	P-value	Description
GO:0042632	4	95	8.01953e-07	cholesterol homeostasis
GO:0055092	4	96	8.36769e-07	sterol homeostasis
GO:0055088	4	140	3.85056e-06	lipid homeostasis
GO:0015918	3	66	0.000108844	sterol transport
GO:0030301	3	66	0.000108844	cholesterol transport
GO:0042157	3	87	0.000251698	lipoprotein metabolic process
GO:0034380	2	6	0.000270568	high-density lipoprotein particle assembly
GO:0015914	3	102	0.000407236	phospholipid transport
GO:0044255	5	1372	0.000761891	cellular lipid metabolic process
GO:0015748	3	134	0.000927572	organophosphate ester transport
GO:0015850	3	139	0.00103576	organic hydroxy compound transport
GO:0008203	3	140	0.00105836	cholesterol metabolic process
GO:0033700	2	12	0.00118987	phospholipid efflux
GO:0016125	3	158	0.00152289	sterol metabolic process
GO:0034377	2	17	0.00245077	plasma lipoprotein particle assembly
GO:0043691	2	17	0.00245077	reverse cholesterol transport
GO:0065005	2	19	0.00308094	protein-lipid complex assembly
GO:0006629	5	1831	0.00316406	lipid metabolic process
GO:0033344	2	31	0.0083691	cholesterol efflux
GO:0034381	2	37	0.0119804	plasma lipoprotein particle clearance
GO:0008202	3	325	0.0132078	steroid metabolic process
GO:0048878	4	1094	0.0140217	chemical homeostasis
GO:0071827	2	40	0.0140273	plasma lipoprotein particle organization
GO:0071825	2	42	0.0154813	protein-lipid complex subunit organization
GO:0006869	3	348	0.0161952	lipid transport
GO:0019216	3	375	0.0202337	regulation of lipid metabolic process
GO:0032371	2	48	0.0202714	regulation of sterol transport
GO:0032374	2	48	0.0202714	regulation of cholesterol transport
GO:0006066	3	446	0.0338902	alcohol metabolic process
GO:0050994	2	67	0.0396675	regulation of lipid catabolic process
GO:0006954	3	483	0.0429387	inflammatory response

Table 2: Overrepresented GO terms with the standard enrichment

2 Network-based enrichment

GO Term	N1	N2	P-value	Description
GO:0043086	6	2637	0.00135551	negative regulation of catalytic activity
GO:0018158	2	13	0.00250327	protein oxidation
GO:0044092	6	3258	0.00474366	negative regulation of molecular function
GO:0032368	3	263	0.0141189	regulation of lipid transport
GO:0019218	3	278	0.0166627	regulation of steroid metabolic process
GO:0045834	3	315	0.0241904	positive regulation of lipid metabolic process
GO:0032369	2	51	0.0407635	negative regulation of lipid transport
GO:0034763	2	56	0.0492112	negative regulation of transmembrane transport

Table 3: Overrepresented terms with the network-based enrichment. Only terms not detected with the standard method.
