# Supplementary material for: NET-GE: a novel NETwork-based Gene Enrichment for detecting biological processes associated to Mendelian diseases
Source: BMC Genomics. 2015 Jun 18;16(Suppl 8):S6. doi: 10.1186/1471-2164-16-S8-S6 (PMC4480278; doi:10.1186/1471-2164-16-S8-S6)
Supplement: Additional file 3 — Detailed results for the OMIM-derived benchmark set. The archive contains pdf documents listing the enriched terms for each one of the 244 diseases in the OMIM-derived benchmark set. [file 1471-2164-16-S8-S6-S3.tgz › SUPPMAT/OMIM144700.pdf]

#144700 RENAL CELL CARCINOMA, NONPAPILLARY; RCC

OMIM Gene ID	HGNC	UniProtAC
142410	HNF1A	P20823
189907	HNF1B	P35680
601982	OGG1	O15527
602773	DIRC2	Q96SL1
603046	RNF139	Q8WU17
607273	FLCN	Q8NFG4
608537	VHL	P40337

Table 1: OMIM - UniProtAC mapping

Legend

- N1: #input proteins associated to the significant GO term
- N2: #proteins associated to the significant GO term
- P-value: Bonferroni-corrected p-value of Fisher's exact test
- *red*: go terms not related to the input proteins
- *blue*: go terms related to the input proteins (enriched uniquely by network-based method)
- *green*: go terms ancestors of terms enriched with the standard method (enriched uniquely by network-based method)

1 Standard enrichment

GO Term	N1	N2	P-value	Description
GO:0060261	2	12	0.000974059	positive regulation of transcription initiation from RNA polymerase II promoter
GO:2000144	2	13	0.00115106	positive regulation of DNA-templated transcription, initiation
GO:0060260	2	22	0.0034062	regulation of transcription initiation from RNA polymerase II promoter
GO:2000142	2	25	0.00442247	regulation of DNA-templated transcription, initiation
GO:0030073	2	48	0.0165947	insulin secretion
GO:0090183	2	76	0.0418245	regulation of kidney development
GO:0030072	2	77	0.0429361	peptide hormone secretion
GO:0002790	2	79	0.0452025	peptide secretion

Table 2: Overrepresented GO terms with the standard enrichment

2 Network-based enrichment

GO Term	N1	N2	P-value	Description
GO:0008285	5	2354	0.00753965	negative regulation of cell proliferation
GO:0061333	2	46	0.0212153	renal tubule morphogenesis
GO:1902532	4	1289	0.0239207	negative regulation of intracellular signal transduction
GO:0031334	3	411	0.0275974	positive regulation of protein complex assembly
GO:0072009	2	62	0.0387118	nephron epithelium development
GO:0060341	5	3341	0.0423352	regulation of cellular localization
GO:0051241	4	1513	0.0449355	negative regulation of multicellular organismal process

Table 3: Overrepresented terms with the network-based enrichment. Only terms not detected with the standard method.
