# Supplementary material for: NET-GE: a novel NETwork-based Gene Enrichment for detecting biological processes associated to Mendelian diseases
Source: BMC Genomics. 2015 Jun 18;16(Suppl 8):S6. doi: 10.1186/1471-2164-16-S8-S6 (PMC4480278; doi:10.1186/1471-2164-16-S8-S6)
Supplement: Additional file 3 — Detailed results for the OMIM-derived benchmark set. The archive contains pdf documents listing the enriched terms for each one of the 244 diseases in the OMIM-derived benchmark set. [file 1471-2164-16-S8-S6-S3.tgz › SUPPMAT/OMIM145500.pdf]

#145500 HYPERTENSION, ESSENTIAL

OMIM Gene ID	HGNC	UniProtAC
102680	ADD1	P35611
106150	AGT	P01019
106165	AGTR1	P30556
131210	SELE	P16581
139130	GNB3	P16520
163729	NOS3	P29474
163730	NOS2	P35228
171190	PNMT	P11086
182330	ATP1B1	P05026
600423	ECE1	P42892
601699	PTGIS	Q16647
603276	RGS5	O15539
605325	CYP3A5	P20815

Table 1: OMIM - UniProtAC mapping

Legend

- N1: #input proteins associated to the significant GO term
- N2: #proteins associated to the significant GO term
- P-value: Bonferroni-corrected p-value of Fisher's exact test
- *red*: go terms not related to the input proteins
- *blue*: go terms related to the input proteins (enriched uniquely by network-based method)
- *green*: go terms ancestors of terms enriched with the standard method (enriched uniquely by network-based method)

1 Standard enrichment

GO Term	N1	N2	P-value	Description
GO:0019932	6	176	1.56507e-08	second-messenger-mediated signaling
GO:0008217	6	197	3.09615e-08	regulation of blood pressure
GO:0001990	4	29	1.98518e-07	regulation of systemic arterial blood pressure by hormone
GO:0003044	4	29	1.98518e-07	regulation of systemic arterial blood pressure mediated by a chemical signal
GO:0050886	4	29	1.98518e-07	endocrine process
GO:0003073	4	52	2.25289e-06	regulation of systemic arterial blood pressure
GO:0044057	6	554	1.51996e-05	regulation of system process
GO:0007263	3	18	2.57862e-05	nitric oxide mediated signal transduction
GO:0043085	8	2150	0.000108075	positive regulation of catalytic activity
GO:0044093	8	2479	0.000324669	positive regulation of molecular function
GO:0033864	2	3	0.000326174	positive regulation of NAD(P)H oxidase activity
GO:1903034	5	529	0.000618179	regulation of response to wounding
GO:0065008	9	3888	0.000624129	regulation of biological quality
GO:0002018	2	4	0.000652221	renin-angiotensin regulation of aldosterone production
GO:0002034	2	4	0.000652221	regulation of blood vessel size by renin-angiotensin
GO:0003072	2	4	0.000652221	renal control of peripheral vascular resistance involved in regulation of systemic arterial blood pressure
GO:0065009	9	3941	0.000701192	regulation of molecular function
GO:0042312	3	53	0.000735146	regulation of vasodilation
GO:0003100	2	5	0.00108683	regulation of systemic arterial blood pressure by endothelin
GO:0045765	4	250	0.00127306	regulation of angiogenesis
GO:0051336	7	1982	0.00140299	regulation of hydrolase activity
GO:0019229	3	68	0.00156804	regulation of vasoconstriction
GO:0033860	2	6	0.00162992	regulation of NAD(P)H oxidase activity
GO:1901342	4	271	0.00175402	regulation of vasculature development
GO:0051049	7	2081	0.00194627	regulation of transport
GO:0010873	2	7	0.00228145	positive regulation of cholesterol esterification
GO:0002028	3	83	0.00286622	regulation of sodium ion transport
GO:0050790	8	3371	0.0034025	regulation of catalytic activity
GO:0050880	3	90	0.00365968	regulation of blood vessel size
GO:0051341	3	90	0.00365968	regulation of oxidoreductase activity
GO:0035150	3	91	0.0037837	regulation of tube size
GO:0051239	8	3432	0.00389758	regulation of multicellular organismal process
GO:0006527	2	9	0.00390953	arginine catabolic process
GO:0010872	2	9	0.00390953	regulation of cholesterol esterification
GO:0051345	6	1431	0.00398183	positive regulation of hydrolase activity
GO:0010518	3	93	0.00404	positive regulation of phospholipase activity
GO:0032101	5	825	0.00544607	regulation of response to external stimulus
GO:0090066	4	362	0.00551889	regulation of anatomical structure size
GO:0010517	3	104	0.00565705	regulation of phospholipase activity
GO:0050727	4	372	0.00614541	regulation of inflammatory response
GO:0060193	3	108	0.00633705	positive regulation of lipase activity
GO:0031284	2	12	0.00716329	positive regulation of guanylate cyclase activity
GO:0019722	3	113	0.00726041	calcium-mediated signaling
GO:0003018	3	117	0.00806005	vascular process in circulatory system
GO:0006883	2	13	0.00846406	cellular sodium ion homeostasis
GO:0031282	2	13	0.00846406	regulation of guanylate cyclase activity
GO:0034374	2	13	0.00846406	low-density lipoprotein particle remodeling
GO:0045766	3	131	0.0113133	positive regulation of angiogenesis
GO:0003081	2	16	0.0130141	regulation of systemic arterial blood pressure by renin-angiotensin
GO:0006809	2	16	0.0130141	nitric oxide biosynthetic process
GO:0032879	7	2827	0.0149347	regulation of localization
GO:0007165	10	7592	0.0167567	signal transduction
GO:0060191	3	155	0.0187179	regulation of lipase activity
GO:0007596	4	501	0.0198057	blood coagulation
GO:0050817	4	501	0.0198057	coagulation
GO:0007599	4	510	0.0212354	hemostasis
GO:0030826	2	21	0.0227525	regulation of cGMP biosynthetic process
GO:0044707	8	4361	0.023542	single-multicellular organism process
GO:0006525	2	22	0.0250228	arginine metabolic process
GO:0003013	3	173	0.0259853	circulatory system process

Table 2: Overrepresented GO terms with the standard enrichment

GO Term	N1	N2	P-value	Description
GO:0032501	8	4447	0.0272189	multicellular organismal process
GO:0003071	2	23	0.0274006	renal system process involved in regulation of systemic arterial blood pressure
GO:0030823	2	24	0.0298858	regulation of cGMP metabolic process
GO:0055078	2	26	0.0351779	sodium ion homeostasis
GO:0045940	2	28	0.0408988	positive regulation of steroid metabolic process
GO:0035813	2	30	0.0470477	regulation of renal sodium excretion

Table 3: Overrepresented GO terms with the standard enrichment

2 Network-based enrichment

GO Term	N1	N2	P-value	Description
GO:0010744	4	62	1.6387e-05	positive regulation of macrophage derived foam cell differentiation
GO:0010817	7	868	2.54244e-05	regulation of hormone levels
GO:0010743	4	93	8.51955e-05	regulation of macrophage derived foam cell differentiation
GO:0043535	4	125	0.000280834	regulation of blood vessel endothelial cell migration
GO:0051249	7	1264	0.000333679	regulation of lymphocyte activation
GO:0032102	6	737	0.000368595	negative regulation of response to external stimulus
GO:0034097	8	2017	0.000371827	response to cytokine
GO:1903035	5	366	0.00039802	negative regulation of response to wounding
GO:0051094	9	3200	0.000733595	positive regulation of developmental process
GO:0002694	7	1443	0.000820724	regulation of leukocyte activation
GO:0023061	5	434	0.000924406	signal release
GO:1901615	7	1475	0.000952236	organic hydroxy compound metabolic process
GO:0071902	6	897	0.00116771	positive regulation of protein serine/threonine kinase activity
GO:0071354	3	45	0.00139287	cellular response to interleukin-6
GO:0050865	7	1568	0.00144007	regulation of cell activation
GO:0060326	5	489	0.00166443	cell chemotaxis
GO:0044062	3	49	0.00180684	regulation of excretion
GO:1900542	8	2485	0.00185221	regulation of purine nucleotide metabolic process
GO:0043066	8	2487	0.00186366	negative regulation of apoptotic process
GO:0090075	3	50	0.00192174	relaxation of muscle
GO:0006140	8	2500	0.00193956	regulation of nucleotide metabolic process
GO:0071345	7	1640	0.00194984	cellular response to cytokine stimulus
GO:0051050	8	2507	0.00198152	positive regulation of transport
GO:0043069	8	2511	0.00200584	negative regulation of programmed cell death
GO:0001666	6	987	0.00204153	response to hypoxia
GO:0036293	6	999	0.00219062	response to decreased oxygen levels
GO:0007600	7	1675	0.00224811	sensory perception
GO:0043270	5	532	0.00251898	positive regulation of ion transport
GO:0009065	3	55	0.00256939	glutamine family amino acid catabolic process
GO:0002526	4	220	0.00269582	acute inflammatory response
GO:0006875	6	1046	0.00286348	cellular metal ion homeostasis
GO:0060548	8	2642	0.00295875	negative regulation of cell death
GO:0097190	6	1052	0.00296041	apoptotic signaling pathway
GO:0051924	5	553	0.00304622	regulation of calcium ion transport
GO:0090287	5	557	0.00315585	regulation of cellular response to growth factor stimulus
GO:0070741	3	59	0.00318098	response to interleukin-6
GO:0001817	7	1774	0.00330844	regulation of cytokine production
GO:0009713	3	60	0.00334764	catechol-containing compound biosynthetic process
GO:0042423	3	60	0.00334764	catecholamine biosynthetic process
GO:0070482	6	1076	0.00337556	response to oxygen levels
GO:0042445	5	566	0.00341393	hormone metabolic process
GO:0050878	7	1837	0.00418197	regulation of body fluid levels
GO:0070555	4	246	0.00420298	response to interleukin-1
GO:0010955	4	247	0.00427124	negative regulation of protein processing
GO:1903318	4	247	0.00427124	negative regulation of protein maturation
GO:0019221	6	1123	0.0043275	cytokine-mediated signaling pathway
GO:0051130	8	2782	0.00438559	positive regulation of cellular component organization
GO:0019725	7	1855	0.00446461	cellular homeostasis
GO:0045909	3	66	0.00447052	positive regulation of vasodilation
GO:0030003	6	1151	0.00499236	cellular cation homeostasis
GO:0022603	8	2832	0.00502199	regulation of anatomical structure morphogenesis
GO:1901606	4	266	0.00573141	alpha-amino acid catabolic process
GO:0030334	7	1926	0.00574195	regulation of cell migration
GO:0046209	3	72	0.00581866	nitric oxide metabolic process
GO:0045785	5	632	0.00585812	positive regulation of cell adhesion
GO:0050900	5	635	0.00599529	leukocyte migration
GO:1901565	8	2901	0.00602982	organonitrogen compound catabolic process
GO:0032846	4	270	0.00608078	positive regulation of homeostatic process
GO:0007268	6	1195	0.00620491	synaptic transmission
GO:0006873	6	1202	0.00641834	cellular ion homeostasis

Table 4: Overrepresented terms with the network-based enrichment. Only terms not detected with the standard method.

GO Term	N1	N2	P-value	Description
GO:0046942	5	650	0.00671963	carboxylic acid transport
GO:0019218	4	278	0.00682668	regulation of steroid metabolic process
GO:0015849	5	654	0.006924	organic acid transport
GO:0043406	5	661	0.00729339	positive regulation of MAP kinase activity
GO:0019752	8	2978	0.00735546	carboxylic acid metabolic process
GO:0055065	6	1233	0.00743736	metal ion homeostasis
GO:0030100	5	667	0.00762223	regulation of endocytosis
GO:0009914	4	288	0.0078521	hormone transport
GO:0050999	3	81	0.00830722	regulation of nitric-oxide synthase activity
GO:0014805	2	8	0.00839151	smooth muscle adaptation
GO:0060452	2	8	0.00839151	positive regulation of cardiac muscle contraction
GO:2000145	7	2039	0.00840483	regulation of cell motility
GO:0048534	5	682	0.00849566	hematopoietic or lymphoid organ development
GO:0007200	3	82	0.00862075	phospholipase C-activating G-protein coupled receptor signaling pathway
GO:2000649	3	82	0.00862075	regulation of sodium ion transmembrane transporter activity
GO:0010543	3	83	0.008942	regulation of platelet activation
GO:0017144	3	84	0.00927109	drug metabolic process
GO:0090288	4	301	0.00935081	negative regulation of cellular response to growth factor stimulus
GO:0051262	4	304	0.00972482	protein tetramerization
GO:0033121	7	2107	0.0104594	regulation of purine nucleotide catabolic process
GO:0030811	7	2111	0.0105924	regulation of nucleotide catabolic process
GO:0010535	2	9	0.0107867	positive regulation of activation of JAK2 kinase activity
GO:0009118	7	2125	0.011069	regulation of nucleoside metabolic process
GO:0045834	4	315	0.0111916	positive regulation of lipid metabolic process
GO:0031349	5	726	0.0115204	positive regulation of defense response
GO:0050728	4	318	0.0116186	negative regulation of inflammatory response
GO:0018958	4	319	0.0117636	phenol-containing compound metabolic process
GO:0071453	4	319	0.0117636	cellular response to oxygen levels
GO:0006915	7	2154	0.0121138	apoptotic process
GO:0050433	3	92	0.0121969	regulation of catecholamine secretion
GO:0006801	3	94	0.0130131	superoxide metabolic process
GO:1902305	3	94	0.0130131	regulation of sodium ion transmembrane transport
GO:0051270	7	2182	0.0132	regulation of cellular component movement
GO:0006874	5	749	0.0134075	cellular calcium ion homeostasis
GO:0009063	4	331	0.0136099	cellular amino acid catabolic process
GO:0043436	8	3244	0.0140434	oxoacid metabolic process
GO:0008202	5	760	0.014392	steroid metabolic process
GO:0044092	8	3258	0.0145066	negative regulation of molecular function
GO:0055080	6	1386	0.0146041	cation homeostasis
GO:2000379	3	98	0.0147523	positive regulation of reactive oxygen species metabolic process
GO:0030097	4	338	0.0147814	hemopoiesis
GO:0001558	6	1390	0.0148483	regulation of cell growth
GO:0040012	7	2224	0.0149823	regulation of locomotion
GO:0006082	8	3284	0.0154018	organic acid metabolic process
GO:0072503	5	772	0.0155299	cellular divalent inorganic cation homeostasis
GO:0012501	7	2247	0.0160412	programmed cell death
GO:0016486	3	101	0.016153	peptide hormone processing
GO:0032228	3	101	0.016153	regulation of synaptic transmission, GABAergic
GO:0010536	2	11	0.0164724	positive regulation of activation of Janus kinase activity
GO:0055074	5	790	0.0173684	calcium ion homeostasis
GO:0055082	6	1433	0.0176902	cellular chemical homeostasis
GO:0002684	7	2287	0.0180324	positive regulation of immune system process
GO:0006805	4	356	0.0181359	xenobiotic metabolic process
GO:0042531	3	106	0.0186782	positive regulation of tyrosine phosphorylation of STAT protein
GO:0072657	4	360	0.018952	protein localization to membrane
GO:0040008	7	2308	0.0191583	regulation of growth
GO:0046189	3	108	0.0197571	phenol-containing compound biosynthetic process
GO:0002685	4	365	0.0200099	regulation of leukocyte migration
GO:0042391	5	824	0.0213051	regulation of membrane potential
GO:0051098	5	826	0.0215569	regulation of binding

Table 5: Overrepresented terms with the network-based enrichment. Only terms not detected with the standard method.

GO Term	N1	N2	P-value	Description
GO:0050778	6	1484	0.0216218	positive regulation of immune response
GO:0072507	5	827	0.0216835	divalent inorganic cation homeostasis
GO:0006275	4	373	0.0217924	regulation of DNA replication
GO:0045807	4	375	0.0222559	positive regulation of endocytosis
GO:0071900	6	1492	0.0222986	regulation of protein serine/threonine kinase activity
GO:0010959	5	835	0.0227183	regulation of metal ion transport
GO:0050776	7	2377	0.023282	regulation of immune response
GO:0038166	2	13	0.0233507	angiotensin-activated signaling pathway
GO:0006935	5	841	0.0235198	chemotaxis
GO:0042330	5	841	0.0235198	taxis
GO:0034612	4	381	0.02369	response to tumor necrosis factor
GO:0071705	6	1511	0.0239754	nitrogen compound transport
GO:0032496	5	847	0.0243431	response to lipopolysaccharide
GO:0007568	5	848	0.0244825	aging
GO:0045428	3	117	0.025123	regulation of nitric oxide biosynthetic process
GO:0032940	6	1530	0.0257541	secretion by cell
GO:0031281	3	119	0.0264332	positive regulation of cyclase activity
GO:0032768	3	119	0.0264332	regulation of monooxygenase activity
GO:0010534	2	14	0.0272364	regulation of activation of JAK2 kinase activity
GO:0045908	2	14	0.0272364	negative regulation of vasodilation
GO:0050801	6	1555	0.0282567	ion homeostasis
GO:0051251	5	874	0.0283327	positive regulation of lymphocyte activation
GO:0055114	8	3568	0.0287098	oxidation-reduction process
GO:0007162	4	404	0.0298268	negative regulation of cell adhesion
GO:0006629	8	3598	0.0305659	lipid metabolic process
GO:0045989	2	15	0.0314197	positive regulation of striated muscle contraction
GO:0009967	8	3621	0.0320577	positive regulation of signal transduction
GO:0043269	6	1590	0.0320905	regulation of ion transport
GO:0006584	3	128	0.032888	catecholamine metabolic process
GO:0008015	3	128	0.032888	blood circulation
GO:0009712	3	128	0.032888	catechol-containing compound metabolic process
GO:0040018	3	128	0.032888	positive regulation of multicellular organism growth
GO:0048260	3	128	0.032888	positive regulation of receptor-mediated endocytosis
GO:0045597	7	2514	0.0336993	positive regulation of cell differentiation
GO:0042509	3	130	0.0344508	regulation of tyrosine phosphorylation of STAT protein
GO:0031347	6	1612	0.0347102	regulation of defense response
GO:0050863	5	912	0.0347975	regulation of T cell activation
GO:0000187	4	421	0.0350644	activation of MAPK activity
GO:0007202	3	131	0.0352503	activation of phospholipase C activity
GO:0010533	2	16	0.0359002	regulation of activation of Janus kinase activity
GO:0002237	5	918	0.035916	response to molecule of bacterial origin
GO:0055117	3	132	0.0360621	regulation of cardiac muscle contraction
GO:0051349	3	133	0.036886	positive regulation of lyase activity
GO:0045860	6	1636	0.0377639	positive regulation of protein kinase activity
GO:0043405	5	930	0.0382374	regulation of MAP kinase activity
GO:0007266	3	135	0.0385707	Rho protein signal transduction
GO:0046427	3	136	0.039432	positive regulation of JAK-STAT cascade
GO:0048661	3	137	0.0403056	positive regulation of smooth muscle cell proliferation
GO:0002696	5	941	0.0404672	positive regulation of leukocyte activation
GO:0048585	8	3759	0.0423832	negative regulation of response to stimulus
GO:0033993	7	2604	0.042473	response to lipid
GO:1902930	3	141	0.0439281	regulation of alcohol biosynthetic process
GO:0015711	5	964	0.0454582	organic anion transport
GO:0051172	8	3798	0.045771	negative regulation of nitrogen compound metabolic process
GO:0010647	8	3801	0.0460408	positive regulation of cell communication
GO:0033674	6	1706	0.0479433	positive regulation of kinase activity
GO:0031348	4	457	0.0483603	negative regulation of defense response
GO:0051480	4	457	0.0483603	cytosolic calcium ion homeostasis

Table 6: Overrepresented terms with the network-based enrichment. Only terms not detected with the standard method.
