# Supplementary material for: NET-GE: a novel NETwork-based Gene Enrichment for detecting biological processes associated to Mendelian diseases
Source: BMC Genomics. 2015 Jun 18;16(Suppl 8):S6. doi: 10.1186/1471-2164-16-S8-S6 (PMC4480278; doi:10.1186/1471-2164-16-S8-S6)
Supplement: Additional file 3 — Detailed results for the OMIM-derived benchmark set. The archive contains pdf documents listing the enriched terms for each one of the 244 diseases in the OMIM-derived benchmark set. [file 1471-2164-16-S8-S6-S3.tgz › SUPPMAT/OMIM145900.pdf]

#145900 HYPERTROPHIC NEUROPATHY OF DEJERINE-SOTTAS

OMIM Gene ID	HGNC	UniProtAC
129010	EGR2	P11161
159440	MPZ	P25189
601097	PMP22	Q01453
605725	PRX	Q9BXM0

Table 1: OMIM - UniProtAC mapping

Legend

- N1: #input proteins associated to the significant GO term
- N2: #proteins associated to the significant GO term
- P-value: Bonferroni-corrected p-value of Fisher's exact test
- *red*: go terms not related to the input proteins
- *blue*: go terms related to the input proteins (enriched uniquely by network-based method)
- *green*: go terms ancestors of terms enriched with the standard method (enriched uniquely by network-based method)

1 Standard enrichment

GO Term	N1	N2	P-value	Description
GO:0008219	4	1106	0.000136426	cell death
GO:0016265	4	1117	0.000141943	death
GO:0007422	2	48	0.00176456	peripheral nervous system development
GO:0007272	2	133	0.0136904	ensheathment of neurons
GO:0008366	2	133	0.0136904	axon ensheathment
GO:0021594	1	1	0.0197123	rhombomere formation
GO:0021660	1	1	0.0197123	rhombomere 3 formation
GO:0021666	1	1	0.0197123	rhombomere 5 formation

Table 2: Overrepresented GO terms with the standard enrichment

2 Network-based enrichment

GO Term	N1	N2	P-value	Description
GO:0034331	2	28	0.00151879	cell junction maintenance
GO:0043954	2	50	0.00491765	cellular component maintenance
GO:0048731	4	2612	0.0140267	system development
GO:0051098	3	826	0.02218	regulation of binding
GO:0030216	2	142	0.0400406	keratinocyte differentiation

Table 3: Overrepresented terms with the network-based enrichment. Only terms not detected with the standard method.
