# Supplementary material for: NET-GE: a novel NETwork-based Gene Enrichment for detecting biological processes associated to Mendelian diseases
Source: BMC Genomics. 2015 Jun 18;16(Suppl 8):S6. doi: 10.1186/1471-2164-16-S8-S6 (PMC4480278; doi:10.1186/1471-2164-16-S8-S6)
Supplement: Additional file 3 — Detailed results for the OMIM-derived benchmark set. The archive contains pdf documents listing the enriched terms for each one of the 244 diseases in the OMIM-derived benchmark set. [file 1471-2164-16-S8-S6-S3.tgz › SUPPMAT/OMIM146200.pdf]

# #146200 HYPOPARATHYROIDISM, FAMILIAL ISOLATED; FIH

| OMIM Gene ID | HGNC | UniProtAC |
|---|---|---|
| 168450 | PTH | P01270 |
| 603716 | GCM2 | O75603 |

Table 1: OMIM - UniProtAC mapping

Legend

- N1: #input proteins associated to the significant GO term
- N2: #proteins associated to the significant GO term
- P-value: Bonferroni-corrected p-value of Fisher's exact test
- *red*: go terms not related to the input proteins
- *blue*: go terms related to the input proteins (enriched uniquely by network-based method)
- *green*: go terms ancestors of terms enriched with the standard method (enriched uniquely by network-based method)

# 1 Standard enrichment

| GO Term | N1 | N2 | P-value | Description |
|---|---|---|---|---|
| GO:0006874 | 2 | 359 | 0.022285 | cellular calcium ion homeostasis |
| GO:0055074 | 2 | 373 | 0.0240596 | calcium ion homeostasis |
| GO:0072503 | 2 | 373 | 0.0240596 | cellular divalent inorganic cation homeostasis |
| GO:0072507 | 2 | 398 | 0.0273975 | divalent inorganic cation homeostasis |
| GO:0006875 | 2 | 503 | 0.0437832 | cellular metal ion homeostasis |
| GO:0030003 | 2 | 536 | 0.0497226 | cellular cation homeostasis |

Table 2: Overrepresented GO terms with the standard enrichment

# 2 Network-based enrichment

*No novel enriched terms*
