# Supplementary material for: NET-GE: a novel NETwork-based Gene Enrichment for detecting biological processes associated to Mendelian diseases
Source: BMC Genomics. 2015 Jun 18;16(Suppl 8):S6. doi: 10.1186/1471-2164-16-S8-S6 (PMC4480278; doi:10.1186/1471-2164-16-S8-S6)
Supplement: Additional file 3 — Detailed results for the OMIM-derived benchmark set. The archive contains pdf documents listing the enriched terms for each one of the 244 diseases in the OMIM-derived benchmark set. [file 1471-2164-16-S8-S6-S3.tgz › SUPPMAT/OMIM147050.pdf]

#147050 IgE RESPONSIVENESS, ATOPIC; IGER

OMIM Gene ID	HGNC	UniProtAC
147138	MS4A2	Q01362
147781	IL4R	P24394
173610	SELP	P16109
601690	PLA2G7	Q13093
605010	SPINK5	Q9NQ38
605383	IL21R	Q9HBE5
606518	HAVCR1	Q96D42
607796	PHF11	Q9UIL8

Table 1: OMIM - UniProtAC mapping

Legend

- N1: #input proteins associated to the significant GO term
- N2: #proteins associated to the significant GO term
- P-value: Bonferroni-corrected p-value of Fisher's exact test
- *red*: go terms not related to the input proteins
- *blue*: go terms related to the input proteins (enriched uniquely by network-based method)
- *green*: go terms ancestors of terms enriched with the standard method (enriched uniquely by network-based method)

1 Standard enrichment

GO Term	N1	N2	P-value	Description
GO:0050865	4	624	0.00190161	regulation of cell activation
GO:0002682	5	1758	0.00419833	regulation of immune system process
GO:0002684	4	1093	0.0172629	positive regulation of immune system process
GO:0050867	3	380	0.0211361	positive regulation of cell activation

Table 2: Overrepresented GO terms with the standard enrichment

2 Network-based enrichment

GO Term	N1	N2	P-value	Description
GO:0050729	3	261	0.0103057	positive regulation of inflammatory response
GO:0050776	5	2377	0.0217005	regulation of immune response
GO:1903036	3	341	0.0228797	positive regulation of response to wounding
GO:0006954	4	1314	0.0485826	inflammatory response

Table 3: Overrepresented terms with the network-based enrichment. Only terms not detected with the standard method.
