# Supplementary material for: NET-GE: a novel NETwork-based Gene Enrichment for detecting biological processes associated to Mendelian diseases
Source: BMC Genomics. 2015 Jun 18;16(Suppl 8):S6. doi: 10.1186/1471-2164-16-S8-S6 (PMC4480278; doi:10.1186/1471-2164-16-S8-S6)
Supplement: Additional file 3 — Detailed results for the OMIM-derived benchmark set. The archive contains pdf documents listing the enriched terms for each one of the 244 diseases in the OMIM-derived benchmark set. [file 1471-2164-16-S8-S6-S3.tgz › SUPPMAT/OMIM149730.pdf]

#149730 LACRIMOAURICULODENTODIGITAL SYNDROME; LADD

OMIM Gene ID	HGNC	UniProtAC
134934	FGFR3	P22607
176943	FGFR2	P21802
602115	FGF10	O15520

Table 1: OMIM - UniProtAC mapping

Legend

- N1: #input proteins associated to the significant GO term
- N2: #proteins associated to the significant GO term
- P-value: Bonferroni-corrected p-value of Fisher's exact test
- *red*: go terms not related to the input proteins
- *blue*: go terms related to the input proteins (enriched uniquely by network-based method)
- *green*: go terms ancestors of terms enriched with the standard method (enriched uniquely by network-based method)

1 Standard enrichment

GO Term	N1	N2	P-value	Description
GO:0090263	3	87	8.38704e-06	positive regulation of canonical Wnt signaling pathway
GO:0060595	2	3	8.95878e-06	fibroblast growth factor receptor signaling pathway involved in mammary gland specification
GO:0060615	2	3	8.95878e-06	mammary gland bud formation
GO:0060667	2	3	8.95878e-06	branch elongation involved in salivary gland morphogenesis
GO:0060915	2	3	8.95878e-06	mesenchymal cell differentiation involved in lung development
GO:0008589	3	118	2.11202e-05	regulation of smoothened signaling pathway
GO:0030177	3	125	2.51425e-05	positive regulation of Wnt signaling pathway
GO:0060560	3	147	4.10403e-05	developmental growth involved in morphogenesis
GO:0070374	3	150	4.36225e-05	positive regulation of ERK1 and ERK2 cascade
GO:0048015	3	182	7.81977e-05	phosphatidylinositol-mediated signaling
GO:0048017	3	182	7.81977e-05	inositol lipid-mediated signaling
GO:0050679	3	188	8.6235e-05	positive regulation of epithelial cell proliferation
GO:0008286	3	195	9.62865e-05	insulin receptor signaling pathway
GO:0007173	3	202	0.00010709	epidermal growth factor receptor signaling pathway
GO:0038127	3	205	0.000111957	ERBB signaling pathway
GO:0008543	3	211	0.00012213	fibroblast growth factor receptor signaling pathway
GO:0030916	2	10	0.000134365	otic vesicle formation
GO:0060601	2	10	0.000134365	lateral sprouting from an epithelium
GO:0045165	3	222	0.000142344	cell fate commitment
GO:0060828	3	225	0.00014822	regulation of canonical Wnt signaling pathway
GO:0070372	3	231	0.000160452	regulation of ERK1 and ERK2 cascade
GO:0032808	2	11	0.000164221	lacrimal gland development
GO:0044344	3	237	0.000173341	cellular response to fibroblast growth factor stimulus
GO:0071774	3	243	0.0001869	response to fibroblast growth factor
GO:0060174	2	12	0.000197062	limb bud formation
GO:0060664	2	12	0.000197062	epithelial cell proliferation involved in salivary gland morphogenesis
GO:0060449	2	13	0.000232887	bud elongation involved in lung branching
GO:0032869	3	270	0.000256698	cellular response to insulin stimulus
GO:0060428	2	14	0.000271697	lung epithelium development
GO:0048011	3	276	0.000274261	neurotrophin TRK receptor signaling pathway
GO:0038179	3	285	0.000302079	neurotrophin signaling pathway
GO:0038095	3	294	0.000331718	Fc-epsilon receptor signaling pathway
GO:0048589	3	296	0.000338557	developmental growth
GO:0070307	2	16	0.00035827	lens fiber cell development
GO:0030111	3	325	0.000448541	regulation of Wnt signaling pathway
GO:0038093	3	350	0.000560589	Fc receptor signaling pathway
GO:0010948	3	358	0.00060003	negative regulation of cell cycle process
GO:0050678	3	361	0.000615284	regulation of epithelial cell proliferation
GO:0060429	3	368	0.000651878	epithelium development
GO:0071375	3	372	0.000673427	cellular response to peptide hormone stimulus
GO:0032868	3	376	0.000695444	response to insulin
GO:0048546	2	23	0.000755255	digestive tract morphogenesis
GO:1901653	3	391	0.000782282	cellular response to peptide
GO:0060602	2	24	0.000823901	branch elongation of an epithelium
GO:0003401	2	29	0.00121187	axis elongation
GO:0002768	3	486	0.00150451	immune response-regulating cell surface receptor signaling pathway
GO:0002009	3	492	0.00156104	morphogenesis of an epithelium
GO:0048557	2	33	0.00157592	embryonic digestive tract morphogenesis
GO:0007346	3	518	0.00182239	regulation of mitotic cell cycle
GO:0048762	2	36	0.00188025	mesenchymal cell differentiation
GO:0043410	3	539	0.0020536	positive regulation of MAPK cascade
GO:0043434	3	567	0.00239122	response to peptide hormone
GO:0040007	3	569	0.00241665	growth
GO:0031069	2	41	0.00244709	hair follicle morphogenesis
GO:0048729	3	578	0.00253336	tissue morphogenesis
GO:0071417	3	588	0.00266738	cellular response to organonitrogen compound
GO:0060349	2	43	0.0026947	bone morphogenesis
GO:1901652	3	600	0.00283433	response to peptide
GO:0002764	3	615	0.00305264	immune response-regulating signaling pathway
GO:0060740	2	48	0.00336583	prostate gland epithelium morphogenesis

Table 2: Overrepresented GO terms with the standard enrichment

GO Term	N1	N2	P-value	Description
GO:1901699	3	645	0.00352231	cellular response to nitrogen compound
GO:0016331	2	51	0.00380426	morphogenesis of embryonic epithelium
GO:0010564	3	662	0.00380868	regulation of cell cycle process
GO:0045839	2	53	0.00411144	negative regulation of mitosis
GO:0032870	3	716	0.00482046	cellular response to hormone stimulus
GO:0035019	2	60	0.00528037	somatic stem cell maintenance
GO:0030282	2	61	0.00545926	bone mineralization
GO:0071363	3	762	0.00581198	cellular response to growth factor stimulus
GO:0070848	3	793	0.00655159	response to growth factor
GO:0043408	3	794	0.00657644	regulation of MAPK cascade
GO:0007169	3	798	0.00667645	transmembrane receptor protein tyrosine kinase signaling pathway
GO:0045087	3	825	0.00737821	innate immune response
GO:0007267	3	859	0.00832976	cell-cell signaling
GO:0051784	2	77	0.00872637	negative regulation of nuclear division
GO:0023052	3	913	0.0100036	signaling
GO:0044700	3	913	0.0100036	single organism signaling
GO:0060688	2	83	0.0101479	regulation of morphogenesis of a branching structure
GO:0008285	3	920	0.0102357	negative regulation of cell proliferation
GO:0008283	3	940	0.0109186	cell proliferation
GO:0048863	2	91	0.0122098	stem cell differentiation
GO:0009888	3	984	0.0125265	tissue development
GO:0010518	2	93	0.0127549	positive regulation of phospholipase activity
GO:1902533	3	1008	0.0134667	positive regulation of intracellular signal transduction
GO:0031214	2	97	0.0138809	biomineral tissue development
GO:0009880	2	99	0.0144618	embryonic pattern specification
GO:0022404	2	99	0.0144618	molting cycle process
GO:0022405	2	99	0.0144618	hair cycle process
GO:0010517	2	104	0.0159659	regulation of phospholipase activity
GO:1901701	3	1086	0.0168446	cellular response to oxygen-containing compound
GO:0007167	3	1091	0.0170785	enzyme linked receptor protein signaling pathway
GO:0010243	3	1094	0.0172199	response to organonitrogen compound
GO:0060193	2	108	0.0172227	positive regulation of lipase activity
GO:0008284	3	1101	0.0175529	positive regulation of cell proliferation
GO:0007154	3	1103	0.0176489	cell communication
GO:0050673	2	111	0.0181963	epithelial cell proliferation
GO:0042327	3	1129	0.0189277	positive regulation of phosphorylation
GO:0050776	3	1167	0.0209058	regulation of immune response
GO:1901698	3	1186	0.0219445	response to nitrogen compound
GO:0051726	3	1232	0.0246006	regulation of cell cycle
GO:0010562	3	1255	0.0260054	positive regulation of phosphorus metabolic process
GO:0045937	3	1255	0.0260054	positive regulation of phosphate metabolic process
GO:0009725	3	1273	0.0271414	response to hormone
GO:0071495	3	1291	0.02831	cellular response to endogenous stimulus
GO:0007088	2	139	0.0285725	regulation of mitosis
GO:0051094	3	1326	0.0306774	positive regulation of developmental process
GO:0002064	2	153	0.0346323	epithelial cell development
GO:0060191	2	155	0.0355454	regulation of lipase activity
GO:0006955	3	1414	0.0372047	immune response
GO:0050680	2	159	0.0374071	negative regulation of epithelial cell proliferation
GO:0051345	3	1431	0.0385638	positive regulation of hydrolase activity
GO:0019827	2	162	0.0388345	stem cell maintenance
GO:0045787	2	165	0.0402886	positive regulation of cell cycle
GO:0042476	2	169	0.0422687	odontogenesis
GO:0009967	3	1548	0.048825	positive regulation of signal transduction

Table 3: Overrepresented GO terms with the standard enrichment

2 Network-based enrichment

GO Term	N1	N2	P-value	Description
GO:0001958	3	93	1.86369e-05	endochondral ossification
GO:0036075	3	93	1.86369e-05	replacement ossification
GO:0060485	3	161	9.80403e-05	mesenchyme development
GO:0061144	2	7	0.000100463	alveolar secondary septum development
GO:0072089	3	169	0.000113495	stem cell proliferation
GO:0010838	2	10	0.000215264	positive regulation of keratinocyte proliferation
GO:0060501	2	10	0.000215264	positive regulation of epithelial cell proliferation involved in lung morphogenesis
GO:0090080	2	10	0.000215264	positive regulation of MAPKKK cascade by fibroblast growth factor receptor signaling pathway
GO:0051781	3	266	0.000445445	positive regulation of cell division
GO:0046620	3	293	0.000595946	regulation of organ growth
GO:0035264	3	305	0.00067248	multicellular organism growth
GO:0035988	2	19	0.000817855	chondrocyte proliferation
GO:2000794	2	19	0.000817855	regulation of epithelial cell proliferation involved in lung morphogenesis
GO:0001957	2	22	0.00110475	intramembranous ossification
GO:0036072	2	22	0.00110475	direct ossification
GO:0060363	2	23	0.00120995	cranial suture morphogenesis
GO:0032355	3	374	0.00124219	response to estradiol
GO:0010463	2	25	0.00143466	mesenchymal cell proliferation
GO:0008595	2	29	0.00194142	anterior/posterior axis specification, embryo
GO:0030949	2	35	0.00284484	positive regulation of vascular endothelial growth factor receptor signaling pathway
GO:0060113	2	36	0.00301212	inner ear receptor cell differentiation
GO:0050731	3	503	0.00302812	positive regulation of peptidyl-tyrosine phosphorylation
GO:0000904	3	542	0.00379012	cell morphogenesis involved in differentiation
GO:0060445	2	41	0.00392015	branching involved in salivary gland morphogenesis
GO:0003148	2	42	0.00411607	outflow tract septum morphogenesis
GO:0097094	2	42	0.00411607	craniofacial suture morphogenesis
GO:0048638	3	605	0.0052744	regulation of developmental growth
GO:0023014	3	653	0.00663445	signal transduction by phosphorylation
GO:0050730	3	663	0.00694442	regulation of peptidyl-tyrosine phosphorylation
GO:0003281	2	56	0.00736002	ventricular septum development
GO:0048534	3	682	0.00755968	hematopoietic or lymphoid organ development
GO:0002067	2	58	0.00789976	glandular epithelial cell differentiation
GO:0021884	2	62	0.00903647	forebrain neuron development
GO:0060045	2	68	0.0108844	positive regulation of cardiac muscle cell proliferation
GO:0045879	2	69	0.0112091	negative regulation of smoothened signaling pathway
GO:0030947	2	71	0.0118728	regulation of vascular endothelial growth factor receptor signaling pathway
GO:0051302	3	796	0.0120272	regulation of cell division
GO:0000902	3	800	0.0122096	cell morphogenesis
GO:0045786	3	824	0.0133432	negative regulation of cell cycle
GO:0006935	3	841	0.0141873	chemotaxis
GO:0042330	3	841	0.0141873	taxis
GO:0016337	3	900	0.0173917	single organismal cell-cell adhesion
GO:0045686	2	86	0.0174576	negative regulation of glial cell differentiation
GO:0003279	2	87	0.017868	cardiac septum development
GO:0030182	3	911	0.0180379	neuron differentiation
GO:0010453	2	89	0.0187031	regulation of cell fate commitment
GO:0000578	2	93	0.0204304	embryonic axis specification
GO:0098602	3	981	0.0225289	single organism cell adhesion
GO:0060043	2	99	0.0231641	regulation of cardiac muscle cell proliferation
GO:0045931	2	100	0.0236363	positive regulation of mitotic cell cycle
GO:0021954	2	102	0.0245951	central nervous system neuron development
GO:0048701	2	102	0.0245951	embryonic cranial skeleton morphogenesis
GO:0048732	3	1014	0.0248823	gland development
GO:0007399	3	1018	0.0251782	nervous system development
GO:0048608	3	1062	0.0285897	reproductive structure development
GO:0002053	2	113	0.0302085	positive regulation of mesenchymal cell proliferation
GO:0014014	2	116	0.0318392	negative regulation of gliogenesis
GO:0055010	2	116	0.0318392	ventricular cardiac muscle tissue morphogenesis
GO:0030855	3	1135	0.0349062	epithelial cell differentiation
GO:0009948	2	122	0.0352289	anterior/posterior axis specification

Table 4: Overrepresented terms with the network-based enrichment. Only terms not detected with the standard method.

GO Term	N1	N2	P-value	Description
GO:0055021	2	130	0.0400145	regulation of cardiac muscle tissue growth
GO:0010464	2	132	0.0412583	regulation of mesenchymal cell proliferation
GO:0001657	2	133	0.0418874	ureteric bud development
GO:0002062	2	133	0.0418874	chondrocyte differentiation
GO:0007411	3	1242	0.0457487	axon guidance
GO:0097485	3	1243	0.0458593	neuron projection guidance
GO:0001837	2	140	0.0464237	epithelial to mesenchymal transition
GO:0051249	3	1264	0.0482251	regulation of lymphocyte activation

Table 5: Overrepresented terms with the network-based enrichment. Only terms not detected with the standard method.
