# Supplementary material for: NET-GE: a novel NETwork-based Gene Enrichment for detecting biological processes associated to Mendelian diseases
Source: BMC Genomics. 2015 Jun 18;16(Suppl 8):S6. doi: 10.1186/1471-2164-16-S8-S6 (PMC4480278; doi:10.1186/1471-2164-16-S8-S6)
Supplement: Additional file 3 — Detailed results for the OMIM-derived benchmark set. The archive contains pdf documents listing the enriched terms for each one of the 244 diseases in the OMIM-derived benchmark set. [file 1471-2164-16-S8-S6-S3.tgz › SUPPMAT/OMIM158320.pdf]

#158320 MUIR-TORRE SYNDROME; MRTES

OMIM Gene ID	HGNC	UniProtAC
120436	MLH1	P40692
609309	MSH2	P43246

Table 1: OMIM - UniProtAC mapping

Legend

- N1: #input proteins associated to the significant GO term
- N2: #proteins associated to the significant GO term
- P-value: Bonferroni-corrected p-value of Fisher's exact test
- *red*: go terms not related to the input proteins
- *blue*: go terms related to the input proteins (enriched uniquely by network-based method)
- *green*: go terms ancestors of terms enriched with the standard method (enriched uniquely by network-based method)

1 Standard enrichment

GO Term	N1	N2	P-value	Description
GO:0002566	2	18	6.14365e-05	somatic diversification of immune receptors via somatic mutation
GO:0016446	2	18	6.14365e-05	somatic hypermutation of immunoglobulin genes
GO:0045910	2	28	0.000151784	negative regulation of DNA recombination
GO:0002204	2	32	0.000199167	somatic recombination of immunoglobulin genes involved in immune response
GO:0002208	2	32	0.000199167	somatic diversification of immunoglobulins involved in immune response
GO:0045190	2	32	0.000199167	isotype switching
GO:0016447	2	44	0.000379862	somatic recombination of immunoglobulin gene segments
GO:0016445	2	47	0.000434071	somatic diversification of immunoglobulins
GO:0002312	2	51	0.000511971	B cell activation involved in immune response
GO:0002562	2	63	0.000784218	somatic diversification of immune receptors via germline recombination within a single locus
GO:0016444	2	63	0.000784218	somatic cell DNA recombination
GO:0002200	2	66	0.000861315	somatic diversification of immune receptors
GO:0006298	2	66	0.000861315	mismatch repair
GO:0000018	2	79	0.00123716	regulation of DNA recombination
GO:0051053	2	108	0.00232013	negative regulation of DNA metabolic process
GO:0002285	2	121	0.00291523	lymphocyte activation involved in immune response
GO:0008630	2	126	0.00316216	intrinsic apoptotic signaling pathway in response to DNA damage
GO:1903046	2	166	0.00549915	meiotic cell cycle process
GO:0002263	2	170	0.00576819	cell activation involved in immune response
GO:0002366	2	170	0.00576819	leukocyte activation involved in immune response
GO:0006302	2	175	0.00611354	double-strand break repair
GO:0042113	2	186	0.00690859	B cell activation
GO:0007281	2	218	0.00949775	germ cell development
GO:0097193	2	246	0.0121006	intrinsic apoptotic signaling pathway
GO:0045128	1	1	0.0151551	negative regulation of reciprocal meiotic recombination
GO:0006310	2	294	0.017295	DNA recombination
GO:0051276	2	326	0.0212719	chromosome organization
GO:0051052	2	374	0.0280082	regulation of DNA metabolic process
GO:0022412	2	398	0.0317234	cellular process involved in reproduction in multicellular organism
GO:0097190	2	439	0.0386049	apoptotic signaling pathway
GO:0000710	1	3	0.0454643	meiotic mismatch repair
GO:0046649	2	484	0.0469352	lymphocyte activation

Table 2: Overrepresented GO terms with the standard enrichment

2 Network-based enrichment

GO Term	N1	N2	P-value	Description
GO:0000712	2	28	0.000329686	resolution of meiotic recombination intermediates
GO:0051307	2	30	0.000379401	meiotic chromosome separation
GO:0007129	2	33	0.000460514	synapsis
GO:0051304	2	46	0.000902713	chromosome separation
GO:0070192	2	101	0.00440454	chromosome organization involved in meiosis
GO:0007292	2	105	0.00476213	female gamete generation
GO:0000724	2	150	0.00974665	double-strand break repair via homologous recombination
GO:0000725	2	151	0.00987752	recombinational repair

Table 3: Overrepresented terms with the network-based enrichment. Only terms not detected with the standard method.
