# Supplementary material for: NET-GE: a novel NETwork-based Gene Enrichment for detecting biological processes associated to Mendelian diseases
Source: BMC Genomics. 2015 Jun 18;16(Suppl 8):S6. doi: 10.1186/1471-2164-16-S8-S6 (PMC4480278; doi:10.1186/1471-2164-16-S8-S6)
Supplement: Additional file 3 — Detailed results for the OMIM-derived benchmark set. The archive contains pdf documents listing the enriched terms for each one of the 244 diseases in the OMIM-derived benchmark set. [file 1471-2164-16-S8-S6-S3.tgz › SUPPMAT/OMIM158810-OMIM254090.pdf]

#158810 BETHLEM MYOPATHY
#254090 ULLRICH CONGENITAL MUSCULAR DYSTROPHY; UCMD

OMIM Gene ID	HGNC	UniProtAC
120220	COL6A1	P12109
120240	COL6A2	P12110
120250	COL6A3	P12111

Table 1: OMIM - UniProtAC mapping

Legend

- N1: #input proteins associated to the significant GO term
- N2: #proteins associated to the significant GO term
- P-value: Bonferroni-corrected p-value of Fisher's exact test
- *red*: go terms not related to the input proteins
- *blue*: go terms related to the input proteins (enriched uniquely by network-based method)
- *green*: go terms ancestors of terms enriched with the standard method (enriched uniquely by network-based method)

1 Standard enrichment

GO Term	N1	N2	P-value	Description
GO:0030574	3	78	7.30168e-07	collagen catabolic process
GO:0044243	3	84	9.14524e-07	multicellular organismal catabolic process
GO:0032963	3	96	1.37135e-06	collagen metabolic process
GO:0044259	3	105	1.79921e-06	multicellular organismal macromolecule metabolic process
GO:0044236	3	112	2.18755e-06	multicellular organismal metabolic process
GO:0022617	3	117	2.49669e-06	extracellular matrix disassembly
GO:0070208	2	13	2.82487e-05	protein heterotrimerization
GO:0022411	3	404	0.000104697	cellular component disassembly
GO:0007411	3	476	0.000171436	axon guidance
GO:0097485	3	477	0.000172521	neuron projection guidance
GO:0030198	3	486	0.000182494	extracellular matrix organization
GO:0043062	3	487	0.000183625	extracellular structure organization
GO:0070206	2	40	0.000282353	protein trimerization
GO:0007155	3	1407	0.00444611	cell adhesion
GO:0022610	3	1410	0.00447462	biological adhesion
GO:0009749	2	171	0.00524935	response to glucose
GO:0051291	2	177	0.0056247	protein heterooligomerization
GO:0009746	2	183	0.00601298	response to hexose
GO:0034284	2	197	0.00696919	response to monosaccharide
GO:1901700	3	1851	0.0101284	response to oxygen-containing compound
GO:0009743	2	247	0.0109573	response to carbohydrate
GO:0006928	3	1973	0.0122671	cellular component movement
GO:0044712	3	2063	0.0140245	single-organism catabolic process

Table 2: Overrepresented GO terms with the standard enrichment

2 Network-based enrichment

No novel enriched terms
