# Supplementary material for: NET-GE: a novel NETwork-based Gene Enrichment for detecting biological processes associated to Mendelian diseases
Source: BMC Genomics. 2015 Jun 18;16(Suppl 8):S6. doi: 10.1186/1471-2164-16-S8-S6 (PMC4480278; doi:10.1186/1471-2164-16-S8-S6)
Supplement: Additional file 3 — Detailed results for the OMIM-derived benchmark set. The archive contains pdf documents listing the enriched terms for each one of the 244 diseases in the OMIM-derived benchmark set. [file 1471-2164-16-S8-S6-S3.tgz › SUPPMAT/OMIM162900.pdf]

#162900 NEVUS, EPIDERMAL

OMIM Gene ID	HGNC	UniProtAC
134934	FGFR3	P22607
164790	NRAS	P01111
171834	PIK3CA	P42336
190020	HRAS	P01112

Table 1: OMIM - UniProtAC mapping

Legend

- N1: #input proteins associated to the significant GO term
- N2: #proteins associated to the significant GO term
- P-value: Bonferroni-corrected p-value of Fisher's exact test
- *red*: go terms not related to the input proteins
- *blue*: go terms related to the input proteins (enriched uniquely by network-based method)
- *green*: go terms ancestors of terms enriched with the standard method (enriched uniquely by network-based method)

1 Standard enrichment

GO Term	N1	N2	P-value	Description
GO:0008286	4	195	4.29052e-07	insulin receptor signaling pathway
GO:0007173	4	202	4.9459e-07	epidermal growth factor receptor signaling pathway
GO:0038127	4	205	5.24864e-07	ERBB signaling pathway
GO:0008543	4	211	5.89558e-07	fibroblast growth factor receptor signaling pathway
GO:0044344	4	237	9.41368e-07	cellular response to fibroblast growth factor stimulus
GO:0071774	4	243	1.04103e-06	response to fibroblast growth factor
GO:0032869	4	270	1.59066e-06	cellular response to insulin stimulus
GO:0048011	4	276	1.73768e-06	neurotrophin TRK receptor signaling pathway
GO:0038179	4	285	1.97703e-06	neurotrophin signaling pathway
GO:0038095	4	294	2.24029e-06	Fc-epsilon receptor signaling pathway
GO:0043523	4	300	2.42986e-06	regulation of neuron apoptotic process
GO:1901214	4	348	4.41183e-06	regulation of neuron death
GO:0038093	4	350	4.51458e-06	Fc receptor signaling pathway
GO:0071375	4	372	5.76713e-06	cellular response to peptide hormone stimulus
GO:0032868	4	376	6.02025e-06	response to insulin
GO:1901653	4	391	7.04431e-06	cellular response to peptide
GO:0002768	4	486	1.6865e-05	immune response-regulating cell surface receptor signaling pathway
GO:0043434	4	567	3.12998e-05	response to peptide hormone
GO:0035022	2	4	3.13857e-05	positive regulation of Rac protein signal transduction
GO:0071417	4	588	3.62146e-05	cellular response to organonitrogen compound
GO:1901652	4	600	3.92707e-05	response to peptide
GO:0002764	4	615	4.33581e-05	immune response-regulating signaling pathway
GO:1901699	4	645	5.24815e-05	cellular response to nitrogen compound
GO:0033674	4	656	5.61631e-05	positive regulation of kinase activity
GO:0032870	4	716	7.97668e-05	cellular response to hormone stimulus
GO:0051347	4	751	9.65829e-05	positive regulation of transferase activity
GO:0071363	4	762	0.000102379	cellular response to growth factor stimulus
GO:0070848	4	793	0.000120121	response to growth factor
GO:0007169	4	798	0.000123185	transmembrane receptor protein tyrosine kinase signaling pathway
GO:0045087	4	825	0.000140756	innate immune response
GO:0001934	4	956	0.000254049	positive regulation of protein phosphorylation
GO:0043549	4	1079	0.000412556	regulation of kinase activity
GO:1901701	4	1086	0.000423382	cellular response to oxygen-containing compound
GO:0007167	4	1091	0.000431244	enzyme linked receptor protein signaling pathway
GO:0010243	4	1094	0.000436013	response to organonitrogen compound
GO:0043524	3	216	0.000457236	negative regulation of neuron apoptotic process
GO:0042327	4	1129	0.00049463	positive regulation of phosphorylation
GO:0050776	4	1167	0.000564759	regulation of immune response
GO:1901698	4	1186	0.000602497	response to nitrogen compound
GO:0051338	4	1198	0.000627285	regulation of transferase activity
GO:0031401	4	1213	0.000659341	positive regulation of protein modification process
GO:1901215	3	244	0.000659788	negative regulation of neuron death
GO:0000165	3	249	0.000701289	MAPK cascade
GO:0010562	4	1255	0.000755633	positive regulation of phosphorus metabolic process
GO:0045937	4	1255	0.000755633	positive regulation of phosphate metabolic process
GO:0035020	2	18	0.000799941	regulation of Rac protein signal transduction
GO:0009725	4	1273	0.000799978	response to hormone
GO:0050900	3	265	0.000845696	leukocyte migration
GO:0071495	4	1291	0.000846249	cellular response to endogenous stimulus
GO:0032270	4	1363	0.00105168	positive regulation of cellular protein metabolic process
GO:0006955	4	1414	0.00121834	immune response
GO:0001932	4	1440	0.00131055	regulation of protein phosphorylation
GO:0023014	3	309	0.00134176	signal transduction by phosphorylation
GO:0051247	4	1526	0.0016532	positive regulation of protein metabolic process
GO:0006952	4	1580	0.00190017	defense response
GO:0002682	4	1758	0.00291345	regulation of immune system process
GO:0042325	4	1770	0.00299388	regulation of phosphorylation
GO:0031399	4	1823	0.00336924	regulation of protein modification process
GO:0048169	2	37	0.00347975	regulation of long-term neuronal synaptic plasticity
GO:1901700	4	1851	0.00358123	response to oxygen-containing compound

Table 2: Overrepresented GO terms with the standard enrichment

GO Term	N1	N2	P-value	Description
GO:0060441	2	39	0.00387135	epithelial tube branching involved in lung morphogenesis
GO:0042981	4	1970	0.00459575	regulation of apoptotic process
GO:0043067	4	1982	0.00470884	regulation of programmed cell death
GO:0009719	4	2012	0.0050007	response to endogenous stimulus
GO:0002009	3	492	0.005416	morphogenesis of an epithelium
GO:0010941	4	2079	0.00570136	regulation of cell death
GO:0007596	3	501	0.00571829	blood coagulation
GO:0050817	3	501	0.00571829	coagulation
GO:0032228	2	48	0.00589135	regulation of synaptic transmission, GABAergic
GO:0007599	3	510	0.00603157	hemostasis
GO:0043085	4	2150	0.00652162	positive regulation of catalytic activity
GO:0032268	4	2272	0.00813392	regulation of cellular protein metabolic process
GO:0008542	2	58	0.00863029	visual learning
GO:0048584	4	2308	0.0086622	positive regulation of response to stimulus
GO:0048729	3	578	0.0087743	tissue morphogenesis
GO:0007632	2	63	0.0101948	visual behavior
GO:0002376	4	2446	0.0109289	immune system process
GO:0045860	3	626	0.0111405	positive regulation of protein kinase activity
GO:0048168	2	66	0.0111958	regulation of neuronal synaptic plasticity
GO:0044093	4	2479	0.011531	positive regulation of molecular function
GO:0046579	2	67	0.0115399	positive regulation of Ras protein signal transduction
GO:0071310	4	2482	0.011587	cellular response to organic substance
GO:0035556	4	2537	0.0126494	intracellular signal transduction
GO:0016310	4	2550	0.0129108	phosphorylation
GO:0051057	2	76	0.0148703	positive regulation of small GTPase mediated signal transduction
GO:0050878	3	717	0.0167189	regulation of body fluid levels
GO:0000186	2	83	0.0177513	activation of MAPKK activity
GO:0008306	2	89	0.020423	associative learning
GO:0070887	4	2904	0.0217222	cellular response to chemical stimulus
GO:0051146	2	93	0.0223077	striated muscle cell differentiation
GO:0043408	3	794	0.0226785	regulation of MAPK cascade
GO:0051246	4	2954	0.0232581	regulation of protein metabolic process
GO:0019220	4	2977	0.0239913	regulation of phosphate metabolic process
GO:0051174	4	2996	0.02461	regulation of phosphorus metabolic process
GO:0045596	3	834	0.0262651	negative regulation of cell differentiation
GO:0010604	4	3285	0.0355765	positive regulation of macromolecule metabolic process
GO:0050790	4	3371	0.0394527	regulation of catalytic activity
GO:0031325	4	3418	0.0417005	positive regulation of cellular metabolic process
GO:0010033	4	3487	0.0451726	response to organic substance
GO:0045859	3	1005	0.0458292	regulation of protein kinase activity
GO:1902533	3	1008	0.0462384	positive regulation of intracellular signal transduction

Table 3: Overrepresented GO terms with the standard enrichment

2 Network-based enrichment

GO Term	N1	N2	P-value	Description
GO:0030111	4	814	0.000482996	regulation of Wnt signaling pathway
GO:0010518	3	199	0.00114124	positive regulation of phospholipase activity
GO:0010517	3	233	0.00183448	regulation of phospholipase activity
GO:0060193	3	234	0.00185827	positive regulation of lipase activity
GO:0007411	4	1242	0.00262444	axon guidance
GO:0097485	4	1243	0.00263292	neuron projection guidance
GO:0097191	3	280	0.00318715	extrinsic apoptotic signaling pathway
GO:1903322	3	310	0.00432685	positive regulation of protein modification by small protein conjugation or removal
GO:0048015	3	342	0.00581091	phosphatidylinositol-mediated signaling
GO:0048017	3	342	0.00581091	inositol lipid-mediated signaling
GO:0002064	3	370	0.00735843	epithelial cell development
GO:0060191	3	379	0.00790847	regulation of lipase activity
GO:0046578	3	410	0.0100111	regulation of Ras protein signal transduction
GO:0031396	3	451	0.0133213	regulation of protein ubiquitination
GO:0007265	3	457	0.0138594	Ras protein signal transduction
GO:0050768	3	457	0.0138594	negative regulation of neurogenesis
GO:0035412	2	50	0.0180446	regulation of catenin import into nucleus
GO:0050731	3	503	0.0184716	positive regulation of peptidyl-tyrosine phosphorylation
GO:1903320	3	520	0.0204046	regulation of protein modification by small protein conjugation or removal
GO:0018108	3	523	0.0207591	peptidyl-tyrosine phosphorylation
GO:0018212	3	533	0.0219701	peptidyl-tyrosine modification
GO:0070372	3	536	0.0223423	regulation of ERK1 and ERK2 cascade
GO:0060828	3	550	0.024135	regulation of canonical Wnt signaling pathway
GO:0090287	3	557	0.0250659	regulation of cellular response to growth factor stimulus
GO:0010721	3	609	0.0327383	negative regulation of cell development
GO:0001501	3	620	0.0345389	skeletal system development
GO:0030947	2	71	0.0365741	regulation of vascular endothelial growth factor receptor signaling pathway
GO:0043066	4	2487	0.0422967	negative regulation of apoptotic process
GO:0043069	4	2511	0.0439542	negative regulation of programmed cell death
GO:0048534	3	682	0.0459264	hematopoietic or lymphoid organ development

Table 4: Overrepresented terms with the network-based enrichment. Only terms not detected with the standard method.
