# Supplementary material for: NET-GE: a novel NETwork-based Gene Enrichment for detecting biological processes associated to Mendelian diseases
Source: BMC Genomics. 2015 Jun 18;16(Suppl 8):S6. doi: 10.1186/1471-2164-16-S8-S6 (PMC4480278; doi:10.1186/1471-2164-16-S8-S6)
Supplement: Additional file 3 — Detailed results for the OMIM-derived benchmark set. The archive contains pdf documents listing the enriched terms for each one of the 244 diseases in the OMIM-derived benchmark set. [file 1471-2164-16-S8-S6-S3.tgz › SUPPMAT/OMIM163200.pdf]

#163200 SCHIMMELPENNING-FEUERSTEIN-MIMS SYNDROME; SFM

OMIM Gene ID	HGNC	UniProtAC
190020	HRAS	P01112
190070	KRAS	P01116

Table 1: OMIM - UniProtAC mapping

Legend

- N1: #input proteins associated to the significant GO term
- N2: #proteins associated to the significant GO term
- P-value: Bonferroni-corrected p-value of Fisher's exact test
- *red*: go terms not related to the input proteins
- *blue*: go terms related to the input proteins (enriched uniquely by network-based method)
- *green*: go terms ancestors of terms enriched with the standard method (enriched uniquely by network-based method)

1 Standard enrichment

GO Term	N1	N2	P-value	Description
GO:0035022	2	4	3.46228e-06	positive regulation of Rac protein signal transduction
GO:0035020	2	18	8.82881e-05	regulation of Rac protein signal transduction
GO:0048169	2	37	0.000384313	regulation of long-term neuronal synaptic plasticity
GO:0060441	2	39	0.000427592	epithelial tube branching involved in lung morphogenesis
GO:0032228	2	48	0.000650909	regulation of synaptic transmission, GABAergic
GO:0035176	2	50	0.000706883	social behavior
GO:0051703	2	50	0.000706883	intraspecies interaction between organisms
GO:0008542	2	58	0.000953857	visual learning
GO:0007632	2	63	0.00112697	visual behavior
GO:0048168	2	66	0.00123776	regulation of neuronal synaptic plasticity
GO:0046579	2	67	0.00127585	positive regulation of Ras protein signal transduction
GO:0051057	2	76	0.00164458	positive regulation of small GTPase mediated signal transduction
GO:0000186	2	83	0.00196369	activation of MAPKK activity
GO:0008306	2	89	0.00225971	associative learning
GO:0051146	2	93	0.0024686	striated muscle cell differentiation
GO:0051705	2	116	0.0038489	multi-organism behavior
GO:0007612	2	163	0.00761875	learning
GO:0008286	2	195	0.0109148	insulin receptor signaling pathway
GO:0042692	2	200	0.0114832	muscle cell differentiation
GO:0007265	2	201	0.0115986	Ras protein signal transduction
GO:0007173	2	202	0.0117146	epidermal growth factor receptor signaling pathway
GO:0046578	2	203	0.0118312	regulation of Ras protein signal transduction
GO:0038127	2	205	0.012066	ERBB signaling pathway
GO:0048167	2	210	0.0126633	regulation of synaptic plasticity
GO:0008543	2	211	0.0127844	fibroblast growth factor receptor signaling pathway
GO:0043524	2	216	0.013399	negative regulation of neuron apoptotic process
GO:0044344	2	237	0.0161377	cellular response to fibroblast growth factor stimulus
GO:0071774	2	243	0.0169669	response to fibroblast growth factor
GO:0048754	2	244	0.0171071	branching morphogenesis of an epithelial tube
GO:1901215	2	244	0.0171071	negative regulation of neuron death
GO:0000165	2	249	0.0178169	MAPK cascade
GO:0050900	2	265	0.0201851	leukocyte migration
GO:0032869	2	270	0.0209554	cellular response to insulin stimulus
GO:0043406	2	275	0.0217402	positive regulation of MAP kinase activity
GO:0048011	2	276	0.0218989	neurotrophin TRK receptor signaling pathway
GO:0038179	2	285	0.0233531	neurotrophin signaling pathway
GO:0061138	2	286	0.0235175	morphogenesis of a branching epithelium
GO:0038095	2	294	0.024854	Fc-epsilon receptor signaling pathway
GO:0043523	2	300	0.0258805	regulation of neuron apoptotic process
GO:0001763	2	303	0.0264016	morphogenesis of a branching structure
GO:0023014	2	309	0.0274593	signal transduction by phosphorylation
GO:0007611	2	329	0.0311351	learning or memory
GO:1901214	2	348	0.0348409	regulation of neuron death
GO:0038093	2	350	0.0352431	Fc receptor signaling pathway
GO:0032147	2	357	0.036669	activation of protein kinase activity
GO:0071902	2	357	0.036669	positive regulation of protein serine/threonine kinase activity
GO:0050890	2	365	0.0383332	cognition
GO:0071375	2	372	0.0398197	cellular response to peptide hormone stimulus
GO:0032868	2	376	0.0406818	response to insulin
GO:0043405	2	381	0.0417724	regulation of MAP kinase activity
GO:0050804	2	390	0.0437719	regulation of synaptic transmission
GO:1901653	2	391	0.0439967	cellular response to peptide
GO:0051056	2	404	0.0469748	regulation of small GTPase mediated signal transduction

Table 2: Overrepresented GO terms with the standard enrichment

2 Network-based enrichment

GO Term	N1	N2	P-value	Description
GO:0002548	2	44	0.00220933	monocyte chemotaxis
GO:0019320	2	84	0.00814134	hexose catabolic process
GO:0045055	2	85	0.00833752	regulated secretory pathway
GO:0046365	2	93	0.00999101	monosaccharide catabolic process
GO:0008361	2	104	0.0125086	regulation of cell size
GO:0051353	2	104	0.0125086	positive regulation of oxidoreductase activity
GO:0030593	2	113	0.0147787	neutrophil chemotaxis
GO:0048640	2	122	0.0172378	negative regulation of developmental growth
GO:1990266	2	123	0.0175228	neutrophil migration
GO:0042446	2	141	0.0230508	hormone biosynthetic process
GO:0071621	2	142	0.0233801	granulocyte chemotaxis
GO:0018279	2	145	0.024382	protein N-linked glycosylation via asparagine
GO:0018196	2	146	0.0247206	peptidyl-asparagine modification
GO:0097530	2	154	0.0275138	granulocyte migration
GO:0032330	2	157	0.0285997	regulation of chondrocyte differentiation
GO:0032411	2	158	0.0289664	positive regulation of transporter activity
GO:0002028	2	181	0.0380443	regulation of sodium ion transport
GO:0030072	2	189	0.0414914	peptide hormone secretion
GO:0002790	2	199	0.0460105	peptide secretion
GO:0061035	2	207	0.0497939	regulation of cartilage development

Table 3: Overrepresented terms with the network-based enrichment. Only terms not detected with the standard method.
