# Supplementary material for: NET-GE: a novel NETwork-based Gene Enrichment for detecting biological processes associated to Mendelian diseases
Source: BMC Genomics. 2015 Jun 18;16(Suppl 8):S6. doi: 10.1186/1471-2164-16-S8-S6 (PMC4480278; doi:10.1186/1471-2164-16-S8-S6)
Supplement: Additional file 3 — Detailed results for the OMIM-derived benchmark set. The archive contains pdf documents listing the enriched terms for each one of the 244 diseases in the OMIM-derived benchmark set. [file 1471-2164-16-S8-S6-S3.tgz › SUPPMAT/OMIM164230.pdf]

#164230 OBSESSIVE-COMPULSIVE DISORDER; OCD

**OMIM Gene ID**	**HGNC**	**UniProtAC**
113505	BDNF	P23560
182135	HTR2A	P28223
182138	SLC6A4	P31645

Table 1: OMIM - UniProtAC mapping

Legend

- N1: #input proteins associated to the significant GO term
- N2: #proteins associated to the significant GO term
- P-value: Bonferroni-corrected p-value of Fisher's exact test
- *red*: go terms not related to the input proteins
- *blue*: go terms related to the input proteins (enriched uniquely by network-based method)
- *green*: go terms ancestors of terms enriched with the standard method (enriched uniquely by network-based method)

1 Standard enrichment

GO Term	N1	N2	P-value	Description
GO:0050805	3	54	1.24846e-06	negative regulation of synaptic transmission
GO:0007611	3	329	0.000296019	learning or memory
GO:0050890	3	365	0.000404579	cognition
GO:0050804	3	390	0.000493795	regulation of synaptic transmission
GO:0042310	2	41	0.00155661	vasoconstriction
GO:0044708	3	596	0.00176706	single-organism behavior
GO:0042493	3	633	0.00211764	response to drug
GO:2000178	2	54	0.00271586	negative regulation of neural precursor cell proliferation
GO:0007610	3	806	0.00437612	behavior
GO:0050880	2	90	0.00759615	regulation of blood vessel size
GO:0035150	2	91	0.00776672	regulation of tube size
GO:0050877	3	1063	0.0100479	neurological system process
GO:0003018	2	117	0.0128646	vascular process in circulatory system
GO:2000177	2	121	0.0137623	regulation of neural precursor cell proliferation
GO:0014070	3	1205	0.0146414	response to organic cyclic compound
GO:0007613	2	155	0.0226107	memory
GO:0023057	3	1420	0.0239689	negative regulation of signaling
GO:0010648	3	1424	0.0241722	negative regulation of cell communication
GO:0009628	3	1467	0.0264304	response to abiotic stimulus
GO:0003013	2	173	0.0281772	circulatory system process
GO:0003008	3	1588	0.0335299	system process
GO:0014076	1	1	0.0358477	response to fluoxetine
GO:0072347	1	1	0.0358477	response to anesthetic
GO:0007623	2	203	0.0388096	circadian rhythm

Table 2: Overrepresented GO terms with the standard enrichment

2 Network-based enrichment

GO Term	N1	N2	P-value	Description
GO:0032526	3	457	0.00243377	response to retinoic acid
GO:0071229	3	589	0.00521815	cellular response to acid chemical
GO:0051968	2	51	0.00653484	positive regulation of synaptic transmission, glutamatergic
GO:0060999	2	56	0.00789227	positive regulation of dendritic spine development
GO:0007420	3	791	0.0126551	brain development
GO:0003012	3	815	0.0138439	muscle system process
GO:0051962	2	75	0.014216	positive regulation of nervous system development
GO:0051965	2	75	0.014216	positive regulation of synapse assembly
GO:0050927	2	84	0.0178552	positive regulation of positive chemotaxis
GO:0045686	2	86	0.0187201	negative regulation of glial cell differentiation
GO:0050926	2	86	0.0187201	regulation of positive chemotaxis
GO:0044089	2	90	0.020511	positive regulation of cellular component biogenesis
GO:0048511	3	930	0.0205793	rhythmic process
GO:0032228	2	101	0.0258572	regulation of synaptic transmission, GABAergic
GO:0060998	2	105	0.0279542	regulation of dendritic spine development
GO:0097305	3	1084	0.032604	response to alcohol
GO:0014014	2	116	0.0341417	negative regulation of gliogenesis
GO:0035176	2	119	0.0359362	social behavior
GO:0051703	2	119	0.0359362	intraspecies interaction between organisms
GO:0042220	2	127	0.0409456	response to cocaine
GO:0001101	3	1170	0.0410043	response to acid chemical
GO:0045761	2	128	0.0415947	regulation of adenylate cyclase activity
GO:0007268	3	1195	0.0436917	synaptic transmission
GO:0071396	3	1198	0.0440218	cellular response to lipid
GO:0031667	3	1219	0.0463796	response to nutrient levels

Table 3: Overrepresented terms with the network-based enrichment. Only terms not detected with the standard method.
