# Supplementary material for: NET-GE: a novel NETwork-based Gene Enrichment for detecting biological processes associated to Mendelian diseases
Source: BMC Genomics. 2015 Jun 18;16(Suppl 8):S6. doi: 10.1186/1471-2164-16-S8-S6 (PMC4480278; doi:10.1186/1471-2164-16-S8-S6)
Supplement: Additional file 3 — Detailed results for the OMIM-derived benchmark set. The archive contains pdf documents listing the enriched terms for each one of the 244 diseases in the OMIM-derived benchmark set. [file 1471-2164-16-S8-S6-S3.tgz › SUPPMAT/OMIM166710.pdf]

#166710 OSTEOPOROSIS

OMIM Gene ID	HGNC	UniProtAC
114131	CALCR	P30988
120150	COL1A1	P02452
120160	COL1A2	P08123
601769	VDR	P11473
603422	PDLIM4	P50479
603506	LRP5	O75197

Table 1: OMIM - UniProtAC mapping

Legend

- N1: #input proteins associated to the significant GO term
- N2: #proteins associated to the significant GO term
- P-value: Bonferroni-corrected p-value of Fisher's exact test
- *red*: go terms not related to the input proteins
- *blue*: go terms related to the input proteins (enriched uniquely by network-based method)
- *green*: go terms ancestors of terms enriched with the standard method (enriched uniquely by network-based method)

1 Standard enrichment

GO Term	N1	N2	P-value	Description
GO:0009719	5	2012	0.000236939	response to endogenous stimulus
GO:0009887	4	653	0.000242135	organ morphogenesis
GO:0070208	2	13	0.000605253	protein heterotrimerization
GO:0043589	2	15	0.000814674	skin morphogenesis
GO:0001501	3	213	0.000971886	skeletal system development
GO:0009725	4	1273	0.00346609	response to hormone
GO:0010033	5	3487	0.00371254	response to organic substance
GO:0070206	2	40	0.00604385	protein trimerization
GO:0065008	5	3888	0.00639987	regulation of biological quality
GO:0060444	2	59	0.0132444	branching involved in mammary gland duct morphogenesis
GO:1901700	4	1851	0.0153211	response to oxygen-containing compound
GO:0042221	5	4712	0.0167403	response to chemical
GO:0030199	2	76	0.0220412	collagen fibril organization
GO:0071230	2	77	0.0226278	cellular response to amino acid stimulus
GO:0030574	2	78	0.023222	collagen catabolic process
GO:0045935	4	2060	0.0234028	positive regulation of nucleobase-containing compound metabolic process
GO:0051173	4	2108	0.0256359	positive regulation of nitrogen compound metabolic process
GO:0009653	4	2131	0.0267603	anatomical structure morphogenesis
GO:0048545	3	646	0.0269006	response to steroid hormone
GO:0044243	2	84	0.0269484	multicellular organismal catabolic process
GO:0031328	4	2177	0.0291188	positive regulation of cellular biosynthetic process
GO:0034103	2	89	0.0302645	regulation of tissue remodeling
GO:0009891	4	2208	0.0307931	positive regulation of biosynthetic process
GO:0032963	2	96	0.0352285	collagen metabolic process
GO:0050878	3	717	0.0366929	regulation of body fluid levels
GO:0044259	2	105	0.0421613	multicellular organismal macromolecule metabolic process
GO:0044236	2	112	0.0479812	multicellular organismal metabolic process

Table 2: Overrepresented GO terms with the standard enrichment

2 Network-based enrichment

GO Term	N1	N2	P-value	Description
GO:0007568	5	848	6.43358e-05	aging
GO:0001934	6	2371	0.00013396	positive regulation of protein phosphorylation
GO:0048608	5	1062	0.000197583	reproductive structure development
GO:0042327	6	2706	0.000296274	positive regulation of phosphorylation
GO:0001101	5	1170	0.000320058	response to acid chemical
GO:0022603	6	2832	0.000389398	regulation of anatomical structure morphogenesis
GO:0008406	4	424	0.000394353	gonad development
GO:0031401	6	2947	0.000494543	positive regulation of protein modification process
GO:0010562	6	3001	0.000551517	positive regulation of phosphorus metabolic process
GO:0045937	6	3001	0.000551517	positive regulation of phosphate metabolic process
GO:0032270	6	3279	0.00093885	positive regulation of cellular protein metabolic process
GO:0001932	6	3361	0.00108894	regulation of protein phosphorylation
GO:0032940	5	1530	0.00121502	secretion by cell
GO:1902930	3	141	0.00152489	regulation of alcohol biosynthetic process
GO:0051247	6	3557	0.0015304	positive regulation of protein metabolic process
GO:0009967	6	3621	0.00170336	positive regulation of signal transduction
GO:0048592	3	147	0.00172877	eye morphogenesis
GO:0030278	4	644	0.00208655	regulation of ossification
GO:0043627	4	647	0.00212549	response to estrogen
GO:0009612	4	658	0.0022729	response to mechanical stimulus
GO:0010647	6	3801	0.00227934	positive regulation of cell communication
GO:0001763	4	690	0.00274523	morphogenesis of a branching structure
GO:0044087	5	1812	0.00281296	regulation of cellular component biogenesis
GO:0043408	5	1837	0.00301069	regulation of MAPK cascade
GO:0071363	5	1903	0.00358628	cellular response to growth factor stimulus
GO:0046903	5	1910	0.00365213	secretion
GO:0030334	5	1926	0.00380624	regulation of cell migration
GO:0050810	3	198	0.00423238	regulation of steroid biosynthetic process
GO:0070848	5	1987	0.00444198	response to growth factor
GO:0010951	4	804	0.00503876	negative regulation of endopeptidase activity
GO:2000145	5	2039	0.00504818	regulation of cell motility
GO:0090066	4	820	0.00544851	regulation of anatomical structure size
GO:0010466	4	836	0.00588259	negative regulation of peptidase activity
GO:0051270	5	2182	0.00706028	regulation of cellular component movement
GO:0040012	5	2224	0.00775848	regulation of locomotion
GO:0048468	5	2280	0.00877365	cell development
GO:0001525	4	949	0.00972241	angiogenesis
GO:0016525	3	262	0.00980001	negative regulation of angiogenesis
GO:0003006	5	2335	0.00987078	developmental process involved in reproduction
GO:0008285	5	2354	0.0102741	negative regulation of cell proliferation
GO:0003002	4	988	0.0114029	regionalization
GO:0019218	3	278	0.0117023	regulation of steroid metabolic process
GO:1902533	5	2418	0.0117301	positive regulation of intracellular signal transduction
GO:0045859	5	2438	0.0122173	regulation of protein kinase activity
GO:0060429	4	1006	0.0122473	epithelium development
GO:0050678	4	1020	0.0129356	regulation of epithelial cell proliferation
GO:0030335	4	1024	0.0131374	positive regulation of cell migration
GO:0043066	5	2487	0.0134788	negative regulation of apoptotic process
GO:0051222	4	1034	0.0136522	positive regulation of protein transport
GO:0007186	5	2507	0.0140225	G-protein coupled receptor signaling pathway
GO:2000147	4	1042	0.0140748	positive regulation of cell motility
GO:0010243	5	2509	0.0140779	response to organonitrogen compound
GO:0043069	5	2511	0.0141334	negative regulation of programmed cell death
GO:0045597	5	2514	0.014217	positive regulation of cell differentiation
GO:0008584	3	300	0.0146961	male gonad development
GO:1901701	5	2540	0.0149577	cellular response to oxygen-containing compound
GO:0051272	4	1060	0.0150609	positive regulation of cellular component movement
GO:0030326	3	303	0.0151398	embryonic limb morphogenesis
GO:0035113	3	303	0.0151398	embryonic appendage morphogenesis
GO:0009888	5	2570	0.0158501	tissue development

Table 3: Overrepresented terms with the network-based enrichment. Only terms not detected with the standard method.

GO Term	N1	N2	P-value	Description
GO:0016477	5	2574	0.0159723	cell migration
GO:0043549	5	2576	0.0160336	regulation of kinase activity
GO:0033993	5	2604	0.0169122	response to lipid
GO:0048731	5	2612	0.0171701	system development
GO:0060560	3	320	0.0178225	developmental growth involved in morphogenesis
GO:0043086	5	2637	0.0179963	negative regulation of catalytic activity
GO:0030198	4	1111	0.0181345	extracellular matrix organization
GO:0060548	5	2642	0.0181652	negative regulation of cell death
GO:0043062	4	1116	0.0184591	extracellular structure organization
GO:0040017	4	1124	0.0189874	positive regulation of locomotion
GO:0051093	5	2690	0.0198522	negative regulation of developmental process
GO:1901698	5	2693	0.0199616	response to nitrogen compound
GO:0002009	4	1159	0.0214315	morphogenesis of an epithelium
GO:0033043	5	2759	0.0224927	regulation of organelle organization
GO:0035107	3	348	0.0228959	appendage morphogenesis
GO:0035108	3	348	0.0228959	limb morphogenesis
GO:0014070	5	2783	0.0234737	response to organic cyclic compound
GO:0071396	4	1198	0.0244221	cellular response to lipid
GO:0008284	5	2808	0.0245315	positive regulation of cell proliferation
GO:0048870	5	2808	0.0245315	cell motility
GO:0051338	5	2819	0.0250087	regulation of transferase activity
GO:0031667	4	1219	0.0261552	response to nutrient levels
GO:0042476	3	366	0.0266145	odontogenesis
GO:0001649	3	378	0.0293027	osteoblast differentiation
GO:0048593	2	47	0.0301922	camera-type eye morphogenesis
GO:0043410	4	1269	0.0306479	positive regulation of MAPK cascade
GO:0008347	2	49	0.0328402	glial cell migration
GO:0046890	3	401	0.034945	regulation of lipid biosynthetic process
GO:0009991	4	1313	0.0350538	response to extracellular stimulus
GO:0071495	5	3038	0.0361492	cellular response to endogenous stimulus
GO:0080134	5	3072	0.0381841	regulation of response to stress
GO:0008585	2	53	0.0384689	female gonad development
GO:0051346	4	1348	0.0388808	negative regulation of hydrolase activity
GO:0048729	4	1359	0.0401451	tissue morphogenesis
GO:0031099	3	421	0.0403982	regeneration
GO:0002076	2	55	0.0414492	osteoblast development
GO:0051051	4	1375	0.0420381	negative regulation of transport
GO:0040011	5	3142	0.0426594	locomotion
GO:0051094	5	3200	0.0466739	positive regulation of developmental process
GO:0009968	5	3204	0.0469614	negative regulation of signal transduction
GO:0010035	4	1420	0.0477173	response to inorganic substance

Table 4: Overrepresented terms with the network-based enrichment. Only terms not detected with the standard method.
