# Supplementary material for: NET-GE: a novel NETwork-based Gene Enrichment for detecting biological processes associated to Mendelian diseases
Source: BMC Genomics. 2015 Jun 18;16(Suppl 8):S6. doi: 10.1186/1471-2164-16-S8-S6 (PMC4480278; doi:10.1186/1471-2164-16-S8-S6)
Supplement: Additional file 3 — Detailed results for the OMIM-derived benchmark set. The archive contains pdf documents listing the enriched terms for each one of the 244 diseases in the OMIM-derived benchmark set. [file 1471-2164-16-S8-S6-S3.tgz › SUPPMAT/OMIM167000.pdf]

#167000 OVARIAN CANCER

OMIM Gene ID	HGNC	UniProtAC
116806	CTNNB1	P35222
164730	AKT1	P31749
171834	PIK3CA	P42336
192090	CDH1	P12830
600632	OPCML	Q14982
602544	PARK2	O60260

Table 1: OMIM - UniProtAC mapping

Legend

- N1: #input proteins associated to the significant GO term
- N2: #proteins associated to the significant GO term
- P-value: Bonferroni-corrected p-value of Fisher's exact test
- *red*: go terms not related to the input proteins
- *blue*: go terms related to the input proteins (enriched uniquely by network-based method)
- *green*: go terms ancestors of terms enriched with the standard method (enriched uniquely by network-based method)

1 Standard enrichment

GO Term	N1	N2	P-value	Description
GO:0071680	2	5	0.000241087	response to indole-3-methanol
GO:0071681	2	5	0.000241087	cellular response to indole-3-methanol
GO:0071417	4	588	0.000976948	cellular response to organonitrogen compound
GO:1901699	4	645	0.00141231	cellular response to nitrogen compound
GO:0042981	5	1970	0.00253367	regulation of apoptotic process
GO:0043067	5	1982	0.00261114	regulation of programmed cell death
GO:0010941	5	2079	0.00330912	regulation of cell death
GO:2000008	2	19	0.00411851	regulation of protein localization to cell surface
GO:0090201	2	20	0.0045758	negative regulation of release of cytochrome c from mitochondria
GO:1901215	3	244	0.00602447	negative regulation of neuron death
GO:1900408	2	24	0.00664506	negative regulation of cellular response to oxidative stress
GO:1902883	2	24	0.00664506	negative regulation of response to oxidative stress
GO:2001234	3	286	0.00969492	negative regulation of apoptotic signaling pathway
GO:1901701	4	1086	0.0111787	cellular response to oxygen-containing compound
GO:0010823	2	31	0.01119	negative regulation of mitochondrion organization
GO:0010243	4	1094	0.0115083	response to organonitrogen compound
GO:0008219	4	1106	0.012016	cell death
GO:0016265	4	1117	0.012496	death
GO:1903201	2	33	0.0127042	regulation of oxidative stress-induced cell death
GO:0060548	4	1147	0.0138773	negative regulation of cell death
GO:1901698	4	1186	0.0158391	response to nitrogen compound
GO:0070887	5	2904	0.017284	cellular response to chemical stimulus
GO:1901214	3	348	0.0174335	regulation of neuron death
GO:0051246	5	2954	0.018803	regulation of protein metabolic process
GO:0050678	3	361	0.0194521	regulation of epithelial cell proliferation
GO:0071495	4	1291	0.0221459	cellular response to endogenous stimulus
GO:0050863	3	385	0.0235737	regulation of T cell activation
GO:0010038	3	402	0.0268178	response to metal ion
GO:0003382	2	48	0.0271121	epithelial cell morphogenesis
GO:0043491	2	48	0.0271121	protein kinase B signaling
GO:0009968	4	1361	0.0272772	negative regulation of signal transduction
GO:0060341	4	1363	0.0274357	regulation of cellular localization
GO:0090199	2	50	0.0294393	regulation of release of cytochrome c from mitochondria
GO:1900407	2	50	0.0294393	regulation of cellular response to oxidative stress
GO:0010604	5	3285	0.0317388	positive regulation of macromolecule metabolic process
GO:0023057	4	1420	0.0322465	negative regulation of signaling
GO:0010648	4	1424	0.0326061	negative regulation of cell communication
GO:0006921	2	54	0.0343802	cellular component disassembly involved in execution phase of apoptosis
GO:1902882	2	54	0.0343802	regulation of response to oxidative stress
GO:0031325	5	3418	0.0385876	positive regulation of cellular metabolic process
GO:0051247	4	1526	0.0428216	positive regulation of protein metabolic process

Table 2: Overrepresented GO terms with the standard enrichment

2 Network-based enrichment

GO Term	N1	N2	P-value	Description
GO:0035412	4	50	2.0627e-07	regulation of catenin import into nucleus
GO:0071248	5	346	2.14494e-06	cellular response to metal ion
GO:0071241	5	388	3.81157e-06	cellular response to inorganic substance
GO:0034976	5	410	5.02606e-06	response to endoplasmic reticulum stress
GO:0017157	5	411	5.08782e-06	regulation of exocytosis
GO:0022409	4	114	5.95823e-06	positive regulation of cell-cell adhesion
GO:0009895	5	445	7.57815e-06	negative regulation of catabolic process
GO:0008016	5	497	1.31826e-05	regulation of heart contraction
GO:0035414	3	23	1.76284e-05	negative regulation of catenin import into nucleus
GO:0060828	5	550	2.18925e-05	regulation of canonical Wnt signaling pathway
GO:0070613	5	609	3.64491e-05	regulation of protein processing
GO:1903317	5	609	3.64491e-05	regulation of protein maturation
GO:0010560	3	30	4.03938e-05	positive regulation of glycoprotein biosynthetic process
GO:0006936	5	640	4.67204e-05	muscle contraction
GO:1903020	3	32	4.93414e-05	positive regulation of glycoprotein metabolic process
GO:0045662	3	34	5.952e-05	negative regulation of myoblast differentiation
GO:0051604	5	741	9.71649e-05	protein maturation
GO:0045862	4	233	0.000106239	positive regulation of proteolysis
GO:0051129	6	1913	0.000109274	negative regulation of cellular component organization
GO:0009896	5	786	0.000130428	positive regulation of catabolic process
GO:0010955	4	247	0.000134275	negative regulation of protein processing
GO:1903318	4	247	0.000134275	negative regulation of protein maturation
GO:0030111	5	814	0.000155331	regulation of Wnt signaling pathway
GO:0003012	5	815	0.000156286	muscle system process
GO:0042176	5	816	0.000157246	regulation of protein catabolic process
GO:0007626	5	829	0.000170153	locomotory behavior
GO:0010959	5	835	0.000176389	regulation of metal ion transport
GO:0010954	4	271	0.00019477	positive regulation of protein processing
GO:1903319	4	271	0.00019477	positive regulation of protein maturation
GO:0033135	4	294	0.000269967	regulation of peptidyl-serine phosphorylation
GO:0022407	4	297	0.000281174	regulation of cell-cell adhesion
GO:0008584	4	300	0.000292725	male gonad development
GO:0043405	5	930	0.000301947	regulation of MAP kinase activity
GO:0010719	3	58	0.000306412	negative regulation of epithelial to mesenchymal transition
GO:0016339	3	59	0.000322805	calcium-dependent cell-cell adhesion
GO:0034405	3	64	0.000413572	response to fluid shear stress
GO:0007399	5	1018	0.000473892	nervous system development
GO:0030335	5	1024	0.000487979	positive regulation of cell migration
GO:0045661	3	68	0.000497336	regulation of myoblast differentiation
GO:0014908	2	4	0.000497984	myotube differentiation involved in skeletal muscle regeneration
GO:2000147	5	1042	0.000532244	positive regulation of cell motility
GO:0010559	3	70	0.000543149	regulation of glycoprotein biosynthetic process
GO:0007186	6	2507	0.000554577	G-protein coupled receptor signaling pathway
GO:0051272	5	1060	0.000579657	positive regulation of cellular component movement
GO:0045444	4	372	0.000692351	fat cell differentiation
GO:0045732	4	374	0.000707353	positive regulation of protein catabolic process
GO:0035335	4	377	0.000730311	peptidyl-tyrosine dephosphorylation
GO:0019221	5	1123	0.000772802	cytokine-mediated signaling pathway
GO:0040017	5	1124	0.000776235	positive regulation of locomotion
GO:0034334	2	5	0.000829907	adherens junction maintenance
GO:0042326	5	1164	0.000923873	negative regulation of phosphorylation
GO:0060444	3	84	0.000944544	branching involved in mammary gland duct morphogenesis
GO:1903018	3	84	0.000944544	regulation of glycoprotein metabolic process
GO:0033043	6	2759	0.000985788	regulation of organelle organization
GO:0031334	4	411	0.00103126	positive regulation of protein complex assembly
GO:0060393	3	88	0.00108751	regulation of pathway-restricted SMAD protein phosphorylation
GO:0008406	4	424	0.00116785	gonad development
GO:0043409	4	424	0.00116785	negative regulation of MAPK cascade
GO:1901654	4	426	0.00119	response to ketone
GO:0060627	5	1229	0.00121081	regulation of vesicle-mediated transport

Table 3: Overrepresented terms with the network-based enrichment. Only terms not detected with the standard method.

GO Term	N1	N2	P-value	Description
GO:0000578	3	93	0.00128558	embryonic axis specification
GO:0008217	4	438	0.0013296	regulation of blood pressure
GO:1902532	5	1289	0.00153488	negative regulation of intracellular signal transduction
GO:0007265	4	457	0.00157521	Ras protein signal transduction
GO:0050768	4	457	0.00157521	negative regulation of neurogenesis
GO:1901991	4	457	0.00157521	negative regulation of mitotic cell cycle phase transition
GO:0048742	3	101	0.00165009	regulation of skeletal muscle fiber development
GO:2001233	5	1313	0.00168239	regulation of apoptotic signaling pathway
GO:0033628	3	102	0.00169998	regulation of cell adhesion mediated by integrin
GO:0044057	5	1316	0.00170159	regulation of system process
GO:0002090	3	104	0.00180274	regulation of receptor internalization
GO:0010771	3	105	0.00185564	negative regulation of cell morphogenesis involved in differentiation
GO:1901988	4	482	0.0019482	negative regulation of cell cycle phase transition
GO:0010563	5	1353	0.00195317	negative regulation of phosphorus metabolic process
GO:0045936	5	1353	0.00195317	negative regulation of phosphate metabolic process
GO:0051148	3	107	0.00196451	negative regulation of muscle cell differentiation
GO:0031400	5	1368	0.00206323	negative regulation of protein modification process
GO:0060326	4	489	0.00206352	cell chemotaxis
GO:0051051	5	1375	0.00211625	negative regulation of transport
GO:0080135	5	1388	0.00221761	regulation of cellular response to stress
GO:0002053	3	113	0.00231643	positive regulation of mesenchymal cell proliferation
GO:0033132	2	8	0.00232319	negative regulation of glucokinase activity
GO:1903300	2	8	0.00232319	negative regulation of hexokinase activity
GO:0046328	4	505	0.00234624	regulation of JNK cascade
GO:2000243	3	115	0.00244243	positive regulation of reproductive process
GO:0010035	5	1420	0.00248365	response to inorganic substance
GO:0009636	4	517	0.00257653	response to toxic substance
GO:1903320	4	520	0.00263665	regulation of protein modification by small protein conjugation or removal
GO:0042462	3	120	0.00277718	eye photoreceptor cell development
GO:0042306	4	527	0.002781	regulation of protein import into nucleus
GO:0043270	4	532	0.00288766	positive regulation of ion transport
GO:0010595	3	122	0.00291918	positive regulation of endothelial cell migration
GO:0042493	5	1473	0.00297971	response to drug
GO:0048666	4	539	0.00304208	neuron development
GO:0001755	3	124	0.00306593	neural crest cell migration
GO:0001667	4	542	0.00311012	ameboidal cell migration
GO:0042461	3	125	0.00314111	photoreceptor cell development
GO:0071900	5	1492	0.00317563	regulation of protein serine/threonine kinase activity
GO:0006470	4	550	0.00329709	protein dephosphorylation
GO:0051241	5	1513	0.00340396	negative regulation of multicellular organismal process
GO:0090287	4	557	0.00346747	regulation of cellular response to growth factor stimulus
GO:0010464	3	132	0.00370194	regulation of mesenchymal cell proliferation
GO:0032872	4	568	0.00374837	regulation of stress-activated MAPK cascade
GO:0070302	4	569	0.00377475	regulation of stress-activated protein kinase signaling cascade
GO:2000179	3	134	0.00387358	positive regulation of neural precursor cell proliferation
GO:0001764	4	578	0.00401819	neuron migration
GO:0001818	4	578	0.00401819	negative regulation of cytokine production
GO:0046427	3	136	0.00405043	positive regulation of JAK-STAT cascade
GO:0044703	4	580	0.00407384	multi-organism reproductive process
GO:0043269	5	1590	0.00435554	regulation of ion transport
GO:0002763	3	140	0.00442006	positive regulation of myeloid leukocyte differentiation
GO:0008104	5	1615	0.00470628	protein localization
GO:0035914	3	143	0.00471153	skeletal muscle cell differentiation
GO:1900180	4	602	0.00472473	regulation of protein localization to nucleus
GO:0050670	4	605	0.00481917	regulation of lymphocyte proliferation
GO:0050796	4	606	0.00485095	regulation of insulin secretion
GO:0034109	3	145	0.00491274	homotypic cell-cell adhesion
GO:0010721	4	609	0.00494723	negative regulation of cell development
GO:0032944	4	609	0.00494723	regulation of mononuclear cell proliferation
GO:0071345	5	1640	0.00507919	cellular response to cytokine stimulus

Table 4: Overrepresented terms with the network-based enrichment. Only terms not detected with the standard method.

GO Term	N1	N2	P-value	Description
GO:0033036	5	1642	0.00510999	macromolecule localization
GO:0001501	4	620	0.00531255	skeletal system development
GO:0042733	3	149	0.00533221	embryonic digit morphogenesis
GO:0070663	4	621	0.00534673	regulation of leukocyte proliferation
GO:0043122	4	630	0.00566182	regulation of I-kappaB kinase/NF-kappaB signaling
GO:0045785	4	632	0.00573365	positive regulation of cell adhesion
GO:0050900	4	635	0.0058427	leukocyte migration
GO:0042308	3	157	0.0062411	negative regulation of protein import into nucleus
GO:0007033	3	158	0.00636145	vacuole organization
GO:1903202	2	13	0.00646915	negative regulation of oxidative stress-induced cell death
GO:0023014	4	653	0.00652987	signal transduction by phosphorylation
GO:0009612	4	658	0.00673102	response to mechanical stimulus
GO:0016485	4	661	0.00685389	protein processing
GO:0043406	4	661	0.00685389	positive regulation of MAP kinase activity
GO:0010717	3	162	0.00685834	regulation of epithelial to mesenchymal transition
GO:0021700	4	666	0.00706238	developmental maturation
GO:0051149	3	165	0.00724744	positive regulation of muscle cell differentiation
GO:0030162	5	1768	0.00737436	regulation of proteolysis
GO:0046822	4	675	0.00744957	regulation of nucleocytoplasmic transport
GO:0090276	4	675	0.00744957	regulation of peptide hormone secretion
GO:0001817	5	1774	0.00749932	regulation of cytokine production
GO:0045861	3	167	0.00751483	negative regulation of proteolysis
GO:0007260	2	14	0.00754674	tyrosine phosphorylation of STAT protein
GO:0043547	5	1783	0.00768991	positive regulation of GTPase activity
GO:0048534	4	682	0.00776149	hematopoietic or lymphoid organ development
GO:0010830	3	169	0.0077887	regulation of myotube differentiation
GO:0002791	4	684	0.00785236	regulation of peptide secretion
GO:0090087	4	688	0.00803653	regulation of peptide transport
GO:0032269	5	1804	0.0081496	negative regulation of cellular protein metabolic process
GO:0021987	3	172	0.00821179	cerebral cortex development
GO:0048011	4	692	0.00822386	neurotrophin TRK receptor signaling pathway
GO:1901990	4	692	0.00822386	regulation of mitotic cell cycle phase transition
GO:0044087	5	1812	0.00833036	regulation of cellular component biogenesis
GO:0007569	3	175	0.00864987	cell aging
GO:0034332	3	176	0.00879924	adherens junction organization
GO:0043408	5	1837	0.00891594	regulation of MAPK cascade
GO:0010770	3	177	0.00895037	positive regulation of cell morphogenesis involved in differentiation
GO:0060688	3	177	0.00895037	regulation of morphogenesis of a branching structure
GO:0090183	3	177	0.00895037	regulation of kidney development
GO:0007270	3	178	0.00910318	neuron-neuron synaptic transmission
GO:0070201	5	1849	0.00920839	regulation of establishment of protein localization
GO:0044265	5	1850	0.00923312	cellular macromolecule catabolic process
GO:0006968	3	179	0.00925771	cellular defense response
GO:0010948	4	717	0.00946955	negative regulation of cell cycle process
GO:0002028	3	181	0.009572	regulation of sodium ion transport
GO:0038179	4	720	0.00962795	neurotrophin signaling pathway
GO:1901987	4	721	0.0096812	regulation of cell cycle phase transition
GO:0030010	3	182	0.00973178	establishment of cell polarity
GO:0090068	4	724	0.00984225	positive regulation of cell cycle process
GO:0046823	3	183	0.00989329	negative regulation of nucleocytoplasmic transport
GO:0071635	2	16	0.00995013	negative regulation of transforming growth factor beta production
GO:0045669	3	184	0.0100566	positive regulation of osteoblast differentiation
GO:0009880	3	185	0.0102217	embryonic pattern specification
GO:0048565	3	185	0.0102217	digestive tract development
GO:0046888	3	186	0.0103885	negative regulation of hormone secretion
GO:0071496	4	736	0.0105064	cellular response to external stimulus
GO:0046330	3	187	0.0105572	positive regulation of JNK cascade
GO:0048857	3	187	0.0105572	neural nucleus development
GO:0071260	3	187	0.0105572	cellular response to mechanical stimulus
GO:0030858	3	188	0.0107277	positive regulation of epithelial cell differentiation

Table 5: Overrepresented terms with the network-based enrichment. Only terms not detected with the standard method.

GO Term	N1	N2	P-value	Description
GO:0009203	5	1920	0.011099	ribonucleoside triphosphate catabolic process
GO:0009207	5	1920	0.011099	purine ribonucleoside triphosphate catabolic process
GO:0043087	5	1921	0.0111277	regulation of GTPase activity
GO:0009146	5	1925	0.0112429	purine nucleoside triphosphate catabolic process
GO:0030334	5	1926	0.0112719	regulation of cell migration
GO:0033124	5	1931	0.0114176	regulation of GTP catabolic process
GO:0051146	3	192	0.0114278	striated muscle cell differentiation
GO:1903321	3	192	0.0114278	negative regulation of protein modification by small protein conjugation or removal
GO:0009143	5	1934	0.0115058	nucleoside triphosphate catabolic process
GO:0045087	5	1935	0.0115353	innate immune response
GO:0090002	3	196	0.0121577	establishment of protein localization to plasma membrane
GO:0051056	4	769	0.0125055	regulation of small GTPase mediated signal transduction
GO:0006941	3	198	0.0125339	striated muscle contraction
GO:0033690	2	18	0.0126844	positive regulation of osteoblast proliferation
GO:0090031	2	18	0.0126844	positive regulation of steroid hormone biosynthetic process
GO:0006152	5	1974	0.0127338	purine nucleoside catabolic process
GO:0046130	5	1974	0.0127338	purine ribonucleoside catabolic process
GO:0045833	3	202	0.0133094	negative regulation of lipid metabolic process
GO:0007610	5	1993	0.0133525	behavior
GO:0010639	4	783	0.013434	negative regulation of organelle organization
GO:0034103	3	203	0.0135081	regulation of tissue remodeling
GO:0042454	5	2000	0.0135864	ribonucleoside catabolic process
GO:0010001	3	205	0.0139114	glial cell differentiation
GO:0009154	5	2010	0.0139261	purine ribonucleotide catabolic process
GO:0009261	5	2011	0.0139605	ribonucleotide catabolic process
GO:0034097	5	2017	0.014168	response to cytokine
GO:2000270	2	19	0.0141755	negative regulation of fibroblast apoptotic process
GO:0006184	4	794	0.0141989	GTP catabolic process
GO:0001938	3	207	0.0143227	positive regulation of endothelial cell proliferation
GO:0000902	4	800	0.0146295	cell morphogenesis
GO:0043407	3	209	0.0147419	negative regulation of MAP kinase activity
GO:2000145	5	2039	0.0149498	regulation of cell motility
GO:0046883	4	805	0.0149957	regulation of hormone secretion
GO:0006195	5	2041	0.0150225	purine nucleotide catabolic process
GO:1901069	4	807	0.0151441	guanosine-containing compound catabolic process
GO:0009164	5	2045	0.0151689	nucleoside catabolic process
GO:0046425	3	211	0.0151692	regulation of JAK-STAT cascade
GO:0006887	4	808	0.0152187	exocytosis
GO:0010634	3	212	0.0153859	positive regulation of epithelial cell migration
GO:0048641	3	212	0.0153859	regulation of skeletal muscle tissue development
GO:1901658	5	2055	0.0155397	glycosyl compound catabolic process
GO:0072523	5	2067	0.0159941	purine-containing compound catabolic process
GO:0045786	4	824	0.0164499	negative regulation of cell cycle
GO:0097306	3	219	0.0169605	cellular response to alcohol
GO:0051046	5	2094	0.0170553	regulation of secretion
GO:0032147	4	832	0.0170926	activation of protein kinase activity
GO:0033138	3	220	0.0171939	positive regulation of peptidyl-serine phosphorylation
GO:0033131	2	21	0.0174058	regulation of glucokinase activity
GO:1903299	2	21	0.0174058	regulation of hexokinase activity
GO:0033121	5	2107	0.0175858	regulation of purine nucleotide catabolic process
GO:0071214	4	838	0.0175868	cellular response to abiotic stimulus
GO:0048609	5	2109	0.0176686	multicellular organismal reproductive process
GO:0030811	5	2111	0.0177516	regulation of nucleotide catabolic process
GO:0006935	4	841	0.0178378	chemotaxis
GO:0042330	4	841	0.0178378	taxis
GO:0009166	5	2116	0.0179607	nucleotide catabolic process
GO:0019216	4	845	0.0181766	regulation of lipid metabolic process
GO:0009118	5	2125	0.0183419	regulation of nucleoside metabolic process
GO:0032496	4	847	0.0183478	response to lipopolysaccharide
GO:0030900	3	225	0.0183924	forebrain development

Table 6: Overrepresented terms with the network-based enrichment. Only terms not detected with the standard method.

GO Term	N1	N2	P-value	Description
GO:1901292	5	2130	0.0185565	nucleoside phosphate catabolic process
GO:0045639	3	226	0.0186386	positive regulation of myeloid cell differentiation
GO:0009205	5	2149	0.0193901	purine ribonucleoside triphosphate metabolic process
GO:0032874	3	230	0.0196451	positive regulation of stress-activated MAPK cascade
GO:0046039	4	862	0.0196705	GTP metabolic process
GO:0009144	5	2159	0.0198406	purine nucleoside triphosphate metabolic process
GO:0032231	3	232	0.0201616	regulation of actin filament bundle assembly
GO:0070304	3	232	0.0201616	positive regulation of stress-activated protein kinase signaling cascade
GO:0009199	5	2170	0.0203458	ribonucleoside triphosphate metabolic process
GO:0010517	3	233	0.0204232	regulation of phospholipase activity
GO:0090317	3	234	0.020687	negative regulation of intracellular protein transport
GO:0043254	4	874	0.0207787	regulation of protein complex assembly
GO:0051270	5	2182	0.0209085	regulation of cellular component movement
GO:0007267	5	2185	0.0210511	cell-cell signaling
GO:0051240	5	2185	0.0210511	positive regulation of multicellular organismal process
GO:0033157	4	878	0.0211582	regulation of intracellular protein transport
GO:0051248	5	2189	0.0212424	negative regulation of protein metabolic process
GO:0061136	3	237	0.0214921	regulation of proteasomal protein catabolic process
GO:2000648	3	237	0.0214921	positive regulation of stem cell proliferation
GO:0032880	5	2200	0.0217757	regulation of protein localization
GO:0051348	4	887	0.022031	negative regulation of transferase activity
GO:0010447	2	24	0.0228707	response to acidic pH
GO:0045663	2	24	0.0228707	positive regulation of myoblast differentiation
GO:0046886	2	24	0.0228707	positive regulation of hormone biosynthetic process
GO:0018193	5	2224	0.0229762	peptidyl-amino acid modification
GO:0040012	5	2224	0.0229762	regulation of locomotion
GO:0009141	5	2225	0.0230273	nucleoside triphosphate metabolic process
GO:0071902	4	897	0.0230319	positive regulation of protein serine/threonine kinase activity
GO:0016337	4	900	0.0233386	single organismal cell-cell adhesion
GO:1901068	4	901	0.0234415	guanosine-containing compound metabolic process
GO:0051052	4	905	0.0238566	regulation of DNA metabolic process
GO:0043506	3	247	0.024325	regulation of JUN kinase activity
GO:0010463	2	25	0.0248574	mesenchymal cell proliferation
GO:0014009	2	25	0.0248574	glial cell proliferation
GO:0097284	2	25	0.0248574	hepatocyte apoptotic process
GO:0046434	5	2260	0.024875	organophosphate catabolic process
GO:0003018	3	250	0.0252207	vascular process in circulatory system
GO:0002237	4	918	0.0252435	response to molecule of bacterial origin
GO:0010631	3	251	0.0255241	epithelial cell migration
GO:0072593	3	252	0.0258298	reactive oxygen species metabolic process
GO:0048468	5	2280	0.0259825	cell development
GO:0040014	3	253	0.026138	regulation of multicellular organism growth
GO:0046879	3	253	0.026138	hormone secretion
GO:0032956	4	928	0.0263504	regulation of actin cytoskeleton organization
GO:0023052	5	2287	0.0263793	signaling
GO:0044700	5	2287	0.0263793	single organism signaling
GO:0002521	4	929	0.0264631	leukocyte differentiation
GO:0048511	4	930	0.0265761	rhythmic process
GO:0050804	4	931	0.0266894	regulation of synaptic transmission
GO:0001504	2	26	0.0269267	neurotransmitter uptake
GO:0060046	2	26	0.0269267	regulation of acrosome reaction
GO:1900006	2	26	0.0269267	positive regulation of dendrite development
GO:0034765	4	934	0.0270317	regulation of ion transmembrane transport
GO:0072331	3	256	0.0270772	signal transduction by p53 class mediator
GO:0031214	3	257	0.0273951	biomineral tissue development
GO:0000122	5	2314	0.027955	negative regulation of transcription from RNA polymerase II promoter
GO:0009057	5	2318	0.0281947	macromolecule catabolic process
GO:0002683	4	945	0.0283147	negative regulation of immune system process
GO:0001525	4	949	0.0287922	angiogenesis
GO:0016525	3	262	0.029022	negative regulation of angiogenesis

Table 7: Overrepresented terms with the network-based enrichment. Only terms not detected with the standard method.

GO Term	N1	N2	P-value	Description
GO:0002433	3	264	0.0296903	immune response-regulating cell surface receptor signaling pathway involved in phagocytosis
GO:0009953	3	264	0.0296903	dorsal/ventral pattern formation
GO:0038094	3	264	0.0296903	Fc-gamma receptor signaling pathway
GO:0038096	3	264	0.0296903	Fc-gamma receptor signaling pathway involved in phagocytosis
GO:0002431	3	266	0.0303687	Fc receptor mediated stimulatory signaling pathway
GO:0051781	3	266	0.0303687	positive regulation of cell division
GO:0008285	5	2354	0.0304261	negative regulation of cell proliferation
GO:0001819	4	963	0.030511	positive regulation of cytokine production
GO:0001933	4	963	0.030511	negative regulation of protein phosphorylation
GO:0001505	3	267	0.0307118	regulation of neurotransmitter levels
GO:0048145	3	268	0.0310573	regulation of fibroblast proliferation
GO:2000269	2	28	0.0313128	regulation of fibroblast apoptotic process
GO:0060021	3	269	0.0314053	palate development
GO:0048660	3	272	0.0324654	regulation of smooth muscle cell proliferation
GO:0042475	3	273	0.0328238	odontogenesis of dentin-containing tooth
GO:0098602	4	981	0.0328318	single organism cell adhesion
GO:0007229	3	274	0.0331849	integrin-mediated signaling pathway
GO:0009798	3	275	0.0335485	axis specification
GO:0014067	2	29	0.0336296	negative regulation of phosphatidylinositol 3-kinase signaling
GO:0034616	2	29	0.0336296	response to laminar fluid shear stress
GO:0046128	5	2404	0.0337558	purine ribonucleoside metabolic process
GO:0003002	4	988	0.0337687	regionalization
GO:0048878	5	2410	0.0341741	chemical homeostasis
GO:0042278	5	2412	0.0343141	purine nucleoside metabolic process
GO:1902533	5	2418	0.0347379	positive regulation of intracellular signal transduction
GO:0032970	4	996	0.0348638	regulation of actin filament-based process
GO:0034762	4	998	0.0351418	regulation of transmembrane transport
GO:0008203	3	282	0.036169	cholesterol metabolic process
GO:0051216	3	282	0.036169	cartilage development
GO:0045859	5	2438	0.0361807	regulation of protein kinase activity
GO:0071456	3	283	0.0365541	cellular response to hypoxia
GO:0006836	3	284	0.0369417	neurotransmitter transport
GO:0002698	3	285	0.0373323	negative regulation of immune effector process
GO:0010594	3	287	0.0381212	regulation of endothelial cell migration
GO:0009914	3	288	0.0385199	hormone transport
GO:0036294	3	288	0.0385199	cellular response to decreased oxygen levels
GO:2000177	3	288	0.0385199	regulation of neural precursor cell proliferation
GO:1901136	5	2479	0.0392867	carbohydrate derivative catabolic process
GO:0002274	3	290	0.0393257	myeloid leukocyte activation
GO:1900542	5	2485	0.0397584	regulation of purine nucleotide metabolic process
GO:0043066	5	2487	0.0399166	negative regulation of apoptotic process
GO:0016311	4	1031	0.0399681	dephosphorylation
GO:0030029	4	1031	0.0399681	actin filament-based process
GO:0009119	5	2491	0.0402347	ribonucleoside metabolic process
GO:0006140	5	2500	0.0409576	regulation of nucleotide metabolic process
GO:0048514	3	294	0.0409702	blood vessel morphogenesis
GO:0010803	2	32	0.0410746	regulation of tumor necrosis factor-mediated signaling pathway
GO:0014902	2	32	0.0410746	myotube differentiation
GO:0051153	3	295	0.0413882	regulation of striated muscle cell differentiation
GO:0043069	5	2511	0.041855	negative regulation of programmed cell death
GO:0032387	3	299	0.0430889	negative regulation of intracellular transport
GO:0097190	4	1052	0.0432857	apoptotic signaling pathway
GO:0007218	3	300	0.0435213	neuropeptide signaling pathway
GO:0070507	3	300	0.0435213	regulation of microtubule cytoskeleton organization
GO:0009150	5	2531	0.0435268	purine ribonucleotide metabolic process
GO:0032232	2	33	0.0437209	negative regulation of actin filament bundle assembly
GO:0071356	3	301	0.0439565	cellular response to tumor necrosis factor
GO:0090288	3	301	0.0439565	negative regulation of cellular response to growth factor stimulus
GO:2000736	3	302	0.0443944	regulation of stem cell differentiation
GO:0030326	3	303	0.0448354	embryonic limb morphogenesis

Table 8: Overrepresented terms with the network-based enrichment. Only terms not detected with the standard method.

GO Term	N1	N2	P-value	Description
GO:0035113	3	303	0.0448354	embryonic appendage morphogenesis
GO:0048608	4	1062	0.0449355	reproductive structure development
GO:0009116	5	2561	0.0461335	nucleoside metabolic process
GO:0001936	3	306	0.0461752	regulation of endothelial cell proliferation
GO:0045598	3	307	0.0466279	regulation of fat cell differentiation
GO:0090090	3	307	0.0466279	negative regulation of canonical Wnt signaling pathway
GO:2001020	3	308	0.0470833	regulation of response to DNA damage stimulus
GO:0043549	5	2576	0.0474823	regulation of kinase activity
GO:0048704	3	310	0.0480028	embryonic skeletal system morphogenesis
GO:1903322	3	310	0.0480028	positive regulation of protein modification by small protein conjugation or removal
GO:0009259	5	2583	0.0481226	ribonucleotide metabolic process
GO:0006163	5	2586	0.048399	purine nucleotide metabolic process
GO:0097305	4	1084	0.0487291	response to alcohol
GO:0032352	2	35	0.049261	positive regulation of hormone metabolic process
GO:0019693	5	2596	0.0493295	ribose phosphate metabolic process
GO:0051090	4	1091	0.0499845	regulation of sequence-specific DNA binding transcription factor activity

Table 9: Overrepresented terms with the network-based enrichment. Only terms not detected with the standard method.
