# Supplementary material for: NET-GE: a novel NETwork-based Gene Enrichment for detecting biological processes associated to Mendelian diseases
Source: BMC Genomics. 2015 Jun 18;16(Suppl 8):S6. doi: 10.1186/1471-2164-16-S8-S6 (PMC4480278; doi:10.1186/1471-2164-16-S8-S6)
Supplement: Additional file 3 — Detailed results for the OMIM-derived benchmark set. The archive contains pdf documents listing the enriched terms for each one of the 244 diseases in the OMIM-derived benchmark set. [file 1471-2164-16-S8-S6-S3.tgz › SUPPMAT/OMIM167800.pdf]

#167800 PANCREATITIS, HEREDITARY; PCTT

**OMIM Gene ID**	**HGNC**	**UniProtAC**
167790	SPINK1	P00995
276000	PRSS1	P07477
601405	CTRC	Q99895
601564	PRSS2	P07478
602421	CFTR	P13569

Table 1: OMIM - UniProtAC mapping

Legend

- N1: #input proteins associated to the significant GO term
- N2: #proteins associated to the significant GO term
- P-value: Bonferroni-corrected p-value of Fisher's exact test
- *red*: go terms not related to the input proteins
- *blue*: go terms related to the input proteins (enriched uniquely by network-based method)
- *green*: go terms ancestors of terms enriched with the standard method (enriched uniquely by network-based method)

1 Standard enrichment

GO Term	N1	N2	P-value	Description
GO:0007586	2	74	0.0119755	digestion
GO:0022617	2	117	0.0300188	extracellular matrix disassembly
GO:1901529	1	1	0.0419946	positive regulation of anion channel activity
GO:1902941	1	1	0.0419946	regulation of voltage-gated chloride channel activity
GO:1902943	1	1	0.0419946	positive regulation of voltage-gated chloride channel activity

Table 2: Overrepresented GO terms with the standard enrichment

2 Network-based enrichment

No novel enriched terms
