# Supplementary material for: NET-GE: a novel NETwork-based Gene Enrichment for detecting biological processes associated to Mendelian diseases
Source: BMC Genomics. 2015 Jun 18;16(Suppl 8):S6. doi: 10.1186/1471-2164-16-S8-S6 (PMC4480278; doi:10.1186/1471-2164-16-S8-S6)
Supplement: Additional file 3 — Detailed results for the OMIM-derived benchmark set. The archive contains pdf documents listing the enriched terms for each one of the 244 diseases in the OMIM-derived benchmark set. [file 1471-2164-16-S8-S6-S3.tgz › SUPPMAT/OMIM168600.pdf]

#168600 PARKINSON DISEASE, LATE-ONSET; PD

**OMIM Gene ID**	**HGNC**	**UniProtAC**
103730	ADH1C	P00326
157140	MAPT	P10636
600075	TBP	P20226
606463	GBA	P04062

Table 1: OMIM - UniProtAC mapping

Legend

- N1: #input proteins associated to the significant GO term
- N2: #proteins associated to the significant GO term
- P-value: Bonferroni-corrected p-value of Fisher's exact test
- *red*: go terms not related to the input proteins
- *blue*: go terms related to the input proteins (enriched uniquely by network-based method)
- *green*: go terms ancestors of terms enriched with the standard method (enriched uniquely by network-based method)

1 Standard enrichment

GO Term	N1	N2	P-value	Description
GO:0008219	3	1106	0.0346594	cell death
GO:0016265	3	1117	0.0356968	death

Table 2: Overrepresented GO terms with the standard enrichment

2 Network-based enrichment

GO Term	N1	N2	P-value	Description
GO:0051384	3	564	0.016146	response to glucocorticoid
GO:0047496	2	65	0.0190005	vesicle transport along microtubule
GO:0031960	3	610	0.0204145	response to corticosteroid
GO:0072384	2	103	0.0479124	organelle transport along microtubule

Table 3: Overrepresented terms with the network-based enrichment. Only terms not detected with the standard method.
