# Supplementary material for: NET-GE: a novel NETwork-based Gene Enrichment for detecting biological processes associated to Mendelian diseases
Source: BMC Genomics. 2015 Jun 18;16(Suppl 8):S6. doi: 10.1186/1471-2164-16-S8-S6 (PMC4480278; doi:10.1186/1471-2164-16-S8-S6)
Supplement: Additional file 3 — Detailed results for the OMIM-derived benchmark set. The archive contains pdf documents listing the enriched terms for each one of the 244 diseases in the OMIM-derived benchmark set. [file 1471-2164-16-S8-S6-S3.tgz › SUPPMAT/OMIM171300.pdf]

#171300 PHEOCHROMOCYTOMA

OMIM Gene ID	HGNC	UniProtAC
154950	MAX	P61244
164761	RET	P07949
185470	SDHB	P21912
600837	GDNF	P39905
602690	SDHD	O14521
605995	KIF1B	O60333
608537	VHL	P40337
613403	TMEM127	O75204

Table 1: OMIM - UniProtAC mapping

Legend

- N1: #input proteins associated to the significant GO term
- N2: #proteins associated to the significant GO term
- P-value: Bonferroni-corrected p-value of Fisher's exact test
- *red*: go terms not related to the input proteins
- *blue*: go terms related to the input proteins (enriched uniquely by network-based method)
- *green*: go terms ancestors of terms enriched with the standard method (enriched uniquely by network-based method)

1 Standard enrichment

GO Term	N1	N2	P-value	Description
GO:0048484	2	15	0.00210227	enteric nervous system development
GO:0001838	2	36	0.0125855	embryonic epithelial tube formation
GO:0072175	2	37	0.0133033	epithelial tube formation
GO:0072215	2	37	0.0133033	regulation of metanephros development
GO:0001656	2	47	0.02157	metanephros development
GO:0006099	2	49	0.0234607	tricarboxylic acid cycle
GO:0001755	2	59	0.0340975	neural crest cell migration

Table 2: Overrepresented GO terms with the standard enrichment

2 Network-based enrichment

GO Term	N1	N2	P-value	Description
GO:0061146	2	10	0.00197349	Peyer's patch morphogenesis
GO:0048483	2	15	0.00460206	autonomic nervous system development
GO:0090183	3	177	0.0070442	regulation of kidney development
GO:0072073	3	223	0.0140637	kidney epithelium development
GO:0048485	2	36	0.0275428	sympathetic nervous system development
GO:0050930	2	41	0.0358278	induction of positive chemotaxis

Table 3: Overrepresented terms with the network-based enrichment. Only terms not detected with the standard method.
