# Supplementary material for: NET-GE: a novel NETwork-based Gene Enrichment for detecting biological processes associated to Mendelian diseases
Source: BMC Genomics. 2015 Jun 18;16(Suppl 8):S6. doi: 10.1186/1471-2164-16-S8-S6 (PMC4480278; doi:10.1186/1471-2164-16-S8-S6)
Supplement: Additional file 3 — Detailed results for the OMIM-derived benchmark set. The archive contains pdf documents listing the enriched terms for each one of the 244 diseases in the OMIM-derived benchmark set. [file 1471-2164-16-S8-S6-S3.tgz › SUPPMAT/OMIM172800.pdf]

#172800 PIEBALD TRAIT; PBT

OMIM Gene ID	HGNC	UniProtAC
164920	KIT	P10721
602150	SNAI2	O43623

Table 1: OMIM - UniProtAC mapping

Legend

- N1: #input proteins associated to the significant GO term
- N2: #proteins associated to the significant GO term
- P-value: Bonferroni-corrected p-value of Fisher's exact test
- *red*: go terms not related to the input proteins
- *blue*: go terms related to the input proteins (enriched uniquely by network-based method)
- *green*: go terms ancestors of terms enriched with the standard method (enriched uniquely by network-based method)

1 Standard enrichment

GO Term	N1	N2	P-value	Description
GO:0043473	2	57	0.00128621	pigmentation
GO:0044344	2	237	0.0225378	cellular response to fibroblast growth factor stimulus
GO:0071774	2	243	0.0236959	response to fibroblast growth factor
GO:0002371	1	1	0.0304163	dendritic cell cytokine production
GO:0032762	1	1	0.0304163	mast cell cytokine production
GO:0035921	1	1	0.0304163	desmosome disassembly
GO:0070563	1	1	0.0304163	negative regulation of vitamin D receptor signaling pathway
GO:0070662	1	1	0.0304163	mast cell proliferation
GO:0097326	1	1	0.0304163	melanocyte adhesion
GO:1900387	1	1	0.0304163	negative regulation of cell-cell adhesion by negative regulation of transcription from RNA polymerase II promo

Table 2: Overrepresented GO terms with the standard enrichment

2 Network-based enrichment

GO Term	N1	N2	P-value	Description
GO:0014068	2	150	0.011559	positive regulation of phosphatidylinositol 3-kinase signaling
GO:0014066	2	210	0.0226992	regulation of phosphatidylinositol 3-kinase signaling
GO:0050673	2	275	0.0389698	epithelial cell proliferation
GO:0008584	2	300	0.0463914	male gonad development

Table 3: Overrepresented terms with the network-based enrichment. Only terms not detected with the standard method.
