# Supplementary material for: NET-GE: a novel NETwork-based Gene Enrichment for detecting biological processes associated to Mendelian diseases
Source: BMC Genomics. 2015 Jun 18;16(Suppl 8):S6. doi: 10.1186/1471-2164-16-S8-S6 (PMC4480278; doi:10.1186/1471-2164-16-S8-S6)
Supplement: Additional file 3 — Detailed results for the OMIM-derived benchmark set. The archive contains pdf documents listing the enriched terms for each one of the 244 diseases in the OMIM-derived benchmark set. [file 1471-2164-16-S8-S6-S3.tgz › SUPPMAT/OMIM174050.pdf]

#174050 POLYCYSTIC LIVER DISEASE; PCLD

OMIM Gene ID	HGNC	UniProtAC
177060	PRKCSH	P14314
608648	SEC63	Q9UGP8

Table 1: OMIM - UniProtAC mapping

Legend

- N1: #input proteins associated to the significant GO term
- N2: #proteins associated to the significant GO term
- P-value: Bonferroni-corrected p-value of Fisher's exact test
- *red*: go terms not related to the input proteins
- *blue*: go terms related to the input proteins (enriched uniquely by network-based method)
- *green*: go terms ancestors of terms enriched with the standard method (enriched uniquely by network-based method)

1 Standard enrichment

GO Term	N1	N2	P-value	Description
GO:0072001	2	18	2.27702e-05	renal system development
GO:0001889	2	173	0.00221421	liver development
GO:0048732	2	391	0.0113471	gland development
GO:0031204	1	3	0.0168504	posttranslational protein targeting to membrane, translocation
GO:0006620	1	5	0.0280832	posttranslational protein targeting to membrane

Table 2: Overrepresented GO terms with the standard enrichment

2 Network-based enrichment

GO Term	N1	N2	P-value	Description
GO:0006112	2	412	0.0474496	energy reserve metabolic process

Table 3: Overrepresented terms with the network-based enrichment. Only terms not detected with the standard method.
