# Supplementary material for: NET-GE: a novel NETwork-based Gene Enrichment for detecting biological processes associated to Mendelian diseases
Source: BMC Genomics. 2015 Jun 18;16(Suppl 8):S6. doi: 10.1186/1471-2164-16-S8-S6 (PMC4480278; doi:10.1186/1471-2164-16-S8-S6)
Supplement: Additional file 3 — Detailed results for the OMIM-derived benchmark set. The archive contains pdf documents listing the enriched terms for each one of the 244 diseases in the OMIM-derived benchmark set. [file 1471-2164-16-S8-S6-S3.tgz › SUPPMAT/OMIM174900.pdf]

# #174900 JUVENILE POLYPOSIS SYNDROME; JPS

| **OMIM Gene ID** | **HGNC** | **UniProtAC** |
|---|---|---|
| 600993 | SMAD4 | Q13485 |
| 601299 | BMPR1A | P36894 |

Table 1: OMIM - UniProtAC mapping

Legend

- N1: #input proteins associated to the significant GO term
- N2: #proteins associated to the significant GO term
- P-value: Bonferroni-corrected p-value of Fisher's exact test
- *red*: go terms not related to the input proteins
- *blue*: go terms related to the input proteins (enriched uniquely by network-based method)
- *green*: go terms ancestors of terms enriched with the standard method (enriched uniquely by network-based method)

# 1 Standard enrichment

| GO Term | N1 | N2 | P-value | Description |
|---|---|---|---|---|
| GO:0060391 | 2 | 17 | 6.9504e-05 | positive regulation of SMAD protein import into nucleus |
| GO:0060390 | 2 | 20 | 9.7101e-05 | regulation of SMAD protein import into nucleus |
| GO:0010862 | 2 | 33 | 0.000269839 | positive regulation of pathway-restricted SMAD protein phosphorylation |
| GO:0060393 | 2 | 45 | 0.000505945 | regulation of pathway-restricted SMAD protein phosphorylation |
| GO:0007498 | 2 | 67 | 0.00112995 | mesoderm development |
| GO:0030509 | 2 | 88 | 0.00195633 | BMP signaling pathway |
| GO:0042307 | 2 | 102 | 0.00263246 | positive regulation of protein import into nucleus |
| GO:0090100 | 2 | 103 | 0.00268459 | positive regulation of transmembrane receptor protein serine/threonine kinase signaling pathway |
| GO:0046824 | 2 | 119 | 0.00358814 | positive regulation of nucleocytoplasmic transport |
| GO:0060021 | 2 | 120 | 0.00364895 | palate development |
| GO:0009798 | 2 | 132 | 0.0044186 | axis specification |
| GO:0007179 | 2 | 189 | 0.00907947 | transforming growth factor beta receptor signaling pathway |
| GO:0090316 | 2 | 193 | 0.00946888 | positive regulation of intracellular protein transport |
| GO:0042306 | 2 | 208 | 0.011002 | regulation of protein import into nucleus |
| GO:2000027 | 2 | 225 | 0.0128786 | regulation of organ morphogenesis |
| GO:0071560 | 2 | 238 | 0.0144134 | cellular response to transforming growth factor beta stimulus |
| GO:0090092 | 2 | 239 | 0.014535 | regulation of transmembrane receptor protein serine/threonine kinase signaling pathway |
| GO:1900180 | 2 | 242 | 0.014903 | regulation of protein localization to nucleus |
| GO:0071559 | 2 | 248 | 0.0156527 | response to transforming growth factor beta |
| GO:0032388 | 2 | 259 | 0.0170749 | positive regulation of intracellular transport |
| GO:0046822 | 2 | 262 | 0.0174736 | regulation of nucleocytoplasmic transport |
| GO:0021998 | 1 | 1 | 0.0192884 | neural plate mediolateral regionalization |
| GO:0048338 | 1 | 1 | 0.0192884 | mesoderm structural organization |
| GO:0048352 | 1 | 1 | 0.0192884 | paraxial mesoderm structural organization |
| GO:0007178 | 2 | 293 | 0.021862 | transmembrane receptor protein serine/threonine kinase signaling pathway |
| GO:0048589 | 2 | 296 | 0.0223128 | developmental growth |
| GO:0033157 | 2 | 322 | 0.026412 | regulation of intracellular protein transport |
| GO:0051222 | 2 | 349 | 0.0310345 | positive regulation of protein transport |
| GO:0003272 | 1 | 2 | 0.0385764 | endocardial cushion formation |
| GO:0048382 | 1 | 2 | 0.0385764 | mesendoderm development |
| GO:0060896 | 1 | 2 | 0.0385764 | neural plate pattern specification |
| GO:0060897 | 1 | 2 | 0.0385764 | neural plate regionalization |
| GO:0048732 | 2 | 391 | 0.0389655 | gland development |
| GO:0001701 | 2 | 432 | 0.0475773 | in utero embryonic development |
| GO:0043009 | 2 | 442 | 0.0498083 | chordate embryonic development |

Table 2: Overrepresented GO terms with the standard enrichment

## 2 Network-based enrichment

| GO Term | N1 | N2 | P-value | Description |
|---|---|---|---|---|
| GO:0042659 | 2 | 37 | 0.0015134 | regulation of cell fate specification |
| GO:0060033 | 2 | 39 | 0.00168383 | anatomical structure regression |
| GO:0032924 | 2 | 43 | 0.00205195 | activin receptor signaling pathway |
| GO:0048645 | 2 | 49 | 0.0026723 | organ formation |
| GO:2000136 | 2 | 49 | 0.0026723 | regulation of cell proliferation involved in heart morphogenesis |
| GO:2000826 | 2 | 68 | 0.00517646 | regulation of heart morphogenesis |
| GO:0010453 | 2 | 89 | 0.00889859 | regulation of cell fate commitment |
| GO:0030501 | 2 | 89 | 0.00889859 | positive regulation of bone mineralization |
| GO:0070169 | 2 | 97 | 0.0105801 | positive regulation of biomineral tissue development |
| GO:0010470 | 2 | 108 | 0.0131298 | regulation of gastrulation |
| GO:0002053 | 2 | 113 | 0.0143796 | positive regulation of mesenchymal cell proliferation |
| GO:0045778 | 2 | 124 | 0.017329 | positive regulation of ossification |
| GO:0010464 | 2 | 132 | 0.0196469 | regulation of mesenchymal cell proliferation |
| GO:0035137 | 2 | 137 | 0.0211694 | hindlimb morphogenesis |
| GO:0071333 | 2 | 148 | 0.0247188 | cellular response to glucose stimulus |
| GO:0071331 | 2 | 158 | 0.0281841 | cellular response to hexose stimulus |
| GO:0071326 | 2 | 160 | 0.0289045 | cellular response to monosaccharide stimulus |
| GO:0060485 | 2 | 161 | 0.0292681 | mesenchyme development |
| GO:0007368 | 2 | 164 | 0.0303724 | determination of left/right symmetry |
| GO:0009855 | 2 | 167 | 0.0314973 | determination of bilateral symmetry |
| GO:0009799 | 2 | 168 | 0.0318768 | specification of symmetry |
| GO:0001678 | 2 | 169 | 0.0322585 | cellular glucose homeostasis |
| GO:0045669 | 2 | 184 | 0.0382576 | positive regulation of osteoblast differentiation |
| GO:0030072 | 2 | 189 | 0.0403709 | peptide hormone secretion |
| GO:0071322 | 2 | 192 | 0.0416661 | cellular response to carbohydrate stimulus |
| GO:0002790 | 2 | 199 | 0.0447679 | peptide secretion |
| GO:0030500 | 2 | 210 | 0.0498671 | regulation of bone mineralization |

Table 3: Overrepresented terms with the network-based enrichment. Only terms not detected with the standard method.
