# Supplementary material for: NET-GE: a novel NETwork-based Gene Enrichment for detecting biological processes associated to Mendelian diseases
Source: BMC Genomics. 2015 Jun 18;16(Suppl 8):S6. doi: 10.1186/1471-2164-16-S8-S6 (PMC4480278; doi:10.1186/1471-2164-16-S8-S6)
Supplement: Additional file 3 — Detailed results for the OMIM-derived benchmark set. The archive contains pdf documents listing the enriched terms for each one of the 244 diseases in the OMIM-derived benchmark set. [file 1471-2164-16-S8-S6-S3.tgz › SUPPMAT/OMIM176807.pdf]

#176807 PROSTATE CANCER

OMIM Gene ID	HGNC	UniProtAC
104155	ZFHX3	Q15911
153622	MSR1	P21757
192090	CDH1	P12830
313700	AR	P10275
600020	MXI1	P50539
600185	BRCA2	P51587
600623	CD82	P27701
601728	PTEN	P60484
601767	HIP1	O00291
602053	KLF6	Q99612
602686	MAD1L1	Q9Y6D9
604373	CHEK2	O96017

Table 1: OMIM - UniProtAC mapping

Legend

- N1: #input proteins associated to the significant GO term
- N2: #proteins associated to the significant GO term
- P-value: Bonferroni-corrected p-value of Fisher's exact test
- *red*: go terms not related to the input proteins
- *blue*: go terms related to the input proteins (enriched uniquely by network-based method)
- *green*: go terms ancestors of terms enriched with the standard method (enriched uniquely by network-based method)

1 Standard enrichment

GO Term	N1	N2	P-value	Description
GO:0006355	9	6146	0.00284533	regulation of transcription, DNA-templated
GO:0032879	7	2827	0.00293164	regulation of localization
GO:2001141	9	6249	0.00328578	regulation of RNA biosynthetic process
GO:0051252	9	6360	0.00382649	regulation of RNA metabolic process
GO:2000112	9	6579	0.00512691	regulation of cellular macromolecule biosynthetic process
GO:0048518	9	6624	0.00543767	positive regulation of biological process
GO:0010556	9	6784	0.0066807	regulation of macromolecule biosynthetic process
GO:0031326	9	6987	0.0086122	regulation of cellular biosynthetic process
GO:0009889	9	7051	0.00931505	regulation of biosynthetic process
GO:0050680	3	159	0.0104621	negative regulation of epithelial cell proliferation
GO:0010468	9	7158	0.0106029	regulation of gene expression
GO:0051726	5	1232	0.0127673	regulation of cell cycle
GO:0048523	8	5279	0.0143302	negative regulation of cellular process
GO:0060341	5	1363	0.0208035	regulation of cellular localization
GO:0019219	9	7797	0.022068	regulation of nucleobase-containing compound metabolic process
GO:0010605	6	2452	0.0230082	negative regulation of macromolecule metabolic process
GO:0022402	5	1409	0.0244118	cell cycle process
GO:0010564	4	662	0.0248617	regulation of cell cycle process
GO:0051171	9	7927	0.0254169	regulation of nitrogen compound metabolic process
GO:0048519	8	5756	0.0275578	negative regulation of biological process
GO:0048522	8	5768	0.0279937	positive regulation of cellular process
GO:0009892	6	2679	0.0381065	negative regulation of metabolic process
GO:1901988	3	259	0.0448327	negative regulation of cell cycle phase transition

Table 2: Overrepresented GO terms with the standard enrichment

2 Network-based enrichment

GO Term	N1	N2	P-value	Description
GO:0007569	5	175	5.89329e-06	cell aging
GO:0008406	6	424	7.2131e-06	gonad development
GO:0000280	7	969	2.43955e-05	nuclear division
GO:0065003	10	3681	2.91009e-05	macromolecular complex assembly
GO:0048285	7	1056	4.40942e-05	organelle fission
GO:0008584	5	300	8.74386e-05	male gonad development
GO:1901987	6	721	0.00016894	regulation of cell cycle phase transition
GO:0048609	8	2109	0.000203262	multicellular organismal reproductive process
GO:0006461	9	3196	0.00023226	protein complex assembly
GO:0051094	9	3200	0.00023481	positive regulation of developmental process
GO:0051248	8	2189	0.000271454	negative regulation of protein metabolic process
GO:0051302	6	796	0.000302927	regulation of cell division
GO:0051147	5	413	0.000427745	regulation of muscle cell differentiation
GO:0007276	7	1582	0.000699934	gamete generation
GO:0045597	8	2514	0.000793127	positive regulation of cell differentiation
GO:0045934	9	3691	0.000813397	negative regulation of nucleobase-containing compound metabolic process
GO:0008283	8	2526	0.00082284	cell proliferation
GO:0051172	9	3798	0.00104217	negative regulation of nitrogen compound metabolic process
GO:0022412	6	998	0.00114413	cellular process involved in reproduction in multicellular organism
GO:0097190	6	1052	0.00155745	apoptotic signaling pathway
GO:0045665	4	213	0.00156807	negative regulation of neuron differentiation
GO:0048608	6	1062	0.00164604	reproductive structure development
GO:0007548	3	53	0.00168068	sex differentiation
GO:0044703	5	580	0.00228448	multi-organism reproductive process
GO:1900180	5	602	0.00274292	regulation of protein localization to nucleus
GO:0007067	5	605	0.00281068	mitotic nuclear division
GO:0045726	2	6	0.00361866	positive regulation of integrin biosynthetic process
GO:0006897	6	1228	0.00383958	endocytosis
GO:0021700	5	666	0.00450159	developmental maturation
GO:0048534	5	682	0.00505634	hematopoietic or lymphoid organ development
GO:2000118	2	7	0.00506509	regulation of sodium-dependent phosphate transport
GO:1901990	5	692	0.00542958	regulation of mitotic cell cycle phase transition
GO:0022602	4	294	0.00564744	ovulation cycle process
GO:0048514	4	294	0.00564744	blood vessel morphogenesis
GO:0051153	4	295	0.00572395	regulation of striated muscle cell differentiation
GO:0010948	5	717	0.00645834	negative regulation of cell cycle process
GO:0031400	6	1368	0.0071859	negative regulation of protein modification process
GO:0007283	6	1413	0.00866668	spermatogenesis
GO:0048232	6	1420	0.00891799	male gamete generation
GO:0000122	7	2314	0.00910205	negative regulation of transcription from RNA polymerase II promoter
GO:0008156	3	94	0.00953729	negative regulation of DNA replication
GO:0003006	7	2335	0.00966873	developmental process involved in reproduction
GO:0006366	6	1448	0.0099838	transcription from RNA polymerase II promoter
GO:0042110	5	787	0.010175	T cell activation
GO:0008285	7	2354	0.0102068	negative regulation of cell proliferation
GO:0007420	5	791	0.0104294	brain development
GO:0010629	8	3561	0.0114234	negative regulation of gene expression
GO:0071900	6	1492	0.011868	regulation of protein serine/threonine kinase activity
GO:0042176	5	816	0.0121357	regulation of protein catabolic process
GO:0045786	5	824	0.0127257	negative regulation of cell cycle
GO:2000113	8	3633	0.0132965	negative regulation of cellular macromolecule biosynthetic process
GO:0044702	8	3664	0.0141808	single organism reproductive process
GO:0006275	4	373	0.014473	regulation of DNA replication
GO:0007568	5	848	0.0146325	aging
GO:0051251	5	874	0.0169448	positive regulation of lymphocyte activation
GO:0010558	8	3771	0.0176329	negative regulation of macromolecule biosynthetic process
GO:0035148	4	396	0.0183223	tube formation
GO:0009888	7	2570	0.0183228	tissue development
GO:0016477	7	2574	0.0185132	cell migration
GO:0007517	4	401	0.0192504	muscle organ development

Table 3: Overrepresented terms with the network-based enrichment. Only terms not detected with the standard method.

GO Term	N1	N2	P-value	Description
GO:0045787	4	401	0.0192504	positive regulation of cell cycle
GO:0034622	6	1660	0.0219366	cellular macromolecular complex assembly
GO:0048511	5	930	0.022901	rhythmic process
GO:0002696	5	941	0.0242431	positive regulation of leukocyte activation
GO:0061061	4	426	0.0244221	muscle structure development
GO:0009887	6	1719	0.0268039	organ morphogenesis
GO:0051783	4	444	0.0287351	regulation of nuclear division
GO:0001666	5	987	0.0305403	response to hypoxia
GO:0003002	5	988	0.0306901	regionalization
GO:0051130	7	2782	0.0309951	positive regulation of cellular component organization
GO:1903047	6	1769	0.0315882	mitotic cell cycle process
GO:0050867	5	996	0.03191	positive regulation of cell activation
GO:1901991	4	457	0.0321826	negative regulation of mitotic cell cycle phase transition
GO:0036293	5	999	0.0323771	response to decreased oxygen levels
GO:0048870	7	2808	0.0329619	cell motility
GO:0060429	5	1006	0.0334877	epithelium development
GO:0048469	4	468	0.0353305	cell maturation
GO:0032269	6	1804	0.0353348	negative regulation of cellular protein metabolic process
GO:0009266	4	469	0.0356275	response to temperature stimulus
GO:0006978	2	18	0.0368218	DNA damage response, signal transduction by p53 class mediator resulting in transcription of p21 class mediat[illegible]
GO:0050878	6	1837	0.0391918	regulation of body fluid levels
GO:0016049	4	484	0.0403075	cell growth
GO:0042772	2	19	0.0411455	DNA damage response, signal transduction resulting in transcription
GO:0001822	4	489	0.0419638	kidney development
GO:0031401	7	2947	0.0453439	positive regulation of protein modification process
GO:0045165	4	501	0.0461433	cell fate commitment
GO:0070482	5	1076	0.0463232	response to oxygen levels
GO:0045664	6	1894	0.0466589	regulation of neuron differentiation
GO:0044344	4	505	0.0476023	cellular response to fibroblast growth factor stimulus
GO:0071363	6	1903	0.0479372	cellular response to growth factor stimulus

Table 4: Overrepresented terms with the network-based enrichment. Only terms not detected with the standard method.
