# Supplementary material for: NET-GE: a novel NETwork-based Gene Enrichment for detecting biological processes associated to Mendelian diseases
Source: BMC Genomics. 2015 Jun 18;16(Suppl 8):S6. doi: 10.1186/1471-2164-16-S8-S6 (PMC4480278; doi:10.1186/1471-2164-16-S8-S6)
Supplement: Additional file 3 — Detailed results for the OMIM-derived benchmark set. The archive contains pdf documents listing the enriched terms for each one of the 244 diseases in the OMIM-derived benchmark set. [file 1471-2164-16-S8-S6-S3.tgz › SUPPMAT/OMIM177200.pdf]

#177200 LIDDLE SYNDROME

OMIM Gene ID	HGNC	UniProtAC
600760	SCNN1B	P51168
600761	SCNN1G	P51170

Table 1: OMIM - UniProtAC mapping

Legend

- N1: #input proteins associated to the significant GO term
- N2: #proteins associated to the significant GO term
- P-value: Bonferroni-corrected p-value of Fisher's exact test
- *red*: go terms not related to the input proteins
- *blue*: go terms related to the input proteins (enriched uniquely by network-based method)
- *green*: go terms ancestors of terms enriched with the standard method (enriched uniquely by network-based method)

1 Standard enrichment

GO Term	N1	N2	P-value	Description
GO:0055078	2	26	1.82521e-05	sodium ion homeostasis
GO:0050891	2	36	3.53809e-05	multicellular organismal water homeostasis
GO:0050909	2	40	4.38048e-05	sensory perception of taste
GO:0030104	2	45	5.55984e-05	water homeostasis
GO:0007588	2	51	7.16044e-05	excretion
GO:0055067	2	104	0.000300794	monovalent inorganic cation homeostasis
GO:0007606	2	116	0.000374588	sensory perception of chemical stimulus
GO:0035725	2	140	0.00054644	sodium ion transmembrane transport
GO:0006814	2	233	0.0015179	sodium ion transport
GO:0007600	2	586	0.00962612	sensory perception
GO:0055065	2	593	0.00985768	metal ion homeostasis
GO:0055080	2	656	0.0120654	cation homeostasis
GO:0050801	2	709	0.0140954	ion homeostasis
GO:0050878	2	717	0.0144155	regulation of body fluid levels
GO:0015672	2	743	0.0154807	monovalent inorganic cation transport
GO:0098662	2	848	0.0201687	inorganic cation transmembrane transport
GO:0098660	2	995	0.0277721	inorganic ion transmembrane transport
GO:0030001	2	1036	0.0301092	metal ion transport
GO:0050877	2	1063	0.0316998	neurological system process
GO:0098655	2	1072	0.0322391	cation transmembrane transport
GO:0048878	2	1094	0.0335766	chemical homeostasis

Table 2: Overrepresented GO terms with the standard enrichment

2 Network-based enrichment

GO Term	N1	N2	P-value	Description
GO:1903362	2	339	0.0142471	regulation of cellular protein catabolic process

Table 3: Overrepresented terms with the network-based enrichment. Only terms not detected with the standard method.
