# Supplementary material for: NET-GE: a novel NETwork-based Gene Enrichment for detecting biological processes associated to Mendelian diseases
Source: BMC Genomics. 2015 Jun 18;16(Suppl 8):S6. doi: 10.1186/1471-2164-16-S8-S6 (PMC4480278; doi:10.1186/1471-2164-16-S8-S6)
Supplement: Additional file 3 — Detailed results for the OMIM-derived benchmark set. The archive contains pdf documents listing the enriched terms for each one of the 244 diseases in the OMIM-derived benchmark set. [file 1471-2164-16-S8-S6-S3.tgz › SUPPMAT/OMIM180800.pdf]

#180800 ROUSSY-LEVY HEREDITARY AREFLEXIC DYSTASIA

OMIM Gene ID	HGNC	UniProtAC
159440	MPZ	P25189
601097	PMP22	Q01453

Table 1: OMIM - UniProtAC mapping

Legend

- N1: #input proteins associated to the significant GO term
- N2: #proteins associated to the significant GO term
- P-value: Bonferroni-corrected p-value of Fisher's exact test
- *red*: go terms not related to the input proteins
- *blue*: go terms related to the input proteins (enriched uniquely by network-based method)
- *green*: go terms ancestors of terms enriched with the standard method (enriched uniquely by network-based method)

1 Standard enrichment

GO Term	N1	N2	P-value	Description
GO:0007268	2	530	0.00433004	synaptic transmission
GO:0007267	2	859	0.0113826	cell-cell signaling
GO:0045217	1	11	0.0128219	cell-cell junction maintenance
GO:0023052	2	913	0.0128596	signaling
GO:0044700	2	913	0.0128596	single organism signaling
GO:0034331	1	14	0.0163181	cell junction maintenance
GO:0007154	2	1103	0.0187723	cell communication
GO:0008219	2	1106	0.0188746	cell death
GO:0016265	2	1117	0.0192521	death
GO:0043954	1	26	0.0303002	cellular component maintenance

Table 2: Overrepresented GO terms with the standard enrichment

2 Network-based enrichment

GO Term	N1	N2	P-value	Description
GO:0007422	2	102	0.000510526	peripheral nervous system development

Table 3: Overrepresented terms with the network-based enrichment. Only terms not detected with the standard method.
