# Supplementary material for: NET-GE: a novel NETwork-based Gene Enrichment for detecting biological processes associated to Mendelian diseases
Source: BMC Genomics. 2015 Jun 18;16(Suppl 8):S6. doi: 10.1186/1471-2164-16-S8-S6 (PMC4480278; doi:10.1186/1471-2164-16-S8-S6)
Supplement: Additional file 3 — Detailed results for the OMIM-derived benchmark set. The archive contains pdf documents listing the enriched terms for each one of the 244 diseases in the OMIM-derived benchmark set. [file 1471-2164-16-S8-S6-S3.tgz › SUPPMAT/OMIM181500.pdf]

#181500 SCHIZOPHRENIA; SCZD

**OMIM Gene ID**	**HGNC**	**UniProtAC**
116790	COMT	P21964
124050	DAO	P14920
126451	DRD3	P35462
164730	AKT1	P31749
182135	HTR2A	P28223
600755	SYN2	Q92777
601525	CHI3L1	P36222
605210	DISC1	Q9NRI5
605566	RTN4R	Q9BZR6
607093	MTHFR	P42898
607145	DTNBP1	Q96EV8
607252	APOL2	Q9BQE5
607254	APOL4	Q9BPW4
607408	DAOA	P59103

Table 1: OMIM - UniProtAC mapping

Legend

- N1: #input proteins associated to the significant GO term
- N2: #proteins associated to the significant GO term
- P-value: Bonferroni-corrected p-value of Fisher's exact test
- *red*: go terms not related to the input proteins
- *blue*: go terms related to the input proteins (enriched uniquely by network-based method)
- *green*: go terms ancestors of terms enriched with the standard method (enriched uniquely by network-based method)

1 Standard enrichment

GO Term	N1	N2	P-value	Description
GO:0014059	3	24	8.97026e-05	regulation of dopamine secretion
GO:0042417	3	35	0.000289374	dopamine metabolic process
GO:0050433	3	50	0.000863737	regulation of catecholamine secretion
GO:0006584	3	53	0.00103166	catecholamine metabolic process
GO:0009712	3	53	0.00103166	catechol-containing compound metabolic process
GO:0060159	2	7	0.00293751	regulation of dopamine receptor signaling pathway
GO:0051952	3	84	0.00416788	regulation of amine transport
GO:0018958	3	128	0.0147893	phenol-containing compound metabolic process
GO:0048148	2	16	0.0167538	behavioral response to cocaine
GO:0007268	4	530	0.0377179	synaptic transmission
GO:0009605	6	1995	0.0493009	response to external stimulus

Table 2: Overrepresented GO terms with the standard enrichment

2 Network-based enrichment

GO Term	N1	N2	P-value	Description
GO:0051341	5	250	0.000120622	regulation of oxidoreductase activity
GO:0050768	5	457	0.0023926	negative regulation of neurogenesis
GO:0030307	5	468	0.00268942	positive regulation of cell growth
GO:0050804	6	931	0.00323494	regulation of synaptic transmission
GO:0051588	4	214	0.00438782	regulation of neurotransmitter transport
GO:0051354	3	64	0.00675142	negative regulation of oxidoreductase activity
GO:0070555	4	246	0.00763109	response to interleukin-1
GO:0043278	3	67	0.00775698	response to morphine
GO:0030815	3	70	0.00885714	negative regulation of cAMP metabolic process
GO:0010721	5	609	0.00976663	negative regulation of cell development
GO:0014072	3	74	0.0104784	response to isoquinoline alkaloid
GO:0030800	3	75	0.0109123	negative regulation of cyclic nucleotide metabolic process
GO:0033135	4	294	0.0154509	regulation of peptidyl-serine phosphorylation
GO:1901565	8	2901	0.0169435	organonitrogen compound catabolic process
GO:1900543	3	94	0.0215602	negative regulation of purine nucleotide metabolic process
GO:0045927	5	757	0.028111	positive regulation of growth
GO:0045980	3	105	0.0300696	negative regulation of nucleotide metabolic process
GO:2000650	2	13	0.0355285	negative regulation of sodium ion transmembrane transporter activity
GO:0042493	6	1473	0.0455208	response to drug

Table 3: Overrepresented terms with the network-based enrichment. Only terms not detected with the standard method.
