# Supplementary material for: NET-GE: a novel NETwork-based Gene Enrichment for detecting biological processes associated to Mendelian diseases
Source: BMC Genomics. 2015 Jun 18;16(Suppl 8):S6. doi: 10.1186/1471-2164-16-S8-S6 (PMC4480278; doi:10.1186/1471-2164-16-S8-S6)
Supplement: Additional file 3 — Detailed results for the OMIM-derived benchmark set. The archive contains pdf documents listing the enriched terms for each one of the 244 diseases in the OMIM-derived benchmark set. [file 1471-2164-16-S8-S6-S3.tgz › SUPPMAT/OMIM182940.pdf]

#182940 NEURAL TUBE DEFECTS

OMIM Gene ID	HGNC	UniProtAC
158105	CCL2	P13500
600533	VANGL2	Q9ULK5
601397	T	O15178
610132	VANGL1	Q8TAA9
610622	FUZ	Q9BT04

Table 1: OMIM - UniProtAC mapping

Legend

- N1: #input proteins associated to the significant GO term
- N2: #proteins associated to the significant GO term
- P-value: Bonferroni-corrected p-value of Fisher's exact test
- *red*: go terms not related to the input proteins
- *blue*: go terms related to the input proteins (enriched uniquely by network-based method)
- *green*: go terms ancestors of terms enriched with the standard method (enriched uniquely by network-based method)

1 Standard enrichment

GO Term	N1	N2	P-value	Description
GO:0001843	3	120	0.000185623	neural tube closure
GO:0060606	3	122	0.000195125	tube closure
GO:0009887	4	653	0.000260525	organ morphogenesis
GO:0035148	3	178	0.000609429	tube formation
GO:0001736	2	16	0.00100172	establishment of planar polarity
GO:0007164	2	16	0.00100172	establishment of tissue polarity
GO:0036342	2	24	0.00230297	post-anal tail morphogenesis
GO:0048646	4	1201	0.00295834	anatomical structure formation involved in morphogenesis
GO:0035058	2	36	0.00525344	nonmotile primary cilium assembly
GO:0003002	3	380	0.00593508	regionalization
GO:0001942	2	51	0.0106235	hair follicle development
GO:0007389	3	579	0.0208847	pattern specification process
GO:0048598	3	638	0.027889	embryonic morphogenesis
GO:0009653	4	2131	0.0287928	anatomical structure morphogenesis
GO:0022404	2	99	0.0403165	molting cycle process
GO:0022405	2	99	0.0403165	hair cycle process

Table 2: Overrepresented GO terms with the standard enrichment

2 Network-based enrichment

GO Term	N1	N2	P-value	Description
GO:2000427	2	15	0.00172617	positive regulation of apoptotic cell clearance
GO:2000425	2	27	0.0057662	regulation of apoptotic cell clearance
GO:0061311	2	51	0.0209154	cell surface receptor signaling pathway involved in heart development
GO:0022604	4	1615	0.0241489	regulation of cell morphogenesis
GO:0045860	4	1636	0.0254176	positive regulation of protein kinase activity
GO:0033674	4	1706	0.030007	positive regulation of kinase activity
GO:0051347	4	1933	0.0491969	positive regulation of transferase activity

Table 3: Overrepresented terms with the network-based enrichment. Only terms not detected with the standard method.
