# Supplementary material for: NET-GE: a novel NETwork-based Gene Enrichment for detecting biological processes associated to Mendelian diseases
Source: BMC Genomics. 2015 Jun 18;16(Suppl 8):S6. doi: 10.1186/1471-2164-16-S8-S6 (PMC4480278; doi:10.1186/1471-2164-16-S8-S6)
Supplement: Additional file 3 — Detailed results for the OMIM-derived benchmark set. The archive contains pdf documents listing the enriched terms for each one of the 244 diseases in the OMIM-derived benchmark set. [file 1471-2164-16-S8-S6-S3.tgz › SUPPMAT/OMIM187500.pdf]

#187500 TETRALOGY OF FALLOT; TOF

**OMIM Gene ID**	**HGNC**	**UniProtAC**
600584	NKX2-5	P52952
601656	GATA6	Q92908
601920	JAG1	P78504
602054	TBX1	O43435
602880	GDF1	P27539
603693	ZFPM2	Q8WW38

Table 1: OMIM - UniProtAC mapping

Legend

- N1: #input proteins associated to the significant GO term
- N2: #proteins associated to the significant GO term
- P-value: Bonferroni-corrected p-value of Fisher's exact test
- *red*: go terms not related to the input proteins
- *blue*: go terms related to the input proteins (enriched uniquely by network-based method)
- *green*: go terms ancestors of terms enriched with the standard method (enriched uniquely by network-based method)

1 Standard enrichment

GO Term	N1	N2	P-value	Description
GO:0060411	5	61	3.35223e-11	cardiac septum morphogenesis
GO:0003148	4	18	3.25567e-10	outflow tract septum morphogenesis
GO:0003221	2	2	1.26361e-05	right ventricular cardiac muscle tissue morphogenesis
GO:0060415	3	71	7.62342e-05	muscle tissue morphogenesis
GO:0051891	2	5	0.000126334	positive regulation of cardioblast differentiation
GO:0045944	5	1312	0.000176146	positive regulation of transcription from RNA polymerase II promoter
GO:0051094	5	1326	0.000185702	positive regulation of developmental process
GO:2000736	3	117	0.000346014	regulation of stem cell differentiation
GO:0051890	2	9	0.000454673	regulation of cardioblast differentiation
GO:0048729	4	578	0.000478111	tissue morphogenesis
GO:0035239	3	141	0.000607422	tube morphogenesis
GO:0045893	5	1762	0.000763152	positive regulation of transcription, DNA-templated
GO:0042693	2	12	0.000833388	muscle cell fate commitment
GO:1902680	5	1811	0.000874488	positive regulation of RNA biosynthetic process
GO:0051254	5	1838	0.000941142	positive regulation of RNA metabolic process
GO:0048513	5	1910	0.00113884	organ development
GO:2000738	2	14	0.0011489	positive regulation of stem cell differentiation
GO:0010628	5	1919	0.00116571	positive regulation of gene expression
GO:0010557	5	2042	0.00158635	positive regulation of macromolecule biosynthetic process
GO:0045935	5	2060	0.00165689	positive regulation of nucleobase-containing compound metabolic process
GO:0042692	3	200	0.00173838	muscle cell differentiation
GO:0016202	3	203	0.00181787	regulation of striated muscle tissue development
GO:1901861	3	204	0.00184489	regulation of muscle tissue development
GO:0051173	5	2108	0.00185727	positive regulation of nitrogen compound metabolic process
GO:0045595	5	2111	0.00187041	regulation of cell differentiation
GO:0048634	3	207	0.00192755	regulation of muscle organ development
GO:0009653	5	2131	0.00195989	anatomical structure morphogenesis
GO:0045596	4	834	0.00205631	negative regulation of cell differentiation
GO:0031328	5	2177	0.00217864	positive regulation of cellular biosynthetic process
GO:0009891	5	2208	0.00233671	positive regulation of biosynthetic process
GO:2000026	5	2229	0.00244887	regulation of multicellular organismal development
GO:0006357	5	2379	0.00338062	regulation of transcription from RNA polymerase II promoter
GO:0035909	2	25	0.00378466	aorta morphogenesis
GO:0060037	2	25	0.00378466	pharyngeal system development
GO:0030154	5	2446	0.00387862	cell differentiation
GO:0009888	4	984	0.00396341	tissue development
GO:0045597	4	985	0.00397939	positive regulation of cell differentiation
GO:0060412	2	26	0.00409976	ventricular septum morphogenesis
GO:0048534	3	272	0.00437147	hematopoietic or lymphoid organ development
GO:0051093	4	1051	0.00514528	negative regulation of developmental process
GO:0030878	2	34	0.0070728	thyroid gland development
GO:0060548	4	1147	0.00727194	negative regulation of cell death
GO:0048646	4	1201	0.00872274	anatomical structure formation involved in morphogenesis
GO:0050793	5	2884	0.00875346	regulation of developmental process
GO:0048732	3	391	0.0129365	gland development
GO:0003007	2	50	0.0154267	heart morphogenesis
GO:0010604	5	3285	0.0166317	positive regulation of macromolecule metabolic process
GO:0055010	2	53	0.0173498	ventricular cardiac muscle tissue morphogenesis
GO:0001974	2	55	0.0186944	blood vessel remodeling
GO:0060043	2	56	0.0193854	regulation of cardiac muscle cell proliferation
GO:0030855	3	451	0.0198014	epithelial cell differentiation
GO:0031325	5	3418	0.0202206	positive regulation of cellular metabolic process
GO:0051239	5	3432	0.0206315	regulation of multicellular organismal process
GO:0055006	2	58	0.0208049	cardiac cell development
GO:0055007	2	59	0.0215333	cardiac muscle cell differentiation
GO:0055021	2	62	0.0237937	regulation of cardiac muscle tissue growth
GO:0001709	2	64	0.0253629	cell fate determination
GO:0048844	2	65	0.0261662	artery morphogenesis
GO:0055008	2	65	0.0261662	cardiac muscle tissue morphogenesis
GO:0009893	5	3630	0.0271855	positive regulation of metabolic process

Table 2: Overrepresented GO terms with the standard enrichment

GO Term	N1	N2	P-value	Description
GO:0007498	2	67	0.0278102	mesoderm development
GO:0048869	5	3694	0.0296239	cellular developmental process
GO:0060420	2	70	0.0303697	regulation of heart growth
GO:0045599	2	72	0.0321383	negative regulation of fat cell differentiation
GO:0035051	2	76	0.0358249	cardiocyte differentiation
GO:0055024	2	77	0.0367776	regulation of cardiac muscle tissue development
GO:0060485	2	77	0.0367776	mesenchyme development

Table 3: Overrepresented GO terms with the standard enrichment

2 Network-based enrichment

GO Term	N1	N2	P-value	Description
GO:0001701	5	893	6.36773e-05	in utero embryonic development
GO:0043009	5	909	6.95753e-05	chordate embryonic development
GO:0009792	5	937	8.09407e-05	embryo development ending in birth or egg hatching
GO:0009790	5	1155	0.000229521	embryo development
GO:0010621	2	6	0.000321425	negative regulation of transcription by transcription factor localization
GO:0003151	3	129	0.000891919	outflow tract morphogenesis
GO:0030182	4	911	0.00632372	neuron differentiation
GO:0001570	3	258	0.00715676	vasculogenesis
GO:0003215	2	28	0.00808567	cardiac right ventricle morphogenesis
GO:1902533	5	2418	0.0089701	positive regulation of intracellular signal transduction
GO:2000018	2	30	0.00930348	regulation of male gonad development
GO:0048514	3	294	0.0105794	blood vessel morphogenesis
GO:0090288	3	301	0.0113505	negative regulation of cellular response to growth factor stimulus
GO:0048731	5	2612	0.0131301	system development
GO:0014706	3	333	0.0153505	striated muscle tissue development
GO:0002009	4	1159	0.0163888	morphogenesis of an epithelium
GO:0001101	4	1170	0.0170115	response to acid chemical
GO:0060537	3	387	0.0240367	muscle tissue development
GO:0007389	4	1481	0.0430524	pattern specification process
GO:0001932	5	3361	0.0454277	regulation of protein phosphorylation
GO:0003208	2	68	0.048572	cardiac ventricle morphogenesis

Table 4: Overrepresented terms with the network-based enrichment. Only terms not detected with the standard method.
