# Supplementary material for: NET-GE: a novel NETwork-based Gene Enrichment for detecting biological processes associated to Mendelian diseases
Source: BMC Genomics. 2015 Jun 18;16(Suppl 8):S6. doi: 10.1186/1471-2164-16-S8-S6 (PMC4480278; doi:10.1186/1471-2164-16-S8-S6)
Supplement: Additional file 3 — Detailed results for the OMIM-derived benchmark set. The archive contains pdf documents listing the enriched terms for each one of the 244 diseases in the OMIM-derived benchmark set. [file 1471-2164-16-S8-S6-S3.tgz › SUPPMAT/OMIM187800-OMIM273800.pdf]

#187800 BLEEDING DISORDER, PLATELET-TYPE, 16; BDPLT16 #273800 GLANZMANN THROMBASTHENIA; GT

**OMIM Gene ID**	**HGNC**	**UniProtAC**
173470	ITGB3	P05106
607759	ITGA2B	P08514

Table 1: OMIM - UniProtAC mapping

Legend

- N1: #input proteins associated to the significant GO term
- N2: #proteins associated to the significant GO term
- P-value: Bonferroni-corrected p-value of Fisher's exact test
- *red*: go terms not related to the input proteins
- *blue*: go terms related to the input proteins (enriched uniquely by network-based method)
- *green*: go terms ancestors of terms enriched with the standard method (enriched uniquely by network-based method)

1 Standard enrichment

GO Term	N1	N2	P-value	Description
GO:0070527	2	48	0.000326246	platelet aggregation
GO:0034109	2	67	0.000639478	homotypic cell-cell adhesion
GO:0002576	2	82	0.000960516	platelet degranulation
GO:0007229	2	166	0.00396093	integrin-mediated signaling pathway
GO:0030168	2	216	0.00671581	platelet activation
GO:0007160	2	228	0.00748456	cell-matrix adhesion
GO:0031589	2	292	0.012288	cell-substrate adhesion
GO:0006887	2	346	0.0172624	exocytosis
GO:0030335	2	384	0.0212685	positive regulation of cell migration
GO:0016337	2	388	0.0217145	single organismal cell-cell adhesion
GO:2000147	2	393	0.0222785	positive regulation of cell motility
GO:0051272	2	405	0.0236616	positive regulation of cellular component movement
GO:0040017	2	414	0.0247262	positive regulation of locomotion
GO:0098602	2	425	0.0260592	single organism cell adhesion
GO:0007411	2	476	0.0326969	axon guidance
GO:0045715	1	3	0.032747	negative regulation of low-density lipoprotein particle receptor biosynthetic process
GO:0097485	2	477	0.0328345	neuron projection guidance
GO:0030198	2	486	0.0340866	extracellular matrix organization
GO:0043062	2	487	0.0342271	extracellular structure organization
GO:0007596	2	501	0.0362255	blood coagulation
GO:0050817	2	501	0.0362255	coagulation
GO:0007599	2	510	0.03754	hemostasis

Table 2: Overrepresented GO terms with the standard enrichment

2 Network-based enrichment

No novel enriched terms
