# Supplementary material for: NET-GE: a novel NETwork-based Gene Enrichment for detecting biological processes associated to Mendelian diseases
Source: BMC Genomics. 2015 Jun 18;16(Suppl 8):S6. doi: 10.1186/1471-2164-16-S8-S6 (PMC4480278; doi:10.1186/1471-2164-16-S8-S6)
Supplement: Additional file 3 — Detailed results for the OMIM-derived benchmark set. The archive contains pdf documents listing the enriched terms for each one of the 244 diseases in the OMIM-derived benchmark set. [file 1471-2164-16-S8-S6-S3.tgz › SUPPMAT/OMIM188890.pdf]

#188890 TOBACCO ADDICTION, SUSCEPTIBILITY TO

OMIM Gene ID	HGNC	UniProtAC
118504	CHRNA4	P43681
122720	CYP2A6	P11509
126455	SLC6A3	Q01959
607340	GABBR2	O75899

Table 1: OMIM - UniProtAC mapping

Legend

- N1: #input proteins associated to the significant GO term
- N2: #proteins associated to the significant GO term
- P-value: Bonferroni-corrected p-value of Fisher's exact test
- *red*: go terms not related to the input proteins
- *blue*: go terms related to the input proteins (enriched uniquely by network-based method)
- *green*: go terms ancestors of terms enriched with the standard method (enriched uniquely by network-based method)

1 Standard enrichment

GO Term	N1	N2	P-value	Description
GO:0035094	2	47	0.00218206	response to nicotine
GO:0007268	3	530	0.00261571	synaptic transmission
GO:0007267	3	859	0.0110868	cell-cell signaling
GO:0023052	3	913	0.0133001	signaling
GO:0044700	3	913	0.0133001	single organism signaling
GO:0007154	3	1103	0.0233745	cell communication
GO:0043279	2	188	0.0353054	response to alkaloid

Table 2: Overrepresented GO terms with the standard enrichment

2 Network-based enrichment

GO Term	N1	N2	P-value	Description
GO:0035176	2	119	0.0378543	social behavior
GO:0051703	2	119	0.0378543	intraspecies interaction between organisms
GO:0042220	2	127	0.0431242	response to cocaine

Table 3: Overrepresented terms with the network-based enrichment. Only terms not detected with the standard method.
