# Supplementary material for: NET-GE: a novel NETwork-based Gene Enrichment for detecting biological processes associated to Mendelian diseases
Source: BMC Genomics. 2015 Jun 18;16(Suppl 8):S6. doi: 10.1186/1471-2164-16-S8-S6 (PMC4480278; doi:10.1186/1471-2164-16-S8-S6)
Supplement: Additional file 3 — Detailed results for the OMIM-derived benchmark set. The archive contains pdf documents listing the enriched terms for each one of the 244 diseases in the OMIM-derived benchmark set. [file 1471-2164-16-S8-S6-S3.tgz › SUPPMAT/OMIM191830.pdf]

#191830 RENAL HYPODYSPLASIA/APLASIA 1; RHDA1

**OMIM Gene ID**	**HGNC**	**UniProtAC**
164761	RET	P07949
167409	PAX2	Q02962
604063	ITGA8	P53708

Table 1: OMIM - UniProtAC mapping

Legend

- N1: #input proteins associated to the significant GO term
- N2: #proteins associated to the significant GO term
- P-value: Bonferroni-corrected p-value of Fisher's exact test
- *red*: go terms not related to the input proteins
- *blue*: go terms related to the input proteins (enriched uniquely by network-based method)
- *green*: go terms ancestors of terms enriched with the standard method (enriched uniquely by network-based method)

1 Standard enrichment

GO Term	N1	N2	P-value	Description
GO:0001656	3	47	8.57774e-07	metanephros development
GO:0072216	2	3	5.98937e-06	positive regulation of metanephros development
GO:0072300	2	3	5.98937e-06	positive regulation of metanephric glomerulus development
GO:0090193	2	4	1.19785e-05	positive regulation of glomerulus development
GO:0035799	2	6	2.99453e-05	ureter maturation
GO:0048799	2	6	2.99453e-05	organ maturation
GO:0072298	2	6	2.99453e-05	regulation of metanephric glomerulus development
GO:0001822	3	181	5.14171e-05	kidney development
GO:0090184	2	11	0.00010979	positive regulation of kidney development
GO:0090192	2	14	0.000181642	regulation of glomerulus development
GO:0001838	2	36	0.00125704	embryonic epithelial tube formation
GO:0072175	2	37	0.00132884	epithelial tube formation
GO:0072215	2	37	0.00132884	regulation of metanephros development
GO:0071695	2	45	0.00197503	anatomical structure maturation
GO:0072164	2	70	0.00481575	mesonephric tubule development
GO:0072163	2	71	0.00495524	mesonephric epithelium development
GO:0042472	2	72	0.00509673	inner ear morphogenesis
GO:0090183	2	76	0.0056826	regulation of kidney development
GO:0071300	2	80	0.00630022	cellular response to retinoic acid
GO:0009888	3	984	0.00837458	tissue development
GO:0072073	2	97	0.00928007	kidney epithelium development
GO:0032526	2	158	0.0246943	response to retinoic acid
GO:0035148	2	178	0.031353	tube formation
GO:0061360	1	1	0.0376758	optic chiasma development
GO:2000595	1	1	0.0376758	regulation of optic nerve formation
GO:2000597	1	1	0.0376758	positive regulation of optic nerve formation
GO:0000904	2	211	0.0440691	cell morphogenesis involved in differentiation
GO:0045893	3	1762	0.0481484	positive regulation of transcription, DNA-templated

Table 2: Overrepresented GO terms with the standard enrichment

2 Network-based enrichment

GO Term	N1	N2	P-value	Description
GO:0048484	2	28	0.00158494	enteric nervous system development
GO:0090287	3	557	0.00360748	regulation of cellular response to growth factor stimulus
GO:0061138	3	624	0.00507509	morphogenesis of a branching epithelium
GO:0072182	2	53	0.005775	regulation of nephron tubule epithelial cell differentiation
GO:0035295	3	664	0.00611675	tube development
GO:0001763	3	690	0.00686496	morphogenesis of a branching structure
GO:2000696	2	62	0.0079235	regulation of epithelial cell differentiation involved in kidney development
GO:0000902	3	800	0.0107059	cell morphogenesis
GO:0061005	2	79	0.0129053	cell differentiation involved in kidney development
GO:0016337	3	900	0.0152496	single organismal cell-cell adhesion
GO:0031128	2	86	0.0153075	developmental induction
GO:0045168	2	90	0.0167719	cell-cell signaling involved in cell fate commitment
GO:0098602	3	981	0.0197542	single organism cell adhesion
GO:0060429	3	1006	0.0213048	epithelium development
GO:0001755	2	124	0.031914	neural crest cell migration
GO:0002009	3	1159	0.0325917	morphogenesis of an epithelium
GO:0001657	2	133	0.0367284	ureteric bud development
GO:0021675	2	133	0.0367284	nerve development
GO:0030155	3	1217	0.0377383	regulation of cell adhesion
GO:0007411	3	1242	0.0401141	axon guidance
GO:0097485	3	1243	0.0402111	neuron projection guidance
GO:0071333	2	148	0.0455014	cellular response to glucose stimulus
GO:0001658	2	155	0.0499157	branching involved in ureteric bud morphogenesis

Table 3: Overrepresented terms with the network-based enrichment. Only terms not detected with the standard method.
