# Supplementary material for: NET-GE: a novel NETwork-based Gene Enrichment for detecting biological processes associated to Mendelian diseases
Source: BMC Genomics. 2015 Jun 18;16(Suppl 8):S6. doi: 10.1186/1471-2164-16-S8-S6 (PMC4480278; doi:10.1186/1471-2164-16-S8-S6)
Supplement: Additional file 3 — Detailed results for the OMIM-derived benchmark set. The archive contains pdf documents listing the enriched terms for each one of the 244 diseases in the OMIM-derived benchmark set. [file 1471-2164-16-S8-S6-S3.tgz › SUPPMAT/OMIM192600.pdf]

#192600 CARDIOMYOPATHY, FAMILIAL HYPERTROPHIC, 1; CMH1

**OMIM Gene ID**	**HGNC**	**UniProtAC**
160760	MYH7	P12883
601253	CAV3	P56539
606566	MYLK2	Q9H1R3

Table 1: OMIM - UniProtAC mapping

Legend

- N1: #input proteins associated to the significant GO term
- N2: #proteins associated to the significant GO term
- P-value: Bonferroni-corrected p-value of Fisher's exact test
- *red*: go terms not related to the input proteins
- *blue*: go terms related to the input proteins (enriched uniquely by network-based method)
- *green*: go terms ancestors of terms enriched with the standard method (enriched uniquely by network-based method)

1 Standard enrichment

GO Term	N1	N2	P-value	Description
GO:0044057	3	554	0.00140922	regulation of system process
GO:0055008	2	65	0.00392056	cardiac muscle tissue morphogenesis
GO:0060415	2	71	0.00468344	muscle tissue morphogenesis
GO:0002027	2	89	0.00737807	regulation of heart rate
GO:0006941	2	96	0.00859036	striated muscle contraction
GO:0006937	2	178	0.0296332	regulation of muscle contraction
GO:0038009	1	1	0.0356092	regulation of signal transduction by receptor internalization
GO:0051394	1	1	0.0356092	regulation of nerve growth factor receptor activity
GO:0060299	1	1	0.0356092	negative regulation of sarcomere organization
GO:0008016	2	202	0.0381723	regulation of heart contraction
GO:0090257	2	221	0.0456951	regulation of muscle system process

Table 2: Overrepresented GO terms with the standard enrichment

2 Network-based enrichment

GO Term	N1	N2	P-value	Description
GO:0051147	3	413	0.0012692	regulation of muscle cell differentiation
GO:0060297	2	32	0.00179816	regulation of sarcomere organization
GO:0006936	3	640	0.00473526	muscle contraction
GO:0003012	3	815	0.00978848	muscle system process
GO:0002026	2	76	0.0103231	regulation of the force of heart contraction
GO:0060048	2	106	0.020145	cardiac muscle contraction
GO:1902115	2	130	0.0303387	regulation of organelle assembly

Table 3: Overrepresented terms with the network-based enrichment. Only terms not detected with the standard method.
