# Supplementary material for: NET-GE: a novel NETwork-based Gene Enrichment for detecting biological processes associated to Mendelian diseases
Source: BMC Genomics. 2015 Jun 18;16(Suppl 8):S6. doi: 10.1186/1471-2164-16-S8-S6 (PMC4480278; doi:10.1186/1471-2164-16-S8-S6)
Supplement: Additional file 3 — Detailed results for the OMIM-derived benchmark set. The archive contains pdf documents listing the enriched terms for each one of the 244 diseases in the OMIM-derived benchmark set. [file 1471-2164-16-S8-S6-S3.tgz › SUPPMAT/OMIM193300.pdf]

#193300 VON HIPPEL-LINDAU SYNDROME; VHL

OMIM Gene ID	HGNC	UniProtAC
168461	CCND1	P24385
608537	VHL	P40337

Table 1: OMIM - UniProtAC mapping

Legend

- N1: #input proteins associated to the significant GO term
- N2: #proteins associated to the significant GO term
- P-value: Bonferroni-corrected p-value of Fisher's exact test
- *red*: go terms not related to the input proteins
- *blue*: go terms related to the input proteins (enriched uniquely by network-based method)
- *green*: go terms ancestors of terms enriched with the standard method (enriched uniquely by network-based method)

1 Standard enrichment

GO Term	N1	N2	P-value	Description
GO:0000320	1	3	0.0478488	re-entry into mitotic cell cycle

Table 2: Overrepresented GO terms with the standard enrichment

2 Network-based enrichment

GO Term	N1	N2	P-value	Description
GO:0044773	2	85	0.0099201	mitotic DNA damage checkpoint
GO:0043618	2	90	0.0111288	regulation of transcription from RNA polymerase II promoter in response to stress
GO:0033762	2	95	0.0124071	response to glucagon
GO:0044774	2	97	0.0129378	mitotic DNA integrity checkpoint
GO:0043620	2	105	0.0151719	regulation of DNA-templated transcription in response to stress
GO:0007595	2	138	0.0262674	lactation

Table 3: Overrepresented terms with the network-based enrichment. Only terms not detected with the standard method.
