# Supplementary material for: NET-GE: a novel NETwork-based Gene Enrichment for detecting biological processes associated to Mendelian diseases
Source: BMC Genomics. 2015 Jun 18;16(Suppl 8):S6. doi: 10.1186/1471-2164-16-S8-S6 (PMC4480278; doi:10.1186/1471-2164-16-S8-S6)
Supplement: Additional file 3 — Detailed results for the OMIM-derived benchmark set. The archive contains pdf documents listing the enriched terms for each one of the 244 diseases in the OMIM-derived benchmark set. [file 1471-2164-16-S8-S6-S3.tgz › SUPPMAT/OMIM194070.pdf]

#194070 WILMS TUMOR 1; WT1

OMIM Gene ID	HGNC	UniProtAC
300037	GPC3	P51654
600185	BRCA2	P51587
607102	WT1	P19544

Table 1: OMIM - UniProtAC mapping

Legend

- N1: #input proteins associated to the significant GO term
- N2: #proteins associated to the significant GO term
- P-value: Bonferroni-corrected p-value of Fisher's exact test
- *red*: go terms not related to the input proteins
- *blue*: go terms related to the input proteins (enriched uniquely by network-based method)
- *green*: go terms ancestors of terms enriched with the standard method (enriched uniquely by network-based method)

1 Standard enrichment

GO Term	N1	N2	P-value	Description
GO:0001658	2	78	0.005899	branching involved in ureteric bud morphogenesis
GO:0008285	3	920	0.00674199	negative regulation of cell proliferation
GO:0050680	2	159	0.0246391	negative regulation of epithelial cell proliferation
GO:0008406	2	168	0.0275123	gonad development
GO:0072302	1	1	0.0371194	negative regulation of metanephric glomerular mesangial cell proliferation

Table 2: Overrepresented GO terms with the standard enrichment

2 Network-based enrichment

GO Term	N1	N2	P-value	Description
GO:0048608	3	1062	0.0151509	reproductive structure development
GO:0007389	3	1481	0.0411223	pattern specification process

Table 3: Overrepresented terms with the network-based enrichment. Only terms not detected with the standard method.
