# Supplementary material for: NET-GE: a novel NETwork-based Gene Enrichment for detecting biological processes associated to Mendelian diseases
Source: BMC Genomics. 2015 Jun 18;16(Suppl 8):S6. doi: 10.1186/1471-2164-16-S8-S6 (PMC4480278; doi:10.1186/1471-2164-16-S8-S6)
Supplement: Additional file 3 — Detailed results for the OMIM-derived benchmark set. The archive contains pdf documents listing the enriched terms for each one of the 244 diseases in the OMIM-derived benchmark set. [file 1471-2164-16-S8-S6-S3.tgz › SUPPMAT/OMIM202400.pdf]

#202400 AFIBRINOGENEMIA, CONGENITAL

OMIM Gene ID	HGNC	UniProtAC
134820	FGA	P02671
134830	FGB	P02675

Table 1: OMIM - UniProtAC mapping

Legend

- N1: #input proteins associated to the significant GO term
- N2: #proteins associated to the significant GO term
- P-value: Bonferroni-corrected p-value of Fisher's exact test
- *red*: go terms not related to the input proteins
- *blue*: go terms related to the input proteins (enriched uniquely by network-based method)
- *green*: go terms ancestors of terms enriched with the standard method (enriched uniquely by network-based method)

1 Standard enrichment

GO Term	N1	N2	P-value	Description
GO:0034116	2	9	9.40122e-06	positive regulation of heterotypic cell-cell adhesion
GO:0034114	2	17	3.55158e-05	regulation of heterotypic cell-cell adhesion
GO:2000352	2	23	6.60696e-05	negative regulation of endothelial cell apoptotic process
GO:1900026	2	31	0.000121432	positive regulation of substrate adhesion-dependent cell spreading
GO:1902042	2	34	0.000146502	negative regulation of extrinsic apoptotic signaling pathway via death domain receptors
GO:2000351	2	34	0.000146502	regulation of endothelial cell apoptotic process
GO:0045907	2	37	0.000173922	positive regulation of vasoconstriction
GO:1900024	2	37	0.000173922	regulation of substrate adhesion-dependent cell spreading
GO:0070527	2	48	0.000294572	platelet aggregation
GO:0022409	2	54	0.000373698	positive regulation of cell-cell adhesion
GO:0045921	2	55	0.000387801	positive regulation of exocytosis
GO:1902041	2	59	0.000446819	regulation of extrinsic apoptotic signaling pathway via death domain receptors
GO:0034109	2	67	0.000577392	homotypic cell-cell adhesion
GO:0019229	2	68	0.000594888	regulation of vasoconstriction
GO:0010770	2	73	0.00068629	positive regulation of cell morphogenesis involved in differentiation
GO:0002576	2	82	0.000867262	platelet degranulation
GO:0090277	2	101	0.00131878	positive regulation of peptide hormone secretion
GO:0002793	2	104	0.00139869	positive regulation of peptide secretion
GO:0051258	2	107	0.00148095	protein polymerization
GO:0022407	2	120	0.00186458	regulation of cell-cell adhesion
GO:0046887	2	133	0.00229234	positive regulation of hormone secretion
GO:0051592	2	145	0.00272635	response to calcium ion
GO:0010811	2	150	0.0029183	positive regulation of cell-substrate adhesion
GO:0070374	2	150	0.0029183	positive regulation of ERK1 and ERK2 cascade
GO:0050714	2	162	0.00340559	positive regulation of protein secretion
GO:2001237	2	165	0.00353329	negative regulation of extrinsic apoptotic signaling pathway
GO:0017157	2	167	0.00361973	regulation of exocytosis
GO:0030168	2	216	0.00606379	platelet activation
GO:0007160	2	228	0.0067579	cell-matrix adhesion
GO:0070372	2	231	0.00693732	regulation of ERK1 and ERK2 cascade
GO:0010810	2	243	0.00767845	regulation of cell-substrate adhesion
GO:0045785	2	253	0.00832478	positive regulation of cell adhesion
GO:2001236	2	253	0.00832478	regulation of extrinsic apoptotic signaling pathway
GO:0050708	2	273	0.00969579	regulation of protein secretion
GO:0072377	1	1	0.00985614	blood coagulation, common pathway
GO:2000260	1	1	0.00985614	regulation of blood coagulation, common pathway
GO:2000261	1	1	0.00985614	negative regulation of blood coagulation, common pathway
GO:0090276	2	279	0.0101275	regulation of peptide hormone secretion
GO:0002791	2	284	0.0104944	regulation of peptide secretion
GO:0090087	2	286	0.010643	regulation of peptide transport
GO:2001234	2	286	0.010643	negative regulation of apoptotic signaling pathway
GO:0031589	2	292	0.011095	cell-substrate adhesion
GO:0046883	2	336	0.0146972	regulation of hormone secretion
GO:0006887	2	346	0.0155864	exocytosis
GO:0010720	2	348	0.0157674	positive regulation of cell development
GO:0051222	2	349	0.0158583	positive regulation of protein transport
GO:0010769	2	381	0.0189043	regulation of cell morphogenesis involved in differentiation
GO:0016337	2	388	0.0196063	single organismal cell-cell adhesion
GO:0051047	2	392	0.020013	positive regulation of secretion
GO:0043623	2	393	0.0201155	cellular protein complex assembly
GO:0010038	2	402	0.0210485	response to metal ion
GO:0098602	2	425	0.0235292	single organism cell adhesion
GO:0060627	2	451	0.0264996	regulation of vesicle-mediated transport
GO:0030155	2	485	0.0306506	regulation of cell adhesion
GO:0030198	2	486	0.0307772	extracellular matrix organization
GO:0043062	2	487	0.0309041	extracellular structure organization
GO:0007596	2	501	0.0327085	blood coagulation
GO:0050817	2	501	0.0327085	coagulation
GO:0007599	2	510	0.0338953	hemostasis
GO:0043410	2	539	0.0378636	positive regulation of MAPK cascade

Table 2: Overrepresented GO terms with the standard enrichment

GO Term	N1	N2	P-value	Description
GO:2001233	2	541	0.0381454	regulation of apoptotic signaling pathway
GO:0044057	2	554	0.0400025	regulation of system process
GO:0032940	2	592	0.0456836	secretion by cell
GO:0022604	2	593	0.0458382	regulation of cell morphogenesis
GO:0051223	2	595	0.0461483	regulation of protein transport
GO:0010035	2	612	0.0488252	response to inorganic substance

Table 3: Overrepresented GO terms with the standard enrichment

2 Network-based enrichment

GO Term	N1	N2	P-value	Description
GO:0044320	2	32	0.000331604	cellular response to leptin stimulus
GO:0044321	2	37	0.000445259	response to leptin
GO:0070555	2	246	0.0201469	response to interleukin-1

Table 4: Overrepresented terms with the network-based enrichment. Only terms not detected with the standard method.
