# Supplementary material for: NET-GE: a novel NETwork-based Gene Enrichment for detecting biological processes associated to Mendelian diseases
Source: BMC Genomics. 2015 Jun 18;16(Suppl 8):S6. doi: 10.1186/1471-2164-16-S8-S6 (PMC4480278; doi:10.1186/1471-2164-16-S8-S6)
Supplement: Additional file 3 — Detailed results for the OMIM-derived benchmark set. The archive contains pdf documents listing the enriched terms for each one of the 244 diseases in the OMIM-derived benchmark set. [file 1471-2164-16-S8-S6-S3.tgz › SUPPMAT/OMIM203200.pdf]

#203200 ALBINISM, OCULOCUTANEOUS, TYPE II; OCA2

OMIM Gene ID	HGNC	UniProtAC
155555	MC1R	Q01726
611409	OCA2	Q04671

Table 1: OMIM - UniProtAC mapping

Legend

- N1: #input proteins associated to the significant GO term
- N2: #proteins associated to the significant GO term
- P-value: Bonferroni-corrected p-value of Fisher's exact test
- *red*: go terms not related to the input proteins
- *blue*: go terms related to the input proteins (enriched uniquely by network-based method)
- *green*: go terms ancestors of terms enriched with the standard method (enriched uniquely by network-based method)

1 Standard enrichment

GO Term	N1	N2	P-value	Description
GO:0042438	2	21	5.24817e-05	melanin biosynthetic process
GO:0006582	2	22	5.77299e-05	melanin metabolic process
GO:0044550	2	25	7.4974e-05	secondary metabolite biosynthetic process
GO:0046189	2	48	0.000281902	phenol-containing compound biosynthetic process
GO:0019748	2	61	0.000457341	secondary metabolic process
GO:0046148	2	72	0.000638778	pigment biosynthetic process
GO:0042440	2	94	0.00109237	pigment metabolic process
GO:0018958	2	128	0.0020313	phenol-containing compound metabolic process
GO:1901617	2	203	0.00512396	organic hydroxy compound biosynthetic process
GO:0015828	1	1	0.00943222	tyrosine transport
GO:0006726	1	4	0.0377273	eye pigment biosynthetic process
GO:0015801	1	4	0.0377273	aromatic amino acid transport
GO:0042441	1	4	0.0377273	eye pigment metabolic process
GO:0043324	1	4	0.0377273	pigment metabolic process involved in developmental pigmentation
GO:0043474	1	4	0.0377273	pigment metabolic process involved in pigmentation
GO:0010739	1	5	0.0471586	positive regulation of protein kinase A signaling
GO:1901615	2	621	0.0481107	organic hydroxy compound metabolic process

Table 2: Overrepresented GO terms with the standard enrichment

2 Network-based enrichment

No novel enriched terms
