# Supplementary material for: NET-GE: a novel NETwork-based Gene Enrichment for detecting biological processes associated to Mendelian diseases
Source: BMC Genomics. 2015 Jun 18;16(Suppl 8):S6. doi: 10.1186/1471-2164-16-S8-S6 (PMC4480278; doi:10.1186/1471-2164-16-S8-S6)
Supplement: Additional file 3 — Detailed results for the OMIM-derived benchmark set. The archive contains pdf documents listing the enriched terms for each one of the 244 diseases in the OMIM-derived benchmark set. [file 1471-2164-16-S8-S6-S3.tgz › SUPPMAT/OMIM209880.pdf]

#209880 CENTRAL HYPOVENTILATION SYNDROME, CONGENITAL; CCHS

**OMIM Gene ID**	**HGNC**	**UniProtAC**
100790	ASCL1	P50553
113505	BDNF	P23560
131242	EDN3	P14138
164761	RET	P07949
600837	GDNF	P39905
603851	PHOX2B	Q99453

Table 1: OMIM - UniProtAC mapping

Legend

- N1: #input proteins associated to the significant GO term
- N2: #proteins associated to the significant GO term
- P-value: Bonferroni-corrected p-value of Fisher's exact test
- *red*: go terms not related to the input proteins
- *blue*: go terms related to the input proteins (enriched uniquely by network-based method)
- *green*: go terms ancestors of terms enriched with the standard method (enriched uniquely by network-based method)

1 Standard enrichment

GO Term	N1	N2	P-value	Description
GO:0001755	4	59	5.53479e-08	neural crest cell migration
GO:0048484	3	15	6.97209e-07	enteric nervous system development
GO:0048485	3	17	1.04186e-06	sympathetic nervous system development
GO:0001667	4	211	9.69755e-06	ameboidal cell migration
GO:0006928	6	1973	1.39177e-05	cellular component movement
GO:0048731	5	879	2.73888e-05	system development
GO:0030182	4	298	3.8764e-05	neuron differentiation
GO:0003358	2	3	4.34018e-05	noradrenergic neuron development
GO:0016477	5	1046	6.52291e-05	cell migration
GO:0048870	5	1136	9.84286e-05	cell motility
GO:0060284	5	1166	0.000112079	regulation of cell development
GO:0050793	6	2884	0.000136089	regulation of developmental process
GO:0048483	2	5	0.000144652	autonomic nervous system development
GO:0040011	5	1284	0.00018115	locomotion
GO:0051094	5	1326	0.000212629	positive regulation of developmental process
GO:0010604	6	3285	0.000297399	positive regulation of macromolecule metabolic process
GO:0031325	6	3418	0.00037743	positive regulation of cellular metabolic process
GO:0051239	6	3432	0.000386808	regulation of multicellular organismal process
GO:0009893	6	3630	0.000541695	positive regulation of metabolic process
GO:0048869	6	3694	0.000601628	cellular developmental process
GO:0061549	2	10	0.000650704	sympathetic ganglion development
GO:0061548	2	12	0.000954229	ganglion development
GO:0045787	3	165	0.00111642	positive regulation of cell cycle
GO:0045666	3	168	0.0011786	positive regulation of neuron differentiation
GO:0045664	4	709	0.00123481	regulation of neuron differentiation
GO:0044707	6	4361	0.0016298	single-multicellular organism process
GO:0032501	6	4447	0.00183252	multicellular organismal process
GO:0045595	5	2111	0.00214162	regulation of cell differentiation
GO:2000026	5	2229	0.00280396	regulation of multicellular organismal development
GO:0050767	4	928	0.0035973	regulation of neurogenesis
GO:0014821	2	24	0.00398704	phasic smooth muscle contraction
GO:0009888	4	984	0.0045381	tissue development
GO:0045597	4	985	0.0045564	positive regulation of cell differentiation
GO:0051960	4	1028	0.005397	regulation of nervous system development
GO:0051130	4	1059	0.00607093	positive regulation of cellular component organization
GO:0043523	3	300	0.00671136	regulation of neuron apoptotic process
GO:0048522	6	5768	0.00873239	positive regulation of cellular process
GO:0001838	2	36	0.00909313	embryonic epithelial tube formation
GO:0072175	2	37	0.00961209	epithelial tube formation
GO:0072215	2	37	0.00961209	regulation of metanephros development
GO:0010720	3	348	0.0104601	positive regulation of cell development
GO:1901214	3	348	0.0104601	regulation of neuron death
GO:0001656	2	47	0.0155906	metanephros development
GO:0060563	2	52	0.0191172	neuroepithelial cell differentiation
GO:0048518	6	6624	0.0200377	positive regulation of biological process
GO:0044767	6	6740	0.0222384	single-organism developmental process
GO:0048839	2	59	0.0246557	inner ear development
GO:0003008	4	1588	0.0300529	system process
GO:0001657	2	66	0.0308944	ureteric bud development
GO:0007346	3	518	0.0342443	regulation of mitotic cell cycle
GO:0023051	5	3708	0.0345557	regulation of signaling
GO:0072164	2	70	0.0347733	mesonephric tubule development
GO:0010646	5	3714	0.0348313	regulation of cell communication
GO:0006939	2	71	0.0357788	smooth muscle contraction
GO:0072163	2	71	0.0357788	mesonephric epithelium development
GO:0032502	6	7299	0.0358756	developmental process
GO:0002065	2	76	0.0410195	columnar/cuboidal epithelial cell differentiation
GO:0090183	2	76	0.0410195	regulation of kidney development
GO:0045893	4	1762	0.0452225	positive regulation of transcription, DNA-templated

Table 2: Overrepresented GO terms with the standard enrichment

2 Network-based enrichment

GO Term	N1	N2	P-value	Description
GO:0050930	3	41	3.78227e-05	induction of positive chemotaxis
GO:0045859	6	2438	0.000167377	regulation of protein kinase activity
GO:0043549	6	2576	0.000232977	regulation of kinase activity
GO:0061005	3	79	0.00027986	cell differentiation involved in kidney development
GO:0030855	5	1135	0.000290795	epithelial cell differentiation
GO:0050927	3	84	0.000337096	positive regulation of positive chemotaxis
GO:0050926	3	86	0.00036201	regulation of positive chemotaxis
GO:0051338	6	2819	0.000400337	regulation of transferase activity
GO:0032526	4	457	0.000562172	response to retinoic acid
GO:0001932	6	3361	0.0011509	regulation of protein phosphorylation
GO:0061146	2	10	0.0013323	Peyer's patch morphogenesis
GO:0021675	3	133	0.00135157	nerve development
GO:0001764	4	578	0.00143404	neuron migration
GO:0006936	4	640	0.00215127	muscle contraction
GO:0032989	5	1879	0.00355926	cellular component morphogenesis
GO:0032103	4	736	0.00374961	positive regulation of response to external stimulus
GO:0071363	5	1903	0.00379032	cellular response to growth factor stimulus
GO:2001239	3	190	0.00395219	regulation of extrinsic apoptotic signaling pathway in absence of ligand
GO:0070848	5	1987	0.00469471	response to growth factor
GO:0071300	3	202	0.00474994	cellular response to retinoic acid
GO:0003012	4	815	0.00562046	muscle system process
GO:0051046	5	2094	0.00608681	regulation of secretion
GO:0072073	3	223	0.00639062	kidney epithelium development
GO:0045596	5	2226	0.00823643	negative regulation of cell differentiation
GO:0001933	4	963	0.010889	negative regulation of protein phosphorylation
GO:0048858	4	963	0.010889	cell projection morphogenesis
GO:0030335	4	1024	0.0138849	positive regulation of cell migration
GO:0030030	5	2507	0.0148203	cell projection organization
GO:2000147	4	1042	0.0148756	positive regulation of cell motility
GO:0032990	4	1056	0.0156817	cell part morphogenesis
GO:0051272	4	1060	0.0159178	positive regulation of cellular component movement
GO:0051090	4	1091	0.0178388	regulation of sequence-specific DNA binding transcription factor activity
GO:0050921	3	324	0.0195487	positive regulation of chemotaxis
GO:0040017	4	1124	0.0200677	positive regulation of locomotion
GO:0051093	5	2690	0.0209817	negative regulation of developmental process
GO:0042326	4	1164	0.0230391	negative regulation of phosphorylation
GO:0001101	4	1170	0.0235115	response to acid chemical
GO:0008284	5	2808	0.0259272	positive regulation of cell proliferation
GO:0031667	4	1219	0.0276433	response to nutrient levels
GO:0021953	3	365	0.0279	central nervous system neuron differentiation
GO:0007411	4	1242	0.0297583	axon guidance
GO:0097485	4	1243	0.0298528	neuron projection guidance
GO:0048520	3	383	0.0322076	positive regulation of behavior
GO:0045944	5	2989	0.0352674	positive regulation of transcription from RNA polymerase II promoter
GO:0009991	4	1313	0.0370482	response to extracellular stimulus
GO:2001233	4	1313	0.0370482	regulation of apoptotic signaling pathway
GO:0044057	4	1316	0.0373828	regulation of system process
GO:0071495	5	3038	0.0382059	cellular response to endogenous stimulus
GO:0072182	2	53	0.0406575	regulation of nephron tubule epithelial cell differentiation
GO:0010563	4	1353	0.0416963	negative regulation of phosphorus metabolic process
GO:0045936	4	1353	0.0416963	negative regulation of phosphate metabolic process
GO:0031400	4	1368	0.0435462	negative regulation of protein modification process

Table 3: Overrepresented terms with the network-based enrichment. Only terms not detected with the standard method.
