# Supplementary material for: NET-GE: a novel NETwork-based Gene Enrichment for detecting biological processes associated to Mendelian diseases
Source: BMC Genomics. 2015 Jun 18;16(Suppl 8):S6. doi: 10.1186/1471-2164-16-S8-S6 (PMC4480278; doi:10.1186/1471-2164-16-S8-S6)
Supplement: Additional file 3 — Detailed results for the OMIM-derived benchmark set. The archive contains pdf documents listing the enriched terms for each one of the 244 diseases in the OMIM-derived benchmark set. [file 1471-2164-16-S8-S6-S3.tgz › SUPPMAT/OMIM209900.pdf]

#209900 BARDET-BIEDL SYNDROME; BBS

OMIM Gene ID	HGNC	UniProtAC
209901	BBS1	Q8NFJ9
600374	BBS4	Q96RK4
602290	TRIM32	Q13049
603650	BBS5	Q8N3I7
604896	MKKS	Q9NPJ1
606151	BBS2	Q9BXC9
606568	LZTFL1	Q9NQ48
607590	BBS7	Q8IWZ6
607968	BBS9	Q3SYG4
608132	TTC8	Q8TAM2
608845	ARL6	Q9H0F7
609883	MKS1	Q9NXB0
609884	TMEM67	Q5HYA8
610142	CEP290	O15078
610148	BBS10	Q8TAM1
610162	CCDC28B	Q9BUN5
610683	BBS12	Q6ZW61
613580	WDPCP	O95876
615870	IFT27	Q9BW83

Table 1: OMIM - UniProtAC mapping

Legend

- N1: #input proteins associated to the significant GO term
- N2: #proteins associated to the significant GO term
- P-value: Bonferroni-corrected p-value of Fisher's exact test
- *red*: go terms not related to the input proteins
- *blue*: go terms related to the input proteins (enriched uniquely by network-based method)
- *green*: go terms ancestors of terms enriched with the standard method (enriched uniquely by network-based method)

1 Standard enrichment

GO Term	N1	N2	P-value	Description
GO:0042384	15	206	1.38815e-28	cilium assembly
GO:0044782	15	210	1.87054e-28	cilium organization
GO:0010927	15	315	9.63965e-26	cellular component assembly involved in morphogenesis
GO:0030031	15	377	1.50081e-24	cell projection assembly
GO:0070925	15	476	5.20885e-23	organelle assembly
GO:0030030	15	1094	1.46428e-17	cell projection organization
GO:0048646	15	1201	5.9225e-17	anatomical structure formation involved in morphogenesis
GO:0060271	8	76	7.40885e-15	cilium morphogenesis
GO:0035058	7	36	1.04135e-14	nonmotile primary cilium assembly
GO:0022607	16	2496	5.52733e-14	cellular component assembly
GO:0007601	9	214	2.42796e-13	visual perception
GO:0050953	9	218	2.87467e-13	sensory perception of light stimulus
GO:0045444	8	167	4.85646e-12	fat cell differentiation
GO:0045494	6	37	8.40947e-12	photoreceptor cell maintenance
GO:0032402	6	40	1.38723e-11	melanosome transport
GO:0051904	6	41	1.62456e-11	pigment granule transport
GO:0032401	6	43	2.20137e-11	establishment of melanosome localization
GO:0051905	6	44	2.5482e-11	establishment of pigment granule localization
GO:0007600	10	586	3.31863e-11	sensory perception
GO:0050877	10	1063	1.19302e-08	neurological system process
GO:0048858	8	446	1.30072e-08	cell projection morphogenesis
GO:0032990	8	476	2.18117e-08	cell part morphogenesis
GO:0051877	3	3	5.83972e-08	pigment granule aggregation in cell center
GO:0051650	6	176	1.31482e-07	establishment of vesicle localization
GO:0016043	16	6578	2.18544e-07	cellular component organization
GO:0038108	3	4	2.33515e-07	negative regulation of appetite by leptin-mediated signaling pathway
GO:0071840	16	6631	2.47348e-07	cellular component organization or biogenesis
GO:0045184	11	2001	2.48912e-07	establishment of protein localization
GO:0044767	16	6740	3.18043e-07	single-organism developmental process
GO:0003008	10	1588	5.95636e-07	system process
GO:0032502	16	7299	1.08332e-06	developmental process
GO:0032989	8	788	1.16058e-06	cellular component morphogenesis
GO:0060295	3	7	2.04131e-06	regulation of cilium movement involved in cell motility
GO:0060296	3	7	2.04131e-06	regulation of cilium beat frequency involved in ciliary motility
GO:1902019	3	7	2.04131e-06	regulation of cilium-dependent cell motility
GO:0051656	6	314	4.22798e-06	establishment of organelle localization
GO:0033210	3	9	4.89603e-06	leptin-mediated signaling pathway
GO:0003352	3	10	6.99214e-06	regulation of cilium movement
GO:0048869	12	3694	1.06951e-05	cellular developmental process
GO:0032096	3	12	1.28107e-05	negative regulation of response to food
GO:0032099	3	12	1.28107e-05	negative regulation of appetite
GO:0021756	3	14	2.11825e-05	striatum development
GO:0044320	3	14	2.11825e-05	cellular response to leptin stimulus
GO:0001947	4	73	2.63621e-05	heart looping
GO:0051131	3	15	2.64697e-05	chaperone-mediated protein complex assembly
GO:0060632	3	16	3.25677e-05	regulation of microtubule-based movement
GO:0044321	3	17	3.95339e-05	response to leptin
GO:0003143	4	82	4.22416e-05	embryonic heart tube morphogenesis
GO:0032095	3	20	6.62143e-05	regulation of response to food
GO:0015031	9	1915	6.86383e-05	protein transport
GO:0044707	12	4361	6.92901e-05	single-multicellular organism process
GO:0032501	12	4447	8.61867e-05	multicellular organismal process
GO:0032098	3	23	0.000102766	regulation of appetite
GO:0032105	3	25	0.000133378	negative regulation of response to extracellular stimulus
GO:0032108	3	25	0.000133378	negative regulation of response to nutrient levels
GO:0048854	3	27	0.000169514	brain morphogenesis
GO:0009653	9	2131	0.000170506	anatomical structure morphogenesis
GO:0060562	4	120	0.000196005	epithelial tube morphogenesis
GO:0035239	4	141	0.000373946	tube morphogenesis
GO:0050893	2	3	0.000388821	sensory processing

Table 2: Overrepresented GO terms with the standard enrichment

GO Term	N1	N2	P-value	Description
GO:0060027	2	3	0.000388821	convergent extension involved in gastrulation
GO:0001895	3	40	0.000570218	retina homeostasis
GO:0051234	13	6974	0.00138062	establishment of localization
GO:0032886	4	206	0.0016917	regulation of microtubule-based process
GO:0032104	3	60	0.00196246	regulation of response to extracellular stimulus
GO:0032107	3	60	0.00196246	regulation of response to nutrient levels
GO:0002009	5	492	0.00199154	morphogenesis of an epithelium
GO:0040018	3	61	0.00206331	positive regulation of multicellular organism growth
GO:0071702	9	2983	0.00285168	organic substance transport
GO:0007608	3	68	0.00286677	sensory perception of smell
GO:0007368	3	69	0.00299613	determination of left/right symmetry
GO:0021766	3	69	0.00299613	hippocampus development
GO:0045927	4	244	0.00330482	positive regulation of growth
GO:0051179	13	7547	0.00348206	localization
GO:0009855	3	73	0.00355213	determination of bilateral symmetry
GO:0014824	2	8	0.00362355	artery smooth muscle contraction
GO:0009799	3	74	0.00370104	specification of symmetry
GO:0048729	5	578	0.00435202	tissue morphogenesis
GO:0014820	2	9	0.00465745	tonic smooth muscle contraction
GO:0021987	3	83	0.00523083	cerebral cortex development
GO:0030154	8	2446	0.00657169	cell differentiation
GO:0048598	5	638	0.00701039	embryonic morphogenesis
GO:0051649	8	2624	0.010989	establishment of localization in cell
GO:0007606	3	116	0.014279	sensory perception of chemical stimulus
GO:0014829	2	16	0.0154922	vascular smooth muscle contraction
GO:0040014	3	123	0.0170106	regulation of multicellular organism growth
GO:2000145	5	786	0.0190534	regulation of cell motility
GO:0060026	2	18	0.0197407	convergent extension
GO:0043623	4	393	0.0214094	cellular protein complex assembly
GO:0044763	17	16559	0.0261236	single-organism cellular process
GO:0051270	5	843	0.0265848	regulation of cellular component movement
GO:0040012	5	864	0.0298771	regulation of locomotion
GO:0043001	2	25	0.038626	Golgi to plasma membrane protein transport

Table 3: Overrepresented GO terms with the standard enrichment

2 Network-based enrichment

GO Term	N1	N2	P-value	Description
GO:0034622	8	1660	0.000989283	cellular macromolecular complex assembly
GO:0042073	3	62	0.00334192	intraciliary transport
GO:0042310	3	76	0.00618072	vasoconstriction
GO:0042311	3	78	0.00668371	vasodilation
GO:0044458	2	11	0.00969848	motile cilium assembly
GO:0010970	4	291	0.0114399	microtubule-based transport
GO:0030705	4	310	0.0146511	cytoskeleton-dependent intracellular transport
GO:0045776	3	114	0.0208539	negative regulation of blood pressure
GO:0033365	4	399	0.0391046	protein localization to organelle
GO:0006939	3	141	0.0392757	smooth muscle contraction
GO:0045599	3	147	0.044449	negative regulation of fat cell differentiation

Table 4: Overrepresented terms with the network-based enrichment. Only terms not detected with the standard method.
