# Supplementary material for: NET-GE: a novel NETwork-based Gene Enrichment for detecting biological processes associated to Mendelian diseases
Source: BMC Genomics. 2015 Jun 18;16(Suppl 8):S6. doi: 10.1186/1471-2164-16-S8-S6 (PMC4480278; doi:10.1186/1471-2164-16-S8-S6)
Supplement: Additional file 3 — Detailed results for the OMIM-derived benchmark set. The archive contains pdf documents listing the enriched terms for each one of the 244 diseases in the OMIM-derived benchmark set. [file 1471-2164-16-S8-S6-S3.tgz › SUPPMAT/OMIM209920.pdf]

#209920 BARE LYMPHOCYTE SYNDROME, TYPE II

**OMIM Gene ID**	**HGNC**	**UniProtAC**
600005	CIITA	P33076
601861	RFXAP	O00287
601863	RFX5	P48382
603200	RFXANK	O14593

Table 1: OMIM - UniProtAC mapping

Legend

- N1: #input proteins associated to the significant GO term
- N2: #proteins associated to the significant GO term
- P-value: Bonferroni-corrected p-value of Fisher's exact test
- *red*: go terms not related to the input proteins
- *blue*: go terms related to the input proteins (enriched uniquely by network-based method)
- *green*: go terms ancestors of terms enriched with the standard method (enriched uniquely by network-based method)

1 Standard enrichment

No enriched terms

2 Network-based enrichment

GO Term	N1	N2	P-value	Description
GO:0031664	2	49	0.00223576	regulation of lipopolysaccharide-mediated signaling pathway
GO:0002832	2	97	0.0088433	negative regulation of response to biotic stimulus
GO:0034121	2	107	0.0107689	regulation of toll-like receptor signaling pathway

Table 2: Overrepresented terms with the network-based enrichment. Only terms not detected with the standard method.
