# Supplementary material for: NET-GE: a novel NETwork-based Gene Enrichment for detecting biological processes associated to Mendelian diseases
Source: BMC Genomics. 2015 Jun 18;16(Suppl 8):S6. doi: 10.1186/1471-2164-16-S8-S6 (PMC4480278; doi:10.1186/1471-2164-16-S8-S6)
Supplement: Additional file 3 — Detailed results for the OMIM-derived benchmark set. The archive contains pdf documents listing the enriched terms for each one of the 244 diseases in the OMIM-derived benchmark set. [file 1471-2164-16-S8-S6-S3.tgz › SUPPMAT/OMIM210250.pdf]

# #210250 SITOSTEROLEMIA

| **OMIM Gene ID** | **HGNC** | **UniProtAC** |
|---|---|---|
| 605459 | ABCG5 | Q9H222 |
| 605460 | ABCG8 | Q9H221 |

Table 1: OMIM - UniProtAC mapping

Legend

- N1: #input proteins associated to the significant GO term
- N2: #proteins associated to the significant GO term
- P-value: Bonferroni-corrected p-value of Fisher's exact test
- *red*: go terms not related to the input proteins
- *blue*: go terms related to the input proteins (enriched uniquely by network-based method)
- *green*: go terms ancestors of terms enriched with the standard method (enriched uniquely by network-based method)

# 1 Standard enrichment

| GO Term | N1 | N2 | P-value | Description |
|---|---|---|---|---|
| GO:0010949 | 2 | 2 | 1.88137e-07 | negative regulation of intestinal phytosterol absorption |
| GO:0045796 | 2 | 2 | 1.88137e-07 | negative regulation of intestinal cholesterol absorption |
| GO:0030299 | 2 | 8 | 5.26782e-06 | intestinal cholesterol absorption |
| GO:0030300 | 2 | 9 | 6.77292e-06 | regulation of intestinal cholesterol absorption |
| GO:0032372 | 2 | 12 | 1.2417e-05 | negative regulation of sterol transport |
| GO:0032375 | 2 | 12 | 1.2417e-05 | negative regulation of cholesterol transport |
| GO:0060457 | 2 | 18 | 2.87849e-05 | negative regulation of digestive system process |
| GO:0050892 | 2 | 27 | 6.6036e-05 | intestinal absorption |
| GO:0032369 | 2 | 28 | 7.11157e-05 | negative regulation of lipid transport |
| GO:0033344 | 2 | 31 | 8.74835e-05 | cholesterol efflux |
| GO:0044058 | 2 | 38 | 0.00013226 | regulation of digestive system process |
| GO:0032371 | 2 | 48 | 0.000212218 | regulation of sterol transport |
| GO:0032374 | 2 | 48 | 0.000212218 | regulation of cholesterol transport |
| GO:0007588 | 2 | 51 | 0.000239875 | excretion |
| GO:0022600 | 2 | 65 | 0.000391324 | digestive system process |
| GO:0015918 | 2 | 66 | 0.000403553 | sterol transport |
| GO:0030301 | 2 | 66 | 0.000403553 | cholesterol transport |
| GO:0042632 | 2 | 95 | 0.00084003 | cholesterol homeostasis |
| GO:0055092 | 2 | 96 | 0.000857903 | sterol homeostasis |
| GO:0032368 | 2 | 112 | 0.00116946 | regulation of lipid transport |
| GO:0015850 | 2 | 139 | 0.00180442 | organic hydroxy compound transport |
| GO:0055088 | 2 | 140 | 0.00183057 | lipid homeostasis |
| GO:0007584 | 2 | 307 | 0.00883697 | response to nutrient |
| GO:0006869 | 2 | 348 | 0.0113593 | lipid transport |
| GO:0031667 | 2 | 493 | 0.0228168 | response to nutrient levels |
| GO:0051051 | 2 | 493 | 0.0228168 | negative regulation of transport |
| GO:0009991 | 2 | 532 | 0.0265735 | response to extracellular stimulus |
| GO:0051241 | 2 | 547 | 0.0280946 | negative regulation of multicellular organismal process |
| GO:0044057 | 2 | 554 | 0.028819 | regulation of system process |
| GO:0006200 | 2 | 569 | 0.0304022 | ATP catabolic process |
| GO:0009158 | 2 | 574 | 0.0309393 | ribonucleoside monophosphate catabolic process |
| GO:0009169 | 2 | 574 | 0.0309393 | purine ribonucleoside monophosphate catabolic process |
| GO:0009128 | 2 | 575 | 0.0310473 | purine nucleoside monophosphate catabolic process |
| GO:0009125 | 2 | 578 | 0.0313723 | nucleoside monophosphate catabolic process |
| GO:0042493 | 2 | 633 | 0.0376326 | response to drug |
| GO:0046034 | 2 | 656 | 0.0404192 | ATP metabolic process |
| GO:0009167 | 2 | 698 | 0.0457648 | purine ribonucleoside monophosphate metabolic process |
| GO:0009126 | 2 | 699 | 0.0458961 | purine nucleoside monophosphate metabolic process |
| GO:0009161 | 2 | 727 | 0.0496494 | ribonucleoside monophosphate metabolic process |

Table 2: Overrepresented GO terms with the standard enrichment

# 2 Network-based enrichment

*No novel enriched terms*
