# Supplementary material for: NET-GE: a novel NETwork-based Gene Enrichment for detecting biological processes associated to Mendelian diseases
Source: BMC Genomics. 2015 Jun 18;16(Suppl 8):S6. doi: 10.1186/1471-2164-16-S8-S6 (PMC4480278; doi:10.1186/1471-2164-16-S8-S6)
Supplement: Additional file 3 — Detailed results for the OMIM-derived benchmark set. The archive contains pdf documents listing the enriched terms for each one of the 244 diseases in the OMIM-derived benchmark set. [file 1471-2164-16-S8-S6-S3.tgz › SUPPMAT/OMIM211400.pdf]

# #211400 BRONCHIECTASIS WITH OR WITHOUT ELEVATED SWEAT CHLORIDE 1; BESC1

| OMIM Gene ID | HGNC | UniProtAC |
|---|---|---|
| 600760 | SCNN1B | P51168 |
| 602421 | CFTR | P13569 |

Table 1: OMIM - UniProtAC mapping

Legend

- N1: #input proteins associated to the significant GO term
- N2: #proteins associated to the significant GO term
- P-value: Bonferroni-corrected p-value of Fisher's exact test
- *red*: go terms not related to the input proteins
- *blue*: go terms related to the input proteins (enriched uniquely by network-based method)
- *green*: go terms ancestors of terms enriched with the standard method (enriched uniquely by network-based method)

# 1 Standard enrichment

| GO Term | N1 | N2 | P-value | Description |
|---|---|---|---|---|
| GO:0055067 | 2 | 104 | 0.00175213 | monovalent inorganic cation homeostasis |
| GO:1901529 | 1 | 1 | 0.0123467 | positive regulation of anion channel activity |
| GO:1902941 | 1 | 1 | 0.0123467 | regulation of voltage-gated chloride channel activity |
| GO:1902943 | 1 | 1 | 0.0123467 | positive regulation of voltage-gated chloride channel activity |
| GO:0010359 | 1 | 2 | 0.0246931 | regulation of anion channel activity |
| GO:2001225 | 1 | 4 | 0.0493846 | regulation of chloride transport |

Table 2: Overrepresented GO terms with the standard enrichment

# 2 Network-based enrichment

| GO Term | N1 | N2 | P-value | Description |
|---|---|---|---|---|
| GO:0030104 | 2 | 114 | 0.00746324 | water homeostasis |
| GO:0007588 | 2 | 120 | 0.00827316 | excretion |
| GO:0035725 | 2 | 244 | 0.0343511 | sodium ion transmembrane transport |

Table 3: Overrepresented terms with the network-based enrichment. Only terms not detected with the standard method.
