# Supplementary material for: NET-GE: a novel NETwork-based Gene Enrichment for detecting biological processes associated to Mendelian diseases
Source: BMC Genomics. 2015 Jun 18;16(Suppl 8):S6. doi: 10.1186/1471-2164-16-S8-S6 (PMC4480278; doi:10.1186/1471-2164-16-S8-S6)
Supplement: Additional file 3 — Detailed results for the OMIM-derived benchmark set. The archive contains pdf documents listing the enriched terms for each one of the 244 diseases in the OMIM-derived benchmark set. [file 1471-2164-16-S8-S6-S3.tgz › SUPPMAT/OMIM211900.pdf]

#211900 TUMORAL CALCINOSIS, HYPERPHOSPHATEMIC, FAMILIAL; HFTC

OMIM Gene ID	HGNC	UniProtAC
601756	GALNT3	Q14435
604824	KL	Q9UEF7
605380	FGF23	Q9GZV9

Table 1: OMIM - UniProtAC mapping

Legend

- N1: #input proteins associated to the significant GO term
- N2: #proteins associated to the significant GO term
- P-value: Bonferroni-corrected p-value of Fisher's exact test
- *red*: go terms not related to the input proteins
- *blue*: go terms related to the input proteins (enriched uniquely by network-based method)
- *green*: go terms ancestors of terms enriched with the standard method (enriched uniquely by network-based method)

1 Standard enrichment

GO Term	N1	N2	P-value	Description
GO:0090080	2	8	2.6415e-05	positive regulation of MAPKKK cascade by fibroblast growth factor receptor signaling pathway
GO:0030500	2	86	0.00344335	regulation of bone mineralization
GO:0070167	2	93	0.00402976	regulation of biomineral tissue development
GO:0048015	2	182	0.0154908	phosphatidylinositol-mediated signaling
GO:0048017	2	182	0.0154908	inositol lipid-mediated signaling
GO:0008286	2	195	0.0177853	insulin receptor signaling pathway
GO:0007173	2	202	0.0190861	epidermal growth factor receptor signaling pathway
GO:0038127	2	205	0.0196577	ERBB signaling pathway
GO:0008543	2	211	0.0208259	fibroblast growth factor receptor signaling pathway
GO:0044344	2	237	0.0262761	cellular response to fibroblast growth factor stimulus
GO:0071774	2	243	0.0276235	response to fibroblast growth factor
GO:0030278	2	261	0.0318662	regulation of ossification
GO:0032869	2	270	0.0341006	cellular response to insulin stimulus
GO:0048011	2	276	0.0356321	neurotrophin TRK receptor signaling pathway
GO:0038179	2	285	0.0379922	neurotrophin signaling pathway
GO:0038095	2	294	0.0404275	Fc-epsilon receptor signaling pathway

Table 2: Overrepresented GO terms with the standard enrichment

2 Network-based enrichment

GO Term	N1	N2	P-value	Description
GO:0043410	3	1269	0.0121311	positive regulation of MAPK cascade
GO:0007169	3	1794	0.0342991	transmembrane receptor protein tyrosine kinase signaling pathway
GO:0043408	3	1837	0.0368265	regulation of MAPK cascade
GO:0019725	3	1855	0.0379203	cellular homeostasis

Table 3: Overrepresented terms with the network-based enrichment. Only terms not detected with the standard method.
