# Supplementary material for: NET-GE: a novel NETwork-based Gene Enrichment for detecting biological processes associated to Mendelian diseases
Source: BMC Genomics. 2015 Jun 18;16(Suppl 8):S6. doi: 10.1186/1471-2164-16-S8-S6 (PMC4480278; doi:10.1186/1471-2164-16-S8-S6)
Supplement: Additional file 3 — Detailed results for the OMIM-derived benchmark set. The archive contains pdf documents listing the enriched terms for each one of the 244 diseases in the OMIM-derived benchmark set. [file 1471-2164-16-S8-S6-S3.tgz › SUPPMAT/OMIM211980.pdf]

#211980 LUNG CANCER

OMIM Gene ID	HGNC	UniProtAC
122720	CYP2A6	P11509
131550	EGFR	P00533
134638	FASLG	P48023
147575	IRF1	P10914
164757	BRAF	P15056
164870	ERBB2	P04626
171834	PIK3CA	P42336
190070	KRAS	P01116
191195	MAP3K8	P41279
601763	CASP8	Q14790
602544	PARK2	O60260
602631	SLC22A18	Q96BI1
603113	PPP2R1B	P30154
604050	DLEC1	Q9Y238
605082	RASSF1	Q9NS23
609413	ERCC6	Q03468

Table 1: OMIM - UniProtAC mapping

Legend

- N1: #input proteins associated to the significant GO term
- N2: #proteins associated to the significant GO term
- P-value: Bonferroni-corrected p-value of Fisher's exact test
- *red*: go terms not related to the input proteins
- *blue*: go terms related to the input proteins (enriched uniquely by network-based method)
- *green*: go terms ancestors of terms enriched with the standard method (enriched uniquely by network-based method)

1 Standard enrichment

GO Term	N1	N2	P-value	Description
GO:0051247	11	1526	1.89035e-09	positive regulation of protein metabolic process
GO:0032270	10	1363	2.74644e-08	positive regulation of cellular protein metabolic process
GO:0051246	12	2954	8.00086e-08	regulation of protein metabolic process
GO:0000186	5	83	2.23296e-07	activation of MAPKK activity
GO:0031401	9	1213	3.797e-07	positive regulation of protein modification process
GO:1902533	8	1008	3.06807e-06	positive regulation of intracellular signal transduction
GO:0009967	9	1548	3.23872e-06	positive regulation of signal transduction
GO:0045860	7	626	3.84201e-06	positive regulation of protein kinase activity
GO:0032268	10	2272	4.01885e-06	regulation of cellular protein metabolic process
GO:0048584	10	2308	4.6787e-06	positive regulation of response to stimulus
GO:0023056	9	1630	5.0871e-06	positive regulation of signaling
GO:0043549	8	1079	5.22647e-06	regulation of kinase activity
GO:0010647	9	1637	5.28122e-06	positive regulation of cell communication
GO:0033674	7	656	5.30618e-06	positive regulation of kinase activity
GO:0009966	11	3261	6.54132e-06	regulation of signal transduction
GO:0010604	11	3285	7.06932e-06	positive regulation of macromolecule metabolic process
GO:0048522	13	5768	9.94628e-06	positive regulation of cellular process
GO:0031325	11	3418	1.0756e-05	positive regulation of cellular metabolic process
GO:0048583	12	4515	1.10818e-05	regulation of response to stimulus
GO:0051338	8	1198	1.18259e-05	regulation of transferase activity
GO:0051347	7	751	1.34578e-05	positive regulation of transferase activity
GO:0031399	9	1823	1.34998e-05	regulation of protein modification process
GO:1902531	9	1847	1.51268e-05	regulation of intracellular signal transduction
GO:0009893	11	3630	2.02929e-05	positive regulation of metabolic process
GO:0008543	5	211	2.47705e-05	fibroblast growth factor receptor signaling pathway
GO:0023051	11	3708	2.53832e-05	regulation of signaling
GO:0010646	11	3714	2.58186e-05	regulation of cell communication
GO:0044344	5	237	4.42384e-05	cellular response to fibroblast growth factor stimulus
GO:0001932	8	1440	4.93643e-05	regulation of protein phosphorylation
GO:0071774	5	243	5.01085e-05	response to fibroblast growth factor
GO:0048518	13	6624	5.58921e-05	positive regulation of biological process
GO:0043085	9	2150	5.64541e-05	positive regulation of catalytic activity
GO:0001934	7	956	7.02119e-05	positive regulation of protein phosphorylation
GO:0048011	5	276	9.44262e-05	neurotrophin TRK receptor signaling pathway
GO:0045859	7	1005	9.86973e-05	regulation of protein kinase activity
GO:0038179	5	285	0.000110744	neurotrophin signaling pathway
GO:0043523	5	300	0.00014285	regulation of neuron apoptotic process
GO:0023014	5	309	0.000165402	signal transduction by phosphorylation
GO:0002376	9	2446	0.000171443	immune system process
GO:0050790	10	3371	0.000176459	regulation of catalytic activity
GO:0044093	9	2479	0.000192334	positive regulation of molecular function
GO:0042327	7	1129	0.000217528	positive regulation of phosphorylation
GO:0035556	9	2537	0.000234499	intracellular signal transduction
GO:0042325	8	1770	0.000242054	regulation of phosphorylation
GO:1901214	5	348	0.000297919	regulation of neuron death
GO:0032147	5	357	0.000337991	activation of protein kinase activity
GO:0010562	7	1255	0.000444918	positive regulation of phosphorus metabolic process
GO:0045937	7	1255	0.000444918	positive regulation of phosphate metabolic process
GO:0042981	8	1970	0.00054892	regulation of apoptotic process
GO:0043067	8	1982	0.000574938	regulation of programmed cell death
GO:0070848	6	793	0.000646669	response to growth factor
GO:0043408	6	794	0.000651442	regulation of MAPK cascade
GO:0065009	10	3941	0.000770734	regulation of molecular function
GO:0010941	8	2079	0.000827212	regulation of cell death
GO:0048523	11	5279	0.00100309	negative regulation of cellular process
GO:0007173	4	202	0.00157808	epidermal growth factor receptor signaling pathway
GO:0038127	4	205	0.00167339	ERBB signaling pathway
GO:0043524	4	216	0.00205984	negative regulation of neuron apoptotic process
GO:0048519	11	5756	0.00243262	negative regulation of biological process
GO:0043410	5	539	0.0025604	positive regulation of MAPK cascade

Table 2: Overrepresented GO terms with the standard enrichment

GO Term	N1	N2	P-value	Description
GO:1901215	4	244	0.00334098	negative regulation of neuron death
GO:0000165	4	249	0.00362056	MAPK cascade
GO:0043069	6	1068	0.00363913	negative regulation of programmed cell death
GO:0042221	10	4712	0.00407361	response to chemical
GO:0002682	7	1758	0.00424605	regulation of immune system process
GO:0010035	5	612	0.00475683	response to inorganic substance
GO:0002764	5	615	0.00487138	immune response-regulating signaling pathway
GO:0043406	4	275	0.00536323	positive regulation of MAP kinase activity
GO:0060548	6	1147	0.00548805	negative regulation of cell death
GO:1901700	7	1851	0.00597332	response to oxygen-containing compound
GO:0050776	6	1167	0.00606102	regulation of immune response
GO:0038095	4	294	0.00698227	Fc-epsilon receptor signaling pathway
GO:0071260	3	90	0.00821997	cellular response to mechanical stimulus
GO:0070887	8	2904	0.0102123	cellular response to chemical stimulus
GO:0042127	7	2008	0.0102165	regulation of cell proliferation
GO:0007611	4	329	0.0108758	learning or memory
GO:0019220	8	2977	0.0122775	regulation of phosphate metabolic process
GO:2001239	3	104	0.0126956	regulation of extrinsic apoptotic signaling pathway in absence of ligand
GO:0051174	8	2996	0.01287	regulation of phosphorus metabolic process
GO:0071363	5	762	0.0137639	cellular response to growth factor stimulus
GO:0038093	4	350	0.0138706	Fc receptor signaling pathway
GO:0006950	9	4134	0.014319	response to stress
GO:0071902	4	357	0.0149923	positive regulation of protein serine/threonine kinase activity
GO:0050890	4	365	0.0163546	cognition
GO:0007169	5	798	0.0171948	transmembrane receptor protein tyrosine kinase signaling pathway
GO:0006955	6	1414	0.0181486	immune response
GO:0043405	4	381	0.0193507	regulation of MAP kinase activity
GO:0050863	4	385	0.0201589	regulation of T cell activation
GO:0001775	5	825	0.0201812	cell activation
GO:0045087	5	825	0.0201812	innate immune response
GO:0050804	4	390	0.0212039	regulation of synaptic transmission
GO:0009628	6	1467	0.0223628	response to abiotic stimulus
GO:0010038	4	402	0.0238743	response to metal ion
GO:0060255	12	8942	0.0242445	regulation of macromolecule metabolic process
GO:0009416	4	424	0.0294026	response to light stimulus
GO:0006952	6	1580	0.0340207	defense response
GO:0003008	6	1588	0.0350036	system process
GO:0010033	8	3487	0.0392903	response to organic substance
GO:0007165	11	7592	0.0393412	signal transduction
GO:0071310	7	2482	0.040688	cellular response to organic substance
GO:0051716	12	9450	0.0441668	cellular response to stimulus
GO:0044763	15	16559	0.0461727	single-organism cellular process
GO:0007612	3	163	0.0486506	learning

Table 3: Overrepresented GO terms with the standard enrichment

2 Network-based enrichment

GO Term	N1	N2	P-value	Description
GO:0031647	8	386	8.89664e-09	regulation of protein stability
GO:0008285	12	2354	5.21e-08	negative regulation of cell proliferation
GO:0045786	9	824	8.06141e-08	negative regulation of cell cycle
GO:0030334	11	1926	1.94039e-07	regulation of cell migration
GO:0010608	10	1385	2.36096e-07	posttranscriptional regulation of gene expression
GO:1901698	12	2693	2.52357e-07	response to nitrogen compound
GO:2000145	11	2039	3.57946e-07	regulation of cell motility
GO:0050870	8	647	5.39632e-07	positive regulation of T cell activation
GO:0051270	11	2182	7.40196e-07	regulation of cellular component movement
GO:0018193	11	2224	9.07756e-07	peptidyl-amino acid modification
GO:0040012	11	2224	9.07756e-07	regulation of locomotion
GO:0046700	12	3029	9.9636e-07	heterocycle catabolic process
GO:0019439	12	3055	1.10072e-06	aromatic compound catabolic process
GO:1901699	10	1626	1.13255e-06	cellular response to nitrogen compound
GO:1901361	12	3169	1.68637e-06	organic cyclic compound catabolic process
GO:0042326	9	1164	1.71226e-06	negative regulation of phosphorylation
GO:0007265	7	457	2.2272e-06	Ras protein signal transduction
GO:0010243	11	2509	3.28881e-06	response to organonitrogen compound
GO:0051249	9	1264	3.53436e-06	regulation of lymphocyte activation
GO:0010959	8	835	4.02594e-06	regulation of metal ion transport
GO:1902532	9	1289	4.19741e-06	negative regulation of intracellular signal transduction
GO:2001233	9	1313	4.93483e-06	regulation of apoptotic signaling pathway
GO:0051251	8	874	5.76006e-06	positive regulation of lymphocyte activation
GO:0097191	6	280	6.18476e-06	extrinsic apoptotic signaling pathway
GO:0010563	9	1353	6.41984e-06	negative regulation of phosphorus metabolic process
GO:0045936	9	1353	6.41984e-06	negative regulation of phosphate metabolic process
GO:0042592	12	3571	6.74658e-06	homeostatic process
GO:1901652	9	1373	7.30039e-06	response to peptide
GO:0002521	8	929	9.29132e-06	leukocyte differentiation
GO:0002696	8	941	1.0273e-05	positive regulation of leukocyte activation
GO:0002694	9	1443	1.12798e-05	regulation of leukocyte activation
GO:2001236	7	581	1.17217e-05	regulation of extrinsic apoptotic signaling pathway
GO:0001933	8	963	1.23083e-05	negative regulation of protein phosphorylation
GO:1901615	9	1475	1.36628e-05	organic hydroxy compound metabolic process
GO:0071417	9	1481	1.41559e-05	cellular response to organonitrogen compound
GO:0034655	11	2890	1.47616e-05	nucleobase-containing compound catabolic process
GO:0071900	9	1492	1.51008e-05	regulation of protein serine/threonine kinase activity
GO:1901565	11	2901	1.53674e-05	organonitrogen compound catabolic process
GO:0050867	8	996	1.60159e-05	positive regulation of cell activation
GO:0030335	8	1024	1.98861e-05	positive regulation of cell migration
GO:0009314	9	1557	2.19061e-05	response to radiation
GO:2000147	8	1042	2.27817e-05	positive regulation of cell motility
GO:0050865	9	1568	2.32923e-05	regulation of cell activation
GO:0044270	11	3027	2.40925e-05	cellular nitrogen compound catabolic process
GO:0097190	8	1052	2.45432e-05	apoptotic signaling pathway
GO:0051272	8	1060	2.60364e-05	positive regulation of cellular component movement
GO:0046434	10	2260	2.75314e-05	organophosphate catabolic process
GO:0030336	7	660	2.81985e-05	negative regulation of cell migration
GO:0050730	7	663	2.90915e-05	regulation of peptidyl-tyrosine phosphorylation
GO:0008286	6	375	3.53049e-05	insulin receptor signaling pathway
GO:2000146	7	688	3.75135e-05	negative regulation of cell motility
GO:0040017	8	1124	4.10983e-05	positive regulation of locomotion
GO:0009968	11	3204	4.388e-05	negative regulation of signal transduction
GO:0050671	6	392	4.59419e-05	positive regulation of lymphocyte proliferation
GO:0051271	7	710	4.65579e-05	negative regulation of cellular component movement
GO:0071407	8	1148	4.84358e-05	cellular response to organic cyclic compound
GO:0032946	6	396	4.87958e-05	positive regulation of mononuclear cell proliferation
GO:0070665	6	403	5.41429e-05	positive regulation of leukocyte proliferation
GO:0031349	7	726	5.42446e-05	positive regulation of defense response
GO:0019637	12	4312	5.93182e-05	organophosphate metabolic process

Table 4: Overrepresented terms with the network-based enrichment. Only terms not detected with the standard method.

GO Term	N1	N2	P-value	Description
GO:0071496	7	736	5.9576e-05	cellular response to external stimulus
GO:1901136	10	2479	6.69384e-05	carbohydrate derivative catabolic process
GO:0060341	11	3341	6.81752e-05	regulation of cellular localization
GO:0043066	10	2487	6.90375e-05	negative regulation of apoptotic process
GO:0010648	11	3347	6.94732e-05	negative regulation of cell communication
GO:0023057	11	3347	6.94732e-05	negative regulation of signaling
GO:0006508	11	3357	7.1686e-05	proteolysis
GO:0043409	6	424	7.31724e-05	negative regulation of MAPK cascade
GO:0007167	10	2504	7.36939e-05	enzyme linked receptor protein signaling pathway
GO:0051050	10	2507	7.45439e-05	positive regulation of transport
GO:0032269	9	1804	7.87569e-05	negative regulation of cellular protein metabolic process
GO:0060627	8	1229	8.22273e-05	regulation of vesicle-mediated transport
GO:1901701	10	2540	8.44882e-05	cellular response to oxygen-containing compound
GO:0042129	6	439	8.99035e-05	regulation of T cell proliferation
GO:0010638	8	1260	9.97345e-05	positive regulation of organelle organization
GO:0043434	8	1303	0.000129313	response to peptide hormone
GO:0009203	9	1920	0.00013502	ribonucleoside triphosphate catabolic process
GO:0009207	9	1920	0.00013502	purine ribonucleoside triphosphate catabolic process
GO:0006954	8	1314	0.000137999	inflammatory response
GO:0009146	9	1925	0.000138087	purine nucleoside triphosphate catabolic process
GO:0032868	7	834	0.000140058	response to insulin
GO:0009143	9	1934	0.000143763	nucleoside triphosphate catabolic process
GO:0071214	7	838	0.000144706	cellular response to abiotic stimulus
GO:0040013	7	840	0.000147079	negative regulation of locomotion
GO:0006629	11	3598	0.000148337	lipid metabolic process
GO:0050821	5	232	0.00015968	protein stabilization
GO:2000113	11	3633	0.000164152	negative regulation of cellular macromolecule biosynthetic process
GO:0050877	10	2735	0.000171275	neurological system process
GO:0006152	9	1974	0.000171542	purine nucleoside catabolic process
GO:0046130	9	1974	0.000171542	purine ribonucleoside catabolic process
GO:0051726	10	2760	0.000186793	regulation of cell cycle
GO:0031400	8	1368	0.000188362	negative regulation of protein modification process
GO:0042454	9	2000	0.000192031	ribonucleoside catabolic process
GO:0045934	11	3691	0.000193719	negative regulation of nucleobase-containing compound metabolic process
GO:0022402	10	2771	0.000194008	cell cycle process
GO:0033157	7	878	0.000198861	regulation of intracellular protein transport
GO:0009154	9	2010	0.000200465	purine ribonucleotide catabolic process
GO:0050731	6	503	0.000200945	positive regulation of peptidyl-tyrosine phosphorylation
GO:0009261	9	2011	0.000201326	ribonucleotide catabolic process
GO:0051130	10	2782	0.00020147	positive regulation of cellular component organization
GO:0014070	10	2783	0.000202161	response to organic cyclic compound
GO:0034097	9	2017	0.000206561	response to cytokine
GO:0044255	10	2817	0.00022694	cellular lipid metabolic process
GO:0006195	9	2041	0.000228716	purine nucleotide catabolic process
GO:0009725	10	2824	0.000232364	response to hormone
GO:0009164	9	2045	0.000232604	nucleoside catabolic process
GO:0043065	8	1406	0.000232692	positive regulation of apoptotic process
GO:0048585	11	3759	0.000234418	negative regulation of response to stimulus
GO:0008219	10	2827	0.000234724	cell death
GO:0009636	6	517	0.000236257	response to toxic substance
GO:0022603	10	2832	0.000238704	regulation of anatomical structure morphogenesis
GO:0042742	6	519	0.000241695	defense response to bacterium
GO:0007283	8	1413	0.000241773	spermatogenesis
GO:0010558	11	3771	0.000242349	negative regulation of macromolecule biosynthetic process
GO:1901658	9	2055	0.00024258	glycosyl compound catabolic process
GO:0043068	8	1416	0.000245758	positive regulation of programmed cell death
GO:0048232	8	1420	0.000251159	male gamete generation
GO:0016265	10	2848	0.000251849	death
GO:0072523	9	2067	0.000255047	purine-containing compound catabolic process
GO:0051172	11	3798	0.000261081	negative regulation of nitrogen compound metabolic process

Table 5: Overrepresented terms with the network-based enrichment. Only terms not detected with the standard method.

GO Term	N1	N2	P-value	Description
GO:0043270	6	532	0.000279624	positive regulation of ion transport
GO:0032386	8	1443	0.000284251	regulation of intracellular transport
GO:0070372	6	536	0.000292235	regulation of ERK1 and ERK2 cascade
GO:0009166	9	2116	0.000311976	nucleotide catabolic process
GO:1901292	9	2130	0.000330164	nucleoside phosphate catabolic process
GO:0042493	8	1473	0.000333027	response to drug
GO:0061098	4	101	0.000349342	positive regulation of protein tyrosine kinase activity
GO:0051345	10	2953	0.000355254	positive regulation of hydrolase activity
GO:0045321	8	1486	0.000356314	leukocyte activation
GO:0009205	9	2149	0.000356334	purine ribonucleoside triphosphate metabolic process
GO:0042102	5	273	0.000358547	positive regulation of T cell proliferation
GO:0006915	9	2154	0.000363516	apoptotic process
GO:0009144	9	2159	0.000370825	purine nucleoside triphosphate metabolic process
GO:0002768	7	967	0.000383444	immune response-regulating cell surface receptor signaling pathway
GO:0009199	9	2170	0.000387359	ribonucleoside triphosphate metabolic process
GO:0051248	9	2189	0.000417446	negative regulation of protein metabolic process
GO:0001818	6	578	0.000455462	negative regulation of cytokine production
GO:0071495	10	3038	0.000464959	cellular response to endogenous stimulus
GO:0006468	10	3039	0.000466411	protein phosphorylation
GO:0010942	8	1542	0.000473439	positive regulation of cell death
GO:0009141	9	2225	0.000480111	nucleoside triphosphate metabolic process
GO:0070232	4	111	0.000510952	regulation of T cell apoptotic process
GO:0008610	8	1559	0.000515001	lipid biosynthetic process
GO:0080134	10	3072	0.000516646	regulation of response to stress
GO:0033135	5	294	0.000517723	regulation of peptidyl-serine phosphorylation
GO:0012501	9	2247	0.00052234	programmed cell death
GO:0007276	8	1582	0.00057622	gamete generation
GO:1900180	6	602	0.000578363	regulation of protein localization to nucleus
GO:0071356	5	301	0.000581698	cellular response to tumor necrosis factor
GO:0009617	6	605	0.000595482	response to bacterium
GO:0050670	6	605	0.000595482	regulation of lymphocyte proliferation
GO:0043269	8	1590	0.000598936	regulation of ion transport
GO:0051222	7	1034	0.000603856	positive regulation of protein transport
GO:0002684	9	2287	0.000607518	positive regulation of immune system process
GO:0032869	6	609	0.000618952	cellular response to insulin stimulus
GO:0032944	6	609	0.000618952	regulation of mononuclear cell proliferation
GO:0031347	8	1612	0.000665436	regulation of defense response
GO:0000122	9	2314	0.000671674	negative regulation of transcription from RNA polymerase II promoter
GO:1903322	5	310	0.000673001	positive regulation of protein modification by small protein conjugation or removal
GO:0009057	9	2318	0.000681667	macromolecule catabolic process
GO:0070663	6	621	0.00069399	regulation of leukocyte proliferation
GO:0032870	8	1636	0.000745156	cellular response to hormone stimulus
GO:0009117	10	3194	0.000746669	nucleotide metabolic process
GO:0043122	6	630	0.000755055	regulation of I-kappaB kinase/NF-kappaB signaling
GO:0071345	8	1640	0.000759213	cellular response to cytokine stimulus
GO:0051094	10	3200	0.00076002	positive regulation of developmental process
GO:0045785	6	632	0.000769209	positive regulation of cell adhesion
GO:0051223	8	1645	0.000777106	regulation of protein transport
GO:0006753	10	3223	0.00081318	nucleoside phosphate metabolic process
GO:0045892	10	3247	0.000872143	negative regulation of transcription, DNA-templated
GO:0016310	11	4277	0.00089709	phosphorylation
GO:0006584	4	128	0.000905684	catecholamine metabolic process
GO:0009712	4	128	0.000905684	catechol-containing compound metabolic process
GO:0046128	9	2404	0.000930447	purine ribonucleoside metabolic process
GO:0030098	6	654	0.000939865	lymphocyte differentiation
GO:0048878	9	2410	0.000950444	chemical homeostasis
GO:0042278	9	2412	0.000957195	purine nucleoside metabolic process
GO:0009611	6	657	0.000965377	response to wounding
GO:0009612	6	658	0.000974006	response to mechanical stimulus
GO:1901099	4	131	0.000993849	negative regulation of signal transduction in absence of ligand

Table 6: Overrepresented terms with the network-based enrichment. Only terms not detected with the standard method.

GO Term	N1	N2	P-value	Description
GO:2001240	4	131	0.000993849	negative regulation of extrinsic apoptotic signaling pathway in absence of ligand
GO:1902679	10	3295	0.00100156	negative regulation of RNA biosynthetic process
GO:0019221	7	1123	0.00105507	cytokine-mediated signaling pathway
GO:0006469	6	668	0.00106385	negative regulation of protein kinase activity
GO:0010976	5	341	0.0010778	positive regulation of neuron projection development
GO:0046822	6	675	0.00113072	regulation of nucleocytoplasmic transport
GO:0046632	4	137	0.00118919	alpha-beta T cell differentiation
GO:0051253	10	3369	0.00123453	negative regulation of RNA metabolic process
GO:0009119	9	2491	0.00125967	ribonucleoside metabolic process
GO:0055086	10	3391	0.00131248	nucleobase-containing small molecule metabolic process
GO:0021854	3	34	0.00133701	hypothalamus development
GO:0030162	8	1768	0.00134782	regulation of proteolysis
GO:0045597	9	2514	0.00136214	positive regulation of cell differentiation
GO:0001817	8	1774	0.00138307	regulation of cytokine production
GO:0043547	8	1783	0.00143744	positive regulation of GTPase activity
GO:0009150	9	2531	0.0014425	purine ribonucleotide metabolic process
GO:0033673	6	709	0.00150667	negative regulation of kinase activity
GO:0009116	9	2561	0.00159445	nucleoside metabolic process
GO:0046649	7	1196	0.00161265	lymphocyte activation
GO:0044087	8	1812	0.00162534	regulation of cellular component biogenesis
GO:0071396	7	1198	0.00163088	cellular response to lipid
GO:0009259	9	2583	0.00171461	ribonucleotide metabolic process
GO:0006163	9	2586	0.00173159	purine nucleotide metabolic process
GO:0019693	9	2596	0.00178927	ribose phosphate metabolic process
GO:0030155	7	1217	0.00181289	regulation of cell adhesion
GO:0033993	9	2604	0.00183661	response to lipid
GO:0034612	5	381	0.00186178	response to tumor necrosis factor
GO:1901657	9	2611	0.00187893	glycosyl compound metabolic process
GO:0048731	9	2612	0.00188505	system development
GO:0070201	8	1849	0.00189548	regulation of establishment of protein localization
GO:0006897	7	1228	0.00192589	endocytosis
GO:0051604	6	741	0.00194903	protein maturation
GO:0044723	8	1859	0.0019748	single-organism carbohydrate metabolic process
GO:0030217	5	386	0.00198512	T cell differentiation
GO:0010629	10	3561	0.0020774	negative regulation of gene expression
GO:0045088	6	751	0.00210732	regulation of innate immune response
GO:0046631	4	159	0.00215712	alpha-beta T cell activation
GO:0010952	5	393	0.00216856	positive regulation of peptidase activity
GO:0002460	4	160	0.00221179	adaptive immune response based on somatic recombination of immune receptors built from immunoglobulin
GO:0045766	5	397	0.00227925	positive regulation of angiogenesis
GO:0061097	4	162	0.00232422	regulation of protein tyrosine kinase activity
GO:0010662	3	41	0.0023769	regulation of striated muscle cell apoptotic process
GO:0051056	6	769	0.00241885	regulation of small GTPase mediated signal transduction
GO:0051093	9	2690	0.00241904	negative regulation of developmental process
GO:0043087	8	1921	0.00253335	regulation of GTPase activity
GO:0010975	7	1281	0.00255737	regulation of neuron projection development
GO:0033124	8	1931	0.00263509	regulation of GTP catabolic process
GO:0006974	8	1933	0.00265586	cellular response to DNA damage stimulus
GO:0072521	9	2722	0.0026737	purine-containing compound metabolic process
GO:0017157	5	411	0.00270233	regulation of exocytosis
GO:0044702	10	3664	0.00271285	single organism reproductive process
GO:0042110	6	787	0.00276726	T cell activation
GO:0006184	6	794	0.00291341	GTP catabolic process
GO:0007154	9	2752	0.00293341	cell communication
GO:0070228	4	172	0.00295137	regulation of lymphocyte apoptotic process
GO:0033043	9	2759	0.00299707	regulation of organelle organization
GO:0050771	4	174	0.00309053	negative regulation of axonogenesis
GO:1901069	6	807	0.00320166	guanosine-containing compound catabolic process
GO:0031345	5	428	0.00329705	negative regulation of cell projection organization
GO:0007270	4	178	0.00338341	neuron-neuron synaptic transmission

Table 7: Overrepresented terms with the network-based enrichment. Only terms not detected with the standard method.

GO Term	N1	N2	P-value	Description
GO:0042176	6	816	0.00341463	regulation of protein catabolic process
GO:0002028	4	181	0.00361626	regulation of sodium ion transport
GO:0051259	7	1363	0.00387411	protein oligomerization
GO:0009895	5	445	0.00399069	negative regulation of catabolic process
GO:0045742	3	49	0.00409846	positive regulation of epidermal growth factor receptor signaling pathway
GO:0051051	7	1375	0.00410777	negative regulation of transport
GO:0071375	6	846	0.00421013	cellular response to peptide hormone stimulus
GO:0032496	6	847	0.00423906	response to lipopolysaccharide
GO:0031396	5	451	0.00426127	regulation of protein ubiquitination
GO:0031295	4	189	0.00429543	T cell costimulation
GO:0080135	7	1388	0.00437418	regulation of cellular response to stress
GO:0031294	4	190	0.00438653	lymphocyte costimulation
GO:0042108	4	191	0.00447907	positive regulation of cytokine biosynthetic process
GO:0048167	5	456	0.00449763	regulation of synaptic plasticity
GO:0002252	7	1396	0.00454527	immune effector process
GO:0050768	5	457	0.00454613	negative regulation of neurogenesis
GO:0046039	6	862	0.00469291	GTP metabolic process
GO:0051046	8	2094	0.0048636	regulation of secretion
GO:0043393	5	465	0.0049491	regulation of protein binding
GO:0033121	8	2107	0.00509597	regulation of purine nucleotide catabolic process
GO:0048609	8	2109	0.00513257	multicellular organismal reproductive process
GO:0030811	8	2111	0.00516936	regulation of nucleotide catabolic process
GO:0009118	8	2125	0.00543341	regulation of nucleoside metabolic process
GO:0051348	6	887	0.00553742	negative regulation of transferase activity
GO:1901653	6	892	0.00572042	cellular response to peptide
GO:0060284	9	2979	0.00572148	regulation of cell development
GO:0031346	6	897	0.00590833	positive regulation of cell projection organization
GO:0045744	4	205	0.00593274	negative regulation of G-protein coupled receptor protein signaling pathway
GO:1901068	6	901	0.00606227	guanosine-containing compound metabolic process
GO:0046683	5	488	0.00626658	response to organophosphorus
GO:1901186	3	57	0.00649372	positive regulation of ERBB signaling pathway
GO:0051240	8	2185	0.00670044	positive regulation of multicellular organismal process
GO:0002237	6	918	0.00675368	response to molecule of bacterial origin
GO:0050778	7	1484	0.00683039	positive regulation of immune response
GO:0046640	3	58	0.00684592	regulation of alpha-beta T cell proliferation
GO:0032880	8	2200	0.0070541	regulation of protein localization
GO:0032409	5	500	0.00705586	regulation of transporter activity
GO:0008306	4	215	0.00716699	associative learning
GO:0034765	6	934	0.00746218	regulation of ion transmembrane transport
GO:0045596	8	2226	0.00770514	negative regulation of cell differentiation
GO:0051241	7	1513	0.00776814	negative regulation of multicellular organismal process
GO:0050770	5	511	0.00784631	regulation of axonogenesis
GO:0033138	4	220	0.00785098	positive regulation of peptidyl-serine phosphorylation
GO:0048545	7	1516	0.00787102	response to steroid hormone
GO:0007264	7	1517	0.00790558	small GTPase mediated signal transduction
GO:0002683	6	945	0.00798368	negative regulation of immune system process
GO:0001525	6	949	0.00818055	angiogenesis
GO:1903320	5	520	0.0085436	regulation of protein modification by small protein conjugation or removal
GO:0045639	4	226	0.00873436	positive regulation of myeloid cell differentiation
GO:0018108	5	523	0.00878656	peptidyl-tyrosine phosphorylation
GO:0001819	6	963	0.00890116	positive regulation of cytokine production
GO:0042306	5	527	0.00911894	regulation of protein import into nucleus
GO:0002757	6	972	0.00939125	immune response-activating signal transduction
GO:0098542	6	972	0.00939125	defense response to other organism
GO:0023052	8	2287	0.00943785	signaling
GO:0044700	8	2287	0.00943785	single organism signaling
GO:0018212	5	533	0.00963598	peptidyl-tyrosine modification
GO:0090316	5	535	0.00981337	positive regulation of intracellular protein transport
GO:0045862	4	233	0.00985582	positive regulation of proteolysis
GO:0006066	6	986	0.0101973	alcohol metabolic process

Table 8: Overrepresented terms with the network-based enrichment. Only terms not detected with the standard method.

GO Term	N1	N2	P-value	Description
GO:0045453	3	67	0.0106006	bone resorption
GO:0034762	6	998	0.0109325	regulation of transmembrane transport
GO:0006470	5	550	0.0112273	protein dephosphorylation
GO:0051495	5	556	0.0118358	positive regulation of cytoskeleton organization
GO:0022604	7	1615	0.0119722	regulation of cell morphogenesis
GO:0050678	6	1020	0.0123927	regulation of epithelial cell proliferation
GO:0043506	4	247	0.0124148	regulation of JUN kinase activity
GO:0051384	5	564	0.0126872	response to glucocorticoid
GO:0002250	4	249	0.0128169	adaptive immune response
GO:0051494	4	249	0.0128169	negative regulation of cytoskeleton organization
GO:2000106	4	251	0.0132286	regulation of leukocyte apoptotic process
GO:0070302	5	569	0.0132433	regulation of stress-activated protein kinase signaling cascade
GO:0031344	7	1640	0.0132524	regulation of cell projection organization
GO:0046879	4	253	0.01365	hormone secretion
GO:0048169	3	73	0.0137388	regulation of long-term neuronal synaptic plasticity
GO:0045930	3	74	0.0143151	negative regulation of mitotic cell cycle
GO:0016032	7	1665	0.0146455	viral process
GO:0044403	7	1665	0.0146455	symbiosis, encompassing mutualism through parasitism
GO:0051048	5	583	0.0149029	negative regulation of secretion
GO:0044764	7	1682	0.0156613	multi-organism cellular process
GO:0050868	4	264	0.016148	negative regulation of T cell activation
GO:0032320	6	1071	0.0163977	positive regulation of Ras GTPase activity
GO:0045637	5	596	0.0165864	regulation of myeloid cell differentiation
GO:1900542	8	2485	0.017551	regulation of purine nucleotide metabolic process
GO:0009894	9	3408	0.0176084	regulation of catabolic process
GO:0048638	5	605	0.0178369	regulation of developmental growth
GO:0010954	4	271	0.0179043	positive regulation of protein processing
GO:1903319	4	271	0.0179043	positive regulation of protein maturation
GO:0051090	6	1091	0.0182314	regulation of sequence-specific DNA binding transcription factor activity
GO:0006140	8	2500	0.0183553	regulation of nucleotide metabolic process
GO:0010721	5	609	0.0184159	negative regulation of cell development
GO:0031960	5	610	0.0185629	response to corticosteroid
GO:0006898	5	614	0.0191603	receptor-mediated endocytosis
GO:0031398	4	276	0.0192426	positive regulation of protein ubiquitination
GO:0005975	8	2526	0.0198241	carbohydrate metabolic process
GO:0008283	8	2526	0.0198241	cell proliferation
GO:0002253	6	1110	0.0201251	activation of immune response
GO:0030198	6	1111	0.0202291	extracellular matrix organization
GO:0097285	4	280	0.0203654	cell-type specific apoptotic process
GO:0010769	6	1115	0.0206493	regulation of cell morphogenesis involved in differentiation
GO:0043062	6	1116	0.0207554	extracellular structure organization
GO:0001959	4	285	0.0218362	regulation of cytokine-mediated signaling pathway
GO:0009653	10	4591	0.021887	anatomical structure morphogenesis
GO:0043367	3	86	0.0225205	CD4-positive, alpha-beta T cell differentiation
GO:0009914	4	288	0.0227555	hormone transport
GO:0051960	8	2584	0.0234663	regulation of nervous system development
GO:0035249	3	88	0.0241339	synaptic transmission, glutamatergic
GO:0050851	4	295	0.0250117	antigen receptor-mediated signaling pathway
GO:0022407	4	297	0.0256855	regulation of cell-cell adhesion
GO:0043491	3	90	0.0258218	protein kinase B signaling
GO:0016485	5	661	0.0273773	protein processing
GO:0035710	3	92	0.027586	CD4-positive, alpha-beta T cell activation
GO:0038034	3	92	0.027586	signal transduction in absence of ligand
GO:0097192	3	92	0.027586	extrinsic apoptotic signaling pathway in absence of ligand
GO:2000351	3	92	0.027586	regulation of endothelial cell apoptotic process
GO:0050878	7	1837	0.02797	regulation of body fluid levels
GO:0021700	5	666	0.0283923	developmental maturation
GO:2001234	5	666	0.0283923	negative regulation of apoptotic signaling pathway
GO:0030100	5	667	0.0285987	regulation of endocytosis
GO:0044265	7	1850	0.0292937	cellular macromolecule catabolic process

Table 9: Overrepresented terms with the network-based enrichment. Only terms not detected with the standard method.

GO Term	N1	N2	P-value	Description
GO:0060992	2	11	0.0294333	response to fungicide
GO:0055085	9	3627	0.0294914	transmembrane transport
GO:0007268	6	1195	0.0306689	synaptic transmission
GO:0051091	5	677	0.0307292	positive regulation of sequence-specific DNA binding transcription factor activity
GO:0048534	5	682	0.0318399	hematopoietic or lymphoid organ development
GO:0044711	9	3675	0.03287	single-organism biosynthetic process
GO:2001056	4	317	0.0331851	positive regulation of cysteine-type endopeptidase activity
GO:0065003	9	3681	0.0333151	macromolecular complex assembly
GO:0060759	4	318	0.0335982	regulation of response to cytokine stimulus
GO:0018958	4	319	0.034015	phenol-containing compound metabolic process
GO:0045664	7	1894	0.0341688	regulation of neuron differentiation
GO:0046824	4	322	0.0352883	positive regulation of nucleocytoplasmic transport
GO:0050854	3	100	0.0354374	regulation of antigen receptor-mediated signaling pathway
GO:0010906	4	323	0.0357204	regulation of glucose metabolic process
GO:0044419	7	1908	0.0358552	interspecies interaction between organisms
GO:0051129	7	1913	0.0364742	negative regulation of cellular component organization
GO:0045184	9	3728	0.0369853	establishment of protein localization
GO:0021954	3	102	0.037607	central nervous system neuron development
GO:0050708	5	710	0.038655	regulation of protein secretion
GO:0045765	5	715	0.0399835	regulation of angiogenesis
GO:0046907	9	3768	0.0403795	intracellular transport
GO:0042531	3	106	0.042205	positive regulation of tyrosine phosphorylation of STAT protein
GO:0048870	8	2808	0.0433959	cell motility
GO:0034121	3	107	0.0434098	regulation of toll-like receptor signaling pathway
GO:0007155	8	2817	0.044431	cell adhesion
GO:0048015	4	342	0.0446965	phosphatidylinositol-mediated signaling
GO:0048017	4	342	0.0446965	inositol lipid-mediated signaling
GO:0071901	4	342	0.0446965	negative regulation of protein serine/threonine kinase activity
GO:0022610	8	2824	0.0452505	biological adhesion
GO:0032102	5	737	0.04626	negative regulation of response to external stimulus
GO:0032318	6	1285	0.0463414	regulation of Ras GTPase activity
GO:0071248	4	346	0.04678	cellular response to metal ion
GO:0007610	7	1993	0.0476573	behavior
GO:0052547	6	1292	0.0477913	regulation of peptidase activity
GO:0032388	5	743	0.0480989	positive regulation of intracellular transport
GO:0009409	3	111	0.0484554	response to cold
GO:0010950	4	351	0.0494838	positive regulation of endopeptidase activity

Table 10: Overrepresented terms with the network-based enrichment. Only terms not detected with the standard method.
