# Supplementary material for: NET-GE: a novel NETwork-based Gene Enrichment for detecting biological processes associated to Mendelian diseases
Source: BMC Genomics. 2015 Jun 18;16(Suppl 8):S6. doi: 10.1186/1471-2164-16-S8-S6 (PMC4480278; doi:10.1186/1471-2164-16-S8-S6)
Supplement: Additional file 3 — Detailed results for the OMIM-derived benchmark set. The archive contains pdf documents listing the enriched terms for each one of the 244 diseases in the OMIM-derived benchmark set. [file 1471-2164-16-S8-S6-S3.tgz › SUPPMAT/OMIM212750.pdf]

# #212750 CELIAC DISEASE; CD

| OMIM Gene ID | HGNC | UniProtAC |
|---|---|---|
| 146880 | HLA-DQA1 | P01909 |
| 604305 | HLA-DQB1 | P01920 |

Table 1: OMIM - UniProtAC mapping

Legend

- N1: #input proteins associated to the significant GO term
- N2: #proteins associated to the significant GO term
- P-value: Bonferroni-corrected p-value of Fisher's exact test
- *red*: go terms not related to the input proteins
- *blue*: go terms related to the input proteins (enriched uniquely by network-based method)
- *green*: go terms ancestors of terms enriched with the standard method (enriched uniquely by network-based method)

# 1 Standard enrichment

| GO Term | N1 | N2 | P-value | Description |
|---|---|---|---|---|
| GO:0031295 | 2 | 93 | 0.000390411 | T cell costimulation |
| GO:0031294 | 2 | 94 | 0.000398899 | lymphocyte costimulation |
| GO:0019886 | 2 | 109 | 0.000537158 | antigen processing and presentation of exogenous peptide antigen via MHC class II |
| GO:0002495 | 2 | 111 | 0.000557144 | antigen processing and presentation of peptide antigen via MHC class II |
| GO:0002504 | 2 | 132 | 0.000789035 | antigen processing and presentation of peptide or polysaccharide antigen via MHC class II |
| GO:0050852 | 2 | 134 | 0.000813221 | T cell receptor signaling pathway |
| GO:0060333 | 2 | 146 | 0.000965991 | interferon-gamma-mediated signaling pathway |
| GO:0050851 | 2 | 171 | 0.00132647 | antigen receptor-mediated signaling pathway |
| GO:0071346 | 2 | 173 | 0.00135777 | cellular response to interferon-gamma |
| GO:0034341 | 2 | 201 | 0.00183433 | response to interferon-gamma |
| GO:0002478 | 2 | 254 | 0.00293229 | antigen processing and presentation of exogenous peptide antigen |
| GO:0019884 | 2 | 256 | 0.00297874 | antigen processing and presentation of exogenous antigen |
| GO:0050870 | 2 | 267 | 0.00324074 | positive regulation of T cell activation |
| GO:0048002 | 2 | 288 | 0.00377161 | antigen processing and presentation of peptide antigen |
| GO:0051251 | 2 | 345 | 0.00541539 | positive regulation of lymphocyte activation |
| GO:0002429 | 2 | 366 | 0.00609573 | immune response-activating cell surface receptor signaling pathway |
| GO:0002696 | 2 | 367 | 0.00612914 | positive regulation of leukocyte activation |
| GO:0019882 | 2 | 376 | 0.00643386 | antigen processing and presentation |
| GO:0050867 | 2 | 380 | 0.00657163 | positive regulation of cell activation |
| GO:0050863 | 2 | 385 | 0.00674596 | regulation of T cell activation |
| GO:0002768 | 2 | 486 | 0.0107555 | immune response-regulating cell surface receptor signaling pathway |
| GO:0002757 | 2 | 508 | 0.0117523 | immune response-activating signal transduction |
| GO:0051249 | 2 | 513 | 0.011985 | regulation of lymphocyte activation |
| GO:0019221 | 2 | 546 | 0.0135782 | cytokine-mediated signaling pathway |
| GO:0002253 | 2 | 566 | 0.014592 | activation of immune response |
| GO:0002694 | 2 | 584 | 0.0155358 | regulation of leukocyte activation |
| GO:0002764 | 2 | 615 | 0.0172304 | immune response-regulating signaling pathway |
| GO:0050865 | 2 | 624 | 0.0177388 | regulation of cell activation |
| GO:0071345 | 2 | 725 | 0.0239513 | cellular response to cytokine stimulus |
| GO:0050778 | 2 | 770 | 0.027019 | positive regulation of immune response |
| GO:0034097 | 2 | 880 | 0.0352958 | response to cytokine |
| GO:0002455 | 1 | 11 | 0.0378828 | humoral immune response mediated by circulating immunoglobulin |

Table 2: Overrepresented GO terms with the standard enrichment

# 2 Network-based enrichment

*No novel enriched terms*
