# Supplementary material for: NET-GE: a novel NETwork-based Gene Enrichment for detecting biological processes associated to Mendelian diseases
Source: BMC Genomics. 2015 Jun 18;16(Suppl 8):S6. doi: 10.1186/1471-2164-16-S8-S6 (PMC4480278; doi:10.1186/1471-2164-16-S8-S6)
Supplement: Additional file 3 — Detailed results for the OMIM-derived benchmark set. The archive contains pdf documents listing the enriched terms for each one of the 244 diseases in the OMIM-derived benchmark set. [file 1471-2164-16-S8-S6-S3.tgz › SUPPMAT/OMIM215150.pdf]

# #215150 OTOSPONDYLOMEGAEPIPHYSEAL DYSPLASIA; OSMED

| OMIM Gene ID | HGNC | UniProtAC |
|---|---|---|
| 120140 | COL2A1 | P02458 |
| 120290 | COL11A2 | P13942 |

Table 1: OMIM - UniProtAC mapping

Legend

- N1: #input proteins associated to the significant GO term
- N2: #proteins associated to the significant GO term
- P-value: Bonferroni-corrected p-value of Fisher's exact test
- *red*: go terms not related to the input proteins
- *blue*: go terms related to the input proteins (enriched uniquely by network-based method)
- *green*: go terms ancestors of terms enriched with the standard method (enriched uniquely by network-based method)

# 1 Standard enrichment

| GO Term | N1 | N2 | P-value | Description |
|---|---|---|---|---|
| GO:0030199 | 2 | 76 | 0.000500176 | collagen fibril organization |
| GO:0030574 | 2 | 78 | 0.000527029 | collagen catabolic process |
| GO:0044243 | 2 | 84 | 0.000611795 | multicellular organismal catabolic process |
| GO:0032963 | 2 | 96 | 0.000800282 | collagen metabolic process |
| GO:0044259 | 2 | 105 | 0.000958234 | multicellular organismal macromolecule metabolic process |
| GO:0044236 | 2 | 112 | 0.00109091 | multicellular organismal metabolic process |
| GO:0022617 | 2 | 117 | 0.00119095 | extracellular matrix disassembly |
| GO:0051216 | 2 | 118 | 0.00121148 | cartilage development |
| GO:0060021 | 2 | 120 | 0.00125307 | palate development |
| GO:0007605 | 2 | 175 | 0.002672 | sensory perception of sound |
| GO:0050954 | 2 | 180 | 0.00282731 | sensory perception of mechanical stimulus |
| GO:0001501 | 2 | 213 | 0.00396245 | skeletal system development |
| GO:0022411 | 2 | 404 | 0.0142868 | cellular component disassembly |
| GO:0030198 | 2 | 486 | 0.0206836 | extracellular matrix organization |
| GO:0043062 | 2 | 487 | 0.0207689 | extracellular structure organization |
| GO:0007600 | 2 | 586 | 0.0300816 | sensory perception |
| GO:0071599 | 1 | 5 | 0.033117 | otic vesicle development |
| GO:0060023 | 1 | 7 | 0.0463625 | soft palate development |

Table 2: Overrepresented GO terms with the standard enrichment

# 2 Network-based enrichment

| GO Term | N1 | N2 | P-value | Description |
|---|---|---|---|---|
| GO:0030500 | 2 | 210 | 0.0190215 | regulation of bone mineralization |
| GO:0070167 | 2 | 231 | 0.023026 | regulation of biomineral tissue development |

Table 3: Overrepresented terms with the network-based enrichment. Only terms not detected with the standard method.
