# Supplementary material for: NET-GE: a novel NETwork-based Gene Enrichment for detecting biological processes associated to Mendelian diseases
Source: BMC Genomics. 2015 Jun 18;16(Suppl 8):S6. doi: 10.1186/1471-2164-16-S8-S6 (PMC4480278; doi:10.1186/1471-2164-16-S8-S6)
Supplement: Additional file 3 — Detailed results for the OMIM-derived benchmark set. The archive contains pdf documents listing the enriched terms for each one of the 244 diseases in the OMIM-derived benchmark set. [file 1471-2164-16-S8-S6-S3.tgz › SUPPMAT/OMIM215600.pdf]

#215600 CIRRHOSIS, FAMILIAL

OMIM Gene ID	HGNC	UniProtAC
148060	KRT8	P05787
148070	KRT18	P05783

Table 1: OMIM - UniProtAC mapping

Legend

- N1: #input proteins associated to the significant GO term
- N2: #proteins associated to the significant GO term
- P-value: Bonferroni-corrected p-value of Fisher's exact test
- *red*: go terms not related to the input proteins
- *blue*: go terms related to the input proteins (enriched uniquely by network-based method)
- *green*: go terms ancestors of terms enriched with the standard method (enriched uniquely by network-based method)

1 Standard enrichment

GO Term	N1	N2	P-value	Description
GO:0097284	2	20	2.50755e-05	hepatocyte apoptotic process
GO:0033209	2	43	0.000119175	tumor necrosis factor-mediated signaling pathway
GO:0071356	2	120	0.000942312	cellular response to tumor necrosis factor
GO:0097191	2	132	0.00114107	extrinsic apoptotic signaling pathway
GO:0097285	2	135	0.00119372	cell-type specific apoptotic process
GO:0034612	2	149	0.00145518	response to tumor necrosis factor
GO:0043000	1	1	0.00498106	Golgi to plasma membrane CFTR protein transport
GO:0097190	2	439	0.0126883	apoptotic signaling pathway
GO:0019221	2	546	0.0196361	cytokine-mediated signaling pathway
GO:0016032	2	701	0.0323805	viral process
GO:0044403	2	701	0.0323805	symbiosis, encompassing mutualism through parasitism
GO:0044764	2	706	0.0328444	multi-organism cellular process
GO:0071345	2	725	0.0346372	cellular response to cytokine stimulus
GO:0044419	2	788	0.040923	interspecies interaction between organisms
GO:0006915	2	805	0.0427089	apoptotic process
GO:0012501	2	868	0.0496598	programmed cell death

Table 2: Overrepresented GO terms with the standard enrichment

2 Network-based enrichment

GO Term	N1	N2	P-value	Description
GO:0045104	2	71	0.00111504	intermediate filament cytoskeleton organization
GO:0045103	2	75	0.00124516	intermediate filament-based process

Table 3: Overrepresented terms with the network-based enrichment. Only terms not detected with the standard method.
