# Supplementary material for: NET-GE: a novel NETwork-based Gene Enrichment for detecting biological processes associated to Mendelian diseases
Source: BMC Genomics. 2015 Jun 18;16(Suppl 8):S6. doi: 10.1186/1471-2164-16-S8-S6 (PMC4480278; doi:10.1186/1471-2164-16-S8-S6)
Supplement: Additional file 3 — Detailed results for the OMIM-derived benchmark set. The archive contains pdf documents listing the enriched terms for each one of the 244 diseases in the OMIM-derived benchmark set. [file 1471-2164-16-S8-S6-S3.tgz › SUPPMAT/OMIM216360.pdf]

#216360 COACH SYNDROME

OMIM Gene ID	HGNC	UniProtAC
609884	TMEM67	Q5HYA8
610937	RPGRIP1L	Q68CZ1
612013	CC2D2A	Q9P2K1

Table 1: OMIM - UniProtAC mapping

Legend

- N1: #input proteins associated to the significant GO term
- N2: #proteins associated to the significant GO term
- P-value: Bonferroni-corrected p-value of Fisher's exact test
- *red*: go terms not related to the input proteins
- *blue*: go terms related to the input proteins (enriched uniquely by network-based method)
- *green*: go terms ancestors of terms enriched with the standard method (enriched uniquely by network-based method)

1 Standard enrichment

GO Term	N1	N2	P-value	Description
GO:0042384	3	206	1.85881e-05	cilium assembly
GO:0044782	3	210	1.96975e-05	cilium organization
GO:0010927	3	315	6.67987e-05	cellular component assembly involved in morphogenesis
GO:0030031	3	377	0.000114695	cell projection assembly
GO:0070925	3	476	0.000231239	organelle assembly
GO:0060271	2	76	0.00139068	cilium morphogenesis
GO:0030030	3	1094	0.00281736	cell projection organization
GO:0048646	3	1201	0.00372844	anatomical structure formation involved in morphogenesis
GO:0009653	3	2131	0.0208508	anatomical structure morphogenesis
GO:0022607	3	2496	0.0335116	cellular component assembly
GO:0048858	2	446	0.0481053	cell projection morphogenesis

Table 2: Overrepresented GO terms with the standard enrichment

2 Network-based enrichment

GO Term	N1	N2	P-value	Description
GO:0007163	2	427	0.0458258	establishment or maintenance of cell polarity

Table 3: Overrepresented terms with the network-based enrichment. Only terms not detected with the standard method.
