# Supplementary material for: NET-GE: a novel NETwork-based Gene Enrichment for detecting biological processes associated to Mendelian diseases
Source: BMC Genomics. 2015 Jun 18;16(Suppl 8):S6. doi: 10.1186/1471-2164-16-S8-S6 (PMC4480278; doi:10.1186/1471-2164-16-S8-S6)
Supplement: Additional file 3 — Detailed results for the OMIM-derived benchmark set. The archive contains pdf documents listing the enriched terms for each one of the 244 diseases in the OMIM-derived benchmark set. [file 1471-2164-16-S8-S6-S3.tgz › SUPPMAT/OMIM217095.pdf]

#217095 CONOTRUNCAL HEART MALFORMATIONS; CTHM

OMIM Gene ID	HGNC	UniProtAC
600584	NKX2-5	P52952
601656	GATA6	Q92908
602054	TBX1	O43435
602880	GDF1	P27539
605194	CFC1	P0CG37
611770	NKX2-6	A6NCS4

Table 1: OMIM - UniProtAC mapping

Legend

- N1: #input proteins associated to the significant GO term
- N2: #proteins associated to the significant GO term
- P-value: Bonferroni-corrected p-value of Fisher's exact test
- *red*: go terms not related to the input proteins
- *blue*: go terms related to the input proteins (enriched uniquely by network-based method)
- *green*: go terms ancestors of terms enriched with the standard method (enriched uniquely by network-based method)

1 Standard enrichment

GO Term	N1	N2	P-value	Description
GO:0003148	3	18	9.68102e-07	outflow tract septum morphogenesis
GO:0060037	3	25	2.72757e-06	pharyngeal system development
GO:0060411	3	61	4.2589e-05	cardiac septum morphogenesis
GO:0051891	2	5	0.000112016	positive regulation of cardioblast differentiation
GO:0055014	2	5	0.000112016	atrial cardiac muscle cell development
GO:0051890	2	9	0.000403143	regulation of cardioblast differentiation
GO:0035239	3	141	0.000538581	tube morphogenesis
GO:0042693	2	12	0.000738937	muscle cell fate commitment
GO:2000738	2	14	0.00101869	positive regulation of stem cell differentiation
GO:0055015	2	16	0.00134315	ventricular cardiac muscle cell development
GO:0016202	3	203	0.00161184	regulation of striated muscle tissue development
GO:1901861	3	204	0.0016358	regulation of muscle tissue development
GO:0048634	3	207	0.0017091	regulation of muscle organ development
GO:0035050	2	23	0.0028304	embryonic heart tube development
GO:0043066	4	1050	0.00454498	negative regulation of apoptotic process
GO:0043069	4	1068	0.00486142	negative regulation of programmed cell death
GO:0008284	4	1101	0.00548375	positive regulation of cell proliferation
GO:0030878	2	34	0.00627122	thyroid gland development
GO:0060548	4	1147	0.00644779	negative regulation of cell death
GO:0048732	3	391	0.0114704	gland development
GO:0003007	2	50	0.0136784	heart morphogenesis
GO:0055013	2	55	0.0165757	cardiac muscle cell development
GO:0060043	2	56	0.0171884	regulation of cardiac muscle cell proliferation
GO:0055006	2	58	0.018447	cardiac cell development
GO:0055007	2	59	0.0190929	cardiac muscle cell differentiation
GO:0055021	2	62	0.021097	regulation of cardiac muscle tissue growth
GO:0007368	2	69	0.0261603	determination of left/right symmetry
GO:0060420	2	70	0.0269278	regulation of heart growth
GO:0060415	2	71	0.0277064	muscle tissue morphogenesis
GO:0009855	2	73	0.0292966	determination of bilateral symmetry
GO:0009799	2	74	0.0301083	specification of symmetry
GO:0035051	2	76	0.0317647	cardiocyte differentiation
GO:0055024	2	77	0.0326095	regulation of cardiac muscle tissue development
GO:0007389	3	579	0.0369199	pattern specification process
GO:0010830	2	90	0.0445936	regulation of myotube differentiation
GO:0051146	2	93	0.0476232	striated muscle cell differentiation
GO:0048513	4	1910	0.0480511	organ development
GO:0048598	3	638	0.0492438	embryonic morphogenesis

Table 2: Overrepresented GO terms with the standard enrichment

2 Network-based enrichment

GO Term	N1	N2	P-value	Description
GO:0010621	2	6	0.00027157	negative regulation of transcription by transcription factor localization
GO:0003151	3	129	0.000753576	outflow tract morphogenesis
GO:0045165	3	501	0.0437978	cell fate commitment

Table 3: Overrepresented terms with the network-based enrichment. Only terms not detected with the standard method.
