# Supplementary material for: NET-GE: a novel NETwork-based Gene Enrichment for detecting biological processes associated to Mendelian diseases
Source: BMC Genomics. 2015 Jun 18;16(Suppl 8):S6. doi: 10.1186/1471-2164-16-S8-S6 (PMC4480278; doi:10.1186/1471-2164-16-S8-S6)
Supplement: Additional file 3 — Detailed results for the OMIM-derived benchmark set. The archive contains pdf documents listing the enriched terms for each one of the 244 diseases in the OMIM-derived benchmark set. [file 1471-2164-16-S8-S6-S3.tgz › SUPPMAT/OMIM219000.pdf]

#219000 FRASER SYNDROME

OMIM Gene ID	HGNC	UniProtAC
604597	GRIP1	Q9Y3R0
607830	FRAS1	Q86XX4
608945	FREM2	Q5SZK8

Table 1: OMIM - UniProtAC mapping

Legend

- N1: #input proteins associated to the significant GO term
- N2: #proteins associated to the significant GO term
- P-value: Bonferroni-corrected p-value of Fisher's exact test
- *red*: go terms not related to the input proteins
- *blue*: go terms related to the input proteins (enriched uniquely by network-based method)
- *green*: go terms ancestors of terms enriched with the standard method (enriched uniquely by network-based method)

1 Standard enrichment

GO Term	N1	N2	P-value	Description
GO:0007154	3	1103	0.00191673	cell communication
GO:0003338	1	5	0.0305985	metanephros morphogenesis
GO:0002009	2	492	0.0388349	morphogenesis of an epithelium

Table 2: Overrepresented GO terms with the standard enrichment

2 Network-based enrichment

GO Term	N1	N2	P-value	Description
GO:0060993	2	41	0.0013643	kidney morphogenesis
GO:0048729	3	1359	0.0208627	tissue morphogenesis

Table 3: Overrepresented terms with the network-based enrichment. Only terms not detected with the standard method.
