# Supplementary material for: NET-GE: a novel NETwork-based Gene Enrichment for detecting biological processes associated to Mendelian diseases
Source: BMC Genomics. 2015 Jun 18;16(Suppl 8):S6. doi: 10.1186/1471-2164-16-S8-S6 (PMC4480278; doi:10.1186/1471-2164-16-S8-S6)
Supplement: Additional file 3 — Detailed results for the OMIM-derived benchmark set. The archive contains pdf documents listing the enriched terms for each one of the 244 diseases in the OMIM-derived benchmark set. [file 1471-2164-16-S8-S6-S3.tgz › SUPPMAT/OMIM219050.pdf]

#219050 CRYPTORCHIDISM, UNILATERAL OR BILATERAL

OMIM Gene ID	HGNC	UniProtAC
146738	INSL3	P51460
606655	RXFP2	Q8WXD0

Table 1: OMIM - UniProtAC mapping

Legend

- N1: #input proteins associated to the significant GO term
- N2: #proteins associated to the significant GO term
- P-value: Bonferroni-corrected p-value of Fisher's exact test
- *red*: go terms not related to the input proteins
- *blue*: go terms related to the input proteins (enriched uniquely by network-based method)
- *green*: go terms ancestors of terms enriched with the standard method (enriched uniquely by network-based method)

1 Standard enrichment

GO Term	N1	N2	P-value	Description
GO:0001556	2	32	6.19784e-05	oocyte maturation
GO:0007193	2	71	0.000310517	adenylate cyclase-inhibiting G-protein coupled receptor signaling pathway
GO:0030819	2	83	0.000425227	positive regulation of cAMP biosynthetic process
GO:0030816	2	90	0.000500451	positive regulation of cAMP metabolic process
GO:0030804	2	94	0.000546185	positive regulation of cyclic nucleotide biosynthetic process
GO:0030810	2	96	0.000569801	positive regulation of nucleotide biosynthetic process
GO:1900373	2	96	0.000569801	positive regulation of purine nucleotide biosynthetic process
GO:0030801	2	103	0.000656396	positive regulation of cyclic nucleotide metabolic process
GO:0030817	2	117	0.000847955	regulation of cAMP biosynthetic process
GO:0045981	2	123	0.000937544	positive regulation of nucleotide metabolic process
GO:1900544	2	123	0.000937544	positive regulation of purine nucleotide metabolic process
GO:0008584	2	129	0.00103164	male gonad development
GO:0030814	2	133	0.00109687	regulation of cAMP metabolic process
GO:0030802	2	137	0.00116409	regulation of cyclic nucleotide biosynthetic process
GO:0030808	2	140	0.00121583	regulation of nucleotide biosynthetic process
GO:1900371	2	140	0.00121583	regulation of purine nucleotide biosynthetic process
GO:0030799	2	155	0.00149136	regulation of cyclic nucleotide metabolic process
GO:0007188	2	162	0.00162955	adenylate cyclase-modulating G-protein coupled receptor signaling pathway
GO:0008406	2	168	0.00175289	gonad development
GO:0048469	2	174	0.00188072	cell maturation
GO:0007187	2	193	0.00231519	G-protein coupled receptor signaling pathway, coupled to cyclic nucleotide second messenger
GO:0021700	2	238	0.00352415	developmental maturation
GO:0022412	2	398	0.00987197	cellular process involved in reproduction in multicellular organism
GO:0048608	2	406	0.0102733	reproductive structure development

Table 2: Overrepresented GO terms with the standard enrichment

2 Network-based enrichment

No novel enriched terms
