# Supplementary material for: NET-GE: a novel NETwork-based Gene Enrichment for detecting biological processes associated to Mendelian diseases
Source: BMC Genomics. 2015 Jun 18;16(Suppl 8):S6. doi: 10.1186/1471-2164-16-S8-S6 (PMC4480278; doi:10.1186/1471-2164-16-S8-S6)
Supplement: Additional file 3 — Detailed results for the OMIM-derived benchmark set. The archive contains pdf documents listing the enriched terms for each one of the 244 diseases in the OMIM-derived benchmark set. [file 1471-2164-16-S8-S6-S3.tgz › SUPPMAT/OMIM219700.pdf]

#219700 CYSTIC FIBROSIS; CF

OMIM Gene ID	HGNC	UniProtAC
146790	FCGR2A	P12318
190180	TGFB1	P01137
602421	CFTR	P13569

Table 1: OMIM - UniProtAC mapping

Legend

- N1: #input proteins associated to the significant GO term
- N2: #proteins associated to the significant GO term
- P-value: Bonferroni-corrected p-value of Fisher's exact test
- *red*: go terms not related to the input proteins
- *blue*: go terms related to the input proteins (enriched uniquely by network-based method)
- *green*: go terms ancestors of terms enriched with the standard method (enriched uniquely by network-based method)

1 Standard enrichment

No enriched terms

2 Network-based enrichment

**GO Term**	**N1**	**N2**	**P-value**	**Description**
GO:0042110	3	787	0.0263987	T cell activation
GO:1902105	3	834	0.0314231	regulation of leukocyte differentiation
GO:0014888	2	80	0.0342838	striated muscle adaptation
GO:0050999	2	81	0.0351511	regulation of nitric-oxide synthase activity
GO:0006833	2	90	0.0434427	water transport
GO:0043500	2	93	0.0464013	muscle adaptation

Table 2: Overrepresented terms with the network-based enrichment. Only terms not detected with the standard method.
