# Supplementary material for: NET-GE: a novel NETwork-based Gene Enrichment for detecting biological processes associated to Mendelian diseases
Source: BMC Genomics. 2015 Jun 18;16(Suppl 8):S6. doi: 10.1186/1471-2164-16-S8-S6 (PMC4480278; doi:10.1186/1471-2164-16-S8-S6)
Supplement: Additional file 3 — Detailed results for the OMIM-derived benchmark set. The archive contains pdf documents listing the enriched terms for each one of the 244 diseases in the OMIM-derived benchmark set. [file 1471-2164-16-S8-S6-S3.tgz › SUPPMAT/OMIM220100.pdf]

#220100 CYSTINURIA

**OMIM Gene ID**	**HGNC**	**UniProtAC**
104614	SLC3A1	Q07837
604144	SLC7A9	P82251

Table 1: OMIM - UniProtAC mapping

Legend

- N1: #input proteins associated to the significant GO term
- N2: #proteins associated to the significant GO term
- P-value: Bonferroni-corrected p-value of Fisher's exact test
- *red*: go terms not related to the input proteins
- *blue*: go terms related to the input proteins (enriched uniquely by network-based method)
- *green*: go terms ancestors of terms enriched with the standard method (enriched uniquely by network-based method)

1 Standard enrichment

GO Term	N1	N2	P-value	Description
GO:0015811	2	8	2.39804e-06	L-cystine transport
GO:0000101	2	13	6.68023e-06	sulfur amino acid transport
GO:0072337	2	32	4.24796e-05	modified amino acid transport
GO:0072348	2	79	0.00026387	sulfur compound transport
GO:0015807	2	88	0.000327846	L-amino acid transport
GO:0003333	2	109	0.000504102	amino acid transmembrane transport
GO:0006865	2	223	0.00211995	amino acid transport
GO:0098656	2	320	0.00437128	anion transmembrane transport
GO:0046942	2	378	0.00610244	carboxylic acid transport
GO:0015849	2	382	0.00623243	organic acid transport
GO:0015711	2	544	0.0126493	organic anion transport
GO:0071705	2	691	0.0204172	nitrogen compound transport
GO:0006820	2	806	0.0277843	anion transport
GO:0006520	2	839	0.0301075	cellular amino acid metabolic process

Table 2: Overrepresented GO terms with the standard enrichment

2 Network-based enrichment

GO Term	N1	N2	P-value	Description
GO:0015804	2	74	0.000160621	neutral amino acid transport
GO:0050900	2	635	0.0119705	leukocyte migration
GO:0015802	1	22	0.0435702	basic amino acid transport

Table 3: Overrepresented terms with the network-based enrichment. Only terms not detected with the standard method.
