# Supplementary material for: NET-GE: a novel NETwork-based Gene Enrichment for detecting biological processes associated to Mendelian diseases
Source: BMC Genomics. 2015 Jun 18;16(Suppl 8):S6. doi: 10.1186/1471-2164-16-S8-S6 (PMC4480278; doi:10.1186/1471-2164-16-S8-S6)
Supplement: Additional file 3 — Detailed results for the OMIM-derived benchmark set. The archive contains pdf documents listing the enriched terms for each one of the 244 diseases in the OMIM-derived benchmark set. [file 1471-2164-16-S8-S6-S3.tgz › SUPPMAT/OMIM220110.pdf]

#220110 MITOCHONDRIAL COMPLEX IV DEFICIENCY

**OMIM Gene ID**	**HGNC**	**UniProtAC**
124089	COX6B1	P14854
602125	COX10	Q12887
612322	FASTKD2	Q9NYY8
612958	TACO1	Q9BSH4
613920	COA5	Q86WW8
614478	COX14	Q96I36
614698	COX20	Q5RI15
614770	PET100	P0DJ07

Table 1: OMIM - UniProtAC mapping

Legend

- N1: #input proteins associated to the significant GO term
- N2: #proteins associated to the significant GO term
- P-value: Bonferroni-corrected p-value of Fisher's exact test
- *red*: go terms not related to the input proteins
- *blue*: go terms related to the input proteins (enriched uniquely by network-based method)
- *green*: go terms ancestors of terms enriched with the standard method (enriched uniquely by network-based method)

1 Standard enrichment

GO Term	N1	N2	P-value	Description
GO:0008535	2	15	0.000167944	respiratory chain complex IV assembly
GO:0017004	2	25	0.000479584	cytochrome complex assembly
GO:0045333	2	44	0.00151077	cellular respiration
GO:0006091	3	531	0.00309098	generation of precursor metabolites and energy
GO:0022904	2	131	0.0135359	respiratory electron transport chain
GO:0022900	2	151	0.0179837	electron transport chain
GO:0015980	2	231	0.0420054	energy derivation by oxidation of organic compounds
GO:1902600	2	232	0.0423684	hydrogen ion transmembrane transport
GO:0006123	1	3	0.0453017	mitochondrial electron transport, cytochrome c to oxygen
GO:0006784	1	3	0.0453017	heme a biosynthetic process
GO:0046160	1	3	0.0453017	heme a metabolic process
GO:0048033	1	3	0.0453017	heme o metabolic process
GO:0048034	1	3	0.0453017	heme O biosynthetic process

Table 2: Overrepresented GO terms with the standard enrichment

2 Network-based enrichment

GO Term	N1	N2	P-value	Description
GO:0097034	1	6	0.0414104	mitochondrial respiratory chain complex IV biogenesis

Table 3: Overrepresented terms with the network-based enrichment. Only terms not detected with the standard method.
