# Supplementary material for: NET-GE: a novel NETwork-based Gene Enrichment for detecting biological processes associated to Mendelian diseases
Source: BMC Genomics. 2015 Jun 18;16(Suppl 8):S6. doi: 10.1186/1471-2164-16-S8-S6 (PMC4480278; doi:10.1186/1471-2164-16-S8-S6)
Supplement: Additional file 3 — Detailed results for the OMIM-derived benchmark set. The archive contains pdf documents listing the enriched terms for each one of the 244 diseases in the OMIM-derived benchmark set. [file 1471-2164-16-S8-S6-S3.tgz › SUPPMAT/OMIM220290.pdf]

#220290 DEAFNESS, AUTOSOMAL RECESSIVE 1A; DFNB1A

**OMIM Gene ID**	**HGNC**	**UniProtAC**
121011	GJB2	P29033
603324	GJB3	O75712
604418	GJB6	O95452

Table 1: OMIM - UniProtAC mapping

Legend

- N1: #input proteins associated to the significant GO term
- N2: #proteins associated to the significant GO term
- P-value: Bonferroni-corrected p-value of Fisher's exact test
- *red*: go terms not related to the input proteins
- *blue*: go terms related to the input proteins (enriched uniquely by network-based method)
- *green*: go terms ancestors of terms enriched with the standard method (enriched uniquely by network-based method)

1 Standard enrichment

GO Term	N1	N2	P-value	Description
GO:0007605	3	175	6.17304e-06	sensory perception of sound
GO:0050954	3	180	6.72065e-06	sensory perception of mechanical stimulus
GO:0007600	3	586	0.000234603	sensory perception
GO:0050877	3	1063	0.00140358	neurological system process
GO:0007154	3	1103	0.00156823	cell communication
GO:0003008	3	1588	0.00468378	system process
GO:0048608	2	406	0.0216607	reproductive structure development

Table 2: Overrepresented GO terms with the standard enrichment

2 Network-based enrichment

GO Term	N1	N2	P-value	Description
GO:0003006	3	2335	0.0288869	developmental process involved in reproduction

Table 3: Overrepresented terms with the network-based enrichment. Only terms not detected with the standard method.
