# Supplementary material for: NET-GE: a novel NETwork-based Gene Enrichment for detecting biological processes associated to Mendelian diseases
Source: BMC Genomics. 2015 Jun 18;16(Suppl 8):S6. doi: 10.1186/1471-2164-16-S8-S6 (PMC4480278; doi:10.1186/1471-2164-16-S8-S6)
Supplement: Additional file 3 — Detailed results for the OMIM-derived benchmark set. The archive contains pdf documents listing the enriched terms for each one of the 244 diseases in the OMIM-derived benchmark set. [file 1471-2164-16-S8-S6-S3.tgz › SUPPMAT/OMIM221770.pdf]

#221770 POLYCYSTIC LIPOMEMBRANOUS OSTEODYSPLASIA WITH SCLEROSING LEUKOENCEPHALOPATHY;

**OMIM Gene ID**	**HGNC**	**UniProtAC**
604142	TYROBP	O43914
605086	TREM2	Q9NZC2

Table 1: OMIM - UniProtAC mapping

Legend

- N1: #input proteins associated to the significant GO term
- N2: #proteins associated to the significant GO term
- P-value: Bonferroni-corrected p-value of Fisher's exact test
- *red*: go terms not related to the input proteins
- *blue*: go terms related to the input proteins (enriched uniquely by network-based method)
- *green*: go terms ancestors of terms enriched with the standard method (enriched uniquely by network-based method)

1 Standard enrichment

GO Term	N1	N2	P-value	Description
GO:1903078	1	1	0.0063588	positive regulation of protein localization to plasma membrane
GO:1903080	1	1	0.0063588	regulation of C-C chemokine receptor CCR7 signaling pathway
GO:1903082	1	1	0.0063588	positive regulation of C-C chemokine receptor CCR7 signaling pathway
GO:2000350	1	1	0.0063588	positive regulation of CD40 signaling pathway
GO:0002582	1	2	0.0127175	positive regulation of antigen processing and presentation of peptide or polysaccharide antigen via MHC class II
GO:0002588	1	2	0.0127175	positive regulation of antigen processing and presentation of peptide antigen via MHC class II
GO:0007411	2	476	0.0190468	axon guidance
GO:0070101	1	3	0.0190759	positive regulation of chemokine-mediated signaling pathway
GO:2000348	1	3	0.0190759	regulation of CD40 signaling pathway
GO:0097485	2	477	0.0191269	neuron projection guidance
GO:0002586	1	4	0.0254341	regulation of antigen processing and presentation of peptide antigen via MHC class II
GO:0002580	1	5	0.0317923	regulation of antigen processing and presentation of peptide or polysaccharide antigen via MHC class II
GO:0002585	1	5	0.0317923	positive regulation of antigen processing and presentation of peptide antigen

Table 2: Overrepresented GO terms with the standard enrichment

2 Network-based enrichment

GO Term	N1	N2	P-value	Description
GO:0001959	2	285	0.0164089	regulation of cytokine-mediated signaling pathway
GO:0060759	2	318	0.0204363	regulation of response to cytokine stimulus

Table 3: Overrepresented terms with the network-based enrichment. Only terms not detected with the standard method.
