# Supplementary material for: NET-GE: a novel NETwork-based Gene Enrichment for detecting biological processes associated to Mendelian diseases
Source: BMC Genomics. 2015 Jun 18;16(Suppl 8):S6. doi: 10.1186/1471-2164-16-S8-S6 (PMC4480278; doi:10.1186/1471-2164-16-S8-S6)
Supplement: Additional file 3 — Detailed results for the OMIM-derived benchmark set. The archive contains pdf documents listing the enriched terms for each one of the 244 diseases in the OMIM-derived benchmark set. [file 1471-2164-16-S8-S6-S3.tgz › SUPPMAT/OMIM226600.pdf]

#226600 EPIDERMOLYSIS BULLOSA DYSTROPHICA, AUTOSOMAL RECESSIVE; RDEB

OMIM Gene ID	HGNC	UniProtAC
120120	COL7A1	Q02388
120353	MMP1	P03956

Table 1: OMIM - UniProtAC mapping

Legend

- N1: #input proteins associated to the significant GO term
- N2: #proteins associated to the significant GO term
- P-value: Bonferroni-corrected p-value of Fisher's exact test
- *red*: go terms not related to the input proteins
- *blue*: go terms related to the input proteins (enriched uniquely by network-based method)
- *green*: go terms ancestors of terms enriched with the standard method (enriched uniquely by network-based method)

1 Standard enrichment

GO Term	N1	N2	P-value	Description
GO:0030574	2	78	0.000307785	collagen catabolic process
GO:0044243	2	84	0.000357288	multicellular organismal catabolic process
GO:0032963	2	96	0.000467365	collagen metabolic process
GO:0044259	2	105	0.000559609	multicellular organismal macromolecule metabolic process
GO:0044236	2	112	0.000637093	multicellular organismal metabolic process
GO:0022617	2	117	0.000695513	extracellular matrix disassembly
GO:0022411	2	404	0.00834346	cellular component disassembly
GO:0030198	2	486	0.0120792	extracellular matrix organization
GO:0043062	2	487	0.012129	extracellular structure organization

Table 2: Overrepresented GO terms with the standard enrichment

2 Network-based enrichment

GO Term	N1	N2	P-value	Description
GO:0030199	2	136	0.00489664	collagen fibril organization
GO:0007160	2	403	0.0432071	cell-matrix adhesion

Table 3: Overrepresented terms with the network-based enrichment. Only terms not detected with the standard method.
