# Supplementary material for: NET-GE: a novel NETwork-based Gene Enrichment for detecting biological processes associated to Mendelian diseases
Source: BMC Genomics. 2015 Jun 18;16(Suppl 8):S6. doi: 10.1186/1471-2164-16-S8-S6 (PMC4480278; doi:10.1186/1471-2164-16-S8-S6)
Supplement: Additional file 3 — Detailed results for the OMIM-derived benchmark set. The archive contains pdf documents listing the enriched terms for each one of the 244 diseases in the OMIM-derived benchmark set. [file 1471-2164-16-S8-S6-S3.tgz › SUPPMAT/OMIM226650.pdf]

#226650 EPIDERMOLYSIS BULLOSA, JUNCTIONAL, NON-HERLITZ TYPE

OMIM Gene ID	HGNC	UniProtAC
113811	COL17A1	Q9UMD9
147557	ITGB4	P16144
150292	LAMC2	Q13753
150310	LAMB3	Q13751
600805	LAMA3	Q16787

Table 1: OMIM - UniProtAC mapping

Legend

- N1: #input proteins associated to the significant GO term
- N2: #proteins associated to the significant GO term
- P-value: Bonferroni-corrected p-value of Fisher's exact test
- *red*: go terms not related to the input proteins
- *blue*: go terms related to the input proteins (enriched uniquely by network-based method)
- *green*: go terms ancestors of terms enriched with the standard method (enriched uniquely by network-based method)

1 Standard enrichment

GO Term	N1	N2	P-value	Description
GO:0031581	5	14	2.66684e-16	hemidesmosome assembly
GO:0007044	5	63	9.36309e-13	cell-substrate junction assembly
GO:0034329	5	254	1.12803e-09	cell junction assembly
GO:0034330	5	300	2.60861e-09	cell junction organization
GO:0030198	5	486	2.94828e-08	extracellular matrix organization
GO:0043062	5	487	2.97887e-08	extracellular structure organization
GO:0022617	4	117	3.71807e-08	extracellular matrix disassembly
GO:0008544	4	151	1.04298e-07	epidermis development
GO:0060429	4	368	3.75004e-06	epithelium development
GO:0022411	4	404	5.45093e-06	cellular component disassembly
GO:0007155	5	1407	6.0776e-06	cell adhesion
GO:0022610	5	1410	6.14277e-06	biological adhesion
GO:0022607	5	2496	0.000107111	cellular component assembly
GO:0009888	4	984	0.000191128	tissue development
GO:0035987	2	26	0.000387364	endodermal cell differentiation
GO:0048856	5	4289	0.00160738	anatomical structure development
GO:0016043	5	6578	0.0136509	cellular component organization
GO:0071840	5	6631	0.01421	cellular component organization or biogenesis
GO:0032502	5	7299	0.0229654	developmental process
GO:0007160	2	228	0.0305147	cell-matrix adhesion
GO:0031589	2	292	0.0499282	cell-substrate adhesion

Table 2: Overrepresented GO terms with the standard enrichment

2 Network-based enrichment

GO Term	N1	N2	P-value	Description
GO:0009611	3	657	0.0193461	response to wounding
GO:0043588	2	99	0.0226832	skin development

Table 3: Overrepresented terms with the network-based enrichment. Only terms not detected with the standard method.
