# Supplementary material for: NET-GE: a novel NETwork-based Gene Enrichment for detecting biological processes associated to Mendelian diseases
Source: BMC Genomics. 2015 Jun 18;16(Suppl 8):S6. doi: 10.1186/1471-2164-16-S8-S6 (PMC4480278; doi:10.1186/1471-2164-16-S8-S6)
Supplement: Additional file 3 — Detailed results for the OMIM-derived benchmark set. The archive contains pdf documents listing the enriched terms for each one of the 244 diseases in the OMIM-derived benchmark set. [file 1471-2164-16-S8-S6-S3.tgz › SUPPMAT/OMIM226700.pdf]

#226700 EPIDERMOLYSIS BULLOSA, JUNCTIONAL, HERLITZ TYPE

OMIM Gene ID	HGNC	UniProtAC
150292	LAMC2	Q13753
150310	LAMB3	Q13751
600805	LAMA3	Q16787

Table 1: OMIM - UniProtAC mapping

Legend

- N1: #input proteins associated to the significant GO term
- N2: #proteins associated to the significant GO term
- P-value: Bonferroni-corrected p-value of Fisher's exact test
- *red*: go terms not related to the input proteins
- *blue*: go terms related to the input proteins (enriched uniquely by network-based method)
- *green*: go terms ancestors of terms enriched with the standard method (enriched uniquely by network-based method)

1 Standard enrichment

GO Term	N1	N2	P-value	Description
GO:0031581	3	14	1.66557e-09	hemidesmosome assembly
GO:0007044	3	63	1.81707e-07	cell-substrate junction assembly
GO:0022617	3	117	1.19028e-06	extracellular matrix disassembly
GO:0008544	3	151	2.57373e-06	epidermis development
GO:0034329	3	254	1.23499e-05	cell junction assembly
GO:0034330	3	300	2.03853e-05	cell junction organization
GO:0060429	3	368	3.76968e-05	epithelium development
GO:0022411	3	404	4.99138e-05	cellular component disassembly
GO:0035987	2	26	5.61011e-05	endodermal cell differentiation
GO:0030198	3	486	8.70028e-05	extracellular matrix organization
GO:0043062	3	487	8.7542e-05	extracellular structure organization
GO:0009888	3	984	0.000724384	tissue development
GO:0007155	3	1407	0.00211965	cell adhesion
GO:0022610	3	1410	0.00213325	biological adhesion
GO:0022607	3	2496	0.0118446	cellular component assembly

Table 2: Overrepresented GO terms with the standard enrichment

2 Network-based enrichment

No novel enriched terms
