# Supplementary material for: NET-GE: a novel NETwork-based Gene Enrichment for detecting biological processes associated to Mendelian diseases
Source: BMC Genomics. 2015 Jun 18;16(Suppl 8):S6. doi: 10.1186/1471-2164-16-S8-S6 (PMC4480278; doi:10.1186/1471-2164-16-S8-S6)
Supplement: Additional file 3 — Detailed results for the OMIM-derived benchmark set. The archive contains pdf documents listing the enriched terms for each one of the 244 diseases in the OMIM-derived benchmark set. [file 1471-2164-16-S8-S6-S3.tgz › SUPPMAT/OMIM226730.pdf]

#226730 EPIDERMOLYSIS BULLOSA JUNCTIONALIS WITH PYLORIC ATRESIA

**OMIM Gene ID**	**HGNC**	**UniProtAC**
147556	ITGA6	P23229
147557	ITGB4	P16144

Table 1: OMIM - UniProtAC mapping

Legend

- N1: #input proteins associated to the significant GO term
- N2: #proteins associated to the significant GO term
- P-value: Bonferroni-corrected p-value of Fisher's exact test
- *red*: go terms not related to the input proteins
- *blue*: go terms related to the input proteins (enriched uniquely by network-based method)
- *green*: go terms ancestors of terms enriched with the standard method (enriched uniquely by network-based method)

1 Standard enrichment

GO Term	N1	N2	P-value	Description
GO:0035878	2	6	3.05371e-06	nail development
GO:0097186	2	9	7.32891e-06	amelogenesis
GO:0031581	2	14	1.85258e-05	hemidesmosome assembly
GO:0072001	2	18	3.11479e-05	renal system development
GO:0043588	2	45	0.000201544	skin development
GO:0046847	2	49	0.000239411	filopodium assembly
GO:0007044	2	63	0.000397593	cell-substrate junction assembly
GO:0048565	2	72	0.000520353	digestive tract development
GO:0042475	2	132	0.00176016	odontogenesis of dentin-containing tooth
GO:0007229	2	166	0.00278803	integrin-mediated signaling pathway
GO:0042476	2	169	0.00289004	odontogenesis
GO:0007160	2	228	0.00526826	cell-matrix adhesion
GO:0035295	2	247	0.00618499	tube development
GO:0034329	2	254	0.00654125	cell junction assembly
GO:0031589	2	292	0.00864934	cell-substrate adhesion
GO:0034330	2	300	0.00913059	cell junction organization
GO:0030031	2	377	0.014429	cell projection assembly
GO:0030198	2	486	0.023993	extracellular matrix organization
GO:0043062	2	487	0.0240919	extracellular structure organization
GO:0009887	2	653	0.0433379	organ morphogenesis
GO:0010668	1	6	0.0460983	ectodermal cell differentiation

Table 2: Overrepresented GO terms with the standard enrichment

2 Network-based enrichment

GO Term	N1	N2	P-value	Description
GO:0045444	2	372	0.0417819	fat cell differentiation

Table 3: Overrepresented terms with the network-based enrichment. Only terms not detected with the standard method.
