# Supplementary material for: NET-GE: a novel NETwork-based Gene Enrichment for detecting biological processes associated to Mendelian diseases
Source: BMC Genomics. 2015 Jun 18;16(Suppl 8):S6. doi: 10.1186/1471-2164-16-S8-S6 (PMC4480278; doi:10.1186/1471-2164-16-S8-S6)
Supplement: Additional file 3 — Detailed results for the OMIM-derived benchmark set. The archive contains pdf documents listing the enriched terms for each one of the 244 diseases in the OMIM-derived benchmark set. [file 1471-2164-16-S8-S6-S3.tgz › SUPPMAT/OMIM231200.pdf]

#231200 BERNARD-SOULIER SYNDROME; BSS

**OMIM Gene ID**	**HGNC**	**UniProtAC**
138720	GP1BB	P13224
173515	GP9	P14770
606672	GP1BA	P07359

Table 1: OMIM - UniProtAC mapping

Legend

- N1: #input proteins associated to the significant GO term
- N2: #proteins associated to the significant GO term
- P-value: Bonferroni-corrected p-value of Fisher's exact test
- *red*: go terms not related to the input proteins
- *blue*: go terms related to the input proteins (enriched uniquely by network-based method)
- *green*: go terms ancestors of terms enriched with the standard method (enriched uniquely by network-based method)

1 Standard enrichment

GO Term	N1	N2	P-value	Description
GO:0007597	3	18	5.00875e-09	blood coagulation, intrinsic pathway
GO:0072376	3	215	1.00258e-05	protein activation cascade
GO:0030168	3	216	1.0167e-05	platelet activation
GO:0007596	3	501	0.000127878	blood coagulation
GO:0050817	3	501	0.000127878	coagulation
GO:0007599	3	510	0.000134908	hemostasis
GO:0050878	3	717	0.000375514	regulation of body fluid levels
GO:0001775	3	825	0.000572357	cell activation
GO:0007155	3	1407	0.00284344	cell adhesion
GO:0022610	3	1410	0.00286168	biological adhesion

Table 2: Overrepresented GO terms with the standard enrichment

2 Network-based enrichment

No novel enriched terms
