# Supplementary material for: NET-GE: a novel NETwork-based Gene Enrichment for detecting biological processes associated to Mendelian diseases
Source: BMC Genomics. 2015 Jun 18;16(Suppl 8):S6. doi: 10.1186/1471-2164-16-S8-S6 (PMC4480278; doi:10.1186/1471-2164-16-S8-S6)
Supplement: Additional file 3 — Detailed results for the OMIM-derived benchmark set. The archive contains pdf documents listing the enriched terms for each one of the 244 diseases in the OMIM-derived benchmark set. [file 1471-2164-16-S8-S6-S3.tgz › SUPPMAT/OMIM231680.pdf]

#231680 MULTIPLE ACYL-CoA DEHYDROGENASE DEFICIENCY; MADD

**OMIM Gene ID**	**HGNC**	**UniProtAC**
130410	ETFB	P38117
231675	ETFDH	Q16134
608053	ETFA	P13804

Table 1: OMIM - UniProtAC mapping

Legend

- N1: #input proteins associated to the significant GO term
- N2: #proteins associated to the significant GO term
- P-value: Bonferroni-corrected p-value of Fisher's exact test
- *red*: go terms not related to the input proteins
- *blue*: go terms related to the input proteins (enriched uniquely by network-based method)
- *green*: go terms ancestors of terms enriched with the standard method (enriched uniquely by network-based method)

1 Standard enrichment

GO Term	N1	N2	P-value	Description
GO:0022904	3	131	1.63452e-06	respiratory electron transport chain
GO:0022900	3	151	2.51096e-06	electron transport chain
GO:0006091	3	531	0.000110767	generation of precursor metabolites and energy
GO:0055114	3	2084	0.0067244	oxidation-reduction process
GO:0033539	1	7	0.0222522	fatty acid beta-oxidation using acyl-CoA dehydrogenase

Table 2: Overrepresented GO terms with the standard enrichment

2 Network-based enrichment

GO Term	N1	N2	P-value	Description
GO:0006635	2	108	0.00271181	fatty acid beta-oxidation
GO:0019395	2	161	0.00603859	fatty acid oxidation
GO:0034440	2	166	0.00642004	lipid oxidation
GO:0009062	2	168	0.00657589	fatty acid catabolic process
GO:0072329	2	213	0.0105743	monocarboxylic acid catabolic process
GO:0044242	2	388	0.0350387	cellular lipid catabolic process
GO:0030258	2	443	0.0456405	lipid modification

Table 3: Overrepresented terms with the network-based enrichment. Only terms not detected with the standard method.
