# Supplementary material for: NET-GE: a novel NETwork-based Gene Enrichment for detecting biological processes associated to Mendelian diseases
Source: BMC Genomics. 2015 Jun 18;16(Suppl 8):S6. doi: 10.1186/1471-2164-16-S8-S6 (PMC4480278; doi:10.1186/1471-2164-16-S8-S6)
Supplement: Additional file 3 — Detailed results for the OMIM-derived benchmark set. The archive contains pdf documents listing the enriched terms for each one of the 244 diseases in the OMIM-derived benchmark set. [file 1471-2164-16-S8-S6-S3.tgz › SUPPMAT/OMIM233650-OMIM601457.pdf]

# #233650 COMBINED CELLULAR AND HUMORAL IMMUNE DEFECTS WITH GRANULOMAS; CCHIDG
# #601457 SEVERE COMBINED IMMUNODEFICIENCY, AUTOSOMAL RECESSIVE, T CELL-NEGATIVE, B CELL-NEGATIVE, NK CELL-POSITIVE

| **OMIM Gene ID** | **HGNC** | **UniProtAC** |
|---|---|---|
| 179615 | RAG1 | P15918 |
| 179616 | RAG2 | P55895 |

Table 1: OMIM - UniProtAC mapping

Legend

- N1: #input proteins associated to the significant GO term
- N2: #proteins associated to the significant GO term
- P-value: Bonferroni-corrected p-value of Fisher's exact test
- *red*: go terms not related to the input proteins
- *blue*: go terms related to the input proteins (enriched uniquely by network-based method)
- *green*: go terms ancestors of terms enriched with the standard method (enriched uniquely by network-based method)

# 1 Standard enrichment

| GO Term | N1 | N2 | P-value | Description |
|---|---|---|---|---|
| GO:0002331 | 2 | 8 | 4.95332e-06 | pre-B cell allelic exclusion |
| GO:0033151 | 2 | 30 | 7.69535e-05 | V(D)J recombination |
| GO:0033077 | 2 | 60 | 0.000313121 | T cell differentiation in thymus |
| GO:0002562 | 2 | 63 | 0.000345495 | somatic diversification of immune receptors via germline recombination within a single locus |
| GO:0016444 | 2 | 63 | 0.000345495 | somatic cell DNA recombination |
| GO:0002200 | 2 | 66 | 0.00037946 | somatic diversification of immune receptors |
| GO:0030183 | 2 | 113 | 0.00111945 | B cell differentiation |
| GO:0030217 | 2 | 154 | 0.00208412 | T cell differentiation |
| GO:0042113 | 2 | 186 | 0.00304364 | B cell activation |
| GO:0030098 | 2 | 263 | 0.0060949 | lymphocyte differentiation |
| GO:0006310 | 2 | 294 | 0.00761946 | DNA recombination |
| GO:0042110 | 2 | 319 | 0.00897278 | T cell activation |
| GO:0002521 | 2 | 375 | 0.0124054 | leukocyte differentiation |
| GO:0046649 | 2 | 484 | 0.0206777 | lymphocyte activation |
| GO:0045321 | 2 | 579 | 0.0296017 | leukocyte activation |
| GO:0016568 | 2 | 721 | 0.0459174 | chromatin modification |

Table 2: Overrepresented GO terms with the standard enrichment

# 2 Network-based enrichment

*No novel enriched terms*
