# Supplementary material for: NET-GE: a novel NETwork-based Gene Enrichment for detecting biological processes associated to Mendelian diseases
Source: BMC Genomics. 2015 Jun 18;16(Suppl 8):S6. doi: 10.1186/1471-2164-16-S8-S6 (PMC4480278; doi:10.1186/1471-2164-16-S8-S6)
Supplement: Additional file 3 — Detailed results for the OMIM-derived benchmark set. The archive contains pdf documents listing the enriched terms for each one of the 244 diseases in the OMIM-derived benchmark set. [file 1471-2164-16-S8-S6-S3.tgz › SUPPMAT/OMIM235200.pdf]

#235200 HEMOCHROMATOSIS, TYPE 1; HFE1

**OMIM Gene ID**	**HGNC**	**UniProtAC**
112261	BMP2	P12643
613609	HFE	Q30201

Table 1: OMIM - UniProtAC mapping

Legend

- N1: #input proteins associated to the significant GO term
- N2: #proteins associated to the significant GO term
- P-value: Bonferroni-corrected p-value of Fisher's exact test
- *red*: go terms not related to the input proteins
- *blue*: go terms related to the input proteins (enriched uniquely by network-based method)
- *green*: go terms ancestors of terms enriched with the standard method (enriched uniquely by network-based method)

1 Standard enrichment

GO Term	N1	N2	P-value	Description
GO:0010106	1	1	0.0246933	cellular response to iron ion starvation
GO:0051042	1	1	0.0246933	negative regulation of calcium-independent cell-cell adhesion
GO:0060804	1	1	0.0246933	positive regulation of Wnt signaling pathway by BMP signaling pathway
GO:0051040	1	2	0.0493862	regulation of calcium-independent cell-cell adhesion
GO:0060128	1	2	0.0493862	corticotropin hormone secreting cell differentiation

Table 2: Overrepresented GO terms with the standard enrichment

2 Network-based enrichment

GO Term	N1	N2	P-value	Description
GO:0042446	2	141	0.0203651	hormone biosynthetic process

Table 3: Overrepresented terms with the network-based enrichment. Only terms not detected with the standard method.
