# Supplementary material for: NET-GE: a novel NETwork-based Gene Enrichment for detecting biological processes associated to Mendelian diseases
Source: BMC Genomics. 2015 Jun 18;16(Suppl 8):S6. doi: 10.1186/1471-2164-16-S8-S6 (PMC4480278; doi:10.1186/1471-2164-16-S8-S6)
Supplement: Additional file 3 — Detailed results for the OMIM-derived benchmark set. The archive contains pdf documents listing the enriched terms for each one of the 244 diseases in the OMIM-derived benchmark set. [file 1471-2164-16-S8-S6-S3.tgz › SUPPMAT/OMIM235400.pdf]

#235400 HEMOLYTIC UREMIC SYNDROME, ATYPICAL, SUSCEPTIBILITY TO, 1; AHUS1

OMIM Gene ID	HGNC	UniProtAC
134370	CFH	P08603
134371	CFHR1	Q03591
605336	CFHR3	Q02985

Table 1: OMIM - UniProtAC mapping

Legend

- N1: #input proteins associated to the significant GO term
- N2: #proteins associated to the significant GO term
- P-value: Bonferroni-corrected p-value of Fisher's exact test
- *red*: go terms not related to the input proteins
- *blue*: go terms related to the input proteins (enriched uniquely by network-based method)
- *green*: go terms ancestors of terms enriched with the standard method (enriched uniquely by network-based method)

1 Standard enrichment

GO Term	N1	N2	P-value	Description
GO:0006956	2	180	0.00104045	complement activation
GO:0072376	2	215	0.00148576	protein activation cascade
GO:0006959	2	294	0.00278171	humoral immune response
GO:0002253	2	566	0.0103267	activation of immune response
GO:0002252	2	657	0.0139176	immune effector process
GO:0050778	2	770	0.0191211	positive regulation of immune response
GO:0006957	1	13	0.031683	complement activation, alternative pathway
GO:0002684	2	1093	0.0385424	positive regulation of immune system process
GO:0050776	2	1167	0.0439406	regulation of immune response

Table 2: Overrepresented GO terms with the standard enrichment

2 Network-based enrichment

No novel enriched terms
