# Supplementary material for: NET-GE: a novel NETwork-based Gene Enrichment for detecting biological processes associated to Mendelian diseases
Source: BMC Genomics. 2015 Jun 18;16(Suppl 8):S6. doi: 10.1186/1471-2164-16-S8-S6 (PMC4480278; doi:10.1186/1471-2164-16-S8-S6)
Supplement: Additional file 3 — Detailed results for the OMIM-derived benchmark set. The archive contains pdf documents listing the enriched terms for each one of the 244 diseases in the OMIM-derived benchmark set. [file 1471-2164-16-S8-S6-S3.tgz › SUPPMAT/OMIM237450.pdf]

# #237450 HYPERBILIRUBINEMIA, ROTOR TYPE; HBLRR

| OMIM Gene ID | HGNC | UniProtAC |
|---|---|---|
| 604843 | SLCO1B1 | Q9Y6L6 |
| 605495 | SLCO1B3 | Q9NPD5 |

Table 1: OMIM - UniProtAC mapping

Legend

- N1: #input proteins associated to the significant GO term
- N2: #proteins associated to the significant GO term
- P-value: Bonferroni-corrected p-value of Fisher's exact test
- *red*: go terms not related to the input proteins
- *blue*: go terms related to the input proteins (enriched uniquely by network-based method)
- *green*: go terms ancestors of terms enriched with the standard method (enriched uniquely by network-based method)

# 1 Standard enrichment

| GO Term | N1 | N2 | P-value | Description |
|---|---|---|---|---|
| GO:0043252 | 2 | 13 | 3.50438e-06 | sodium-independent organic anion transport |
| GO:0015721 | 2 | 30 | 1.95437e-05 | bile acid and bile salt transport |
| GO:0008206 | 2 | 44 | 4.25021e-05 | bile acid metabolic process |
| GO:0015718 | 2 | 158 | 0.000557245 | monocarboxylic acid transport |
| GO:0008202 | 2 | 325 | 0.00236547 | steroid metabolic process |
| GO:0046942 | 2 | 378 | 0.00320128 | carboxylic acid transport |
| GO:0015849 | 2 | 382 | 0.00326947 | organic acid transport |
| GO:0015711 | 2 | 544 | 0.00663571 | organic anion transport |
| GO:0032787 | 2 | 767 | 0.0131981 | monocarboxylic acid metabolic process |
| GO:0006820 | 2 | 806 | 0.0145754 | anion transport |

Table 2: Overrepresented GO terms with the standard enrichment

# 2 Network-based enrichment

*No novel enriched terms*
