# Supplementary material for: NET-GE: a novel NETwork-based Gene Enrichment for detecting biological processes associated to Mendelian diseases
Source: BMC Genomics. 2015 Jun 18;16(Suppl 8):S6. doi: 10.1186/1471-2164-16-S8-S6 (PMC4480278; doi:10.1186/1471-2164-16-S8-S6)
Supplement: Additional file 3 — Detailed results for the OMIM-derived benchmark set. The archive contains pdf documents listing the enriched terms for each one of the 244 diseases in the OMIM-derived benchmark set. [file 1471-2164-16-S8-S6-S3.tgz › SUPPMAT/OMIM248200.pdf]

#248200 STARGARDT DISEASE 1; STGD1

OMIM Gene ID	HGNC	UniProtAC
601691	ABCA4	P78363
605080	CNGB3	Q9NQW8

Table 1: OMIM - UniProtAC mapping

Legend

- N1: #input proteins associated to the significant GO term
- N2: #proteins associated to the significant GO term
- P-value: Bonferroni-corrected p-value of Fisher's exact test
- *red*: go terms not related to the input proteins
- *blue*: go terms related to the input proteins (enriched uniquely by network-based method)
- *green*: go terms ancestors of terms enriched with the standard method (enriched uniquely by network-based method)

1 Standard enrichment

GO Term	N1	N2	P-value	Description
GO:0007603	2	89	0.000758735	phototransduction, visible light
GO:0009584	2	106	0.00107823	detection of visible light
GO:0007602	2	112	0.00120437	phototransduction
GO:0009583	2	129	0.00159963	detection of light stimulus
GO:0007601	2	214	0.00441582	visual perception
GO:0050953	2	218	0.00458283	sensory perception of light stimulus
GO:0009581	2	221	0.00471013	detection of external stimulus
GO:0009582	2	224	0.00483916	detection of abiotic stimulus
GO:0006649	1	2	0.0146251	phospholipid transfer to membrane
GO:0009416	2	424	0.017375	response to light stimulus
GO:0007600	2	586	0.0332101	sensory perception
GO:0009314	2	591	0.0337798	response to radiation

Table 2: Overrepresented GO terms with the standard enrichment

2 Network-based enrichment

GO Term	N1	N2	P-value	Description
GO:0071804	2	351	0.0130614	cellular potassium ion transport
GO:0071805	2	351	0.0130614	potassium ion transmembrane transport
GO:0006813	2	419	0.0186211	potassium ion transport

Table 3: Overrepresented terms with the network-based enrichment. Only terms not detected with the standard method.
