# Supplementary material for: NET-GE: a novel NETwork-based Gene Enrichment for detecting biological processes associated to Mendelian diseases
Source: BMC Genomics. 2015 Jun 18;16(Suppl 8):S6. doi: 10.1186/1471-2164-16-S8-S6 (PMC4480278; doi:10.1186/1471-2164-16-S8-S6)
Supplement: Additional file 3 — Detailed results for the OMIM-derived benchmark set. The archive contains pdf documents listing the enriched terms for each one of the 244 diseases in the OMIM-derived benchmark set. [file 1471-2164-16-S8-S6-S3.tgz › SUPPMAT/OMIM248600.pdf]

# #248600 MAPLE SYRUP URINE DISEASE; MSUD

| OMIM Gene ID | HGNC | UniProtAC |
|---|---|---|
| 248610 | DBT | P11182 |
| 248611 | BCKDHB | P21953 |
| 608348 | BCKDHA | P12694 |

Table 1: OMIM - UniProtAC mapping

Legend

- N1: #input proteins associated to the significant GO term
- N2: #proteins associated to the significant GO term
- P-value: Bonferroni-corrected p-value of Fisher's exact test
- *red*: go terms not related to the input proteins
- *blue*: go terms related to the input proteins (enriched uniquely by network-based method)
- *green*: go terms ancestors of terms enriched with the standard method (enriched uniquely by network-based method)

# 1 Standard enrichment

| GO Term | N1 | N2 | P-value | Description |
|---|---|---|---|---|
| GO:0009083 | 3 | 21 | 7.27317e-09 | branched-chain amino acid catabolic process |
| GO:0009081 | 3 | 34 | 3.27238e-08 | branched-chain amino acid metabolic process |
| GO:0009063 | 3 | 173 | 4.63759e-06 | cellular amino acid catabolic process |
| GO:0016054 | 3 | 278 | 1.93711e-05 | organic acid catabolic process |
| GO:0046395 | 3 | 278 | 1.93711e-05 | carboxylic acid catabolic process |
| GO:0044282 | 3 | 358 | 4.14689e-05 | small molecule catabolic process |
| GO:0006520 | 3 | 839 | 0.000536354 | cellular amino acid metabolic process |
| GO:0051591 | 2 | 153 | 0.00239349 | response to cAMP |
| GO:1901565 | 3 | 1431 | 0.0026652 | organonitrogen compound catabolic process |
| GO:0046683 | 2 | 188 | 0.00361598 | response to organophosphorus |
| GO:0019752 | 3 | 1590 | 0.00365673 | carboxylic acid metabolic process |
| GO:0043436 | 3 | 1732 | 0.00472729 | oxoacid metabolic process |
| GO:0051384 | 2 | 215 | 0.00473011 | response to glucocorticoid |
| GO:0006082 | 3 | 1753 | 0.00490142 | organic acid metabolic process |
| GO:0031960 | 2 | 226 | 0.00522668 | response to corticosteroid |
| GO:0014074 | 2 | 233 | 0.00555552 | response to purine-containing compound |
| GO:0044712 | 3 | 2063 | 0.00799072 | single-organism catabolic process |
| GO:0007584 | 2 | 307 | 0.00964207 | response to nutrient |
| GO:0044248 | 3 | 2821 | 0.0204394 | cellular catabolic process |
| GO:0031667 | 2 | 493 | 0.0248133 | response to nutrient levels |
| GO:1901575 | 3 | 3074 | 0.026449 | organic substance catabolic process |
| GO:1901564 | 3 | 3152 | 0.0285146 | organonitrogen compound metabolic process |
| GO:0009991 | 2 | 532 | 0.0288787 | response to extracellular stimulus |
| GO:0009056 | 3 | 3347 | 0.0341428 | catabolic process |
| GO:0048545 | 2 | 646 | 0.0425089 | response to steroid hormone |

Table 2: Overrepresented GO terms with the standard enrichment

# 2 Network-based enrichment

*No novel enriched terms*
