# Supplementary material for: NET-GE: a novel NETwork-based Gene Enrichment for detecting biological processes associated to Mendelian diseases
Source: BMC Genomics. 2015 Jun 18;16(Suppl 8):S6. doi: 10.1186/1471-2164-16-S8-S6 (PMC4480278; doi:10.1186/1471-2164-16-S8-S6)
Supplement: Additional file 3 — Detailed results for the OMIM-derived benchmark set. The archive contains pdf documents listing the enriched terms for each one of the 244 diseases in the OMIM-derived benchmark set. [file 1471-2164-16-S8-S6-S3.tgz › SUPPMAT/OMIM252010.pdf]

#252010 MITOCHONDRIAL COMPLEX I DEFICIENCY

OMIM Gene ID	HGNC	UniProtAC
157655	NDUFS1	P28331
161015	NDUFV1	P49821
300078	NDUFA1	O15239
600532	NDUFV2	P19404
601445	NDUFB9	Q9Y6M9
602694	NDUFS4	O43181
602985	NDUFS2	O75306
603839	NDUFB3	O43676
603846	NDUFS3	O75489
603848	NDUFS6	O75380
606934	NDUFAF1	Q9Y375
609653	NDUFAF2	Q8N183
611776	NDUFAF4	Q9P032
612360	NDUFAF5	Q5TEU4
612638	NDUFA11	Q86Y39
612911	NDUFAF3	Q9BU61
613621	NUBPL	Q8TB37
613622	FOXRED1	Q96CU9

Table 1: OMIM - UniProtAC mapping

Legend

- N1: #input proteins associated to the significant GO term
- N2: #proteins associated to the significant GO term
- P-value: Bonferroni-corrected p-value of Fisher's exact test
- *red*: go terms not related to the input proteins
- *blue*: go terms related to the input proteins (enriched uniquely by network-based method)
- *green*: go terms ancestors of terms enriched with the standard method (enriched uniquely by network-based method)

1 Standard enrichment

GO Term	N1	N2	P-value	Description
GO:0022904	13	131	9.46066e-27	respiratory electron transport chain
GO:0006120	11	49	3.24338e-26	mitochondrial electron transport, NADH to ubiquinone
GO:0022900	13	151	6.50809e-26	electron transport chain
GO:0006091	13	531	1.14553e-18	generation of precursor metabolites and energy
GO:0055114	14	2084	1.13598e-12	oxidation-reduction process
GO:0010257	5	16	1.14009e-11	NADH dehydrogenase complex assembly
GO:0032981	5	16	1.14009e-11	mitochondrial respiratory chain complex I assembly
GO:0097031	5	16	1.14009e-11	mitochondrial respiratory chain complex I biogenesis
GO:0070271	5	26	1.712e-10	protein complex biogenesis
GO:0033108	5	28	2.55637e-10	mitochondrial respiratory chain complex assembly
GO:0044085	5	118	4.43481e-07	cellular component biogenesis
GO:0072593	4	110	3.94854e-05	reactive oxygen species metabolic process
GO:0044281	11	4403	0.000152026	small molecule metabolic process
GO:0043623	5	393	0.000178321	cellular protein complex assembly
GO:0044710	14	8611	0.000235897	single-organism metabolic process
GO:0034622	5	788	0.00522126	cellular macromolecular complex assembly
GO:0006461	6	1365	0.00550896	protein complex assembly
GO:0065003	6	1623	0.0145079	macromolecular complex assembly
GO:0070584	2	29	0.0168774	mitochondrion morphogenesis
GO:0071822	6	1933	0.0380092	protein complex subunit organization
GO:0045333	2	44	0.0391589	cellular respiration

Table 2: Overrepresented GO terms with the standard enrichment

2 Network-based enrichment

GO Term	N1	N2	P-value	Description
GO:0007005	7	554	2.03194e-06	mitochondrion organization
GO:0040007	7	1496	0.00164951	growth
GO:0035264	4	305	0.00420943	multicellular organism growth
GO:0048731	8	2612	0.00667385	system development
GO:0032989	7	1879	0.00727234	cellular component morphogenesis
GO:0072358	2	21	0.0127182	cardiovascular system development
GO:0007568	5	848	0.0151756	aging
GO:0043436	8	3244	0.0315906	oxoacid metabolic process
GO:0006082	8	3284	0.0344482	organic acid metabolic process
GO:0007399	5	1018	0.0358418	nervous system development
GO:0032990	5	1056	0.0425273	cell part morphogenesis

Table 3: Overrepresented terms with the network-based enrichment. Only terms not detected with the standard method.
