# Supplementary material for: NET-GE: a novel NETwork-based Gene Enrichment for detecting biological processes associated to Mendelian diseases
Source: BMC Genomics. 2015 Jun 18;16(Suppl 8):S6. doi: 10.1186/1471-2164-16-S8-S6 (PMC4480278; doi:10.1186/1471-2164-16-S8-S6)
Supplement: Additional file 3 — Detailed results for the OMIM-derived benchmark set. The archive contains pdf documents listing the enriched terms for each one of the 244 diseases in the OMIM-derived benchmark set. [file 1471-2164-16-S8-S6-S3.tgz › SUPPMAT/OMIM252160.pdf]

#252160 MOLYBDENUM COFACTOR DEFICIENCY, COMPLEMENTATION GROUP B; MOCODB

OMIM Gene ID	HGNC	UniProtAC
603708	MOCS2	O96007
603708	MOCS2	O96033

Table 1: OMIM - UniProtAC mapping

Legend

- N1: #input proteins associated to the significant GO term
- N2: #proteins associated to the significant GO term
- P-value: Bonferroni-corrected p-value of Fisher's exact test
- *red*: go terms not related to the input proteins
- *blue*: go terms related to the input proteins (enriched uniquely by network-based method)
- *green*: go terms ancestors of terms enriched with the standard method (enriched uniquely by network-based method)

1 Standard enrichment

GO Term	N1	N2	P-value	Description
GO:0006777	2	17	6.87402e-06	Mo-molybdopterin cofactor biosynthetic process
GO:0019720	2	17	6.87402e-06	Mo-molybdopterin cofactor metabolic process
GO:0032324	2	17	6.87402e-06	molybdopterin cofactor biosynthetic process
GO:0043545	2	17	6.87402e-06	molybdopterin cofactor metabolic process
GO:0051189	2	17	6.87402e-06	prosthetic group metabolic process
GO:0006767	2	109	0.000297503	water-soluble vitamin metabolic process
GO:0006766	2	148	0.000549821	vitamin metabolic process
GO:0009108	2	203	0.00103631	coenzyme biosynthetic process
GO:0051188	2	252	0.00159851	cofactor biosynthetic process
GO:0006732	2	369	0.00343175	coenzyme metabolic process
GO:0051186	2	461	0.00535921	cofactor metabolic process
GO:0090407	2	789	0.0157125	organophosphate biosynthetic process
GO:1901566	2	954	0.0229764	organonitrogen compound biosynthetic process

Table 2: Overrepresented GO terms with the standard enrichment

2 Network-based enrichment

No novel enriched terms
