# Supplementary material for: NET-GE: a novel NETwork-based Gene Enrichment for detecting biological processes associated to Mendelian diseases
Source: BMC Genomics. 2015 Jun 18;16(Suppl 8):S6. doi: 10.1186/1471-2164-16-S8-S6 (PMC4480278; doi:10.1186/1471-2164-16-S8-S6)
Supplement: Additional file 3 — Detailed results for the OMIM-derived benchmark set. The archive contains pdf documents listing the enriched terms for each one of the 244 diseases in the OMIM-derived benchmark set. [file 1471-2164-16-S8-S6-S3.tgz › SUPPMAT/OMIM254500.pdf]

#254500 MYELOMA, MULTIPLE

**OMIM Gene ID**	**HGNC**	**UniProtAC**
168461	CCND1	P24385
601837	LIG4	P49917
601900	IRF4	Q15306

Table 1: OMIM - UniProtAC mapping

Legend

- N1: #input proteins associated to the significant GO term
- N2: #proteins associated to the significant GO term
- P-value: Bonferroni-corrected p-value of Fisher's exact test
- *red*: go terms not related to the input proteins
- *blue*: go terms related to the input proteins (enriched uniquely by network-based method)
- *green*: go terms ancestors of terms enriched with the standard method (enriched uniquely by network-based method)

1 Standard enrichment

GO Term	N1	N2	P-value	Description
GO:0010165	2	40	0.00147086	response to X-ray
GO:0051301	2	177	0.0293008	cell division
GO:0010212	2	188	0.0330604	response to ionizing radiation
GO:0045366	1	1	0.0356092	regulation of interleukin-13 biosynthetic process
GO:0045368	1	1	0.0356092	positive regulation of interleukin-13 biosynthetic process
GO:0051102	1	1	0.0356092	DNA ligation involved in DNA recombination

Table 2: Overrepresented GO terms with the standard enrichment

2 Network-based enrichment

GO Term	N1	N2	P-value	Description
GO:0030217	3	386	0.0024217	T cell differentiation
GO:0002244	3	406	0.00281907	hematopoietic progenitor cell differentiation
GO:0043369	2	28	0.0032046	CD4-positive or CD8-positive, alpha-beta T cell lineage commitment
GO:0042113	3	431	0.00337401	B cell activation
GO:0002360	2	38	0.00595869	T cell lineage commitment
GO:0030098	3	654	0.0118163	lymphocyte differentiation
GO:0042110	3	787	0.0206068	T cell activation
GO:0060740	2	75	0.0235036	prostate gland epithelium morphogenesis
GO:0045622	2	77	0.0247816	regulation of T-helper cell differentiation
GO:0032259	3	916	0.0325092	methylation
GO:0002521	3	929	0.0339146	leukocyte differentiation
GO:0031016	2	100	0.0419044	pancreas development

Table 3: Overrepresented terms with the network-based enrichment. Only terms not detected with the standard method.
