# Supplementary material for: NET-GE: a novel NETwork-based Gene Enrichment for detecting biological processes associated to Mendelian diseases
Source: BMC Genomics. 2015 Jun 18;16(Suppl 8):S6. doi: 10.1186/1471-2164-16-S8-S6 (PMC4480278; doi:10.1186/1471-2164-16-S8-S6)
Supplement: Additional file 3 — Detailed results for the OMIM-derived benchmark set. The archive contains pdf documents listing the enriched terms for each one of the 244 diseases in the OMIM-derived benchmark set. [file 1471-2164-16-S8-S6-S3.tgz › SUPPMAT/OMIM254780.pdf]

#254780 MYOCLONIC EPILEPSY OF LAFORA

**OMIM Gene ID**	**HGNC**	**UniProtAC**
607566	EPM2A	O95278
608072	NHLRC1	Q6VVB1

Table 1: OMIM - UniProtAC mapping

Legend

- N1: #input proteins associated to the significant GO term
- N2: #proteins associated to the significant GO term
- P-value: Bonferroni-corrected p-value of Fisher's exact test
- *red*: go terms not related to the input proteins
- *blue*: go terms related to the input proteins (enriched uniquely by network-based method)
- *green*: go terms ancestors of terms enriched with the standard method (enriched uniquely by network-based method)

1 Standard enrichment

GO Term	N1	N2	P-value	Description
GO:0005978	2	39	0.000123804	glycogen biosynthetic process
GO:0009250	2	39	0.000123804	glucan biosynthetic process
GO:0033692	2	68	0.0003806	cellular polysaccharide biosynthetic process
GO:0000271	2	72	0.000427048	polysaccharide biosynthetic process
GO:0005977	2	82	0.000554861	glycogen metabolic process
GO:0006073	2	83	0.000568562	cellular glucan metabolic process
GO:0044042	2	83	0.000568562	glucan metabolic process
GO:0034637	2	86	0.000610665	cellular carbohydrate biosynthetic process
GO:0044264	2	116	0.0011144	cellular polysaccharide metabolic process
GO:0005976	2	128	0.001358	polysaccharide metabolic process
GO:0006914	2	166	0.00228811	autophagy
GO:0006112	2	185	0.00284365	energy reserve metabolic process
GO:0006006	2	225	0.00421033	glucose metabolic process
GO:0016051	2	226	0.00424792	carbohydrate biosynthetic process
GO:0015980	2	231	0.00443839	energy derivation by oxidation of organic compounds
GO:0019318	2	321	0.00858105	hexose metabolic process
GO:0044262	2	325	0.00879659	cellular carbohydrate metabolic process
GO:0005996	2	362	0.010917	monosaccharide metabolic process
GO:0046959	1	3	0.018917	habituation
GO:0006091	2	531	0.0235102	generation of precursor metabolites and energy
GO:0046958	1	7	0.0441371	nonassociative learning

Table 2: Overrepresented GO terms with the standard enrichment

2 Network-based enrichment

No novel enriched terms
