# Supplementary material for: NET-GE: a novel NETwork-based Gene Enrichment for detecting biological processes associated to Mendelian diseases
Source: BMC Genomics. 2015 Jun 18;16(Suppl 8):S6. doi: 10.1186/1471-2164-16-S8-S6 (PMC4480278; doi:10.1186/1471-2164-16-S8-S6)
Supplement: Additional file 3 — Detailed results for the OMIM-derived benchmark set. The archive contains pdf documents listing the enriched terms for each one of the 244 diseases in the OMIM-derived benchmark set. [file 1471-2164-16-S8-S6-S3.tgz › SUPPMAT/OMIM255310.pdf]

#255310 MYOPATHY, CONGENITAL, WITH FIBER-TYPE DISPROPORTION; CFTD

OMIM Gene ID	HGNC	UniProtAC
102610	ACTA1	P68133
191030	TPM3	P06753
606210	SEPN1	Q9NZV5

Table 1: OMIM - UniProtAC mapping

Legend

- N1: #input proteins associated to the significant GO term
- N2: #proteins associated to the significant GO term
- P-value: Bonferroni-corrected p-value of Fisher's exact test
- *red*: go terms not related to the input proteins
- *blue*: go terms related to the input proteins (enriched uniquely by network-based method)
- *green*: go terms ancestors of terms enriched with the standard method (enriched uniquely by network-based method)

1 Standard enrichment

GO Term	N1	N2	P-value	Description
GO:0030049	2	38	0.000147958	muscle filament sliding
GO:0033275	2	38	0.000147958	actin-myosin filament sliding
GO:0070252	2	51	0.000268283	actin-mediated cell contraction
GO:0030048	2	74	0.00056811	actin filament-based movement
GO:0006936	2	261	0.007113	muscle contraction
GO:0043503	1	2	0.0079483	skeletal muscle fiber adaptation
GO:0003012	2	320	0.0106887	muscle system process
GO:0030029	2	510	0.0270897	actin filament-based process
GO:0030240	1	7	0.0278153	skeletal muscle thin filament assembly

Table 2: Overrepresented GO terms with the standard enrichment

2 Network-based enrichment

No novel enriched terms
