# Supplementary material for: NET-GE: a novel NETwork-based Gene Enrichment for detecting biological processes associated to Mendelian diseases
Source: BMC Genomics. 2015 Jun 18;16(Suppl 8):S6. doi: 10.1186/1471-2164-16-S8-S6 (PMC4480278; doi:10.1186/1471-2164-16-S8-S6)
Supplement: Additional file 3 — Detailed results for the OMIM-derived benchmark set. The archive contains pdf documents listing the enriched terms for each one of the 244 diseases in the OMIM-derived benchmark set. [file 1471-2164-16-S8-S6-S3.tgz › SUPPMAT/OMIM256000.pdf]

#256000 LEIGH SYNDROME; LS

OMIM Gene ID	HGNC	UniProtAC
185620	SURF1	Q15526
600857	SDHA	P31040
601825	NDUFS7	O75251
602125	COX10	Q12887
602137	NDUFA2	O43678
602141	NDUFS8	O00217
602694	NDUFS4	O43181
603646	COX15	Q7KZN9
603647	BCS1L	Q9Y276
603834	NDUFA9	Q16795
603835	NDUFA10	O95299
603846	NDUFS3	O75489
609653	NDUFAF2	Q8N183
612392	NDUFAF6	Q330K2
613622	FOXRED1	Q96CU9
614530	NDUFA12	Q9UI09

Table 1: OMIM - UniProtAC mapping

Legend

- N1: #input proteins associated to the significant GO term
- N2: #proteins associated to the significant GO term
- P-value: Bonferroni-corrected p-value of Fisher's exact test
- *red*: go terms not related to the input proteins
- *blue*: go terms related to the input proteins (enriched uniquely by network-based method)
- *green*: go terms ancestors of terms enriched with the standard method (enriched uniquely by network-based method)

1 Standard enrichment

GO Term	N1	N2	P-value	Description
GO:0022904	12	131	8.02872e-25	respiratory electron transport chain
GO:0022900	12	151	4.73075e-24	electron transport chain
GO:0006091	13	531	9.65138e-20	generation of precursor metabolites and energy
GO:0006120	7	49	1.10305e-14	mitochondrial electron transport, NADH to ubiquinone
GO:0055114	14	2084	6.21722e-14	oxidation-reduction process
GO:0010257	5	16	7.3061e-12	NADH dehydrogenase complex assembly
GO:0032981	5	16	7.3061e-12	mitochondrial respiratory chain complex I assembly
GO:0097031	5	16	7.3061e-12	mitochondrial respiratory chain complex I biogenesis
GO:0070271	5	26	1.09759e-10	protein complex biogenesis
GO:0033108	5	28	1.63908e-10	mitochondrial respiratory chain complex assembly
GO:0043623	8	393	3.77339e-10	cellular protein complex assembly
GO:0008535	4	15	7.17943e-09	respiratory chain complex IV assembly
GO:0017004	4	25	6.63654e-08	cytochrome complex assembly
GO:0034622	8	788	9.47812e-08	cellular macromolecular complex assembly
GO:0044085	5	118	2.85481e-07	cellular component biogenesis
GO:0045333	4	44	7.08753e-07	cellular respiration
GO:0044281	12	4403	1.77512e-06	small molecule metabolic process
GO:0006461	8	1365	6.97905e-06	protein complex assembly
GO:0044710	14	8611	1.87195e-05	single-organism metabolic process
GO:0065003	8	1623	2.66009e-05	macromolecular complex assembly
GO:0071822	8	1933	0.000101633	protein complex subunit organization
GO:0006123	2	3	0.000123803	mitochondrial electron transport, cytochrome c to oxygen
GO:0006784	2	3	0.000123803	heme a biosynthetic process
GO:0046160	2	3	0.000123803	heme a metabolic process
GO:0043933	8	2217	0.000288269	macromolecular complex subunit organization
GO:0015980	4	231	0.000575382	energy derivation by oxidation of organic compounds
GO:0022607	8	2496	0.000705024	cellular component assembly
GO:0072593	3	110	0.0032145	reactive oxygen species metabolic process
GO:0009060	2	27	0.0143992	aerobic respiration
GO:0006783	2	32	0.0203224	heme biosynthetic process
GO:1902600	3	232	0.0296482	hydrogen ion transmembrane transport
GO:0006779	2	45	0.0404328	porphyrin-containing compound biosynthetic process
GO:0042168	2	48	0.0460348	heme metabolic process

Table 2: Overrepresented GO terms with the standard enrichment

2 Network-based enrichment

GO Term	N1	N2	P-value	Description
GO:0007005	5	554	0.00099379	mitochondrion organization
GO:0000266	2	41	0.0371835	mitochondrial fission
GO:0009165	4	661	0.049	nucleotide biosynthetic process

Table 3: Overrepresented terms with the network-based enrichment. Only terms not detected with the standard method.
