# Supplementary material for: NET-GE: a novel NETwork-based Gene Enrichment for detecting biological processes associated to Mendelian diseases
Source: BMC Genomics. 2015 Jun 18;16(Suppl 8):S6. doi: 10.1186/1471-2164-16-S8-S6 (PMC4480278; doi:10.1186/1471-2164-16-S8-S6)
Supplement: Additional file 3 — Detailed results for the OMIM-derived benchmark set. The archive contains pdf documents listing the enriched terms for each one of the 244 diseases in the OMIM-derived benchmark set. [file 1471-2164-16-S8-S6-S3.tgz › SUPPMAT/OMIM259500.pdf]

#259500 OSTEOGENIC SARCOMA

OMIM Gene ID	HGNC	UniProtAC
191170	TP53	P04637
604373	CHEK2	O96017
614041	RB1	P06400

Table 1: OMIM - UniProtAC mapping

Legend

- N1: #input proteins associated to the significant GO term
- N2: #proteins associated to the significant GO term
- P-value: Bonferroni-corrected p-value of Fisher's exact test
- *red*: go terms not related to the input proteins
- *blue*: go terms related to the input proteins (enriched uniquely by network-based method)
- *green*: go terms ancestors of terms enriched with the standard method (enriched uniquely by network-based method)

1 Standard enrichment

GO Term	N1	N2	P-value	Description
GO:0034349	2	8	7.24053e-05	glial cell apoptotic process
GO:0000075	3	241	0.000157877	cell cycle checkpoint
GO:1901988	3	259	0.000196131	negative regulation of cell cycle phase transition
GO:1902850	2	15	0.000271486	microtubule cytoskeleton organization involved in mitosis
GO:0090399	2	17	0.000351627	replicative senescence
GO:1901987	3	350	0.000485474	regulation of cell cycle phase transition
GO:0010948	3	358	0.000519631	negative regulation of cell cycle process
GO:0010564	3	662	0.00329835	regulation of cell cycle process
GO:0008156	2	53	0.00356054	negative regulation of DNA replication
GO:0010332	2	65	0.00537326	response to gamma radiation
GO:0051402	2	68	0.00588444	neuron apoptotic process
GO:0072401	2	72	0.0066021	signal transduction involved in DNA integrity checkpoint
GO:0072413	2	72	0.0066021	signal transduction involved in mitotic cell cycle checkpoint
GO:0072422	2	72	0.0066021	signal transduction involved in DNA damage checkpoint
GO:1902402	2	72	0.0066021	signal transduction involved in mitotic DNA damage checkpoint
GO:1902403	2	72	0.0066021	signal transduction involved in mitotic DNA integrity checkpoint
GO:0072395	2	73	0.00678795	signal transduction involved in cell cycle checkpoint
GO:1903047	3	886	0.0079163	mitotic cell cycle process
GO:0070997	2	80	0.00816104	neuron death
GO:0007569	2	93	0.0110459	cell aging
GO:0051053	2	108	0.014915	negative regulation of DNA metabolic process
GO:0042770	2	118	0.0178158	signal transduction in response to DNA damage
GO:1902807	2	124	0.0196797	negative regulation of cell cycle G1/S phase transition
GO:2000134	2	124	0.0196797	negative regulation of G1/S transition of mitotic cell cycle
GO:0008630	2	126	0.0203216	intrinsic apoptotic signaling pathway in response to DNA damage
GO:0051726	3	1232	0.0213043	regulation of cell cycle
GO:0000077	2	130	0.0216361	DNA damage checkpoint
GO:0097285	2	135	0.023337	cell-type specific apoptotic process
GO:0031570	2	140	0.0251022	DNA integrity checkpoint
GO:0007093	2	148	0.0280599	mitotic cell cycle checkpoint
GO:0022402	3	1409	0.0318788	cell cycle process
GO:0006275	2	163	0.0340482	regulation of DNA replication
GO:2000045	2	164	0.0344679	regulation of G1/S transition of mitotic cell cycle
GO:1902806	2	166	0.0353151	regulation of cell cycle G1/S phase transition
GO:0006302	2	175	0.0392542	double-strand break repair
GO:0010212	2	188	0.0453104	response to ionizing radiation
GO:0007050	2	189	0.0457943	cell cycle arrest
GO:2000113	3	1622	0.0486455	negative regulation of cellular macromolecule biosynthetic process
GO:0072428	1	1	0.0488037	signal transduction involved in intra-S DNA damage checkpoint
GO:0033044	2	197	0.0497568	regulation of chromosome organization

Table 2: Overrepresented GO terms with the standard enrichment

2 Network-based enrichment

GO Term	N1	N2	P-value	Description
GO:0000003	3	76	2.56347e-05	reproduction
GO:0021545	3	82	3.22932e-05	cranial nerve development
GO:0021533	3	95	5.04728e-05	cell differentiation in hindbrain
GO:0043966	3	99	5.71947e-05	histone H3 acetylation
GO:0021675	3	133	0.000139772	nerve development
GO:0030518	3	149	0.00019701	intracellular steroid hormone receptor signaling pathway
GO:0021559	2	7	0.000255073	trigeminal nerve development
GO:0007051	3	202	0.000493515	spindle organization
GO:0042472	3	207	0.000531269	inner ear morphogenesis
GO:0046425	3	211	0.000562822	regulation of JAK-STAT cascade
GO:0016573	3	238	0.000809021	histone acetylation
GO:0000086	3	254	0.000984186	G2/M transition of mitotic cell cycle
GO:0044839	3	254	0.000984186	cell cycle G2/M phase transition
GO:0018393	3	258	0.00103161	internal peptidyl-lysine acetylation
GO:0018394	3	260	0.00105588	peptidyl-lysine acetylation
GO:0006475	3	269	0.00116983	internal protein amino acid acetylation
GO:0060021	3	269	0.00116983	palate development
GO:0042475	3	273	0.00122299	odontogenesis of dentin-containing tooth
GO:0009798	3	275	0.00125017	axis specification
GO:0000209	3	287	0.00142172	protein polyubiquitination
GO:0032466	2	16	0.00145729	negative regulation of cytokinesis
GO:0006473	3	292	0.0014976	protein acetylation
GO:0090239	2	18	0.00185798	regulation of histone H4 acetylation
GO:0060560	3	320	0.00197283	developmental growth involved in morphogenesis
GO:0021516	2	20	0.0023072	dorsal spinal cord development
GO:0008343	2	21	0.00255001	adult feeding behavior
GO:0001568	3	358	0.00276518	blood vessel development
GO:0021953	3	365	0.00293105	central nervous system neuron differentiation
GO:0042476	3	366	0.00295528	odontogenesis
GO:0090307	2	24	0.00335124	spindle assembly involved in mitosis
GO:0051301	3	386	0.00346818	cell division
GO:0035148	3	396	0.0037455	tube formation
GO:0045787	3	401	0.00388956	positive regulation of cell cycle
GO:0043543	3	403	0.00394818	protein acylation
GO:0048705	3	410	0.00415805	skeletal system morphogenesis
GO:0097193	3	421	0.00450265	intrinsic apoptotic signaling pathway
GO:0007423	3	423	0.00456727	sensory organ development
GO:0051782	2	28	0.00458939	negative regulation of cell division
GO:0008406	3	424	0.00459983	gonad development
GO:0018205	3	438	0.00507184	peptidyl-lysine modification
GO:0051783	3	444	0.00528362	regulation of nuclear division
GO:0007265	3	457	0.00576257	Ras protein signal transduction
GO:0048708	2	32	0.00602156	astrocyte differentiation
GO:0048469	3	468	0.00618972	cell maturation
GO:0009266	3	469	0.00622958	response to temperature stimulus
GO:0030856	3	470	0.0062696	regulation of epithelial cell differentiation
GO:0048568	3	476	0.00651331	embryonic organ development
GO:0001822	3	489	0.00706286	kidney development
GO:0045165	3	501	0.00759681	cell fate commitment
GO:0034599	3	502	0.00764247	cellular response to oxidative stress
GO:0010565	3	517	0.00834969	regulation of cellular ketone metabolic process
GO:0046777	3	520	0.00849618	protein autophosphorylation
GO:0007417	3	521	0.00854539	central nervous system development
GO:0048666	3	539	0.00946385	neuron development
GO:0030522	3	544	0.00973018	intracellular receptor signaling pathway
GO:0001764	3	578	0.0116748	neuron migration
GO:0060707	2	45	0.0120157	trophoblast giant cell differentiation
GO:0009952	3	585	0.0121049	anterior/posterior pattern specification
GO:0044772	3	590	0.0124184	mitotic cell cycle phase transition
GO:0044770	3	595	0.0127374	cell cycle phase transition

Table 3: Overrepresented terms with the network-based enrichment. Only terms not detected with the standard method.

**GO Term**	**N1**	**N2**	**P-value**	**Description**
GO:0007067	3	605	0.0133916	mitotic nuclear division
GO:0030163	3	637	0.0156348	protein catabolic process
GO:0007507	3	638	0.0157087	heart development
GO:0016048	2	52	0.0160915	detection of temperature stimulus
GO:0008585	2	53	0.0167222	female gonad development
GO:0032504	2	53	0.0167222	multicellular organism reproduction
GO:0045445	2	54	0.017365	myoblast differentiation
GO:0050769	3	660	0.0173931	positive regulation of neurogenesis
GO:0035295	3	664	0.0177118	tube development
GO:0021700	3	666	0.0178725	developmental maturation
GO:0007052	2	55	0.0180199	mitotic spindle organization
GO:0006469	3	668	0.0180343	negative regulation of protein kinase activity
GO:0090276	3	675	0.0186081	regulation of peptide hormone secretion
GO:0009581	3	680	0.0190253	detection of external stimulus
GO:0002791	3	684	0.0193635	regulation of peptide secretion
GO:0002040	2	57	0.0193661	sprouting angiogenesis
GO:0009582	3	688	0.0197057	detection of abiotic stimulus
GO:0090087	3	688	0.0197057	regulation of peptide transport
GO:0033673	3	709	0.0215686	negative regulation of kinase activity
GO:0048589	3	714	0.0220288	developmental growth
GO:0021884	2	62	0.0229434	forebrain neuron development
GO:0030521	2	63	0.0236951	androgen receptor signaling pathway
GO:0010927	3	737	0.0242301	cellular component assembly involved in morphogenesis
GO:0007601	3	760	0.0265735	visual perception
GO:0010842	2	67	0.0268233	retina layer formation
GO:0050953	3	769	0.02753	sensory perception of light stimulus
GO:0042474	2	68	0.0276354	middle ear morphogenesis
GO:0070936	2	68	0.0276354	protein K48-linked ubiquitination
GO:0007420	3	791	0.0299642	brain development
GO:0007611	3	793	0.0301925	learning or memory
GO:0051302	3	796	0.0305367	regulation of cell division
GO:0046883	3	805	0.0315856	regulation of hormone secretion
GO:0002052	2	73	0.0318784	positive regulation of neuroblast proliferation
GO:0042176	3	816	0.0328999	regulation of protein catabolic process
GO:0060706	2	75	0.0336601	cell differentiation involved in embryonic placenta development
GO:0045786	3	824	0.0338782	negative regulation of cell cycle
GO:0000226	3	828	0.0343746	microtubule cytoskeleton organization
GO:0007626	3	829	0.0344995	locomotory behavior
GO:0006284	2	77	0.0354902	base-excision repair
GO:0019216	3	845	0.0365385	regulation of lipid metabolic process
GO:0007568	3	848	0.0369294	aging
GO:0021522	2	80	0.0383262	spinal cord motor neuron differentiation
GO:0090329	2	81	0.0392958	regulation of DNA-dependent DNA replication
GO:0043254	3	874	0.0404357	regulation of protein complex assembly
GO:0051348	3	887	0.0422692	negative regulation of transferase activity
GO:0018022	2	84	0.0422769	peptidyl-lysine methylation
GO:0050890	3	896	0.0435705	cognition
GO:0051052	3	905	0.044898	regulation of DNA metabolic process
GO:0030182	3	911	0.0457979	neuron differentiation
GO:0016570	3	929	0.0485698	histone modification
GO:0016569	3	935	0.049518	covalent chromatin modification

Table 4: Overrepresented terms with the network-based enrichment. Only terms not detected with the standard method.
