# Supplementary material for: NET-GE: a novel NETwork-based Gene Enrichment for detecting biological processes associated to Mendelian diseases
Source: BMC Genomics. 2015 Jun 18;16(Suppl 8):S6. doi: 10.1186/1471-2164-16-S8-S6 (PMC4480278; doi:10.1186/1471-2164-16-S8-S6)
Supplement: Additional file 3 — Detailed results for the OMIM-derived benchmark set. The archive contains pdf documents listing the enriched terms for each one of the 244 diseases in the OMIM-derived benchmark set. [file 1471-2164-16-S8-S6-S3.tgz › SUPPMAT/OMIM260350.pdf]

#260350 PANCREATIC CANCER

**OMIM Gene ID**	**HGNC**	**UniProtAC**
190070	KRAS	P01116
191170	TP53	P04637
600993	SMAD4	Q13485
602216	STK11	Q15831

Table 1: OMIM - UniProtAC mapping

Legend

- N1: #input proteins associated to the significant GO term
- N2: #proteins associated to the significant GO term
- P-value: Bonferroni-corrected p-value of Fisher's exact test
- *red*: go terms not related to the input proteins
- *blue*: go terms related to the input proteins (enriched uniquely by network-based method)
- *green*: go terms ancestors of terms enriched with the standard method (enriched uniquely by network-based method)

1 Standard enrichment

GO Term	N1	N2	P-value	Description
GO:0017015	3	127	0.000122625	regulation of transforming growth factor beta receptor signaling pathway
GO:0001934	4	956	0.000337914	positive regulation of protein phosphorylation
GO:0030308	3	199	0.000475176	negative regulation of cell growth
GO:0007167	4	1091	0.000573603	enzyme linked receptor protein signaling pathway
GO:0042327	4	1129	0.000657913	positive regulation of phosphorylation
GO:0090287	3	237	0.000804024	regulation of cellular response to growth factor stimulus
GO:0090092	3	239	0.000824607	regulation of transmembrane receptor protein serine/threonine kinase signaling pathway
GO:0031401	4	1213	0.000876997	positive regulation of protein modification process
GO:0010562	4	1255	0.00100508	positive regulation of phosphorus metabolic process
GO:0045937	4	1255	0.00100508	positive regulation of phosphate metabolic process
GO:0071495	4	1291	0.00112561	cellular response to endogenous stimulus
GO:0032270	4	1363	0.00139886	positive regulation of cellular protein metabolic process
GO:0045926	3	303	0.00168261	negative regulation of growth
GO:0001932	4	1440	0.00174318	regulation of protein phosphorylation
GO:0009628	4	1467	0.00187779	response to abiotic stimulus
GO:0051247	4	1526	0.00219894	positive regulation of protein metabolic process
GO:0009967	4	1548	0.00232864	positive regulation of signal transduction
GO:0030511	2	29	0.00282235	positive regulation of transforming growth factor beta receptor signaling pathway
GO:0023056	4	1630	0.00286321	positive regulation of signaling
GO:0010647	4	1637	0.00291277	positive regulation of cell communication
GO:0042325	4	1770	0.0039822	regulation of phosphorylation
GO:0031399	4	1823	0.00448146	regulation of protein modification process
GO:0001558	3	455	0.00569918	regulation of cell growth
GO:0042127	4	2008	0.00659872	regulation of cell proliferation
GO:0009719	4	2012	0.0066515	response to endogenous stimulus
GO:0045595	4	2111	0.0080616	regulation of cell differentiation
GO:0009653	4	2131	0.00837168	anatomical structure morphogenesis
GO:0032268	4	2272	0.010819	regulation of cellular protein metabolic process
GO:0072332	2	58	0.0114793	intrinsic apoptotic signaling pathway by p53 class mediator
GO:0048584	4	2308	0.0115217	positive regulation of response to stimulus
GO:0009314	3	591	0.0124743	response to radiation
GO:0071310	4	2482	0.015412	cellular response to organic substance
GO:0035556	4	2537	0.0168251	intracellular signal transduction
GO:0071363	3	762	0.0266758	cellular response to growth factor stimulus
GO:0007369	2	89	0.0271648	gastrulation
GO:0050793	4	2884	0.0281048	regulation of developmental process
GO:0070887	4	2904	0.028893	cellular response to chemical stimulus
GO:0070848	3	793	0.0300516	response to growth factor
GO:0051246	4	2954	0.0309359	regulation of protein metabolic process
GO:0019220	4	2977	0.0319112	regulation of phosphate metabolic process
GO:0051174	4	2996	0.0327341	regulation of phosphorus metabolic process
GO:0040008	3	822	0.0334555	regulation of growth
GO:0090100	2	103	0.0364214	positive regulation of transmembrane receptor protein serine/threonine kinase signaling pathway
GO:0009966	4	3261	0.0459523	regulation of signal transduction
GO:0008285	3	920	0.04683	negative regulation of cell proliferation
GO:0010604	4	3285	0.0473207	positive regulation of macromolecule metabolic process

Table 2: Overrepresented GO terms with the standard enrichment

2 Network-based enrichment

GO Term	N1	N2	P-value	Description
GO:0051403	4	197	2.9519e-06	stress-activated MAPK cascade
GO:0031098	4	201	3.20103e-06	stress-activated protein kinase signaling cascade
GO:0050680	4	392	4.69916e-05	negative regulation of epithelial cell proliferation
GO:0007050	4	403	5.25144e-05	cell cycle arrest
GO:0032409	4	500	0.000124796	regulation of transporter activity
GO:0000165	4	537	0.00016618	MAPK cascade
GO:2001236	4	581	0.000227905	regulation of extrinsic apoptotic signaling pathway
GO:0032869	4	609	0.000275249	cellular response to insulin stimulus
GO:0048536	3	102	0.000276794	spleen development
GO:0023014	4	653	0.000364081	signal transduction by phosphorylation
GO:0043406	4	661	0.000382295	positive regulation of MAP kinase activity
GO:0050730	4	663	0.000386955	regulation of peptidyl-tyrosine phosphorylation
GO:0032410	3	122	0.000475735	negative regulation of transporter activity
GO:0071496	4	736	0.000588178	cellular response to external stimulus
GO:0046632	3	137	0.000675283	alpha-beta T cell differentiation
GO:0000902	4	800	0.000821564	cell morphogenesis
GO:0001709	3	147	0.000835281	cell fate determination
GO:0045786	4	824	0.00092488	negative regulation of cell cycle
GO:0032868	4	834	0.000970688	response to insulin
GO:0010959	4	835	0.000975359	regulation of metal ion transport
GO:0071375	4	846	0.00102788	cellular response to peptide hormone stimulus
GO:0046631	3	159	0.00105835	alpha-beta T cell activation
GO:0035019	3	163	0.00114069	somatic stem cell maintenance
GO:0031064	2	10	0.00121063	negative regulation of histone deacetylation
GO:1901653	4	892	0.0012708	cellular response to peptide
GO:0071902	4	897	0.00129959	positive regulation of protein serine/threonine kinase activity
GO:0043405	4	930	0.001502	regulation of MAP kinase activity
GO:0001933	4	963	0.00172719	negative regulation of protein phosphorylation
GO:0030072	3	189	0.00178174	peptide hormone secretion
GO:0002757	4	972	0.00179277	immune response-activating signal transduction
GO:0034762	4	998	0.00199275	regulation of transmembrane transport
GO:0002790	3	199	0.00208098	peptide secretion
GO:0050678	4	1020	0.00217464	regulation of epithelial cell proliferation
GO:0032088	3	213	0.00255354	negative regulation of NF-kappaB transcription factor activity
GO:0002526	3	220	0.00281449	acute inflammatory response
GO:0097529	3	222	0.00289219	myeloid leukocyte migration
GO:0002253	4	1110	0.00305131	activation of immune response
GO:0010769	4	1115	0.00310672	regulation of cell morphogenesis involved in differentiation
GO:0030855	4	1135	0.00333603	epithelial cell differentiation
GO:0009416	4	1146	0.00346743	response to light stimulus
GO:0060070	3	237	0.00352079	canonical Wnt signaling pathway
GO:0042326	4	1164	0.00369076	negative regulation of phosphorylation
GO:0046879	3	253	0.00428496	hormone secretion
GO:0015833	3	254	0.00433609	peptide transport
GO:0055065	4	1233	0.00464816	metal ion homeostasis
GO:0009267	3	263	0.00481452	cellular response to starvation
GO:0002764	4	1248	0.00487879	immune response-regulating signaling pathway
GO:0043410	4	1269	0.00521597	positive regulation of MAPK cascade
GO:0006338	3	273	0.0053859	chromatin remodeling
GO:0042102	3	273	0.0053859	positive regulation of T cell proliferation
GO:0010975	4	1281	0.00541631	regulation of neuron projection development
GO:0043434	4	1303	0.00579855	response to peptide hormone
GO:0097285	3	280	0.00581159	cell-type specific apoptotic process
GO:2001233	4	1313	0.00597883	regulation of apoptotic signaling pathway
GO:0071456	3	283	0.00600068	cellular response to hypoxia
GO:0044057	4	1316	0.00603373	regulation of system process
GO:0051705	3	285	0.00612899	multi-organism behavior
GO:0009914	3	288	0.00632485	hormone transport
GO:0036294	3	288	0.00632485	cellular response to decreased oxygen levels
GO:0016568	4	1339	0.00646724	chromatin modification

Table 3: Overrepresented terms with the network-based enrichment. Only terms not detected with the standard method.

GO Term	N1	N2	P-value	Description
GO:0010563	4	1353	0.0067423	negative regulation of phosphorus metabolic process
GO:0045936	4	1353	0.0067423	negative regulation of phosphate metabolic process
GO:0048332	2	23	0.00680291	mesoderm morphogenesis
GO:0051259	4	1363	0.00694406	protein oligomerization
GO:0007596	4	1367	0.00702602	blood coagulation
GO:0050817	4	1367	0.00702602	coagulation
GO:0031400	4	1368	0.00704663	negative regulation of protein modification process
GO:1901652	4	1373	0.00715035	response to peptide
GO:0051051	4	1375	0.00719214	negative regulation of transport
GO:0042886	3	301	0.00722171	amide transport
GO:2000736	3	302	0.00729399	regulation of stem cell differentiation
GO:0007599	4	1382	0.00733989	hemostasis
GO:0055080	4	1386	0.00742531	cation homeostasis
GO:0009566	3	305	0.00751377	fertilization
GO:0043065	4	1406	0.00786377	positive regulation of apoptotic process
GO:0043068	4	1416	0.00809012	positive regulation of programmed cell death
GO:0071453	3	319	0.00859768	cellular response to oxygen levels
GO:0042493	4	1473	0.00947513	response to drug
GO:1901615	4	1475	0.00952672	organic hydroxy compound metabolic process
GO:0071417	4	1481	0.00968286	cellular response to organonitrogen compound
GO:0050778	4	1484	0.00976163	positive regulation of immune response
GO:0071900	4	1492	0.00997405	regulation of protein serine/threonine kinase activity
GO:0003215	2	28	0.010162	cardiac right ventricle morphogenesis
GO:0006633	3	339	0.0103194	fatty acid biosynthetic process
GO:0071705	4	1511	0.0104924	nitrogen compound transport
GO:0010212	3	346	0.0109721	response to ionizing radiation
GO:0032940	4	1530	0.0110308	secretion by cell
GO:0010942	4	1542	0.0113813	positive regulation of cell death
GO:0050801	4	1555	0.0117703	ion homeostasis
GO:0006325	4	1572	0.0122941	chromatin organization
GO:0043269	4	1590	0.0128674	regulation of ion transport
GO:0032787	4	1592	0.0129324	monocarboxylic acid metabolic process
GO:0002064	3	370	0.0134177	epithelial cell development
GO:0031347	4	1612	0.0135952	regulation of defense response
GO:0022604	4	1615	0.0136968	regulation of cell morphogenesis
GO:0008286	3	375	0.0139689	insulin receptor signaling pathway
GO:1901699	4	1626	0.0140742	cellular response to nitrogen compound
GO:0001649	3	378	0.0143068	osteoblast differentiation
GO:0032870	4	1636	0.0144239	cellular response to hormone stimulus
GO:0045860	4	1636	0.0144239	positive regulation of protein kinase activity
GO:0031344	4	1640	0.0145656	regulation of cell projection organization
GO:0043687	3	383	0.014882	post-translational protein modification
GO:0060039	2	34	0.0150781	pericardium development
GO:0030217	3	386	0.0152343	T cell differentiation
GO:0050671	3	392	0.0159555	positive regulation of lymphocyte proliferation
GO:0035239	3	393	0.0160779	tube morphogenesis
GO:0032946	3	396	0.0164488	positive regulation of mononuclear cell proliferation
GO:0048485	2	36	0.0169312	sympathetic nervous system development
GO:0061140	2	36	0.0169312	lung secretory cell differentiation
GO:0031669	3	400	0.016952	cellular response to nutrient levels
GO:0033674	4	1706	0.0170581	positive regulation of kinase activity
GO:0070665	3	403	0.0173361	positive regulation of leukocyte proliferation
GO:0048617	2	37	0.017898	embryonic foregut morphogenesis
GO:0061024	4	1750	0.0188889	membrane organization
GO:0032026	2	39	0.019912	response to magnesium ion
GO:0060395	2	39	0.019912	SMAD protein signal transduction
GO:0001817	4	1774	0.0199475	regulation of cytokine production
GO:0042594	3	427	0.0206189	response to starvation
GO:0032269	4	1804	0.0213326	negative regulation of cellular protein metabolic process
GO:0043433	3	432	0.021351	negative regulation of sequence-specific DNA binding transcription factor activity

Table 4: Overrepresented terms with the network-based enrichment. Only terms not detected with the standard method.

GO Term	N1	N2	P-value	Description
GO:0000075	3	434	0.0216487	cell cycle checkpoint
GO:0023061	3	434	0.0216487	signal release
GO:0032925	2	41	0.0220331	regulation of activin receptor signaling pathway
GO:0042692	3	437	0.0221003	muscle cell differentiation
GO:0042129	3	439	0.0224048	regulation of T cell proliferation
GO:0032412	3	442	0.0228667	regulation of ion transmembrane transporter activity
GO:0043408	4	1837	0.0229383	regulation of MAPK cascade
GO:0050878	4	1837	0.0229383	regulation of body fluid levels
GO:0050808	3	446	0.0234925	synapse organization
GO:0044723	4	1859	0.024058	single-organism carbohydrate metabolic process
GO:0032989	4	1879	0.025111	cellular component morphogenesis
GO:0007265	3	457	0.0252718	Ras protein signal transduction
GO:0019932	3	457	0.0252718	second-messenger-mediated signaling
GO:0022898	3	457	0.0252718	regulation of transmembrane transporter activity
GO:0050768	3	457	0.0252718	negative regulation of neurogenesis
GO:0043524	3	459	0.0256046	negative regulation of neuron apoptotic process
GO:0045664	4	1894	0.0259232	regulation of neuron differentiation
GO:0046903	4	1910	0.026811	secretion
GO:0048469	3	468	0.0271383	cell maturation
GO:0051347	4	1933	0.0281269	positive regulation of transferase activity
GO:0007409	3	476	0.0285518	axonogenesis
GO:0031668	3	479	0.0290942	cellular response to extracellular stimulus
GO:0007605	3	487	0.0305741	sensory perception of sound
GO:0046683	3	488	0.0307625	response to organophosphorus
GO:0007281	3	489	0.0309517	germ cell development
GO:0060561	2	49	0.0315886	apoptotic process involved in morphogenesis
GO:0034599	3	502	0.0334818	cellular response to oxidative stress
GO:0050731	3	503	0.0336819	positive regulation of peptidyl-tyrosine phosphorylation
GO:0043903	3	510	0.0351051	regulation of symbiosis, encompassing mutualism through parasitism
GO:0050954	3	510	0.0351051	sensory perception of mechanical stimulus
GO:0050770	3	511	0.0353117	regulation of axonogenesis
GO:0032007	2	52	0.0356134	negative regulation of TOR signaling
GO:1902230	2	52	0.0356134	negative regulation of intrinsic apoptotic signaling pathway in response to DNA damage
GO:0010565	3	517	0.0365676	regulation of cellular ketone metabolic process
GO:0006886	4	2070	0.036997	intracellular protein transport
GO:0060441	2	53	0.0370084	epithelial tube branching involved in lung morphogenesis
GO:0090342	2	53	0.0370084	regulation of cell aging
GO:0090150	3	524	0.03807	establishment of protein localization to membrane
GO:1901215	3	524	0.03807	negative regulation of neuron death
GO:1901797	2	54	0.0384304	negative regulation of signal transduction by p53 class mediator
GO:0051046	4	2094	0.038744	regulation of secretion
GO:0043270	3	532	0.0398367	positive regulation of ion transport
GO:0036296	2	56	0.0413542	response to increased oxygen levels
GO:0055093	2	56	0.0413542	response to hyperoxia
GO:2000647	2	56	0.0413542	negative regulation of stem cell proliferation
GO:0048754	3	542	0.0421204	branching morphogenesis of an epithelial tube
GO:1901532	2	57	0.0428563	regulation of hematopoietic progenitor cell differentiation
GO:0032535	3	546	0.0430577	regulation of cellular component size
GO:0006915	4	2154	0.0433828	apoptotic process
GO:0051924	3	553	0.0447311	regulation of calcium ion transport
GO:0014074	3	555	0.0452169	response to purine-containing compound
GO:0031589	3	556	0.0454611	cell-substrate adhesion
GO:0051240	4	2185	0.0459362	positive regulation of multicellular organismal process
GO:0051248	4	2189	0.046274	negative regulation of protein metabolic process
GO:0051384	3	564	0.0474468	response to glucocorticoid
GO:0032321	3	565	0.0476992	positive regulation of Rho GTPase activity
GO:0042445	3	566	0.0479521	hormone metabolic process
GO:0033500	3	567	0.0482062	carbohydrate homeostasis
GO:0042593	3	567	0.0482062	glucose homeostasis
GO:0060850	2	61	0.0491321	regulation of transcription involved in cell fate commitment

Table 5: Overrepresented terms with the network-based enrichment. Only terms not detected with the standard method.

GO Term	N1	N2	P-value	Description
GO:0018193	4	2224	0.0493073	peptidyl-amino acid modification
GO:0045596	4	2226	0.0494851	negative regulation of cell differentiation

Table 6: Overrepresented terms with the network-based enrichment. Only terms not detected with the standard method.
