# Supplementary material for: NET-GE: a novel NETwork-based Gene Enrichment for detecting biological processes associated to Mendelian diseases
Source: BMC Genomics. 2015 Jun 18;16(Suppl 8):S6. doi: 10.1186/1471-2164-16-S8-S6 (PMC4480278; doi:10.1186/1471-2164-16-S8-S6)
Supplement: Additional file 3 — Detailed results for the OMIM-derived benchmark set. The archive contains pdf documents listing the enriched terms for each one of the 244 diseases in the OMIM-derived benchmark set. [file 1471-2164-16-S8-S6-S3.tgz › SUPPMAT/OMIM261100.pdf]

#261100 MEGALOBLASTIC ANEMIA 1

OMIM Gene ID	HGNC	UniProtAC
602997	CUBN	O60494
605799	AMN	Q9BXJ7

Table 1: OMIM - UniProtAC mapping

Legend

- N1: #input proteins associated to the significant GO term
- N2: #proteins associated to the significant GO term
- P-value: Bonferroni-corrected p-value of Fisher's exact test
- *red*: go terms not related to the input proteins
- *blue*: go terms related to the input proteins (enriched uniquely by network-based method)
- *green*: go terms ancestors of terms enriched with the standard method (enriched uniquely by network-based method)

1 Standard enrichment

GO Term	N1	N2	P-value	Description
GO:0015889	2	7	1.97543e-06	cobalamin transport
GO:0009235	2	21	1.97543e-05	cobalamin metabolic process
GO:0051180	2	50	0.000115234	vitamin transport
GO:0042157	2	87	0.000351909	lipoprotein metabolic process
GO:0033013	2	99	0.000456326	tetrapyrrole metabolic process
GO:0006767	2	109	0.000553686	water-soluble vitamin metabolic process
GO:0006766	2	148	0.00102328	vitamin metabolic process
GO:0006898	2	279	0.00364806	receptor-mediated endocytosis
GO:0006897	2	537	0.013538	endocytosis
GO:0071705	2	691	0.0224254	nitrogen compound transport

Table 2: Overrepresented GO terms with the standard enrichment

2 Network-based enrichment

No novel enriched terms
