# Supplementary material for: NET-GE: a novel NETwork-based Gene Enrichment for detecting biological processes associated to Mendelian diseases
Source: BMC Genomics. 2015 Jun 18;16(Suppl 8):S6. doi: 10.1186/1471-2164-16-S8-S6 (PMC4480278; doi:10.1186/1471-2164-16-S8-S6)
Supplement: Additional file 3 — Detailed results for the OMIM-derived benchmark set. The archive contains pdf documents listing the enriched terms for each one of the 244 diseases in the OMIM-derived benchmark set. [file 1471-2164-16-S8-S6-S3.tgz › SUPPMAT/OMIM261550.pdf]

#261550 PERSISTENT MULLERIAN DUCT SYNDROME, TYPES I AND II; PMDS

OMIM Gene ID	HGNC	UniProtAC
600956	AMHR2	Q16671
600957	AMH	P03971

Table 1: OMIM - UniProtAC mapping

Legend

- N1: #input proteins associated to the significant GO term
- N2: #proteins associated to the significant GO term
- P-value: Bonferroni-corrected p-value of Fisher's exact test
- *red*: go terms not related to the input proteins
- *blue*: go terms related to the input proteins (enriched uniquely by network-based method)
- *green*: go terms ancestors of terms enriched with the standard method (enriched uniquely by network-based method)

1 Standard enrichment

GO Term	N1	N2	P-value	Description
GO:0001880	2	9	5.2566e-06	Mullerian duct regression
GO:0007548	2	22	3.37298e-05	sex differentiation
GO:0060033	2	22	3.37298e-05	anatomical structure regression
GO:0001546	1	2	0.0110218	preantral ovarian follicle growth
GO:1902612	1	2	0.0110218	regulation of anti-Mullerian hormone signaling pathway
GO:1902613	1	2	0.0110218	negative regulation of anti-Mullerian hormone signaling pathway
GO:1990262	1	2	0.0110218	anti-Mullerian hormone signaling pathway
GO:0007506	1	8	0.0440835	gonadal mesoderm development

Table 2: Overrepresented GO terms with the standard enrichment

2 Network-based enrichment

GO Term	N1	N2	P-value	Description
GO:0022602	2	294	0.00791678	ovulation cycle process
GO:0071560	2	511	0.023951	cellular response to transforming growth factor beta stimulus
GO:0071559	2	521	0.0248986	response to transforming growth factor beta
GO:0007178	2	618	0.0350435	transmembrane receptor protein serine/threonine kinase signaling pathway
GO:0023014	2	653	0.0391286	signal transduction by phosphorylation
GO:0051091	2	677	0.04206	positive regulation of sequence-specific DNA binding transcription factor activity

Table 3: Overrepresented terms with the network-based enrichment. Only terms not detected with the standard method.
