# Supplementary material for: NET-GE: a novel NETwork-based Gene Enrichment for detecting biological processes associated to Mendelian diseases
Source: BMC Genomics. 2015 Jun 18;16(Suppl 8):S6. doi: 10.1186/1471-2164-16-S8-S6 (PMC4480278; doi:10.1186/1471-2164-16-S8-S6)
Supplement: Additional file 3 — Detailed results for the OMIM-derived benchmark set. The archive contains pdf documents listing the enriched terms for each one of the 244 diseases in the OMIM-derived benchmark set. [file 1471-2164-16-S8-S6-S3.tgz › SUPPMAT/OMIM264350.pdf]

#264350 PSEUDOHYPOALDOSTERONISM, TYPE I, AUTOSOMAL RECESSIVE; PHA1B

OMIM Gene ID	HGNC	UniProtAC
600228	SCNN1A	P37088
600760	SCNN1B	P51168
600761	SCNN1G	P51170

Table 1: OMIM - UniProtAC mapping

Legend

- N1: #input proteins associated to the significant GO term
- N2: #proteins associated to the significant GO term
- P-value: Bonferroni-corrected p-value of Fisher's exact test
- *red*: go terms not related to the input proteins
- *blue*: go terms related to the input proteins (enriched uniquely by network-based method)
- *green*: go terms ancestors of terms enriched with the standard method (enriched uniquely by network-based method)

1 Standard enrichment

GO Term	N1	N2	P-value	Description
GO:0055078	3	26	1.16067e-08	sodium ion homeostasis
GO:0050891	3	36	3.18739e-08	multicellular organismal water homeostasis
GO:0050909	3	40	4.41056e-08	sensory perception of taste
GO:0030104	3	45	6.3346e-08	water homeostasis
GO:0007588	3	51	9.29656e-08	excretion
GO:0055067	3	104	8.12936e-07	monovalent inorganic cation homeostasis
GO:0007606	3	116	1.13148e-06	sensory perception of chemical stimulus
GO:0035725	3	140	1.99805e-06	sodium ion transmembrane transport
GO:0006814	3	233	9.29056e-06	sodium ion transport
GO:0007600	3	586	0.000148954	sensory perception
GO:0055065	3	593	0.000154365	metal ion homeostasis
GO:0055080	3	656	0.000209078	cation homeostasis
GO:0050801	3	709	0.000264049	ion homeostasis
GO:0050878	3	717	0.000273101	regulation of body fluid levels
GO:0015672	3	743	0.000303946	monovalent inorganic cation transport
GO:0098662	3	848	0.0004521	inorganic cation transmembrane transport
GO:0098660	3	995	0.000730708	inorganic ion transmembrane transport
GO:0030001	3	1036	0.000824908	metal ion transport
GO:0050877	3	1063	0.000891164	neurological system process
GO:0098655	3	1072	0.000914016	cation transmembrane transport
GO:0048878	3	1094	0.000971504	chemical homeostasis
GO:0034220	3	1538	0.00270151	ion transmembrane transport
GO:0006812	3	1582	0.00294022	cation transport
GO:0003008	3	1588	0.00297383	system process
GO:0042592	3	1658	0.00338495	homeostatic process
GO:0055085	3	2352	0.00966816	transmembrane transport
GO:0006811	3	2423	0.0105708	ion transport
GO:0065008	3	3888	0.0436948	regulation of biological quality

Table 2: Overrepresented GO terms with the standard enrichment

2 Network-based enrichment

GO Term	N1	N2	P-value	Description
GO:1903362	3	339	0.000221413	regulation of cellular protein catabolic process
GO:0042176	3	816	0.00310404	regulation of protein catabolic process
GO:0019228	2	75	0.00317576	neuronal action potential
GO:0001508	2	182	0.0188093	action potential

Table 3: Overrepresented terms with the network-based enrichment. Only terms not detected with the standard method.
