# Supplementary material for: NET-GE: a novel NETwork-based Gene Enrichment for detecting biological processes associated to Mendelian diseases
Source: BMC Genomics. 2015 Jun 18;16(Suppl 8):S6. doi: 10.1186/1471-2164-16-S8-S6 (PMC4480278; doi:10.1186/1471-2164-16-S8-S6)
Supplement: Additional file 3 — Detailed results for the OMIM-derived benchmark set. The archive contains pdf documents listing the enriched terms for each one of the 244 diseases in the OMIM-derived benchmark set. [file 1471-2164-16-S8-S6-S3.tgz › SUPPMAT/OMIM264800.pdf]

#264800 PSEUDOXANTHOMA ELASTICUM; PXE

OMIM Gene ID	HGNC	UniProtAC
603234	ABCC6	O95255
608124	XYLT1	Q86Y38
608125	XYLT2	Q9H1B5

Table 1: OMIM - UniProtAC mapping

Legend

- N1: #input proteins associated to the significant GO term
- N2: #proteins associated to the significant GO term
- P-value: Bonferroni-corrected p-value of Fisher's exact test
- *red*: go terms not related to the input proteins
- *blue*: go terms related to the input proteins (enriched uniquely by network-based method)
- *green*: go terms ancestors of terms enriched with the standard method (enriched uniquely by network-based method)

1 Standard enrichment

GO Term	N1	N2	P-value	Description
GO:0015012	2	23	0.00013422	heparan sulfate proteoglycan biosynthetic process
GO:0030206	2	26	0.000172408	chondroitin sulfate biosynthetic process
GO:0030166	2	35	0.000315591	proteoglycan biosynthetic process
GO:0030201	2	38	0.000372854	heparan sulfate proteoglycan metabolic process
GO:0009101	2	61	0.000970194	glycoprotein biosynthetic process
GO:0006029	2	62	0.00100252	proteoglycan metabolic process
GO:0030204	2	73	0.00139297	chondroitin sulfate metabolic process
GO:0006024	2	113	0.00335178	glycosaminoglycan biosynthetic process
GO:0009100	2	113	0.00335178	glycoprotein metabolic process
GO:0006023	2	114	0.00341156	aminoglycan biosynthetic process
GO:0044272	2	186	0.00910126	sulfur compound biosynthetic process
GO:0030203	2	217	0.0123906	glycosaminoglycan metabolic process
GO:0006022	2	232	0.0141633	aminoglycan metabolic process
GO:1901135	3	2199	0.0248874	carbohydrate derivative metabolic process

Table 2: Overrepresented GO terms with the standard enrichment

2 Network-based enrichment

No novel enriched terms
