# Supplementary material for: NET-GE: a novel NETwork-based Gene Enrichment for detecting biological processes associated to Mendelian diseases
Source: BMC Genomics. 2015 Jun 18;16(Suppl 8):S6. doi: 10.1186/1471-2164-16-S8-S6 (PMC4480278; doi:10.1186/1471-2164-16-S8-S6)
Supplement: Additional file 3 — Detailed results for the OMIM-derived benchmark set. The archive contains pdf documents listing the enriched terms for each one of the 244 diseases in the OMIM-derived benchmark set. [file 1471-2164-16-S8-S6-S3.tgz › SUPPMAT/OMIM266600.pdf]

#266600 INFLAMMATORY BOWEL DISEASE 1; IBD1

OMIM Gene ID	HGNC	UniProtAC
147620	IL6	P05231
605956	NOD2	Q9HC29

Table 1: OMIM - UniProtAC mapping

Legend

- N1: #input proteins associated to the significant GO term
- N2: #proteins associated to the significant GO term
- P-value: Bonferroni-corrected p-value of Fisher's exact test
- *red*: go terms not related to the input proteins
- *blue*: go terms related to the input proteins (enriched uniquely by network-based method)
- *green*: go terms ancestors of terms enriched with the standard method (enriched uniquely by network-based method)

1 Standard enrichment

GO Term	N1	N2	P-value	Description
GO:0032494	2	14	9.72284e-05	response to peptidoglycan
GO:0002251	2	28	0.000403873	organ or tissue specific immune response
GO:0002828	2	54	0.00152895	regulation of type 2 immune response
GO:0032755	2	59	0.00182811	positive regulation of interleukin-6 production
GO:0002688	2	72	0.00273096	regulation of leukocyte chemotaxis
GO:0050830	2	78	0.00320855	defense response to Gram-positive bacterium
GO:0050871	2	87	0.00399706	positive regulation of B cell activation
GO:0002685	2	119	0.00750157	regulation of leukocyte migration
GO:0032675	2	126	0.008414	regulation of interleukin-6 production
GO:0050864	2	130	0.00895895	regulation of B cell activation
GO:0002700	2	137	0.00995365	regulation of production of molecular mediator of immune response
GO:0050920	2	141	0.0105456	regulation of chemotaxis
GO:0070374	2	150	0.0119399	positive regulation of ERK1 and ERK2 cascade
GO:0050707	2	156	0.0129175	regulation of cytokine secretion
GO:0050714	2	162	0.0139336	positive regulation of protein secretion
GO:0050679	2	188	0.0187812	positive regulation of epithelial cell proliferation
GO:0050731	2	193	0.0197962	positive regulation of peptidyl-tyrosine phosphorylation
GO:0071219	2	198	0.0208379	cellular response to molecule of bacterial origin
GO:0050795	2	205	0.0223412	regulation of behavior
GO:0071216	2	215	0.0245796	cellular response to biotic stimulus
GO:0070372	2	231	0.0283833	regulation of ERK1 and ERK2 cascade
GO:0042742	2	233	0.028878	defense response to bacterium
GO:0009617	2	261	0.0362524	response to bacterium
GO:0051091	2	264	0.0370922	positive regulation of sequence-specific DNA binding transcription factor activity
GO:0050730	2	266	0.0376575	regulation of peptidyl-tyrosine phosphorylation
GO:0050870	2	267	0.0379416	positive regulation of T cell activation
GO:0002699	2	269	0.0385133	positive regulation of immune effector process
GO:0050708	2	273	0.0396693	regulation of protein secretion
GO:0032498	1	1	0.0403254	detection of muramyl dipeptide
GO:0032499	1	1	0.0403254	detection of peptidoglycan

Table 2: Overrepresented GO terms with the standard enrichment

2 Network-based enrichment

GO Term	N1	N2	P-value	Description
GO:0052031	2	16	0.000410215	modulation by symbiont of host defense response
GO:0052255	2	16	0.000410215	modulation by organism of defense response of other organism involved in symbiotic interaction
GO:0052509	2	16	0.000410215	positive regulation by symbiont of host defense response
GO:0052510	2	16	0.000410215	positive regulation by organism of defense response of other organism involved in symbiotic interaction
GO:0052552	2	16	0.000410215	modulation by organism of immune response of other organism involved in symbiotic interaction
GO:0052553	2	16	0.000410215	modulation by symbiont of host immune response
GO:0002282	2	17	0.000464911	microglial cell activation involved in immune response
GO:0032740	2	17	0.000464911	positive regulation of interleukin-17 production
GO:0052564	2	23	0.000864871	response to immune response of other organism involved in symbiotic interaction
GO:0052572	2	23	0.000864871	response to host immune response
GO:0051770	2	24	0.000943496	positive regulation of nitric-oxide synthase biosynthetic process
GO:0052173	2	25	0.00102554	response to defenses of other organism involved in symbiotic interaction
GO:0052200	2	25	0.00102554	response to host defenses
GO:0075136	2	25	0.00102554	response to host
GO:0002862	2	26	0.001111	negative regulation of inflammatory response to antigenic stimulus
GO:0001774	2	30	0.00148703	microglial cell activation
GO:0051769	2	31	0.00158959	regulation of nitric-oxide synthase biosynthetic process
GO:0002281	2	38	0.00240318	macrophage activation involved in immune response
GO:0010669	2	40	0.0026664	epithelial structure maintenance
GO:0002227	2	43	0.00308687	innate immune response in mucosa
GO:0032660	2	43	0.00308687	regulation of interleukin-17 production
GO:0032733	2	51	0.00435855	positive regulation of interleukin-10 production
GO:0044126	2	51	0.00435855	regulation of growth of symbiont in host
GO:0044130	2	51	0.00435855	negative regulation of growth of symbiont in host
GO:0044144	2	51	0.00435855	modulation of growth of symbiont involved in interaction with host
GO:0044146	2	51	0.00435855	negative regulation of growth of symbiont involved in interaction with host
GO:0002861	2	54	0.00489181	regulation of inflammatory response to antigenic stimulus
GO:0090279	2	58	0.00565073	regulation of calcium ion import
GO:0045408	2	62	0.00646431	regulation of interleukin-6 biosynthetic process
GO:0090022	2	62	0.00646431	regulation of neutrophil chemotaxis
GO:1902622	2	63	0.00667626	regulation of neutrophil migration
GO:0042346	2	67	0.00755822	positive regulation of NF-kappaB import into nucleus
GO:0002385	2	68	0.00778726	mucosal immune response
GO:0002820	2	71	0.00849488	negative regulation of adaptive immune response
GO:1901222	2	75	0.00948623	regulation of NIK/NF-kappaB signaling
GO:0050663	2	77	0.0100024	cytokine secretion
GO:0042116	2	78	0.0102656	macrophage activation
GO:0071622	2	82	0.0113527	regulation of granulocyte chemotaxis
GO:0032735	2	84	0.0119168	positive regulation of interleukin-12 production
GO:0070206	2	87	0.0127885	protein trimerization
GO:0044003	2	89	0.0133867	modification by symbiont of host morphology or physiology
GO:0002275	2	96	0.0155882	myeloid cell activation involved in immune response
GO:0050710	2	100	0.0169214	negative regulation of cytokine secretion
GO:0051353	2	104	0.0183093	positive regulation of oxidoreductase activity
GO:0042345	2	105	0.0186648	regulation of NF-kappaB import into nucleus
GO:0042993	2	106	0.0190237	positive regulation of transcription factor import into nucleus
GO:0034121	2	107	0.019386	regulation of toll-like receptor signaling pathway
GO:0030593	2	113	0.0216321	neutrophil chemotaxis
GO:0032760	2	114	0.0220183	positive regulation of tumor necrosis factor production
GO:0032653	2	120	0.0244078	regulation of interleukin-10 production
GO:0007254	2	121	0.0248181	JNK cascade
GO:0034162	2	121	0.0248181	toll-like receptor 9 signaling pathway
GO:0042036	2	121	0.0248181	negative regulation of cytokine biosynthetic process
GO:1990266	2	123	0.0256488	neutrophil migration
GO:0031644	2	124	0.0260691	regulation of neurological system process
GO:0032663	2	132	0.029556	regulation of interleukin-2 production
GO:2000107	2	136	0.0313815	negative regulation of leukocyte apoptotic process
GO:0071621	2	142	0.0342223	granulocyte chemotaxis
GO:0008593	2	145	0.0356888	regulation of Notch signaling pathway
GO:0002755	2	146	0.0361843	MyD88-dependent toll-like receptor signaling pathway

Table 3: Overrepresented terms with the network-based enrichment. Only terms not detected with the standard method.

GO Term	N1	N2	P-value	Description
GO:0097530	2	154	0.0402729	granulocyte migration
GO:0022600	2	155	0.0407993	digestive system process
GO:0030838	2	162	0.0445801	positive regulation of actin filament polymerization
GO:0032655	2	166	0.0468159	regulation of interleukin-12 production
GO:0050709	2	170	0.0491061	negative regulation of protein secretion

Table 4: Overrepresented terms with the network-based enrichment. Only terms not detected with the standard method.
