# Supplementary material for: NET-GE: a novel NETwork-based Gene Enrichment for detecting biological processes associated to Mendelian diseases
Source: BMC Genomics. 2015 Jun 18;16(Suppl 8):S6. doi: 10.1186/1471-2164-16-S8-S6 (PMC4480278; doi:10.1186/1471-2164-16-S8-S6)
Supplement: Additional file 3 — Detailed results for the OMIM-derived benchmark set. The archive contains pdf documents listing the enriched terms for each one of the 244 diseases in the OMIM-derived benchmark set. [file 1471-2164-16-S8-S6-S3.tgz › SUPPMAT/OMIM267430.pdf]

#267430 RENAL TUBULAR DYSGENESIS; RTD

OMIM Gene ID	HGNC	UniProtAC
106150	AGT	P01019
106165	AGTR1	P30556
106180	ACE	P12821
179820	REN	P00797

Table 1: OMIM - UniProtAC mapping

Legend

- N1: #input proteins associated to the significant GO term
- N2: #proteins associated to the significant GO term
- P-value: Bonferroni-corrected p-value of Fisher's exact test
- *red*: go terms not related to the input proteins
- *blue*: go terms related to the input proteins (enriched uniquely by network-based method)
- *green*: go terms ancestors of terms enriched with the standard method (enriched uniquely by network-based method)

1 Standard enrichment

GO Term	N1	N2	P-value	Description
GO:0002018	3	4	9.83877e-10	renin-angiotensin regulation of aldosterone production
GO:0002003	3	13	7.03346e-08	angiotensin maturation
GO:0003081	3	16	1.3771e-07	regulation of systemic arterial blood pressure by renin-angiotensin
GO:0001822	4	181	2.81903e-07	kidney development
GO:0008217	4	197	3.96674e-07	regulation of blood pressure
GO:0003071	3	23	4.35446e-07	renal system process involved in regulation of systemic arterial blood pressure
GO:0001990	3	29	8.98323e-07	regulation of systemic arterial blood pressure by hormone
GO:0003044	3	29	8.98323e-07	regulation of systemic arterial blood pressure mediated by a chemical signal
GO:0050886	3	29	8.98323e-07	endocrine process
GO:0016486	3	43	3.03314e-06	peptide hormone processing
GO:0003073	3	52	5.43072e-06	regulation of systemic arterial blood pressure
GO:0019229	3	68	1.23113e-05	regulation of vasoconstriction
GO:0002019	2	3	1.39244e-05	regulation of renal output by angiotensin
GO:0033864	2	3	1.39244e-05	positive regulation of NAD(P)H oxidase activity
GO:0002034	2	4	2.78479e-05	regulation of blood vessel size by renin-angiotensin
GO:0003072	2	4	2.78479e-05	renal control of peripheral vascular resistance involved in regulation of systemic arterial blood pressure
GO:0003014	3	100	3.96972e-05	renal system process
GO:0033860	2	6	6.9615e-05	regulation of NAD(P)H oxidase activity
GO:0010873	2	7	9.7457e-05	positive regulation of cholesterol esterification
GO:0042756	2	8	0.000129938	drinking behavior
GO:0010872	2	9	0.000167058	regulation of cholesterol esterification
GO:0002016	2	10	0.000208815	regulation of blood volume by renin-angiotensin
GO:0034374	2	13	0.000361907	low-density lipoprotein particle remodeling
GO:0042445	3	231	0.000496524	hormone metabolic process
GO:0016485	3	301	0.00110031	protein processing
GO:0010817	3	337	0.00154475	regulation of hormone levels
GO:0051604	3	340	0.00158641	protein maturation
GO:0003008	4	1588	0.00172042	system process
GO:0045940	2	28	0.00175293	positive regulation of steroid metabolic process
GO:0035813	2	30	0.00201712	regulation of renal sodium excretion
GO:0034367	2	31	0.00215615	macromolecular complex remodeling
GO:0034368	2	31	0.00215615	protein-lipid complex remodeling
GO:0034369	2	31	0.00215615	plasma lipoprotein particle remodeling
GO:0044062	2	32	0.00229981	regulation of excretion
GO:0010744	2	37	0.00308751	positive regulation of macrophage derived foam cell differentiation
GO:0048513	4	1910	0.00360282	organ development
GO:0071827	2	40	0.00361562	plasma lipoprotein particle organization
GO:0051353	2	42	0.0039908	positive regulation of oxidoreductase activity
GO:0071825	2	42	0.0039908	protein-lipid complex subunit organization
GO:0042312	2	53	0.00638466	regulation of vasodilation
GO:0044057	3	554	0.00685692	regulation of system process
GO:0001974	2	55	0.00687995	blood vessel remodeling
GO:0010743	2	55	0.00687995	regulation of macrophage derived foam cell differentiation
GO:2000379	2	57	0.00739365	positive regulation of reactive oxygen species metabolic process
GO:0007200	2	60	0.00819888	phospholipase C-activating G-protein coupled receptor signaling pathway
GO:0050880	2	90	0.018532	regulation of blood vessel size
GO:0051341	2	90	0.018532	regulation of oxidoreductase activity
GO:0035150	2	91	0.0189478	regulation of tube size
GO:0010518	2	93	0.0197931	positive regulation of phospholipase activity
GO:0048771	2	103	0.0242956	tissue remodeling
GO:0010517	2	104	0.0247711	regulation of phospholipase activity
GO:0060193	2	108	0.0267191	positive regulation of lipase activity
GO:0050729	2	110	0.0277206	positive regulation of inflammatory response
GO:0003018	2	117	0.0313704	vascular process in circulatory system
GO:0019218	2	120	0.0330033	regulation of steroid metabolic process
GO:0007631	2	130	0.0387444	feeding behavior
GO:2000377	2	131	0.0393437	regulation of reactive oxygen species metabolic process
GO:1903036	2	135	0.0417866	positive regulation of response to wounding
GO:0045834	2	139	0.0443028	positive regulation of lipid metabolic process

Table 2: Overrepresented GO terms with the standard enrichment

2 Network-based enrichment

GO Term	N1	N2	P-value	Description
GO:0031532	3	110	0.000205303	actin cytoskeleton reorganization
GO:0050727	4	853	0.000627015	regulation of inflammatory response
GO:0015711	4	964	0.00102363	organic anion transport
GO:0050810	3	198	0.0012097	regulation of steroid biosynthetic process
GO:0009408	3	232	0.00194888	response to heat
GO:0032844	4	1155	0.0021116	regulation of homeostatic process
GO:0043255	3	259	0.00271358	regulation of carbohydrate biosynthetic process
GO:1903034	4	1274	0.00312731	regulation of response to wounding
GO:0034614	3	279	0.0033933	cellular response to reactive oxygen species
GO:0006518	3	280	0.00342998	peptide metabolic process
GO:0006820	4	1397	0.00452334	anion transport
GO:0043065	4	1406	0.00464117	positive regulation of apoptotic process
GO:0043068	4	1416	0.00477476	positive regulation of programmed cell death
GO:0010942	4	1542	0.00671718	positive regulation of cell death
GO:0050865	4	1568	0.00718228	regulation of cell activation
GO:0001823	2	31	0.0073771	mesonephros development
GO:0031347	4	1612	0.00802385	regulation of defense response
GO:0046890	3	401	0.0100802	regulation of lipid biosynthetic process
GO:0010893	2	37	0.0105634	positive regulation of steroid biosynthetic process
GO:0006112	3	412	0.0109322	energy reserve metabolic process
GO:0000187	3	421	0.0116638	activation of MAPK activity
GO:0042692	3	437	0.0130435	muscle cell differentiation
GO:0043408	4	1837	0.0135381	regulation of MAPK cascade
GO:0043603	3	447	0.0139586	cellular amide metabolic process
GO:0046903	4	1910	0.0158238	secretion
GO:0009266	3	469	0.0161196	response to temperature stimulus
GO:0034599	3	502	0.0197608	cellular response to oxidative stress
GO:0050731	3	503	0.0198789	positive regulation of peptidyl-tyrosine phosphorylation
GO:0010675	3	504	0.0199975	regulation of cellular carbohydrate metabolic process
GO:0000302	3	514	0.0212093	response to reactive oxygen species
GO:0043207	4	2065	0.0216251	response to external biotic stimulus
GO:0006109	3	523	0.0223407	regulation of carbohydrate metabolic process
GO:0051046	4	2094	0.0228666	regulation of secretion
GO:0015980	3	547	0.0255521	energy derivation by oxidation of organic compounds
GO:0032101	4	2173	0.0265204	regulation of response to external stimulus
GO:0090287	3	557	0.0269757	regulation of cellular response to growth factor stimulus
GO:0051240	4	2185	0.0271114	positive regulation of multicellular organismal process
GO:0009607	4	2197	0.0277124	response to biotic stimulus
GO:0045596	4	2226	0.0292059	negative regulation of cell differentiation
GO:0040008	4	2308	0.0337563	regulation of growth
GO:0001934	4	2371	0.0375982	positive regulation of protein phosphorylation
GO:0050974	2	72	0.0404837	detection of mechanical stimulus involved in sensory perception
GO:1902533	4	2418	0.0406713	positive regulation of intracellular signal transduction
GO:0009651	2	73	0.0416224	response to salt stress
GO:0030278	3	644	0.0416409	regulation of ossification
GO:0046942	3	650	0.0428117	carboxylic acid transport
GO:0015849	3	654	0.0436042	organic acid transport
GO:0009611	3	657	0.0442049	response to wounding
GO:0009612	3	658	0.0444063	response to mechanical stimulus
GO:0043406	3	661	0.0450143	positive regulation of MAP kinase activity
GO:0050730	3	663	0.0454227	regulation of peptidyl-tyrosine phosphorylation
GO:0043066	4	2487	0.0455193	negative regulation of apoptotic process
GO:0006816	3	668	0.0464543	calcium ion transport
GO:0043069	4	2511	0.0473031	negative regulation of programmed cell death

Table 3: Overrepresented terms with the network-based enrichment. Only terms not detected with the standard method.
