# Supplementary material for: NET-GE: a novel NETwork-based Gene Enrichment for detecting biological processes associated to Mendelian diseases
Source: BMC Genomics. 2015 Jun 18;16(Suppl 8):S6. doi: 10.1186/1471-2164-16-S8-S6 (PMC4480278; doi:10.1186/1471-2164-16-S8-S6)
Supplement: Additional file 3 — Detailed results for the OMIM-derived benchmark set. The archive contains pdf documents listing the enriched terms for each one of the 244 diseases in the OMIM-derived benchmark set. [file 1471-2164-16-S8-S6-S3.tgz › SUPPMAT/OMIM268220.pdf]

#268220 RHABDOMYOSARCOMA 2; RMS2

OMIM Gene ID	HGNC	UniProtAC
136533	FOXO1	Q12778
167410	PAX7	P23759
606597	PAX3	P23760

Table 1: OMIM - UniProtAC mapping

Legend

- N1: #input proteins associated to the significant GO term
- N2: #proteins associated to the significant GO term
- P-value: Bonferroni-corrected p-value of Fisher's exact test
- *red*: go terms not related to the input proteins
- *blue*: go terms related to the input proteins (enriched uniquely by network-based method)
- *green*: go terms ancestors of terms enriched with the standard method (enriched uniquely by network-based method)

1 Standard enrichment

GO Term	N1	N2	P-value	Description
GO:0021527	2	17	0.000195857	spinal cord association neuron differentiation
GO:0021515	2	57	0.00229682	cell differentiation in spinal cord
GO:0048663	2	58	0.0023788	neuron fate commitment
GO:0021953	2	135	0.0129988	central nervous system neuron differentiation
GO:0045944	3	1312	0.0143337	positive regulation of transcription from RNA polymerase II promoter
GO:0014813	1	1	0.0271838	skeletal muscle satellite cell commitment
GO:0045893	3	1762	0.03474	positive regulation of transcription, DNA-templated
GO:0045165	2	222	0.0351997	cell fate commitment
GO:1902680	3	1811	0.0377212	positive regulation of RNA biosynthetic process
GO:0051254	3	1838	0.0394347	positive regulation of RNA metabolic process
GO:0010628	3	1919	0.0448844	positive regulation of gene expression

Table 2: Overrepresented GO terms with the standard enrichment

2 Network-based enrichment

GO Term	N1	N2	P-value	Description
GO:1902692	3	122	5.92674e-05	regulation of neuroblast proliferation
GO:2000179	3	134	7.87082e-05	positive regulation of neural precursor cell proliferation
GO:0010001	3	205	0.000284034	glial cell differentiation
GO:2000648	3	237	0.000439762	positive regulation of stem cell proliferation
GO:0045638	3	261	0.000588035	negative regulation of myeloid cell differentiation
GO:0009953	3	264	0.000608626	dorsal/ventral pattern formation
GO:2000177	3	288	0.000790916	regulation of neural precursor cell proliferation
GO:0072091	3	327	0.00115915	regulation of stem cell proliferation
GO:0007179	3	364	0.00160032	transforming growth factor beta receptor signaling pathway
GO:0051054	3	411	0.00230589	positive regulation of DNA metabolic process
GO:0031099	3	421	0.00247876	regeneration
GO:0007623	3	457	0.00317236	circadian rhythm
GO:0043393	3	465	0.00334226	regulation of protein binding
GO:0016202	3	469	0.00342946	regulation of striated muscle tissue development
GO:1901861	3	471	0.00347361	regulation of muscle tissue development
GO:0048634	3	482	0.00372326	regulation of muscle organ development
GO:0050731	3	503	0.00423252	positive regulation of peptidyl-tyrosine phosphorylation
GO:0071560	3	511	0.00443811	cellular response to transforming growth factor beta stimulus
GO:0071559	3	521	0.00470434	response to transforming growth factor beta
GO:0048666	3	539	0.00520996	neuron development
GO:0090287	3	557	0.00575059	regulation of cellular response to growth factor stimulus
GO:0045637	3	596	0.00704756	regulation of myeloid cell differentiation
GO:0007178	3	618	0.00785857	transmembrane receptor protein serine/threonine kinase signaling pathway
GO:0001501	3	620	0.00793524	skeletal system development
GO:0050769	3	660	0.00957512	positive regulation of neurogenesis
GO:0050730	3	663	0.00970649	regulation of peptidyl-tyrosine phosphorylation
GO:0035295	3	664	0.00975054	tube development
GO:0048534	3	682	0.0105665	hematopoietic or lymphoid organ development
GO:0048736	2	59	0.011429	appendage development
GO:0060173	2	59	0.011429	limb development
GO:0048589	3	714	0.0121271	developmental growth
GO:0007420	3	791	0.0164956	brain development
GO:0002052	2	73	0.0175494	positive regulation of neuroblast proliferation
GO:0051098	3	826	0.0187867	regulation of binding
GO:0040036	2	82	0.0221731	regulation of fibroblast growth factor receptor signaling pathway
GO:0001701	3	893	0.0237456	in utero embryonic development
GO:0051052	3	905	0.0247169	regulation of DNA metabolic process
GO:0043009	3	909	0.0250464	chordate embryonic development
GO:0010453	2	89	0.0261421	regulation of cell fate commitment
GO:0050918	2	89	0.0261421	positive chemotaxis
GO:0002521	3	929	0.0267382	leukocyte differentiation
GO:0048511	3	930	0.0268247	rhythmic process
GO:0009792	3	937	0.0274357	embryo development ending in birth or egg hatching
GO:0001525	3	949	0.0285046	angiogenesis
GO:0042246	2	95	0.0298034	tissue regeneration
GO:0010720	3	977	0.0311056	positive regulation of cell development
GO:0003002	3	988	0.0321692	regionalization
GO:0016331	2	100	0.0330374	morphogenesis of embryonic epithelium
GO:0060429	3	1006	0.0339615	epithelium development
GO:0048536	2	102	0.0343776	spleen development
GO:0048732	3	1014	0.034779	gland development
GO:0046189	2	108	0.0385575	phenol-containing compound biosynthetic process
GO:0019221	3	1123	0.0472571	cytokine-mediated signaling pathway

Table 3: Overrepresented terms with the network-based enrichment. Only terms not detected with the standard method.
