# Supplementary material for: NET-GE: a novel NETwork-based Gene Enrichment for detecting biological processes associated to Mendelian diseases
Source: BMC Genomics. 2015 Jun 18;16(Suppl 8):S6. doi: 10.1186/1471-2164-16-S8-S6 (PMC4480278; doi:10.1186/1471-2164-16-S8-S6)
Supplement: Additional file 3 — Detailed results for the OMIM-derived benchmark set. The archive contains pdf documents listing the enriched terms for each one of the 244 diseases in the OMIM-derived benchmark set. [file 1471-2164-16-S8-S6-S3.tgz › SUPPMAT/OMIM269160.pdf]

#269160 SCHIZENCEPHALY

OMIM Gene ID	HGNC	UniProtAC
600035	EMX2	Q04743
600725	SHH	Q15465
603714	SIX3	O95343

Table 1: OMIM - UniProtAC mapping

Legend

- N1: #input proteins associated to the significant GO term
- N2: #proteins associated to the significant GO term
- P-value: Bonferroni-corrected p-value of Fisher's exact test
- *red*: go terms not related to the input proteins
- *blue*: go terms related to the input proteins (enriched uniquely by network-based method)
- *green*: go terms ancestors of terms enriched with the standard method (enriched uniquely by network-based method)

1 Standard enrichment

GO Term	N1	N2	P-value	Description
GO:0072001	2	18	0.000380111	renal system development
GO:0003002	3	380	0.000597434	regionalization
GO:0007389	3	579	0.00211914	pattern specification process
GO:0061351	2	96	0.0113132	neural precursor cell proliferation
GO:0009953	2	104	0.0132862	dorsal/ventral pattern formation
GO:0001654	2	128	0.0201539	eye development
GO:0007423	2	187	0.043077	sensory organ development
GO:0030178	2	193	0.0458884	negative regulation of Wnt signaling pathway
GO:0014858	1	1	0.0468961	positive regulation of skeletal muscle cell proliferation
GO:0060439	1	1	0.0468961	trachea morphogenesis
GO:0060458	1	1	0.0468961	right lung development
GO:0060459	1	1	0.0468961	left lung development
GO:0060516	1	1	0.0468961	primary prostatic bud elongation
GO:0060782	1	1	0.0468961	regulation of mesenchymal cell proliferation involved in prostate gland development
GO:0060783	1	1	0.0468961	mesenchymal smoothened signaling pathway involved in prostate gland development
GO:0061189	1	1	0.0468961	positive regulation of sclerotome development
GO:0061190	1	1	0.0468961	regulation of sclerotome development
GO:0080125	1	1	0.0468961	multicellular structure septum development
GO:2000061	1	1	0.0468961	regulation of ureter smooth muscle cell differentiation
GO:2000062	1	1	0.0468961	negative regulation of ureter smooth muscle cell differentiation
GO:2000063	1	1	0.0468961	positive regulation of ureter smooth muscle cell differentiation
GO:2000356	1	1	0.0468961	regulation of kidney smooth muscle cell differentiation
GO:2000357	1	1	0.0468961	negative regulation of kidney smooth muscle cell differentiation
GO:2000358	1	1	0.0468961	positive regulation of kidney smooth muscle cell differentiation

Table 2: Overrepresented GO terms with the standard enrichment

2 Network-based enrichment

GO Term	N1	N2	P-value	Description
GO:0048706	3	155	0.000132363	embryonic skeletal system development
GO:0021798	2	16	0.000869058	forebrain dorsal/ventral pattern formation
GO:0021796	2	25	0.00217226	cerebral cortex regionalization
GO:0021846	2	43	0.00653613	cell proliferation in forebrain
GO:0009952	3	585	0.00721874	anterior/posterior pattern specification
GO:0048714	2	47	0.00782391	positive regulation of oligodendrocyte differentiation
GO:0001501	3	620	0.00859598	skeletal system development
GO:0060038	2	54	0.0103556	cardiac muscle cell proliferation
GO:0014855	2	58	0.0119612	striated muscle cell proliferation
GO:0033002	2	67	0.0159961	muscle cell proliferation
GO:0042474	2	68	0.0164804	middle ear morphogenesis
GO:0002066	2	70	0.0174709	columnar/cuboidal epithelial cell development
GO:0040036	2	82	0.0240194	regulation of fibroblast growth factor receptor signaling pathway
GO:0031128	2	86	0.026433	developmental induction
GO:0045686	2	86	0.026433	negative regulation of glial cell differentiation
GO:0045168	2	90	0.0289618	cell-cell signaling involved in cell fate commitment
GO:0045687	2	92	0.0302695	positive regulation of glial cell differentiation
GO:0048858	3	963	0.0322663	cell projection morphogenesis
GO:0031018	2	96	0.0329714	endocrine pancreas development
GO:0010470	2	108	0.0417681	regulation of gastrulation
GO:0032990	3	1056	0.042558	cell part morphogenesis
GO:0048713	2	111	0.0441293	regulation of oligodendrocyte differentiation
GO:0014014	2	116	0.0482085	negative regulation of gliogenesis

Table 3: Overrepresented terms with the network-based enrichment. Only terms not detected with the standard method.
