# Supplementary material for: NET-GE: a novel NETwork-based Gene Enrichment for detecting biological processes associated to Mendelian diseases
Source: BMC Genomics. 2015 Jun 18;16(Suppl 8):S6. doi: 10.1186/1471-2164-16-S8-S6 (PMC4480278; doi:10.1186/1471-2164-16-S8-S6)
Supplement: Additional file 3 — Detailed results for the OMIM-derived benchmark set. The archive contains pdf documents listing the enriched terms for each one of the 244 diseases in the OMIM-derived benchmark set. [file 1471-2164-16-S8-S6-S3.tgz › SUPPMAT/OMIM275210.pdf]

#275210 RESTRICTIVE DERMOPATHY, LETHAL

OMIM Gene ID	HGNC	UniProtAC
150330	LMNA	P02545
606480	ZMPSTE24	O75844

Table 1: OMIM - UniProtAC mapping

Legend

- N1: #input proteins associated to the significant GO term
- N2: #proteins associated to the significant GO term
- P-value: Bonferroni-corrected p-value of Fisher's exact test
- *red*: go terms not related to the input proteins
- *blue*: go terms related to the input proteins (enriched uniquely by network-based method)
- *green*: go terms ancestors of terms enriched with the standard method (enriched uniquely by network-based method)

1 Standard enrichment

GO Term	N1	N2	P-value	Description
GO:0006998	2	60	0.000487078	nuclear envelope organization
GO:0035105	1	1	0.010386	sterol regulatory element binding protein import into nucleus
GO:0030327	1	2	0.0207719	prenylated protein catabolic process
GO:0030951	1	3	0.0311573	establishment or maintenance of microtubule cytoskeleton polarity
GO:0071586	1	3	0.0311573	CAAX-box protein processing
GO:0044802	2	572	0.0449393	single-organism membrane organization

Table 2: Overrepresented GO terms with the standard enrichment

2 Network-based enrichment

No novel enriched terms
