# Supplementary material for: NET-GE: a novel NETwork-based Gene Enrichment for detecting biological processes associated to Mendelian diseases
Source: BMC Genomics. 2015 Jun 18;16(Suppl 8):S6. doi: 10.1186/1471-2164-16-S8-S6 (PMC4480278; doi:10.1186/1471-2164-16-S8-S6)
Supplement: Additional file 3 — Detailed results for the OMIM-derived benchmark set. The archive contains pdf documents listing the enriched terms for each one of the 244 diseases in the OMIM-derived benchmark set. [file 1471-2164-16-S8-S6-S3.tgz › SUPPMAT/OMIM276300.pdf]

#276300 MISMATCH REPAIR CANCER SYNDROME; MMRCS

**OMIM Gene ID**	**HGNC**	**UniProtAC**
120436	MLH1	P40692
600259	PMS2	P54278
600678	MSH6	P52701
609309	MSH2	P43246

Table 1: OMIM - UniProtAC mapping

Legend

- N1: #input proteins associated to the significant GO term
- N2: #proteins associated to the significant GO term
- P-value: Bonferroni-corrected p-value of Fisher's exact test
- *red*: go terms not related to the input proteins
- *blue*: go terms related to the input proteins (enriched uniquely by network-based method)
- *green*: go terms ancestors of terms enriched with the standard method (enriched uniquely by network-based method)

1 Standard enrichment

GO Term	N1	N2	P-value	Description
GO:0002566	4	18	1.05329e-11	somatic diversification of immune receptors via somatic mutation
GO:0016446	4	18	1.05329e-11	somatic hypermutation of immunoglobulin genes
GO:0016447	4	44	4.67273e-10	somatic recombination of immunoglobulin gene segments
GO:0016445	4	47	6.13955e-10	somatic diversification of immunoglobulins
GO:0002562	4	63	2.05036e-09	somatic diversification of immune receptors via germline recombination within a single locus
GO:0016444	4	63	2.05036e-09	somatic cell DNA recombination
GO:0002200	4	66	2.48081e-09	somatic diversification of immune receptors
GO:0006298	4	66	2.48081e-09	mismatch repair
GO:0045910	3	28	4.25361e-07	negative regulation of DNA recombination
GO:0002204	3	32	6.43963e-07	somatic recombination of immunoglobulin genes involved in immune response
GO:0002208	3	32	6.43963e-07	somatic diversification of immunoglobulins involved in immune response
GO:0045190	3	32	6.43963e-07	isotype switching
GO:0006310	4	294	1.0498e-06	DNA recombination
GO:0002312	3	51	2.70271e-06	B cell activation involved in immune response
GO:0000710	2	3	7.35392e-06	meiotic mismatch repair
GO:0000018	3	79	1.02573e-05	regulation of DNA recombination
GO:0006200	4	569	1.48757e-05	ATP catabolic process
GO:0009158	4	574	1.54069e-05	ribonucleoside monophosphate catabolic process
GO:0009169	4	574	1.54069e-05	purine ribonucleoside monophosphate catabolic process
GO:0009128	4	575	1.55148e-05	purine nucleoside monophosphate catabolic process
GO:0009125	4	578	1.5842e-05	nucleoside monophosphate catabolic process
GO:0046034	4	656	2.6318e-05	ATP metabolic process
GO:0051053	3	108	2.64658e-05	negative regulation of DNA metabolic process
GO:0009167	4	698	3.37519e-05	purine ribonucleoside monophosphate metabolic process
GO:0009126	4	699	3.39463e-05	purine nucleoside monophosphate metabolic process
GO:0051096	2	6	3.67658e-05	positive regulation of helicase activity
GO:0002285	3	121	3.73225e-05	lymphocyte activation involved in immune response
GO:0009161	4	727	3.9734e-05	ribonucleoside monophosphate metabolic process
GO:0008630	3	126	4.2181e-05	intrinsic apoptotic signaling pathway in response to DNA damage
GO:0009123	4	745	4.38266e-05	nucleoside monophosphate metabolic process
GO:0006281	4	781	5.29515e-05	DNA repair
GO:0051095	2	9	8.82283e-05	regulation of helicase activity
GO:1903046	3	166	9.69405e-05	meiotic cell cycle process
GO:0002263	3	170	0.000104155	cell activation involved in immune response
GO:0002366	3	170	0.000104155	leukocyte activation involved in immune response
GO:0042113	3	186	0.000136583	B cell activation
GO:0009203	4	1015	0.000151325	ribonucleoside triphosphate catabolic process
GO:0009207	4	1015	0.000151325	purine ribonucleoside triphosphate catabolic process
GO:0009146	4	1019	0.000153728	purine nucleoside triphosphate catabolic process
GO:0009143	4	1024	0.000156772	nucleoside triphosphate catabolic process
GO:0006152	4	1028	0.00015924	purine nucleoside catabolic process
GO:0046130	4	1028	0.00015924	purine ribonucleoside catabolic process
GO:0042454	4	1038	0.000165536	ribonucleoside catabolic process
GO:0009154	4	1045	0.000170053	purine ribonucleotide catabolic process
GO:0009261	4	1046	0.000170706	ribonucleotide catabolic process
GO:0009164	4	1050	0.000173336	nucleoside catabolic process
GO:1901658	4	1057	0.000178012	glycosyl compound catabolic process
GO:0006195	4	1060	0.000180044	purine nucleotide catabolic process
GO:0072523	4	1071	0.000187646	purine-containing compound catabolic process
GO:0009166	4	1110	0.00021655	nucleotide catabolic process
GO:1901292	4	1118	0.000222869	nucleoside phosphate catabolic process
GO:0006974	4	1132	0.00023426	cellular response to DNA damage stimulus
GO:0009205	4	1139	0.000240116	purine ribonucleoside triphosphate metabolic process
GO:0009144	4	1146	0.000246081	purine nucleoside triphosphate metabolic process
GO:0009199	4	1146	0.000246081	ribonucleoside triphosphate metabolic process
GO:0046434	4	1175	0.000271987	organophosphate catabolic process
GO:0009141	4	1177	0.000273846	nucleoside triphosphate metabolic process
GO:0097193	3	246	0.000316858	intrinsic apoptotic signaling pathway
GO:0046128	4	1238	0.000335267	purine ribonucleoside metabolic process
GO:1901136	4	1238	0.000335267	carbohydrate derivative catabolic process

Table 2: Overrepresented GO terms with the standard enrichment

GO Term	N1	N2	P-value	Description
GO:0042278	4	1241	0.000338535	purine nucleoside metabolic process
GO:0009119	4	1278	0.000380803	ribonucleoside metabolic process
GO:0009150	4	1297	0.000403987	purine ribonucleotide metabolic process
GO:0009116	4	1319	0.000432135	nucleoside metabolic process
GO:0009259	4	1320	0.000433447	ribonucleotide metabolic process
GO:0019693	4	1325	0.000440059	ribose phosphate metabolic process
GO:0006163	4	1334	0.000452153	purine nucleotide metabolic process
GO:1901657	4	1339	0.000458977	glycosyl compound metabolic process
GO:0072521	4	1396	0.000542363	purine-containing compound metabolic process
GO:1901565	4	1431	0.000598898	organonitrogen compound catabolic process
GO:0006259	4	1502	0.000727043	DNA metabolic process
GO:0008340	2	25	0.000734822	determination of adult lifespan
GO:0034655	4	1517	0.000756553	nucleobase-containing compound catabolic process
GO:0044270	4	1582	0.000894941	cellular nitrogen compound catabolic process
GO:0046700	4	1586	0.000904035	heterocycle catabolic process
GO:0019439	4	1589	0.0009109	aromatic compound catabolic process
GO:1901361	4	1634	0.00101866	organic cyclic compound catabolic process
GO:0051052	3	374	0.00111529	regulation of DNA metabolic process
GO:0009117	4	1702	0.00119929	nucleotide metabolic process
GO:0006753	4	1724	0.00126256	nucleoside phosphate metabolic process
GO:0055086	4	1817	0.00155812	nucleobase-containing small molecule metabolic process
GO:0097190	3	439	0.00180351	apoptotic signaling pathway
GO:0033554	4	1930	0.00198378	cellular response to stress
GO:0046649	3	484	0.00241627	lymphocyte activation
GO:0044712	4	2063	0.0025903	single-organism catabolic process
GO:1901135	4	2199	0.00334449	carbohydrate derivative metabolic process
GO:0045321	3	579	0.00413296	leukocyte activation
GO:0019637	4	2393	0.00469136	organophosphate metabolic process
GO:0002376	4	2446	0.00512125	immune system process
GO:0002252	3	657	0.00603263	immune effector process
GO:0044248	4	2821	0.00906369	cellular catabolic process
GO:0001775	3	825	0.0119153	cell activation
GO:1901575	4	3074	0.0127816	organic substance catabolic process
GO:1901564	4	3152	0.0141297	organonitrogen compound metabolic process
GO:0009056	4	3347	0.0179664	catabolic process
GO:0045128	1	1	0.0308402	negative regulation of reciprocal meiotic recombination
GO:0006302	2	175	0.0370946	double-strand break repair
GO:0009411	2	177	0.0379473	response to UV
GO:0006950	4	4134	0.041828	response to stress

Table 3: Overrepresented GO terms with the standard enrichment

2 Network-based enrichment

GO Term	N1	N2	P-value	Description
GO:0000712	2	28	0.0020863	resolution of meiotic recombination intermediates
GO:0051307	2	30	0.0024007	meiotic chromosome separation
GO:0007129	2	33	0.0029136	synapsis
GO:0051304	2	46	0.00570833	chromosome separation
GO:0007131	2	66	0.0118209	reciprocal meiotic recombination
GO:0035825	2	66	0.0118209	reciprocal DNA recombination
GO:0022402	4	2771	0.0244087	cell cycle process
GO:0070192	2	101	0.027791	chromosome organization involved in meiosis
GO:0007292	2	105	0.0300425	female gamete generation
GO:0000280	3	969	0.0490542	nuclear division

Table 4: Overrepresented terms with the network-based enrichment. Only terms not detected with the standard method.
