# Supplementary material for: NET-GE: a novel NETwork-based Gene Enrichment for detecting biological processes associated to Mendelian diseases
Source: BMC Genomics. 2015 Jun 18;16(Suppl 8):S6. doi: 10.1186/1471-2164-16-S8-S6 (PMC4480278; doi:10.1186/1471-2164-16-S8-S6)
Supplement: Additional file 3 — Detailed results for the OMIM-derived benchmark set. The archive contains pdf documents listing the enriched terms for each one of the 244 diseases in the OMIM-derived benchmark set. [file 1471-2164-16-S8-S6-S3.tgz › SUPPMAT/OMIM303700.pdf]

#303700 BLUE CONE MONOCHROMACY; BCM

OMIM Gene ID	HGNC	UniProtAC
300821	OPN1MW	P04001
300822	OPN1LW	P04000

Table 1: OMIM - UniProtAC mapping

Legend

- N1: #input proteins associated to the significant GO term
- N2: #proteins associated to the significant GO term
- P-value: Bonferroni-corrected p-value of Fisher's exact test
- *red*: go terms not related to the input proteins
- *blue*: go terms related to the input proteins (enriched uniquely by network-based method)
- *green*: go terms ancestors of terms enriched with the standard method (enriched uniquely by network-based method)

1 Standard enrichment

GO Term	N1	N2	P-value	Description
GO:0018298	2	16	9.7719e-06	protein-chromophore linkage
GO:0032467	2	43	7.35336e-05	positive regulation of cytokinesis
GO:0032465	2	65	0.000169379	regulation of cytokinesis
GO:0007603	2	89	0.000318889	phototransduction, visible light
GO:0001523	2	96	0.000371331	retinoid metabolic process
GO:0016101	2	105	0.00044462	diterpenoid metabolic process
GO:0009584	2	106	0.000453171	detection of visible light
GO:0051781	2	110	0.000488187	positive regulation of cell division
GO:0007602	2	112	0.000506183	phototransduction
GO:0006721	2	119	0.000571736	terpenoid metabolic process
GO:0009583	2	129	0.000672307	detection of light stimulus
GO:0006720	2	155	0.000971894	isoprenoid metabolic process
GO:0007601	2	214	0.00185592	visual perception
GO:0050953	2	218	0.00192612	sensory perception of light stimulus
GO:0009581	2	221	0.00197962	detection of external stimulus
GO:0009582	2	224	0.00203385	detection of abiotic stimulus
GO:0090068	2	276	0.00309036	positive regulation of cell cycle process
GO:0051302	2	316	0.00405288	regulation of cell division
GO:0009416	2	424	0.00730255	response to light stimulus
GO:0007600	2	586	0.0139579	sensory perception
GO:0009314	2	591	0.0141973	response to radiation
GO:0010564	2	662	0.0178167	regulation of cell cycle process
GO:0051606	2	761	0.0235486	detection of stimulus
GO:0050877	2	1063	0.0459647	neurological system process

Table 2: Overrepresented GO terms with the standard enrichment

2 Network-based enrichment

No novel enriched terms
