# Supplementary material for: NET-GE: a novel NETwork-based Gene Enrichment for detecting biological processes associated to Mendelian diseases
Source: BMC Genomics. 2015 Jun 18;16(Suppl 8):S6. doi: 10.1186/1471-2164-16-S8-S6 (PMC4480278; doi:10.1186/1471-2164-16-S8-S6)
Supplement: Additional file 3 — Detailed results for the OMIM-derived benchmark set. The archive contains pdf documents listing the enriched terms for each one of the 244 diseases in the OMIM-derived benchmark set. [file 1471-2164-16-S8-S6-S3.tgz › SUPPMAT/OMIM308800.pdf]

#308800 KERATOSIS FOLLICULARIS SPINULOSA DECALVANS, X-LINKED; KFSDX

OMIM Gene ID	HGNC	UniProtAC
300294	MBTPS2	O43462
313020	SAT1	P21673

Table 1: OMIM - UniProtAC mapping

Legend

- N1: #input proteins associated to the significant GO term
- N2: #proteins associated to the significant GO term
- P-value: Bonferroni-corrected p-value of Fisher's exact test
- *red*: go terms not related to the input proteins
- *blue*: go terms related to the input proteins (enriched uniquely by network-based method)
- *green*: go terms ancestors of terms enriched with the standard method (enriched uniquely by network-based method)

1 Standard enrichment

GO Term	N1	N2	P-value	Description
GO:0032917	1	2	0.0117637	polyamine acetylation
GO:0032918	1	2	0.0117637	spermidine acetylation
GO:0009447	1	3	0.0176452	putrescine catabolic process
GO:0009445	1	5	0.0294079	putrescine metabolic process
GO:0006598	1	6	0.035289	polyamine catabolic process

Table 2: Overrepresented GO terms with the standard enrichment

2 Network-based enrichment

GO Term	N1	N2	P-value	Description
GO:0008215	1	12	0.046818	spermine metabolic process

Table 3: Overrepresented terms with the network-based enrichment. Only terms not detected with the standard method.
