# Supplementary material for: NET-GE: a novel NETwork-based Gene Enrichment for detecting biological processes associated to Mendelian diseases
Source: BMC Genomics. 2015 Jun 18;16(Suppl 8):S6. doi: 10.1186/1471-2164-16-S8-S6 (PMC4480278; doi:10.1186/1471-2164-16-S8-S6)
Supplement: Additional file 3 — Detailed results for the OMIM-derived benchmark set. The archive contains pdf documents listing the enriched terms for each one of the 244 diseases in the OMIM-derived benchmark set. [file 1471-2164-16-S8-S6-S3.tgz › SUPPMAT/OMIM600791.pdf]

#600791 DEAFNESS, AUTOSOMAL RECESSIVE 4, WITH ENLARGED VESTIBULAR AQUEDUCT;

**OMIM Gene ID**	**HGNC**	**UniProtAC**
601093	FOXI1	Q12951
602208	KCNJ10	P78508
605646	SLC26A4	O43511

Table 1: OMIM - UniProtAC mapping

Legend

- N1: #input proteins associated to the significant GO term
- N2: #proteins associated to the significant GO term
- P-value: Bonferroni-corrected p-value of Fisher's exact test
- *red*: go terms not related to the input proteins
- *blue*: go terms related to the input proteins (enriched uniquely by network-based method)
- *green*: go terms ancestors of terms enriched with the standard method (enriched uniquely by network-based method)

1 Standard enrichment

GO Term	N1	N2	P-value	Description
GO:0055067	2	104	0.00459386	monovalent inorganic cation homeostasis
GO:0051933	1	2	0.0324291	amino acid uptake involved in synaptic transmission
GO:0051935	1	2	0.0324291	L-glutamate uptake involved in synaptic transmission
GO:1902837	1	2	0.0324291	amino acid import into cell
GO:1990123	1	2	0.0324291	L-glutamate import into cell
GO:0009637	1	3	0.0486422	response to blue light

Table 2: Overrepresented GO terms with the standard enrichment

2 Network-based enrichment

No novel enriched terms
