# Supplementary material for: NET-GE: a novel NETwork-based Gene Enrichment for detecting biological processes associated to Mendelian diseases
Source: BMC Genomics. 2015 Jun 18;16(Suppl 8):S6. doi: 10.1186/1471-2164-16-S8-S6 (PMC4480278; doi:10.1186/1471-2164-16-S8-S6)
Supplement: Additional file 3 — Detailed results for the OMIM-derived benchmark set. The archive contains pdf documents listing the enriched terms for each one of the 244 diseases in the OMIM-derived benchmark set. [file 1471-2164-16-S8-S6-S3.tgz › SUPPMAT/OMIM600794.pdf]

#600794 NEURONOPATHY, DISTAL HEREDITARY MOTOR, TYPE VA; HMN5A

OMIM Gene ID	HGNC	UniProtAC
600287	GARS	P41250
606158	BSCL2	Q96G97

Table 1: OMIM - UniProtAC mapping

Legend

- N1: #input proteins associated to the significant GO term
- N2: #proteins associated to the significant GO term
- P-value: Bonferroni-corrected p-value of Fisher's exact test
- *red*: go terms not related to the input proteins
- *blue*: go terms related to the input proteins (enriched uniquely by network-based method)
- *green*: go terms ancestors of terms enriched with the standard method (enriched uniquely by network-based method)

1 Standard enrichment

GO Term	N1	N2	P-value	Description
GO:0006426	1	1	0.00455714	glycyl-tRNA aminoacylation
GO:0015960	1	4	0.0182278	diadenosine polyphosphate biosynthetic process
GO:0015965	1	4	0.0182278	diadenosine tetraphosphate metabolic process
GO:0015966	1	4	0.0182278	diadenosine tetraphosphate biosynthetic process
GO:0015959	1	5	0.0227845	diadenosine polyphosphate metabolic process

Table 2: Overrepresented GO terms with the standard enrichment

2 Network-based enrichment

No novel enriched terms
