# Supplementary material for: NET-GE: a novel NETwork-based Gene Enrichment for detecting biological processes associated to Mendelian diseases
Source: BMC Genomics. 2015 Jun 18;16(Suppl 8):S6. doi: 10.1186/1471-2164-16-S8-S6 (PMC4480278; doi:10.1186/1471-2164-16-S8-S6)
Supplement: Additional file 3 — Detailed results for the OMIM-derived benchmark set. The archive contains pdf documents listing the enriched terms for each one of the 244 diseases in the OMIM-derived benchmark set. [file 1471-2164-16-S8-S6-S3.tgz › SUPPMAT/OMIM600807.pdf]

#600807 ASTHMA, SUSCEPTIBILITY TO

OMIM Gene ID	HGNC	UniProtAC
109690	ADRB2	P07550
142871	HLA-G	P17693
147683	IL13	P35225
152390	ALOX5	P09917
158375	MUC7	Q8TAX7
161561	IL12B	P29460
191160	TNF	P01375
192020	SCGB1A1	P11684
601156	CCL11	P51671
601690	PLA2G7	Q13093
605238	HNMT	P50135
606531	SCGB3A2	Q96PL1
607796	PHF11	Q9UIL8

Table 1: OMIM - UniProtAC mapping

Legend

- N1: #input proteins associated to the significant GO term
- N2: #proteins associated to the significant GO term
- P-value: Bonferroni-corrected p-value of Fisher's exact test
- *red*: go terms not related to the input proteins
- *blue*: go terms related to the input proteins (enriched uniquely by network-based method)
- *green*: go terms ancestors of terms enriched with the standard method (enriched uniquely by network-based method)

1 Standard enrichment

GO Term	N1	N2	P-value	Description
GO:0034097	7	880	2.96973e-06	response to cytokine
GO:0009607	6	964	0.000251334	response to biotic stimulus
GO:0051241	5	547	0.000518872	negative regulation of multicellular organismal process
GO:0001818	4	215	0.000555042	negative regulation of cytokine production
GO:0050670	4	238	0.000832461	regulation of lymphocyte proliferation
GO:0032944	4	241	0.000875065	regulation of mononuclear cell proliferation
GO:0070663	4	248	0.000980775	regulation of leukocyte proliferation
GO:0002699	4	269	0.00135534	positive regulation of immune effector process
GO:0051094	6	1326	0.00162543	positive regulation of developmental process
GO:0001817	5	702	0.00177029	regulation of cytokine production
GO:0009628	6	1467	0.00292533	response to abiotic stimulus
GO:0050727	4	372	0.00490105	regulation of inflammatory response
GO:0051240	5	866	0.00494263	positive regulation of multicellular organismal process
GO:0001819	4	374	0.00500603	positive regulation of cytokine production
GO:0032649	3	105	0.00512952	regulation of interferon-gamma production
GO:0032496	4	389	0.00584743	response to lipopolysaccharide
GO:0050729	3	110	0.00590025	positive regulation of inflammatory response
GO:0043207	5	921	0.00667136	response to external biotic stimulus
GO:0002237	4	414	0.0074769	response to molecule of bacterial origin
GO:0006950	8	4134	0.00769145	response to stress
GO:0002544	2	13	0.00818765	chronic inflammatory response
GO:0002682	6	1758	0.00832992	regulation of immune system process
GO:0048660	3	127	0.00908636	regulation of smooth muscle cell proliferation
GO:1903036	3	135	0.0109137	positive regulation of response to wounding
GO:0002697	4	463	0.0116167	regulation of immune effector process
GO:0032501	8	4447	0.013354	multicellular organismal process
GO:0002684	5	1093	0.0153109	positive regulation of immune system process
GO:0002824	3	157	0.0171526	positive regulation of adaptive immune response based on somatic recombination of immune receptors built [illegible]
GO:0051249	4	513	0.0173807	regulation of lymphocyte activation
GO:0042127	6	2008	0.0178727	regulation of cell proliferation
GO:0002821	3	160	0.0181515	positive regulation of adaptive immune response
GO:0002708	3	161	0.0184929	positive regulation of lymphocyte mediated immunity
GO:0033993	5	1142	0.0189253	response to lipid
GO:0002705	3	163	0.0191882	positive regulation of leukocyte mediated immunity
GO:1903034	4	529	0.019606	regulation of response to wounding
GO:1902107	3	170	0.0217574	positive regulation of leukocyte differentiation
GO:0042129	3	181	0.0262367	regulation of T cell proliferation
GO:0002694	4	584	0.0288801	regulation of leukocyte activation
GO:0043279	3	188	0.0293814	response to alkaloid
GO:0051239	7	3432	0.0304163	regulation of multicellular organismal process
GO:0042346	2	25	0.0314243	positive regulation of NF-kappaB import into nucleus
GO:2000026	6	2229	0.0324287	regulation of multicellular organismal development
GO:0010033	7	3487	0.0337667	response to organic substance
GO:0002861	2	27	0.0367535	regulation of inflammatory response to antigenic stimulus
GO:0050865	4	624	0.0374111	regulation of cell activation
GO:0048584	6	2308	0.0395261	positive regulation of response to stimulus
GO:0045672	2	29	0.0424975	positive regulation of osteoclast differentiation
GO:0051384	3	215	0.0438219	response to glucocorticoid
GO:0002706	3	222	0.0482039	regulation of lymphocyte mediated immunity

Table 2: Overrepresented GO terms with the standard enrichment

2 Network-based enrichment

GO Term	N1	N2	P-value	Description
GO:0001894	6	489	1.82048e-05	tissue homeostasis
GO:0031347	8	1612	2.68857e-05	regulation of defense response
GO:0002532	3	21	0.000103564	production of molecular mediator involved in inflammatory response
GO:0080134	9	3072	0.000177263	regulation of response to stress
GO:0048661	4	137	0.00028904	positive regulation of smooth muscle cell proliferation
GO:0060249	6	783	0.000296434	anatomical structure homeostasis
GO:0051251	6	874	0.00056634	positive regulation of lymphocyte activation
GO:0050863	6	912	0.000727258	regulation of T cell activation
GO:0002696	6	941	0.00087396	positive regulation of leukocyte activation
GO:2000021	5	471	0.000883367	regulation of ion homeostasis
GO:0050867	6	996	0.00121933	positive regulation of cell activation
GO:0030335	6	1024	0.00143425	positive regulation of cell migration
GO:2000147	6	1042	0.0015882	positive regulation of cell motility
GO:0051272	6	1060	0.00175551	positive regulation of cellular component movement
GO:0008284	8	2808	0.00200554	positive regulation of cell proliferation
GO:0022603	8	2832	0.0021411	regulation of anatomical structure morphogenesis
GO:0019221	6	1123	0.00245958	cytokine-mediated signaling pathway
GO:0040017	6	1124	0.00247239	positive regulation of locomotion
GO:0030334	7	1926	0.00286078	regulation of cell migration
GO:0050795	5	630	0.00369698	regulation of behavior
GO:0016192	8	3055	0.00382928	vesicle-mediated transport
GO:0050900	5	635	0.00384312	leukocyte migration
GO:0006955	8	3063	0.00390671	immune response
GO:2000145	7	2039	0.00420049	regulation of cell motility
GO:0030595	4	281	0.00508995	leukocyte chemotaxis
GO:0051270	7	2182	0.00662304	regulation of cellular component movement
GO:0032735	3	84	0.00732534	positive regulation of interleukin-12 production
GO:0031349	5	726	0.00740182	positive regulation of defense response
GO:0040012	7	2224	0.007526	regulation of locomotion
GO:0032103	5	736	0.00791324	positive regulation of response to external stimulus
GO:0050921	4	324	0.00894752	positive regulation of chemotaxis
GO:0072503	5	772	0.00998957	cellular divalent inorganic cation homeostasis
GO:1901342	5	774	0.0101164	regulation of vasculature development
GO:0008285	7	2354	0.0110062	negative regulation of cell proliferation
GO:0055074	5	790	0.0111772	calcium ion homeostasis
GO:0050778	6	1484	0.0124065	positive regulation of immune response
GO:0042592	8	3571	0.0125832	homeostatic process
GO:1902533	7	2418	0.0131648	positive regulation of intracellular signal transduction
GO:0072507	5	827	0.0139672	divalent inorganic cation homeostasis
GO:0032713	2	11	0.0142933	negative regulation of interleukin-4 production
GO:0032714	2	11	0.0142933	negative regulation of interleukin-5 production
GO:0042531	3	106	0.0147655	positive regulation of tyrosine phosphorylation of STAT protein
GO:0045580	4	369	0.0149568	regulation of T cell differentiation
GO:0045597	7	2514	0.0170645	positive regulation of cell differentiation
GO:0048520	4	383	0.0173226	positive regulation of behavior
GO:0032729	3	114	0.0183745	positive regulation of interferon-gamma production
GO:0048585	8	3759	0.0185611	negative regulation of response to stimulus
GO:0001916	3	117	0.0198651	positive regulation of T cell mediated cytotoxicity
GO:0010647	8	3801	0.0201879	positive regulation of cell communication
GO:0007162	4	404	0.0213764	negative regulation of cell adhesion
GO:0071345	6	1640	0.0220638	cellular response to cytokine stimulus
GO:0045471	4	417	0.0242138	response to ethanol
GO:0001914	3	127	0.0254068	regulation of T cell mediated cytotoxicity
GO:0002683	5	945	0.0266846	negative regulation of immune system process
GO:0042509	3	130	0.0272487	regulation of tyrosine phosphorylation of STAT protein
GO:0032855	3	132	0.0285244	positive regulation of Rac GTPase activity
GO:0033632	2	16	0.0311543	regulation of cell-cell adhesion mediated by integrin
GO:0046427	3	136	0.0311926	positive regulation of JAK-STAT cascade
GO:0050920	4	450	0.0326629	regulation of chemotaxis
GO:0014070	7	2783	0.0335024	response to organic cyclic compound

Table 3: Overrepresented terms with the network-based enrichment. Only terms not detected with the standard method.

GO Term	N1	N2	P-value	Description
GO:0071621	3	142	0.0354962	granulocyte chemotaxis
GO:0009266	4	469	0.038418	response to temperature stimulus
GO:0045619	4	475	0.0403816	regulation of lymphocyte differentiation
GO:0006875	5	1046	0.0435876	cellular metal ion homeostasis
GO:0019725	6	1855	0.0446814	cellular homeostasis
GO:0097530	3	154	0.0452423	granulocyte migration
GO:0060326	4	489	0.0452506	cell chemotaxis
GO:0042510	2	20	0.0492882	regulation of tyrosine phosphorylation of Stat1 protein

Table 4: Overrepresented terms with the network-based enrichment. Only terms not detected with the standard method.
