# Supplementary material for: NET-GE: a novel NETwork-based Gene Enrichment for detecting biological processes associated to Mendelian diseases
Source: BMC Genomics. 2015 Jun 18;16(Suppl 8):S6. doi: 10.1186/1471-2164-16-S8-S6 (PMC4480278; doi:10.1186/1471-2164-16-S8-S6)
Supplement: Additional file 3 — Detailed results for the OMIM-derived benchmark set. The archive contains pdf documents listing the enriched terms for each one of the 244 diseases in the OMIM-derived benchmark set. [file 1471-2164-16-S8-S6-S3.tgz › SUPPMAT/OMIM601067.pdf]

#601067 USHER SYNDROME, TYPE ID; USH1D

**OMIM Gene ID**	**HGNC**	**UniProtAC**
605514	PCDH15	Q96QU1
605516	CDH23	Q9H251

Table 1: OMIM - UniProtAC mapping

Legend

- N1: #input proteins associated to the significant GO term
- N2: #proteins associated to the significant GO term
- P-value: Bonferroni-corrected p-value of Fisher's exact test
- *red*: go terms not related to the input proteins
- *blue*: go terms related to the input proteins (enriched uniquely by network-based method)
- *green*: go terms ancestors of terms enriched with the standard method (enriched uniquely by network-based method)

1 Standard enrichment

GO Term	N1	N2	P-value	Description
GO:0050957	2	6	1.01088e-06	equilibrioception
GO:0045494	2	37	4.48832e-05	photoreceptor cell maintenance
GO:0007605	2	175	0.00102605	sensory perception of sound
GO:0050954	2	180	0.00108569	sensory perception of mechanical stimulus
GO:0050953	2	218	0.00159403	sensory perception of light stimulus
GO:0007156	2	251	0.00211443	homophilic cell adhesion
GO:0098609	2	306	0.00314486	cell-cell adhesion
GO:0007600	2	586	0.0115513	sensory perception
GO:0050877	2	1063	0.0380398	neurological system process

Table 2: Overrepresented GO terms with the standard enrichment

2 Network-based enrichment

No novel enriched terms
