# Supplementary material for: NET-GE: a novel NETwork-based Gene Enrichment for detecting biological processes associated to Mendelian diseases
Source: BMC Genomics. 2015 Jun 18;16(Suppl 8):S6. doi: 10.1186/1471-2164-16-S8-S6 (PMC4480278; doi:10.1186/1471-2164-16-S8-S6)
Supplement: Additional file 3 — Detailed results for the OMIM-derived benchmark set. The archive contains pdf documents listing the enriched terms for each one of the 244 diseases in the OMIM-derived benchmark set. [file 1471-2164-16-S8-S6-S3.tgz › SUPPMAT/OMIM601367.pdf]

#601367 STROKE, ISCHEMIC

OMIM Gene ID	HGNC	UniProtAC
163729	NOS3	P29474
176930	F2	P00734
603700	ALOX5AP	P20292
605437	PRKCH	P24723
612309	F5	P12259

Table 1: OMIM - UniProtAC mapping

Legend

- N1: #input proteins associated to the significant GO term
- N2: #proteins associated to the significant GO term
- P-value: Bonferroni-corrected p-value of Fisher's exact test
- *red*: go terms not related to the input proteins
- *blue*: go terms related to the input proteins (enriched uniquely by network-based method)
- *green*: go terms ancestors of terms enriched with the standard method (enriched uniquely by network-based method)

1 Standard enrichment

GO Term	N1	N2	P-value	Description
GO:0007596	4	501	7.11876e-05	blood coagulation
GO:0050817	4	501	7.11876e-05	coagulation
GO:0007599	4	510	7.64437e-05	hemostasis
GO:0050878	4	717	0.000298324	regulation of body fluid levels
GO:0010544	2	13	0.000513316	negative regulation of platelet activation
GO:0030168	3	216	0.000859649	platelet activation
GO:0010543	2	35	0.0039111	regulation of platelet activation
GO:0044707	5	4361	0.00963917	single-multicellular organism process
GO:0032501	5	4447	0.0106283	multicellular organismal process
GO:0030195	2	59	0.0112325	negative regulation of blood coagulation
GO:1900047	2	59	0.0112325	negative regulation of hemostasis
GO:1903034	3	529	0.012574	regulation of response to wounding
GO:0050819	2	66	0.0140765	negative regulation of coagulation
GO:0001775	3	825	0.047226	cell activation
GO:0032101	3	825	0.047226	regulation of response to external stimulus

Table 2: Overrepresented GO terms with the standard enrichment

2 Network-based enrichment

GO Term	N1	N2	P-value	Description
GO:0010035	5	1420	0.000204854	response to inorganic substance
GO:0003013	4	444	0.000225765	circulatory system process
GO:0008015	3	128	0.000807589	blood circulation
GO:0002576	3	185	0.00244979	platelet degranulation
GO:0010522	3	190	0.00265438	regulation of calcium ion transport into cytosol
GO:0010038	4	951	0.00472726	response to metal ion
GO:0009408	3	232	0.00483718	response to heat
GO:0019722	3	249	0.00598106	calcium-mediated signaling
GO:0030193	3	273	0.00788242	regulation of blood coagulation
GO:1900046	3	273	0.00788242	regulation of hemostasis
GO:0080134	5	3072	0.00974444	regulation of response to stress
GO:0030003	4	1151	0.0101048	cellular cation homeostasis
GO:0032844	4	1155	0.0102452	regulation of homeostatic process
GO:0050818	3	299	0.0103536	regulation of coagulation
GO:0006873	4	1202	0.0120058	cellular ion homeostasis
GO:0051592	3	321	0.0128074	response to calcium ion
GO:1903036	3	341	0.015348	positive regulation of response to wounding
GO:0051591	3	378	0.0208887	response to cAMP
GO:0055080	4	1386	0.0211415	cation homeostasis
GO:0006629	5	3598	0.0214864	lipid metabolic process
GO:0055082	4	1433	0.0241336	cellular chemical homeostasis
GO:0061041	3	405	0.0256744	regulation of wound healing
GO:2001258	2	45	0.0260862	negative regulation of cation channel activity
GO:0051054	3	411	0.0268281	positive regulation of DNA metabolic process
GO:0042493	4	1473	0.0269194	response to drug
GO:0070528	2	49	0.0309798	protein kinase C signaling
GO:0050801	4	1555	0.0333719	ion homeostasis
GO:0007265	3	457	0.0368319	Ras protein signal transduction
GO:0019932	3	457	0.0368319	second-messenger-mediated signaling
GO:0009266	3	469	0.0397953	response to temperature stimulus
GO:2000021	3	471	0.040304	regulation of ion homeostasis
GO:0018200	2	57	0.0420238	peptidyl-glutamic acid modification
GO:0046683	3	488	0.0448029	response to organophosphorus
GO:0008016	3	497	0.0473135	regulation of heart contraction
GO:0010744	2	62	0.0497764	positive regulation of macrophage derived foam cell differentiation

Table 3: Overrepresented terms with the network-based enrichment. Only terms not detected with the standard method.
