# Supplementary material for: NET-GE: a novel NETwork-based Gene Enrichment for detecting biological processes associated to Mendelian diseases
Source: BMC Genomics. 2015 Jun 18;16(Suppl 8):S6. doi: 10.1186/1471-2164-16-S8-S6 (PMC4480278; doi:10.1186/1471-2164-16-S8-S6)
Supplement: Additional file 3 — Detailed results for the OMIM-derived benchmark set. The archive contains pdf documents listing the enriched terms for each one of the 244 diseases in the OMIM-derived benchmark set. [file 1471-2164-16-S8-S6-S3.tgz › SUPPMAT/OMIM601386.pdf]

#601386 DEAFNESS, AUTOSOMAL RECESSIVE 12; DFNB12

OMIM Gene ID	HGNC	UniProtAC
108733	ATP2B2	Q01814
605516	CDH23	Q9H251

Table 1: OMIM - UniProtAC mapping

Legend

- N1: #input proteins associated to the significant GO term
- N2: #proteins associated to the significant GO term
- P-value: Bonferroni-corrected p-value of Fisher's exact test
- *red*: go terms not related to the input proteins
- *blue*: go terms related to the input proteins (enriched uniquely by network-based method)
- *green*: go terms ancestors of terms enriched with the standard method (enriched uniquely by network-based method)

1 Standard enrichment

GO Term	N1	N2	P-value	Description
GO:0007605	2	175	0.00421107	sensory perception of sound
GO:0050954	2	180	0.00445584	sensory perception of mechanical stimulus
GO:0051480	2	222	0.006785	cytosolic calcium ion homeostasis
GO:0045299	1	1	0.010439	otolith mineralization
GO:0006874	2	359	0.0177739	cellular calcium ion homeostasis
GO:0055074	2	373	0.0191892	calcium ion homeostasis
GO:0072503	2	373	0.0191892	cellular divalent inorganic cation homeostasis
GO:0006816	2	390	0.0209807	calcium ion transport
GO:0072507	2	398	0.0218514	divalent inorganic cation homeostasis
GO:0070838	2	433	0.0258689	divalent metal ion transport
GO:0072511	2	437	0.0263495	divalent inorganic cation transport
GO:0006875	2	503	0.0349202	cellular metal ion homeostasis
GO:0030003	2	536	0.0396573	cellular cation homeostasis
GO:0006873	2	553	0.0422151	cellular ion homeostasis
GO:0007600	2	586	0.0474086	sensory perception
GO:0055065	2	593	0.0485491	metal ion homeostasis

Table 2: Overrepresented GO terms with the standard enrichment

2 Network-based enrichment

GO Term	N1	N2	P-value	Description
GO:0050974	2	72	0.000732354	detection of mechanical stimulus involved in sensory perception
GO:0050982	2	100	0.00141829	detection of mechanical stimulus

Table 3: Overrepresented terms with the network-based enrichment. Only terms not detected with the standard method.
