# Supplementary material for: NET-GE: a novel NETwork-based Gene Enrichment for detecting biological processes associated to Mendelian diseases
Source: BMC Genomics. 2015 Jun 18;16(Suppl 8):S6. doi: 10.1186/1471-2164-16-S8-S6 (PMC4480278; doi:10.1186/1471-2164-16-S8-S6)
Supplement: Additional file 3 — Detailed results for the OMIM-derived benchmark set. The archive contains pdf documents listing the enriched terms for each one of the 244 diseases in the OMIM-derived benchmark set. [file 1471-2164-16-S8-S6-S3.tgz › SUPPMAT/OMIM601462.pdf]

#601462 MYASTHENIC SYNDROME, CONGENITAL, SLOW-CHANNEL; SCCMS

OMIM Gene ID	HGNC	UniProtAC
100690	CHRNA1	P02708
100710	CHRNB1	P11230
100720	CHRND	Q07001
100725	CHRNE	Q04844

Table 1: OMIM - UniProtAC mapping

Legend

- N1: #input proteins associated to the significant GO term
- N2: #proteins associated to the significant GO term
- P-value: Bonferroni-corrected p-value of Fisher's exact test
- *red*: go terms not related to the input proteins
- *blue*: go terms related to the input proteins (enriched uniquely by network-based method)
- *green*: go terms ancestors of terms enriched with the standard method (enriched uniquely by network-based method)

1 Standard enrichment

GO Term	N1	N2	P-value	Description
GO:0006936	4	261	1.69852e-07	muscle contraction
GO:0003012	4	320	3.85446e-07	muscle system process
GO:0042391	4	353	5.71781e-07	regulation of membrane potential
GO:0048630	2	3	1.92061e-06	skeletal muscle tissue growth
GO:0007268	4	530	2.92222e-06	synaptic transmission
GO:0007267	4	859	2.02522e-05	cell-cell signaling
GO:0023052	4	913	2.5856e-05	signaling
GO:0044700	4	913	2.5856e-05	single organism signaling
GO:0098655	4	1072	4.91907e-05	cation transmembrane transport
GO:0007154	4	1103	5.5141e-05	cell communication
GO:0050879	2	20	0.000121566	multicellular organismal movement
GO:0050881	2	20	0.000121566	musculoskeletal movement
GO:0007271	2	25	0.000191912	synaptic transmission, cholinergic
GO:0034220	4	1538	0.00020877	ion transmembrane transport
GO:0006812	4	1582	0.00023373	cation transport
GO:0003008	4	1588	0.000237299	system process
GO:0007274	2	28	0.000241783	neuromuscular synaptic transmission
GO:0055085	4	2352	0.00114334	transmembrane transport
GO:0006811	4	2423	0.00128788	ion transport
GO:0050905	2	125	0.00494022	neuromuscular process
GO:0050877	3	1063	0.00663019	neurological system process
GO:0065008	4	3888	0.0085462	regulation of biological quality
GO:0044707	4	4361	0.0135296	single-multicellular organism process
GO:0032501	4	4447	0.0146292	multicellular organismal process
GO:0044765	4	5114	0.02559	single-organism transport
GO:0048589	2	296	0.0276628	developmental growth
GO:0070050	1	5	0.0402659	neuron cellular homeostasis

Table 2: Overrepresented GO terms with the standard enrichment

2 Network-based enrichment

GO Term	N1	N2	P-value	Description
GO:0046716	2	51	0.00246279	muscle cell cellular homeostasis
GO:0007528	2	106	0.0107256	neuromuscular junction development
GO:0035094	2	107	0.0109295	response to nicotine
GO:0044802	3	1291	0.0403665	single-organism membrane organization

Table 3: Overrepresented terms with the network-based enrichment. Only terms not detected with the standard method.
