# Supplementary material for: NET-GE: a novel NETwork-based Gene Enrichment for detecting biological processes associated to Mendelian diseases
Source: BMC Genomics. 2015 Jun 18;16(Suppl 8):S6. doi: 10.1186/1471-2164-16-S8-S6 (PMC4480278; doi:10.1186/1471-2164-16-S8-S6)
Supplement: Additional file 3 — Detailed results for the OMIM-derived benchmark set. The archive contains pdf documents listing the enriched terms for each one of the 244 diseases in the OMIM-derived benchmark set. [file 1471-2164-16-S8-S6-S3.tgz › SUPPMAT/OMIM601626.pdf]

#601626 LEUKEMIA, ACUTE MYELOID; AML

OMIM Gene ID	HGNC	UniProtAC
114350	NUP214	P35658
116897	CEBPA	P49715
121360	CBFB	Q13951
136351	FLT3	P36888
137295	GATA2	P23769
147796	JAK2	O60674
151385	RUNX1	Q01196
164040	NPM1	P06748
164920	KIT	P10721
187270	TERT	O14746
600358	GMPS	P49915
600618	ETV6	P41212
600700	LPP	Q93052
601402	MLF1	P58340
601768	SH3GL1	Q99961
602409	MLLT10	P55197
603025	PICALM	Q13492
604332	CHIC2	Q9UKJ5
604763	ARHGEF12	Q9NZN5
606681	NSD1	Q96L73
607083	WHSC1L1	Q9BZ95

Table 1: OMIM - UniProtAC mapping

Legend

- N1: #input proteins associated to the significant GO term
- N2: #proteins associated to the significant GO term
- P-value: Bonferroni-corrected p-value of Fisher's exact test
- *red*: go terms not related to the input proteins
- *blue*: go terms related to the input proteins (enriched uniquely by network-based method)
- *green*: go terms ancestors of terms enriched with the standard method (enriched uniquely by network-based method)

1 Standard enrichment

GO Term	N1	N2	P-value	Description
GO:0002318	4	9	9.66087e-09	myeloid progenitor cell differentiation
GO:0030097	6	154	2.34141e-07	hemopoiesis
GO:0048534	6	272	7.12766e-06	hematopoietic or lymphoid organ development
GO:0010604	12	3285	2.79612e-05	positive regulation of macromolecule metabolic process
GO:0031325	12	3418	4.36467e-05	positive regulation of cellular metabolic process
GO:0010557	10	2042	4.64188e-05	positive regulation of macromolecule biosynthetic process
GO:0031328	10	2177	8.49235e-05	positive regulation of cellular biosynthetic process
GO:0009893	12	3630	8.55046e-05	positive regulation of metabolic process
GO:0009891	10	2208	9.70092e-05	positive regulation of biosynthetic process
GO:0030099	5	244	0.000219893	myeloid cell differentiation
GO:0060216	3	24	0.000323523	definitive hemopoiesis
GO:0010628	9	1919	0.000407122	positive regulation of gene expression
GO:0051173	9	2108	0.00089762	positive regulation of nitrogen compound metabolic process
GO:0048519	13	5756	0.00156296	negative regulation of biological process
GO:0048522	13	5768	0.00160153	positive regulation of cellular process
GO:0045893	8	1762	0.00283978	positive regulation of transcription, DNA-templated
GO:0030154	9	2446	0.00309942	cell differentiation
GO:0032502	14	7299	0.00319476	developmental process
GO:1902680	8	1811	0.00348352	positive regulation of RNA biosynthetic process
GO:0042531	3	52	0.00349733	positive regulation of tyrosine phosphorylation of STAT protein
GO:0002244	4	188	0.00362311	hematopoietic progenitor cell differentiation
GO:0051254	8	1838	0.00388874	positive regulation of RNA metabolic process
GO:0048568	4	194	0.00410346	embryonic organ development
GO:0048523	12	5279	0.00513407	negative regulation of cellular process
GO:0042509	3	61	0.00567712	regulation of tyrosine phosphorylation of STAT protein
GO:0046427	3	65	0.00688031	positive regulation of JAK-STAT cascade
GO:0014068	3	67	0.00754041	positive regulation of phosphatidylinositol 3-kinase signaling
GO:0048518	13	6624	0.00795947	positive regulation of biological process
GO:0045935	8	2060	0.00903643	positive regulation of nucleobase-containing compound metabolic process
GO:0021700	4	238	0.00920161	developmental maturation
GO:0044767	13	6740	0.00971015	single-organism developmental process
GO:0048869	10	3694	0.0110298	cellular developmental process
GO:0019221	5	546	0.0113393	cytokine-mediated signaling pathway
GO:0090304	12	5696	0.0115199	nucleic acid metabolic process
GO:0034654	10	3760	0.0129213	nucleobase-containing compound biosynthetic process
GO:0044260	16	11069	0.013143	cellular macromolecule metabolic process
GO:0006351	9	2949	0.014358	transcription, DNA-templated
GO:0019438	10	3919	0.0186822	aromatic compound biosynthetic process
GO:0018130	10	3931	0.0191959	heterocycle biosynthetic process
GO:0046425	3	92	0.0195908	regulation of JAK-STAT cascade
GO:0014066	3	95	0.0215699	regulation of phosphatidylinositol 3-kinase signaling
GO:0044271	10	3991	0.0219542	cellular nitrogen compound biosynthetic process
GO:0034645	10	4013	0.0230487	cellular macromolecule biosynthetic process
GO:0006139	13	7311	0.0244509	nucleobase-containing compound metabolic process
GO:0006355	12	6146	0.0256029	regulation of transcription, DNA-templated
GO:1901362	10	4099	0.0277961	organic cyclic compound biosynthetic process
GO:0006807	14	8714	0.0282601	nitrogen compound metabolic process
GO:0002320	2	14	0.0289941	lymphoid progenitor cell differentiation
GO:2001141	12	6249	0.0304438	regulation of RNA biosynthetic process
GO:0032774	9	3250	0.0314394	RNA biosynthetic process
GO:0071310	8	2482	0.0351352	cellular response to organic substance
GO:1902106	3	112	0.0353025	negative regulation of leukocyte differentiation
GO:0051252	12	6360	0.0365537	regulation of RNA metabolic process
GO:0046483	13	7642	0.0401962	heterocycle metabolic process
GO:0006725	13	7662	0.0413875	cellular aromatic compound metabolic process
GO:0071345	5	725	0.0441166	cellular response to cytokine stimulus
GO:0009059	10	4344	0.0462741	macromolecule biosynthetic process
GO:0042523	2	18	0.048683	positive regulation of tyrosine phosphorylation of Stat5 protein

Table 2: Overrepresented GO terms with the standard enrichment

2 Network-based enrichment

GO Term	N1	N2	P-value	Description
GO:0045667	6	360	0.000129433	regulation of osteoblast differentiation
GO:0010629	12	3561	0.000454654	negative regulation of gene expression
GO:0002573	5	242	0.000643148	myeloid leukocyte differentiation
GO:0045944	11	2989	0.000802864	positive regulation of transcription from RNA polymerase II promoter
GO:0048585	12	3759	0.000824883	negative regulation of response to stimulus
GO:0045638	5	261	0.000934209	negative regulation of myeloid cell differentiation
GO:0051052	7	905	0.00159232	regulation of DNA metabolic process
GO:0009968	11	3204	0.00161849	negative regulation of signal transduction
GO:0002521	7	929	0.00189651	leukocyte differentiation
GO:0045637	6	596	0.00247187	regulation of myeloid cell differentiation
GO:0010648	11	3347	0.00250828	negative regulation of cell communication
GO:0023057	11	3347	0.00250828	negative regulation of signaling
GO:0006366	8	1448	0.00274175	transcription from RNA polymerase II promoter
GO:0030278	6	644	0.00386851	regulation of ossification
GO:0030098	6	654	0.00422819	lymphocyte differentiation
GO:0051270	9	2182	0.00557588	regulation of cellular component movement
GO:0040012	9	2224	0.00652725	regulation of locomotion
GO:0016032	8	1665	0.00776995	viral process
GO:0044403	8	1665	0.00776995	symbiosis, encompassing mutualism through parasitism
GO:0048646	10	2968	0.00780023	anatomical structure formation involved in morphogenesis
GO:0044764	8	1682	0.00837771	multi-organism cellular process
GO:0042326	7	1164	0.00845563	negative regulation of phosphorylation
GO:0045669	4	184	0.00896918	positive regulation of osteoblast differentiation
GO:0046649	7	1196	0.0101057	lymphocyte activation
GO:0036017	2	6	0.010151	response to erythropoietin
GO:0036018	2	6	0.010151	cellular response to erythropoietin
GO:0008285	9	2354	0.0104186	negative regulation of cell proliferation
GO:1902533	9	2418	0.0129803	positive regulation of intracellular signal transduction
GO:0007169	8	1794	0.0134895	transmembrane receptor protein tyrosine kinase signaling pathway
GO:0032269	8	1804	0.0140532	negative regulation of cellular protein metabolic process
GO:0006461	10	3196	0.0151997	protein complex assembly
GO:0048469	5	468	0.0162078	cell maturation
GO:0007167	9	2504	0.0172684	enzyme linked receptor protein signaling pathway
GO:0045597	9	2514	0.0178385	positive regulation of cell differentiation
GO:0035162	3	68	0.0188693	embryonic hemopoiesis
GO:0032270	10	3279	0.0191239	positive regulation of cellular protein metabolic process
GO:0045639	4	226	0.020189	positive regulation of myeloid cell differentiation
GO:0071363	8	1903	0.0208059	cellular response to growth factor stimulus
GO:0044419	8	1908	0.0212097	interspecies interaction between organisms
GO:0009888	9	2570	0.0213412	tissue development
GO:0001816	4	232	0.0223805	cytokine production
GO:0010563	7	1353	0.0226295	negative regulation of phosphorus metabolic process
GO:0045936	7	1353	0.0226295	negative regulation of phosphate metabolic process
GO:0048731	9	2612	0.0243431	system development
GO:0070848	8	1987	0.0285301	response to growth factor
GO:0016570	6	929	0.0314038	histone modification
GO:0016569	6	935	0.0325672	covalent chromatin modification
GO:2000145	8	2039	0.0344359	regulation of cell motility
GO:0010810	5	552	0.0358841	regulation of cell-substrate adhesion
GO:0016525	4	262	0.0360655	negative regulation of angiogenesis
GO:0045646	3	85	0.0369326	regulation of erythrocyte differentiation
GO:0051247	10	3557	0.0394362	positive regulation of protein metabolic process
GO:0045321	7	1486	0.0415383	leukocyte activation
GO:0071900	7	1492	0.0426319	regulation of protein serine/threonine kinase activity
GO:0070661	4	281	0.0474257	leukocyte proliferation
GO:2000113	10	3633	0.0475288	negative regulation of cellular macromolecule biosynthetic process

Table 3: Overrepresented terms with the network-based enrichment. Only terms not detected with the standard method.
