# Supplementary material for: NET-GE: a novel NETwork-based Gene Enrichment for detecting biological processes associated to Mendelian diseases
Source: BMC Genomics. 2015 Jun 18;16(Suppl 8):S6. doi: 10.1186/1471-2164-16-S8-S6 (PMC4480278; doi:10.1186/1471-2164-16-S8-S6)
Supplement: Additional file 3 — Detailed results for the OMIM-derived benchmark set. The archive contains pdf documents listing the enriched terms for each one of the 244 diseases in the OMIM-derived benchmark set. [file 1471-2164-16-S8-S6-S3.tgz › SUPPMAT/OMIM601634.pdf]

#601634 NEURAL TUBE DEFECTS, FOLATE-SENSITIVE

**OMIM Gene ID**	**HGNC**	**UniProtAC**
156570	MTR	Q99707
172460	MTHFD1	P11586
602568	MTRR	Q9UBK8
607093	MTHFR	P42898

Table 1: OMIM - UniProtAC mapping

Legend

- N1: #input proteins associated to the significant GO term
- N2: #proteins associated to the significant GO term
- P-value: Bonferroni-corrected p-value of Fisher's exact test
- *red*: go terms not related to the input proteins
- *blue*: go terms related to the input proteins (enriched uniquely by network-based method)
- *green*: go terms ancestors of terms enriched with the standard method (enriched uniquely by network-based method)

1 Standard enrichment

GO Term	N1	N2	P-value	Description
GO:0009086	4	22	1.22002e-11	methionine biosynthetic process
GO:0006555	4	25	2.10981e-11	methionine metabolic process
GO:0000097	4	29	3.96128e-11	sulfur amino acid biosynthetic process
GO:0009067	4	35	8.73279e-11	aspartate family amino acid biosynthetic process
GO:0009066	4	58	7.07613e-10	aspartate family amino acid metabolic process
GO:0000096	4	60	8.13296e-10	sulfur amino acid metabolic process
GO:0042558	4	78	2.37905e-09	pteridine-containing compound metabolic process
GO:0006767	4	109	9.2786e-09	water-soluble vitamin metabolic process
GO:0046655	3	13	1.79985e-08	folic acid metabolic process
GO:0006766	4	148	3.20069e-08	vitamin metabolic process
GO:1901607	4	160	4.38547e-08	alpha-amino acid biosynthetic process
GO:0044272	4	186	8.05186e-08	sulfur compound biosynthetic process
GO:0008652	4	222	1.64269e-07	cellular amino acid biosynthetic process
GO:1901605	4	396	1.68316e-06	alpha-amino acid metabolic process
GO:0006760	3	65	2.74602e-06	folic acid-containing compound metabolic process
GO:0016053	4	457	2.99151e-06	organic acid biosynthetic process
GO:0046394	4	457	2.99151e-06	carboxylic acid biosynthetic process
GO:0006790	4	463	3.15228e-06	sulfur compound metabolic process
GO:0044283	4	631	1.09125e-05	small molecule biosynthetic process
GO:0043648	3	129	2.19441e-05	dicarboxylic acid metabolic process
GO:0006520	4	839	3.41886e-05	cellular amino acid metabolic process
GO:1901566	4	954	5.72007e-05	organonitrogen compound biosynthetic process
GO:0035999	2	11	6.53075e-05	tetrahydrofolate interconversion
GO:0009235	2	21	0.000249268	cobalamin metabolic process
GO:0006575	3	340	0.000405959	cellular modified amino acid metabolic process
GO:0019752	4	1590	0.000442478	carboxylic acid metabolic process
GO:0046653	2	30	0.000516177	tetrahydrofolate metabolic process
GO:0006732	3	369	0.000519008	coenzyme metabolic process
GO:0043436	4	1732	0.000623202	oxoacid metabolic process
GO:0006082	4	1753	0.000654007	organic acid metabolic process
GO:0044711	4	1895	0.000893313	single-organism biosynthetic process
GO:0051186	3	461	0.00101182	cofactor metabolic process
GO:0006730	2	57	0.00189202	one-carbon metabolic process
GO:0033013	2	99	0.00574224	tetrapyrrole metabolic process
GO:1901564	4	3152	0.00684637	organonitrogen compound metabolic process
GO:0006805	2	163	0.0155933	xenobiotic metabolic process
GO:0044281	4	4403	0.0260823	small molecule metabolic process
GO:0000105	1	2	0.0298851	histidine biosynthetic process

Table 2: Overrepresented GO terms with the standard enrichment

2 Network-based enrichment

No novel enriched terms
