# Supplementary material for: NET-GE: a novel NETwork-based Gene Enrichment for detecting biological processes associated to Mendelian diseases
Source: BMC Genomics. 2015 Jun 18;16(Suppl 8):S6. doi: 10.1186/1471-2164-16-S8-S6 (PMC4480278; doi:10.1186/1471-2164-16-S8-S6)
Supplement: Additional file 3 — Detailed results for the OMIM-derived benchmark set. The archive contains pdf documents listing the enriched terms for each one of the 244 diseases in the OMIM-derived benchmark set. [file 1471-2164-16-S8-S6-S3.tgz › SUPPMAT/OMIM601665.pdf]

#601665 OBESITY

OMIM Gene ID	HGNC	UniProtAC
109690	ADRB2	P07550
109691	ADRB3	P13945
113730	UCP1	P25874
155541	MC4R	P32245
173335	ENPP1	P22413
176830	POMC	P01189
186357	SDC3	O75056
600781	PYY	P10082
601487	PPARG	P37231
602044	UCP3	P55916
602311	AGRP	O00253
602606	CARTPT	Q16568
603128	SIM1	P81133
604630	NR0B2	Q15466
605353	GHRL	Q9UBU3
608886	PPARGC1B	Q86YN6

Table 1: OMIM - UniProtAC mapping

Legend

- N1: #input proteins associated to the significant GO term
- N2: #proteins associated to the significant GO term
- P-value: Bonferroni-corrected p-value of Fisher's exact test
- *red*: go terms not related to the input proteins
- *blue*: go terms related to the input proteins (enriched uniquely by network-based method)
- *green*: go terms ancestors of terms enriched with the standard method (enriched uniquely by network-based method)

1 Standard enrichment

GO Term	N1	N2	P-value	Description
GO:0007631	6	130	1.05616e-08	feeding behavior
GO:0002024	3	7	1.99505e-06	diet induced thermogenesis
GO:0007186	9	1577	3.03804e-06	G-protein coupled receptor signaling pathway
GO:0050873	4	54	6.13738e-06	brown fat cell differentiation
GO:0008343	3	11	9.3955e-06	adult feeding behavior
GO:0051716	15	9450	1.05651e-05	cellular response to stimulus
GO:0009409	4	63	1.15334e-05	response to cold
GO:0008217	5	197	1.40125e-05	regulation of blood pressure
GO:0009725	8	1273	1.51275e-05	response to hormone
GO:0006091	6	531	4.89058e-05	generation of precursor metabolites and energy
GO:0051241	6	547	5.82752e-05	negative regulation of multicellular organismal process
GO:0032098	3	23	0.000100533	regulation of appetite
GO:0007165	13	7592	0.000241116	signal transduction
GO:0050896	15	11721	0.000248056	response to stimulus
GO:0032870	6	716	0.000283792	cellular response to hormone stimulus
GO:0032868	5	376	0.000348155	response to insulin
GO:0002025	2	3	0.000461354	vasodilation by norepinephrine-epinephrine involved in regulation of systemic arterial blood pressure
GO:0009719	8	2012	0.000514054	response to endogenous stimulus
GO:0044253	3	39	0.000516648	positive regulation of multicellular organismal metabolic process
GO:0007610	6	806	0.000566957	behavior
GO:0045444	4	167	0.000589374	fat cell differentiation
GO:0042755	3	42	0.000648486	eating behavior
GO:0009755	4	180	0.000794912	hormone-mediated signaling pathway
GO:0051240	6	866	0.000861388	positive regulation of multicellular organismal process
GO:0045124	3	48	0.000975504	regulation of bone resorption
GO:0044246	3	49	0.00103886	regulation of multicellular organismal metabolic process
GO:0009266	4	195	0.00109354	response to temperature stimulus
GO:0046850	3	61	0.00202305	regulation of bone remodeling
GO:0042592	7	1658	0.00229446	homeostatic process
GO:0051239	9	3432	0.00242637	regulation of multicellular organismal process
GO:0043434	5	567	0.00261437	response to peptide hormone
GO:0031649	2	7	0.00322629	heat generation
GO:0010243	6	1094	0.00333325	response to organonitrogen compound
GO:1901652	5	600	0.00344443	response to peptide
GO:0030072	3	77	0.00409491	peptide hormone secretion
GO:0002790	3	79	0.00442453	peptide secretion
GO:0023051	9	3708	0.00463336	regulation of signaling
GO:0010646	9	3714	0.00469622	regulation of cell communication
GO:1901700	7	1851	0.004763	response to oxygen-containing compound
GO:0048545	5	646	0.00493335	response to steroid hormone
GO:0030819	3	83	0.0051355	positive regulation of cAMP biosynthetic process
GO:1901698	6	1186	0.00530235	response to nitrogen compound
GO:0045926	4	303	0.00627062	negative regulation of growth
GO:0034103	3	89	0.00633759	regulation of tissue remodeling
GO:0030816	3	90	0.00655444	positive regulation of cAMP metabolic process
GO:0010562	6	1255	0.00733102	positive regulation of phosphorus metabolic process
GO:0045937	6	1255	0.00733102	positive regulation of phosphate metabolic process
GO:0030804	3	94	0.00747084	positive regulation of cyclic nucleotide biosynthetic process
GO:0030810	3	96	0.0079592	positive regulation of nucleotide biosynthetic process
GO:1900373	3	96	0.0079592	positive regulation of purine nucleotide biosynthetic process
GO:0071495	6	1291	0.00861726	cellular response to endogenous stimulus
GO:0015833	3	100	0.00899826	peptide transport
GO:0046883	4	336	0.00942079	regulation of hormone secretion
GO:0010817	4	337	0.00953154	regulation of hormone levels
GO:0030801	3	103	0.00983374	positive regulation of cyclic nucleotide metabolic process
GO:0046879	3	103	0.00983374	hormone secretion
GO:2000849	2	12	0.0101273	regulation of glucocorticoid secretion
GO:0051049	7	2081	0.0102988	regulation of transport
GO:0009914	3	109	0.0116553	hormone transport
GO:0032368	3	112	0.012644	regulation of lipid transport

Table 2: Overrepresented GO terms with the standard enrichment

GO Term	N1	N2	P-value	Description
GO:0031326	11	6987	0.0137405	regulation of cellular biosynthetic process
GO:2000846	2	14	0.0139564	regulation of corticosteroid hormone secretion
GO:0030817	3	117	0.0144123	regulation of cAMP biosynthetic process
GO:0009889	11	7051	0.0150505	regulation of biosynthetic process
GO:0040008	5	822	0.0158125	regulation of growth
GO:0060259	2	15	0.0160996	regulation of feeding behavior
GO:0040014	3	123	0.0167404	regulation of multicellular organism growth
GO:0045981	3	123	0.0167404	positive regulation of nucleotide metabolic process
GO:1900544	3	123	0.0167404	positive regulation of purine nucleotide metabolic process
GO:0044707	9	4361	0.0177235	single-multicellular organism process
GO:0009628	6	1467	0.0178316	response to abiotic stimulus
GO:0007166	9	4368	0.0179583	cell surface receptor signaling pathway
GO:0048519	10	5756	0.0202815	negative regulation of biological process
GO:0032501	9	4447	0.0207989	multicellular organismal process
GO:0071875	2	17	0.0208424	adrenergic receptor signaling pathway
GO:0030814	3	133	0.021149	regulation of cAMP metabolic process
GO:0046887	3	133	0.021149	positive regulation of hormone secretion
GO:0030802	3	137	0.0231067	regulation of cyclic nucleotide biosynthetic process
GO:0048583	9	4515	0.023547	regulation of response to stimulus
GO:0042886	3	139	0.0241287	amide transport
GO:0030808	3	140	0.0246506	regulation of nucleotide biosynthetic process
GO:1900371	3	140	0.0246506	regulation of purine nucleotide biosynthetic process
GO:0045780	2	19	0.0261933	positive regulation of bone resorption
GO:0046852	2	19	0.0261933	positive regulation of bone remodeling
GO:0031325	8	3418	0.0270728	positive regulation of cellular metabolic process
GO:0032095	2	20	0.0290965	regulation of response to food
GO:2000831	2	20	0.0290965	regulation of steroid hormone secretion
GO:0010033	8	3487	0.0313293	response to organic substance
GO:0048585	6	1639	0.0333498	negative regulation of response to stimulus
GO:0042221	9	4712	0.0333504	response to chemical
GO:0030799	3	155	0.0333939	regulation of cyclic nucleotide metabolic process
GO:0007188	3	162	0.0380888	adenylate cyclase-modulating G-protein coupled receptor signaling pathway
GO:0009893	8	3630	0.0419826	positive regulation of metabolic process
GO:0040015	2	24	0.0422247	negative regulation of multicellular organism growth
GO:0023061	3	168	0.0424421	signal release
GO:0003085	2	25	0.0458851	negative regulation of systemic arterial blood pressure
GO:0031323	12	9728	0.0481539	regulation of cellular metabolic process
GO:0007218	3	176	0.0487377	neuropeptide signaling pathway

Table 3: Overrepresented GO terms with the standard enrichment

2 Network-based enrichment

GO Term	N1	N2	P-value	Description
GO:0048511	10	930	3.66551e-09	rhythmic process
GO:0007623	8	457	2.68196e-08	circadian rhythm
GO:0001659	5	68	2.54577e-07	temperature homeostasis
GO:0050795	8	630	3.42744e-07	regulation of behavior
GO:0030278	8	644	4.07791e-07	regulation of ossification
GO:0009888	11	2570	3.3297e-06	tissue development
GO:0044708	9	1465	1.00931e-05	single-organism behavior
GO:0030534	7	605	1.21469e-05	adult behavior
GO:0048871	5	148	1.32033e-05	multicellular organismal homeostasis
GO:0003006	10	2335	2.95467e-05	developmental process involved in reproduction
GO:0043436	11	3244	3.92019e-05	oxoacid metabolic process
GO:0006082	11	3284	4.45989e-05	organic acid metabolic process
GO:0044702	11	3664	0.000140648	single organism reproductive process
GO:0044711	11	3675	0.000145126	single-organism biosynthetic process
GO:0016101	5	240	0.000148194	diterpenoid metabolic process
GO:0051051	8	1375	0.000153601	negative regulation of transport
GO:0014070	10	2783	0.000158486	response to organic cyclic compound
GO:0048609	9	2109	0.000237708	multicellular organismal reproductive process
GO:0032846	5	270	0.00026609	positive regulation of homeostatic process
GO:0060123	3	22	0.000270697	regulation of growth hormone secretion
GO:0006721	5	271	0.000271016	terpenoid metabolic process
GO:0051384	6	564	0.000309138	response to glucocorticoid
GO:0033500	6	567	0.000318933	carbohydrate homeostasis
GO:0042593	6	567	0.000318933	glucose homeostasis
GO:0051048	6	583	0.000375595	negative regulation of secretion
GO:0050796	6	606	0.000471379	regulation of insulin secretion
GO:0031960	6	610	0.000489926	response to corticosteroid
GO:0048608	7	1062	0.000567209	reproductive structure development
GO:0043627	6	647	0.000691835	response to estrogen
GO:0048878	9	2410	0.000745108	chemical homeostasis
GO:0006720	5	333	0.000751507	isoprenoid metabolic process
GO:0090276	6	675	0.000886434	regulation of peptide hormone secretion
GO:0009416	7	1146	0.000948304	response to light stimulus
GO:0002791	6	684	0.000957784	regulation of peptide secretion
GO:0090087	6	688	0.000990977	regulation of peptide transport
GO:0005975	9	2526	0.001112	carbohydrate metabolic process
GO:0043408	8	1837	0.0014142	regulation of MAPK cascade
GO:0031667	7	1219	0.001437	response to nutrient levels
GO:0033993	9	2604	0.00143983	response to lipid
GO:0048520	5	383	0.00149764	positive regulation of behavior
GO:0006629	10	3598	0.00179415	lipid metabolic process
GO:0043410	7	1269	0.00188224	positive regulation of MAPK cascade
GO:0051093	9	2690	0.00189643	negative regulation of developmental process
GO:0042327	9	2706	0.00199407	positive regulation of phosphorylation
GO:0009991	7	1313	0.0023653	response to extracellular stimulus
GO:0045670	4	176	0.00253572	regulation of osteoclast differentiation
GO:0006006	5	435	0.00279876	glucose metabolic process
GO:0044255	9	2817	0.00280092	cellular lipid metabolic process
GO:0032024	4	182	0.00289786	positive regulation of insulin secretion
GO:0032147	6	832	0.00299619	activation of protein kinase activity
GO:0055078	3	48	0.00301718	sodium ion homeostasis
GO:0003013	5	444	0.00309424	circulatory system process
GO:0050880	4	187	0.0032279	regulation of blood vessel size
GO:0071375	6	846	0.00330057	cellular response to peptide hormone stimulus
GO:0035150	4	198	0.00405151	regulation of tube size
GO:0007586	4	201	0.00430094	digestion
GO:1901653	6	892	0.00448457	cellular response to peptide
GO:0007190	3	55	0.00456717	activation of adenylate cyclase activity
GO:0071417	7	1481	0.00528315	cellular response to organonitrogen compound
GO:0071705	7	1511	0.0060366	nitrogen compound transport

Table 4: Overrepresented terms with the network-based enrichment. Only terms not detected with the standard method.

GO Term	N1	N2	P-value	Description
GO:2000253	2	6	0.00630186	positive regulation of feeding behavior
GO:0048468	8	2280	0.00723079	cell development
GO:0050801	7	1555	0.00730373	ion homeostasis
GO:0009314	7	1557	0.00736626	response to radiation
GO:0006839	4	231	0.00746728	mitochondrial transport
GO:0001666	6	987	0.00804106	response to hypoxia
GO:0090277	4	239	0.00854444	positive regulation of peptide hormone secretion
GO:0015980	5	547	0.00857058	energy derivation by oxidation of organic compounds
GO:0036293	6	999	0.00862012	response to decreased oxygen levels
GO:0008285	8	2354	0.0091846	negative regulation of cell proliferation
GO:0002793	4	247	0.00973266	positive regulation of peptide secretion
GO:1901699	7	1626	0.0098168	cellular response to nitrogen compound
GO:0055067	4	249	0.0100479	monovalent inorganic cation homeostasis
GO:0003018	4	250	0.0102083	vascular process in circulatory system
GO:0007492	3	72	0.010331	endoderm development
GO:0042752	4	251	0.0103707	regulation of circadian rhythm
GO:0001818	5	578	0.0112047	negative regulation of cytokine production
GO:1902533	8	2418	0.0112228	positive regulation of intracellular signal transduction
GO:0043255	4	259	0.011739	regulation of carbohydrate biosynthetic process
GO:0051047	6	1063	0.0123142	positive regulation of secretion
GO:0045762	3	77	0.0126527	positive regulation of adenylate cyclase activity
GO:0032845	3	78	0.013155	negative regulation of homeostatic process
GO:0042311	3	78	0.013155	vasodilation
GO:0070482	6	1076	0.0132029	response to oxygen levels
GO:0055086	9	3391	0.0132425	nucleobase-containing small molecule metabolic process
GO:0019318	5	609	0.0144373	hexose metabolic process
GO:0032869	5	609	0.0144373	cellular response to insulin stimulus
GO:0031175	5	614	0.0150209	neuron projection development
GO:1901701	8	2540	0.0161932	cellular response to oxygen-containing compound
GO:0043367	3	86	0.0176551	CD4-positive, alpha-beta T cell differentiation
GO:0032844	6	1155	0.0198001	regulation of homeostatic process
GO:0055114	9	3568	0.0201903	oxidation-reduction process
GO:0044060	3	91	0.0209272	regulation of endocrine process
GO:0035710	3	92	0.0216263	CD4-positive, alpha-beta T cell activation
GO:0010883	3	93	0.0223407	regulation of lipid storage
GO:0032680	4	306	0.0226449	regulation of tumor necrosis factor production
GO:0014060	2	11	0.0230745	regulation of epinephrine secretion
GO:0070858	2	11	0.0230745	negative regulation of bile acid biosynthetic process
GO:0019725	7	1855	0.023375	cellular homeostasis
GO:0044723	7	1859	0.0237072	single-organism carbohydrate metabolic process
GO:0019935	3	95	0.0238157	cyclic-nucleotide-mediated signaling
GO:0005996	5	680	0.0246099	monosaccharide metabolic process
GO:0009581	5	680	0.0246099	detection of external stimulus
GO:1901566	7	1881	0.0256063	organonitrogen compound biosynthetic process
GO:0009582	5	688	0.0260383	detection of abiotic stimulus
GO:0071877	2	12	0.0276816	regulation of adrenergic receptor signaling pathway
GO:0010906	4	323	0.0280033	regulation of glucose metabolic process
GO:0007154	8	2752	0.0293244	cell communication
GO:0015850	4	327	0.0293891	organic hydroxy compound transport
GO:0010558	9	3771	0.0318636	negative regulation of macromolecule biosynthetic process
GO:0010647	9	3801	0.0340077	positive regulation of cell communication
GO:0071496	5	736	0.03603	cellular response to external stimulus
GO:0014062	2	14	0.0381457	regulation of serotonin secretion
GO:0048806	3	114	0.0411447	genitalia development
GO:0006954	6	1314	0.0412286	inflammatory response
GO:0043951	2	15	0.0440019	negative regulation of cAMP-mediated signaling
GO:0030279	3	119	0.0467833	negative regulation of ossification
GO:0050994	3	121	0.0491741	regulation of lipid catabolic process
GO:0007602	4	374	0.0497257	phototransduction

Table 5: Overrepresented terms with the network-based enrichment. Only terms not detected with the standard method.
