# Supplementary material for: NET-GE: a novel NETwork-based Gene Enrichment for detecting biological processes associated to Mendelian diseases
Source: BMC Genomics. 2015 Jun 18;16(Suppl 8):S6. doi: 10.1186/1471-2164-16-S8-S6 (PMC4480278; doi:10.1186/1471-2164-16-S8-S6)
Supplement: Additional file 3 — Detailed results for the OMIM-derived benchmark set. The archive contains pdf documents listing the enriched terms for each one of the 244 diseases in the OMIM-derived benchmark set. [file 1471-2164-16-S8-S6-S3.tgz › SUPPMAT/OMIM601675.pdf]

#601675 TRICHOTHIODYSTROPHY, PHOTOSENSITIVE; TTDP

OMIM Gene ID	HGNC	UniProtAC
126340	ERCC2	P18074
133510	ERCC3	P19447
608780	GTF2H5	Q6ZYL4

Table 1: OMIM - UniProtAC mapping

Legend

- N1: #input proteins associated to the significant GO term
- N2: #proteins associated to the significant GO term
- P-value: Bonferroni-corrected p-value of Fisher's exact test
- *red*: go terms not related to the input proteins
- *blue*: go terms related to the input proteins (enriched uniquely by network-based method)
- *green*: go terms ancestors of terms enriched with the standard method (enriched uniquely by network-based method)

1 Standard enrichment

GO Term	N1	N2	P-value	Description
GO:0006362	3	26	8.03765e-08	transcription elongation from RNA polymerase I promoter
GO:0006354	3	98	4.70191e-06	DNA-templated transcription, elongation
GO:0006289	3	116	7.83547e-06	nucleotide-excision repair
GO:0033683	2	7	2.44991e-05	nucleotide-excision repair, DNA incision
GO:0009650	2	18	0.000178459	UV protection
GO:0035315	2	18	0.000178459	hair cell differentiation
GO:0000718	2	21	0.000244931	nucleotide-excision repair, DNA damage removal
GO:0044349	2	21	0.000244931	DNA excision
GO:0006363	2	24	0.000321891	termination of RNA polymerase I transcription
GO:0006361	2	27	0.00040934	transcription initiation from RNA polymerase I promoter
GO:0006360	2	33	0.000615696	transcription from RNA polymerase I promoter
GO:0006370	2	34	0.000654163	7-methylguanosine mRNA capping
GO:0009452	2	35	0.000693799	7-methylguanosine RNA capping
GO:0036260	2	35	0.000693799	RNA capping
GO:0009314	3	591	0.00105818	response to radiation
GO:0006283	2	49	0.00137093	transcription-coupled nucleotide-excision repair
GO:0050434	2	54	0.00166806	positive regulation of viral transcription
GO:0006281	3	781	0.00244505	DNA repair
GO:0006368	2	72	0.00297847	transcription elongation from RNA polymerase II promoter
GO:0048524	2	81	0.00377493	positive regulation of viral process
GO:0046782	2	85	0.0041591	regulation of viral transcription
GO:0006353	2	86	0.00425807	DNA-templated transcription, termination
GO:0006396	3	1004	0.00519885	RNA processing
GO:0032508	2	99	0.00565011	DNA duplex unwinding
GO:0043902	2	100	0.00576531	positive regulation of multi-organism process
GO:0032392	2	101	0.00588168	DNA geometric change
GO:0009913	2	105	0.00635876	epidermal cell differentiation
GO:0006974	3	1132	0.00745404	cellular response to DNA damage stimulus
GO:0009628	3	1467	0.0162333	response to abiotic stimulus
GO:0006259	3	1502	0.017424	DNA metabolic process
GO:0009411	2	177	0.0181168	response to UV
GO:0006308	2	181	0.0189459	DNA catabolic process
GO:0050792	2	182	0.0191561	regulation of viral process
GO:0000717	1	1	0.0220173	nucleotide-excision repair, DNA duplex unwinding
GO:0006294	1	1	0.0220173	nucleotide-excision repair, preincision complex assembly
GO:0071103	2	205	0.0243088	DNA conformation change
GO:0006367	2	214	0.0264913	transcription initiation from RNA polymerase II promoter
GO:0043903	2	223	0.0287673	regulation of symbiosis, encompassing mutualism through parasitism
GO:0033554	3	1930	0.0369828	cellular response to stress
GO:0006352	2	291	0.0489789	DNA-templated transcription, initiation

Table 2: Overrepresented GO terms with the standard enrichment

2 Network-based enrichment

GO Term	N1	N2	P-value	Description
GO:0034622	3	1660	0.0424833	cellular macromolecular complex assembly

Table 3: Overrepresented terms with the network-based enrichment. Only terms not detected with the standard method.
