# Supplementary material for: NET-GE: a novel NETwork-based Gene Enrichment for detecting biological processes associated to Mendelian diseases
Source: BMC Genomics. 2015 Jun 18;16(Suppl 8):S6. doi: 10.1186/1471-2164-16-S8-S6 (PMC4480278; doi:10.1186/1471-2164-16-S8-S6)
Supplement: Additional file 3 — Detailed results for the OMIM-derived benchmark set. The archive contains pdf documents listing the enriched terms for each one of the 244 diseases in the OMIM-derived benchmark set. [file 1471-2164-16-S8-S6-S3.tgz › SUPPMAT/OMIM601680.pdf]

#601680 ARTHROGRYPOSIS, DISTAL, TYPE 2B; DA2B

**OMIM Gene ID**	**HGNC**	**UniProtAC**
160720	MYH3	P11055
190990	TPM2	P07951
191043	TNNI2	P48788
600692	TNNT3	P45378

Table 1: OMIM - UniProtAC mapping

Legend

- N1: #input proteins associated to the significant GO term
- N2: #proteins associated to the significant GO term
- P-value: Bonferroni-corrected p-value of Fisher's exact test
- *red*: go terms not related to the input proteins
- *blue*: go terms related to the input proteins (enriched uniquely by network-based method)
- *green*: go terms ancestors of terms enriched with the standard method (enriched uniquely by network-based method)

1 Standard enrichment

GO Term	N1	N2	P-value	Description
GO:0030049	4	38	1.38828e-10	muscle filament sliding
GO:0033275	4	38	1.38828e-10	actin-myosin filament sliding
GO:0070252	4	51	4.70001e-10	actin-mediated cell contraction
GO:0030048	4	74	2.16404e-09	actin filament-based movement
GO:0003009	3	30	2.88022e-07	skeletal muscle contraction
GO:0006936	4	261	3.55348e-07	muscle contraction
GO:0003012	4	320	8.06394e-07	muscle system process
GO:0030029	4	510	5.23938e-06	actin filament-based process
GO:0006941	3	96	1.01228e-05	striated muscle contraction
GO:0003008	4	1588	0.000496455	system process
GO:0006928	4	1973	0.00118388	cellular component movement
GO:0043462	2	48	0.00150841	regulation of ATPase activity
GO:1903289	2	48	0.00150841	regulation of ATP catabolic process
GO:0044707	4	4361	0.0283053	single-multicellular organism process
GO:0032501	4	4447	0.0306058	multicellular organismal process

Table 2: Overrepresented GO terms with the standard enrichment

2 Network-based enrichment

No novel enriched terms
