# Supplementary material for: NET-GE: a novel NETwork-based Gene Enrichment for detecting biological processes associated to Mendelian diseases
Source: BMC Genomics. 2015 Jun 18;16(Suppl 8):S6. doi: 10.1186/1471-2164-16-S8-S6 (PMC4480278; doi:10.1186/1471-2164-16-S8-S6)
Supplement: Additional file 3 — Detailed results for the OMIM-derived benchmark set. The archive contains pdf documents listing the enriched terms for each one of the 244 diseases in the OMIM-derived benchmark set. [file 1471-2164-16-S8-S6-S3.tgz › SUPPMAT/OMIM601859.pdf]

#601859 AUTOIMMUNE LYMPHOPROLIFERATIVE SYNDROME; ALPS

**OMIM Gene ID**	**HGNC**	**UniProtAC**
134637	FAS	P25445
134638	FASLG	P48023

Table 1: OMIM - UniProtAC mapping

Legend

- N1: #input proteins associated to the significant GO term
- N2: #proteins associated to the significant GO term
- P-value: Bonferroni-corrected p-value of Fisher's exact test
- *red*: go terms not related to the input proteins
- *blue*: go terms related to the input proteins (enriched uniquely by network-based method)
- *green*: go terms ancestors of terms enriched with the standard method (enriched uniquely by network-based method)

1 Standard enrichment

GO Term	N1	N2	P-value	Description
GO:0097527	2	6	6.04425e-06	necroptotic signaling pathway
GO:0006925	2	9	1.45062e-05	inflammatory cell apoptotic process
GO:0070231	2	19	6.89044e-05	T cell apoptotic process
GO:0070227	2	21	8.46194e-05	lymphocyte apoptotic process
GO:0071887	2	28	0.000152315	leukocyte apoptotic process
GO:0008625	2	47	0.000435589	extrinsic apoptotic signaling pathway via death domain receptors
GO:2001239	2	104	0.0021582	regulation of extrinsic apoptotic signaling pathway in absence of ligand
GO:0006919	2	105	0.0022001	activation of cysteine-type endopeptidase activity involved in apoptotic process
GO:0097202	2	110	0.00241568	activation of cysteine-type endopeptidase activity
GO:0031638	2	123	0.00302332	zymogen activation
GO:0097191	2	132	0.00348389	extrinsic apoptotic signaling pathway
GO:0097285	2	135	0.00364467	cell-type specific apoptotic process
GO:0043280	2	140	0.00392071	positive regulation of cysteine-type endopeptidase activity involved in apoptotic process
GO:2001056	2	148	0.00438329	positive regulation of cysteine-type endopeptidase activity
GO:0010950	2	160	0.0051255	positive regulation of endopeptidase activity
GO:0010952	2	174	0.0060648	positive regulation of peptidase activity
GO:0043281	2	252	0.0127437	regulation of cysteine-type endopeptidase activity involved in apoptotic process
GO:2001236	2	253	0.0128452	regulation of extrinsic apoptotic signaling pathway
GO:2000116	2	263	0.0138828	regulation of cysteine-type endopeptidase activity
GO:0016485	2	301	0.0181932	protein processing
GO:0051604	2	340	0.023222	protein maturation
GO:0097049	1	2	0.030416	motor neuron apoptotic process
GO:0097190	2	439	0.0387398	apoptotic signaling pathway
GO:0046666	1	3	0.0456232	retinal cell programmed cell death
GO:0048388	1	3	0.0456232	endosomal lumen acidification

Table 2: Overrepresented GO terms with the standard enrichment

2 Network-based enrichment

GO Term	N1	N2	P-value	Description
GO:2000351	2	92	0.00704546	regulation of endothelial cell apoptotic process
GO:0050829	2	93	0.00720031	defense response to Gram-negative bacterium
GO:0002637	2	133	0.0147743	regulation of immunoglobulin production
GO:0002460	2	160	0.0214091	adaptive immune response based on somatic recombination of immune receptors built from immunoglobulin su[illegible]
GO:0050871	2	179	0.0268135	positive regulation of B cell activation
GO:0031343	2	201	0.0338303	positive regulation of cell killing
GO:0031341	2	224	0.0420371	regulation of cell killing
GO:0045582	2	229	0.043939	positive regulation of T cell differentiation
GO:0003014	2	240	0.0482714	renal system process

Table 3: Overrepresented terms with the network-based enrichment. Only terms not detected with the standard method.
