# Supplementary material for: NET-GE: a novel NETwork-based Gene Enrichment for detecting biological processes associated to Mendelian diseases
Source: BMC Genomics. 2015 Jun 18;16(Suppl 8):S6. doi: 10.1186/1471-2164-16-S8-S6 (PMC4480278; doi:10.1186/1471-2164-16-S8-S6)
Supplement: Additional file 3 — Detailed results for the OMIM-derived benchmark set. The archive contains pdf documents listing the enriched terms for each one of the 244 diseases in the OMIM-derived benchmark set. [file 1471-2164-16-S8-S6-S3.tgz › SUPPMAT/OMIM602080.pdf]

#602080 PAGET DISEASE OF BONE; PDB

**OMIM Gene ID**	**HGNC**	**UniProtAC**
601530	SQSTM1	Q13501
603499	TNFRSF11A	Q9Y6Q6

Table 1: OMIM - UniProtAC mapping

Legend

- N1: #input proteins associated to the significant GO term
- N2: #proteins associated to the significant GO term
- P-value: Bonferroni-corrected p-value of Fisher's exact test
- *red*: go terms not related to the input proteins
- *blue*: go terms related to the input proteins (enriched uniquely by network-based method)
- *green*: go terms ancestors of terms enriched with the standard method (enriched uniquely by network-based method)

1 Standard enrichment

GO Term	N1	N2	P-value	Description
GO:0032103	2	245	0.0120442	positive regulation of response to external stimulus
GO:0060086	1	2	0.030416	circadian temperature homeostasis
GO:0071810	1	2	0.030416	regulation of fever generation by regulation of prostaglandin secretion
GO:0071812	1	2	0.030416	positive regulation of fever generation by positive regulation of prostaglandin secretion
GO:0071847	1	2	0.030416	TNFSF11-mediated signaling pathway
GO:0071848	1	2	0.030416	positive regulation of ERK1 and ERK2 cascade via TNFSF11-mediated signaling

Table 2: Overrepresented GO terms with the standard enrichment

2 Network-based enrichment

GO Term	N1	N2	P-value	Description
GO:0002250	2	249	0.0351643	adaptive immune response

Table 3: Overrepresented terms with the network-based enrichment. Only terms not detected with the standard method.
