# Supplementary material for: NET-GE: a novel NETwork-based Gene Enrichment for detecting biological processes associated to Mendelian diseases
Source: BMC Genomics. 2015 Jun 18;16(Suppl 8):S6. doi: 10.1186/1471-2164-16-S8-S6 (PMC4480278; doi:10.1186/1471-2164-16-S8-S6)
Supplement: Additional file 3 — Detailed results for the OMIM-derived benchmark set. The archive contains pdf documents listing the enriched terms for each one of the 244 diseases in the OMIM-derived benchmark set. [file 1471-2164-16-S8-S6-S3.tgz › SUPPMAT/OMIM602089.pdf]

#602089 HEMANGIOMA, CAPILLARY INFANTILE

OMIM Gene ID	HGNC	UniProtAC
136352	FLT4	P35916
191306	KDR	P35968
606410	ANTXR1	Q9H6X2

Table 1: OMIM - UniProtAC mapping

Legend

- N1: #input proteins associated to the significant GO term
- N2: #proteins associated to the significant GO term
- P-value: Bonferroni-corrected p-value of Fisher's exact test
- *red*: go terms not related to the input proteins
- *blue*: go terms related to the input proteins (enriched uniquely by network-based method)
- *green*: go terms ancestors of terms enriched with the standard method (enriched uniquely by network-based method)

1 Standard enrichment

GO Term	N1	N2	P-value	Description
GO:0001945	2	9	4.06326e-05	lymph vessel development
GO:0038084	2	26	0.00036671	vascular endothelial growth factor signaling pathway
GO:0048010	2	38	0.000793055	vascular endothelial growth factor receptor signaling pathway
GO:0035924	2	43	0.00101859	cellular response to vascular endothelial growth factor stimulus
GO:0010595	2	55	0.00167473	positive regulation of endothelial cell migration
GO:0001938	2	72	0.0028817	positive regulation of endothelial cell proliferation
GO:0010634	2	83	0.00383588	positive regulation of epithelial cell migration
GO:0001936	2	101	0.00569058	regulation of endothelial cell proliferation
GO:0010594	2	110	0.00675438	regulation of endothelial cell migration
GO:0070374	2	150	0.0125816	positive regulation of ERK1 and ERK2 cascade
GO:0010632	2	155	0.013436	regulation of epithelial cell migration
GO:0050679	2	188	0.0197772	positive regulation of epithelial cell proliferation
GO:0070372	2	231	0.0298659	regulation of ERK1 and ERK2 cascade
GO:0046777	2	268	0.0401971	protein autophosphorylation
GO:0009653	3	2131	0.0481725	anatomical structure morphogenesis

Table 2: Overrepresented GO terms with the standard enrichment

2 Network-based enrichment

GO Term	N1	N2	P-value	Description
GO:0000904	3	542	0.00336186	cell morphogenesis involved in differentiation
GO:0031589	3	556	0.00362967	cell-substrate adhesion
GO:0030947	2	71	0.0105312	regulation of vascular endothelial growth factor receptor signaling pathway
GO:0000902	3	800	0.01083	cell morphogenesis
GO:0050918	2	89	0.0165898	positive chemotaxis
GO:0034446	2	95	0.0189132	substrate adhesion-dependent cell spreading
GO:0098602	3	981	0.0199833	single organism cell adhesion
GO:0030593	2	113	0.0267951	neutrophil chemotaxis
GO:1990266	2	123	0.0317641	neutrophil migration
GO:0010812	2	141	0.0417698	negative regulation of cell-substrate adhesion
GO:0071621	2	142	0.0423656	granulocyte chemotaxis
GO:0097530	2	154	0.049844	granulocyte migration

Table 3: Overrepresented terms with the network-based enrichment. Only terms not detected with the standard method.
