# Supplementary material for: NET-GE: a novel NETwork-based Gene Enrichment for detecting biological processes associated to Mendelian diseases
Source: BMC Genomics. 2015 Jun 18;16(Suppl 8):S6. doi: 10.1186/1471-2164-16-S8-S6 (PMC4480278; doi:10.1186/1471-2164-16-S8-S6)
Supplement: Additional file 3 — Detailed results for the OMIM-derived benchmark set. The archive contains pdf documents listing the enriched terms for each one of the 244 diseases in the OMIM-derived benchmark set. [file 1471-2164-16-S8-S6-S3.tgz › SUPPMAT/OMIM603387.pdf]

#603387 MEGALENCEPHALY-POLYMICROGYRIA-POLYDACTYLY-HYDROCEPHALUS SYNDROME 1;

**OMIM Gene ID**	**HGNC**	**UniProtAC**
171834	PIK3CA	P42336
603157	PIK3R2	O00459

Table 1: OMIM - UniProtAC mapping

Legend

- N1: #input proteins associated to the significant GO term
- N2: #proteins associated to the significant GO term
- P-value: Bonferroni-corrected p-value of Fisher's exact test
- *red*: go terms not related to the input proteins
- *blue*: go terms related to the input proteins (enriched uniquely by network-based method)
- *green*: go terms ancestors of terms enriched with the standard method (enriched uniquely by network-based method)

1 Standard enrichment

GO Term	N1	N2	P-value	Description
GO:0036092	2	33	0.000166796	phosphatidylinositol-3-phosphate biosynthetic process
GO:0050852	2	134	0.002815	T cell receptor signaling pathway
GO:0006661	2	146	0.00334381	phosphatidylinositol biosynthetic process
GO:0050851	2	171	0.00459162	antigen receptor-mediated signaling pathway
GO:0048015	2	182	0.00520321	phosphatidylinositol-mediated signaling
GO:0048017	2	182	0.00520321	inositol lipid-mediated signaling
GO:0008286	2	195	0.00597528	insulin receptor signaling pathway
GO:0002433	2	201	0.00634961	immune response-regulating cell surface receptor signaling pathway involved in phagocytosis
GO:0038094	2	201	0.00634961	Fc-gamma receptor signaling pathway
GO:0038096	2	201	0.00634961	Fc-gamma receptor signaling pathway involved in phagocytosis
GO:0007173	2	202	0.00641311	epidermal growth factor receptor signaling pathway
GO:0002431	2	203	0.00647692	Fc receptor mediated stimulatory signaling pathway
GO:0038127	2	205	0.00660548	ERBB signaling pathway
GO:0008543	2	211	0.00699878	fibroblast growth factor receptor signaling pathway
GO:0044344	2	237	0.00883449	cellular response to fibroblast growth factor stimulus
GO:0071774	2	243	0.00928845	response to fibroblast growth factor
GO:0046474	2	259	0.0105546	glycerophospholipid biosynthetic process
GO:0050900	2	265	0.0110502	leukocyte migration
GO:0032869	2	270	0.0114719	cellular response to insulin stimulus
GO:0048011	2	276	0.0119885	neurotrophin TRK receptor signaling pathway
GO:0038179	2	285	0.0127845	neurotrophin signaling pathway
GO:0046488	2	288	0.0130556	phosphatidylinositol metabolic process
GO:0045017	2	293	0.0135136	glycerolipid biosynthetic process
GO:0038095	2	294	0.0136062	Fc-epsilon receptor signaling pathway
GO:0008654	2	316	0.0157224	phospholipid biosynthetic process
GO:0038093	2	350	0.0192937	Fc receptor signaling pathway
GO:0002429	2	366	0.0211006	immune response-activating cell surface receptor signaling pathway
GO:0071375	2	372	0.0217991	cellular response to peptide hormone stimulus
GO:0032868	2	376	0.022271	response to insulin
GO:1901653	2	391	0.0240858	cellular response to peptide
GO:0006650	2	416	0.0272687	glycerophospholipid metabolic process
GO:0002768	2	486	0.0372305	immune response-regulating cell surface receptor signaling pathway
GO:0007596	2	501	0.0395667	blood coagulation
GO:0050817	2	501	0.0395667	coagulation
GO:0002757	2	508	0.0406811	immune response-activating signal transduction
GO:0007599	2	510	0.0410024	hemostasis
GO:0046486	2	522	0.0429565	glycerolipid metabolic process
GO:0006644	2	531	0.0444521	phospholipid metabolic process
GO:0044028	1	4	0.047689	DNA hypomethylation
GO:0044029	1	4	0.047689	hypomethylation of CpG island

Table 2: Overrepresented GO terms with the standard enrichment

2 Network-based enrichment

GO Term	N1	N2	P-value	Description
GO:0043551	2	88	0.00444933	regulation of phosphatidylinositol 3-kinase activity
GO:0043550	2	113	0.00735513	regulation of lipid kinase activity
GO:0030595	2	281	0.0457254	leukocyte chemotaxis
GO:0002274	2	290	0.0487067	myeloid leukocyte activation

Table 3: Overrepresented terms with the network-based enrichment. Only terms not detected with the standard method.
