# Supplementary material for: NET-GE: a novel NETwork-based Gene Enrichment for detecting biological processes associated to Mendelian diseases
Source: BMC Genomics. 2015 Jun 18;16(Suppl 8):S6. doi: 10.1186/1471-2164-16-S8-S6 (PMC4480278; doi:10.1186/1471-2164-16-S8-S6)
Supplement: Additional file 3 — Detailed results for the OMIM-derived benchmark set. The archive contains pdf documents listing the enriched terms for each one of the 244 diseases in the OMIM-derived benchmark set. [file 1471-2164-16-S8-S6-S3.tgz › SUPPMAT/OMIM603554.pdf]

#603554 OMENN SYNDROME

**OMIM Gene ID**	**HGNC**	**UniProtAC**
179615	RAG1	P15918
179616	RAG2	P55895
605988	DCLRE1C	Q96SD1

Table 1: OMIM - UniProtAC mapping

Legend

- N1: #input proteins associated to the significant GO term
- N2: #proteins associated to the significant GO term
- P-value: Bonferroni-corrected p-value of Fisher's exact test
- *red*: go terms not related to the input proteins
- *blue*: go terms related to the input proteins (enriched uniquely by network-based method)
- *green*: go terms ancestors of terms enriched with the standard method (enriched uniquely by network-based method)

1 Standard enrichment

GO Term	N1	N2	P-value	Description
GO:0030183	3	113	3.94568e-06	B cell differentiation
GO:0042113	3	186	1.7783e-05	B cell activation
GO:0002331	2	8	1.78065e-05	pre-B cell allelic exclusion
GO:0030098	3	263	5.05127e-05	lymphocyte differentiation
GO:0006310	3	294	7.06481e-05	DNA recombination
GO:0002521	3	375	0.000146931	leukocyte differentiation
GO:0033151	2	30	0.000276529	V(D)J recombination
GO:0046649	3	484	0.000316476	lymphocyte activation
GO:0045321	3	579	0.000542356	leukocyte activation
GO:0033077	2	60	0.00112459	T cell differentiation in thymus
GO:0002562	2	63	0.0012408	somatic diversification of immune receptors via germline recombination within a single locus
GO:0016444	2	63	0.0012408	somatic cell DNA recombination
GO:0002200	2	66	0.00136271	somatic diversification of immune receptors
GO:0001775	3	825	0.00157138	cell activation
GO:0030217	2	154	0.00747276	T cell differentiation
GO:0006259	3	1502	0.00949826	DNA metabolic process
GO:0042110	2	319	0.0320786	T cell activation
GO:0002376	3	2446	0.0410524	immune system process
GO:0030154	3	2446	0.0410524	cell differentiation

Table 2: Overrepresented GO terms with the standard enrichment

2 Network-based enrichment

No novel enriched terms
