# Supplementary material for: NET-GE: a novel NETwork-based Gene Enrichment for detecting biological processes associated to Mendelian diseases
Source: BMC Genomics. 2015 Jun 18;16(Suppl 8):S6. doi: 10.1186/1471-2164-16-S8-S6 (PMC4480278; doi:10.1186/1471-2164-16-S8-S6)
Supplement: Additional file 3 — Detailed results for the OMIM-derived benchmark set. The archive contains pdf documents listing the enriched terms for each one of the 244 diseases in the OMIM-derived benchmark set. [file 1471-2164-16-S8-S6-S3.tgz › SUPPMAT/OMIM603896.pdf]

#603896 LEUKOENCEPHALOPATHY WITH VANISHING WHITE MATTER; VWM

**OMIM Gene ID**	**HGNC**	**UniProtAC**
603945	EIF2B5	Q13144
606273	EIF2B3	Q9NR50
606454	EIF2B2	P49770
606686	EIF2B1	Q14232
606687	EIF2B4	Q9UI10

Table 1: OMIM - UniProtAC mapping

Legend

- N1: #input proteins associated to the significant GO term
- N2: #proteins associated to the significant GO term
- P-value: Bonferroni-corrected p-value of Fisher's exact test
- *red*: go terms not related to the input proteins
- *blue*: go terms related to the input proteins (enriched uniquely by network-based method)
- *green*: go terms ancestors of terms enriched with the standard method (enriched uniquely by network-based method)

1 Standard enrichment

GO Term	N1	N2	P-value	Description
GO:0014003	5	38	1.04621e-13	oligodendrocyte development
GO:0032057	4	6	1.17986e-13	negative regulation of translational initiation in response to stress
GO:0032055	4	9	9.91016e-13	negative regulation of translation in response to stress
GO:0043558	4	9	9.91016e-13	regulation of translational initiation in response to stress
GO:0021782	5	66	1.86274e-12	glial cell development
GO:0006446	5	82	5.68716e-12	regulation of translational initiation
GO:0043555	4	14	7.87224e-12	regulation of translation in response to stress
GO:0009408	5	97	1.34326e-11	response to heat
GO:0045947	4	20	3.8098e-11	negative regulation of translational initiation
GO:0009749	5	171	2.39409e-10	response to glucose
GO:0009746	5	183	3.37373e-10	response to hexose
GO:0009266	5	195	4.65064e-10	response to temperature stimulus
GO:0034284	5	197	4.89666e-10	response to monosaccharide
GO:0006413	5	222	8.95062e-10	translational initiation
GO:0009743	5	247	1.53313e-09	response to carbohydrate
GO:0006417	5	357	9.79286e-09	regulation of translation
GO:0017148	4	107	4.05049e-08	negative regulation of translation
GO:0043434	5	567	1.00005e-07	response to peptide hormone
GO:0006412	5	568	1.00893e-07	translation
GO:0010608	5	572	1.04509e-07	posttranscriptional regulation of gene expression
GO:1901652	5	600	1.32826e-07	response to peptide
GO:0010467	5	675	2.39803e-07	gene expression
GO:0048468	5	855	7.84381e-07	cell development
GO:0043547	5	1011	1.81651e-06	positive regulation of GTPase activity
GO:0043087	5	1074	2.45898e-06	regulation of GTPase activity
GO:0033124	5	1084	2.57584e-06	regulation of GTP catabolic process
GO:0010243	5	1094	2.69709e-06	response to organonitrogen compound
GO:0033121	5	1134	3.2286e-06	regulation of purine nucleotide catabolic process
GO:0030811	5	1135	3.24289e-06	regulation of nucleotide catabolic process
GO:0009118	5	1143	3.35901e-06	regulation of nucleoside metabolic process
GO:1901698	5	1186	4.04148e-06	response to nitrogen compound
GO:0009725	5	1273	5.76117e-06	response to hormone
GO:1900542	5	1287	6.08553e-06	regulation of purine nucleotide metabolic process
GO:0006140	5	1290	6.1569e-06	regulation of nucleotide metabolic process
GO:0001541	3	74	9.59483e-06	ovarian follicle development
GO:0051345	5	1431	1.03501e-05	positive regulation of hydrolase activity
GO:0009628	5	1467	1.17212e-05	response to abiotic stimulus
GO:0031329	5	1522	1.40929e-05	regulation of cellular catabolic process
GO:0009894	5	1690	2.38038e-05	regulation of catabolic process
GO:1901700	5	1851	3.75378e-05	response to oxygen-containing compound
GO:0042552	3	129	5.16182e-05	myelination
GO:0051336	5	1982	5.28579e-05	regulation of hydrolase activity
GO:0007272	3	133	5.66013e-05	ensheathment of neurons
GO:0008366	3	133	5.66013e-05	axon ensheathment
GO:0009719	5	2012	5.69854e-05	response to endogenous stimulus
GO:0022602	3	138	6.32677e-05	ovulation cycle process
GO:0043085	5	2150	7.94247e-05	positive regulation of catalytic activity
GO:0032269	4	737	9.44219e-05	negative regulation of cellular protein metabolic process
GO:0032268	5	2272	0.000104692	regulation of cellular protein metabolic process
GO:0044093	5	2479	0.000161962	positive regulation of molecular function
GO:0051248	4	875	0.000187284	negative regulation of protein metabolic process
GO:0051246	5	2954	0.000389373	regulation of protein metabolic process
GO:0019220	5	2977	0.00040478	regulation of phosphate metabolic process
GO:0051174	5	2996	0.000417873	regulation of phosphorus metabolic process
GO:0050790	5	3371	0.000753857	regulation of catalytic activity
GO:0010033	5	3487	0.00089289	response to organic substance
GO:0048869	5	3694	0.0011915	cellular developmental process
GO:0048511	3	391	0.00144493	rhythmic process
GO:0048608	3	406	0.00161717	reproductive structure development
GO:0065009	5	3941	0.00164709	regulation of molecular function

Table 2: Overrepresented GO terms with the standard enrichment

GO Term	N1	N2	P-value	Description
GO:0034645	5	4013	0.00180321	cellular macromolecule biosynthetic process
GO:0006950	5	4134	0.00209212	response to stress
GO:2000113	4	1622	0.00218265	negative regulation of cellular macromolecule biosynthetic process
GO:0048856	5	4289	0.00251508	anatomical structure development
GO:0010558	4	1696	0.00260524	negative regulation of macromolecule biosynthetic process
GO:0009059	5	4344	0.00268061	macromolecule biosynthetic process
GO:0031327	4	1764	0.00304473	negative regulation of cellular biosynthetic process
GO:0009890	4	1786	0.00319806	negative regulation of biosynthetic process
GO:0042221	5	4712	0.00402614	response to chemical
GO:0033554	4	1930	0.0043483	cellular response to stress
GO:0044267	5	5636	0.00985981	cellular protein metabolic process
GO:0010605	4	2452	0.0112052	negative regulation of macromolecule metabolic process
GO:0031324	4	2479	0.0117002	negative regulation of cellular metabolic process
GO:0044249	5	5837	0.0117487	cellular biosynthetic process
GO:1901576	5	6037	0.013905	organic substance biosynthetic process
GO:0009892	4	2679	0.0158895	negative regulation of metabolic process
GO:0009058	5	6217	0.016106	biosynthetic process
GO:0003006	3	922	0.0186276	developmental process involved in reproduction
GO:2000112	5	6579	0.0213759	regulation of cellular macromolecule biosynthetic process
GO:0044767	5	6740	0.0241234	single-organism developmental process
GO:0010556	5	6784	0.0249214	regulation of macromolecule biosynthetic process
GO:0031326	5	6987	0.0288813	regulation of cellular biosynthetic process
GO:0009889	5	7051	0.0302289	regulation of biosynthetic process
GO:0010468	5	7158	0.0325939	regulation of gene expression
GO:0032502	5	7299	0.0359341	developmental process
GO:0019538	5	7743	0.0482803	protein metabolic process
GO:0019219	5	7797	0.0499879	regulation of nucleobase-containing compound metabolic process

Table 3: Overrepresented GO terms with the standard enrichment

2 Network-based enrichment

GO Term	N1	N2	P-value	Description
GO:0043255	3	259	0.00197893	regulation of carbohydrate biosynthetic process
GO:0016071	4	1213	0.00365985	mRNA metabolic process
GO:0044702	5	3664	0.00691833	single organism reproductive process

Table 4: Overrepresented terms with the network-based enrichment. Only terms not detected with the standard method.
