# Supplementary material for: NET-GE: a novel NETwork-based Gene Enrichment for detecting biological processes associated to Mendelian diseases
Source: BMC Genomics. 2015 Jun 18;16(Suppl 8):S6. doi: 10.1186/1471-2164-16-S8-S6 (PMC4480278; doi:10.1186/1471-2164-16-S8-S6)
Supplement: Additional file 3 — Detailed results for the OMIM-derived benchmark set. The archive contains pdf documents listing the enriched terms for each one of the 244 diseases in the OMIM-derived benchmark set. [file 1471-2164-16-S8-S6-S3.tgz › SUPPMAT/OMIM603932.pdf]

#603932 INTERVERTEBRAL DISC DISEASE; IDD

OMIM Gene ID	HGNC	UniProtAC
120260	COL9A2	Q14055
120270	COL9A3	Q14050
120280	COL11A1	P12107
188061	THBS2	P35442
603489	CILP	O75339
608135	ASPN	Q9BXN1

Table 1: OMIM - UniProtAC mapping

Legend

- N1: #input proteins associated to the significant GO term
- N2: #proteins associated to the significant GO term
- P-value: Bonferroni-corrected p-value of Fisher's exact test
- *red*: go terms not related to the input proteins
- *blue*: go terms related to the input proteins (enriched uniquely by network-based method)
- *green*: go terms ancestors of terms enriched with the standard method (enriched uniquely by network-based method)

1 Standard enrichment

GO Term	N1	N2	P-value	Description
GO:0030574	3	78	2.38358e-05	collagen catabolic process
GO:0044243	3	84	2.98434e-05	multicellular organismal catabolic process
GO:0032963	3	96	4.47184e-05	collagen metabolic process
GO:0044259	3	105	5.86396e-05	multicellular organismal macromolecule metabolic process
GO:0044236	3	112	7.12661e-05	multicellular organismal metabolic process
GO:0022617	3	117	8.13133e-05	extracellular matrix disassembly
GO:0022411	3	404	0.00335173	cellular component disassembly
GO:0030198	3	486	0.00581353	extracellular matrix organization
GO:0043062	3	487	0.0058492	extracellular structure organization
GO:0070171	1	2	0.0448266	negative regulation of tooth mineralization

Table 2: Overrepresented GO terms with the standard enrichment

2 Network-based enrichment

No novel enriched terms
