# Supplementary material for: NET-GE: a novel NETwork-based Gene Enrichment for detecting biological processes associated to Mendelian diseases
Source: BMC Genomics. 2015 Jun 18;16(Suppl 8):S6. doi: 10.1186/1471-2164-16-S8-S6 (PMC4480278; doi:10.1186/1471-2164-16-S8-S6)
Supplement: Additional file 3 — Detailed results for the OMIM-derived benchmark set. The archive contains pdf documents listing the enriched terms for each one of the 244 diseases in the OMIM-derived benchmark set. [file 1471-2164-16-S8-S6-S3.tgz › SUPPMAT/OMIM604091.pdf]

#604091 HYPOALPHALIPOPROTEINEMIA, PRIMARY

OMIM Gene ID	HGNC	UniProtAC
107680	APOA1	P02647
600046	ABCA1	O95477

Table 1: OMIM - UniProtAC mapping

Legend

- N1: #input proteins associated to the significant GO term
- N2: #proteins associated to the significant GO term
- P-value: Bonferroni-corrected p-value of Fisher's exact test
- *red*: go terms not related to the input proteins
- *blue*: go terms related to the input proteins (enriched uniquely by network-based method)
- *green*: go terms ancestors of terms enriched with the standard method (enriched uniquely by network-based method)

1 Standard enrichment

GO Term	N1	N2	P-value	Description
GO:0032489	2	4	4.3131e-06	regulation of Cdc42 protein signal transduction
GO:0034380	2	6	1.07828e-05	high-density lipoprotein particle assembly
GO:0055091	2	10	3.23483e-05	phospholipid homeostasis
GO:0033700	2	12	4.74441e-05	phospholipid efflux
GO:0034377	2	17	9.77638e-05	plasma lipoprotein particle assembly
GO:0043691	2	17	9.77638e-05	reverse cholesterol transport
GO:0065005	2	19	0.000122924	protein-lipid complex assembly
GO:0033344	2	31	0.000334265	cholesterol efflux
GO:0071827	2	40	0.000560701	plasma lipoprotein particle organization
GO:0071825	2	42	0.000618931	protein-lipid complex subunit organization
GO:0032371	2	48	0.000810865	regulation of sterol transport
GO:0032374	2	48	0.000810865	regulation of cholesterol transport
GO:0055081	2	51	0.000916536	anion homeostasis
GO:0015918	2	66	0.00154193	sterol transport
GO:0030301	2	66	0.00154193	cholesterol transport
GO:0035023	2	73	0.00188914	regulation of Rho protein signal transduction
GO:0042157	2	87	0.00268922	lipoprotein metabolic process
GO:0042632	2	95	0.00320967	cholesterol homeostasis
GO:0055092	2	96	0.00327796	sterol homeostasis
GO:0015914	2	102	0.0037028	phospholipid transport
GO:0032368	2	112	0.00446838	regulation of lipid transport
GO:0015748	2	134	0.00640568	organophosphate ester transport
GO:0015850	2	139	0.00689449	organic hydroxy compound transport
GO:0008203	2	140	0.00699443	cholesterol metabolic process
GO:0055088	2	140	0.00699443	lipid homeostasis
GO:0016125	2	158	0.00891592	sterol metabolic process
GO:0046578	2	203	0.0147386	regulation of Ras protein signal transduction
GO:0060354	1	1	0.0271309	negative regulation of cell adhesion molecule production
GO:0007584	2	307	0.0337651	response to nutrient
GO:0008202	2	325	0.0378475	steroid metabolic process
GO:0006869	2	348	0.0434028	lipid transport

Table 2: Overrepresented GO terms with the standard enrichment

2 Network-based enrichment

GO Term	N1	N2	P-value	Description
GO:0018158	2	13	0.000120459	protein oxidation
GO:0042362	2	27	0.000542065	fat-soluble vitamin biosynthetic process
GO:0071397	2	36	0.000972935	cellular response to cholesterol
GO:0036315	2	43	0.00139454	cellular response to sterol
GO:0010874	2	44	0.00146095	regulation of cholesterol efflux
GO:0032369	2	51	0.00196904	negative regulation of lipid transport
GO:0035025	2	54	0.00220995	positive regulation of Rho protein signal transduction
GO:0009110	2	55	0.00229335	vitamin biosynthetic process
GO:1900026	2	55	0.00229335	positive regulation of substrate adhesion-dependent cell spreading
GO:0070723	2	56	0.00237829	response to cholesterol
GO:0001935	2	58	0.00255281	endothelial cell proliferation
GO:0034367	2	60	0.00273349	macromolecular complex remodeling
GO:0034368	2	60	0.00273349	protein-lipid complex remodeling
GO:0034369	2	60	0.00273349	plasma lipoprotein particle remodeling
GO:0036314	2	65	0.00321223	response to sterol
GO:1900024	2	70	0.0037296	regulation of substrate adhesion-dependent cell spreading
GO:0016126	2	77	0.00451876	sterol biosynthetic process
GO:0001960	2	96	0.00704221	negative regulation of cytokine-mediated signaling pathway
GO:0050710	2	100	0.00764451	negative regulation of cytokine secretion
GO:0032720	2	109	0.00909003	negative regulation of tumor necrosis factor production
GO:0060761	2	111	0.0094282	negative regulation of response to cytokine stimulus
GO:0051057	2	134	0.0137616	positive regulation of small GTPase mediated signal transduction
GO:0030819	2	139	0.0148118	positive regulation of cAMP biosynthetic process
GO:0030816	2	166	0.0211498	positive regulation of cAMP metabolic process
GO:0050709	2	170	0.0221845	negative regulation of protein secretion
GO:0010770	2	177	0.0240547	positive regulation of cell morphogenesis involved in differentiation
GO:0030804	2	180	0.0248794	positive regulation of cyclic nucleotide biosynthetic process
GO:0006767	2	190	0.0277287	water-soluble vitamin metabolic process
GO:0030810	2	192	0.0283171	positive regulation of nucleotide biosynthetic process
GO:1900373	2	192	0.0283171	positive regulation of purine nucleotide biosynthetic process
GO:0030817	2	212	0.0345408	regulation of cAMP biosynthetic process
GO:0030801	2	216	0.0358597	positive regulation of cyclic nucleotide metabolic process
GO:0097306	2	219	0.0368651	cellular response to alcohol
GO:0050821	2	232	0.0413822	protein stabilization
GO:0035821	2	242	0.0450347	modification of morphology or physiology of other organism
GO:0072593	2	252	0.0488415	reactive oxygen species metabolic process

Table 3: Overrepresented terms with the network-based enrichment. Only terms not detected with the standard method.
