# Supplementary material for: NET-GE: a novel NETwork-based Gene Enrichment for detecting biological processes associated to Mendelian diseases
Source: BMC Genomics. 2015 Jun 18;16(Suppl 8):S6. doi: 10.1186/1471-2164-16-S8-S6 (PMC4480278; doi:10.1186/1471-2164-16-S8-S6)
Supplement: Additional file 3 — Detailed results for the OMIM-derived benchmark set. The archive contains pdf documents listing the enriched terms for each one of the 244 diseases in the OMIM-derived benchmark set. [file 1471-2164-16-S8-S6-S3.tgz › SUPPMAT/OMIM604229.pdf]

#604229 PETERS ANOMALY

OMIM Gene ID	HGNC	UniProtAC
601542	PITX2	Q99697
601771	CYP1B1	Q16678
607108	PAX6	P26367

Table 1: OMIM - UniProtAC mapping

Legend

- N1: #input proteins associated to the significant GO term
- N2: #proteins associated to the significant GO term
- P-value: Bonferroni-corrected p-value of Fisher's exact test
- *red*: go terms not related to the input proteins
- *blue*: go terms related to the input proteins (enriched uniquely by network-based method)
- *green*: go terms ancestors of terms enriched with the standard method (enriched uniquely by network-based method)

1 Standard enrichment

GO Term	N1	N2	P-value	Description
GO:0061072	2	9	7.01973e-05	iris morphogenesis
GO:0030334	3	733	0.00337784	regulation of cell migration
GO:2000145	3	786	0.00416595	regulation of cell motility
GO:0051270	3	843	0.00514092	regulation of cellular component movement
GO:0040012	3	864	0.00553526	regulation of locomotion
GO:0016477	3	1046	0.00982778	cell migration
GO:0048514	2	104	0.0104263	blood vessel morphogenesis
GO:0009913	2	105	0.0106285	epidermal cell differentiation
GO:0048870	3	1136	0.012592	cell motility
GO:0001654	2	128	0.0158157	eye development
GO:0009798	2	132	0.0168224	axis specification
GO:0040011	3	1284	0.018188	locomotion
GO:0007423	2	187	0.0338045	sensory organ development
GO:0021763	1	1	0.0368015	subthalamic nucleus development
GO:0060127	1	1	0.0368015	prolactin secreting cell differentiation
GO:0060578	1	1	0.0368015	superior vena cava morphogenesis
GO:0048562	2	199	0.0382864	embryonic organ morphogenesis
GO:0001667	2	211	0.0430464	ameboidal cell migration
GO:0001764	2	213	0.0438667	neuron migration
GO:0007601	2	214	0.0442797	visual perception
GO:0050953	2	218	0.0459512	sensory perception of light stimulus

Table 2: Overrepresented GO terms with the standard enrichment

2 Network-based enrichment

GO Term	N1	N2	P-value	Description
GO:0021546	2	19	0.00100545	rhombomere development
GO:0043433	3	432	0.00235601	negative regulation of sequence-specific DNA binding transcription factor activity
GO:0030238	2	34	0.0032976	male sex determination
GO:0048546	2	51	0.007492	digestive tract morphogenesis
GO:0060412	2	54	0.00840816	ventricular septum morphogenesis
GO:0030336	3	660	0.00842177	negative regulation of cell migration
GO:2000146	3	688	0.00954153	negative regulation of cell motility
GO:0045765	3	715	0.0107113	regulation of angiogenesis
GO:1901342	3	774	0.0135921	regulation of vasculature development
GO:0007530	2	70	0.0141853	sex determination
GO:0040013	3	840	0.0173794	negative regulation of locomotion
GO:0021983	2	85	0.0209633	pituitary gland development
GO:0001525	3	949	0.0250711	angiogenesis
GO:0008045	2	96	0.0267708	motor neuron axon guidance
GO:0060563	2	104	0.0314388	neuroepithelial cell differentiation
GO:0030335	3	1024	0.0315047	positive regulation of cell migration
GO:2000147	3	1042	0.0331972	positive regulation of cell motility
GO:0051272	3	1060	0.0349491	positive regulation of cellular component movement
GO:0051090	3	1091	0.038109	regulation of sequence-specific DNA binding transcription factor activity
GO:0030198	3	1111	0.0402454	extracellular matrix organization
GO:0010769	3	1115	0.0406822	regulation of cell morphogenesis involved in differentiation
GO:0043062	3	1116	0.0407918	extracellular structure organization
GO:0001569	2	119	0.0411997	patterning of blood vessels
GO:0040017	3	1124	0.0416762	positive regulation of locomotion

Table 3: Overrepresented terms with the network-based enrichment. Only terms not detected with the standard method.
