# Supplementary material for: NET-GE: a novel NETwork-based Gene Enrichment for detecting biological processes associated to Mendelian diseases
Source: BMC Genomics. 2015 Jun 18;16(Suppl 8):S6. doi: 10.1186/1471-2164-16-S8-S6 (PMC4480278; doi:10.1186/1471-2164-16-S8-S6)
Supplement: Additional file 3 — Detailed results for the OMIM-derived benchmark set. The archive contains pdf documents listing the enriched terms for each one of the 244 diseases in the OMIM-derived benchmark set. [file 1471-2164-16-S8-S6-S3.tgz › SUPPMAT/OMIM604271.pdf]

#604271 SHORT STATURE, IDIOPATHIC, AUTOSOMAL

OMIM Gene ID	HGNC	UniProtAC
600946	GHR	P10912
601898	GHSR	Q92847

Table 1: OMIM - UniProtAC mapping

Legend

- N1: #input proteins associated to the significant GO term
- N2: #proteins associated to the significant GO term
- P-value: Bonferroni-corrected p-value of Fisher's exact test
- *red*: go terms not related to the input proteins
- *blue*: go terms related to the input proteins (enriched uniquely by network-based method)
- *green*: go terms ancestors of terms enriched with the standard method (enriched uniquely by network-based method)

1 Standard enrichment

GO Term	N1	N2	P-value	Description
GO:0040018	2	61	0.000773368	positive regulation of multicellular organism growth
GO:0040014	2	123	0.00317079	regulation of multicellular organism growth
GO:0045927	2	244	0.0125286	positive regulation of growth
GO:0071375	2	372	0.0291623	cellular response to peptide hormone stimulus
GO:0043134	1	2	0.0318997	regulation of hindgut contraction
GO:1901653	2	391	0.0322214	cellular response to peptide

Table 2: Overrepresented GO terms with the standard enrichment

2 Network-based enrichment

No novel enriched terms
