# Supplementary material for: NET-GE: a novel NETwork-based Gene Enrichment for detecting biological processes associated to Mendelian diseases
Source: BMC Genomics. 2015 Jun 18;16(Suppl 8):S6. doi: 10.1186/1471-2164-16-S8-S6 (PMC4480278; doi:10.1186/1471-2164-16-S8-S6)
Supplement: Additional file 3 — Detailed results for the OMIM-derived benchmark set. The archive contains pdf documents listing the enriched terms for each one of the 244 diseases in the OMIM-derived benchmark set. [file 1471-2164-16-S8-S6-S3.tgz › SUPPMAT/OMIM604302.pdf]

#604302 RHEUMATOID ARTHRITIS, SYSTEMIC JUVENILE

**OMIM Gene ID**	**HGNC**	**UniProtAC**
147620	IL6	P05231
153620	MIF	P14174

Table 1: OMIM - UniProtAC mapping

Legend

- N1: #input proteins associated to the significant GO term
- N2: #proteins associated to the significant GO term
- P-value: Bonferroni-corrected p-value of Fisher's exact test
- *red*: go terms not related to the input proteins
- *blue*: go terms related to the input proteins (enriched uniquely by network-based method)
- *green*: go terms ancestors of terms enriched with the standard method (enriched uniquely by network-based method)

1 Standard enrichment

GO Term	N1	N2	P-value	Description
GO:0032722	2	41	0.000717247	positive regulation of chemokine production
GO:0032642	2	71	0.00217362	regulation of chemokine production
GO:0033138	2	86	0.00319701	positive regulation of peptidyl-serine phosphorylation
GO:0050871	2	87	0.00327223	positive regulation of B cell activation
GO:0033135	2	120	0.00624533	regulation of peptidyl-serine phosphorylation
GO:0050864	2	130	0.00733433	regulation of B cell activation
GO:0002700	2	137	0.00814865	regulation of production of molecular mediator of immune response
GO:0050671	2	142	0.00875658	positive regulation of lymphocyte proliferation
GO:0032946	2	145	0.00913181	positive regulation of mononuclear cell proliferation
GO:0070374	2	150	0.00977475	positive regulation of ERK1 and ERK2 cascade
GO:0070665	2	150	0.00977475	positive regulation of leukocyte proliferation
GO:0050707	2	156	0.0105751	regulation of cytokine secretion
GO:0050714	2	162	0.0114069	positive regulation of protein secretion
GO:0050731	2	193	0.0162063	positive regulation of peptidyl-tyrosine phosphorylation
GO:0070372	2	231	0.0232363	regulation of ERK1 and ERK2 cascade
GO:0050670	2	238	0.0246691	regulation of lymphocyte proliferation
GO:0032944	2	241	0.0252962	regulation of mononuclear cell proliferation
GO:0032103	2	245	0.0261446	positive regulation of response to external stimulus
GO:0070663	2	248	0.0267902	regulation of leukocyte proliferation
GO:0050730	2	266	0.0308287	regulation of peptidyl-tyrosine phosphorylation
GO:0002699	2	269	0.0315293	positive regulation of immune effector process
GO:0050708	2	273	0.0324757	regulation of protein secretion
GO:0061078	1	1	0.0330128	positive regulation of prostaglandin secretion involved in immune response

Table 2: Overrepresented GO terms with the standard enrichment

2 Network-based enrichment

GO Term	N1	N2	P-value	Description
GO:0031666	2	14	0.000295009	positive regulation of lipopolysaccharide-mediated signaling pathway
GO:0070207	2	37	0.00215909	protein homotrimerization
GO:0031664	2	49	0.00381242	regulation of lipopolysaccharide-mediated signaling pathway
GO:0002833	2	62	0.00613036	positive regulation of response to biotic stimulus
GO:0043030	2	79	0.00998817	regulation of macrophage activation
GO:0070229	2	80	0.0102443	negative regulation of lymphocyte apoptotic process
GO:0070206	2	87	0.0121278	protein trimerization
GO:0050918	2	89	0.0126951	positive chemotaxis
GO:0030890	2	90	0.0129837	positive regulation of B cell proliferation
GO:0046456	2	93	0.0138687	icosanoid biosynthetic process
GO:1901570	2	93	0.0138687	fatty acid derivative biosynthetic process
GO:0006636	2	119	0.0227611	unsaturated fatty acid biosynthetic process
GO:2000107	2	136	0.0297603	negative regulation of leukocyte apoptotic process
GO:0032370	2	143	0.0329147	positive regulation of lipid transport
GO:0030888	2	153	0.0376964	regulation of B cell proliferation
GO:0032890	2	156	0.0391941	regulation of organic acid transport
GO:0070228	2	172	0.0476748	regulation of lymphocyte apoptotic process

Table 3: Overrepresented terms with the network-based enrichment. Only terms not detected with the standard method.
