# Supplementary material for: NET-GE: a novel NETwork-based Gene Enrichment for detecting biological processes associated to Mendelian diseases
Source: BMC Genomics. 2015 Jun 18;16(Suppl 8):S6. doi: 10.1186/1471-2164-16-S8-S6 (PMC4480278; doi:10.1186/1471-2164-16-S8-S6)
Supplement: Additional file 3 — Detailed results for the OMIM-derived benchmark set. The archive contains pdf documents listing the enriched terms for each one of the 244 diseases in the OMIM-derived benchmark set. [file 1471-2164-16-S8-S6-S3.tgz › SUPPMAT/OMIM604571.pdf]

#604571 BARE LYMPHOCYTE SYNDROME, TYPE I

OMIM Gene ID	HGNC	UniProtAC
170260	TAP1	Q03518
170261	TAP2	Q03519
601962	TAPBP	O15533

Table 1: OMIM - UniProtAC mapping

Legend

- N1: #input proteins associated to the significant GO term
- N2: #proteins associated to the significant GO term
- P-value: Bonferroni-corrected p-value of Fisher's exact test
- *red*: go terms not related to the input proteins
- *blue*: go terms related to the input proteins (enriched uniquely by network-based method)
- *green*: go terms ancestors of terms enriched with the standard method (enriched uniquely by network-based method)

1 Standard enrichment

GO Term	N1	N2	P-value	Description
GO:0019885	3	14	6.33728e-09	antigen processing and presentation of endogenous peptide antigen via MHC class I
GO:0002483	3	15	7.92159e-09	antigen processing and presentation of endogenous peptide antigen
GO:0019883	3	17	1.18389e-08	antigen processing and presentation of endogenous antigen
GO:0046967	2	2	6.57075e-07	cytosol to ER transport
GO:0015833	3	100	2.81521e-06	peptide transport
GO:0019060	2	5	6.57039e-06	intracellular transport of viral protein in host cell
GO:0030581	2	5	6.57039e-06	symbiont intracellular protein transport in host
GO:0051708	2	5	6.57039e-06	intracellular protein transport in other organism involved in symbiotic interaction
GO:0042886	3	139	7.62544e-06	amide transport
GO:0002479	3	141	7.9618e-06	antigen processing and presentation of exogenous peptide antigen via MHC class I, TAP-dependent
GO:0042590	3	146	8.84571e-06	antigen processing and presentation of exogenous peptide antigen via MHC class I
GO:0006858	2	7	1.37973e-05	extracellular transport
GO:0002474	3	188	1.89741e-05	antigen processing and presentation of peptide antigen via MHC class I
GO:0002478	3	254	4.69899e-05	antigen processing and presentation of exogenous peptide antigen
GO:0019884	3	256	4.81132e-05	antigen processing and presentation of exogenous antigen
GO:0048002	3	288	6.85946e-05	antigen processing and presentation of peptide antigen
GO:0019882	3	376	0.000153017	antigen processing and presentation
GO:0044766	2	32	0.000325736	multi-organism transport
GO:0046794	2	32	0.000325736	transport of virus
GO:0075733	2	32	0.000325736	intracellular transport of virus
GO:1902583	2	32	0.000325736	multi-organism intracellular transport
GO:0071705	3	691	0.000953227	nitrogen compound transport
GO:0051701	2	104	0.00351295	interaction with host
GO:1902582	3	1419	0.00827329	single-organism intracellular transport
GO:0050822	1	1	0.0123996	peptide stabilization
GO:0050823	1	1	0.0123996	peptide antigen stabilization
GO:0046907	3	1995	0.0230052	intracellular transport
GO:0002485	1	3	0.037197	antigen processing and presentation of endogenous peptide antigen via MHC class I via ER pathway, TAP-de
GO:0002488	1	3	0.037197	antigen processing and presentation of endogenous peptide antigen via MHC class Ib via ER pathway
GO:0002489	1	3	0.037197	antigen processing and presentation of endogenous peptide antigen via MHC class Ib via ER pathway, TAP-
GO:0002591	1	3	0.037197	positive regulation of antigen processing and presentation of peptide antigen via MHC class I
GO:0046968	1	3	0.037197	peptide antigen transport
GO:0002376	3	2446	0.0424117	immune system process
GO:0002476	1	4	0.0495946	antigen processing and presentation of endogenous peptide antigen via MHC class Ib
GO:0002589	1	4	0.0495946	regulation of antigen processing and presentation of peptide antigen via MHC class I

Table 2: Overrepresented GO terms with the standard enrichment

2 Network-based enrichment

GO Term	N1	N2	P-value	Description
GO:0002477	2	7	2.31575e-05	antigen processing and presentation of exogenous peptide antigen via MHC class Ib
GO:0002481	2	7	2.31575e-05	antigen processing and presentation of exogenous protein antigen via MHC class Ib, TAP-dependent
GO:0002428	2	9	3.9697e-05	antigen processing and presentation of peptide antigen via MHC class Ib
GO:0002484	2	9	3.9697e-05	antigen processing and presentation of endogenous peptide antigen via MHC class I via ER pathway
GO:0002475	2	15	0.000115769	antigen processing and presentation via MHC class Ib
GO:0006886	3	2070	0.0488688	intracellular protein transport

Table 3: Overrepresented terms with the network-based enrichment. Only terms not detected with the standard method.
