# Supplementary material for: NET-GE: a novel NETwork-based Gene Enrichment for detecting biological processes associated to Mendelian diseases
Source: BMC Genomics. 2015 Jun 18;16(Suppl 8):S6. doi: 10.1186/1471-2164-16-S8-S6 (PMC4480278; doi:10.1186/1471-2164-16-S8-S6)
Supplement: Additional file 3 — Detailed results for the OMIM-derived benchmark set. The archive contains pdf documents listing the enriched terms for each one of the 244 diseases in the OMIM-derived benchmark set. [file 1471-2164-16-S8-S6-S3.tgz › SUPPMAT/OMIM605027.pdf]

#605027 LYMPHOMA, NON-HODGKIN, FAMILIAL

OMIM Gene ID	HGNC	UniProtAC
170280	PRF1	P14222
601762	CASP10	Q92851
603615	RAD54L	Q92698
604289	RAD54B	Q9Y620

Table 1: OMIM - UniProtAC mapping

Legend

- N1: #input proteins associated to the significant GO term
- N2: #proteins associated to the significant GO term
- P-value: Bonferroni-corrected p-value of Fisher's exact test
- *red*: go terms not related to the input proteins
- *blue*: go terms related to the input proteins (enriched uniquely by network-based method)
- *green*: go terms ancestors of terms enriched with the standard method (enriched uniquely by network-based method)

1 Standard enrichment

GO Term	N1	N2	P-value	Description
GO:0000724	2	89	0.00552505	double-strand break repair via homologous recombination
GO:0000725	2	90	0.00565041	recombinational repair
GO:0006302	2	175	0.0214155	double-strand break repair
GO:0006950	4	4134	0.0241482	response to stress
GO:0002357	1	2	0.0356078	defense response to tumor cell
GO:0002418	1	2	0.0356078	immune response to tumor cell

Table 2: Overrepresented GO terms with the standard enrichment

2 Network-based enrichment

No novel enriched terms
