# Supplementary material for: NET-GE: a novel NETwork-based Gene Enrichment for detecting biological processes associated to Mendelian diseases
Source: BMC Genomics. 2015 Jun 18;16(Suppl 8):S6. doi: 10.1186/1471-2164-16-S8-S6 (PMC4480278; doi:10.1186/1471-2164-16-S8-S6)
Supplement: Additional file 3 — Detailed results for the OMIM-derived benchmark set. The archive contains pdf documents listing the enriched terms for each one of the 244 diseases in the OMIM-derived benchmark set. [file 1471-2164-16-S8-S6-S3.tgz › SUPPMAT/OMIM605899.pdf]

#605899 GLYCINE ENCEPHALOPATHY; GCE

OMIM Gene ID	HGNC	UniProtAC
238300	GLDC	P23378
238310	AMT	P48728
238330	GCSH	P23434

Table 1: OMIM - UniProtAC mapping

Legend

- N1: #input proteins associated to the significant GO term
- N2: #proteins associated to the significant GO term
- P-value: Bonferroni-corrected p-value of Fisher's exact test
- *red*: go terms not related to the input proteins
- *blue*: go terms related to the input proteins (enriched uniquely by network-based method)
- *green*: go terms ancestors of terms enriched with the standard method (enriched uniquely by network-based method)

1 Standard enrichment

GO Term	N1	N2	P-value	Description
GO:0006546	3	22	5.84355e-09	glycine catabolic process
GO:0009071	3	30	1.54057e-08	serine family amino acid catabolic process
GO:0006544	3	58	1.17083e-07	glycine metabolic process
GO:0009069	3	84	3.61556e-07	serine family amino acid metabolic process
GO:1901606	3	136	1.5559e-06	alpha-amino acid catabolic process
GO:0009063	3	173	3.21792e-06	cellular amino acid catabolic process
GO:0016054	3	278	1.34412e-05	organic acid catabolic process
GO:0046395	3	278	1.34412e-05	carboxylic acid catabolic process
GO:0044282	3	358	2.87743e-05	small molecule catabolic process
GO:1901605	3	396	3.89756e-05	alpha-amino acid metabolic process
GO:0006520	3	839	0.000372164	cellular amino acid metabolic process
GO:1901565	3	1431	0.00184932	organonitrogen compound catabolic process
GO:0019752	3	1590	0.00253732	carboxylic acid metabolic process
GO:0043436	3	1732	0.00328016	oxoacid metabolic process
GO:0006082	3	1753	0.00340099	organic acid metabolic process
GO:0019464	1	2	0.00540484	glycine decarboxylation via glycine cleavage system
GO:0044712	3	2063	0.00554458	single-organism catabolic process
GO:0044248	3	2821	0.0141825	cellular catabolic process
GO:1901575	3	3074	0.0183523	organic substance catabolic process
GO:1901564	3	3152	0.0197856	organonitrogen compound metabolic process
GO:0032259	2	543	0.0208722	methylation
GO:0009056	3	3347	0.0236909	catabolic process

Table 2: Overrepresented GO terms with the standard enrichment

2 Network-based enrichment

GO Term	N1	N2	P-value	Description
GO:0006576	2	355	0.0495821	cellular biogenic amine metabolic process
GO:0044106	2	355	0.0495821	cellular amine metabolic process

Table 3: Overrepresented terms with the network-based enrichment. Only terms not detected with the standard method.
