# Supplementary material for: NET-GE: a novel NETwork-based Gene Enrichment for detecting biological processes associated to Mendelian diseases
Source: BMC Genomics. 2015 Jun 18;16(Suppl 8):S6. doi: 10.1186/1471-2164-16-S8-S6 (PMC4480278; doi:10.1186/1471-2164-16-S8-S6)
Supplement: Additional file 3 — Detailed results for the OMIM-derived benchmark set. The archive contains pdf documents listing the enriched terms for each one of the 244 diseases in the OMIM-derived benchmark set. [file 1471-2164-16-S8-S6-S3.tgz › SUPPMAT/OMIM606054.pdf]

#606054 PROPIONIC ACIDEMIA

OMIM Gene ID	HGNC	UniProtAC
232000	PCCA	P05165
232050	PCCB	P05166

Table 1: OMIM - UniProtAC mapping

Legend

- N1: #input proteins associated to the significant GO term
- N2: #proteins associated to the significant GO term
- P-value: Bonferroni-corrected p-value of Fisher's exact test
- *red*: go terms not related to the input proteins
- *blue*: go terms related to the input proteins (enriched uniquely by network-based method)
- *green*: go terms ancestors of terms enriched with the standard method (enriched uniquely by network-based method)

1 Standard enrichment

GO Term	N1	N2	P-value	Description
GO:0019626	2	8	1.80836e-06	short-chain fatty acid catabolic process
GO:0006768	2	13	5.03755e-06	biotin metabolic process
GO:0046459	2	19	1.10439e-05	short-chain fatty acid metabolic process
GO:0006635	2	56	9.94598e-05	fatty acid beta-oxidation
GO:0019395	2	80	0.000204086	fatty acid oxidation
GO:0034440	2	81	0.000209253	lipid oxidation
GO:0009062	2	83	0.00021978	fatty acid catabolic process
GO:0072329	2	106	0.000359411	monocarboxylic acid catabolic process
GO:0006767	2	109	0.000380143	water-soluble vitamin metabolic process
GO:0006766	2	148	0.000702549	vitamin metabolic process
GO:0044242	2	188	0.00113526	cellular lipid catabolic process
GO:0043603	2	260	0.00217455	cellular amide metabolic process
GO:0030258	2	266	0.00227627	lipid modification
GO:0016054	2	278	0.00248669	organic acid catabolic process
GO:0046395	2	278	0.00248669	carboxylic acid catabolic process
GO:0016042	2	323	0.00335858	lipid catabolic process
GO:0044282	2	358	0.00412712	small molecule catabolic process
GO:0006631	2	421	0.00570989	fatty acid metabolic process
GO:0051186	2	461	0.00684788	cofactor metabolic process
GO:0006790	2	463	0.0069075	sulfur compound metabolic process
GO:0032787	2	767	0.0189723	monocarboxylic acid metabolic process

Table 2: Overrepresented GO terms with the standard enrichment

2 Network-based enrichment

No novel enriched terms
