# Supplementary material for: NET-GE: a novel NETwork-based Gene Enrichment for detecting biological processes associated to Mendelian diseases
Source: BMC Genomics. 2015 Jun 18;16(Suppl 8):S6. doi: 10.1186/1471-2164-16-S8-S6 (PMC4480278; doi:10.1186/1471-2164-16-S8-S6)
Supplement: Additional file 3 — Detailed results for the OMIM-derived benchmark set. The archive contains pdf documents listing the enriched terms for each one of the 244 diseases in the OMIM-derived benchmark set. [file 1471-2164-16-S8-S6-S3.tgz › SUPPMAT/OMIM606176.pdf]

#606176 DIABETES MELLITUS, PERMANENT NEONATAL; PNDM

**OMIM Gene ID**	**HGNC**	**UniProtAC**
138079	GCK	P35557
176730	INS	P01308
600509	ABCC8	Q09428
600937	KCNJ11	Q14654

Table 1: OMIM - UniProtAC mapping

Legend

- N1: #input proteins associated to the significant GO term
- N2: #proteins associated to the significant GO term
- P-value: Bonferroni-corrected p-value of Fisher's exact test
- *red*: go terms not related to the input proteins
- *blue*: go terms related to the input proteins (enriched uniquely by network-based method)
- *green*: go terms ancestors of terms enriched with the standard method (enriched uniquely by network-based method)

1 Standard enrichment

GO Term	N1	N2	P-value	Description
GO:0006112	4	185	3.28559e-07	energy reserve metabolic process
GO:0015980	4	231	8.03914e-07	energy derivation by oxidation of organic compounds
GO:0050796	4	258	1.25439e-06	regulation of insulin secretion
GO:0090276	4	279	1.71846e-06	regulation of peptide hormone secretion
GO:0002791	4	284	1.8457e-06	regulation of peptide secretion
GO:0090087	4	286	1.89852e-06	regulation of peptide transport
GO:0046883	4	336	3.62807e-06	regulation of hormone secretion
GO:0006091	4	531	2.27804e-05	generation of precursor metabolites and energy
GO:0051046	4	829	0.000135885	regulation of secretion
GO:0007154	4	1103	0.000426617	cell communication
GO:0045721	2	14	0.000450562	negative regulation of gluconeogenesis
GO:0045821	2	14	0.000450562	positive regulation of glycolytic process
GO:0006006	3	225	0.000489528	glucose metabolic process
GO:0051048	3	225	0.000489528	negative regulation of secretion
GO:0045725	2	15	0.000519861	positive regulation of glycogen biosynthetic process
GO:0070875	2	17	0.000673295	positive regulation of glycogen metabolic process
GO:0033500	3	264	0.000791707	carbohydrate homeostasis
GO:0042593	3	264	0.000791707	glucose homeostasis
GO:0060341	4	1363	0.000995796	regulation of cellular localization
GO:0019318	3	321	0.00142448	hexose metabolic process
GO:0005975	4	1491	0.00142647	carbohydrate metabolic process
GO:0005996	3	362	0.00204348	monosaccharide metabolic process
GO:0006110	2	32	0.00245425	regulation of glycolytic process
GO:0005979	2	39	0.00366562	regulation of glycogen biosynthetic process
GO:0010962	2	39	0.00366562	regulation of glucan biosynthetic process
GO:0010907	2	40	0.00385841	positive regulation of glucose metabolic process
GO:0031018	2	42	0.00425879	endocrine pancreas development
GO:0043470	2	42	0.00425879	regulation of carbohydrate catabolic process
GO:0043471	2	42	0.00425879	regulation of cellular carbohydrate catabolic process
GO:0010677	2	44	0.0046789	negative regulation of cellular carbohydrate metabolic process
GO:0032885	2	45	0.00489635	regulation of polysaccharide biosynthetic process
GO:0051051	3	493	0.00515948	negative regulation of transport
GO:0070873	2	47	0.00534604	regulation of glycogen metabolic process
GO:0051049	4	2081	0.00541921	regulation of transport
GO:0055114	4	2084	0.00545055	oxidation-reduction process
GO:0045912	2	51	0.00630459	negative regulation of carbohydrate metabolic process
GO:0032881	2	53	0.00681339	regulation of polysaccharide metabolic process
GO:0006111	2	57	0.00789014	regulation of gluconeogenesis
GO:0071417	3	588	0.00874574	cellular response to organonitrogen compound
GO:0010676	2	61	0.00904573	positive regulation of cellular carbohydrate metabolic process
GO:0045913	2	68	0.0112574	positive regulation of carbohydrate metabolic process
GO:1901699	3	645	0.0115357	cellular response to nitrogen compound
GO:0046888	2	79	0.0152197	negative regulation of hormone secretion
GO:0001678	2	86	0.0180507	cellular glucose homeostasis
GO:0032879	4	2827	0.0184706	regulation of localization
GO:0015758	2	88	0.0189038	glucose transport
GO:0043467	2	93	0.0211223	regulation of generation of precursor metabolites and energy
GO:0008645	2	95	0.022044	hexose transport
GO:0015749	2	97	0.0229854	monosaccharide transport
GO:0090277	2	101	0.0249269	positive regulation of peptide hormone secretion
GO:0002793	2	104	0.0264345	positive regulation of peptide secretion
GO:0007267	3	859	0.0271627	cell-cell signaling
GO:0043255	2	111	0.0301238	regulation of carbohydrate biosynthetic process
GO:0023052	3	913	0.0325853	signaling
GO:0044700	3	913	0.0325853	single organism signaling
GO:0051050	3	919	0.0332286	positive regulation of transport
GO:0010827	2	128	0.0400817	regulation of glucose transport
GO:0010906	2	133	0.0432794	regulation of glucose metabolic process
GO:0046887	2	133	0.0432794	positive regulation of hormone secretion
GO:0030001	3	1036	0.0475089	metal ion transport

Table 2: Overrepresented GO terms with the standard enrichment

GO Term	N1	N2	P-value	Description
GO:0008643	2	140	0.0479612	carbohydrate transport
GO:0044723	3	1049	0.0493087	single-organism carbohydrate metabolic process

Table 3: Overrepresented GO terms with the standard enrichment

2 Network-based enrichment

GO Term	N1	N2	P-value	Description
GO:0009743	4	561	0.000146	response to carbohydrate
GO:0009730	2	5	0.00019838	detection of carbohydrate stimulus
GO:0009732	2	5	0.00019838	detection of hexose stimulus
GO:0034287	2	5	0.00019838	detection of monosaccharide stimulus
GO:0051594	2	5	0.00019838	detection of glucose
GO:0046676	3	105	0.00022278	negative regulation of insulin secretion
GO:0032811	2	9	0.000714055	negative regulation of epinephrine secretion
GO:0014060	2	11	0.00109083	regulation of epinephrine secretion
GO:0051156	2	20	0.00376696	glucose 6-phosphate metabolic process
GO:0033604	2	21	0.00416332	negative regulation of catecholamine secretion
GO:0055082	4	1433	0.00625634	cellular chemical homeostasis
GO:0071804	3	351	0.00844502	cellular potassium ion transport
GO:0071805	3	351	0.00844502	potassium ion transmembrane transport
GO:0043269	4	1590	0.00948644	regulation of ion transport
GO:0044320	2	32	0.00982903	cellular response to leptin stimulus
GO:0032355	3	374	0.0102164	response to estradiol
GO:0044321	2	37	0.0131952	response to leptin
GO:0009749	3	416	0.0140573	response to glucose
GO:0006813	3	419	0.0143633	potassium ion transport
GO:0043279	3	424	0.0148833	response to alkaloid
GO:0009746	3	439	0.0165178	response to hexose
GO:0051953	2	42	0.0170553	negative regulation of amine transport
GO:0003013	3	444	0.017088	circulatory system process
GO:0019725	4	1855	0.0175843	cellular homeostasis
GO:0019932	3	457	0.0186315	second-messenger-mediated signaling
GO:0034284	3	458	0.0187539	response to monosaccharide
GO:0046683	3	488	0.0226795	response to organophosphorus
GO:0070509	2	49	0.0232885	calcium ion import
GO:0005978	2	56	0.0304882	glycogen biosynthetic process
GO:0009250	2	56	0.0304882	glucan biosynthetic process
GO:0014074	3	555	0.0333359	response to purine-containing compound
GO:0009607	4	2197	0.0346169	response to biotic stimulus

Table 4: Overrepresented terms with the network-based enrichment. Only terms not detected with the standard method.
