# Supplementary material for: NET-GE: a novel NETwork-based Gene Enrichment for detecting biological processes associated to Mendelian diseases
Source: BMC Genomics. 2015 Jun 18;16(Suppl 8):S6. doi: 10.1186/1471-2164-16-S8-S6 (PMC4480278; doi:10.1186/1471-2164-16-S8-S6)
Supplement: Additional file 3 — Detailed results for the OMIM-derived benchmark set. The archive contains pdf documents listing the enriched terms for each one of the 244 diseases in the OMIM-derived benchmark set. [file 1471-2164-16-S8-S6-S3.tgz › SUPPMAT/OMIM606657.pdf]

# #606657 GLAUCOMA, NORMAL TENSION, SUSCEPTIBILITY TO

| OMIM Gene ID | HGNC | UniProtAC |
|---|---|---|
| 602432 | OPTN | Q96CV9 |
| 605290 | OPA1 | O60313 |

Table 1: OMIM - UniProtAC mapping

Legend

- N1: #input proteins associated to the significant GO term
- N2: #proteins associated to the significant GO term
- P-value: Bonferroni-corrected p-value of Fisher's exact test
- *red*: go terms not related to the input proteins
- *blue*: go terms related to the input proteins (enriched uniquely by network-based method)
- *green*: go terms ancestors of terms enriched with the standard method (enriched uniquely by network-based method)

# 1 Standard enrichment

| GO Term | N1 | N2 | P-value | Description |
|---|---|---|---|---|
| GO:0090161 | 1 | 3 | 0.0338598 | Golgi ribbon formation |
| GO:0001920 | 1 | 4 | 0.0451456 | negative regulation of receptor recycling |

Table 2: Overrepresented GO terms with the standard enrichment

# 2 Network-based enrichment

| GO Term | N1 | N2 | P-value | Description |
|---|---|---|---|---|
| GO:0097191 | 2 | 280 | 0.019075 | extrinsic apoptotic signaling pathway |

Table 3: Overrepresented terms with the network-based enrichment. Only terms not detected with the standard method.
