# Supplementary material for: NET-GE: a novel NETwork-based Gene Enrichment for detecting biological processes associated to Mendelian diseases
Source: BMC Genomics. 2015 Jun 18;16(Suppl 8):S6. doi: 10.1186/1471-2164-16-S8-S6 (PMC4480278; doi:10.1186/1471-2164-16-S8-S6)
Supplement: Additional file 3 — Detailed results for the OMIM-derived benchmark set. The archive contains pdf documents listing the enriched terms for each one of the 244 diseases in the OMIM-derived benchmark set. [file 1471-2164-16-S8-S6-S3.tgz › SUPPMAT/OMIM606690.pdf]

#606690 LYMPHANGIOLEIOMYOMATOSIS; LAM

OMIM Gene ID	HGNC	UniProtAC
191092	TSC2	P49815
605284	TSC1	Q92574

Table 1: OMIM - UniProtAC mapping

Legend

- N1: #input proteins associated to the significant GO term
- N2: #proteins associated to the significant GO term
- P-value: Bonferroni-corrected p-value of Fisher's exact test
- *red*: go terms not related to the input proteins
- *blue*: go terms related to the input proteins (enriched uniquely by network-based method)
- *green*: go terms ancestors of terms enriched with the standard method (enriched uniquely by network-based method)

1 Standard enrichment

GO Term	N1	N2	P-value	Description
GO:0045792	2	20	7.65604e-05	negative regulation of cell size
GO:0032007	2	38	0.000283274	negative regulation of TOR signaling
GO:0046627	2	44	0.000381191	negative regulation of insulin receptor signaling pathway
GO:1900077	2	46	0.000417054	negative regulation of cellular response to insulin stimulus
GO:0008361	2	62	0.000761976	regulation of cell size
GO:0032006	2	67	0.000890923	regulation of TOR signaling
GO:0046626	2	73	0.00105895	regulation of insulin receptor signaling pathway
GO:1900076	2	98	0.00191522	regulation of cellular response to insulin stimulus
GO:0001843	2	120	0.00287706	neural tube closure
GO:0060606	2	122	0.00297418	tube closure
GO:0051291	2	177	0.00627635	protein heterooligomerization
GO:0035148	2	178	0.00634767	tube formation
GO:0007050	2	189	0.00715881	cell cycle arrest
GO:0008286	2	195	0.0076218	insulin receptor signaling pathway
GO:0032535	2	269	0.0145247	regulation of cellular component size
GO:0032869	2	270	0.0146331	cellular response to insulin stimulus
GO:0006913	2	318	0.0203099	nucleocytoplasmic transport
GO:0051169	2	323	0.0209546	nuclear transport
GO:0045786	2	343	0.0236342	negative regulation of cell cycle
GO:0090066	2	362	0.0263291	regulation of anatomical structure size
GO:0071375	2	372	0.0278059	cellular response to peptide hormone stimulus
GO:0032868	2	376	0.0284079	response to insulin
GO:1901653	2	391	0.0307228	cellular response to peptide
GO:0006407	1	3	0.0456232	rRNA export from nucleus

Table 2: Overrepresented GO terms with the standard enrichment

2 Network-based enrichment

No novel enriched terms
