# Supplementary material for: NET-GE: a novel NETwork-based Gene Enrichment for detecting biological processes associated to Mendelian diseases
Source: BMC Genomics. 2015 Jun 18;16(Suppl 8):S6. doi: 10.1186/1471-2164-16-S8-S6 (PMC4480278; doi:10.1186/1471-2164-16-S8-S6)
Supplement: Additional file 3 — Detailed results for the OMIM-derived benchmark set. The archive contains pdf documents listing the enriched terms for each one of the 244 diseases in the OMIM-derived benchmark set. [file 1471-2164-16-S8-S6-S3.tgz › SUPPMAT/OMIM606764.pdf]

#606764 GASTROINTESTINAL STROMAL TUMOR; GIST

OMIM Gene ID	HGNC	UniProtAC
164920	KIT	P10721
173490	PDGFRA	P16234
185470	SDHB	P21912
602413	SDHC	Q99643

Table 1: OMIM - UniProtAC mapping

Legend

- N1: #input proteins associated to the significant GO term
- N2: #proteins associated to the significant GO term
- P-value: Bonferroni-corrected p-value of Fisher's exact test
- *red*: go terms not related to the input proteins
- *blue*: go terms related to the input proteins (enriched uniquely by network-based method)
- *green*: go terms ancestors of terms enriched with the standard method (enriched uniquely by network-based method)

1 Standard enrichment

GO Term	N1	N2	P-value	Description
GO:0009060	2	27	0.00153324	aerobic respiration
GO:0043552	2	29	0.00177337	positive regulation of phosphatidylinositol 3-kinase activity
GO:0090218	2	30	0.00189997	positive regulation of lipid kinase activity
GO:0045333	2	44	0.00412984	cellular respiration
GO:0006099	2	49	0.00513302	tricarboxylic acid cycle
GO:0043551	2	60	0.00772272	regulation of phosphatidylinositol 3-kinase activity
GO:0014068	2	67	0.00964447	positive regulation of phosphatidylinositol 3-kinase signaling
GO:0010863	2	68	0.00993636	positive regulation of phospholipase C activity
GO:0043550	2	70	0.0105332	regulation of lipid kinase activity
GO:1900274	2	70	0.0105332	regulation of phospholipase C activity
GO:0010518	2	93	0.0186436	positive regulation of phospholipase activity
GO:0014066	2	95	0.0194572	regulation of phosphatidylinositol 3-kinase signaling
GO:0010517	2	104	0.0233325	regulation of phospholipase activity
GO:0060193	2	108	0.0251673	positive regulation of lipase activity
GO:0022904	2	131	0.0370587	respiratory electron transport chain
GO:0022602	2	138	0.0411309	ovulation cycle process
GO:0045834	2	139	0.0417299	positive regulation of lipid metabolic process
GO:0022900	2	151	0.0492535	electron transport chain

Table 2: Overrepresented GO terms with the standard enrichment

2 Network-based enrichment

GO Term	N1	N2	P-value	Description
GO:0043473	2	115	0.0377463	pigmentation

Table 3: Overrepresented terms with the network-based enrichment. Only terms not detected with the standard method.
