# Supplementary material for: NET-GE: a novel NETwork-based Gene Enrichment for detecting biological processes associated to Mendelian diseases
Source: BMC Genomics. 2015 Jun 18;16(Suppl 8):S6. doi: 10.1186/1471-2164-16-S8-S6 (PMC4480278; doi:10.1186/1471-2164-16-S8-S6)
Supplement: Additional file 3 — Detailed results for the OMIM-derived benchmark set. The archive contains pdf documents listing the enriched terms for each one of the 244 diseases in the OMIM-derived benchmark set. [file 1471-2164-16-S8-S6-S3.tgz › SUPPMAT/OMIM606864.pdf]

#606864 PARAGANGLIOMA AND GASTRIC STROMAL SARCOMA

OMIM Gene ID	HGNC	UniProtAC
185470	SDHB	P21912
602413	SDHC	Q99643
602690	SDHD	O14521

Table 1: OMIM - UniProtAC mapping

Legend

- N1: #input proteins associated to the significant GO term
- N2: #proteins associated to the significant GO term
- P-value: Bonferroni-corrected p-value of Fisher's exact test
- *red*: go terms not related to the input proteins
- *blue*: go terms related to the input proteins (enriched uniquely by network-based method)
- *green*: go terms ancestors of terms enriched with the standard method (enriched uniquely by network-based method)

1 Standard enrichment

GO Term	N1	N2	P-value	Description
GO:0006099	3	49	4.72921e-08	tricarboxylic acid cycle
GO:0022904	3	131	9.39847e-07	respiratory electron transport chain
GO:0022900	3	151	1.4438e-06	electron transport chain
GO:0009060	2	27	3.39885e-05	aerobic respiration
GO:0006091	3	531	6.36911e-05	generation of precursor metabolites and energy
GO:0045333	2	44	9.1577e-05	cellular respiration
GO:0015980	2	231	0.00256312	energy derivation by oxidation of organic compounds
GO:0055114	3	2084	0.00386653	oxidation-reduction process
GO:0006105	1	17	0.0310654	succinate metabolic process
GO:0044281	3	4403	0.0364925	small molecule metabolic process

Table 2: Overrepresented GO terms with the standard enrichment

2 Network-based enrichment

No novel enriched terms
