# Supplementary material for: NET-GE: a novel NETwork-based Gene Enrichment for detecting biological processes associated to Mendelian diseases
Source: BMC Genomics. 2015 Jun 18;16(Suppl 8):S6. doi: 10.1186/1471-2164-16-S8-S6 (PMC4480278; doi:10.1186/1471-2164-16-S8-S6)
Supplement: Additional file 3 — Detailed results for the OMIM-derived benchmark set. The archive contains pdf documents listing the enriched terms for each one of the 244 diseases in the OMIM-derived benchmark set. [file 1471-2164-16-S8-S6-S3.tgz › SUPPMAT/OMIM607174.pdf]

#607174 MENINGIOMA, FAMILIAL, SUSCEPTIBILITY TO

OMIM Gene ID	HGNC	UniProtAC
156100	MN1	Q10571
190040	PDGFB	P01127
601728	PTEN	P60484
603111	SMARCE1	Q969G3
607035	SUFU	Q9UMX1
607379	NF2	P35240

Table 1: OMIM - UniProtAC mapping

Legend

- N1: #input proteins associated to the significant GO term
- N2: #proteins associated to the significant GO term
- P-value: Bonferroni-corrected p-value of Fisher's exact test
- *red*: go terms not related to the input proteins
- *blue*: go terms related to the input proteins (enriched uniquely by network-based method)
- *green*: go terms ancestors of terms enriched with the standard method (enriched uniquely by network-based method)

1 Standard enrichment

GO Term	N1	N2	P-value	Description
GO:0030336	3	243	0.00442799	negative regulation of cell migration
GO:0010605	5	2452	0.0055748	negative regulation of macromolecule metabolic process
GO:0031324	5	2479	0.00588511	negative regulation of cellular metabolic process
GO:2000146	3	269	0.00600468	negative regulation of cell motility
GO:0051271	3	274	0.00634518	negative regulation of cellular component movement
GO:0009892	5	2679	0.00863638	negative regulation of metabolic process
GO:0040013	3	316	0.00972294	negative regulation of locomotion
GO:2000058	2	38	0.012582	regulation of protein ubiquitination involved in ubiquitin-dependent protein catabolic process
GO:0001953	2	40	0.0139581	negative regulation of cell-matrix adhesion
GO:0045840	2	43	0.0161558	positive regulation of mitosis
GO:0048008	2	54	0.0255825	platelet-derived growth factor receptor signaling pathway
GO:0010638	3	454	0.028679	positive regulation of organelle organization
GO:0010812	2	58	0.0295429	negative regulation of cell-substrate adhesion
GO:0051785	2	64	0.0360153	positive regulation of nuclear division
GO:2000113	4	1622	0.0405096	negative regulation of cellular macromolecule biosynthetic process
GO:0045934	4	1654	0.043744	negative regulation of nucleobase-containing compound metabolic process
GO:0010563	3	529	0.0452078	negative regulation of phosphorus metabolic process
GO:0045936	3	529	0.0452078	negative regulation of phosphate metabolic process
GO:0051172	4	1688	0.047386	negative regulation of nitrogen compound metabolic process
GO:0010558	4	1696	0.0482746	negative regulation of macromolecule biosynthetic process

Table 2: Overrepresented GO terms with the standard enrichment

2 Network-based enrichment

GO Term	N1	N2	P-value	Description
GO:0007173	4	432	0.000632252	epidermal growth factor receptor signaling pathway
GO:0038127	4	452	0.000757454	ERBB signaling pathway
GO:0043588	3	99	0.000780423	skin development
GO:0050730	4	663	0.0034853	regulation of peptidyl-tyrosine phosphorylation
GO:0032269	5	1804	0.00409471	negative regulation of cellular protein metabolic process
GO:0048011	4	692	0.00413202	neurotrophin TRK receptor signaling pathway
GO:0038179	4	720	0.00483749	neurotrophin signaling pathway
GO:0042176	4	816	0.00795134	regulation of protein catabolic process
GO:0051248	5	2189	0.0106731	negative regulation of protein metabolic process
GO:0060346	2	25	0.0124894	bone trabecula formation
GO:0072111	2	25	0.0124894	cell proliferation involved in kidney development
GO:0051897	3	254	0.0132889	positive regulation of protein kinase B signaling
GO:0048015	3	342	0.0323444	phosphatidylinositol-mediated signaling
GO:0048017	3	342	0.0323444	inositol lipid-mediated signaling
GO:0014911	2	45	0.041149	positive regulation of smooth muscle cell migration
GO:0006275	3	373	0.0419033	regulation of DNA replication
GO:0048646	5	2968	0.0479574	anatomical structure formation involved in morphogenesis
GO:0008543	3	394	0.0493374	fibroblast growth factor receptor signaling pathway
GO:0045944	5	2989	0.0496511	positive regulation of transcription from RNA polymerase II promoter

Table 3: Overrepresented terms with the network-based enrichment. Only terms not detected with the standard method.
