# Supplementary material for: NET-GE: a novel NETwork-based Gene Enrichment for detecting biological processes associated to Mendelian diseases
Source: BMC Genomics. 2015 Jun 18;16(Suppl 8):S6. doi: 10.1186/1471-2164-16-S8-S6 (PMC4480278; doi:10.1186/1471-2164-16-S8-S6)
Supplement: Additional file 3 — Detailed results for the OMIM-derived benchmark set. The archive contains pdf documents listing the enriched terms for each one of the 244 diseases in the OMIM-derived benchmark set. [file 1471-2164-16-S8-S6-S3.tgz › SUPPMAT/OMIM607208.pdf]

# #607208 EPILEPTIC ENCEPHALOPATHY, EARLY INFANTILE, 6; EIEE6

| OMIM Gene ID | HGNC | UniProtAC |
|---|---|---|
| 182389 | SCN1A | P35498 |
| 603415 | SCN9A | Q15858 |

Table 1: OMIM - UniProtAC mapping

Legend

- N1: #input proteins associated to the significant GO term
- N2: #proteins associated to the significant GO term
- P-value: Bonferroni-corrected p-value of Fisher's exact test
- *red*: go terms not related to the input proteins
- *blue*: go terms related to the input proteins (enriched uniquely by network-based method)
- *green*: go terms ancestors of terms enriched with the standard method (enriched uniquely by network-based method)

# 1 Standard enrichment

| GO Term | N1 | N2 | P-value | Description |
|---|---|---|---|---|
| GO:0019228 | 2 | 34 | 4.56835e-05 | neuronal action potential |
| GO:0086010 | 2 | 40 | 6.3517e-05 | membrane depolarization during action potential |
| GO:0001508 | 2 | 83 | 0.000277114 | action potential |
| GO:0035725 | 2 | 140 | 0.000792338 | sodium ion transmembrane transport |
| GO:0051899 | 2 | 148 | 0.000885822 | membrane depolarization |
| GO:0006814 | 2 | 233 | 0.00220095 | sodium ion transport |
| GO:0042391 | 2 | 353 | 0.00505922 | regulation of membrane potential |
| GO:0034765 | 2 | 424 | 0.00730255 | regulation of ion transmembrane transport |
| GO:0034762 | 2 | 437 | 0.00775773 | regulation of transmembrane transport |
| GO:0044708 | 2 | 596 | 0.0144388 | single-organism behavior |
| GO:0043269 | 2 | 721 | 0.0211366 | regulation of ion transport |
| GO:0015672 | 2 | 743 | 0.022447 | monovalent inorganic cation transport |
| GO:0007610 | 2 | 806 | 0.0264178 | behavior |
| GO:0098662 | 2 | 848 | 0.0292446 | inorganic cation transmembrane transport |
| GO:0019227 | 1 | 11 | 0.0338031 | neuronal action potential propagation |
| GO:0050884 | 1 | 12 | 0.0368756 | neuromuscular process controlling posture |
| GO:0098660 | 2 | 995 | 0.0402695 | inorganic ion transmembrane transport |
| GO:0030001 | 2 | 1036 | 0.0436583 | metal ion transport |
| GO:0098655 | 2 | 1072 | 0.0467467 | cation transmembrane transport |

Table 2: Overrepresented GO terms with the standard enrichment

# 2 Network-based enrichment

| GO Term | N1 | N2 | P-value | Description |
|---|---|---|---|---|
| GO:0086046 | 1 | 7 | 0.02185 | membrane depolarization during SA node cell action potential |
| GO:0086014 | 1 | 16 | 0.0499362 | atrial cardiac muscle cell action potential |

Table 3: Overrepresented terms with the network-based enrichment. Only terms not detected with the standard method.
