# Supplementary material for: NET-GE: a novel NETwork-based Gene Enrichment for detecting biological processes associated to Mendelian diseases
Source: BMC Genomics. 2015 Jun 18;16(Suppl 8):S6. doi: 10.1186/1471-2164-16-S8-S6 (PMC4480278; doi:10.1186/1471-2164-16-S8-S6)
Supplement: Additional file 3 — Detailed results for the OMIM-derived benchmark set. The archive contains pdf documents listing the enriched terms for each one of the 244 diseases in the OMIM-derived benchmark set. [file 1471-2164-16-S8-S6-S3.tgz › SUPPMAT/OMIM607507.pdf]

#607507 PSORIATIC ARTHRITIS, SUSCEPTIBILITY TO

OMIM Gene ID	HGNC	UniProtAC
153440	LTA	P01374
605956	NOD2	Q9HC29

Table 1: OMIM - UniProtAC mapping

Legend

- N1: #input proteins associated to the significant GO term
- N2: #proteins associated to the significant GO term
- P-value: Bonferroni-corrected p-value of Fisher's exact test
- *red*: go terms not related to the input proteins
- *blue*: go terms related to the input proteins (enriched uniquely by network-based method)
- *green*: go terms ancestors of terms enriched with the standard method (enriched uniquely by network-based method)

1 Standard enrichment

GO Term	N1	N2	P-value	Description
GO:0002925	2	8	1.93809e-05	positive regulation of humoral immune response mediated by circulating immunoglobulin
GO:0002923	2	14	6.29877e-05	regulation of humoral immune response mediated by circulating immunoglobulin
GO:0002714	2	16	8.30611e-05	positive regulation of B cell mediated immunity
GO:0002891	2	16	8.30611e-05	positive regulation of immunoglobulin mediated immune response
GO:0002922	2	16	8.30611e-05	positive regulation of humoral immune response
GO:0002861	2	27	0.000242953	regulation of inflammatory response to antigenic stimulus
GO:0044126	2	32	0.000343319	regulation of growth of symbiont in host
GO:0044130	2	32	0.000343319	negative regulation of growth of symbiont in host
GO:0044144	2	32	0.000343319	modulation of growth of symbiont involved in interaction with host
GO:0044146	2	32	0.000343319	negative regulation of growth of symbiont involved in interaction with host
GO:0002889	2	57	0.00110471	regulation of immunoglobulin mediated immune response
GO:0002712	2	58	0.00114416	regulation of B cell mediated immunity
GO:0002920	2	61	0.00126668	regulation of humoral immune response
GO:0050830	2	78	0.0020786	defense response to Gram-positive bacterium
GO:0032649	2	105	0.00377927	regulation of interferon-gamma production
GO:0043901	2	154	0.00815452	negative regulation of multi-organism process
GO:0002824	2	157	0.00847635	positive regulation of adaptive immune response based on somatic recombination of immune receptors built fr[illegible]
GO:0002821	2	160	0.00880444	positive regulation of adaptive immune response
GO:0002708	2	161	0.00891521	positive regulation of lymphocyte mediated immunity
GO:0002705	2	163	0.00913879	positive regulation of leukocyte mediated immunity
GO:0002706	2	222	0.0169797	regulation of lymphocyte mediated immunity
GO:0043903	2	223	0.0171334	regulation of symbiosis, encompassing mutualism through parasitism
GO:0042742	2	233	0.0187081	defense response to bacterium
GO:0002822	2	235	0.0190313	regulation of adaptive immune response based on somatic recombination of immune receptors built from imm[illegible]
GO:0002819	2	246	0.0208587	regulation of adaptive immune response
GO:0002703	2	259	0.0231262	regulation of leukocyte mediated immunity
GO:0009617	2	261	0.0234855	response to bacterium
GO:0002699	2	269	0.0249501	positive regulation of immune effector process
GO:0032498	1	1	0.0261241	detection of muramyl dipeptide
GO:0032499	1	1	0.0261241	detection of peptidoglycan
GO:0045926	2	303	0.031669	negative regulation of growth
GO:0007584	2	307	0.0325121	response to nutrient
GO:0031349	2	321	0.0355501	positive regulation of defense response
GO:0050727	2	372	0.0477642	regulation of inflammatory response
GO:0001819	2	374	0.0482799	positive regulation of cytokine production

Table 2: Overrepresented GO terms with the standard enrichment

2 Network-based enrichment

GO Term	N1	N2	P-value	Description
GO:1990266	2	123	0.00805834	neutrophil migration
GO:0097530	2	154	0.012653	granulocyte migration
GO:0097529	2	222	0.0263466	myeloid leukocyte migration
GO:0050729	2	261	0.0364413	positive regulation of inflammatory response

Table 3: Overrepresented terms with the network-based enrichment. Only terms not detected with the standard method.
