# Supplementary material for: NET-GE: a novel NETwork-based Gene Enrichment for detecting biological processes associated to Mendelian diseases
Source: BMC Genomics. 2015 Jun 18;16(Suppl 8):S6. doi: 10.1186/1471-2164-16-S8-S6 (PMC4480278; doi:10.1186/1471-2164-16-S8-S6)
Supplement: Additional file 3 — Detailed results for the OMIM-derived benchmark set. The archive contains pdf documents listing the enriched terms for each one of the 244 diseases in the OMIM-derived benchmark set. [file 1471-2164-16-S8-S6-S3.tgz › SUPPMAT/OMIM607785.pdf]

#607785 JUVENILE MYELOMONOCYTIC LEUKEMIA; JMML

**OMIM Gene ID**	**HGNC**	**UniProtAC**
176876	PTPN11	Q06124
605370	ARHGAP26	Q9UNA1
613113	NF1	P21359

Table 1: OMIM - UniProtAC mapping

Legend

- N1: #input proteins associated to the significant GO term
- N2: #proteins associated to the significant GO term
- P-value: Bonferroni-corrected p-value of Fisher's exact test
- *red*: go terms not related to the input proteins
- *blue*: go terms related to the input proteins (enriched uniquely by network-based method)
- *green*: go terms ancestors of terms enriched with the standard method (enriched uniquely by network-based method)

1 Standard enrichment

GO Term	N1	N2	P-value	Description
GO:0048731	3	879	0.00664726	system development
GO:0007162	2	145	0.0231593	negative regulation of cell adhesion
GO:0048015	2	182	0.0365141	phosphatidylinositol-mediated signaling
GO:0048017	2	182	0.0365141	inositol lipid-mediated signaling
GO:0051463	1	1	0.041968	negative regulation of cortisol secretion
GO:0060125	1	1	0.041968	negative regulation of growth hormone secretion

Table 2: Overrepresented GO terms with the standard enrichment

2 Network-based enrichment

GO Term	N1	N2	P-value	Description
GO:0045671	2	62	0.0113543	negative regulation of osteoclast differentiation
GO:0051056	3	769	0.0136241	regulation of small GTPase mediated signal transduction
GO:0045762	2	77	0.0175635	positive regulation of adenylate cyclase activity
GO:2000178	2	83	0.0204243	negative regulation of neural precursor cell proliferation
GO:0030036	3	914	0.0228895	actin cytoskeleton organization
GO:0030325	2	95	0.0267917	adrenal gland development
GO:0045124	2	99	0.0291056	regulation of bone resorption
GO:0030029	3	1031	0.0328652	actin filament-based process
GO:0032320	3	1071	0.0368448	positive regulation of Ras GTPase activity
GO:0046580	2	115	0.0393169	negative regulation of Ras protein signal transduction
GO:0031281	2	119	0.0421085	positive regulation of cyclase activity
GO:0051058	2	120	0.0428215	negative regulation of small GTPase mediated signal transduction
GO:0043535	2	125	0.0464752	regulation of blood vessel endothelial cell migration
GO:0042326	3	1164	0.0473112	negative regulation of phosphorylation
GO:0045761	2	128	0.048739	regulation of adenylate cyclase activity

Table 3: Overrepresented terms with the network-based enrichment. Only terms not detected with the standard method.
