# Supplementary material for: NET-GE: a novel NETwork-based Gene Enrichment for detecting biological processes associated to Mendelian diseases
Source: BMC Genomics. 2015 Jun 18;16(Suppl 8):S6. doi: 10.1186/1471-2164-16-S8-S6 (PMC4480278; doi:10.1186/1471-2164-16-S8-S6)
Supplement: Additional file 3 — Detailed results for the OMIM-derived benchmark set. The archive contains pdf documents listing the enriched terms for each one of the 244 diseases in the OMIM-derived benchmark set. [file 1471-2164-16-S8-S6-S3.tgz › SUPPMAT/OMIM607948.pdf]

# #607948 MYCOBACTERIUM TUBERCULOSIS, SUSCEPTIBILITY TO

| **OMIM Gene ID** | **HGNC** | **UniProtAC** |
|---|---|---|
| 107470 | IFNGR1 | P15260 |
| 147570 | IFNG | P01579 |
| 155540 | MC3R | P41968 |
| 158105 | CCL2 | P13500 |
| 600266 | SLC11A1 | P49279 |
| 602441 | CISH | Q9NSE2 |
| 603028 | TLR2 | O60603 |
| 604457 | SP110 | Q9HB58 |
| 604672 | CD209 | Q9NNX6 |
| 606252 | TIRAP | P58753 |
| 608212 | IRGM | A1A4Y4 |

Table 1: OMIM - UniProtAC mapping

Legend

- N1: #input proteins associated to the significant GO term
- N2: #proteins associated to the significant GO term
- P-value: Bonferroni-corrected p-value of Fisher's exact test
- *red*: go terms not related to the input proteins
- *blue*: go terms related to the input proteins (enriched uniquely by network-based method)
- *green*: go terms ancestors of terms enriched with the standard method (enriched uniquely by network-based method)

# 1 Standard enrichment

| GO Term | N1 | N2 | P-value | Description |
|---|---|---|---|---|
| GO:0051704 | 8 | 1558 | 1.26205e-06 | multi-organism process |
| GO:0009617 | 5 | 261 | 7.01381e-06 | response to bacterium |
| GO:0051707 | 6 | 607 | 7.51645e-06 | response to other organism |
| GO:0006955 | 7 | 1414 | 3.04573e-05 | immune response |
| GO:0032496 | 5 | 389 | 5.13591e-05 | response to lipopolysaccharide |
| GO:0006952 | 7 | 1580 | 6.52997e-05 | defense response |
| GO:0002237 | 5 | 414 | 7.00005e-05 | response to molecule of bacterial origin |
| GO:0043207 | 6 | 921 | 8.92031e-05 | response to external biotic stimulus |
| GO:0044126 | 3 | 32 | 9.38251e-05 | regulation of growth of symbiont in host |
| GO:0044130 | 3 | 32 | 9.38251e-05 | negative regulation of growth of symbiont in host |
| GO:0044144 | 3 | 32 | 9.38251e-05 | modulation of growth of symbiont involved in interaction with host |
| GO:0044146 | 3 | 32 | 9.38251e-05 | negative regulation of growth of symbiont involved in interaction with host |
| GO:0009607 | 6 | 964 | 0.000116799 | response to biotic stimulus |
| GO:0002682 | 7 | 1758 | 0.000135702 | regulation of immune system process |
| GO:0006954 | 5 | 483 | 0.000150432 | inflammatory response |
| GO:0032735 | 3 | 43 | 0.000233039 | positive regulation of interleukin-12 production |
| GO:0071219 | 4 | 198 | 0.000243118 | cellular response to molecule of bacterial origin |
| GO:0034341 | 4 | 201 | 0.000258193 | response to interferon-gamma |
| GO:0071216 | 4 | 215 | 0.000337958 | cellular response to biotic stimulus |
| GO:0042742 | 4 | 233 | 0.000465924 | defense response to bacterium |
| GO:0032760 | 3 | 56 | 0.000522363 | positive regulation of tumor necrosis factor production |
| GO:0001817 | 5 | 702 | 0.0009536 | regulation of cytokine production |
| GO:0032642 | 3 | 71 | 0.00107448 | regulation of chemokine production |
| GO:0002376 | 7 | 2446 | 0.00128546 | immune system process |
| GO:0032655 | 3 | 79 | 0.00148474 | regulation of interleukin-12 production |
| GO:0045088 | 4 | 320 | 0.00164789 | regulation of innate immune response |
| GO:0044419 | 5 | 788 | 0.00168256 | interspecies interaction between organisms |
| GO:0002687 | 3 | 84 | 0.00178758 | positive regulation of leukocyte migration |
| GO:0042108 | 3 | 85 | 0.00185268 | positive regulation of cytokine biosynthetic process |
| GO:0050896 | 11 | 11721 | 0.00266492 | response to stimulus |
| GO:0001819 | 4 | 374 | 0.00305842 | positive regulation of cytokine production |
| GO:0051701 | 3 | 104 | 0.00340551 | interaction with host |
| GO:0032680 | 3 | 115 | 0.00460926 | regulation of tumor necrosis factor production |
| GO:0002685 | 3 | 119 | 0.00510845 | regulation of leukocyte migration |
| GO:0032700 | 2 | 12 | 0.00525127 | negative regulation of interleukin-17 production |
| GO:0071220 | 2 | 12 | 0.00525127 | cellular response to bacterial lipoprotein |
| GO:0071221 | 2 | 12 | 0.00525127 | cellular response to bacterial lipopeptide |
| GO:0098542 | 4 | 431 | 0.00535983 | defense response to other organism |
| GO:0032675 | 3 | 126 | 0.00606586 | regulation of interleukin-6 production |
| GO:0034123 | 2 | 14 | 0.00723809 | positive regulation of toll-like receptor signaling pathway |
| GO:0051770 | 2 | 14 | 0.00723809 | positive regulation of nitric-oxide synthase biosynthetic process |
| GO:0070391 | 2 | 14 | 0.00723809 | response to lipoteichoic acid |
| GO:0071223 | 2 | 14 | 0.00723809 | cellular response to lipoteichoic acid |
| GO:0009605 | 6 | 1995 | 0.00819743 | response to external stimulus |
| GO:0002684 | 5 | 1093 | 0.00831989 | positive regulation of immune system process |
| GO:0042035 | 3 | 143 | 0.00886867 | regulation of cytokine biosynthetic process |
| GO:0045410 | 2 | 16 | 0.0095417 | positive regulation of interleukin-6 biosynthetic process |
| GO:0070339 | 2 | 16 | 0.0095417 | response to bacterial lipopeptide |
| GO:0033993 | 5 | 1142 | 0.0102953 | response to lipid |
| GO:0051249 | 4 | 513 | 0.0106502 | regulation of lymphocyte activation |
| GO:0043901 | 3 | 154 | 0.0110741 | negative regulation of multi-organism process |
| GO:0050776 | 5 | 1167 | 0.011436 | regulation of immune response |
| GO:0032493 | 2 | 18 | 0.0121618 | response to bacterial lipoprotein |
| GO:0051769 | 2 | 18 | 0.0121618 | regulation of nitric-oxide synthase biosynthetic process |
| GO:0009891 | 6 | 2208 | 0.014701 | positive regulation of biosynthetic process |
| GO:0002263 | 3 | 170 | 0.0148865 | cell activation involved in immune response |
| GO:0002366 | 3 | 170 | 0.0148865 | leukocyte activation involved in immune response |
| GO:0071346 | 3 | 173 | 0.015686 | cellular response to interferon-gamma |
| GO:0010562 | 5 | 1255 | 0.0162637 | positive regulation of phosphorus metabolic process |
| GO:0045937 | 5 | 1255 | 0.0162637 | positive regulation of phosphate metabolic process |

Table 2: Overrepresented GO terms with the standard enrichment

| GO Term | N1 | N2 | P-value | Description |
|---|---|---|---|---|
| GO:0032757 | 2 | 21 | 0.0166847 | positive regulation of interleukin-8 production |
| GO:0002694 | 4 | 584 | 0.0177234 | regulation of leukocyte activation |
| GO:0032660 | 2 | 23 | 0.0200947 | regulation of interleukin-17 production |
| GO:0007165 | 9 | 7592 | 0.0204644 | signal transduction |
| GO:0071222 | 3 | 191 | 0.0210836 | cellular response to lipopolysaccharide |
| GO:0050865 | 4 | 624 | 0.0229782 | regulation of cell activation |
| GO:0060334 | 2 | 25 | 0.0238201 | regulation of interferon-gamma-mediated signaling pathway |
| GO:0042832 | 2 | 26 | 0.025801 | defense response to protozoan |
| GO:0060330 | 2 | 26 | 0.025801 | regulation of response to interferon-gamma |
| GO:0065008 | 7 | 3888 | 0.0286354 | regulation of biological quality |
| GO:0045408 | 2 | 28 | 0.0299991 | regulation of interleukin-6 biosynthetic process |
| GO:0001818 | 3 | 215 | 0.0300099 | negative regulation of cytokine production |
| GO:0019079 | 2 | 29 | 0.032216 | viral genome replication |
| GO:0043903 | 3 | 223 | 0.0334603 | regulation of symbiosis, encompassing mutualism through parasitism |
| GO:0001562 | 2 | 30 | 0.0345117 | response to protozoan |
| GO:0043489 | 2 | 31 | 0.0368861 | RNA stabilization |
| GO:0048255 | 2 | 31 | 0.0368861 | mRNA stabilization |
| GO:0031347 | 4 | 706 | 0.0372344 | regulation of defense response |
| GO:0050670 | 3 | 238 | 0.0406144 | regulation of lymphocyte proliferation |
| GO:0051247 | 5 | 1526 | 0.0417231 | positive regulation of protein metabolic process |
| GO:0071622 | 2 | 33 | 0.0418701 | regulation of granulocyte chemotaxis |
| GO:0032944 | 3 | 241 | 0.0421561 | regulation of mononuclear cell proliferation |
| GO:0006950 | 7 | 4134 | 0.0429229 | response to stress |
| GO:0032103 | 3 | 245 | 0.044271 | positive regulation of response to external stimulus |
| GO:0070663 | 3 | 248 | 0.0459022 | regulation of leukocyte proliferation |
| GO:0000060 | 2 | 36 | 0.0499349 | protein import into nucleus, translocation |

Table 3: Overrepresented GO terms with the standard enrichment

## 2 Network-based enrichment

| GO Term | N1 | N2 | P-value | Description |
|---|---|---|---|---|
| GO:0001816 | 4 | 232 | 0.00105677 | cytokine production |
| GO:0034097 | 7 | 2017 | 0.00113914 | response to cytokine |
| GO:0002440 | 3 | 61 | 0.00138473 | production of molecular mediator of immune response |
| GO:0002274 | 4 | 290 | 0.00256822 | myeloid leukocyte activation |
| GO:0042116 | 3 | 78 | 0.0029181 | macrophage activation |
| GO:0002252 | 6 | 1396 | 0.00299662 | immune effector process |
| GO:0042592 | 8 | 3571 | 0.00307329 | homeostatic process |
| GO:0051092 | 4 | 312 | 0.00343308 | positive regulation of NF-kappaB transcription factor activity |
| GO:0050778 | 6 | 1484 | 0.00427672 | positive regulation of immune response |
| GO:0006935 | 5 | 841 | 0.00598896 | chemotaxis |
| GO:0042330 | 5 | 841 | 0.00598896 | taxis |
| GO:0007259 | 3 | 100 | 0.00617787 | JAK-STAT cascade |
| GO:0051251 | 5 | 874 | 0.00722659 | positive regulation of lymphocyte activation |
| GO:0043123 | 4 | 400 | 0.00917674 | positive regulation of I-kappaB kinase/NF-kappaB signaling |
| GO:0045428 | 3 | 117 | 0.00990804 | regulation of nitric oxide biosynthetic process |
| GO:0002696 | 5 | 941 | 0.0103568 | positive regulation of leukocyte activation |
| GO:0010759 | 2 | 13 | 0.0112028 | positive regulation of macrophage chemotaxis |
| GO:0050766 | 3 | 122 | 0.0112352 | positive regulation of phagocytosis |
| GO:0050867 | 5 | 996 | 0.0136511 | positive regulation of cell activation |
| GO:0045087 | 6 | 1935 | 0.019832 | innate immune response |
| GO:0045080 | 2 | 18 | 0.021955 | positive regulation of chemokine biosynthetic process |
| GO:0030888 | 3 | 153 | 0.0221481 | regulation of B cell proliferation |
| GO:0019221 | 5 | 1123 | 0.0244228 | cytokine-mediated signaling pathway |
| GO:0002460 | 3 | 160 | 0.0253193 | adaptive immune response based on somatic recombination of immune receptors built from immunoglobulin s |
| GO:0002690 | 3 | 164 | 0.027259 | positive regulation of leukocyte chemotaxis |
| GO:0050830 | 3 | 173 | 0.0319764 | defense response to Gram-positive bacterium |
| GO:0050764 | 3 | 179 | 0.0354025 | regulation of phagocytosis |
| GO:0050871 | 3 | 179 | 0.0354025 | positive regulation of B cell activation |
| GO:0010758 | 2 | 23 | 0.036272 | regulation of macrophage chemotaxis |
| GO:0006897 | 5 | 1228 | 0.0376011 | endocytosis |
| GO:0055065 | 5 | 1233 | 0.0383446 | metal ion homeostasis |
| GO:0071675 | 2 | 24 | 0.0395622 | regulation of mononuclear cell migration |

Table 4: Overrepresented terms with the network-based enrichment. Only terms not detected with the standard method.
