# Supplementary material for: NET-GE: a novel NETwork-based Gene Enrichment for detecting biological processes associated to Mendelian diseases
Source: BMC Genomics. 2015 Jun 18;16(Suppl 8):S6. doi: 10.1186/1471-2164-16-S8-S6 (PMC4480278; doi:10.1186/1471-2164-16-S8-S6)
Supplement: Additional file 3 — Detailed results for the OMIM-derived benchmark set. The archive contains pdf documents listing the enriched terms for each one of the 244 diseases in the OMIM-derived benchmark set. [file 1471-2164-16-S8-S6-S3.tgz › SUPPMAT/OMIM608089.pdf]

#608089 ENDOMETRIAL CANCER

**OMIM Gene ID**	**HGNC**	**UniProtAC**
192090	CDH1	P12830
600678	MSH6	P52701
601728	PTEN	P60484
604395	MLH3	Q9UHC1

Table 1: OMIM - UniProtAC mapping

Legend

- N1: #input proteins associated to the significant GO term
- N2: #proteins associated to the significant GO term
- P-value: Bonferroni-corrected p-value of Fisher's exact test
- *red*: go terms not related to the input proteins
- *blue*: go terms related to the input proteins (enriched uniquely by network-based method)
- *green*: go terms ancestors of terms enriched with the standard method (enriched uniquely by network-based method)

1 Standard enrichment

GO Term	N1	N2	P-value	Description
GO:0007131	2	40	0.00486239	reciprocal meiotic recombination
GO:0035825	2	40	0.00486239	reciprocal DNA recombination
GO:0006298	2	66	0.0133593	mismatch repair
GO:0007416	2	71	0.0154741	synapse assembly

Table 2: Overrepresented GO terms with the standard enrichment

2 Network-based enrichment

GO Term	N1	N2	P-value	Description
GO:0007144	2	14	0.00172889	female meiosis I
GO:1903046	3	288	0.00446729	meiotic cell cycle process
GO:2000105	2	26	0.00617163	positive regulation of DNA-dependent DNA replication
GO:0008104	4	1615	0.00967417	protein localization
GO:0033036	4	1642	0.0103381	macromolecule localization
GO:0007130	2	39	0.014064	synaptonemal complex assembly
GO:0060395	2	39	0.014064	SMAD protein signal transduction
GO:0045197	2	41	0.0155621	establishment or maintenance of epithelial cell apical/basal polarity
GO:0070193	2	41	0.0155621	synaptonemal complex organization
GO:0009203	4	1920	0.0193368	ribonucleoside triphosphate catabolic process
GO:0009207	4	1920	0.0193368	purine ribonucleoside triphosphate catabolic process
GO:0009146	4	1925	0.0195391	purine nucleoside triphosphate catabolic process
GO:0006974	4	1933	0.0198663	cellular response to DNA damage stimulus
GO:0009143	4	1934	0.0199074	nucleoside triphosphate catabolic process
GO:0006152	4	1974	0.0216076	purine nucleoside catabolic process
GO:0046130	4	1974	0.0216076	purine ribonucleoside catabolic process
GO:0042454	4	2000	0.0227696	ribonucleoside catabolic process
GO:0009154	4	2010	0.0232286	purine ribonucleotide catabolic process
GO:0009261	4	2011	0.023275	ribonucleotide catabolic process
GO:0006195	4	2041	0.0246964	purine nucleotide catabolic process
GO:0009164	4	2045	0.0248906	nucleoside catabolic process
GO:1901658	4	2055	0.0253815	glycosyl compound catabolic process
GO:0072523	4	2067	0.0259799	purine-containing compound catabolic process
GO:0007548	2	53	0.0261394	sex differentiation
GO:0009166	4	2116	0.0285344	nucleotide catabolic process
GO:1901292	4	2130	0.0292976	nucleoside phosphate catabolic process
GO:0010894	2	57	0.0302698	negative regulation of steroid biosynthetic process
GO:0009205	4	2149	0.0303578	purine ribonucleoside triphosphate metabolic process
GO:0009144	4	2159	0.0309273	purine nucleoside triphosphate metabolic process
GO:0009199	4	2170	0.0315628	ribonucleoside triphosphate metabolic process
GO:0032967	2	61	0.0347024	positive regulation of collagen biosynthetic process
GO:0009141	4	2225	0.0348887	nucleoside triphosphate metabolic process
GO:0046434	4	2260	0.0371379	organophosphate catabolic process
GO:0010714	2	64	0.0382249	positive regulation of collagen metabolic process
GO:0035567	2	65	0.0394367	non-canonical Wnt signaling pathway
GO:0035088	2	68	0.0431856	establishment or maintenance of apical/basal cell polarity
GO:0061245	2	68	0.0431856	establishment or maintenance of bipolar cell polarity
GO:0046128	4	2404	0.0475544	purine ribonucleoside metabolic process
GO:0042278	4	2412	0.048191	purine nucleoside metabolic process
GO:0051898	2	73	0.0498108	negative regulation of protein kinase B signaling

Table 3: Overrepresented terms with the network-based enrichment. Only terms not detected with the standard method.
