# Supplementary material for: NET-GE: a novel NETwork-based Gene Enrichment for detecting biological processes associated to Mendelian diseases
Source: BMC Genomics. 2015 Jun 18;16(Suppl 8):S6. doi: 10.1186/1471-2164-16-S8-S6 (PMC4480278; doi:10.1186/1471-2164-16-S8-S6)
Supplement: Additional file 3 — Detailed results for the OMIM-derived benchmark set. The archive contains pdf documents listing the enriched terms for each one of the 244 diseases in the OMIM-derived benchmark set. [file 1471-2164-16-S8-S6-S3.tgz › SUPPMAT/OMIM608133.pdf]

#608133 RETINITIS PIGMENTOSA 7; RP7

**OMIM Gene ID**	**HGNC**	**UniProtAC**
179605	PRPH2	P23942
180721	ROM1	Q03395

Table 1: OMIM - UniProtAC mapping

Legend

- N1: #input proteins associated to the significant GO term
- N2: #proteins associated to the significant GO term
- P-value: Bonferroni-corrected p-value of Fisher's exact test
- *red*: go terms not related to the input proteins
- *blue*: go terms related to the input proteins (enriched uniquely by network-based method)
- *green*: go terms ancestors of terms enriched with the standard method (enriched uniquely by network-based method)

1 Standard enrichment

GO Term	N1	N2	P-value	Description
GO:0007601	2	214	0.00102396	visual perception
GO:0050953	2	218	0.00106268	sensory perception of light stimulus
GO:0007600	2	586	0.0077009	sensory perception
GO:0060219	1	6	0.0101734	camera-type eye photoreceptor cell differentiation
GO:0001754	1	11	0.01865	eye photoreceptor cell differentiation
GO:0046530	1	11	0.01865	photoreceptor cell differentiation
GO:0061298	1	12	0.0203452	retina vasculature development in camera-type eye
GO:0050877	2	1063	0.0253598	neurological system process
GO:0007155	2	1407	0.0444394	cell adhesion
GO:0022610	2	1410	0.0446291	biological adhesion

Table 2: Overrepresented GO terms with the standard enrichment

2 Network-based enrichment

No novel enriched terms
