# Supplementary material for: NET-GE: a novel NETwork-based Gene Enrichment for detecting biological processes associated to Mendelian diseases
Source: BMC Genomics. 2015 Jun 18;16(Suppl 8):S6. doi: 10.1186/1471-2164-16-S8-S6 (PMC4480278; doi:10.1186/1471-2164-16-S8-S6)
Supplement: Additional file 3 — Detailed results for the OMIM-derived benchmark set. The archive contains pdf documents listing the enriched terms for each one of the 244 diseases in the OMIM-derived benchmark set. [file 1471-2164-16-S8-S6-S3.tgz › SUPPMAT/OMIM608161.pdf]

#608161 MACULAR DYSTROPHY, VITELLIFORM, ADULT-ONSET

OMIM Gene ID	HGNC	UniProtAC
179605	PRPH2	P23942
607854	BEST1	O76090

Table 1: OMIM - UniProtAC mapping

Legend

- N1: #input proteins associated to the significant GO term
- N2: #proteins associated to the significant GO term
- P-value: Bonferroni-corrected p-value of Fisher's exact test
- *red*: go terms not related to the input proteins
- *blue*: go terms related to the input proteins (enriched uniquely by network-based method)
- *green*: go terms ancestors of terms enriched with the standard method (enriched uniquely by network-based method)

1 Standard enrichment

GO Term	N1	N2	P-value	Description
GO:0007601	2	214	0.00169593	visual perception
GO:0050953	2	218	0.00176007	sensory perception of light stimulus
GO:0007600	2	586	0.0127546	sensory perception
GO:0030321	1	9	0.0252735	transepithelial chloride transport
GO:0050877	2	1063	0.0420022	neurological system process
GO:0070633	1	16	0.0449266	transepithelial transport

Table 2: Overrepresented GO terms with the standard enrichment

2 Network-based enrichment

No novel enriched terms
