# Supplementary material for: NET-GE: a novel NETwork-based Gene Enrichment for detecting biological processes associated to Mendelian diseases
Source: BMC Genomics. 2015 Jun 18;16(Suppl 8):S6. doi: 10.1186/1471-2164-16-S8-S6 (PMC4480278; doi:10.1186/1471-2164-16-S8-S6)
Supplement: Additional file 3 — Detailed results for the OMIM-derived benchmark set. The archive contains pdf documents listing the enriched terms for each one of the 244 diseases in the OMIM-derived benchmark set. [file 1471-2164-16-S8-S6-S3.tgz › SUPPMAT/OMIM608415.pdf]

#608415 PROLONGED ELECTRORETINAL RESPONSE SUPPRESSION; PERRS

OMIM Gene ID	HGNC	UniProtAC
604067	RGS9	O75916
607814	RGS9BP	Q6ZS82

Table 1: OMIM - UniProtAC mapping

Legend

- N1: #input proteins associated to the significant GO term
- N2: #proteins associated to the significant GO term
- P-value: Bonferroni-corrected p-value of Fisher's exact test
- *red*: go terms not related to the input proteins
- *blue*: go terms related to the input proteins (enriched uniquely by network-based method)
- *green*: go terms ancestors of terms enriched with the standard method (enriched uniquely by network-based method)

1 Standard enrichment

GO Term	N1	N2	P-value	Description
GO:0022400	2	30	5.4356e-05	regulation of rhodopsin mediated signaling pathway
GO:0016056	2	34	7.01006e-05	rhodopsin mediated signaling pathway
GO:0007603	2	89	0.000489329	phototransduction, visible light
GO:0009584	2	106	0.000695383	detection of visible light
GO:0007602	2	112	0.00077673	phototransduction
GO:0071482	2	112	0.00077673	cellular response to light stimulus
GO:0009583	2	129	0.00103164	detection of light stimulus
GO:0071478	2	172	0.00183761	cellular response to radiation
GO:0008277	2	209	0.00271606	regulation of G-protein coupled receptor protein signaling pathway
GO:0009581	2	221	0.00303769	detection of external stimulus
GO:0009582	2	224	0.00312091	detection of abiotic stimulus
GO:0071214	2	302	0.00567939	cellular response to abiotic stimulus
GO:0009416	2	424	0.0112056	response to light stimulus
GO:0009314	2	591	0.0217855	response to radiation
GO:0051606	2	761	0.0361349	detection of stimulus
GO:0032101	2	825	0.0424727	regulation of response to external stimulus

Table 2: Overrepresented GO terms with the standard enrichment

2 Network-based enrichment

No novel enriched terms
