# Supplementary material for: NET-GE: a novel NETwork-based Gene Enrichment for detecting biological processes associated to Mendelian diseases
Source: BMC Genomics. 2015 Jun 18;16(Suppl 8):S6. doi: 10.1186/1471-2164-16-S8-S6 (PMC4480278; doi:10.1186/1471-2164-16-S8-S6)
Supplement: Additional file 3 — Detailed results for the OMIM-derived benchmark set. The archive contains pdf documents listing the enriched terms for each one of the 244 diseases in the OMIM-derived benchmark set. [file 1471-2164-16-S8-S6-S3.tgz › SUPPMAT/OMIM608446.pdf]

#608446 MYOCARDIAL INFARCTION, SUSCEPTIBILITY TO

OMIM Gene ID	HGNC	UniProtAC
133430	ESR1	P03372
134570	F13A1	P00488
150571	LGALS2	P05162
153440	LTA	P01374
173470	ITGB3	P05106
601176	GCLM	P48507
602600	LRP8	Q14114
602601	OLR1	P78380
602855	PSMA6	P60900
603594	TNFSF4	P23510
606857	GCLC	P48506
613878	F7	P08709

Table 1: OMIM - UniProtAC mapping

Legend

- N1: #input proteins associated to the significant GO term
- N2: #proteins associated to the significant GO term
- P-value: Bonferroni-corrected p-value of Fisher's exact test
- *red*: go terms not related to the input proteins
- *blue*: go terms related to the input proteins (enriched uniquely by network-based method)
- *green*: go terms ancestors of terms enriched with the standard method (enriched uniquely by network-based method)

1 Standard enrichment

GO Term	N1	N2	P-value	Description
GO:0044707	9	4361	0.000148596	single-multicellular organism process
GO:0007596	5	501	0.000161285	blood coagulation
GO:0050817	5	501	0.000161285	coagulation
GO:0007599	5	510	0.000176152	hemostasis
GO:0032501	9	4447	0.000176367	multicellular organismal process
GO:0042221	9	4712	0.00029289	response to chemical
GO:0050878	5	717	0.000946352	regulation of body fluid levels
GO:0065008	8	3888	0.00143973	regulation of biological quality
GO:0006534	2	10	0.00320327	cysteine metabolic process
GO:0051409	2	11	0.00391448	response to nitrosative stress
GO:0051090	4	415	0.00412819	regulation of sequence-specific DNA binding transcription factor activity
GO:0048583	8	4515	0.00453816	regulation of response to stimulus
GO:1901700	6	1851	0.0047547	response to oxygen-containing compound
GO:0002714	2	16	0.0085339	positive regulation of B cell mediated immunity
GO:0002891	2	16	0.0085339	positive regulation of immunoglobulin mediated immune response
GO:0033993	5	1142	0.0092079	response to lipid
GO:1903034	4	529	0.0107485	regulation of response to wounding
GO:0051900	2	19	0.012155	regulation of mitochondrial depolarization
GO:0003013	3	173	0.0140292	circulatory system process
GO:0048523	8	5279	0.0149286	negative regulation of cellular process
GO:0010605	6	2452	0.023969	negative regulation of macromolecule metabolic process
GO:0006750	2	27	0.024918	glutathione biosynthetic process
GO:1901685	2	27	0.024918	glutathione derivative metabolic process
GO:1901687	2	27	0.024918	glutathione derivative biosynthetic process
GO:0048519	8	5756	0.0287086	negative regulation of biological process
GO:0006536	2	29	0.0288134	glutamate metabolic process
GO:0048522	8	5768	0.0291627	positive regulation of cellular process
GO:0019184	2	30	0.0308666	nonribosomal peptide biosynthetic process
GO:0006950	7	4134	0.0383894	response to stress
GO:0032103	3	245	0.0395951	positive regulation of response to external stimulus
GO:0009892	6	2679	0.0396979	negative regulation of metabolic process
GO:0051091	3	264	0.0494339	positive regulation of sequence-specific DNA binding transcription factor activity

Table 2: Overrepresented GO terms with the standard enrichment

2 Network-based enrichment

GO Term	N1	N2	P-value	Description
GO:0043207	9	2065	4.69135e-06	response to external biotic stimulus
GO:0006954	8	1314	4.69516e-06	inflammatory response
GO:0032101	9	2173	7.36046e-06	regulation of response to external stimulus
GO:0009607	9	2197	8.10999e-06	response to biotic stimulus
GO:0051707	8	1426	8.937e-06	response to other organism
GO:0030334	8	1926	9.40782e-05	regulation of cell migration
GO:2000145	8	2039	0.000146693	regulation of cell motility
GO:0080134	9	3072	0.000154265	regulation of response to stress
GO:0051270	8	2182	0.000248481	regulation of cellular component movement
GO:0040012	8	2224	0.000288122	regulation of locomotion
GO:0032496	6	847	0.000409796	response to lipopolysaccharide
GO:0051241	7	1513	0.000484963	negative regulation of multicellular organismal process
GO:0016337	6	900	0.000585532	single organismal cell-cell adhesion
GO:0002237	6	918	0.000657751	response to molecule of bacterial origin
GO:0002822	5	464	0.000713958	regulation of adaptive immune response based on somatic recombination of immune receptors built from imm[illegible]
GO:0098542	6	972	0.000919808	defense response to other organism
GO:0098602	6	981	0.000970864	single organism cell adhesion
GO:0002819	5	507	0.00110565	regulation of adaptive immune response
GO:0030335	6	1024	0.00124817	positive regulation of cell migration
GO:2000147	6	1042	0.00138215	positive regulation of cell motility
GO:0001817	7	1774	0.00142853	regulation of cytokine production
GO:0051272	6	1060	0.00152775	positive regulation of cellular component movement
GO:0009725	8	2824	0.00182325	response to hormone
GO:0040017	6	1124	0.00215162	positive regulation of locomotion
GO:0001101	6	1170	0.00271859	response to acid chemical
GO:2001234	5	666	0.00422439	negative regulation of apoptotic signaling pathway
GO:0007584	5	690	0.0050236	response to nutrient
GO:0051240	7	2185	0.00581711	positive regulation of multicellular organismal process
GO:0045765	5	715	0.0059785	regulation of angiogenesis
GO:1901342	5	774	0.00880388	regulation of vasculature development
GO:0003254	3	98	0.0101471	regulation of membrane depolarization
GO:0050776	7	2377	0.010221	regulation of immune response
GO:0042493	6	1473	0.0103429	response to drug
GO:0051247	8	3557	0.010629	positive regulation of protein metabolic process
GO:0042592	8	3571	0.0109506	homeostatic process
GO:0051351	3	106	0.0128498	positive regulation of ligase activity
GO:0050727	5	853	0.0141295	regulation of inflammatory response
GO:0043069	7	2511	0.014733	negative regulation of programmed cell death
GO:0048520	4	383	0.0150751	positive regulation of behavior
GO:0032729	3	114	0.0159906	positive regulation of interferon-gamma production
GO:0043604	3	117	0.0172878	amide biosynthetic process
GO:0031347	6	1612	0.0173915	regulation of defense response
GO:0051896	4	402	0.0182431	regulation of protein kinase B signaling
GO:0071345	6	1640	0.0192013	cellular response to cytokine stimulus
GO:0010595	3	122	0.0196013	positive regulation of endothelial cell migration
GO:0060548	7	2642	0.0206586	negative regulation of cell death
GO:0002706	4	431	0.023994	regulation of lymphocyte mediated immunity
GO:0001819	5	963	0.0254432	positive regulation of cytokine production
GO:0051340	3	136	0.0271457	regulation of ligase activity
GO:0043603	4	447	0.0276884	cellular amide metabolic process
GO:0050920	4	450	0.0284253	regulation of chemotaxis
GO:0030162	6	1768	0.0295473	regulation of proteolysis
GO:0043524	4	459	0.0307229	negative regulation of neuron apoptotic process
GO:0007155	7	2817	0.0315935	cell adhesion
GO:0022610	7	2824	0.032116	biological adhesion
GO:0008219	7	2827	0.0323421	cell death
GO:0032269	6	1804	0.033159	negative regulation of cellular protein metabolic process
GO:0042398	3	146	0.033568	cellular modified amino acid biosynthetic process
GO:0016265	7	2848	0.0339629	death
GO:2000021	4	471	0.0339962	regulation of ion homeostasis

Table 3: Overrepresented terms with the network-based enrichment. Only terms not detected with the standard method.

GO Term	N1	N2	P-value	Description
GO:0051222	5	1034	0.035879	positive regulation of protein transport
GO:0002697	5	1046	0.0379326	regulation of immune effector process

Table 4: Overrepresented terms with the network-based enrichment. Only terms not detected with the standard method.
