# Supplementary material for: NET-GE: a novel NETwork-based Gene Enrichment for detecting biological processes associated to Mendelian diseases
Source: BMC Genomics. 2015 Jun 18;16(Suppl 8):S6. doi: 10.1186/1471-2164-16-S8-S6 (PMC4480278; doi:10.1186/1471-2164-16-S8-S6)
Supplement: Additional file 3 — Detailed results for the OMIM-derived benchmark set. The archive contains pdf documents listing the enriched terms for each one of the 244 diseases in the OMIM-derived benchmark set. [file 1471-2164-16-S8-S6-S3.tgz › SUPPMAT/OMIM608930.pdf]

#608930 MYASTHENIC SYNDROME, CONGENITAL, FAST-CHANNEL

**OMIM Gene ID**	**HGNC**	**UniProtAC**
100690	CHRNA1	P02708
100720	CHRND	Q07001
100725	CHRNE	Q04844

Table 1: OMIM - UniProtAC mapping

Legend

- N1: #input proteins associated to the significant GO term
- N2: #proteins associated to the significant GO term
- P-value: Bonferroni-corrected p-value of Fisher's exact test
- *red*: go terms not related to the input proteins
- *blue*: go terms related to the input proteins (enriched uniquely by network-based method)
- *green*: go terms ancestors of terms enriched with the standard method (enriched uniquely by network-based method)

1 Standard enrichment

GO Term	N1	N2	P-value	Description
GO:0048630	2	3	6.94969e-07	skeletal muscle tissue growth
GO:0006936	3	261	1.79805e-05	muscle contraction
GO:0003012	3	320	3.3209e-05	muscle system process
GO:0050879	2	20	4.40015e-05	multicellular organismal movement
GO:0050881	2	20	4.40015e-05	musculoskeletal movement
GO:0042391	3	353	4.46183e-05	regulation of membrane potential
GO:0007268	3	530	0.000151445	synaptic transmission
GO:0007267	3	859	0.000646173	cell-cell signaling
GO:0023052	3	913	0.000776017	signaling
GO:0044700	3	913	0.000776017	single organism signaling
GO:0098655	3	1072	0.00125677	cation transmembrane transport
GO:0007154	3	1103	0.00136909	cell communication
GO:0050905	2	125	0.00179147	neuromuscular process
GO:0034220	3	1538	0.00371458	ion transmembrane transport
GO:0006812	3	1582	0.00404281	cation transport
GO:0003008	3	1588	0.00408901	system process
GO:0048589	2	296	0.0100618	developmental growth
GO:0055085	3	2352	0.0132937	transmembrane transport
GO:0006811	3	2423	0.0145349	ion transport
GO:0070050	1	5	0.0218561	neuron cellular homeostasis
GO:0040007	2	569	0.0370605	growth

Table 2: Overrepresented GO terms with the standard enrichment

2 Network-based enrichment

GO Term	N1	N2	P-value	Description
GO:0046716	2	51	0.00119817	muscle cell cellular homeostasis
GO:0007528	2	106	0.0052239	neuromuscular junction development

Table 3: Overrepresented terms with the network-based enrichment. Only terms not detected with the standard method.
