# Supplementary material for: NET-GE: a novel NETwork-based Gene Enrichment for detecting biological processes associated to Mendelian diseases
Source: BMC Genomics. 2015 Jun 18;16(Suppl 8):S6. doi: 10.1186/1471-2164-16-S8-S6 (PMC4480278; doi:10.1186/1471-2164-16-S8-S6)
Supplement: Additional file 3 — Detailed results for the OMIM-derived benchmark set. The archive contains pdf documents listing the enriched terms for each one of the 244 diseases in the OMIM-derived benchmark set. [file 1471-2164-16-S8-S6-S3.tgz › SUPPMAT/OMIM608931.pdf]

#608931 MYASTHENIC SYNDROME, CONGENITAL, ASSOCIATED WITH ACETYLCHOLINE RECEPTOR

OMIM Gene ID	HGNC	UniProtAC
100710	CHRNB1	P11230
100725	CHRNE	Q04844
601296	MUSK	O15146
601592	RAPSN	Q13702

Table 1: OMIM - UniProtAC mapping

Legend

- N1: #input proteins associated to the significant GO term
- N2: #proteins associated to the significant GO term
- P-value: Bonferroni-corrected p-value of Fisher's exact test
- *red*: go terms not related to the input proteins
- *blue*: go terms related to the input proteins (enriched uniquely by network-based method)
- *green*: go terms ancestors of terms enriched with the standard method (enriched uniquely by network-based method)

1 Standard enrichment

GO Term	N1	N2	P-value	Description
GO:0007271	3	25	1.61129e-07	synaptic transmission, cholinergic
GO:0007268	3	530	0.00171111	synaptic transmission
GO:0043525	2	68	0.0030058	positive regulation of neuron apoptotic process
GO:1901216	2	84	0.00459715	positive regulation of neuron death
GO:0007267	3	859	0.00725262	cell-cell signaling
GO:0023052	3	913	0.00870048	signaling
GO:0044700	3	913	0.00870048	single organism signaling
GO:0007154	3	1103	0.0152908	cell communication
GO:2000539	1	1	0.0166389	regulation of protein geranylgeranylation
GO:2000541	1	1	0.0166389	positive regulation of protein geranylgeranylation
GO:0006936	2	261	0.0444654	muscle contraction
GO:0003008	3	1588	0.0452185	system process

Table 2: Overrepresented GO terms with the standard enrichment

2 Network-based enrichment

GO Term	N1	N2	P-value	Description
GO:0021545	3	82	3.58654e-05	cranial nerve development
GO:0035094	3	107	8.03381e-05	response to nicotine
GO:0021675	3	133	0.000155054	nerve development
GO:0001941	2	18	0.00103326	postsynaptic membrane organization
GO:0044802	4	1291	0.00140308	single-organism membrane organization
GO:0061024	4	1750	0.00474309	membrane organization
GO:0043279	3	424	0.00506923	response to alkaloid
GO:0007610	4	1993	0.00798212	behavior
GO:0030534	3	605	0.0146975	adult behavior
GO:0050877	4	2735	0.0283319	neurological system process
GO:0003012	3	815	0.035803	muscle system process
GO:0042391	3	824	0.0369961	regulation of membrane potential
GO:0007528	2	106	0.0374499	neuromuscular junction development
GO:0007626	3	829	0.0376702	locomotory behavior

Table 3: Overrepresented terms with the network-based enrichment. Only terms not detected with the standard method.
