# Supplementary material for: NET-GE: a novel NETwork-based Gene Enrichment for detecting biological processes associated to Mendelian diseases
Source: BMC Genomics. 2015 Jun 18;16(Suppl 8):S6. doi: 10.1186/1471-2164-16-S8-S6 (PMC4480278; doi:10.1186/1471-2164-16-S8-S6)
Supplement: Additional file 3 — Detailed results for the OMIM-derived benchmark set. The archive contains pdf documents listing the enriched terms for each one of the 244 diseases in the OMIM-derived benchmark set. [file 1471-2164-16-S8-S6-S3.tgz › SUPPMAT/OMIM608971.pdf]

#608971 SEVERE COMBINED IMMUNODEFICIENCY, AUTOSOMAL RECESSIVE, T CELL-NEGATIVE,

OMIM Gene ID	HGNC	UniProtAC
146661	IL7R	P16871
151460	PTPRC	P08575

Table 1: OMIM - UniProtAC mapping

Legend

- N1: #input proteins associated to the significant GO term
- N2: #proteins associated to the significant GO term
- P-value: Bonferroni-corrected p-value of Fisher's exact test
- *red*: go terms not related to the input proteins
- *blue*: go terms related to the input proteins (enriched uniquely by network-based method)
- *green*: go terms ancestors of terms enriched with the standard method (enriched uniquely by network-based method)

1 Standard enrichment

GO Term	N1	N2	P-value	Description
GO:0001915	2	7	8.69781e-06	negative regulation of T cell mediated cytotoxicity
GO:0001911	2	12	2.7336e-05	negative regulation of leukocyte mediated cytotoxicity
GO:0031342	2	12	2.7336e-05	negative regulation of cell killing
GO:0002710	2	17	5.63288e-05	negative regulation of T cell mediated immunity
GO:0002823	2	24	0.000114314	negative regulation of adaptive immune response based on somatic recombination of immune receptors built f
GO:0002707	2	25	0.000124255	negative regulation of lymphocyte mediated immunity
GO:0002704	2	27	0.000145378	negative regulation of leukocyte mediated immunity
GO:0002820	2	29	0.000168158	negative regulation of adaptive immune response
GO:0042100	2	44	0.000391816	B cell proliferation
GO:0002698	2	107	0.00234882	negative regulation of immune effector process
GO:0001914	2	109	0.00243787	regulation of T cell mediated cytotoxicity
GO:0050777	2	118	0.0028591	negative regulation of immune response
GO:0046651	2	132	0.003581	lymphocyte proliferation
GO:0001910	2	134	0.00369077	regulation of leukocyte mediated cytotoxicity
GO:0032943	2	134	0.00369077	mononuclear cell proliferation
GO:0070661	2	143	0.0042052	leukocyte proliferation
GO:0031341	2	144	0.0042644	regulation of cell killing
GO:0002709	2	151	0.00469062	regulation of T cell mediated immunity
GO:0030217	2	154	0.00487948	T cell differentiation
GO:0042113	2	186	0.00712599	B cell activation
GO:0002706	2	222	0.0101603	regulation of lymphocyte mediated immunity
GO:0002822	2	235	0.0113879	regulation of adaptive immune response based on somatic recombination of immune receptors built from imm
GO:0002819	2	246	0.0124814	regulation of adaptive immune response
GO:0002703	2	259	0.0138382	regulation of leukocyte mediated immunity
GO:0030098	2	263	0.0142698	lymphocyte differentiation
GO:0050870	2	267	0.014708	positive regulation of T cell activation
GO:0048534	2	272	0.0152651	hematopoietic or lymphoid organ development
GO:0042110	2	319	0.0210077	T cell activation
GO:0051251	2	345	0.0245775	positive regulation of lymphocyte activation
GO:0002683	2	357	0.0263196	negative regulation of immune system process
GO:0002696	2	367	0.0278168	positive regulation of leukocyte activation
GO:0002521	2	375	0.0290445	leukocyte differentiation
GO:0050867	2	380	0.0298251	positive regulation of cell activation
GO:0050863	2	385	0.0306163	regulation of T cell activation
GO:0002378	1	2	0.0312638	immunoglobulin biosynthetic process
GO:0038111	1	2	0.0312638	interleukin-7-mediated signaling pathway
GO:2000471	1	2	0.0312638	regulation of hematopoietic stem cell migration
GO:2000473	1	2	0.0312638	positive regulation of hematopoietic stem cell migration
GO:0002697	2	463	0.0442981	regulation of immune effector process
GO:0046649	2	484	0.0484122	lymphocyte activation

Table 2: Overrepresented GO terms with the standard enrichment

2 Network-based enrichment

No novel enriched terms
