# Supplementary material for: NET-GE: a novel NETwork-based Gene Enrichment for detecting biological processes associated to Mendelian diseases
Source: BMC Genomics. 2015 Jun 18;16(Suppl 8):S6. doi: 10.1186/1471-2164-16-S8-S6 (PMC4480278; doi:10.1186/1471-2164-16-S8-S6)
Supplement: Additional file 3 — Detailed results for the OMIM-derived benchmark set. The archive contains pdf documents listing the enriched terms for each one of the 244 diseases in the OMIM-derived benchmark set. [file 1471-2164-16-S8-S6-S3.tgz › SUPPMAT/OMIM609015.pdf]

#609015 TRIFUNCTIONAL PROTEIN DEFICIENCY

OMIM Gene ID	HGNC	UniProtAC
143450	HADHB	P55084
600890	HADHA	P40939

Table 1: OMIM - UniProtAC mapping

Legend

- N1: #input proteins associated to the significant GO term
- N2: #proteins associated to the significant GO term
- P-value: Bonferroni-corrected p-value of Fisher's exact test
- *red*: go terms not related to the input proteins
- *blue*: go terms related to the input proteins (enriched uniquely by network-based method)
- *green*: go terms ancestors of terms enriched with the standard method (enriched uniquely by network-based method)

1 Standard enrichment

GO Term	N1	N2	P-value	Description
GO:0035965	2	7	1.8575e-06	cardiolipin acyl-chain remodeling
GO:0032048	2	13	6.89926e-06	cardiolipin metabolic process
GO:0046471	2	34	4.96218e-05	phosphatidylglycerol metabolic process
GO:0006635	2	56	0.000136217	fatty acid beta-oxidation
GO:0019395	2	80	0.00027951	fatty acid oxidation
GO:0034440	2	81	0.000286586	lipid oxidation
GO:0009062	2	83	0.000301003	fatty acid catabolic process
GO:0072329	2	106	0.000492237	monocarboxylic acid catabolic process
GO:0044242	2	188	0.00155481	cellular lipid catabolic process
GO:0046474	2	259	0.00295528	glycerophospholipid biosynthetic process
GO:0030258	2	266	0.0031175	lipid modification
GO:0016054	2	278	0.00340568	organic acid catabolic process
GO:0046395	2	278	0.00340568	carboxylic acid catabolic process
GO:0045017	2	293	0.00378381	glycerolipid biosynthetic process
GO:0008654	2	316	0.00440227	phospholipid biosynthetic process
GO:0016042	2	323	0.00459979	lipid catabolic process
GO:0044282	2	358	0.00565237	small molecule catabolic process
GO:0006650	2	416	0.00763522	glycerophospholipid metabolic process
GO:0006631	2	421	0.00782006	fatty acid metabolic process
GO:0046486	2	522	0.0120278	glycerolipid metabolic process
GO:0006644	2	531	0.0124466	phospholipid metabolic process
GO:0032787	2	767	0.0259838	monocarboxylic acid metabolic process
GO:0008610	2	768	0.0260517	lipid biosynthetic process
GO:0090407	2	789	0.0274968	organophosphate biosynthetic process

Table 2: Overrepresented GO terms with the standard enrichment

2 Network-based enrichment

No novel enriched terms
