# Supplementary material for: NET-GE: a novel NETwork-based Gene Enrichment for detecting biological processes associated to Mendelian diseases
Source: BMC Genomics. 2015 Jun 18;16(Suppl 8):S6. doi: 10.1186/1471-2164-16-S8-S6 (PMC4480278; doi:10.1186/1471-2164-16-S8-S6)
Supplement: Additional file 3 — Detailed results for the OMIM-derived benchmark set. The archive contains pdf documents listing the enriched terms for each one of the 244 diseases in the OMIM-derived benchmark set. [file 1471-2164-16-S8-S6-S3.tgz › SUPPMAT/OMIM609135.pdf]

# #609135 APLASTIC ANEMIA

| OMIM Gene ID | HGNC | UniProtAC |
|---|---|---|
| 147570 | IFNG | P01579 |
| 602667 | NBN | O60934 |

Table 1: OMIM - UniProtAC mapping

Legend

- N1: #input proteins associated to the significant GO term
- N2: #proteins associated to the significant GO term
- P-value: Bonferroni-corrected p-value of Fisher's exact test
- *red*: go terms not related to the input proteins
- *blue*: go terms related to the input proteins (enriched uniquely by network-based method)
- *green*: go terms ancestors of terms enriched with the standard method (enriched uniquely by network-based method)

# 1 Standard enrichment

| GO Term | N1 | N2 | P-value | Description |
|---|---|---|---|---|
| GO:0002285 | 2 | 121 | 0.00543292 | lymphocyte activation involved in immune response |
| GO:0002263 | 2 | 170 | 0.0107498 | cell activation involved in immune response |
| GO:0002366 | 2 | 170 | 0.0107498 | leukocyte activation involved in immune response |
| GO:0007050 | 2 | 189 | 0.0132949 | cell cycle arrest |
| GO:0032113 | 1 | 1 | 0.0282437 | regulation of carbohydrate phosphatase activity |
| GO:0060549 | 1 | 1 | 0.0282437 | regulation of fructose 1,6-bisphosphate 1-phosphatase activity |
| GO:0060550 | 1 | 1 | 0.0282437 | positive regulation of fructose 1,6-bisphosphate 1-phosphatase activity |
| GO:0060551 | 1 | 1 | 0.0282437 | regulation of fructose 1,6-bisphosphate metabolic process |
| GO:0060552 | 1 | 1 | 0.0282437 | positive regulation of fructose 1,6-bisphosphate metabolic process |
| GO:0045786 | 2 | 343 | 0.0438921 | negative regulation of cell cycle |

Table 2: Overrepresented GO terms with the standard enrichment

# 2 Network-based enrichment

| GO Term | N1 | N2 | P-value | Description |
|---|---|---|---|---|
| GO:0045911 | 2 | 55 | 0.00268405 | positive regulation of DNA recombination |
| GO:0000018 | 2 | 154 | 0.0212935 | regulation of DNA recombination |

Table 3: Overrepresented terms with the network-based enrichment. Only terms not detected with the standard method.
