# Supplementary material for: NET-GE: a novel NETwork-based Gene Enrichment for detecting biological processes associated to Mendelian diseases
Source: BMC Genomics. 2015 Jun 18;16(Suppl 8):S6. doi: 10.1186/1471-2164-16-S8-S6 (PMC4480278; doi:10.1186/1471-2164-16-S8-S6)
Supplement: Additional file 3 — Detailed results for the OMIM-derived benchmark set. The archive contains pdf documents listing the enriched terms for each one of the 244 diseases in the OMIM-derived benchmark set. [file 1471-2164-16-S8-S6-S3.tgz › SUPPMAT/OMIM609532.pdf]

#609532 HEPATITIS C VIRUS, SUSCEPTIBILITY TO

**OMIM Gene ID**	**HGNC**	**UniProtAC**
147570	IFNG	P01579
151460	PTPRC	P08575
601373	CCR5	P51681
607402	IFNL3	Q8IZI9

Table 1: OMIM - UniProtAC mapping

Legend

- N1: #input proteins associated to the significant GO term
- N2: #proteins associated to the significant GO term
- P-value: Bonferroni-corrected p-value of Fisher's exact test
- *red*: go terms not related to the input proteins
- *blue*: go terms related to the input proteins (enriched uniquely by network-based method)
- *green*: go terms ancestors of terms enriched with the standard method (enriched uniquely by network-based method)

1 Standard enrichment

GO Term	N1	N2	P-value	Description
GO:0043207	4	921	0.000216672	response to external biotic stimulus
GO:0009607	4	964	0.000260136	response to biotic stimulus
GO:0051607	3	192	0.000317625	defense response to virus
GO:0009615	3	321	0.00148989	response to virus
GO:0051704	4	1558	0.00177908	multi-organism process
GO:0006952	4	1580	0.00188181	defense response
GO:0098542	3	431	0.00360706	defense response to other organism
GO:0051209	2	41	0.00424239	release of sequestered calcium ion into cytosol
GO:0051283	2	41	0.00424239	negative regulation of sequestering of calcium ion
GO:0009605	4	1995	0.004787	response to external stimulus
GO:0051282	2	45	0.00512118	regulation of sequestering of calcium ion
GO:0060402	2	53	0.00712625	calcium ion transport into cytosol
GO:0060401	2	54	0.00740011	cytosolic calcium ion transport
GO:0051707	3	607	0.0100608	response to other organism
GO:0002376	4	2446	0.0108233	immune system process
GO:0002252	3	657	0.0127494	immune effector process
GO:0050871	2	87	0.0193231	positive regulation of B cell activation
GO:2000648	2	87	0.0193231	positive regulation of stem cell proliferation
GO:0042102	2	103	0.0271176	positive regulation of T cell proliferation
GO:0001959	2	115	0.0338246	regulation of cytokine-mediated signaling pathway
GO:0060759	2	122	0.0380774	regulation of response to cytokine stimulus
GO:0030595	2	123	0.0387054	leukocyte chemotaxis
GO:0050864	2	130	0.0432446	regulation of B cell activation
GO:0072091	2	137	0.0480343	regulation of stem cell proliferation

Table 2: Overrepresented GO terms with the standard enrichment

2 Network-based enrichment

GO Term	N1	N2	P-value	Description
GO:0031295	3	189	0.000936324	T cell costimulation
GO:0031294	3	190	0.000951324	lymphocyte costimulation
GO:0034393	2	13	0.00110262	positive regulation of smooth muscle cell apoptotic process
GO:0033028	2	15	0.00148417	myeloid cell apoptotic process
GO:0006925	2	17	0.00192221	inflammatory cell apoptotic process
GO:0050777	3	282	0.00312006	negative regulation of immune response
GO:0002698	3	285	0.00322086	negative regulation of immune effector process
GO:0002274	3	290	0.00339359	myeloid leukocyte activation
GO:0022407	3	297	0.00364563	regulation of cell-cell adhesion
GO:0050778	4	1484	0.00512986	positive regulation of immune response
GO:0043901	3	350	0.00596834	negative regulation of multi-organism process
GO:0010661	2	31	0.00656858	positive regulation of muscle cell apoptotic process
GO:0007204	3	371	0.00710847	positive regulation of cytosolic calcium ion concentration
GO:0002263	3	385	0.00794377	cell activation involved in immune response
GO:0002366	3	385	0.00794377	leukocyte activation involved in immune response
GO:0050671	3	392	0.00838483	positive regulation of lymphocyte proliferation
GO:0032946	3	396	0.00864402	positive regulation of mononuclear cell proliferation
GO:0034391	2	36	0.00889758	regulation of smooth muscle cell apoptotic process
GO:0048247	2	36	0.00889758	lymphocyte chemotaxis
GO:0008037	3	401	0.00897543	cell recognition
GO:0070665	3	403	0.00911032	positive regulation of leukocyte proliferation
GO:0050718	2	40	0.0110143	positive regulation of interleukin-1 beta secretion
GO:0002706	3	431	0.0111426	regulation of lymphocyte mediated immunity
GO:0051044	2	41	0.0115787	positive regulation of membrane protein ectodomain proteolysis
GO:0042129	3	439	0.011774	regulation of T cell proliferation
GO:0050716	2	42	0.0121571	positive regulation of interleukin-1 secretion
GO:0051480	3	457	0.0132806	cytosolic calcium ion homeostasis
GO:0002822	3	464	0.0138995	regulation of adaptive immune response based on somatic recombination of immune receptors built from imm
GO:2000021	3	471	0.0145371	regulation of ion homeostasis
GO:0036230	2	50	0.0172911	granulocyte activation
GO:0050706	2	50	0.0172911	regulation of interleukin-1 beta secretion
GO:0050731	3	503	0.0177002	positive regulation of peptidyl-tyrosine phosphorylation
GO:0002819	3	507	0.0181251	regulation of adaptive immune response
GO:0043903	3	510	0.0184482	regulation of symbiosis, encompassing mutualism through parasitism
GO:0042104	2	53	0.0194484	positive regulation of activated T cell proliferation
GO:0051043	2	53	0.0194484	regulation of membrane protein ectodomain proteolysis
GO:0002861	2	54	0.0201957	regulation of inflammatory response to antigenic stimulus
GO:0002703	3	528	0.0204669	regulation of leukocyte mediated immunity
GO:0043270	3	532	0.0209347	positive regulation of ion transport
GO:0002699	3	536	0.0214093	positive regulation of immune effector process
GO:0050704	2	56	0.0217322	regulation of interleukin-1 secretion
GO:0051924	3	553	0.0235067	regulation of calcium ion transport
GO:0071887	2	61	0.0258195	leukocyte apoptotic process
GO:0032731	2	63	0.0275526	positive regulation of interleukin-1 beta production
GO:0002684	4	2287	0.0289769	positive regulation of immune system process
GO:0050670	3	605	0.0307589	regulation of lymphocyte proliferation
GO:0032944	3	609	0.0313712	regulation of mononuclear cell proliferation
GO:0032732	2	69	0.0330891	positive regulation of interleukin-1 production
GO:0072676	2	69	0.0330891	lymphocyte migration
GO:0070663	3	621	0.0332564	regulation of leukocyte proliferation
GO:0050776	4	2377	0.0338179	regulation of immune response
GO:0002820	2	71	0.0350468	negative regulation of adaptive immune response
GO:0045785	3	632	0.0350493	positive regulation of cell adhesion
GO:0050900	3	635	0.0355492	leukocyte migration
GO:0002429	3	638	0.0360538	immune response-activating cell surface receptor signaling pathway
GO:0050870	3	647	0.037596	positive regulation of T cell activation
GO:0050730	3	663	0.0404444	regulation of peptidyl-tyrosine phosphorylation
GO:0048534	3	682	0.0440086	hematopoietic or lymphoid organ development
GO:0032735	2	84	0.0491387	positive regulation of interleukin-12 production

Table 3: Overrepresented terms with the network-based enrichment. Only terms not detected with the standard method.
