# Supplementary material for: NET-GE: a novel NETwork-based Gene Enrichment for detecting biological processes associated to Mendelian diseases
Source: BMC Genomics. 2015 Jun 18;16(Suppl 8):S6. doi: 10.1186/1471-2164-16-S8-S6 (PMC4480278; doi:10.1186/1471-2164-16-S8-S6)
Supplement: Additional file 3 — Detailed results for the OMIM-derived benchmark set. The archive contains pdf documents listing the enriched terms for each one of the 244 diseases in the OMIM-derived benchmark set. [file 1471-2164-16-S8-S6-S3.tgz › SUPPMAT/OMIM610424.pdf]

#610424 HEPATITIS B VIRUS, SUSCEPTIBILITY TO

OMIM Gene ID	HGNC	UniProtAC
107470	IFNGR1	P15260
123889	IL10RB	Q08334
602376	IFNAR2	P48551

Table 1: OMIM - UniProtAC mapping

Legend

- N1: #input proteins associated to the significant GO term
- N2: #proteins associated to the significant GO term
- P-value: Bonferroni-corrected p-value of Fisher's exact test
- *red*: go terms not related to the input proteins
- *blue*: go terms related to the input proteins (enriched uniquely by network-based method)
- *green*: go terms ancestors of terms enriched with the standard method (enriched uniquely by network-based method)

1 Standard enrichment

GO Term	N1	N2	P-value	Description
GO:0009615	3	321	4.26647e-05	response to virus
GO:0019221	3	546	0.000210771	cytokine-mediated signaling pathway
GO:0051707	3	607	0.000289761	response to other organism
GO:0071345	3	725	0.000494127	cellular response to cytokine stimulus
GO:0034097	3	880	0.000884282	response to cytokine
GO:0043207	3	921	0.00101388	response to external biotic stimulus
GO:0009607	3	964	0.00116279	response to biotic stimulus
GO:0001959	2	115	0.00192882	regulation of cytokine-mediated signaling pathway
GO:0060759	2	122	0.00217161	regulation of response to cytokine stimulus
GO:0051704	3	1558	0.00491461	multi-organism process
GO:0009605	3	1995	0.0103228	response to external stimulus
GO:0045088	2	320	0.0149642	regulation of innate immune response
GO:0071310	3	2482	0.019884	cellular response to organic substance
GO:0070887	3	2904	0.0318541	cellular response to chemical stimulus

Table 2: Overrepresented GO terms with the standard enrichment

2 Network-based enrichment

GO Term	N1	N2	P-value	Description
GO:0002252	3	1396	0.0111606	immune effector process
GO:0031347	3	1612	0.0171889	regulation of defense response

Table 3: Overrepresented terms with the network-based enrichment. Only terms not detected with the standard method.
