# Supplementary material for: NET-GE: a novel NETwork-based Gene Enrichment for detecting biological processes associated to Mendelian diseases
Source: BMC Genomics. 2015 Jun 18;16(Suppl 8):S6. doi: 10.1186/1471-2164-16-S8-S6 (PMC4480278; doi:10.1186/1471-2164-16-S8-S6)
Supplement: Additional file 3 — Detailed results for the OMIM-derived benchmark set. The archive contains pdf documents listing the enriched terms for each one of the 244 diseases in the OMIM-derived benchmark set. [file 1471-2164-16-S8-S6-S3.tgz › SUPPMAT/OMIM610799.pdf]

#610799 INVASIVE PNEUMOCOCCAL DISEASE, RECURRENT ISOLATED, 1; IPD1

OMIM Gene ID	HGNC	UniProtAC
606252	TIRAP	P58753
606883	IRAK4	Q9NWZ3

Table 1: OMIM - UniProtAC mapping

Legend

- N1: #input proteins associated to the significant GO term
- N2: #proteins associated to the significant GO term
- P-value: Bonferroni-corrected p-value of Fisher's exact test
- *red*: go terms not related to the input proteins
- *blue*: go terms related to the input proteins (enriched uniquely by network-based method)
- *green*: go terms ancestors of terms enriched with the standard method (enriched uniquely by network-based method)

1 Standard enrichment

GO Term	N1	N2	P-value	Description
GO:0038123	2	71	0.000945505	toll-like receptor TLR1:TLR2 signaling pathway
GO:0038124	2	71	0.000945505	toll-like receptor TLR6:TLR2 signaling pathway
GO:0034134	2	73	0.000999917	toll-like receptor 2 signaling pathway
GO:0002755	2	88	0.0014565	MyD88-dependent toll-like receptor signaling pathway
GO:0034142	2	99	0.00184573	toll-like receptor 4 signaling pathway
GO:0002224	2	139	0.00364923	toll-like receptor signaling pathway
GO:0002221	2	166	0.00521073	pattern recognition receptor signaling pathway
GO:0002758	2	169	0.00540138	innate immune response-activating signal transduction
GO:0002218	2	181	0.0061981	activation of innate immune response
GO:0043123	2	211	0.00842964	positive regulation of I-kappaB kinase/NF-kappaB signaling
GO:0045089	2	224	0.00950299	positive regulation of innate immune response
GO:0032658	1	1	0.0143603	regulation of interleukin-15 production
GO:0032738	1	1	0.0143603	positive regulation of interleukin-15 production
GO:0035664	1	1	0.0143603	TIRAP-dependent toll-like receptor signaling pathway
GO:0035665	1	1	0.0143603	TIRAP-dependent toll-like receptor 4 signaling pathway
GO:2000338	1	1	0.0143603	regulation of chemokine (C-X-C motif) ligand 1 production
GO:2000340	1	1	0.0143603	positive regulation of chemokine (C-X-C motif) ligand 1 production
GO:0043122	2	292	0.0161653	regulation of I-kappaB kinase/NF-kappaB signaling
GO:0045088	2	320	0.01942	regulation of innate immune response
GO:0031349	2	321	0.0195417	positive regulation of defense response
GO:0034137	1	2	0.0287203	positive regulation of toll-like receptor 2 signaling pathway
GO:0034141	1	3	0.0430798	positive regulation of toll-like receptor 3 signaling pathway
GO:0002757	2	508	0.0489982	immune response-activating signal transduction

Table 2: Overrepresented GO terms with the standard enrichment

2 Network-based enrichment

GO Term	N1	N2	P-value	Description
GO:0034145	2	13	4.23083e-05	positive regulation of toll-like receptor 4 signaling pathway
GO:0034123	2	38	0.000381316	positive regulation of toll-like receptor signaling pathway
GO:0034121	2	107	0.00307601	regulation of toll-like receptor signaling pathway
GO:0001816	2	232	0.0145345	cytokine production
GO:0001959	2	285	0.0219514	regulation of cytokine-mediated signaling pathway
GO:0060759	2	318	0.0273392	regulation of response to cytokine stimulus

Table 3: Overrepresented terms with the network-based enrichment. Only terms not detected with the standard method.
