# Supplementary material for: NET-GE: a novel NETwork-based Gene Enrichment for detecting biological processes associated to Mendelian diseases
Source: BMC Genomics. 2015 Jun 18;16(Suppl 8):S6. doi: 10.1186/1471-2164-16-S8-S6 (PMC4480278; doi:10.1186/1471-2164-16-S8-S6)
Supplement: Additional file 3 — Detailed results for the OMIM-derived benchmark set. The archive contains pdf documents listing the enriched terms for each one of the 244 diseases in the OMIM-derived benchmark set. [file 1471-2164-16-S8-S6-S3.tgz › SUPPMAT/OMIM611162.pdf]

#611162 MALARIA, SUSCEPTIBILITY TO

OMIM Gene ID	HGNC	UniProtAC
109270	SLC4A1	P02730
110750	GYPC	P04921
111300	GYPA	P02724
111740	GYPB	P06028
120620	CR1	P17927
141900	HBB	P68871
146790	FCGR2A	P12318
147840	ICAM1	P05362
163730	NOS2	P35228
173510	CD36	P16671
191160	TNF	P01375
305900	G6PD	P11413
602441	CISH	Q9NSE2
604590	FCGR2B	P31994
606252	TIRAP	P58753
613665	ACKR1	Q16570

Table 1: OMIM - UniProtAC mapping

Legend

- N1: #input proteins associated to the significant GO term
- N2: #proteins associated to the significant GO term
- P-value: Bonferroni-corrected p-value of Fisher's exact test
- *red*: go terms not related to the input proteins
- *blue*: go terms related to the input proteins (enriched uniquely by network-based method)
- *green*: go terms ancestors of terms enriched with the standard method (enriched uniquely by network-based method)

1 Standard enrichment

GO Term	N1	N2	P-value	Description
GO:0071219	5	198	8.25177e-06	cellular response to molecule of bacterial origin
GO:0071216	5	215	1.24653e-05	cellular response to biotic stimulus
GO:0051707	6	607	5.15205e-05	response to other organism
GO:0002684	7	1093	5.45866e-05	positive regulation of immune system process
GO:0002682	8	1758	5.77397e-05	regulation of immune system process
GO:0050776	7	1167	8.53944e-05	regulation of immune response
GO:0006950	10	4134	0.000183926	response to stress
GO:0044126	3	32	0.000227048	regulation of growth of symbiont in host
GO:0044130	3	32	0.000227048	negative regulation of growth of symbiont in host
GO:0044144	3	32	0.000227048	modulation of growth of symbiont involved in interaction with host
GO:0044146	3	32	0.000227048	negative regulation of growth of symbiont involved in interaction with host
GO:0032496	5	389	0.000238394	response to lipopolysaccharide
GO:0006955	7	1414	0.000315179	immune response
GO:0002237	5	414	0.000324382	response to molecule of bacterial origin
GO:0045429	3	38	0.00038566	positive regulation of nitric oxide biosynthetic process
GO:0002697	5	463	0.000563398	regulation of immune effector process
GO:0043207	6	921	0.00059832	response to external biotic stimulus
GO:0051704	7	1558	0.000607445	multi-organism process
GO:0006952	7	1580	0.000667748	defense response
GO:0071222	4	191	0.000693328	cellular response to lipopolysaccharide
GO:0002376	8	2446	0.00073562	immune system process
GO:0009607	6	964	0.000781087	response to biotic stimulus
GO:2000343	2	4	0.000868941	positive regulation of chemokine (C-X-C motif) ligand 2 production
GO:0045428	3	56	0.00126227	regulation of nitric oxide biosynthetic process
GO:0042742	4	233	0.00153051	defense response to bacterium
GO:1901701	6	1086	0.00156371	cellular response to oxygen-containing compound
GO:1901700	7	1851	0.00193595	response to oxygen-containing compound
GO:0002703	4	259	0.00232994	regulation of leukocyte mediated immunity
GO:0009617	4	261	0.00240215	response to bacterium
GO:0032642	3	71	0.0025941	regulation of chemokine production
GO:0070887	8	2904	0.00271581	cellular response to chemical stimulus
GO:0050896	13	11721	0.00281064	response to stimulus
GO:2000341	2	7	0.00303936	regulation of chemokine (C-X-C motif) ligand 2 production
GO:0009605	7	1995	0.00319473	response to external stimulus
GO:0050830	3	78	0.00344759	defense response to Gram-positive bacterium
GO:0051716	12	9450	0.00368742	cellular response to stimulus
GO:0002687	3	84	0.00431239	positive regulation of leukocyte migration
GO:0048583	9	4515	0.00641445	regulation of response to stimulus
GO:0050778	5	770	0.00679747	positive regulation of immune response
GO:0045087	5	825	0.00950012	innate immune response
GO:0071220	2	12	0.00954216	cellular response to bacterial lipoprotein
GO:0071221	2	12	0.00954216	cellular response to bacterial lipopeptide
GO:0051240	5	866	0.0120146	positive regulation of multicellular organismal process
GO:0002685	3	119	0.012298	regulation of leukocyte migration
GO:0002923	2	14	0.013151	regulation of humoral immune response mediated by circulating immunoglobulin
GO:0070391	2	14	0.013151	response to lipoteichoic acid
GO:0071223	2	14	0.013151	cellular response to lipoteichoic acid
GO:0043900	4	406	0.0137508	regulation of multi-organism process
GO:0032675	3	126	0.0145968	regulation of interleukin-6 production
GO:0070339	2	16	0.0173347	response to bacterial lipopeptide
GO:0098542	4	431	0.0173857	defense response to other organism
GO:0007263	2	18	0.0220924	nitric oxide mediated signal transduction
GO:0032493	2	18	0.0220924	response to bacterial lipoprotein
GO:0051092	3	148	0.0236264	positive regulation of NF-kappaB transcription factor activity
GO:0065008	8	3888	0.0241852	regulation of biological quality
GO:0070374	3	150	0.0245932	positive regulation of ERK1 and ERK2 cascade
GO:0043901	3	154	0.0266043	negative regulation of multi-organism process
GO:0006954	4	483	0.0271587	inflammatory response
GO:0002757	4	508	0.033077	immune response-activating signal transduction
GO:0071396	4	529	0.0387393	cellular response to lipid

Table 2: Overrepresented GO terms with the standard enrichment

**GO Term**	**N1**	**N2**	**P-value**	**Description**
GO:0019932	3	176	0.0396191	second-messenger-mediated signaling
GO:0043410	4	539	0.0416725	positive regulation of MAPK cascade
GO:0051246	7	2954	0.0424555	regulation of protein metabolic process
GO:0033993	5	1142	0.0454396	response to lipid

Table 3: Overrepresented GO terms with the standard enrichment

2 Network-based enrichment

GO Term	N1	N2	P-value	Description
GO:0002755	5	146	1.03439e-05	MyD88-dependent toll-like receptor signaling pathway
GO:0034142	5	172	2.35674e-05	toll-like receptor 4 signaling pathway
GO:0045471	6	417	3.992e-05	response to ethanol
GO:0002224	5	235	0.000112205	toll-like receptor signaling pathway
GO:0032735	4	84	0.000118838	positive regulation of interleukin-12 production
GO:0032755	4	111	0.000366237	positive regulation of interleukin-6 production
GO:0002221	5	306	0.000416739	pattern recognition receptor signaling pathway
GO:0038123	4	117	0.00045264	toll-like receptor TLR1:TLR2 signaling pathway
GO:0038124	4	117	0.00045264	toll-like receptor TLR6:TLR2 signaling pathway
GO:0002758	5	316	0.000488711	innate immune response-activating signal transduction
GO:0002253	7	1110	0.000538527	activation of immune response
GO:0034134	4	124	0.000571708	toll-like receptor 2 signaling pathway
GO:0019371	3	29	0.000633624	cyclooxygenase pathway
GO:0002218	5	341	0.000712317	activation of innate immune response
GO:0042592	10	3571	0.000844042	homeostatic process
GO:0010888	3	33	0.000945078	negative regulation of lipid storage
GO:0002764	7	1248	0.00118963	immune response-regulating signaling pathway
GO:0045088	6	751	0.00127995	regulation of innate immune response
GO:0043123	5	400	0.00156545	positive regulation of I-kappaB kinase/NF-kappaB signaling
GO:0042522	3	40	0.00170816	regulation of tyrosine phosphorylation of Stat5 protein
GO:0032655	4	166	0.00183864	regulation of interleukin-12 production
GO:0090023	3	43	0.00213191	positive regulation of neutrophil chemotaxis
GO:1902624	3	43	0.00213191	positive regulation of neutrophil migration
GO:0008217	5	438	0.00244624	regulation of blood pressure
GO:0046330	4	187	0.00295665	positive regulation of JNK cascade
GO:0002673	4	190	0.00315013	regulation of acute inflammatory response
GO:0080134	9	3072	0.00336547	regulation of response to stress
GO:0032757	3	50	0.00337951	positive regulation of interleukin-8 production
GO:0071624	3	51	0.00358976	positive regulation of granulocyte chemotaxis
GO:0045089	5	486	0.00407414	positive regulation of innate immune response
GO:0034599	5	502	0.00477425	cellular response to oxidative stress
GO:0050801	7	1555	0.0052061	ion homeostasis
GO:0015711	6	964	0.00546913	organic anion transport
GO:0070741	3	59	0.00559171	response to interleukin-6
GO:0002526	4	220	0.00564213	acute inflammatory response
GO:0090022	3	62	0.00649994	regulation of neutrophil chemotaxis
GO:0031347	7	1612	0.00661855	regulation of defense response
GO:0032874	4	230	0.00673029	positive regulation of stress-activated MAPK cascade
GO:1902622	3	63	0.0068231	regulation of neutrophil migration
GO:0001816	4	232	0.00696535	cytokine production
GO:0070304	4	232	0.00696535	positive regulation of stress-activated protein kinase signaling cascade
GO:0048878	8	2410	0.0071094	chemical homeostasis
GO:0042990	4	237	0.00757964	regulation of transcription factor import into nucleus
GO:0051209	3	72	0.0102224	release of sequestered calcium ion into cytosol
GO:0051283	3	72	0.0102224	negative regulation of sequestering of calcium ion
GO:0097305	6	1084	0.0107658	response to alcohol
GO:0032368	4	263	0.0114441	regulation of lipid transport
GO:0001817	7	1774	0.012503	regulation of cytokine production
GO:0002693	2	8	0.0125102	positive regulation of cellular extravasation
GO:0045907	3	78	0.0130184	positive regulation of vasoconstriction
GO:0051282	3	78	0.0130184	regulation of sequestering of calcium ion
GO:0048660	4	272	0.0130716	regulation of smooth muscle cell proliferation
GO:0030193	4	273	0.0132624	regulation of blood coagulation
GO:1900046	4	273	0.0132624	regulation of hemostasis
GO:0043122	5	630	0.0144478	regulation of I-kappaB kinase/NF-kappaB signaling
GO:0071622	3	82	0.0151383	regulation of granulocyte chemotaxis
GO:0001959	4	285	0.0157172	regulation of cytokine-mediated signaling pathway
GO:0050878	7	1837	0.0157499	regulation of body fluid levels
GO:0050818	4	299	0.0189889	regulation of coagulation
GO:0006873	6	1202	0.0194861	cellular ion homeostasis

Table 4: Overrepresented terms with the network-based enrichment. Only terms not detected with the standard method.

GO Term	N1	N2	P-value	Description
GO:0030890	3	90	0.0200385	positive regulation of B cell proliferation
GO:0071803	2	10	0.0200952	positive regulation of podosome assembly
GO:0032680	4	306	0.0208015	regulation of tumor necrosis factor production
GO:0014070	8	2783	0.0209011	response to organic cyclic compound
GO:0010743	3	93	0.0221162	regulation of macrophage derived foam cell differentiation
GO:0010883	3	93	0.0221162	regulation of lipid storage
GO:0002720	3	94	0.0228391	positive regulation of cytokine production involved in immune response
GO:0007155	8	2817	0.0228797	cell adhesion
GO:0022610	8	2824	0.0233063	biological adhesion
GO:0060759	4	318	0.0242025	regulation of response to cytokine stimulus
GO:1903034	6	1274	0.027176	regulation of response to wounding
GO:0051099	4	331	0.0283327	positive regulation of binding
GO:0031349	5	726	0.0287168	positive regulation of defense response
GO:0034097	7	2017	0.0291545	response to cytokine
GO:0034146	3	104	0.0309441	toll-like receptor 5 signaling pathway
GO:0034166	3	105	0.0318454	toll-like receptor 10 signaling pathway
GO:0009991	6	1313	0.0322759	response to extracellular stimulus
GO:0042531	3	106	0.0327642	positive regulation of tyrosine phosphorylation of STAT protein
GO:0034121	3	107	0.0337003	regulation of toll-like receptor signaling pathway
GO:0035094	3	107	0.0337003	response to nicotine
GO:0001775	7	2070	0.0345619	cell activation
GO:0002702	3	108	0.0346536	positive regulation of production of molecular mediator of immune response
GO:0006953	3	109	0.0356249	acute-phase response
GO:0007159	3	109	0.0356249	leukocyte cell-cell adhesion
GO:0010950	4	351	0.0356739	positive regulation of endopeptidase activity
GO:0009615	5	761	0.0360409	response to virus
GO:0051259	6	1363	0.0399273	protein oligomerization
GO:0070486	2	14	0.0405948	leukocyte aggregation
GO:0007596	6	1367	0.0405981	blood coagulation
GO:0050817	6	1367	0.0405981	coagulation
GO:0032760	3	114	0.0407506	positive regulation of tumor necrosis factor production
GO:0055081	3	115	0.0418307	anion homeostasis
GO:0007599	6	1382	0.0431958	hemostasis
GO:0006275	4	373	0.0452753	regulation of DNA replication
GO:0002252	6	1396	0.0457405	immune effector process
GO:0032355	4	374	0.0457526	response to estradiol
GO:0006820	6	1397	0.0459269	anion transport
GO:0051712	2	15	0.0468279	positive regulation of killing of cells of other organism
GO:0051248	7	2189	0.049813	negative regulation of protein metabolic process

Table 5: Overrepresented terms with the network-based enrichment. Only terms not detected with the standard method.
