# Supplementary material for: NET-GE: a novel NETwork-based Gene Enrichment for detecting biological processes associated to Mendelian diseases
Source: BMC Genomics. 2015 Jun 18;16(Suppl 8):S6. doi: 10.1186/1471-2164-16-S8-S6 (PMC4480278; doi:10.1186/1471-2164-16-S8-S6)
Supplement: Additional file 3 — Detailed results for the OMIM-derived benchmark set. The archive contains pdf documents listing the enriched terms for each one of the 244 diseases in the OMIM-derived benchmark set. [file 1471-2164-16-S8-S6-S3.tgz › SUPPMAT/OMIM612052.pdf]

#612052 SMOKING AS A QUANTITATIVE TRAIT LOCUS 3; SQTL3

**OMIM Gene ID**	**HGNC**	**UniProtAC**
118503	CHRNA3	P32297
118505	CHRNA5	P30532

Table 1: OMIM - UniProtAC mapping

Legend

- N1: #input proteins associated to the significant GO term
- N2: #proteins associated to the significant GO term
- P-value: Bonferroni-corrected p-value of Fisher's exact test
- *red*: go terms not related to the input proteins
- *blue*: go terms related to the input proteins (enriched uniquely by network-based method)
- *green*: go terms ancestors of terms enriched with the standard method (enriched uniquely by network-based method)

1 Standard enrichment

GO Term	N1	N2	P-value	Description
GO:0035095	2	9	6.41911e-06	behavioral response to nicotine
GO:0035094	2	47	0.000192752	response to nicotine
GO:0043279	2	188	0.00313431	response to alkaloid
GO:0030534	2	222	0.00437408	adult behavior
GO:0060084	1	3	0.0201887	synaptic transmission involved in micturition
GO:0007268	2	530	0.0249961	synaptic transmission
GO:0044708	2	596	0.0316159	single-organism behavior

Table 2: Overrepresented GO terms with the standard enrichment

2 Network-based enrichment

GO Term	N1	N2	P-value	Description
GO:0007271	2	35	0.000182276	synaptic transmission, cholinergic
GO:0050879	2	38	0.000215361	multicellular organismal movement
GO:0050881	2	38	0.000215361	musculoskeletal movement
GO:0007274	2	59	0.000524158	neuromuscular synaptic transmission
GO:0050982	2	100	0.00151641	detection of mechanical stimulus
GO:0048747	2	123	0.00229852	muscle fiber development
GO:0042472	2	207	0.0065316	inner ear morphogenesis
GO:0055002	2	242	0.00893336	striated muscle cell development
GO:0055001	2	285	0.0123978	muscle cell development
GO:0050905	2	306	0.0142956	neuromuscular process
GO:0070588	2	353	0.0190327	calcium ion transmembrane transport
GO:0007204	2	371	0.0210261	positive regulation of cytosolic calcium ion concentration
GO:0051480	2	457	0.03192	cytosolic calcium ion homeostasis

Table 3: Overrepresented terms with the network-based enrichment. Only terms not detected with the standard method.
