# Supplementary material for: NET-GE: a novel NETwork-based Gene Enrichment for detecting biological processes associated to Mendelian diseases
Source: BMC Genomics. 2015 Jun 18;16(Suppl 8):S6. doi: 10.1186/1471-2164-16-S8-S6 (PMC4480278; doi:10.1186/1471-2164-16-S8-S6)
Supplement: Additional file 3 — Detailed results for the OMIM-derived benchmark set. The archive contains pdf documents listing the enriched terms for each one of the 244 diseases in the OMIM-derived benchmark set. [file 1471-2164-16-S8-S6-S3.tgz › SUPPMAT/OMIM612781.pdf]

#612781 ISOLATED GROWTH HORMONE DEFICIENCY, TYPE IB; IGHD1B

**OMIM Gene ID**	**HGNC**	**UniProtAC**
139191	GHRHR	Q02643
139250	GH1	P01241

Table 1: OMIM - UniProtAC mapping

Legend

- N1: #input proteins associated to the significant GO term
- N2: #proteins associated to the significant GO term
- P-value: Bonferroni-corrected p-value of Fisher's exact test
- *red*: go terms not related to the input proteins
- *blue*: go terms related to the input proteins (enriched uniquely by network-based method)
- *green*: go terms ancestors of terms enriched with the standard method (enriched uniquely by network-based method)

1 Standard enrichment

GO Term	N1	N2	P-value	Description
GO:0043568	2	16	3.77397e-05	positive regulation of insulin-like growth factor receptor signaling pathway
GO:0043567	2	26	0.000102212	regulation of insulin-like growth factor receptor signaling pathway
GO:0040018	2	61	0.00057553	positive regulation of multicellular organism growth
GO:0040014	2	123	0.00235966	regulation of multicellular organism growth
GO:0021700	2	238	0.00886977	developmental maturation
GO:0045927	2	244	0.00932357	positive regulation of growth
GO:0043627	2	278	0.0121091	response to estrogen
GO:0071375	2	372	0.0217022	cellular response to peptide hormone stimulus
GO:0060133	1	2	0.0237393	somatotropin secreting cell development
GO:0070977	1	2	0.0237393	bone maturation
GO:1901653	2	391	0.0239788	cellular response to peptide

Table 2: Overrepresented GO terms with the standard enrichment

2 Network-based enrichment

No novel enriched terms
