# Supplementary material for: NET-GE: a novel NETwork-based Gene Enrichment for detecting biological processes associated to Mendelian diseases
Source: BMC Genomics. 2015 Jun 18;16(Suppl 8):S6. doi: 10.1186/1471-2164-16-S8-S6 (PMC4480278; doi:10.1186/1471-2164-16-S8-S6)
Supplement: Additional file 3 — Detailed results for the OMIM-derived benchmark set. The archive contains pdf documents listing the enriched terms for each one of the 244 diseases in the OMIM-derived benchmark set. [file 1471-2164-16-S8-S6-S3.tgz › SUPPMAT/OMIM613090.pdf]

#613090 BARTTER SYNDROME, TYPE 4B

**OMIM Gene ID**	**HGNC**	**UniProtAC**
602023	CLCNKB	P51801
602024	CLCNKA	P51800

Table 1: OMIM - UniProtAC mapping

Legend

- N1: #input proteins associated to the significant GO term
- N2: #proteins associated to the significant GO term
- P-value: Bonferroni-corrected p-value of Fisher's exact test
- *red*: go terms not related to the input proteins
- *blue*: go terms related to the input proteins (enriched uniquely by network-based method)
- *green*: go terms ancestors of terms enriched with the standard method (enriched uniquely by network-based method)

1 Standard enrichment

GO Term	N1	N2	P-value	Description
GO:0007588	2	51	5.54934e-05	excretion
GO:1902476	2	120	0.000310763	chloride transmembrane transport
GO:0044070	2	137	0.000405471	regulation of anion transport
GO:0006821	2	155	0.000519461	chloride transport
GO:0098661	2	185	0.000740782	inorganic anion transmembrane transport
GO:0015698	2	249	0.00134385	inorganic anion transport
GO:0098656	2	320	0.00222147	anion transmembrane transport
GO:0034765	2	424	0.00390309	regulation of ion transmembrane transport
GO:0034762	2	437	0.00414637	regulation of transmembrane transport
GO:0043269	2	721	0.0112971	regulation of ion transport
GO:0006820	2	806	0.0141199	anion transport
GO:0098660	2	995	0.0215234	inorganic ion transmembrane transport

Table 2: Overrepresented GO terms with the standard enrichment

2 Network-based enrichment

No novel enriched terms
