# Supplementary material for: NET-GE: a novel NETwork-based Gene Enrichment for detecting biological processes associated to Mendelian diseases
Source: BMC Genomics. 2015 Jun 18;16(Suppl 8):S6. doi: 10.1186/1471-2164-16-S8-S6 (PMC4480278; doi:10.1186/1471-2164-16-S8-S6)
Supplement: Additional file 3 — Detailed results for the OMIM-derived benchmark set. The archive contains pdf documents listing the enriched terms for each one of the 244 diseases in the OMIM-derived benchmark set. [file 1471-2164-16-S8-S6-S3.tgz › SUPPMAT/OMIM613254.pdf]

#613254 TUBEROUS SCLEROSIS 2; TSC2

OMIM Gene ID	HGNC	UniProtAC
147570	IFNG	P01579
191092	TSC2	P49815

Table 1: OMIM - UniProtAC mapping

Legend

- N1: #input proteins associated to the significant GO term
- N2: #proteins associated to the significant GO term
- P-value: Bonferroni-corrected p-value of Fisher's exact test
- *red*: go terms not related to the input proteins
- *blue*: go terms related to the input proteins (enriched uniquely by network-based method)
- *green*: go terms ancestors of terms enriched with the standard method (enriched uniquely by network-based method)

1 Standard enrichment

GO Term	N1	N2	P-value	Description
GO:0007050	2	189	0.0143925	cell cycle arrest
GO:0032113	1	1	0.0305752	regulation of carbohydrate phosphatase activity
GO:0060549	1	1	0.0305752	regulation of fructose 1,6-bisphosphate 1-phosphatase activity
GO:0060550	1	1	0.0305752	positive regulation of fructose 1,6-bisphosphate 1-phosphatase activity
GO:0060551	1	1	0.0305752	regulation of fructose 1,6-bisphosphate metabolic process
GO:0060552	1	1	0.0305752	positive regulation of fructose 1,6-bisphosphate metabolic process
GO:0045786	2	343	0.0475154	negative regulation of cell cycle
GO:0006935	2	350	0.0494775	chemotaxis
GO:0042330	2	350	0.0494775	taxis

Table 2: Overrepresented GO terms with the standard enrichment

2 Network-based enrichment

GO Term	N1	N2	P-value	Description
GO:0045429	2	70	0.00458692	positive regulation of nitric oxide biosynthetic process
GO:0045428	2	117	0.0128889	regulation of nitric oxide biosynthetic process
GO:0002526	2	220	0.0457552	acute inflammatory response

Table 3: Overrepresented terms with the network-based enrichment. Only terms not detected with the standard method.
