# Supplementary material for: NET-GE: a novel NETwork-based Gene Enrichment for detecting biological processes associated to Mendelian diseases
Source: BMC Genomics. 2015 Jun 18;16(Suppl 8):S6. doi: 10.1186/1471-2164-16-S8-S6 (PMC4480278; doi:10.1186/1471-2164-16-S8-S6)
Supplement: Additional file 3 — Detailed results for the OMIM-derived benchmark set. The archive contains pdf documents listing the enriched terms for each one of the 244 diseases in the OMIM-derived benchmark set. [file 1471-2164-16-S8-S6-S3.tgz › SUPPMAT/OMIM613652.pdf]

#613652 C1q DEFICIENCY; C1QD

**OMIM Gene ID**	**HGNC**	**UniProtAC**
120550	C1QA	P02745
120570	C1QB	P02746
120575	C1QC	P02747

Table 1: OMIM - UniProtAC mapping

Legend

- N1: #input proteins associated to the significant GO term
- N2: #proteins associated to the significant GO term
- P-value: Bonferroni-corrected p-value of Fisher's exact test
- *red*: go terms not related to the input proteins
- *blue*: go terms related to the input proteins (enriched uniquely by network-based method)
- *green*: go terms ancestors of terms enriched with the standard method (enriched uniquely by network-based method)

1 Standard enrichment

GO Term	N1	N2	P-value	Description
GO:0006958	3	163	4.58651e-06	complement activation, classical pathway
GO:0006956	3	180	6.18727e-06	complement activation
GO:0072376	3	215	1.05727e-05	protein activation cascade
GO:0006959	3	294	2.71363e-05	humoral immune response
GO:0002253	3	566	0.00019458	activation of immune response
GO:0002252	3	657	0.000304553	immune effector process
GO:0050778	3	770	0.000490605	positive regulation of immune response
GO:0045087	3	825	0.000603577	innate immune response
GO:0002684	3	1093	0.00140482	positive regulation of immune system process
GO:0050776	3	1167	0.00171021	regulation of immune response
GO:0006955	3	1414	0.00304354	immune response
GO:0006952	3	1580	0.00424717	defense response
GO:0002682	3	1758	0.0058515	regulation of immune system process
GO:0048584	3	2308	0.0132463	positive regulation of response to stimulus
GO:0002376	3	2446	0.0157685	immune system process

Table 2: Overrepresented GO terms with the standard enrichment

2 Network-based enrichment

GO Term	N1	N2	P-value	Description
GO:0045638	2	261	0.00948886	negative regulation of myeloid cell differentiation
GO:0045637	2	596	0.0492524	regulation of myeloid cell differentiation

Table 3: Overrepresented terms with the network-based enrichment. Only terms not detected with the standard method.
