# Supplementary material for: NET-GE: a novel NETwork-based Gene Enrichment for detecting biological processes associated to Mendelian diseases
Source: BMC Genomics. 2015 Jun 18;16(Suppl 8):S6. doi: 10.1186/1471-2164-16-S8-S6 (PMC4480278; doi:10.1186/1471-2164-16-S8-S6)
Supplement: Additional file 3 — Detailed results for the OMIM-derived benchmark set. The archive contains pdf documents listing the enriched terms for each one of the 244 diseases in the OMIM-derived benchmark set. [file 1471-2164-16-S8-S6-S3.tgz › SUPPMAT/OMIM613659.pdf]

#613659 GASTRIC CANCER

OMIM Gene ID	HGNC	UniProtAC
147575	IRF1	P10914
164870	ERBB2	P04626
171834	PIK3CA	P42336
176943	FGFR2	P21802
601762	CASP10	Q92851
602053	KLF6	Q99612
604933	MUTYH	Q9UIF7
611731	APC	P25054

Table 1: OMIM - UniProtAC mapping

Legend

- N1: #input proteins associated to the significant GO term
- N2: #proteins associated to the significant GO term
- P-value: Bonferroni-corrected p-value of Fisher's exact test
- *red*: go terms not related to the input proteins
- *blue*: go terms related to the input proteins (enriched uniquely by network-based method)
- *green*: go terms ancestors of terms enriched with the standard method (enriched uniquely by network-based method)

1 Standard enrichment

GO Term	N1	N2	P-value	Description
GO:0002376	7	2446	2.99794e-05	immune system process
GO:0006950	7	4134	0.00113591	response to stress
GO:0048015	3	182	0.00506019	phosphatidylinositol-mediated signaling
GO:0048017	3	182	0.00506019	inositol lipid-mediated signaling
GO:0006952	5	1580	0.00536721	defense response
GO:0007173	3	202	0.00691602	epidermal growth factor receptor signaling pathway
GO:0038127	3	205	0.00722819	ERBB signaling pathway
GO:0008543	3	211	0.00788021	fibroblast growth factor receptor signaling pathway
GO:0010604	6	3285	0.00866042	positive regulation of macromolecule metabolic process
GO:0002682	5	1758	0.00904757	regulation of immune system process
GO:0044344	3	237	0.0111556	cellular response to fibroblast growth factor stimulus
GO:0006915	4	805	0.0111998	apoptotic process
GO:0048522	7	5768	0.0112193	positive regulation of cellular process
GO:0071774	3	243	0.012021	response to fibroblast growth factor
GO:0001775	4	825	0.0123359	cell activation
GO:0045087	4	825	0.0123359	innate immune response
GO:0051716	8	9450	0.0128518	cellular response to stimulus
GO:0012501	4	868	0.0150655	programmed cell death
GO:0030098	3	263	0.0152243	lymphocyte differentiation
GO:0009893	6	3630	0.015509	positive regulation of metabolic process
GO:0048011	3	276	0.017582	neurotrophin TRK receptor signaling pathway
GO:0008285	4	920	0.0189356	negative regulation of cell proliferation
GO:0038179	3	285	0.0193479	neurotrophin signaling pathway
GO:0038095	3	294	0.0212272	Fc-epsilon receptor signaling pathway
GO:1902105	3	321	0.0275785	regulation of leukocyte differentiation
GO:0048518	7	6624	0.0288915	positive regulation of biological process
GO:0045786	3	343	0.0335927	negative regulation of cell cycle
GO:0038093	3	350	0.035673	Fc receptor signaling pathway
GO:0008219	4	1106	0.0389626	cell death
GO:0050678	3	361	0.0391105	regulation of epithelial cell proliferation
GO:0016265	4	1117	0.0404995	death
GO:0002521	3	375	0.0437919	leukocyte differentiation
GO:0007528	2	53	0.0449359	neuromuscular junction development
GO:0045839	2	53	0.0449359	negative regulation of mitosis
GO:0030154	5	2446	0.0450526	cell differentiation
GO:0007166	6	4368	0.0454115	cell surface receptor signaling pathway
GO:0050863	3	385	0.0473521	regulation of T cell activation
GO:0050776	4	1167	0.0480551	regulation of immune response
GO:0071310	5	2482	0.0483483	cellular response to organic substance

Table 2: Overrepresented GO terms with the standard enrichment

2 Network-based enrichment

GO Term	N1	N2	P-value	Description
GO:0030155	6	1217	9.30698e-05	regulation of cell adhesion
GO:0045785	5	632	0.000194817	positive regulation of cell adhesion
GO:0097191	4	280	0.000502061	extrinsic apoptotic signaling pathway
GO:0050878	6	1837	0.00106935	regulation of body fluid levels
GO:0010629	7	3561	0.00173444	negative regulation of gene expression
GO:0050671	4	392	0.00191973	positive regulation of lymphocyte proliferation
GO:0032946	4	396	0.00199884	positive regulation of mononuclear cell proliferation
GO:0070665	4	403	0.00214308	positive regulation of leukocyte proliferation
GO:0021954	3	102	0.00232695	central nervous system neuron development
GO:0097190	5	1052	0.0024261	apoptotic signaling pathway
GO:0050731	4	503	0.00516617	positive regulation of peptidyl-tyrosine phosphorylation
GO:0016477	6	2574	0.00779285	cell migration
GO:2000027	4	574	0.0087135	regulation of organ morphogenesis
GO:1901652	5	1373	0.00898218	response to peptide
GO:0007599	5	1382	0.00927443	hemostasis
GO:0043065	5	1406	0.0100906	positive regulation of apoptotic process
GO:1901698	6	2693	0.0101561	response to nitrogen compound
GO:0043068	5	1416	0.010447	positive regulation of programmed cell death
GO:0050670	4	605	0.0107273	regulation of lymphocyte proliferation
GO:0032944	4	609	0.0110103	regulation of mononuclear cell proliferation
GO:0070663	4	621	0.0118924	regulation of leukocyte proliferation
GO:0048870	6	2808	0.0129727	cell motility
GO:0045321	5	1486	0.0132298	leukocyte activation
GO:0030278	4	644	0.0137286	regulation of ossification
GO:0050730	4	663	0.0153974	regulation of peptidyl-tyrosine phosphorylation
GO:0010942	5	1542	0.0158524	positive regulation of cell death
GO:0048534	4	682	0.0172123	hematopoietic or lymphoid organ development
GO:0051345	6	2953	0.017412	positive regulation of hydrolase activity
GO:0045944	6	2989	0.0186888	positive regulation of transcription from RNA polymerase II promoter
GO:0010948	4	717	0.0209644	negative regulation of cell cycle process
GO:0006955	6	3063	0.0215561	immune response
GO:0040011	6	3142	0.025007	locomotion
GO:0048812	4	780	0.0292017	neuron projection morphogenesis
GO:0045892	6	3247	0.0302855	negative regulation of transcription, DNA-templated
GO:1902679	6	3295	0.0329863	negative regulation of RNA biosynthetic process
GO:0043408	5	1837	0.0372099	regulation of MAPK cascade
GO:0051253	6	3369	0.0375358	negative regulation of RNA metabolic process
GO:0051251	4	874	0.0456505	positive regulation of lymphocyte activation
GO:0030334	5	1926	0.0468242	regulation of cell migration

Table 3: Overrepresented terms with the network-based enrichment. Only terms not detected with the standard method.
