# Supplementary material for: NET-GE: a novel NETwork-based Gene Enrichment for detecting biological processes associated to Mendelian diseases
Source: BMC Genomics. 2015 Jun 18;16(Suppl 8):S6. doi: 10.1186/1471-2164-16-S8-S6 (PMC4480278; doi:10.1186/1471-2164-16-S8-S6)
Supplement: Additional file 3 — Detailed results for the OMIM-derived benchmark set. The archive contains pdf documents listing the enriched terms for each one of the 244 diseases in the OMIM-derived benchmark set. [file 1471-2164-16-S8-S6-S3.tgz › SUPPMAT/OMIM613688.pdf]

#613688 LONG QT SYNDROME 2; LQT2

OMIM Gene ID	HGNC	UniProtAC
152427	KCNH2	Q12809
603313	ALG10	Q5BKT4

Table 1: OMIM - UniProtAC mapping

Legend

- N1: #input proteins associated to the significant GO term
- N2: #proteins associated to the significant GO term
- P-value: Bonferroni-corrected p-value of Fisher's exact test
- *red*: go terms not related to the input proteins
- *blue*: go terms related to the input proteins (enriched uniquely by network-based method)
- *green*: go terms ancestors of terms enriched with the standard method (enriched uniquely by network-based method)

1 Standard enrichment

GO Term	N1	N2	P-value	Description
GO:1902302	1	1	0.00694169	regulation of potassium ion export
GO:1902303	1	1	0.00694169	negative regulation of potassium ion export
GO:0003064	1	2	0.0138832	regulation of heart rate by hormone
GO:0003062	1	7	0.0485879	regulation of heart rate by chemical signal
GO:1901380	1	7	0.0485879	negative regulation of potassium ion transmembrane transport

Table 2: Overrepresented GO terms with the standard enrichment

2 Network-based enrichment

GO Term	N1	N2	P-value	Description
GO:0006487	2	174	0.00417685	protein N-linked glycosylation

Table 3: Overrepresented terms with the network-based enrichment. Only terms not detected with the standard method.
