# Supplementary material for: NET-GE: a novel NETwork-based Gene Enrichment for detecting biological processes associated to Mendelian diseases
Source: BMC Genomics. 2015 Jun 18;16(Suppl 8):S6. doi: 10.1186/1471-2164-16-S8-S6 (PMC4480278; doi:10.1186/1471-2164-16-S8-S6)
Supplement: Additional file 3 — Detailed results for the OMIM-derived benchmark set. The archive contains pdf documents listing the enriched terms for each one of the 244 diseases in the OMIM-derived benchmark set. [file 1471-2164-16-S8-S6-S3.tgz › SUPPMAT/OMIM614279.pdf]

#614279 46,XY SEX REVERSAL 8; SRXY8

OMIM Gene ID	HGNC	UniProtAC
600450	AKR1C2	P52895
600451	AKR1C4	P17516

Table 1: OMIM - UniProtAC mapping

Legend

- N1: #input proteins associated to the significant GO term
- N2: #proteins associated to the significant GO term
- P-value: Bonferroni-corrected p-value of Fisher's exact test
- *red*: go terms not related to the input proteins
- *blue*: go terms related to the input proteins (enriched uniquely by network-based method)
- *green*: go terms ancestors of terms enriched with the standard method (enriched uniquely by network-based method)

1 Standard enrichment

GO Term	N1	N2	P-value	Description
GO:0009753	2	4	1.1204e-06	response to jasmonic acid
GO:0071395	2	4	1.1204e-06	cellular response to jasmonic acid stimulus
GO:0030638	2	8	5.22851e-06	polyketide metabolic process
GO:0044597	2	8	5.22851e-06	daunorubicin metabolic process
GO:0044598	2	8	5.22851e-06	doxorubicin metabolic process
GO:0030647	2	10	8.40297e-06	aminoglycoside antibiotic metabolic process
GO:0016137	2	18	2.85701e-05	glycoside metabolic process
GO:1901661	2	43	0.00016862	quinone metabolic process
GO:0019748	2	61	0.000341721	secondary metabolic process
GO:0042180	2	93	0.000798842	cellular ketone metabolic process
GO:0034754	2	133	0.00163915	cellular hormone metabolic process
GO:0042445	2	231	0.00496055	hormone metabolic process
GO:0071229	2	233	0.005047	cellular response to acid chemical
GO:0008202	2	325	0.00983148	steroid metabolic process
GO:0010817	2	337	0.0105721	regulation of hormone levels
GO:0001101	2	456	0.0193716	response to acid chemical
GO:0051186	2	461	0.0197993	cofactor metabolic process
GO:0032870	2	716	0.0477979	cellular response to hormone stimulus

Table 2: Overrepresented GO terms with the standard enrichment

2 Network-based enrichment

No novel enriched terms
