# Supplementary material for: NET-GE: a novel NETwork-based Gene Enrichment for detecting biological processes associated to Mendelian diseases
Source: BMC Genomics. 2015 Jun 18;16(Suppl 8):S6. doi: 10.1186/1471-2164-16-S8-S6 (PMC4480278; doi:10.1186/1471-2164-16-S8-S6)
Supplement: Additional file 3 — Detailed results for the OMIM-derived benchmark set. The archive contains pdf documents listing the enriched terms for each one of the 244 diseases in the OMIM-derived benchmark set. [file 1471-2164-16-S8-S6-S3.tgz › SUPPMAT/OMIM614286.pdf]

#614286 MYELODYSPLASTIC SYNDROME; MDS

**OMIM Gene ID**	**HGNC**	**UniProtAC**
137295	GATA2	P23769
605590	SF3B1	O75533
612839	TET2	Q6N021
612990	ASXL1	Q8IXJ9

Table 1: OMIM - UniProtAC mapping

Legend

- N1: #input proteins associated to the significant GO term
- N2: #proteins associated to the significant GO term
- P-value: Bonferroni-corrected p-value of Fisher's exact test
- *red*: go terms not related to the input proteins
- *blue*: go terms related to the input proteins (enriched uniquely by network-based method)
- *green*: go terms ancestors of terms enriched with the standard method (enriched uniquely by network-based method)

1 Standard enrichment

GO Term	N1	N2	P-value	Description
GO:0045599	2	72	0.00652949	negative regulation of fat cell differentiation
GO:0045598	2	144	0.026235	regulation of fat cell differentiation
GO:0006211	1	1	0.0322179	5-methylcytosine catabolic process
GO:0019857	1	1	0.0322179	5-methylcytosine metabolic process
GO:0045944	3	1312	0.0496386	positive regulation of transcription from RNA polymerase II promoter

Table 2: Overrepresented GO terms with the standard enrichment

2 Network-based enrichment

No novel enriched terms
