# Supplementary material for: NET-GE: a novel NETwork-based Gene Enrichment for detecting biological processes associated to Mendelian diseases
Source: BMC Genomics. 2015 Jun 18;16(Suppl 8):S6. doi: 10.1186/1471-2164-16-S8-S6 (PMC4480278; doi:10.1186/1471-2164-16-S8-S6)
Supplement: Additional file 3 — Detailed results for the OMIM-derived benchmark set. The archive contains pdf documents listing the enriched terms for each one of the 244 diseases in the OMIM-derived benchmark set. [file 1471-2164-16-S8-S6-S3.tgz › SUPPMAT/OMIM614519.pdf]

#614519 HEMORRHAGE, INTRACEREBRAL, SUSCEPTIBILITY TO; ICH

OMIM Gene ID	HGNC	UniProtAC
106180	ACE	P12821
120090	COL4A2	P08572
120130	COL4A1	P02462

Table 1: OMIM - UniProtAC mapping

Legend

- N1: #input proteins associated to the significant GO term
- N2: #proteins associated to the significant GO term
- P-value: Bonferroni-corrected p-value of Fisher's exact test
- *red*: go terms not related to the input proteins
- *blue*: go terms related to the input proteins (enriched uniquely by network-based method)
- *green*: go terms ancestors of terms enriched with the standard method (enriched uniquely by network-based method)

1 Standard enrichment

GO Term	N1	N2	P-value	Description
GO:0044243	3	84	1.93539e-06	multicellular organismal catabolic process
GO:0044259	3	105	3.80764e-06	multicellular organismal macromolecule metabolic process
GO:0044236	3	112	4.62946e-06	multicellular organismal metabolic process
GO:0030574	2	78	0.00229897	collagen catabolic process
GO:0032963	2	96	0.00348983	collagen metabolic process
GO:0022617	2	117	0.0051915	extracellular matrix disassembly
GO:0044712	3	2063	0.0296798	single-organism catabolic process
GO:0002005	1	3	0.0433964	angiotensin catabolic process in blood
GO:0002019	1	3	0.0433964	regulation of renal output by angiotensin
GO:0044254	1	3	0.0433964	multicellular organismal protein catabolic process
GO:0044266	1	3	0.0433964	multicellular organismal macromolecule catabolic process
GO:0030154	3	2446	0.0494803	cell differentiation

Table 2: Overrepresented GO terms with the standard enrichment

2 Network-based enrichment

No novel enriched terms
