# Supplementary material for: NET-GE: a novel NETwork-based Gene Enrichment for detecting biological processes associated to Mendelian diseases
Source: BMC Genomics. 2015 Jun 18;16(Suppl 8):S6. doi: 10.1186/1471-2164-16-S8-S6 (PMC4480278; doi:10.1186/1471-2164-16-S8-S6)
Supplement: Additional file 3 — Detailed results for the OMIM-derived benchmark set. The archive contains pdf documents listing the enriched terms for each one of the 244 diseases in the OMIM-derived benchmark set. [file 1471-2164-16-S8-S6-S3.tgz › SUPPMAT/OMIM615489.pdf]

#615489 MACULAR DEGENERATION, AGE-RELATED, 14; ARMD14

OMIM Gene ID	HGNC	UniProtAC
138470	CFB	P00751
613927	C2	P06681

Table 1: OMIM - UniProtAC mapping

Legend

- N1: #input proteins associated to the significant GO term
- N2: #proteins associated to the significant GO term
- P-value: Bonferroni-corrected p-value of Fisher's exact test
- *red*: go terms not related to the input proteins
- *blue*: go terms related to the input proteins (enriched uniquely by network-based method)
- *green*: go terms ancestors of terms enriched with the standard method (enriched uniquely by network-based method)

1 Standard enrichment

GO Term	N1	N2	P-value	Description
GO:0030449	2	38	6.0208e-05	regulation of complement activation
GO:2000257	2	40	6.68023e-05	regulation of protein activation cascade
GO:0002920	2	61	0.000156729	regulation of humoral immune response
GO:0002673	2	91	0.000350713	regulation of acute inflammatory response
GO:0006956	2	180	0.00137973	complement activation
GO:0072376	2	215	0.00197025	protein activation cascade
GO:0070613	2	278	0.00329756	regulation of protein processing
GO:1903317	2	278	0.00329756	regulation of protein maturation
GO:0006959	2	294	0.00368879	humoral immune response
GO:0050727	2	372	0.00590997	regulation of inflammatory response
GO:0002697	2	463	0.00915994	regulation of immune effector process
GO:1903034	2	529	0.0119608	regulation of response to wounding
GO:0002253	2	566	0.0136941	activation of immune response
GO:0002252	2	657	0.018456	immune effector process
GO:2000427	1	6	0.0193931	positive regulation of apoptotic cell clearance
GO:0031347	2	706	0.0213139	regulation of defense response
GO:0050778	2	770	0.0253563	positive regulation of immune response
GO:0032101	2	825	0.0291105	regulation of response to external stimulus
GO:0045087	2	825	0.0291105	innate immune response
GO:0006957	1	13	0.0420144	complement activation, alternative pathway
GO:2000425	1	13	0.0420144	regulation of apoptotic cell clearance

Table 2: Overrepresented GO terms with the standard enrichment

2 Network-based enrichment

No novel enriched terms
